# Copper‐Catalyzed Highly Enantioselective Addition of a Silicon Nucleophile to 3‐Substituted 2*H*‐Azirines Using an Si−B Reagent

**DOI:** 10.1002/anie.202215032

**Published:** 2023-01-09

**Authors:** Zhi‐Yuan Zhao, Ming Cui, Elisabeth Irran, Martin Oestreich

**Affiliations:** ^1^ Institut für Chemie Technische Universität Berlin Strasse des 17. Juni 115 10623 Berlin Germany

**Keywords:** Asymmetric Catalysis, Aziridines, Copper, Nucleophilic Addition, Silicon

## Abstract

3‐Substituted 2*H*‐azirines can be considered strained cyclic ketimines, and highly enantioselective addition reactions of silicon nucleophiles to either acyclic or cyclic ketimines have been elusive so far. The present work closes this gap for those azirines by means of a copper‐catalyzed silylation using a silyl boronic ester as a latent silicon nucleophile. The resulting *C*‐silylated, unprotected (N−H) aziridines are obtained in high yields and with excellent enantioselectivities and can be further converted into valuable compounds with hardly any erosion of the enantiomeric excess.

## Graphical abstract

The first catalytic asymmetric synthesis of *C*‐silylated, unprotected aziridines has been accomplished. Using a silyl boronic ester as a latent silicon nucleophile, a chiral copper catalyst enables its addition of 3‐substituted 2*H*‐azirines in high yields and with excellent enantioselectivities. These azirines can be considered strained cyclic and hence reactive ketimines, for which an effective silylation is still not known.

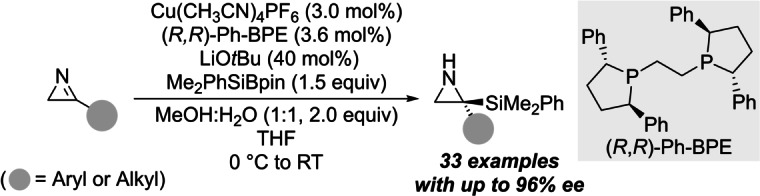

Aziridines are an important class of small‐ring heterocycles, either employed as synthetic building blocks^[1]^ or found as key motifs in bioactive natural products as well as pharmaceuticals.^[2]^ From a variety of known methods of their stereoselective preparation,^[1]^ nucleophilic addition to an existing unsaturated, nitrogen‐containing three‐membered ring is a viable possibility.^[3]^ By this, 2*H*‐azirines are converted into unprotected (N−H) aziridines with carbon^[4]^ as well as heteroatom nucleophiles.[^5^, ^6^] A related enantioselective silylation has not been described yet, perhaps also because of the propensity of *C*‐silylated aziridines to form azirine‐derived products by desilylative elimination.[^1h^, ^7^] Previously reported approaches to *N*‐protected aziridines decorated with a silyl group mainly rely on their uncatalyzed (directed) metalation followed by the addition of a silicon electrophile (Scheme 1, top).[^8^, ^9^] Conversely, catalytic asymmetric strategies are scarce with a copper‐catalyzed (2+1) cycloaddition of trimethylsilyl diazomethane and imines by Jørgensen being essentially the only general example.[^7^, ^10^] Also, Ooi kinetically resolved a racemic mixture of a *C*‐silylated aziridine by an organocatalytic ring opening.^[11]^

**Scheme 1:**
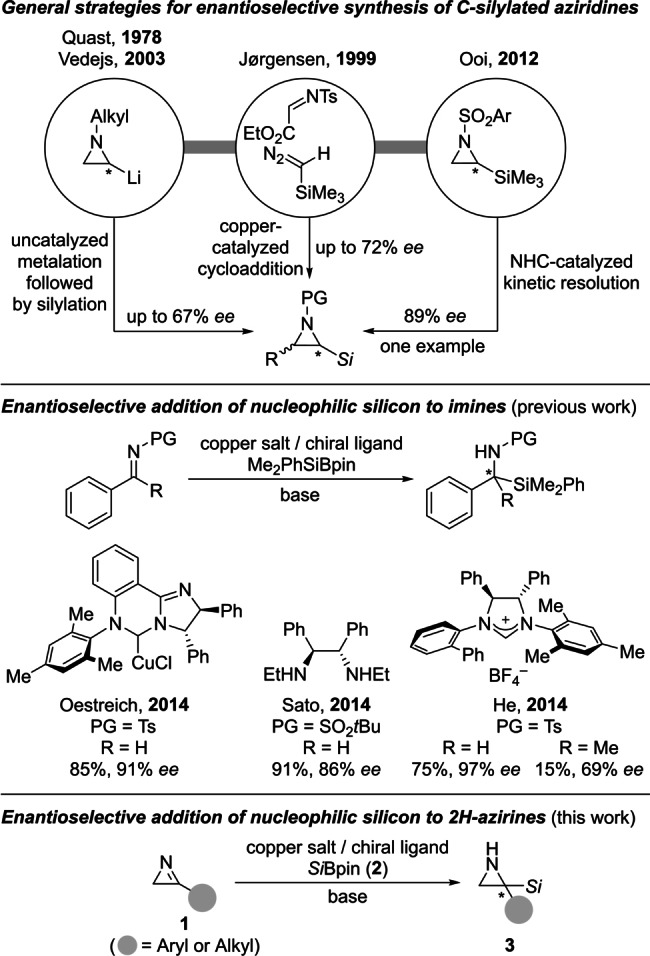
Enantioselective synthesis of *C*‐silylated aziridines and addition of silicon nucleophiles to C=N bonds. PG=protective group, *Si*=triorganosilyl, Ts=4‐toluenesulfonyl.

2*H*‐Azirines can be viewed as strained cyclic imines, and its 3‐substituted derivatives are basically highly reactive ketimines. We^[12a]^ and independently the laboratories of Sato^[12b]^ and He^[12c]^ developed copper‐catalyzed enantioselective addition reactions to aldimines with Si−B reagents^[13]^ as the source of the silicon nucleophiles (Scheme 1, middle).^[12]^ However, it was only the catalyst system introduced by He and co‐workers that enabled the silylation of less reactive ketimines in low yields but with promising levels of enantioselection.^[12c]^ Based on these advances, we decided to tackle the challenge of an enantioselective silylation of those 3‐substituted 2*H*‐azirines **1** for the synthesis of *C*‐silylated, unprotected aziridines **3** (Scheme 1, bottom).^[14]^

We initially attempted to apply the optimal reaction conditions previously reported for the silylation of aldimines[^12a^, ^15^] to 3‐phenyl‐substituted 2*H*‐azirine **1 a** (cf. Scheme 1, middle left). We knew that McQuade's preformed NHC‐copper(I) complex is difficult to prepare in high purity^[16]^ and that those reactions are especially sensitive towards the quality of the base used. This was confirmed by the following experiments with 5.0 mol % of McQuade's catalyst, 1.5 equiv of NaOMe, and 1.5 equiv of Me_2_PhSiBpin (**2 a**) in Et_2_O at 0 °C to room temperature. While NaOMe stored outside a glovebox led to (*R*)‐**3 aa** in 95 % NMR yield and with 93 % *ee*, a batch of NaOMe from a glovebox did not change the yield but furnished merely 81 % *ee*. We therefore abandoned this protocol and moved on with screening other catalyst systems (Table 1; see the Supporting Information for detailed optimization data). Gratifyingly, the *C*‐silylated aziridine **3 aa** was obtained in 85 % yield and with excellent 95 % *ee* in the presence of 3.0 mol % of Cu(CH_3_CN)_4_PF_6_ and 3.6 mol % of (*R*,*R*)‐Ph‐BPE (**L1**) in THF at room temperature (entry 1).^[17]^ Other copper salts were inferior with regard to both yield and enantioinduction (entries 2–4). The solvent also had an influence with decent performance of the catalyst in Et_2_O and toluene but severely deteriorated yield and enantioselection in 2‐Me‐THF (entries 5–7). The alkoxide base LiO*t*Bu could be replaced by LiOMe with no effect on the reaction outcome (entry 8); LiOMe was later used with several other substrates as enantiomeric excesses were occasionally higher than with LiO*t*Bu.

**Table 1:** Selected examples of the optimization of the copper‐catalyzed silylation of 2*H*‐azirines.^[a]^

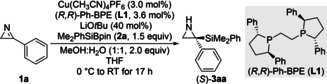			
Entry	Variation	Yield [%]^[b]^	ee [%]^[c]^
1^[d]^	none	98 (85)^[e]^	95
2	(Ph_3_P)_2_CuBH_4_ instead of Cu(CH_3_CN)_4_PF_6_	79	88
3	CuCN instead of Cu(CH_3_CN)_4_PF_6_	31	21
4	Cu(acac)_2_ instead of Cu(CH_3_CN)_4_PF_6_	45	38
5	Et_2_O instead of THF	85	87
6	toluene instead of THF	92	89
7	2‐Me‐THF instead of THF	46	31
8	LiOMe instead of LiO*t*Bu	96	95

[a] All reactions were performed on a 0.20 mmol scale; MeOH was used as the proton source except in entries 1 and 8. [b] Determined by ^1^H NMR spectroscopy with CH_2_Br_2_ as an internal standard. [c] Determined by HPLC analysis on a chiral stationary phase. [d] 93 % yield and 92 % *ee* were obtained on a 1.0‐mmol scale. [e] Isolated yield after purification by flash chromatography on silica gel.

With the optimized reaction conditions in hand, the substrate scope was tested with different aryl and alkyl groups at C3 of the 2*H*‐azirine (Scheme 2).^[18]^ Azirines **1 b**–**f** bearing alkyl‐substituted aryl groups led to reaction outcomes similar to parent **1 a**; **1 g** with a phthalimidomethyl group in the *para* position converted into **3 ga** in 95 % yield and with 92 % *ee*, already demonstrating superb functional‐group tolerance. Likewise, a [1,1′‐biphenyl]‐4‐yl group as in **1 h** and a naphth‐2‐yl group as in **1 i** instead of the phenyl substituent were perfectly compatible. Electron‐donating groups such as methoxy and benzyloxy as in **1 j**–**m** as well as a catechol protected as a methylene acetal as in **1 n** had no influence on yield and enantioselectivity; a thioether as in **1 o** was equally tolerated. Similarly, an acetyloxy group in **1 p** and a trifluoromethoxy group in **1 q** did not interfere. A trifluoromethyl group directly attached to the aryl ring as in **1 r** was not detrimental, and cyano (**1 s**) as well as acetyl (**1 t**) as other electron‐withdrawing groups were also accepted. Aryl‐substituted azirines **1 u**–**z** with monohalogenation in the *para* or *meta* position provided the corresponding *C*‐silylated aziridines in good yields and with high enantioselectivities throughout. The dihalogenated derivatives **1 a′** and **1 b′** with one *ortho* substituent showed a trend towards lower enantioinduction with increasing size of the halogen atom (F in **3 a′a** with 86 % *ee* and Cl in **3 b′a** with 50 % *ee*). As an example of a heteroaryl group, a thienyl group as in **1 c′** was included into the survey but the *ee* value of 77 % for **3 c′a** was low in comparison. A few 3‐alkyl‐substituted 2*H*‐azirines (gray box) showed that the enantioinduction is slightly diminished for a 2° alkyl group (80 % *ee* for **3 d′a**) but remains good for 1° alkyl residues, even containing a primary C(sp^3^)−Cl bond (87 % and 85 % *ee* for **3 e′a**–**3 g′a**). The absolute configurations of **3 na** and **3 pa** were assigned as *S* by X‐ray diffraction, and the molecular structures are depicted in Scheme 2.

**Scheme 2:**
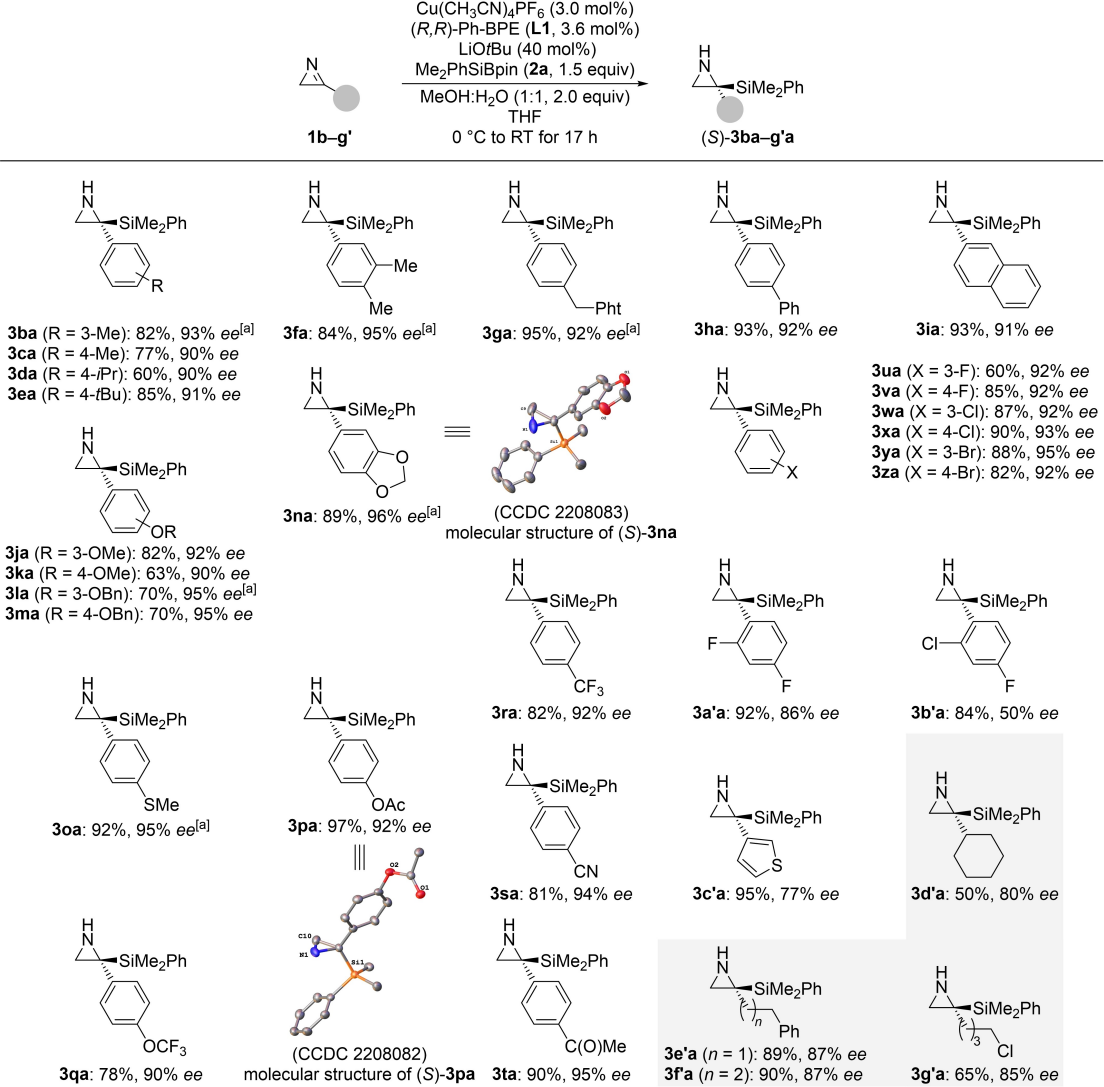
Scope I: Variation of the electrophile. All reactions were performed on a 0.20 mmol scale. Yields are isolated after purification by flash chromatography on silica gel. Enantiomeric excesses were determined by HPLC analysis on chiral stationary phases. [a] LiOMe instead of LiO*t*Bu at −10 °C. Ac=acetyl, Bn=benzyl, Pht=phthalimido.

To see whether the new procedure extends to imines other than 2*H*‐azirines **1**, we probed less strained cyclic ketimine **4** as well as acyclic *N*‐methyl‐substituted aldimine **5** and ketimine **6** (Figure 1). Neither of these electrophiles did react in the desired way. This underscores that the high reactivity of the C=N bond in 2*H*‐azirines **1** is a requirement. We also subjected other Si−B reagents **2 b**–**d** to the general protocol with **1 a** as the substrate but only Et_3_SiBpin (**2 b**) afforded **3 ab** in 84 % yield with 64 % *ee* (Figure 2). The silyl boronic esters **2 c** and **2 d** with bulkier silyl groups did not engage in the addition reaction, and there was no conversion of the starting materials.

**Figure 1:**
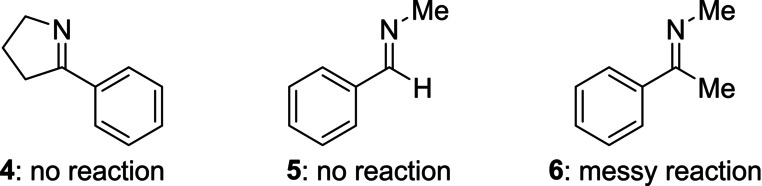
Scope II: Control experiments with representative imines.

**Figure 2:**
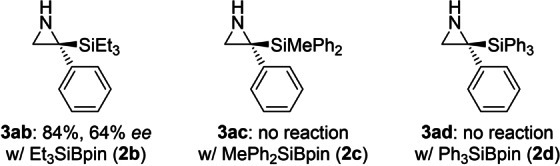
Scope III: Variation of the nucleophile. See footnote of Scheme 2.

To showcase the synthetic value of these chiral aziridines, we performed two transformations of (*S*)‐**3 aa** (Scheme 3). Unprotected (*S*)‐**3 aa** was acylated with 3,5‐dinitrobenzoyl chloride and then treated with Lawesson's reagent (top). Nakamura and co‐workers had shown before that this induces a rearrangement of the amide (not shown) to yield the oxazoline derivative (*S*)‐**7** with retention of the configuration.^[5c]^ Also, the sulfonamide (*S*)‐**8** was hydrogenated to give the β‐silylated amine derivative (*S*)‐**9** in 95 % yield with 90 % *ee* (bottom).^[9e]^

**Scheme 3:**
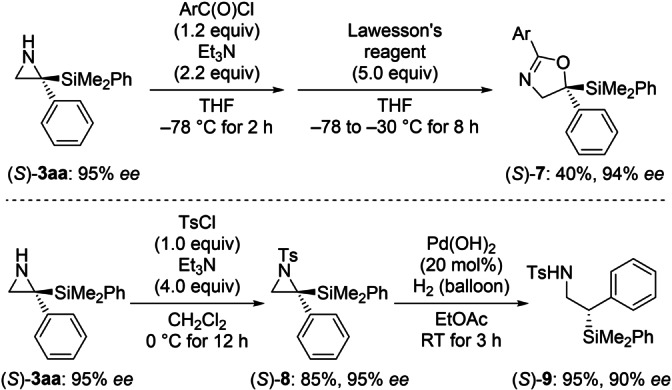
A *C*‐silylated, unprotected aziridine as a versatile building block. Ar=3,5‐dinitrophenyl.

To delineate the enantioinduction of the (*R*,*R*)‐Ph‐BPE‐copper(I) catalyst, two plausible transition states are proposed based on an established model for chiral complexes of copper(I) and (*R*,*R*)‐Ph‐BPE (**L1**) (Figure 3).^[19]^ Accordingly, one of the empty quadrants of the chiral catalyst's pocket can accommodate the substituent at C3 of the 2*H*‐azirine (left) while a less favored transition state would suffer from steric interactions between that substituent and a phenyl group of the ligand backbone (right). This is in agreement with *S* being the induced absolute configuration. The model also helps understanding why bulkier silyl groups are not transferred and it also suggests that the transition states are perhaps less compact with no π‐π stacking interactions for the triethylsilyl group (see Figure 2).

**Figure 3:**
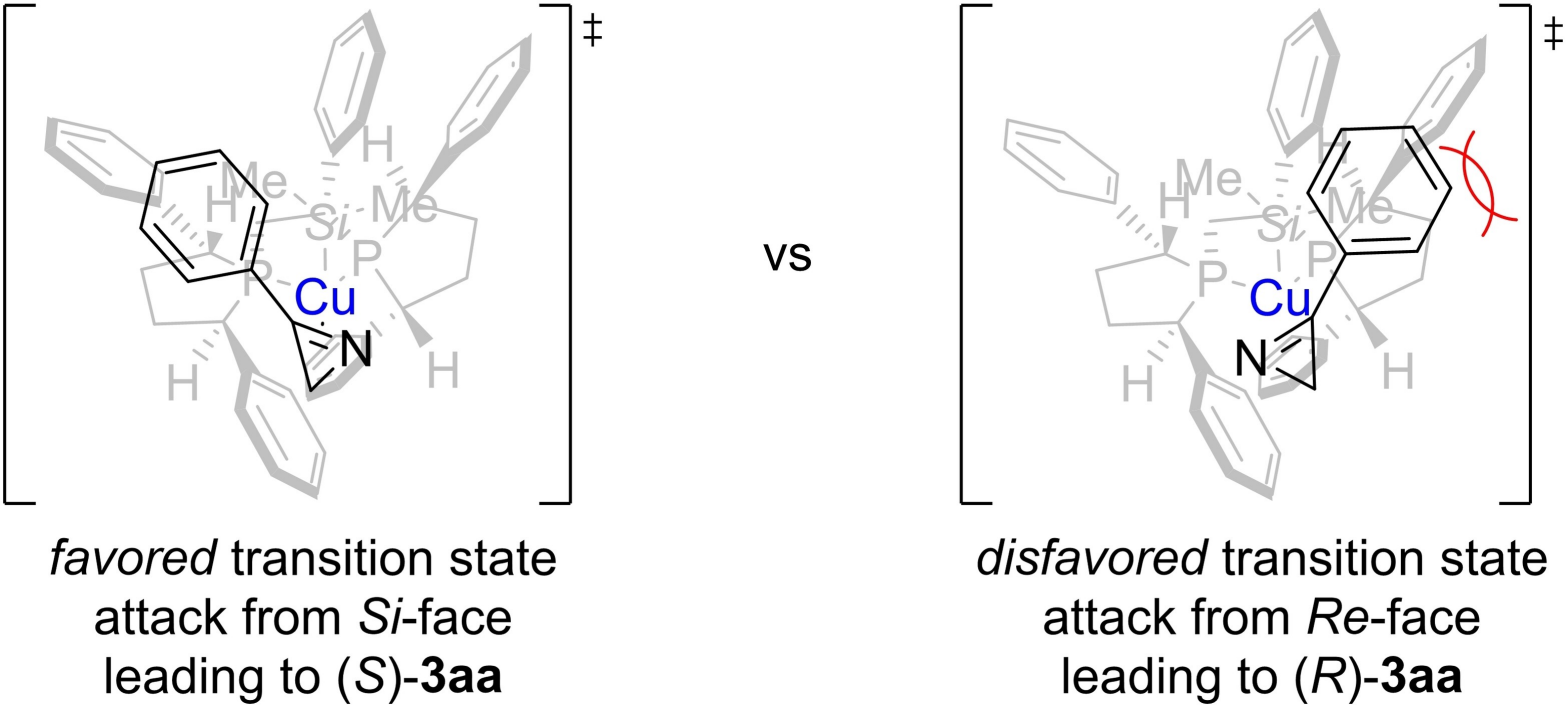
Proposed mechanistic model with dimethyl(phenyl)silyl group (copper in blue).

In summary, we described here a catalytic asymmetric access to *C*‐silylated, *N*‐unprotected aziridines with consistently high levels of enantioinduction. This was achieved by an enantioselective copper‐catalyzed silylation of 3‐substituted 2*H*‐azirines with a silyl boronic ester as a silicon pronucleophile. Unlike other cyclic and acyclic ketimines, these strained cyclic ketimines are sufficiently reactive, that is electrophilic, to engage in this addition reaction.

## Conflict of interest

The authors declare no conflict of interest.

## Supporting information

As a service to our authors and readers, this journal provides supporting information supplied by the authors. Such materials are peer reviewed and may be re‐organized for online delivery, but are not copy‐edited or typeset. Technical support issues arising from supporting information (other than missing files) should be addressed to the authors.

Supporting Information

Supporting Information

Supporting Information

Supporting Information

Supporting Information

## Data Availability

The data that support the findings of this study are available from the corresponding author upon reasonable request.
