# Supplementary material for: Copper‐Catalyzed Highly Enantioselective Addition of a Silicon Nucleophile to 3‐Substituted 2H‐Azirines Using an Si−B Reagent
Source: Angew Chem Int Ed Engl. 2023 Jan 9;62(7):e202215032. doi: 10.1002/anie.202215032 (PMC10108078; doi:10.1002/anie.202215032)
Supplement: Supplementary file 1 — Supporting Information [file ANIE-62-0-s004.pdf]

Angewandte Chemie
Eine Zeitschrift der Gesellschaft Deutscher Chemiker

Supporting Information

Copper-Catalyzed Highly Enantioselective Addition of a Silicon Nucleophile to 3-Substituted 2*H*-Azirines Using an Si–B Reagent

*Z.-Y. Zhao, M. Cui, E. Irran, M. Oestreich**

Supporting Information

Copper-Catalyzed Highly Enantioselective Addition of a Silicon Nucleophile to 3-Substituted 2*H*-Azirines Using an Si–B Reagent

*Zhi-Yuan Zhao, Ming Cui, Elisabeth Irran, and Martin Oestreich**

Table of Contents

1 General Information 3

2 Optimization Study 5

3 General Procedures 13

4 Experimental Details and Characterization Data for Starting Materials 20

5 Experimental Details and Characterization Data for Chiral Silylaziridines 26

6 Large-Scale Synthesis of (*S*)-**3aa** 60

7 Transformations of (*S*)-**3aa** 61

8 Determination of the Absolute Configuration 66

9 HPLC Traces 78

10 NMR Spectra 115

11 References 256

1 General Information

All reactions were performed in flame-dried glassware using conventional Schlenk techniques under a static pressure of nitrogen unless stated otherwise. Liquids and solutions were transferred with syringes. All metal salts were purchased from commercial suppliers and used as received. All solvents (CH_2Cl_2, toluene, Et_2O, MeOH, and THF *et al.*) were dried and purified following standard procedures. Technical grade solvents for extraction or chromatography (*n*-hexane, CH_2Cl_2, ethanol, ethyl acetate, and *n*-pentane *et al.*) were distilled prior to use. Analytical thin layer chromatography (TLC) was performed on ALUGRAM® Xtra SIL G/UV_{254} TLC-Sheets by Macherey-Nagel. Flash column chromatography was performed on silica gel 60 (40-63 µm, 230-400 mesh, ASTM) by Grace using the indicated solvents. ^{1}H, ^{13}C, ^{19}F, and ^{29}Si NMR spectra were recorded in $CDCl_3$ on Bruker AV400 or AV500 instruments. Chemical shifts were reported in parts per million (ppm) and were referenced to the residual solvent resonance as the internal standard ($CHCl_3$: δ = 7.26 ppm for ^{1}H NMR and $CDCl_3$: δ = 77.00 ppm for ^{13}C NMR). All other nuclei (^{19}F and ^{29}Si) were referenced in compliance with the unified scale for NMR chemical shifts as recommended by the IUPAC stating the chemical shift relative to $BF_3 \cdot Et_2O$, CCl_3F, and Me_4Si.[S1] Data were reported as follows: chemical shift, multiplicity (s = singlet, d = doublet, t = triplet, q = quartet, sept = septet, m = multiplet), coupling constants (Hz), and integration. Gas liquid chromatography (GLC) was performed on an *Agilent Technologies 7820A* gas chromatograph equipped with a HP-5 capillary column (30 m × 0.32 mm, 0.25 µm film thickness) by *Agilent Technologies/CS-Chromatographie Service* using the following program: N_2 carrier gas, injection temperature 250 °C, detector temperature 300 °C, flow rate: 1.7 mL/min; temperature program: start temperature 40 °C, heating rate 10 °C/min, end temperature 280 °C for 10 min. Infrared (IR) spectra were recorded on an *Agilent Technologies Cary* 630 FT-IR spectrometer equipped with an ATR unit and the signals were reported in wave-numbers (cm^{-1}). Melting points (m.p.) were

determined with a Stuart Scientific SMP20 melting point apparatus and were not corrected. Enantiomeric excesses were determined by analytical high performance liquid chromatography (HPLC) analysis on an *Agilent Technologies* 1290 Infinity instrument with a chiral stationary phase using a *Daicel* Chiralcel AD-H column*, Daicel* Chiralcel OD-H column, *Daicel* Chiralcel IA, or a *Daicel* Chiralcel IC column (*n*heptane/*iso*propanol mixtures as solvent). Data for the single crystal structure determination were collected with an *Agilent SuperNova* diffractometer equipped with a CCD area Atlas detector and a mirror monochromator by utilizing Cu-K_{α} radiation (λ = 1.5418 Å). Software packages used: CrysAlis PRO for data collection, cell refinement, and data reduction,[S2] SHELXS-97 for structure solution,[S3] SHELXL-97 for structure refinement[S4], and Mercury 3.1.1[S5] for graphic. Single crystals are presented by Olex2-1.5. High-resolution mass spectra (HRMS) were obtained from the Analytical Facility at the *Institut für Chemie, Technische Universität Berlin* on a Thermo Fisher Scientific LTQ Orbitrap XL apparatus using APCI/LIFDI techniques with a linear ion trap analyzer. Optical rotations were measured on a *Schmidt & Haensch Polartronic* H532 Polarimeter with $[\alpha]_{\lambda}$values reported in 10^{-1} (° cm^2 g^{-1}); with the concentration *c* in g/100 mL and λ indicated.

2 Optimization Study

Table S1. Ligand screening.

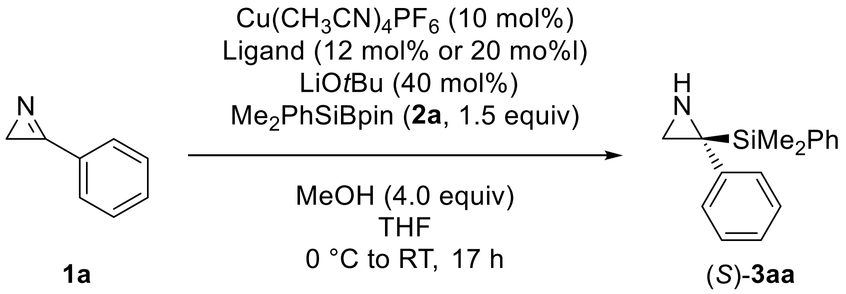

Entry[a]	Ligand	Yield/%[b]	*ee*/%[c]
1	**L1**	95	91
2	**L2**	70	56
3	**L3**	52	62
4	**L4**	90	12
5	**L5**	80	−10
6	**L6**	93	4
7	**L7**	87	87
8	**L8**	50	−27
9	**L9**	11	−18
10	**L10**	14	9
11	**L11**	trace	n.d.
12	**L12**	40	40
13	**L13**	80	−13
14	**L14**	50	−19
15	**L15**	35	52
16	**L16**	trace	n.d.
17	**L17**	40	17
18	**L18**	trace	n.d.

[a] All reactions were performed on a 0.20 mmol scale. [b] Yield was determined by ^{1}H NMR analysis with CH_2Br_2 as an internal standard. [c] Determined by HPLC analysis on a chiral stationary phase. n.d. = not detected.

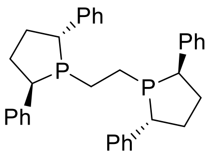

L1

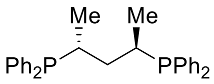

L2

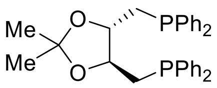

L3

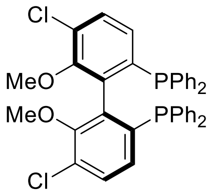

L4

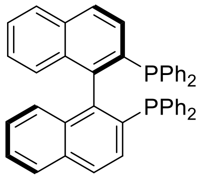

L5

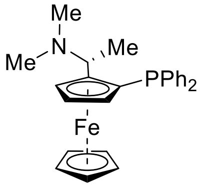

L6

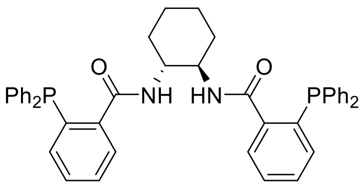

L7

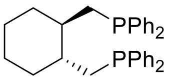

L8

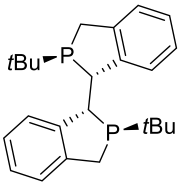

L9

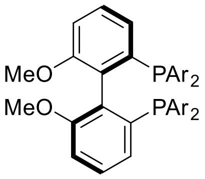

L10 (Ar = 3,5-di-*tert*-butyl-4-methoxyphenyl)

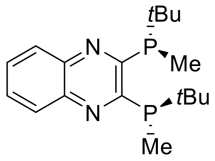

L11

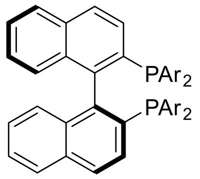

L12 (Ar = 3,5-Xylyl)

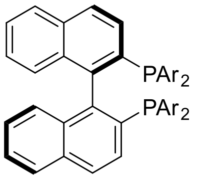

L13 (Ar = 4-Me-Ph)

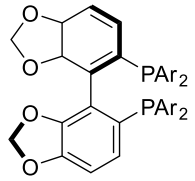

L14 (Ar = Ph), (*R*)-Segphos
L15 (Ar = 3,5-Xylyl), (*R*)-DM-Segphos
L16 (Ar = 3,5-di-*tert*-butyl-4-methoxyphenyl), (*R*)-DTBM-Segphos

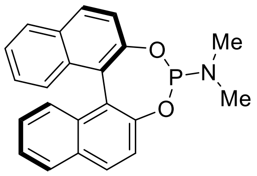

L17

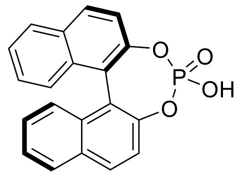

L18

Table S2. Catalyst loading.

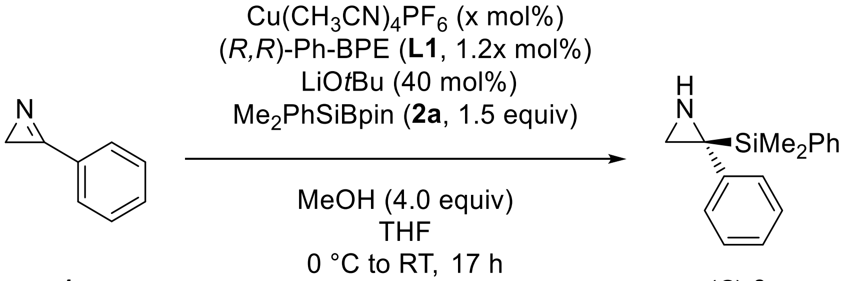

1a (*S*)-**3aa**

**Entry**[a]	**X mol%**	**Yield/%**[b]	***ee*/%**[c]
1	10	95	91
2	5	87	87
3	3	97	90

[a] All reactions were performed on a 0.20 mmol scale. [b] Yield was determined by ^1H NMR analysis with CH_2Br_2 as an internal standard. [c] Determined by HPLC analysis on a chiral stationary phase.

Table S3. Catalyst screening.

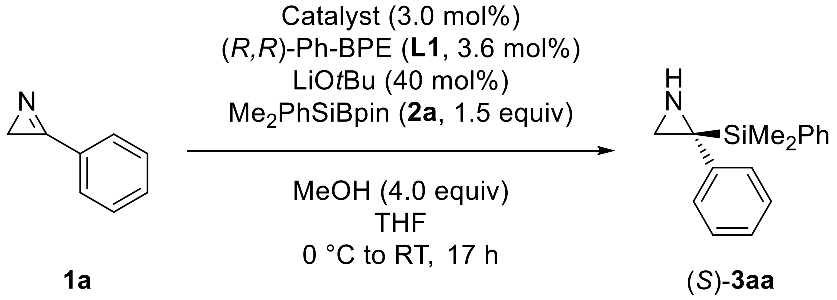

**Entry**[a]	**Catalyst**	**Yield/%**[b]	***ee*/%**[c]
1	$(Ph_3P)_2CuBH_4$	79	88
2	CuCN	31	21
3	CuI	18	57
4	CuTc	38	29
5	$Cu(acac)_2$	45	38
6	$Cu(NO_3)_2$	trace	n.d.
7	$Cu(OTf)_2$	80	49

[a] All reactions were performed on a 0.20 mmol scale. [b] Yield was determined by ^1H NMR analysis with CH_2Br_2 as an internal standard. [c] Determined by HPLC analysis on a chiral stationary phase. n.d. = not detected.

Table S4. Solvent screening.

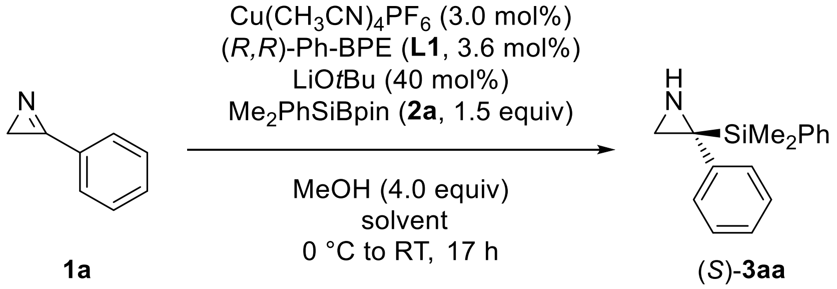

Entry[a]	Solvent	Yield/%[b]	*ee*/%[c]
1	Toluene	92	89
2	CH_3CN	trace	n.d.
3	2-Me-THF	46	31
4	Et_2O	85	87
5[d]	THF	98(85)[e]	95

[a] All reactions were performed on a 0.20 mmol scale. [b] Yield was determined by ^1H NMR analysis with CH_2Br_2 as an internal standard. [c] Determined by HPLC analysis on a chiral stationary phase. [d] MeOH:H_2O (1:1, 2.0 equiv) as proton source. [e] Isolated yield after flash chromatography on silica gel. n.d. = not detected.

Table S5. Additive screening.

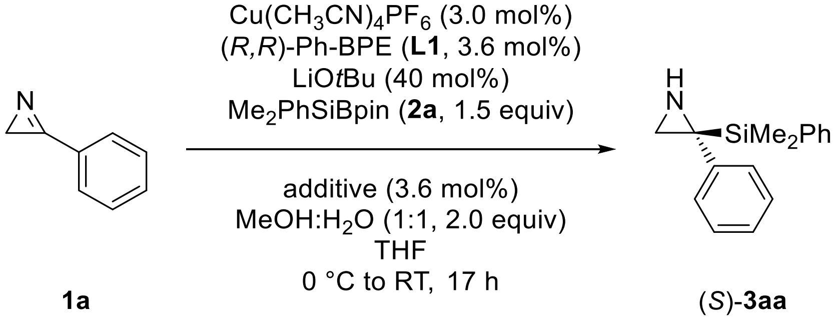

**Entry**[a]	**Additives**	**Yield/%**[b]	***ee*/%**[c]
1	$NaPF_6$	69	82
2	$NaBAr_4$[d]	61	84

[a] All reactions were performed on a 0.20 mmol scale. [b] Yield was determined by ^{1}H NMR analysis with CH_2Br_2 as an internal standard. [c] Determined by HPLC analysis on a chiral stationary phase. [d] Ar = 3,5-Ditrifluoromethylphenyl

Table S6. Base screening.

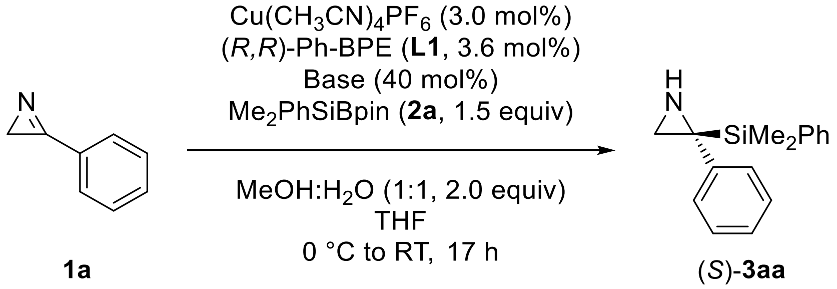

Entry[a]	Base	Yield/%[b]	*ee*/% [c]
1	KO*t*Bu	98	94
2	NaO*t*Bu	98	94
3	LiOMe	96	95

[a] All reactions were performed on a 0.20 mmol scale. [b] Yield was determined by ^{1}H NMR analysis with CH_2Br_2 as an internal standard. [c] Determined by HPLC analysis on a chiral stationary phase.

Table S7. McQuade's N-heterocyclic carbene/copper-catalyzed silylation of 2*H*-aziridines.

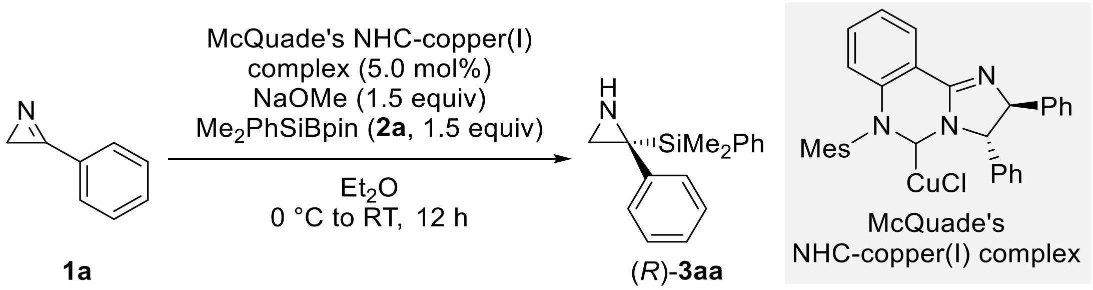

**Entry**[a]	**Base**	**Yield/%**[b]	***ee*/%** [c]
1	NaOMe stored in the glovebox[d]	95	81
2	NaOMe stored outside the glovebox	95	93

[a] All reactions were performed outside the glove box on a 0.10 mmol scale. [b] Yield was determined by ^{1}H NMR analysis with CH_2Br_2 as an internal standard. [c] Determined by HPLC analysis on a chiral stationary phase. [d] NaOMe was quickly weighed out outside the glovebox and then returned for storage.

3 General Procedures

3.1 General Procedure for the Preparation of Silylboronic Esters (GP 1)

Method A

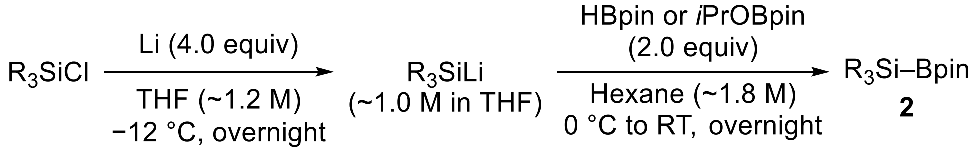

To a flame-dried two-neck round-bottom flask charged with a magnetic stir bar and activated lithium chunks (999 mg, 144 mmol, 4.00 equiv) are added THF (30 mL, ~1.2 M) and R_3SiCl (36.0 mmol, 1.00 equiv) at −12 °C under N_2. After stirring overnight, the freshly prepared R_3SiLi solution (~1 M) is added dropwise to a solution of 4,4,5,5-tetramethyl-1,3,2-dioxaborolane (HBpin, 9.21 g, 72.0 mmol, 2.00 equiv) or isopropoxy-4,4,5,5-tetramethyl-1,3,2-dioxaborolane (*i*PrOBpin, 13.4 g, 72.0 mmol, 2.00 equiv) in hexane (40 mL, ~1.8 M) over 30 minutes at 0 °C under N_2, and then the reaction mixture is stirred overnight at room temperature. The finished reaction mixture is filtered through Celite® and concentrated under reduced pressure. Distillation of the residue affords the silylboronic ester R_3Si–Bpin.[S6a]

Method B

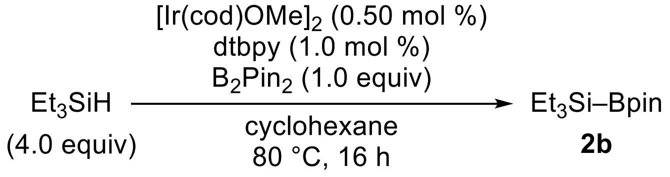

To a flame-dried three-neck round-bottom flask equipped with a condenser and a magnetic stir bar are added $[Ir(cod)OMe]_2$ (66.3 mg, 0.100 mmol, 0.500 mol%), 4,4′-di-*tert*-butyl-2,2′-dipyridyl (dtbpy, 53.7 mg, 0.200 mmol, 1.00 mol%) and bis(pinacolato)diboron (5.08 g, 20.0 mmol, 1.00 equiv). The flask is evacuated and backfilled with N_2 (3 times), and then cyclohexane (40 mL, ~0.5 M) and Et_3SiH (4.65

g, 80.0 mmol, 4.00 equiv) are added successively. The reaction mixture is stirred at 80 °C for 16 hours in an oil bath, and then cooled to room temperature. The solvent is removed under reduced pressure. Purification of the residue by flash chromatography on silica gel using cylohexane/EtOAc = 20/1 as eluent affords the crude gray product, which is further purified by short-path distillation, yielding the Et_3Si–Bpin as a colourless oil.[S6b]

3.2 General Procedure for the Preparation of 2*H*-Azirines (GP2)

Method A

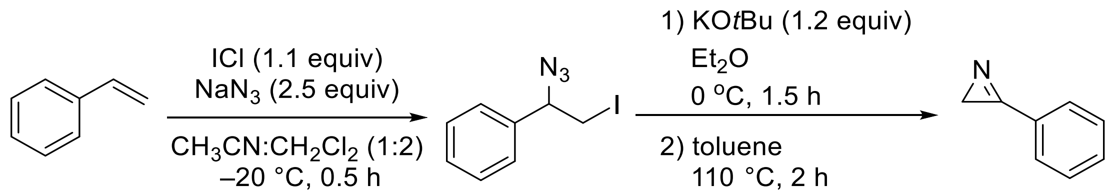

To a suspension of NaN_3 (7.15 g, 110 mmol, 2.50 equiv) in acetonitrile (30 mL) was added dropwise a solution of iodine monochloride (8.07 g, 49.7 mmol, 1.10 equiv) in CH_2Cl_2 (60 mL) at −20 °C, and the mixture was stirred at the same temperature. After 30 min, a solution of styrene (5 mL, 43.6 mmol, 1.00 equiv) in CH_2Cl_2 (20 mL) was added slowly, and the mixture was stirred for 1 h. The reaction was quenched with saturated aqueous $Na_2S_2O_3$, and the organic materials were extracted twice with Et_2O. The combined extracts were washed with brine and dried over $MgSO_4$. After evaporation of solvents, the resulting crude materials were used immediately for the next step without any further purification.

To a solution of the obtained compounds above in Et_2O (100 mL) was added KO*t*Bu (5.92 g, 52.3 mmol, 1.20 equiv) at 0 °C, and the mixture was stirred for 1.5 h at the same temperature. The reaction mixture was filtered through Celite®, and the solvent was removed in vacuo. After evaporation of solvents, the resulting crude materials were used immediately for the next step without any further purification.

A solution of the vinyl azide in toluene (20 mL) was stirred at 110 °C for 2 h. After consumption of all the vinyl azide as monitored by TLC, the solvent was removed under reduced pressure and the crude residue was applied to the silica gel chromatography (*n*-pentane/ethyl acetate) to afford 2*H*-azirines as a colorless oil.[S7]

Method B

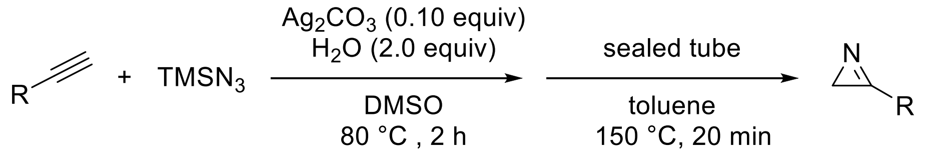

To a solution of alkyne (2.00 mmol, 1.00 equiv), $TMSN_3$ (461 mg, 4.00 mmol, 2.00 equiv) and H_2O (72.1 µL, 4.00 mmol, 2.00 equiv) in DMSO (4 mL) at 80 °C, Ag_2CO_3 (110 mg, 0.400 mmol, 0.100 equiv) were added. The mixture was stirred for 2 h. Upon completion as indicated by TLC, the reaction was quenched with water and extracted with diethyl ether (10 mL × 3). The combined organic layers were washed for three times with water, dried over anhydrous Na_2SO_4. The solvent was removed under reduced pressure to give the crude product which was used in next step without further purification. The crude vinyl azide was heated in toluene (0.1 M) in sealed tube at 150 °C for 20 minutes. The reaction mixture was cooled to room temperature and concentrated under reduced pressure to give the crude product, which was purified by flash silica gel column chromatography (*n*-pentane) to afford 2*H*-azirines.[S8]

3.3 General Procedure for the Synthesis of Racemic Products as HPLC Reference Compounds (GP3)

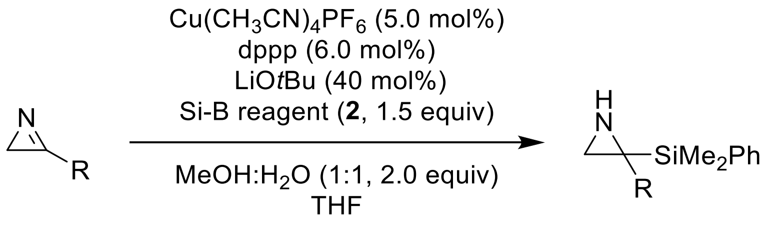

1 *Rac*-**3**

To a flame-dried Schlenk tube equipped with a septum and a magnetic stir bar were added $Cu(CH_3CN)_4PF_6$ (3.73 mg, 5.00 mol%) and 1,3-bis(diphenylphosphino)propane (dppp, 4.95 mg, 6.00 mol%). The tube was evacuated under high vacuum and backfilled with nitrogen gas (3 times). THF (2.0 mL) was added to the tube, and the resulting mixture was stirred under room temperature for 30 minutes. The mixture was then cooled to 0 °C, and LiO*t*Bu (2.2 M, 36.4 μL, 0.080 mmol, 40.0 mol%) and Si–B reagent (**2**, 0.300 mmol, 1.50 equiv) were successively added dropwise. The mixture was stirred under 0 °C for 5 minutes. And then the mixture of methanol/water (1:1, 28.0 μL, 2.00 equiv) was added, followed by dropwise addition of 2*H*-azirine **1** (0.200 mmol, 1.00 equiv). The ice bath was subsequently removed, and the reaction was stirred for 17 h at room temperature. After the indicated reaction time, the reaction mixture was filtered through a short plug of silica, and the filter cake was washed with EtOAc (15 mL). The filtrate was concentrated under vacuum. Purification of the residue by flash column chromatography on silica gel with *n*-hexane and ethyl acetate as the eluent to afford the silylated product *rac*-**3**.

3.4 Typical Procedures for the Synthesis of Enantioenriched Products (GP4)

Method A:

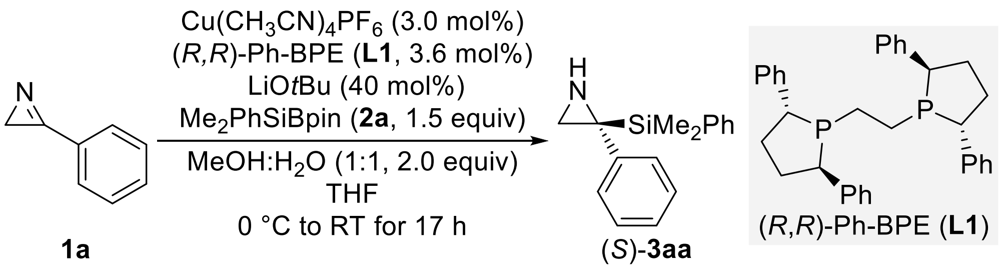

To a flame-dried Schlenk tube equipped with a septum and a magnetic stir bar were added $Cu(CH_3CN)_4PF_6$ (2.24 mg, 3.00 mol%) and (*R*,*R*)-Ph-BPE (3.65 mg, 3.60 mol%). The tube was evacuated under high vacuum and backfilled with nitrogen gas (3 times). THF (2.0 mL) was added to the tube, and the resulting mixture was stirred under room temperature for 30 minutes. The mixture was then cooled to 0 °C, and LiO*t*Bu (2.2 M, 36.4 μL, 0.0800 mmol, 40.0 mol%) and $Me_2PhSiBpin$ (**2a**, 0.300 mmol, 1.50 equiv) were successively added dropwise. The mixture was stirred under 0 °C for 5 minutes. And then the mixture of methanol/water (1:1, 28.0 μL, 2.00 equiv) was added, followed by dropwise addition of 2*H*-azirine **1a** (0.200 mmol, 1.00 equiv). The ice bath was subsequently removed, and the reaction was stirred for 17 h at room temperature. After the indicated reaction time, the reaction mixture was filtered through a short plug of silica, and the filter cake was washed with EtOAc (15 mL). The filtrate was concentrated under vacuum. Purification of the residue by flash column chromatography on silica gel with *n*-hexane and ethyl acetate as the eluent to afford the silylated product (*S*)-**3aa**.

Method B:

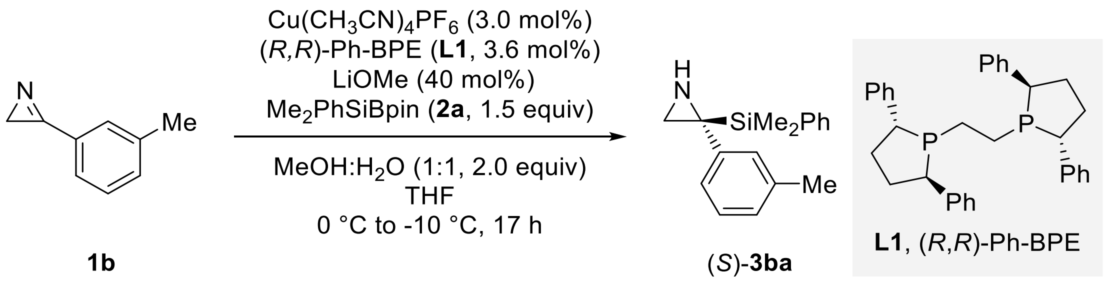

To a flame-dried Schlenk tube equipped with a septum and a magnetic stir bar were added $Cu(CH_3CN)_4PF_6$ (2.24 mg, 3.00 mol%) and (*R*,*R*)-Ph-BPE (3.65 mg, 3.60 mol%) and LiOMe (3.04 mg, 40.0 mol%). The tube was evacuated under high vacuum and backfilled with nitrogen gas (3 times). THF (2.0 mL) was added to the tube, and the resulting mixture was stirred under room temperature for 30 minutes. The mixture was then cooled to 0 °C, $Me_2PhSiBpin$ (**2a**, 0.300 mmol, 1.50 equiv) were added dropwise. The mixture was stirred under 0 °C for 5 minutes. And then the mixture of methanol/water (1:1, 28.0 μL, 2.00 equiv) was added, followed by dropwise addition of 2*H*-azirine **1b** (0.200 mmol, 1.00 equiv). The ice bath was subsequently removed, and the reaction was stirred for 17 h at −10 °C. After the indicated reaction time, the reaction mixture was filtered through a short plug of silica, and the filter cake was washed with EtOAc (15 mL). The filtrate was concentrated under vacuum. Purification of the residue by flash column chromatography on silica gel with *n*-hexane and ethyl acetate as the eluent to afford the silylated product (*S*)-**3ba**.

4 Experimental Details and Characterization Data for Starting Materials

4.1 Experimental Details and Characterization Data for Silylboronic Esters

Me_2PhSi–Bpin, $MePh_2Si$–Bpin, and Ph_3Si–Bpin were prepared according to **GP 1** (Method A). Data are in agreement with those reported.[S6a]

Et_3Si–Bpin was prepared according to **GP1** (Method B). Data are in agreement with those reported.[S6b]

4.2 Experimental Details and Characterization Data for 2*H*-Azirines

1a,[S8] **1b**,[S8] **1c**,[S8] **1d**,[S9b] **1e**,[S8] **1h**,[S8] **1i**,[S8] **1j**,[S8] **1k**,[S9c] **1o**,[S8] **1p**,[S9a] **1q**,[S8] **1r**,[S9b] **1s**[S9a] **1t**,[S8] **1u**,[S8], **1v**,[S8] **1w**,[S8] **1x**,[S8] **1y**,[S9a] **1z**,[S8] **1c'**,[S8]were synthesized according to known procedures and all spectroscopic data matched those reported.

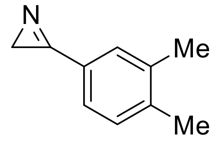

1f
$C_{10}H_{11}N$
M = 145.21 g/mol

3-(3,4-dimethylphenyl)-2*H*-azirine (**1f**): Prepared from 1,2-dimethyl-4-vinylbenzene (651 mg, 5.00 mmol, 1.00 equiv) according to the **GP2** (*Method A*). Purification by flash column chromatography on silica gel using *n*-hexane:EtOAc = 10:1 as eluent afforded **1f** as colorless oil (290 mg, 40% yield). $\mathbf{R_f}$ = 0.50 (*n*-hexane:EtOAc = 10:1). ^{1}**H NMR** (500 MHz, $CDCl_3$, 298 K) δ 7.68 (s, 1H), 7.63 (d, *J* = 7.7 Hz, 1H), 7.31 (d, *J* = 7.8 Hz, 1H), 2.35 (s, 3H), 2.35 (s, 3H), 1.74 (s, 2H) ppm. ^{13}**C NMR** (126 MHz, $CDCl_3$, 298 K) δ 165.3, 142.4, 137.5, 130.5, 130.3, 127.2, 123.1, 20.1, 19.6, 19.4 ppm. **HRMS** (APCI) m/z: $[M+H]^+$ calcd for $C_{10}H_{12}N^+$ 146.0966; found 146.0964. **IR** (ATR): ṽ 708, 751, 820, 887, 984, 1002, 1275, 1315, 1384, 1405, 1450, 1497, 1606, 1737, 2919, 2973, 3038 cm^{-1}.

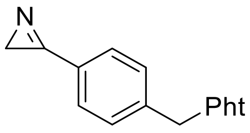

1g
$C_{17}H_{12}N_2O_2$
M = 276.30 g/mol

2-(4-(2*H*-azirin-3-yl)benzyl)isoindoline-1,3-dione (**1g**): Prepared from 2-(4-vinylbenzyl)isoindoline-1,3-dione (1.32 g, 5.00 mmol, 1.00 equiv) according to the **GP2** (*Method A*). Purification by flash column chromatography on silica gel using *n*-hexane:EtOAc = 10:1 as eluent afforded **1g** as pale yellow solid (967 mg, 70% yield). $\mathbf{R_f}$ = 0.10 (*n*-hexane:EtOAc = 10:1). **M.P.** 105–107 °C. ^{1}**H NMR** (500 MHz, $CDCl_3$, 298 K) δ 7.90–7.80 (m, 4H), 7.77–7.69 (m, 2H), 7.61 (d, *J* = 8.0 Hz, 2H), 4.93 (s, 2H), 1.76 (s, 2H) ppm. ^{13}**C NMR** (126 MHz, $CDCl_3$, 298 K) δ 167.9, 165.4, 141.3, 134.2, 132.0, 129.9, 129.1, 125.0, 123.5, 41.4, 19.7 ppm. **HRMS** (APCI) m/z: $[M+H]^+$ calcd for $C_{17}H_{13}N_2O_2^+$ 277.0974; found 277.0972. **IR** (ATR): ṽ 715, 764, 935, 1082, 1326, 1391, 1423, 1606, 1697, 2928, 3040 cm^{-1}.

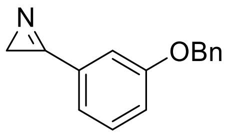

1l
$C_{15}H_{13}NO$
M = 223.28 g/mol

3-(3-(benzyloxy)phenyl)-2*H*-azirine (**1l**): Prepared from methyl 1-(benzyloxy)-3-vinylbenzene (1.04 g, 5.00 mmol, 1.00 equiv) according to the **GP2** (*Method A*). Purification by flash column chromatography on silica gel using *n*-hexane:EtOAc = 10:1 as eluent afforded **1l** as pale yellow solid (781 mg, 70% yield). $\mathbf{R_f}$ = 0.30 (*n*-hexane:EtOAc = 10:1). **M.P.** 58–60 °C. ^{1}**H NMR** (500 MHz, $CDCl_3$, 298 K) δ 7.88–7.83 (m, 2H), 7.50–7.39 (m, 4H), 7.38–7.33 (m, 1H), 7.17–7.10 (m, 2H), 5.16 (s, 2H), 1.74 (s, 2H) ppm. ^{13}**C NMR** (126 MHz, $CDCl_3$, 298 K) δ 164.3, 162.4, 136.1, 131.5, 128.7, 128.2, 127.4, 118.3, 115.4, 70.2, 19.3 ppm. **HRMS** (APCI) m/z: $[M+H]^+$ calcd for $C_{15}H_{14}NO^+$ 224.1074; found 224.1070. **IR** (ATR): ṽ 659, 694, 733, 834, 993, 1021, 1107, 1166, 1251, 1308, 1380, 1421, 1452, 1500, 1573, 1601, 1733, 2857, 3037 cm^{-1}.

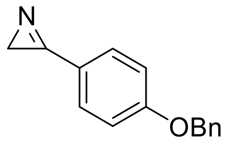

1m
$C_{15}H_{13}NO$
M = 223.28 g/mol

3-(4-(benzyloxy)phenyl)-2*H*-azirine (**1m**): Prepared from methyl 1-(benzyloxy)-4-vinylbenzene (1.04 g, 5.00 mmol, 1.00 equiv) according to the **GP2** (*Method A*). Purification by flash column chromatography on silica gel using *n*-hexane:EtOAc = 10:1 as eluent afforded **1m** as pale yellow oil (726 mg, 65% yield). **R*f*** = 0.30 (*n*-hexane:EtOAc = 10:1). **^{1}H NMR** (500 MHz, $CDCl_3$, 298 K) δ 7.55–7.50 (m, 2H), 7.49–7.44 (m, 3H), 7.43–7.38 (m, 2H), 7.38–7.32 (m, 1H), 7.21 (ddd, *J* = 8.0, 2.6, 1.3 Hz, 1H), 5.14 (s, 2H), 1.80 (s, 2H) ppm. **^{13}C NMR** (126 MHz, $CDCl_3$, 298 K) δ 165.8, 159.1, 136.3, 130.1, 128.6, 128.1, 127.4, 126.7, 122.5, 120.2, 114.5, 70.2, 19.9 ppm. **HRMS** (APCI) m/z: $[M+H]^+$ calcd for $C_{15}H_{14}NO^+$ 224.1074; found 224.1070. **IR** (ATR): ṽ 683, 738, 794, 857, 927, 988, 1009, 1080, 1115, 1159, 1227, 1275, 1292, 1323, 1384, 1434, 1454, 1488, 1586, 1736, 2933, 2977, 3047 cm^{-1}.

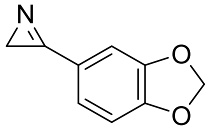

1n
$C_9H_7NO_2$
M = 161.16 g/mol

3-(benzo[d][1,3]dioxol-5-yl)-2*H*-azirine (**1n**): Prepared from 5-vinylbenzo[*d*][1,3]dioxole (731 mg, 5.00 mmol, 1.00 equiv) according to the **GP2** (*Method A*). Purification by flash column chromatography on silica gel using *n*-hexane:EtOAc = 10:1 as eluent afforded **1n** as pale yellow solid (524 mg, 65% yield). **R*f*** = 0.30 (*n*-hexane:EtOAc = 10:1). **M.P.** 73–75 °C. **^{1}H NMR** (500 MHz, $CDCl_3$, 298 K) δ 7.40 (dd, *J* = 8.0, 1.6 Hz, 1H), 7.35 (d, *J* = 1.5 Hz, 1H), 6.96 (d, *J* = 8.0 Hz, 1H), 6.07 (s, 2H), 1.74 (s, 2H) ppm. **^{13}C NMR** (126 MHz, $CDCl_3$, 298 K) δ 164.7, 151.6, 148.4, 126.0, 119.7, 108.8, 108.4, 101.9, 19.8 ppm. **HRMS** (APCI) m/z: $[M+H]^+$ calcd for

$C_9H_8NO_2^+$ 162.0551; found 162.0550. **IR** (ATR): ṽ 663, 766, 826, 856, 906, 927, 995, 1031, 1110, 1227, 1257, 1290, 1435, 1488, 1733, 2197, 2964 cm^{-1}.

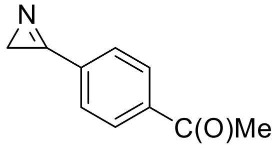

1t
$C_{10}H_9NO$
M = 159.19 g/mol

1-(4-(2*H*-azirin-3-yl)phenyl)ethan-1-one (**1t**): Prepared from 1-(4-vinylphenyl)ethan-1-one (731 mg, 5.00 mmol, 1.00 equiv) according to the **GP2** (*Method A*). Purification by flash column chromatography on silica gel using *n*-hexane:EtOAc = 5:1 as eluent afforded **1t** as white solid (557 mg, 70% yield). **R*f*** = 0.20 (*n*-hexane:EtOAc = 5:1). **M**.**P**. 45–47 °C. **^{1}H NMR** (500 MHz, $CDCl_3$) δ 8.17–8.09 (m, 2H), 8.06–7.95 (m, 2H), 2.67 (s, 3H), 1.85 (s, 2H) ppm. **^{13}C NMR** (126 MHz, $CDCl_3$) δ 197.1, 165.8, 140.2, 129.7, 129.3, 128.8, 26.8, 20.2 ppm. **HRMS** (APCI) m/z: [M+H]$^+$ calcd for $C_{10}H_{10}NO^+$ 160.0757; found 160.0757. **IR** (ATR): ṽ 712, 833, 958, 993, 1253, 1359, 1339, 1602, 1675 cm^{-1}.

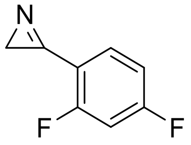

1a'
$C_8H_5F_2N$
M = 153.13 g/mol

3-(2,4-difluorophenyl)-2*H*-azirine (**1a'**): Prepared from 2,4-difluoro-1-vinylbenzene (691 mg, 5.00 mmol, 1.00 equiv) according to the **GP2** (*Method A*). Purification by flash column chromatography on silica gel using *n*-hexane:EtOAc = 10:1 as eluent afforded **1a'** as colorless oil (475 mg, 62% yield). **R*f*** = 0.50 (*n*-hexane:EtOAc = 10:1). **^{1}H NMR** (500 MHz, $CDCl_3$, 298 K) δ 7.92–7.84 (m, 1H), 7.25–7.17 (m, 2H), 1.82–1.66 (m, 2H) ppm. **^{13}C NMR** (126 MHz, $CDCl_3$, 298 K) δ 166.0 (dd, *J* = 257.6, 11.8 Hz), 162.3 (dd, *J* = 262.8, 11.8 Hz), 161.2 (s), 132.6 (dd, *J* = 10.7, 3.3 Hz), 112.5 (dd, *J* = 22.2, 3.6 Hz), 111.0 (d, *J* = 15.4 Hz), 105.2 (dd, *J* = 25.7, 23.5 Hz), 18.5 (s) ppm. **^{19}F NMR** (471 MHz, $CDCl_3$, 298 K) δ −99.2–−101.8 (m), −106.9–−107.2 (m) ppm. **HRMS** (APCI) m/z:

$[M+H]^+$ calcd for $C_8H_6F_2N^+$ 154.0466; found 154.0463. **IR** (ATR): ṽ 732, 768, 818, 852, 963, 995, 1089, 1132, 1145, 1272, 1315, 1431, 1500, 1607, 1741, 2984, 3050 cm^{-1}.

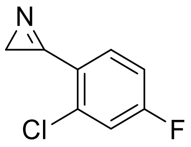

1b'
C_8H_5ClFN
M = 169.58 g/mol

3-(2-chloro-4-fluorophenyl)-2*H*-azirine (**1b'**): Prepared from 2-chloro-4-fluoro-1-vinylbenzene (773 mg, 5.00 mmol, 1.00 equiv) according to the **GP2** (*Method A*). Purification by flash column chromatography on silica gel using *n*-hexane:EtOAc = 10:1 as eluent afforded **1b'** as pale yellow oil (577 mg, 68% yield). **R_f** = 0.50 (*n*-hexane:EtOAc = 10:1). **^{1}H NMR** (500 MHz, $CDCl_3$, 298 K) δ 7.86 (dd, *J* = 8.6, 6.0 Hz, 1H), 7.29 (dd, *J* = 8.4, 2.5 Hz, 1H), 7.18 (ddd, *J* = 8.6, 7.8, 2.5 Hz, 1H), 1.83 (s, 2H) ppm. **^{13}C NMR** (126 MHz, $CDCl_3$, 298 K) δ 165.8, 163.8 (d, *J* = 12.7 Hz), 137.6 (d, *J* = 11.1 Hz), 133.9 (d, *J* = 10.1 Hz), 120.6 (d, *J* = 3.4 Hz), 118.4 (d, *J* = 25.1 Hz), 114.9 (d, *J* = 21.9 Hz), 20.1 ppm. **^{19}F NMR** (471 MHz, $CDCl_3$, 298 K) δ −103.43 (td, *J* = 8.0, 6.0 Hz) ppm. **HRMS** (APCI) m/z: $[M+H]^+$ calcd for $C_8H_6ClFN^+$ 170.0167; found 170.0167. **IR** (ATR): ṽ 686, 759, 821, 860, 906, 988, 1044, 1210, 1262, 1311, 1397, 1459, 1486, 1574, 1593, 1730, 2981, 3051 cm^{-1}.

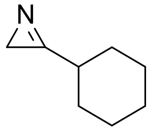

1d'
$C_8H_{13}N$
M = 123.20 g/mol

3-cyclohexyl-2*H*-azirine (**1d'**): Prepared from ethynylcyclohexane (541 mg, 5.00 mmol, 1.00 equiv) according to the **GP2** (*Method B*). Purification by flash column chromatography on silica gel using *n*-hexane:EtOAc = 10:1 as eluent afforded **1d'** as colorless oil (154 mg, 25% yield). **R_f** = 0.50 (*n*-hexane:EtOAc = 10:1). **^{1}H NMR** (500 MHz, $CDCl_3$, 298 K) δ 2.85–2.72 (m, 1H), 2.00–1.91 (m, 1H), 1.75–1.68 (m, 1H), 1.67–

1.59 (m, 1H), 1.59–1.48 (m, 1H), 1.48–1.36 (m, 1H), 1.34 (d, *J* = 0.6 Hz, 1H) ppm. **^{13}C NMR** (126 MHz, $CDCl_3$, 298 K) δ 172.0, 36.5, 27.7, 25.8, 24.8, 18.2 ppm. **HRMS** (APCI) m/z: $[M+H]^+$ calcd for $C_8H_{14}N^+$ 124.1122; found 124.1121. **IR** (ATR): ṽ 730, 796, 913, 984, 1013, 1090, 1258, 1448, 1759, 2855, 2929 cm^{-1}.

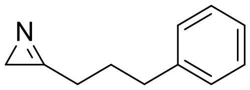

1f'
$C_{11}H_{13}N$
M = 159.23 g/mol

3-(3-phenylpropyl)-2*H*-azirine (**1f'**): Prepared from pent-4-yn-1-ylbenzene (721 mg, 5.00 mmol, 1.00 equiv) according to the **GP2** (*Method B*). Purification by flash column chromatography on silica gel using *n*-hexane:EtOAc = 10:1 as eluent afforded **1f'** as colorless oil (239 mg, 30% yield). **R_f** = 0.50 (*n*-hexane:EtOAc = 10:1). **^{1}H NMR** (500 MHz, $CDCl_3$, 298 K) δ 7.33–7.27 (m, 2H), 7.24–7.17 (m, 3H), 2.80 (t, *J* = 7.3 Hz, 2H), 2.75 (t, *J* = 7.4 Hz), 2.13–2.06 (m, 2H), 1.37 (s, 2H) ppm. **^{13}C NMR** (126 MHz, $CDCl_3$, 298 K) δ 169.5, 140.9, 128.4, 126.1, 77.0, 35.0, 27.7, 25.7, 18.7 ppm. **HRMS** (APCI) m/z: $[M+H]^+$ calcd for $C_{11}H_{14}N^+$ 160.1124; found 160.1121. **IR** (ATR): ṽ 698, 744, 988, 1452, 1494, 1062, 1763, 2096, 2860, 2937, 3026 cm^{-1}.

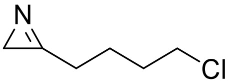

1g'
$C_6H_{10}ClN$
M = 131.60 g/mol

3-(4-chlorobutyl)-2*H*-azirine (**1g'**): Prepared from 6-chlorohex-1-yne (583 mg, 5.00 mmol, 1.00 equiv) according to the **GP2** (*Method B*). Purification by flash column chromatography on silica gel using *n*-hexane:EtOAc = 10:1 as eluent afforded **1g'** as red oil (329 mg, 50% yield). **R_f** = 0.50 (*n*-hexane:EtOAc = 5:1). **^{1}H NMR** (500 MHz, $CDCl_3$) δ 3.58 (t, *J* = 6.1 Hz, 2H), 2.84 (t, *J* = 6.8 Hz, 2H), 1.99–1.82 (m, 4H), 1.39 (s, 2H) ppm. **^{13}C NMR** (126 MHz, $CDCl_3$) δ 169.4, 44.3, 31.8, 27.7, 21.5, 18.9 ppm. **HRMS** (APCI) m/z: $[M+H]^+$ calcd for $C_6H_{11}ClN^+$ 132.0575; found 132.0574. **IR** (ATR): ṽ 727, 763, 798, 988, 1257, 1280, 1305, 1419, 1450, 1764 cm^{-1}.

5 Experimental Details and Characterization Data for Chiral Silylaziridines

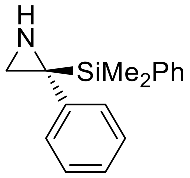

(*S*)-**3aa**
$C_{16}H_{19}NSi$
M = 253.42 g/mol

(*S*)-2-(dimethyl(phenyl)silyl)-2-phenylaziridine [(*S*)-**3aa**]: Prepared from 3-phenyl-2*H*-azirine (**1a**, 23.4 mg, 0.200 mmol, 1.00 equiv) according to **GP4** (*Method A*). The residue was purified by flash column chromatography on silica gel using *n*-hexane:ethyl acetate = 10:1 as the eluent to afford (*S*)-**3aa** as colorless oil (48.1 mg, 85% yield). **R*f*** = 0.10 (*n*-hexane:ethyl acetate = 10:1). **^{1}H NMR** (500 MHz, $CDCl_3$, 298 K) δ 7.53–7.48 (m, 2H), 7.44–7.39 (m, 1H), 7.39–7.34 (m, 2H), 7.25–7.20 (m, 4H), 7.20–7.13 (m, 1H), 1.89 (s, 1H), 1.83 (s, 1H), 0.71 (s, 1H), 0.38 (s, 3H), 0.31 (s, 3H) ppm. **^{13}C NMR** (126 MHz, $CDCl_3$, 298 K) δ 143.3, 134.9, 134.2, 129.7, 128.7, 127.9, 127.9, 126.0, 32.1, 29.7, −4.6, −5.1 ppm. **^{1}H/^{29}Si HMQC NMR** (500/99 MHz, $CDCl_3$, 298 K, optimized for *J* = 7 Hz): δ 7.53–7.48/−0.8, 0.38/−0.8, 0.31/−0.8 ppm. **HRMS** (APCI) m/z: $[M+H]^+$ calcd for $C_{16}H_{20}NSi^+$ 254.1360; found 254.1361. **IR** (ATR): ṽ 661, 694, 732, 774, 821, 873, 1011, 1026, 1070, 1095, 1114, 1213, 1246, 1427, 1486, 1594, 2959, 3046 cm^{-1}. Optical rotation: $[\alpha]_D^{20}$ = −11.1 (*c* 1.0, CH_2Cl_2, 95% *ee*). The enantiomeric excess of (*S*)-**3aa** was determined by HPLC analysis on a chiral stationary phase (Daicel Chiralcel IC column, column temperature 20 °C, solvent heptane:*i*PrOH = 99:1, flow rate 0.6 mL/min): t_R = 33.1 min (major), t_R = 37.9 min (minor).

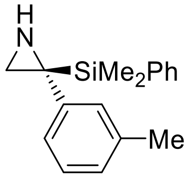

(*S*)-**3ba**
$C_{17}H_{21}NSi$
M = 267.45 g/mol

(*S*)-2-(dimethyl(phenyl)silyl)-2-(*m*-tolyl)aziridine [(*S*)-**3ba**]: Prepared from 3-(*m*-tolyl)-2*H*-azirine (**1b**, 26.2 mg, 0.200 mmol, 1.00 equiv) according to **GP4** (*Method B*). The residue was purified by flash column chromatography on silica gel using *n*-hexane:ethyl acetate = 10:1 as the eluent to afford (*S*)-**3ba** as colorless oil (43.9 mg, 82% yield). **R_f** = 0.10 (*n*-hexane:ethyl acetate = 10:1). **^{1}H NMR** (500 MHz, $CDCl_3$, 298 K) δ 7.58–7.54 (m, 2H), 7.45–7.34 (m, 3H), 7.14 (t, *J* = 7.5 Hz, 1H), 7.09–7.01 (m, 2H), 6.99 (d, *J* = 7.4 Hz, 1H), 2.30 (s, 3H), 1.87 (s, 1H), 1.81 (s, 1H), 0.58 (s, 1H), 0.39 (s, 3H), 0.32 (s, 3H). **^{13}C NMR** (126 MHz, $CDCl_3$, 298 K) δ 143.0, 137.4, 135.0, 134.2, 129.6, 129.4, 127.8, 127.7, 126.8, 125.7, 31.9, 29.6, 21.3, −4.6, −5.1 ppm. **^{1}H/^{29}Si HMQC NMR** (500/99 MHz, $CDCl_3$, 298 K, optimized for *J* = 7 Hz): δ 7.45–7.34/−0.8, 0.39/−0.8, 0.32/−0.8 ppm. **HRMS** (APCI) m/z: $[M+H]^+$ calcd for $C_{17}H_{22}NSi^+$ 268.1516; found 268.1516. **IR** (ATR): ṽ 662, 699, 734, 775, 814, 908, 1109, 1247, 1426, 1484, 1601, 2955 cm^{-1}. Optical rotation: $[\alpha]_D^{20}$ = −15.1 (*c* 1.0, CH_2Cl_2, 93% *ee*). The enantiomeric excess of (*S*)-**3ba** was determined by HPLC analysis on a chiral stationary phase (Daicel Chiralcel IA column, column temperature 20 °C, solvent heptane:*i*PrOH = 99:1, flow rate 0.7 mL/min): t_R = 29.6 min (major), t_R = 18.7 min (minor).

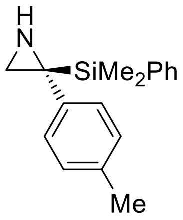

(*S*)-**3ca**
$C_{17}H_{21}NSi$
M = 267.45 g/mol

(*S*)-2-(dimethyl(phenyl)silyl)-2-(p-tolyl)aziridine [(*S*)-**3ca**]: Prepared from 3-(*p*-tolyl)-2*H*-azirine (**1c**, 26.2 mg, 0.200 mmol, 1.00 equiv) according to **GP4** (*Method A*). The residue was purified by flash column chromatography on silica gel using *n*-hexane:ethyl acetate = 10:1 as the eluent to afford (*S*)-**3ca** as colorless oil (41.2 mg, 77% yield). **R_f** = 0.10 (*n*-hexane:ethyl acetate = 10:1). **^{1}H NMR** (500 MHz, $CDCl_3$, 298 K) δ 7.53–7.48 (m, 2H), 7.44–7.39 (m, 1H), 7.39–7.35 (m, 2H), 7.12 (d, J = 8.0 Hz, 2H), 7.05 (d, J = 7.8 Hz, 2H), 2.31 (s, 3H), 1.85 (s, 1H), 1.79 (s, 1H), 0.90 (s, 1H), 0.37 (s, 3H), 0.31 (s, 3H) ppm. **^{13}C NMR** (126 MHz, $CDCl_3$, 298 K) δ 140.1, 135.5, 135.1, 134.2, 129.6, 128.6, 128.5, 127.8, 31.8, 29.8, 21.0, −4.6, −5.1 ppm. **HMQC NMR** (500/99 MHz, $CDCl_3$, 298 K, optimized for *J* = 7 Hz): δ 7.53–7.48/−0.9, 0.37/−0.9, 0.31/−0.9 ppm. **HRMS** (APCI) m/z: $[M+H]^+$ calcd for $C_{17}H_{22}NSi^+$ 268.1516; found 268.1516. **IR** (ATR): ṽ 699, 734, 774, 813, 829, 875, 1109, 1247, 1426, 1509, 2955 cm^{-1}. Optical rotation: $[\alpha]_D^{20}$ = −10.9 (*c* 1.0, CH_2Cl_2, 90% *ee*). The enantiomeric excess of (*S*)-**3ca** was determined by HPLC analysis on a chiral stationary phase (Daicel Chiralcel IC column, column temperature 25 °C, solvent heptane:*i*PrOH = 99:1, flow rate 1 mL/min): t_R = 21.4 min (major), t_R = 27.3 min (minor).

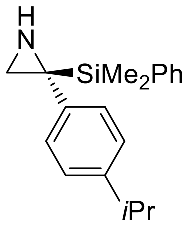

(S)-**3da**
$C_{19}H_{25}NSi$
M = 295.50 /mol

(*S*)-2-(dimethyl(phenyl)silyl)-2-(4-isopropylphenyl)aziridine [(*S*)-**3da**]: Prepared from 3-(4-isopropylphenyl)-2*H*-azirine (**1d**, 31.8 mg, 0.200 mmol, 1.00 equiv) according to **GP4** (*Method A*). The residue was purified by flash column chromatography on silica gel using *n*-hexane:ethyl acetate = 10:1 as the eluent to afford (*S*)-**3da** as colorless oil (35.5 mg, 60% yield). **R_f** = 0.10 (*n*-hexane:ethyl acetate = 10:1). **^{1}H NMR** (500 MHz, $CDCl_3$, 298 K) δ 7.53–7.48 (m, 2H), 7.43–7.33 (m, 3H), 7.14 (d, *J* = 7.9 Hz, 2H), 7.09 (d, *J* = 7.8 Hz, 2H), 2.86 (sept, *J* = 6.9 Hz, 1H), 1.84 (s, 2H), 0.98 (s, 1H), 1.22(s, 3H), 1.24(s, 3H), 0.38 (s, 3H), 0.31 (s, 3H) ppm. **^{13}C NMR** (126 MHz, $CDCl_3$, 298 K) δ 146.6, 140.4, 135.2, 134.3, 129.6, 128.5, 127.8, 126.0, 33.7, 31.7, 29.7, 24.0, 23.9, −4.5, −5.0 ppm. **^{1}H/^{29}Si HMQC NMR** (500/99 MHz, $CDCl_3$, 298 K, optimized for *J* = 7 Hz): δ 7.53–7.48/−0.9, 0.38/−0.9, 0.31/−0.9 ppm. **HRMS** (APCI) m/z: $[M+H]^+$ calcd for $C_{19}H_{26}NSi^+$ 296.1829; found 296.1828. **IR** (ATR): ṽ 699, 734, 773, 816, 876, 1109, 1247, 1409, 1426, 1458, 1508, 2956 cm^{-1}. Optical rotation: $[\alpha]_D^{20}$ = −10.3 (*c* 1.0, CH_2Cl_2, 90% *ee*). The enantiomeric excess of (*S*)-**3da** was determined by HPLC analysis on a chiral stationary phase (Daicel Chiralcel IA column, column temperature 20 °C, solvent heptane:*i*PrOH = 99:1, flow rate 0.7 mL/min): t_R = 33.8 min (major), t_R = 24.1 min (minor).

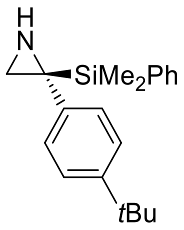

(*S*)-**3ea**
$C_{20}H_{27}NSi$
M = 309.53 g/mol

(*S*)-2-(4-(*tert*-butyl)phenyl)-2-(dimethyl(phenyl)silyl)aziridine [(*S*)-**3ea**]: Prepared from 3-(4-(*tert*-butyl)phenyl)-2*H*-azirine (**1e**, 34.7 mg, 0.200 mmol, 1.00 equiv) according to **GP4** (*Method A*). The residue was purified by flash column chromatography on silica gel using *n*-hexane:ethyl acetate = 10:1 as the eluent to afford (*S*)-**3ea** as colorless oil (52.6 mg, 85% yield). $\mathbf{R_f}$ = 0.10 (*n*-hexane:ethyl acetate = 10:1). **^{1}H NMR** (500 MHz, $CDCl_3$, 298 K) δ 7.56–7.49 (m, 2H), 7.44–7.34 (m, 3H), 7.29–7.24 (m, 2H), 7.17 (d, *J* = 8.1 Hz, 2H), 1.84 (s, 1H), 1.80 (s, 1H), 1.31 (s, 9H), 0.39 (s, 3H), 0.33 (s, 3H) ppm. **^{13}C NMR** (126 MHz, $CDCl_3$, 298 K) δ 148.8, 140.0, 135.2, 134.2, 129.6, 128.2, 127.8, 124.8, 34.3, 31.6, 31.3, 29.7, −4.5, −5.0 ppm. **^{1}H/^{29}Si HMQC NMR** (500/99 MHz, $CDCl_3$, 298 K, optimized for *J* = 7 Hz): δ 7.56–7.49/−0.7, 1.84/−0.7, 1.80/−0.7, 1.31/−0.7, 0.39/−0.7, 0.33/−0.7 ppm. **HRMS** (APCI) m/z: $[M+H]^+$ calcd for $C_{20}H_{28}NSi^+$ 310.1985; found 310.1985. **IR** (ATR): ṽ 701, 739, 778, 818, 877, 1010, 1109, 1140, 1213, 1248, 1360, 1425, 1459, 1505, 2953 cm^{-1}. Optical rotation: $[\alpha]_D^{20}$ = −5.28 (*c* 1.0, CH_2Cl_2, 91% *ee*). The enantiomeric excess of (*S*)-**3ea** was determined by HPLC analysis on a chiral stationary phase (Daicel Chiralcel IC column, column temperature 20 °C, solvent heptane:*i*PrOH = 99:1, flow rate 0.7 mL/min): t_R = 33.2 min (major), t_R = 28.4 min (minor).

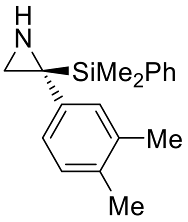

(*S*)-**3fa**
$C_{18}H_{23}NSi$
M = 281.47 g/mol

(*S*)-2-(dimethyl(phenyl)silyl)-2-(3,4-dimethylphenyl)aziridine [(*S*)-**3fa**]: Prepared from 3-(3,4-dimethylphenyl)-2*H*-azirine (**1f**, 29.0 mg, 0.200 mmol, 1.00 equiv) according to **GP4** (*Method B*). The residue was purified by flash column chromatography on silica gel using *n*-hexane:ethyl acetate = 10:1 as the eluent to afford (*S*)-**3fa** as colorless oil (47.3 mg, 84% yield). $\mathbf{R_f}$ = 0.10 (*n*-hexane:ethyl acetate = 10:1). **^{1}H NMR** (500 MHz, $CDCl_3$, 298 K) δ 7.55–7.49 (m, 2H), 7.44–7.35 (m, 3H), 7.00 (d, *J* = 7.3 Hz, 2H), 6.95 (d, *J* = 7.7 Hz, 1H), 2.22 (s, 3H), 2.21 (s, 3H), 1.83 (s, 1H), 1.78 (s, 1H), 0.67 (s, 1H), 0.38 (s, 3H), 0.32 (s, 3H) ppm. **^{13}C NMR** (126 MHz, $CDCl_3$, 298 K) δ 140.5, 135.9, 135.2, 134.3, 134.2, 129.9, 129.6, 129.1, 127.8, 126.0, 31.7, 29.798, 19.7, 19.3, −4.5, −5.0 ppm. **^{1}H/^{29}Si HMQC NMR** (500/99 MHz, $CDCl_3$, 298 K, optimized for *J* = 7 Hz): δ 7.55–7.49/−1.2, 2.21/−1.2, 1.83/−1.2, 0.38/−1.2, 0.32/−1.2 ppm. **HRMS** (APCI) m/z: $[M+H]^+$ calcd for $C_{18}H_{24}NSi^+$ 282.1672; found 282.1671. **IR** (ATR): ṽ 655, 697, 733, 775, 817, 1093, 1112, 1218, 1249, 1424, 2932, 2960 cm^{-1}. Optical rotation: $[\alpha]_D^{20}$ = −12.0 (*c* 1.0, CH_2Cl_2, 95% *ee*). The enantiomeric excess of (*S*)-**3fa** was determined by HPLC analysis on a chiral stationary phase (Daicel Chiralcel IA column, column temperature 20 °C, solvent heptane:*i*PrOH = 99:1, flow rate 0.6 mL/min): t_R = 37.6 min (major), t_R = 25.0 min (minor).

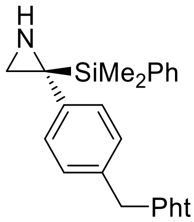

(*S*)-**3ga**
$C_{25}H_{24}N_2O_2Si$
M = 412.56 g/mol

(*S*)-2-(4-(2-(dimethyl(phenyl)silyl)aziridin-2-yl)benzyl)isoindoline-1,3-dione [(*S*)-**3ga**]: Prepared from 2-(4-(2*H*-azirin-3-yl)benzyl)isoindoline-1,3-dione (**1g**, 55.3 mg, 0.200 mmol, 1.00 equiv) according to **GP4** (*Method B*). The residue was purified by flash column chromatography on silica gel using *n*-hexane:ethyl acetate = 5:1 as the eluent to afford (*S*)-**3ga** as white solid (78.4 mg, 95% yield). $\mathbf{R}_{f}$ = 0.10 (*n*-hexane:ethyl acetate = 5:1). **M.P**. 67–69 °C. **^{1}H NMR** (500 MHz, $CDCl_3$, 298 K) δ 7.83 (dd, *J* = 5.4, 3.1 Hz, 2H), 7.68 (dd, *J* = 5.5, 3.0 Hz, 2H), 7.47 (dd, *J* = 7.8, 1.4 Hz, 2H), 7.41–7.36 (m, 1H), 7.34 (dd, *J* = 11.1, 4.4 Hz, 2H), 7.30 (d, *J* = 7.7 Hz, 2H), 7.17 (d, *J* = 7.6 Hz, 2H), 4.79 (d, *J* = 2.1 Hz, 2H), 1.83 (s, 1H), 1.73 (s, 1H), 0.77 (s, 1H), 0.34 (s, 3H), 0.27 (s, 3H) ppm. **^{13}C NMR** (126 MHz, $CDCl_3$, 298 K) δ 168.0, 143.0, 134.8,134.1, 132.1, 129.7, 128.9, 128.2, 127.9, 123.3, 41.3, 31.7, 29.7, −4.6, −5.1 ppm. **^{1}H/^{29}Si HMQC NMR** (500/99 MHz, $CDCl_3$, 298 K, optimized for *J* = 7 Hz): δ 7.47/−0.9, 0.37/−0.9, 0.31/−0.9 ppm. **HRMS** (APCI) m/z: $[M+H]^+$ calcd for $C_{25}H_{25}N_2O_2Si^+$ 413.1680; found 413.1680. **IR** (ATR): ṽ 691, 715, 771, 807, 830, 937, 1084, 1111, 1241, 1330, 1393, 1426, 1711, 1763, 2975 cm^{-1}. Optical rotation: $[\alpha]_D^{20}$ = −4.60 (*c* 1.0, CH_2Cl_2, 92% *ee*). The enantiomeric excess of (*S*)-**3ga** was determined by HPLC analysis on a chiral stationary phase (Daicel Chiralcel IA column, column temperature 20 °C, solvent heptane:*i*PrOH = 80:20, flow rate 1 mL/min): t_R = 25.9 min (major), t_R = 15.5 min (minor).

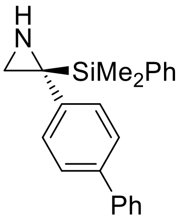

(*S*)-**3ha**
$C_{22}H_{23}NSi$
M = 329.52 g/mol

(*S*)-2-([1,1'-biphenyl]-4-yl)-2-(dimethyl(phenyl)silyl)aziridine [(*S*)-**3ha**]: Prepared from 3-([1,1'-biphenyl]-4-yl)-2*H*-azirine (**1h**, 38.7 mg, 0.200 mmol, 1.00 equiv) according to **GP4** (*Method A*). The residue was purified by flash column chromatography on silica gel using *n*-hexane:ethyl acetate = 10:1 as the eluent to afford (*S*)-**3ha** as colorless oil (61.3 mg, 93% yield). $\mathbf{R}_f$ = 0.10 (*n*-hexane:ethyl acetate = 10:1). **^{1}H NMR** (500 MHz, $CDCl_3$, 298 K) δ 7.64–7.62 (m, 2H), 7.57–7.51 (m, 2H), 7.50 (d, *J* = 7.9 Hz, 2H), 7.48–7.37 (m, 5H), 7.37–7.27 (m, 3H), 1.94 (s, 2H), 0.76 (s, 1H), 0.43 (s, 3H), 0.37 (s, 3H) ppm. **^{13}C NMR** (126 MHz, $CDCl_3$, 298 K) δ 142.4, 140.9, 138.9, 134.9, 134.2, 129.7, 129.0, 128.6, 127.9, 127.0, 126.9, 126.6, 31.8, 29.8, −4.5, −5.0 ppm. **^{1}H/^{29}Si HMQC NMR** (500/99 MHz, $CDCl_3$, 298 K, optimized for *J* = 7 Hz): δ 7.57–7.51/−0.7, 0.43/−0.7, 0.37/−0.7 ppm. **HRMS** (APCI) m/z: $[M+H]^+$ calcd for $C_{22}H_{24}NSi^+$ 330.1672; found 330.1674. **IR** (ATR): ṽ 658, 690, 731, 760, 775, 818, 876, 1110, 1245, 1424, 1484, 2961, 3042 cm^{-1}. Optical rotation: $[\alpha]_D^{20}$ = −8.15 (*c* 1.0, CH_2Cl_2, 92% *ee*). The enantiomeric excess of (*S*)-**3ha** was determined by HPLC analysis on a chiral stationary phase (Daicel Chiralcel IA column, column temperature 20 °C, solvent heptane:*i*PrOH = 98:2, flow rate 0.8 mL/min): t_R = 34.5 min (major), t_R = 25.0 min (minor).

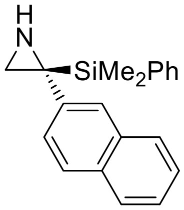

(*S*)-**3ia**
$C_{20}H_{21}NSi$
M = 303.48 g/mol

(*S*)-2-(dimethyl(phenyl)silyl)-2-(naphthalen-2-yl)aziridine [(*S*)-**3ia**]: Prepared from 3-(naphthalen-2-yl)-2*H*-azirine (**1i**, 33.4 mg, 0.200 mmol, 1.00 equiv) according to **GP4** (*Method A*). The residue was purified by flash column chromatography on silica gel using *n*-hexane:ethyl acetate = 10:1 as the eluent to afford (*S*)-**3ia** as white solid (55.8 mg, 92% yield). $\mathbf{R}_f$ = 0.10 (*n*-hexane:ethyl acetate = 10:1). **M.P.** 57–59 °C **^{1}H NMR** (500 MHz, $CDCl_3$, 298 K) δ 7.81–7.74 (m, 2H), 7.72 (d, *J* = 8.5 Hz, 1H), 7.68 (s, 1H), 7.55–7.50 (m, 2H), 7.47–7.40 (m, 3H), 7.37 (m, 3H), 1.96 (s, 1H), 1.88 (s, 1H), 0.89 (s, 1H), 0.42 (s, 3H), 0.34 (s, 3H) ppm. **^{13}C NMR** (126 MHz, $CDCl_3$, 298 K) δ 140.9, 134.9, 134.3, 133.3, 132.1, 129.8, 127.9, 127.6, 127.6, 127.4, 127.2, 127.0, 125.8, 125.3, 32.4, 30.0, −4.5, −5.0 ppm. **^{1}H/^{29}Si HMQC NMR** (500/99 MHz, $CDCl_3$, 298 K, optimized for *J* = 7 Hz): δ 7.55–7.50/−0.7, 1.96/−0.7, 1.88/−0.7, 0.42/−0.7, 0.34/−0.7 ppm. **HRMS** (APCI) m/z: $[M+H]^+$ calcd for $C_{20}H_{22}NSi^+$ 304.1517; found 304.1517. **IR** (ATR): ṽ 653, 674, 700, 737, 774, 811, 830, 1111, 1142, 1247, 1331, 1425, 1454, 1471, 1517, 2974 cm^{-1}. Optical rotation: $[\alpha]_D^{20}$ = −8.45 (*c* 1.0, CH_2Cl_2, 91% *ee*). The enantiomeric excess of (*S*)-**3ia** was determined by HPLC analysis on a chiral stationary phase (Daicel Chiralcel IA column, column temperature 20 °C, solvent heptane:*i*PrOH = 99:1, flow rate 1 mL/min): t_R = 32.5 min (major), t_R = 23.6 min (minor).

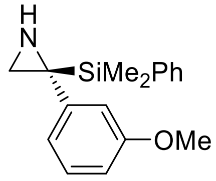

(*S*)-**3ja**
$C_{17}H_{21}NOSi$
M = 283.45 g/mol

(*S*)-2-(dimethyl(phenyl)silyl)-2-(3-methoxyphenyl)aziridine [(*S*)-**3ja**]: Prepared from 3-(3-methoxyphenyl)-2*H*-azirine (**1j**, 29.4 mg, 0.200 mmol, 1.00 equiv) according to **GP4** (*Method A*). The residue was purified by flash column chromatography on silica gel using *n*-hexane:ethyl acetate = 5:1 as the eluent to afford (*S*)-**3ja** as colorless oil (46.5 mg, 82% yield). $\mathbf{R_f}$ = 0.10 (*n*-hexane:ethyl acetate = 5:1). **^{1}H NMR** (500 MHz, $CDCl_3$, 298 K) δ 7.50 (dd, *J* = 7.7, 1.4 Hz, 2H), 7.44–7.33 (m, 3H), 7.15 (t, *J* = 7.8 Hz, 1H), 6.82 (d, *J* = 7.4 Hz, 1H), 6.78–6.69 (m, 2H), 3.72 (s, 3H), 1.88 (s, 1H), 1.82 (s, 1H), 0.50 (s, 1H), 0.38 (s, 3H), 0.31 (s, 3H). **^{13}C NMR** (126 MHz, $CDCl_3$, 298 K) δ 159.2, 144.8, 135.0, 134.2, 129.687, 128.9, 127.9, 121.0, 113.9, 112.1, 55.0, 32.2, 29.8, −4.6, −5.1. **^{29}Si DEPT NMR** (99 MHz, $CDCl_3$, 298 K): δ −22.0 ppm. **HRMS** (APCI) m/z: $[M+H]^+$ calcd for $C_{17}H_{22}NOSi^+$ 284.1465; found 284.1465. **IR** (ATR): ṽ 661, 697, 735, 775, 814, 1043, 1110, 1222, 1248, 1426, 1484, 1579, 1597, 2955 cm^{-1}. Optical rotation: $[\alpha]_D^{20}$ = −13.0 (*c* 1.0, CH_2Cl_2, 92% *ee*). The enantiomeric excess of (*S*)-**3ja** was determined by HPLC analysis on a chiral stationary phase (Daicel Chiralcel IA column, column temperature 20 °C, solvent heptane:*i*PrOH = 99:1, flow rate 0.8 mL/min): t_R = 36.7 min (major), t_R = 25.5 min (minor).

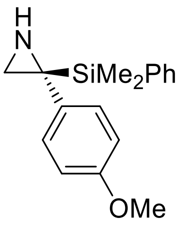

(S)-**3ka**
$C_{17}H_{21}NOSi$
M = 283.45 g/mol

(*S*)-2-(dimethyl(phenyl)silyl)-2-(4-methoxyphenyl)aziridine [(*S*)-**3ka**]: Prepared from 3-(4-methoxyphenyl)-2*H*-azirine (**1k**, 29.4 mg, 0.200 mmol, 1.00 equiv) according to **GP4** (*Method A*). The residue was purified by flash column chromatography on silica gel using *n*-hexane:ethyl acetate = 5:1 as the eluent to afford (*S*)-**3ka** as colorless oil (35.7 mg, 63% yield). $\mathbf{R_f}$ = 0.10 (*n*-hexane:ethyl acetate = 5:1). **^{1}H NMR** (500 MHz, $CDCl_3$, 298 K) δ 7.52–7.47 (m, 1H), 7.13 (d, *J* = 8.6 Hz, 1H), 6.78 (d, *J* = 8.7 Hz, 1H), 3.77 (s, 2H), 1.84 (s, 1H), 1.77 (s, 1H), 0.67 (s, 1H), 0.36 (s, 2H), 0.30 (s, 2H) ppm. **^{13}C NMR** (126 MHz, $CDCl_3$, 298 K) δ 157.9, 135.3, 135.2, 134.2, 129.7, 129.6, 127.8, 113.4, 55.2, 31.3, 29.8, −4.6, −5.0 ppm. **^{1}H/^{29}Si HMQC NMR** (500/99 MHz, $CDCl_3$, 298 K, optimized for *J* = 7 Hz): δ 7.52–7.47/−1.1, 0.36/−1.1, 0.30/−1.1 ppm. **HRMS** (APCI) m/z: $[M+H]^+$ calcd for $C_{17}H_{22}NOSi^+$ 284.1465; found 284.1464. **IR** (ATR): ṽ 655, 699, 734, 774, 803, 819, 880, 1032, 1104, 1172, 1244, 1426, 1456, 1506, 1606, 2958 cm^{-1}. Optical rotation: $[\alpha]_D^{20}$ = −3.12 (*c* 1.0, CH_2Cl_2, 90% *ee*). The enantiomeric excess of (*S*)-**3ka** was determined by HPLC analysis on a chiral stationary phase (Daicel Chiralcel IC column, column temperature 25 °C, solvent heptane:*i*PrOH = 96:4, flow rate 1 mL/min): t_R = 19.4 min (major), t_R = 31.4 min (minor).

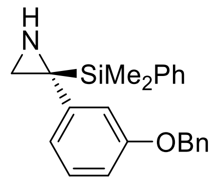

(*S*)-**3la**
$C_{23}H_{25}NOSi$
M = 359.54 g/mol

(*S*)-2-(3-(benzyloxy)phenyl)-2-(dimethyl(phenyl)silyl)aziridine [(*S*)-**3la**]: Prepared from 3-(3-(benzyloxy)phenyl)-2*H*-azirine (**1l**, 44.7 mg, 0.200 mmol, 1.00 equiv) according to **GP4** (*Method B*). The residue was purified by flash column chromatography on silica gel using *n*-hexane:ethyl acetate = 5:1 as the eluent to afford (*S*)-**3la** as white solid (50.3 mg, 70% yield). $\mathbf{R_f}$ = 0.10 (*n*-hexane:ethyl acetate = 5:1). **M.P**. 63–65 °C. **^{1}H NMR** (500 MHz, $CDCl_3$, 298 K) δ 7.53–7.47 (m, 2H), 7.46–7.42 (m, 2H), 7.41–7.38 (m, 3H), 7.38–7.30 (m, 3H), 7.14 (d, *J* = 8.6 Hz, 2H), 6.86 (d, *J* = 8.7 Hz, 2H), 5.03 (s, 2H), 1.85 (s, 1H), 1.78 (s, 1H), 0.86 (s, 1H), 0.37 (s, 3H), 0.31 (s, 3H) ppm. **^{13}C NMR** (126 MHz, $CDCl_3$, 298 K) δ 157.2, 137.1, 135.6, 135.1, 134.2, 129.7, 129.6, 128.5, 127.9, 127.5, 114.4, 70.0, 31.4, 29.8, −4.6, −5.1 ppm. **^{1}H/^{29}Si HMQC NMR** (500/99 MHz, $CDCl_3$, 298 K, optimized for *J* = 7 Hz): δ 7.41–7.38/−1.1, 0.37/−1.1, 0.31/−1.1 ppm. **HRMS** (APCI) m/z: $[M+H]^+$ calcd for $C_{23}H_{26}NOSi^+$ 360.1778; found 360.1779. **IR** (ATR): ṽ 656, 697, 744, 776, 807, 825, 861, 1008, 1107, 1174, 1232, 1386, 1427, 1453, 1506, 1603, 2922, 2955 cm^{-1}. Optical rotation: $[\alpha]_D^{20}$ = −5.82 (*c* 1.0, CH_2Cl_2, 95% *ee*). The enantiomeric excess of (*S*)-**3la** was determined by HPLC analysis on a chiral stationary phase (Daicel Chiralcel IA column, column temperature 20 °C, solvent heptane:*i*PrOH = 95:5, flow rate 0.8 mL/min): t_R = 22.6 min (major), t_R = 16.8 min (minor).

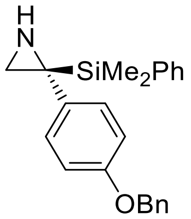

(*S*)-**3ma**
$C_{23}H_{25}NOSi$
M = 359.54 g/mol

(*S*)-2-(4-(benzyloxy)phenyl)-2-(dimethyl(phenyl)silyl)aziridine [(*S*)-**3ma**]: Prepared from 3-(4-(benzyloxy)phenyl)-2*H*-azirine (**1m**, 44.7 mg, 0.200 mmol, 1.00 equiv) according to **GP4** (*Method A*). The residue was purified by flash column chromatography on silica gel using *n*-hexane:ethyl acetate = 5:1 as the eluent to afford (*S*)-**3ma** as colorless oil (50.3 mg, 70% yield). **R_f** = 0.10 (*n*-hexane:ethyl acetate = 5:1). **^{1}H NMR** (500 MHz, $CDCl_3$, 298 K) δ 7.53–7.47 (m, 2H), 7.45–7.35 (m, 7H), 7.35–7.29 (m, 1H), 7.16 (t, *J* = 8.0 Hz, 1H), 6.88–6.76 (m, 3H), 4.97 (s, 2H), 1.88 (s, 1H), 1.81 (s, 1H), 0.94 (s, 1H), 0.37 (s, 3H), 0.30 (s, 3H) ppm. **^{13}C NMR** (126 MHz, $CDCl_3$, 298 K) δ 158.4, 144.8, 137.1, 135.0, 134.3, 129.7, 128.9, 128.5, 127.9, 127.8, 127.4, 121.3, 114.9, 113.0, 69.8, 32.2, 29.8, −4.6, −5.1 ppm. **^{1}H/^{29}Si HMQC NMR** (500/99 MHz, $CDCl_3$, 298 K, optimized for *J* = 7 Hz): δ 7.53–7.47/−0.7, 0.37/−0.7, 0.31/−0.7 ppm. **HRMS** (APCI) m/z: $[M+H]^+$ calcd for $C_{23}H_{26}NOSi^+$ 360.1778; found 360.1780. **IR** (ATR): ṽ 663, 695, 733, 776, 814, 1025, 1110, 1208, 1248, 1426, 1483, 1578, 2954 cm^{-1}. Optical rotation: $[\alpha]_D^{20}$ = −13.6 (*c* 1.0, CH_2Cl_2, 95% *ee*). The enantiomeric excess of (*S*)-**3ma** was determined by HPLC analysis on a chiral stationary phase (Daicel Chiralcel IA column, column temperature 20 °C, solvent heptane:*i*PrOH = 95:5, flow rate 0.7 mL/min): t_R = 35.0 min (major), t_R = 19.9 min (minor).

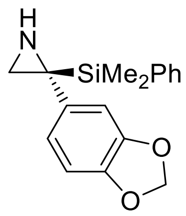

(*S*)-**3na**
$C_{17}H_{19}NO_2Si$
M = 297.43 g/mol

(*S*)-2-(benzo[d][1,3]dioxol-5-yl)-2-(dimethyl(phenyl)silyl)aziridine [(*S*)-**3na**]: Prepared from 3-(benzo[*d*][1,3]dioxol-5-yl)-2*H*-azirine (**1n**, 32.2 mg, 0.200 mmol, 1.00 equiv) according to **GP4** (*Method B*). The residue was purified by flash column chromatography on silica gel using *n*-hexane:ethyl acetate = 5:1 as the eluent to afford (*S*)-**3na** as white solid (52.9 mg, 89% yield). $\mathbf{R}_f$ = 0.10 (*n*-hexane:ethyl acetate = 5:1). **M**.**P**. 41–43 °C. **^{1}H NMR** (500 MHz, $CDCl_3$, 298 K) δ 7.52–7.47 (m, 2H), 7.44–7.32 (m, 3H), 6.72 (s, 1H), 6.68 (s, 2H), 5.90 (dd, *J* = 3.1, 1.5 Hz, 2H), 1.82 (s, 1H), 1.76 (s, 1H),0.66 (s, 1H), 0.37 (s, 3H), 0.31 (s, 3H) ppm. **^{13}C NMR** (126 MHz, $CDCl_3$, 298 K) δ 147.1, 145.8, 137.2, 134.9, 134.2, 129.7, 127.9, 121.7, 109.2, 107.8, 100.7, 31.8, 29.9, −4.6, −5.1 ppm. **^{1}H/^{29}Si HMQC NMR** (500/99 MHz, $CDCl_3$, 298 K, optimized for *J* = 7 Hz): δ 7.52–7.47/−1.1, 0.37/−1.1, 0.31/−1.1 ppm. **HRMS** (APCI) m/z: $[M+H]^+$ calcd for $C_{17}H_{20}NO_2Si^+$ 298.1258; found 298.1259. **IR** (ATR): ṽ 661, 698, 732, 774, 817, 872, 937, 1013, 1036, 1085, 1099, 1111, 1218, 1249, 1426, 1478, 1501, 2878, 2955 cm^{-1}. Optical rotation: $[\alpha]_D^{20}$ = −10.1 (*c* 1.0, CH_2Cl_2, 96% *ee*). The enantiomeric excess of (*S*)-**3na** was determined by HPLC analysis on a chiral stationary phase (Daicel Chiralcel IA column, column temperature 20 °C, solvent heptane:*i*PrOH = 95:5, flow rate 0.7 mL/min): t_R = 16.1 min (major), t_R = 15.0 min (minor).

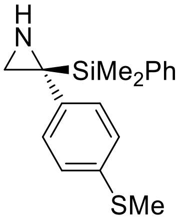

(*S*)-**3oa**
$C_{17}H_{21}NSSi$
M = 299.51 g/mol

(*S*)-2-(dimethyl(phenyl)silyl)-2-(4-(methylthio)phenyl)aziridine [(*S*)-**3oa**]: Prepared from 3-(4-(methylthio)phenyl)-2*H*-azirine (**1o**, 32.6 mg, 0.200 mmol, 1.00 equiv) according to **GP4** (*Method B*). The residue was purified by flash column chromatography on silica gel using *n*-hexane:ethyl acetate = 5:1 as the eluent to afford (*S*)-**3oa** as white solid (55.1 mg, 92% yield). **R*f*** = 0.10 (*n*-hexane:ethyl acetate = 5:1). **M.P**. 74–76 °C. **^{1}H NMR** (500 MHz, $CDCl_3$, 298 K) δ 7.53–7.44 (m, 2H), 7.44–7.33 (m, 3H), 7.14 (s, 4H), 2.45 (s, 3H), 1.88 (s, 1H), 1.77 (s, 1H), 0.76 (s, 1H), 0.36 (s, 3H), 0.30 (s, 3H) ppm. **^{13}C NMR** (126 MHz, $CDCl_3$, 298 K) δ 140.5, 135.7, 134.9, 134.2, 129.7, 129.1, 127.9, 126.5, 29.8, 16.1, −4.6, −5.1. **^{1}H/^{29}Si HMQC NMR** (500/99 MHz, $CDCl_3$, 298 K, optimized for *J* = 7 Hz): δ 7.44–7.33/−1.0, 0.36/−1.0, 0.30/−1.0 ppm. **HRMS** (APCI) m/z: $[M+H]^+$ calcd for $C_{17}H_{22}NSSi^+$ 300.1237; found 300.1236. **IR** (ATR): ṽ 703, 736, 775, 811, 829, 875, 969, 1018, 1090, 1112, 1136, 1212, 1248, 1425, 1489, 2917, 2960 cm^{-1}. Optical rotation: $[\alpha]_D^{20}$ = −9.91 (*c* 1.0, CH_2Cl_2, 95% *ee*). The enantiomeric excess of (*S*)-**3oa** was determined by HPLC analysis on a chiral stationary phase (Daicel Chiralcel IA column, column temperature 20 °C, solvent heptane:*i*PrOH = 95:5, flow rate 0.8 mL/min): t_R = 17.9 min (major), t_R = 12.7 min (minor).

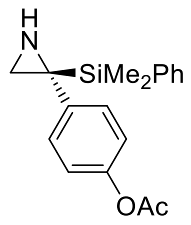

(*S*)-**3pa**
$C_{18}H_{21}NO_2Si$
M = 311.46 g/mol

(*S*)-4-(2-(dimethyl(phenyl)silyl)aziridin-2-yl)phenyl acetate [(*S*)-**3pa**]: Prepared from 4-(2*H*-azirin-3-yl)phenyl acetate (**1p**, 35.0 mg, 0.200 mmol, 1.00 equiv) according to **GP4** (*Method A*). The residue was purified by flash column chromatography on silica gel using *n*-hexane:ethyl acetate = 5:1 as the eluent to afford (*S*)-**3pa** as white solid (60.4 mg, 97% yield). **R*f*** = 0.10 (*n*-hexane:ethyl acetate = 10:1). **M**.**P**. 53–55 °C. **^{1}H NMR** (500 MHz, $CDCl_3$, 298 K) δ 7.50–7.46 (m, 2H), 7.43–7.34 (m, 3H), 7.21 (d, *J* = 8.4 Hz, 2H), 6.96 (d, *J* = 8.5 Hz, 2H), 2.27 (s, 3H), 1.87 (s, 1H), 1.78 (s, 1H), 0.37 (s, 3H), 0.30 (s, 3H) ppm. **^{13}C NMR** (126 MHz, $CDCl_3$, 298 K) δ 169.3, 148.9, 140.7, 134.7, 134.2, 129.8, 129.5, 127.9, 120.9, 31.2, 29.8, 21.1, −4.6, −5.1 ppm. **^{1}H/^{29}Si HMQC NMR** (500/99 MHz, $CDCl_3$, 298 K, optimized for *J* = 7 Hz): δ 7.50–7.46/−0.8, 0.37/−0.8, 0.30/−0.8 ppm. **HRMS** (APCI) m/z: $[M+H]^+$ calcd for $C_{18}H_{22}NO_2Si^+$ 312.1414; found 312.1415. **IR** (ATR): ṽ 669, 703, 738, 776, 822, 878, 912, 1009, 1099, 1133, 1191, 1214, 1255, 1373, 1427, 1502, 1747, 2961 cm^{-1}. Optical rotation: $[\alpha]_D^{20}$ = −13.0 (*c* 1.0, CH_2Cl_2, 92% *ee*). The enantiomeric excess of (*S*)-**3pa** was determined by HPLC analysis on a chiral stationary phase (Daicel Chiralcel IA column, column temperature 20 °C, solvent heptane:*i*PrOH = 96:4, flow rate 0.8 mL/min): t_R = 25.7 min (major), t_R = 22.1 min (minor).

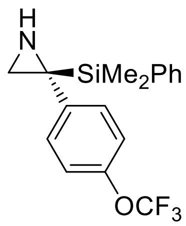

(S)-**3qa**
$C_{17}H_{18}F_3NOSi$
M = 337.42 g/mol

(*S*)-2-(dimethyl(phenyl)silyl)-2-(4-(trifluoromethoxy)phenyl)aziridine [(*S*)-**3qa**]: Prepared from 3-(4-(trifluoromethoxy)phenyl)-2*H*-azirine (**1q**, 40.2 mg, 0.200 mmol, 1.00 equiv) according to **GP4** (*Method A*). The residue was purified by flash column chromatography on silica gel using *n*-hexane:ethyl acetate = 10:1 as the eluent to afford (*S*)-**3qa** as colorless oil (52.6 mg, 78% yield). $\mathbf{R_f}$ = 0.10 (*n*-hexane:ethyl acetate = 10:1). **^{1}H NMR** (500 MHz, $CDCl_3$, 298 K) δ 7.50–7.46 (m, 2H), 7.45–7.40 (m, 1H), 7.40–7.35 (m, 2H), 7.24 (d, J = 8.4 Hz, 2H), 7.08 (d, J = 7.9 Hz, 2H), 1.93 (s, 1H), 1.79 (s, 1H), 0.65 (s, 1H), 0.38 (s, 3H), 0.31 (s, 3H) ppm. **^{13}C NMR** (126 MHz, $CDCl_3$, 298 K) δ 147.5, 142.1, 134.4, 134.2, 129.9, 128.0, 123.53, 120.47, 120.47 (q, J = 256.8 Hz), 31.4, 29.8, −4.7, −5.2 ppm. **^{19}F NMR** (471 MHz, $CDCl_3$): δ −57.9 ppm. **^{1}H/^{29}Si HMQC NMR** (500/99 MHz, $CDCl_3$, 298 K, optimized for J = 7 Hz): δ 7.50–7.46/−0.5, 0.38/−0.5, 0.31/−0.5 ppm. **HRMS** (APCI) m/z: $[M+H]^+$ calcd for $C_{17}H_{19}F_3NOSi^+$ 338.1182; found 338.1185. **IR** (ATR): ṽ 660, 700, 735, 776, 818, 834, 878, 1012, 1101, 1154, 1206, 1248, 1426, 1504, 2960, 3045 cm^{-1}. Optical rotation: $[\alpha]_D^{20}$ = −11.7 (*c* 1.0, CH_2Cl_2, 90% *ee*). The enantiomeric excess of (*S*)-**3qa** was determined by HPLC analysis on a chiral stationary phase (Daicel Chiralcel IA column, column temperature 20 °C, solvent heptane:*i*PrOH = 99:1, flow rate 0.8 mL/min): t_R = 29.4 min (major), t_R = 18.8 min (minor).

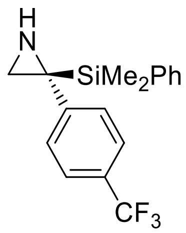

(*S*)-**3ra**
$C_{17}H_{18}F_3NSi$
M = 321.42 g/mol

(*S*)-2-(dimethyl(phenyl)silyl)-2-(4-(trifluoromethyl)phenyl)aziridine [(*S*)-**3ra**]: Prepared from 3-(4-(trifluoromethyl)phenyl)-2*H*-azirine (**1r**, 37.0 mg, 0.200 mmol, 1.00 equiv) according to **GP4** (*Method A*). The residue was purified by flash column chromatography on silica gel using *n*-hexane:ethyl acetate = 10:1 as the eluent to afford (*S*)-**3ra** as colorless oil (52.7 mg, 82% yield). **R*f*** = 0.10 (*n*-hexane:ethyl acetate = 10:1). **^{1}H NMR** (500 MHz, $CDCl_3$, 298 K) δ 7.52–7.47 (m, 4H), 7.46–7.41 (m, 1H), 7.41–7.37 (m, 2H), 7.34 (d, *J* = 7.8 Hz, 2H), 1.96 (s, 1H), 1.79 (s, 1H), 0.68 (s, 1H), 0.40 (s, 3H), 0.31 (s, 3H) ppm. **^{13}C NMR** (126 MHz, $CDCl_3$, 298 K) δ 147.6, 134.2, 130.0, 128.9, 128.3 (q, *J* = 32.1 Hz), 128.1, 124.9, 124.9 (q, *J* = 3.5 Hz), 124.9, 124.9, 124.8, 124.3 (q, *J* = 271.7 Hz),32.0, 29.9, −4.6, −5.2 ppm. **^{19}F NMR** (471 MHz, $CDCl_3$, 298 K): δ −62.3 ppm. **^{1}H/^{29}Si HMQC NMR** (500/99 MHz, $CDCl_3$, 298 K, optimized for *J* = 7 Hz): δ 7.52–7.47/−0.4, 0.40/−0.4, 0.31/−0.4 ppm. **HRMS** (APCI) m/z: $[M+H]^+$ calcd for $C_{17}H_{19}F_3NSi^+$ 322.1233; found 322.1235. **IR** (ATR): ṽ 671, 700, 734, 776, 813, 831, 879, 1014, 1065, 1105, 1160, 1251, 1321, 1614, 2957 cm^{-1}. Optical rotation: $[\alpha]_D^{20}$ = −18.3 (*c* 1.0, CH_2Cl_2, 92% *ee*). The enantiomeric excess of (*S*)-**3ra** was determined by HPLC analysis on a chiral stationary phase (Daicel Chiralcel IA column, column temperature 20 °C, solvent heptane:*i*PrOH = 99:1, flow rate 1.0 mL/min): t_R = 26.9 min (major), t_R = 16.7 min (minor).

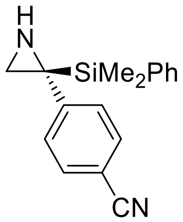

(*S*)-**3sa**
$C_{17}H_{18}N_2Si$
M = 278.43 g/mol

(*S*)-2-(dimethyl(phenyl)silyl)-2-(4-isocyanophenyl)aziridine [(*S*)-**3sa**]: Prepared from 3-(4-isocyanophenyl)-2*H*-azirine (**1s**, 28.4 mg, 0.200 mmol, 1.00 equiv) according to **GP4** (*Method A*). The residue was purified by flash column chromatography on silica gel using *n*-hexane:ethyl acetate = 5:1 as the eluent to afford (*S*)-**3sa** as colorless oil (45.1 mg, 81% yield). $\mathbf{R_f}$ = 0.10 (*n*-hexane:ethyl acetate = 5:1). **^{1}H NMR** (500 MHz, $CDCl_3$, 298 K) δ 7.51 (s, 1H), 7.50 (s, 1H), 7.48–7.44 (m, 2H), 7.43–7.40 (m, 1H), 7.40–7.35 (m, 2H), 7.32 (s, 1H), 7.30 (s, 1H), 2.00 (s, 1H), 1.80 (s, 1H), 0.64 (s, 1H), 0.38 (s, 3H), 0.29 (s, 3H) ppm. **^{13}C NMR** (126 MHz, $CDCl_3$, 298 K) δ 149.3, 134.1, 133.8, 131.7, 130.1, 129.3, 128.1, 119.0, 109.8, 32.3, 30.0, −4.7, −5.2 ppm. **^{1}H/^{29}Si HMQC NMR** (500/99 MHz, $CDCl_3$, 298 K, optimized for *J* = 7 Hz): δ 7.48–7.44/−2.1, 2.00/−2.1, −1.80/−2.1 ppm. **HRMS** (APCI) m/z: $[M+H]^+$ calcd for $C_{17}H_{19}N_2Si^+$ 279.1312; found 279.1311. **IR** (ATR): ṽ 674, 699, 735, 777, 813, 833, 879, 981, 1009, 1110, 1145, 1251, 1427, 1450, 1471, 1500, 1603, 2225, 2975 cm^{-1}. Optical rotation: $[\alpha]_D^{20}$ = −12.1 (*c* 1.0, CH_2Cl_2, 94% *ee*). The enantiomeric excess of (*S*)-**3sa** was determined by HPLC analysis on a chiral stationary phase (Daicel Chiralcel IA column, column temperature 20 °C, solvent heptane:*i*PrOH = 90:10, flow rate 0.7 mL/min): t_R = 19.3 min (major), t_R = 14.9 min (minor).

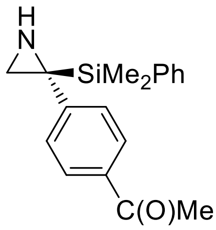

(*S*)-**3ta**
$C_{18}H_{21}NOSi$
M = 295.46 g/mol

(*S*)-1-(4-(2-(dimethyl(phenyl)silyl)aziridin-2-yl)phenyl)ethan-1-one [(*S*)-**3ta**]: Prepared from 1-(4-(2*H*-azirin-3-yl)phenyl)ethan-1-one (**1t**, 31.8 mg, 0.200 mmol, 1.00 equiv) according to **GP4** (*Method A*). The residue was purified by flash column chromatography on silica gel using *n*-hexane:ethyl acetate = 3:1 as the eluent to afford (*S*)-**3ta** as colorless oil (53.2mg, 90% yield). **R*f*** = 0.10 (*n*-hexane:ethyl acetate = 3:1). **^{1}H NMR** (500 MHz, $CDCl_3$) δ 7.83 (d, *J* = 8.0 Hz, 2H), 7.47 (d, *J* = 6.6 Hz, 2H), 7.44–7.34 (m, 3H), 7.31 (d, *J* = 7.9 Hz, 2H), 2.56 (s, 3H), 1.95 (s, 1H), 1.79 (s, 1H), 0.86 (s, 1H), 0.38 (s, 3H), 0.30 (s, 3H) ppm. **^{13}C NMR** (126 MHz, $CDCl_3$) δ 197.7, 135.1, 134.2, 129.9, 128.7, 128.1, 128.0, 32.3, 29.9, 26.5, −4.6, −5.1 ppm. **^{1}H/^{29}Si HMQC NMR** (500/99 MHz, $CDCl_3$, 298 K, optimized for *J* = 7 Hz): δ 7.47/−0.3, 0.38/−0.3, 0.30/−0.3 ppm. **HRMS** (APCI) m/z: $[M+H]^+$ calcd for $C_{18}H_{22}NOSi^+$ 296.1465; found 296.1466. **IR** (ATR): ṽ 700, 733, 774, 815, 877, 957, 1112, 1248, 1269, 1399, 1598, 1676 cm^{-1}. Optical rotation: $[\alpha]_D^{20}$ = −39.4 (*c* 1.0, CH_2Cl_2, 95% *ee*). The enantiomeric excess of (*S*)-**3ta** was determined by HPLC analysis on a chiral stationary phase (Daicel Chiralcel IA column, column temperature 20 °C, solvent heptane:*i*PrOH = 90:10, flow rate 1.0 mL/min): t_R = 15.5 min (major), t_R = 11.1 min (minor).

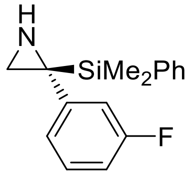

(*S*)-**3ua**
$C_{16}H_{18}FNSi$
M = 271.41 g/mol

(*S*)-2-(dimethyl(phenyl)silyl)-2-(3-fluorophenyl)aziridine [(*S*)-**3ua**]: Prepared from 3-(3-fluorophenyl)-2*H*-azirine (**1u**, 27.0 mg, 0.200 mmol, 1.00 equiv) according to **GP4** (*Method A*). The residue was purified by flash column chromatography on silica gel using *n*-hexane:ethyl acetate = 10:1 as the eluent to afford (*S*)-**3ua** as colorless oil (32.6 mg, 60% yield). **R_f** = 0.10 (*n*-hexane:ethyl acetate = 10:1). **^{1}H NMR** (500 MHz, $CDCl_3$, 298 K) δ 7.51–7.46 (m, 2H), 7.45–7.40 (m, 1H), 7.40–7.35 (m, 2H), 7.23–7.15 (m, 1H), 7.00 (d, *J* = 7.5 Hz, 1H), 6.94 (d, *J* = 9.7 Hz, 1H), 6.85 (td, *J* = 8.5, 2.1 Hz, 1H), 1.90 (s, 1H), 1.80 (s, 1H), 0.62 (s, 1H), 0.39 (s, 3H), 0.31 (s, 3H) ppm. **^{13}C NMR** (126 MHz, $CDCl_3$, 298 K) δ 162.6 (d, *J* = 245.8 Hz), 134.4, 134.2, 129.9, 129.4 (d, *J* = 8.3 Hz), 128.0, 124.2, 115.5 (d, *J* = 21.7 Hz), 113.0 (d, *J* = 21.3 Hz), 32.0, 29.8, −4.5, −5.1 ppm. **^{19}F NMR** (471 MHz, $CDCl_3$, 298 K): δ −113.7 ppm. **^{1}H/^{29}Si HMQC NMR** (500/99 MHz, $CDCl_3$, 298 K, optimized for *J* = 7 Hz): δ 7.51–7.46/−0.5, 0.38/−0.5, 0.31/−0.5 ppm. **HRMS** (APCI) m/z: $[M+H]^+$ calcd for $C_{16}H_{19}FNSi^+$ 272.1265; found 272.1266. **IR** (ATR): ṽ 660, 694, 735, 776, 815, 879, 922, 1109, 1198, 1249, 1427, 1483, 1581, 1609, 2956 cm^{-1}. Optical rotation: $[\alpha]_D^{20}$ = −11.8 (*c* 1.0, CH_2Cl_2, 92% *ee*). The enantiomeric excess of (*S*)-**3ua** was determined by HPLC analysis on a chiral stationary phase (Daicel Chiralcel IA column, column temperature 20 °C, solvent heptane:*i*PrOH = 99:1, flow rate 0.7 mL/min): t_R = 25.7 min (major), t_R = 19.1 min (minor).

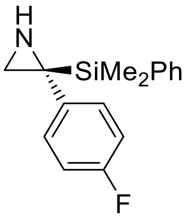

(*S*)-**3va**
$C_{16}H_{18}FNSi$
M = 271.41 g/mol

(*S*)-2-(dimethyl(phenyl)silyl)-2-(4-fluorophenyl)aziridine [(*S*)-**3va**]: Prepared from 3-(4-fluorophenyl)-2*H*-azirine (**1v**, 27.0 mg, 0.200 mmol, 1.00 equiv) according to **GP4** (*Method A*). The residue was purified by flash column chromatography on silica gel using *n*-hexane:ethyl acetate = 10:1 as the eluent to afford (*S*)-**3va** as colorless oil (46.1 mg, 85% yield). **R*f*** = 0.10 (*n*-hexane:ethyl acetate = 10:1). **^{1}H NMR** (500 MHz, $CDCl_3$, 298 K) δ 7.51–7.45 (m, 2H), 7.44–7.39 (m, 1H), 7.39–7.34 (m, 2H), 7.15 (dd, *J* = 8.5, 5.5 Hz, 2H), 6.92 (t, *J* = 8.7 Hz, 2H), 1.90 (s, 1H), 1.78 (s, 1H), 0.77 (s, 1H), 0.37 (s, 3H), 0.30 (s, 3H) ppm. **^{13}C NMR** (126 MHz, $CDCl_3$, 298 K) δ 161.3 (d, *J* = 244.1 Hz), 138.9, 134.7, 134.2, 130.1 (d, *J* = 8.0 Hz), 129.8, 127.9, 114.7 (d, *J* = 21.4 Hz), 31.4, 29.8, −4.7, −5.2 ppm. **^{19}F NMR** (471 MHz, $CDCl_3$, 298 K): δ −116.6 ppm. **^{1}H/^{29}Si HMQC NMR** (500/99 MHz, $CDCl_3$, 298 K, optimized for *J* = 7 Hz): δ 7.51–7.45/−0.8, 0.37/−0.8, 0.30/−0.8 ppm. **HRMS** (APCI) m/z: $[M+H]^+$ calcd for $C_{16}H_{19}FNSi^+$ 272.1265; found 272.1265. **IR** (ATR): ṽ 655, 700, 734, 775, 809, 832, 879, 1011, 1092, 1213, 1246, 1426, 1501, 2956 cm^{-1}. Optical rotation: $[\alpha]_D^{20}$ = −13.9 (*c* 1.0, CH_2Cl_2, 92% *ee*). The enantiomeric excess of (*S*)-**3va** was determined by HPLC analysis on a chiral stationary phase (Daicel Chiralcel IA column, column temperature 20 °C, solvent heptane:*i*PrOH = 99:1, flow rate 0.7 mL/min): t_R = 35.3 min (major), t_R = 25.6 min (minor).

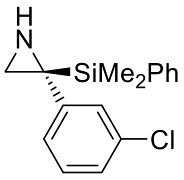

(S)-**3wa**
$C_{16}H_{18}ClNSi$
M = 287.86 g/mol

(*S*)-2-(3-chlorophenyl)-2-(dimethyl(phenyl)silyl)aziridine [(*S*)-**3wa**]: Prepared from 3-(3-chlorophenyl)-2*H*-azirine (**1w**, 30.3 mg, 0.200 mmol, 1.00 equiv) according to **GP4** (*Method A*). The residue was purified by flash column chromatography on silica gel using *n*-hexane:ethyl acetate = 10:1 as the eluent to afford (*S*)-**3wa** as colorless oil (50.1 mg, 87% yield). **R*f*** = 0.10 (*n*-hexane:ethyl acetate = 10:1). **^{1}H NMR** (500 MHz, $CDCl_3$, 298 K) δ 7.50–7.45 (m, 2H), 7.45–7.40 (m, 1H), 7.40–7.36 (m, 2H), 7.22 (s, 1H), 7.18–7.07 (m, 3H), 1.88 (s, 1H), 1.77 (s, 1H), 0.39 (s, 3H), 0.31 (s, 3H) ppm. **^{13}C NMR** (126 MHz, $CDCl_3$, 298 K) δ 134.3, 134.2, 133.8, 130.0, 129.2, 128.7, 128.0, 126.8, 126.3, 29.7, −5.1 ppm. **^{1}H/^{29}Si HMQC NMR** (500/99 MHz, $CDCl_3$, 298 K, optimized for J = 7 Hz): δ 7.50–7.45/−0.7, 0.39/−0.7, 0.31/−0.7 ppm. **HRMS** (APCI) m/z: $[M+H]^+$ calcd for $C_{16}H_{19}ClNSi^+$ 288.0970; found 288.0970. **IR** (ATR): ṽ 657, 691, 734, 776, 824, 881, 1016, 1076, 1110, 1249, 1408, 1425, 1472, 1564, 1591, 2955 cm^{-1}. Optical rotation: $[\alpha]_D^{20}$ = −6.05 (*c* 1.0, CH_2Cl_2, 92% *ee*). The enantiomeric excess of (*S*)-**3wa** was determined by HPLC analysis on a chiral stationary phase (Daicel Chiralcel IC column, column temperature 25 °C, solvent heptane:*i*PrOH = 99:1, flow rate 0.7 mL/min): t_R = 17.8 min (major), t_R = 20.4 min (minor).

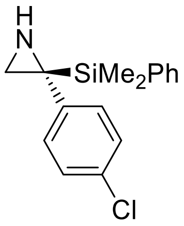

(*S*)-**3xa**
$C_{16}H_{18}ClNSi$
M = 287.86 g/mol

(*S*)-2-(4-chlorophenyl)-2-(dimethyl(phenyl)silyl)aziridine [(*S*)-**3xa**]: Prepared from 3-(4-chlorophenyl)-2*H*-azirine (**1x**, 30.3 mg, 0.200 mmol, 1.00 equiv) according to **GP4** (*Method A*). The residue was purified by flash column chromatography on silica gel using *n*-hexane:ethyl acetate = 10:1 as the eluent to afford (*S*)-**3xa** as colorless oil (51.8 mg, 90% yield). $\mathbf{R_f}$ = 0.10 (*n*-hexane:ethyl acetate = 10:1). **^{1}H NMR** (500 MHz, $CDCl_3$, 298 K) δ 7.51–7.45 (m, 2H), 7.44–7.35 (m, 3H), 7.21 (d, *J* = 8.4 Hz, 2H), 7.16 (d, *J* = 8.4 Hz, 2H), 1.92 (s, 1H), 1.79 (s, 1H), 0.65 (s, 1H), 0.37 (s, 3H), 0.30 (s, 1H) ppm. **^{13}C NMR** (126 MHz, $CDCl_3$, 298 K) δ 142.0, 134.4, 134.1, 131.7, 129.9, 129.8, 128.0, 127.9, 31.3, 29.7, −4.6, −5.2 ppm. **^{1}H/^{29}Si HMQC NMR** (500/99 MHz, $CDCl_3$, 298 K, optimized for *J* = 7 Hz): δ 7.51–7.45/−0.7, 0.37/−0.7, 0.30/−0.7 ppm. **HRMS** (APCI) m/z: $[M+H]^+$ calcd for $C_{16}H_{19}NSi^+$ 288.0970; found 288.0969. **IR** (ATR): ṽ 699, 733, 774, 813, 828, 848, 875, 970, 1008, 1090, 1112, 1247, 1425, 1488, 2949 cm^{-1}. Optical rotation: $[\alpha]_D^{20}$ = −13.1 (*c* 1.0, CH_2Cl_2, 93% *ee*). The enantiomeric excess of (*S*)-**3xa** was determined by HPLC analysis on a chiral stationary phase (Daicel Chiralcel IC column, column temperature 25 °C, solvent heptane:*i*PrOH = 99:1, flow rate 0.7 mL/min): t_R = 20.2 min (major), t_R = 28.0 min (minor).

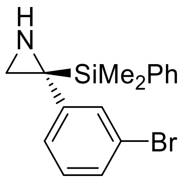

(S)-**3ya**
$C_{16}H_{18}BrNSi$
M = 332.32 g/mol

(S)-2-(3-bromophenyl)-2-(dimethyl(phenyl)silyl)aziridine [(S)-**3ya**]: Prepared from 3-(3-bromophenyl)-2*H*-azirine (**1y**, 39.2 mg, 0.200 mmol, 1.00 equiv) according to **GP4** (*Method A*). The residue was purified by flash column chromatography on silica gel using *n*-hexane:ethyl acetate = 10:1 as the eluent to afford (S)-**3ya** as colorless oil (58.5 mg, 88% yield). **R_f** = 0.10 (*n*-hexane:ethyl acetate = 10:1). **^{1}H NMR** (500 MHz, $CDCl_3$, 298 K) δ 7.56–7.46 (m, 2H), 7.45–7.34 (m, 4H), 7.30 (d, J = 7.8 Hz, 1H), 7.16 (d, J = 7.0 Hz, 1H), 7.10 (t, J = 7.6 Hz, 1H), 1.93 (s, 2H), 0.40 (s, 3H), 0.31 (s, 3H) ppm. **^{13}C NMR** (126 MHz, $CDCl_3$, 298 K) δ 134.3, 134.1, 131.5, 129.9, 129.5, 129.2, 128.0, 127.2, 122.0, 31.2, 29.6, −4.4, −5.2 ppm. **^{1}H/^{29}Si HMQC NMR** (500/99 MHz, $CDCl_3$, 298 K, optimized for J = 7 Hz): δ 7.56–7.46/−0.1, 0.40/−0.1, 0.31/−0.1 ppm. **HRMS** (APCI) m/z: $[M+H+HCl]^+$ calcd for $C_{16}H_{20}BrClNSi^+$ 368.0231; found 368.0231. **IR** (ATR): ṽ 654, 681, 696, 734, 774, 821, 876, 1067, 1109, 1248, 1406, 1426, 1471, 1559, 1589, 2954 cm^{-1}. Optical rotation: $[\alpha]_D^{20}$ = −12.2 (c 1.0, CH_2Cl_2, 95% *ee*). The enantiomeric excess of (S)-**3ya** was determined by HPLC analysis on a chiral stationary phase (Daicel Chiralcel IC column, column temperature 20 °C, solvent heptane:*i*PrOH = 99:1, flow rate 0.8 mL/min): t_R = 16.3 min (major), t_R = 18.2 min (minor).

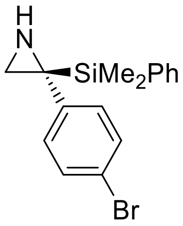

(*S*)-**3za**
$C_{16}H_{18}BrNSi$
M = 332.32 g/mol

(*S*)-2-(4-bromophenyl)-2-(dimethyl(phenyl)silyl)aziridine [(*S*)-**3za**]: Prepared from 3-(4-bromophenyl)-2*H*-azirine (**1z**, 39.2 mg, 0.200 mmol, 1.00 equiv) according to **GP4** (*Method A*). The residue was purified by flash column chromatography on silica gel using *n*-hexane:ethyl acetate = 10:1 as the eluent to afford (*S*)-**3za** as colorless oil (54.5 mg, 82% yield). **R*f*** = 0.10 (*n*-hexane:ethyl acetate = 10:1). **^{1}H NMR** (500 MHz, $CDCl_3$, 298 K) δ 7.51–7.45 (m, 2H), 7.44–7.40 (m, 1H), 7.39–7.32 (m, 4H), 7.10 (d, *J* = 8.2 Hz, 2H), 1.91 (s, 1H), 1.77 (s, 1H), 0.65 (s, 1H), 0.37 (s, 3H), 0.30 (s, 3H) ppm. **^{13}C NMR** (126 MHz, $CDCl_3$, 298 K) δ 142.3, 134.4, 134.1, 131.0, 130.3, 129.8, 127.9, 119.8, 31.6, 29.8, −4.7, −5.2 ppm. **^{1}H/^{29}Si HMQC NMR** (500/99 MHz, $CDCl_3$, 298 K, optimized for *J* = 7 Hz): δ 7.51–7.45/−0.7, 0.37/−0.7, 0.30/−0.7 ppm. **HRMS** (APCI) m/z: $[M+H]^+$ calcd for $C_{16}H_{19}BrNSi^+$ 332.0465; found 332.0466. **IR** (ATR): ṽ 673, 701, 736, 777, 812, 830, 875, 1005, 1066, 1096, 1113, 1134, 1214, 1250, 1388, 1426, 1482, 2958 cm^{-1}. Optical rotation: $[\alpha]_D^{20}$ = −8.95 (*c* 1.0, CH_2Cl_2, 92% *ee*). The enantiomeric excess of (*S*)-**3za** was determined by HPLC analysis on a chiral stationary phase (Daicel Chiralcel IC column, column temperature 25 °C, solvent heptane:*i*PrOH = 99:1, flow rate 0.8 mL/min): t_R = 18.6 min (major), t_R = 28.0 min (minor).

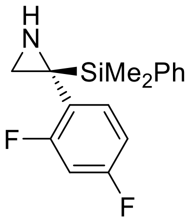

(S)-**3a'a**
$C_{16}H_{17}F_2NSi$
M = 289.40 g/mol

(*S*)-2-(2,4-difluorophenyl)-2-(dimethyl(phenyl)silyl)aziridine [(*S*)-**3a'a**]: Prepared from 3-(2,4-difluorophenyl)-2*H*-azirine (**1a'**, 30.6 mg, 0.200 mmol, 1.00 equiv) according to **GP4** (*Method A*). The residue was purified by flash column chromatography on silica gel using *n*-hexane:ethyl acetate = 10:1 as the eluent to afford (*S*)-**3a'a** as colorless oil (53.2 mg, 92% yield). $\mathbf{R_f}$ = 0.10 (*n*-hexane:ethyl acetate = 10:1). ^{1}**H NMR** (500 MHz, $CDCl_3$, 298 K) δ 7.51–7.46 (m, 2H), 7.45–7.35 (m, 3H), 7.20 (dd, *J* = 15.1, 8.3 Hz, 1H), 6.82–6.67 (m, 2H), 1.91 (s, 1H), 1.81 (s, 1H), 0.72 (s, 1H), 0.38 (d, *J* = 1.4 Hz, 3H), 0.33 (d, *J* = 0.7 Hz, 3H) ppm. ^{13}**C NMR** (126 MHz, $CDCl_3$, 298 K) δ 161.4 (dd, *J* = 247.2, 11.8 Hz), 161.1 (dd, *J* = 248.9, 11.9 Hz), 134.2, 132.0 (dd, *J* = 9.0, 6.3 Hz), 130.0, 128.0, 110.9 (dd, *J* = 21.0, 3.5 Hz), 103.4 (t, *J* = 25.5 Hz), 29.6, 27.0, −5.0, −5.3 ppm. ^{19}**F NMR** (471 MHz, $CDCl_3$, 298 K): δ −110.9, 112.8 ppm. ^{1}**H/**^{29}**Si HMQC NMR** (500/99 MHz, $CDCl_3$, 298 K, optimized for *J* = 7 Hz): δ 7.51–7.46/−0.1, 0.38/−0.1, 0.33/−0.1 ppm. **HRMS** (APCI) m/z: $[M+H]^+$ calcd for $C_{16}H_{18}F_2NSi^+$ 290.1171; found 290.1170. **IR** (ATR): ṽ 656, 699, 734, 775, 821, 879, 960, 1013, 1089, 1133, 1121, 1259, 1417, 1494, 1589, 1607, 2956 cm^{-1}. Optical rotation: $[\alpha]_D^{20}$ = −9.01 (*c* 1.0, CH_2Cl_2, 86% *ee*). The enantiomeric excess of (*S*)-**3a'a** was determined by HPLC analysis on a chiral stationary phase (Daicel Chiralcel IA column, column temperature 25 °C, solvent heptane:*i*PrOH = 99:1, flow rate 0.6 mL/min): t_R = 38.9 min (major), t_R = 32.6 min (minor).

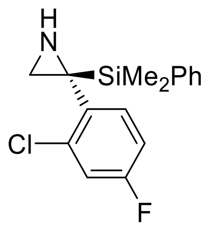

(*S*)-**3b'a**
$C_{16}H_{17}ClFNSi$
M = 305.85 g/mol

(*S*)-2-(2-chloro-4-fluorophenyl)-2-(dimethyl(phenyl)silyl)aziridine [(*S*)-**3b'a**]: Prepared from 3-(2-chloro-4-fluorophenyl)-2*H*-azirine (**1b'**, 33.9 mg, 0.200 mmol, 1.00 equiv) according to **GP4** (*Method A*). The residue was purified by flash column chromatography on silica gel using *n*-hexane:ethyl acetate = 10:1 as the eluent to afford (*S*)-**3b'a** as colorless oil (51.4 mg, 84% yield). $\mathbf{R_f}$ = 0.10 (*n*-hexane:ethyl acetate = 10:1). **^{1}H NMR** (500 MHz, $CDCl_3$, 298 K) δ 7.51–7.46 (m, 1H), 7.45–7.40 (m, 1H), 7.40–7.35 (m, 1H), 7.26 (t, *J* = 7.2 Hz, 1H), 7.04 (dd, *J* = 8.6, 2.6 Hz, 1H), 6.90 (td, *J* = 8.3, 2.6 Hz, 1H), 2.00 (s, 1H), 1.85 (s, 1H), 0.69 (s, 1H), 0.40 (s, 2H), 0.34 (s, 2H) ppm. **^{13}C NMR** (126 MHz, $CDCl_3$, 298 K) δ 160.9 (d, *J* = 248.2 Hz), 137.0, 134.5, 134.3, 134.2, 132.7 (d, *J* = 8.4 Hz), 129.9, 128.0, 116.4 (d, *J* = 24.6 Hz), 113.788 (d, *J* = 21.0 Hz), 31.4, 30.4, −4.3, −4.8 ppm. **^{19}F NMR** (471 MHz, $CDCl_3$, 298 K): δ −114.4 ppm. **^{1}H/^{29}Si HMQC NMR** (500/99 MHz, $CDCl_3$, 298 K, optimized for *J* = 7 Hz): δ 7.51–7.46/−0.4, 2.00/−0.4, 1.85/−0.4, 0.38/−0.4, 0.31/−0.4 ppm. **HRMS** (APCI) m/z: $[M+H]^+$ calcd for $C_{16}H_{18}ClFNSi^+$ 306.0876; found 306.0875. **IR** (ATR): ṽ 654, 699, 734, 774, 817, 903, 1108, 1202, 1251, 1483, 1596, 2956 cm^{-1}. Optical rotation: $[\alpha]_D^{20}$ = −8.47 (*c* 1.0, CH_2Cl_2, 50% *ee*). The enantiomeric excess of (*S*)-**3b'a** was determined by HPLC analysis on a chiral stationary phase (Daicel Chiralcel IA column, column temperature 20 °C, solvent heptane:*i*PrOH = 99:1, flow rate 0.8 mL/min): t_R = 28.3 min (major), t_R = 21.2 min (minor).

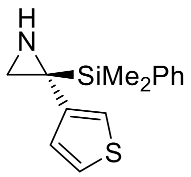

(*S*)-**3c'a**
$C_{14}H_{17}NSSi$
M = 259.44 g/mol

(*S*)-2-(dimethyl(phenyl)silyl)-2-(thiophen-3-yl)aziridine [(*S*)-**3c'a**]: Prepared from 3-3-(thiophen-3-yl)-2*H*-azirine (**1c'**, 24.6 mg, 0.200 mmol, 1.00 equiv) according to **GP4** (*Method A*). The residue was purified by flash column chromatography on silica gel using *n*-hexane:ethyl acetate = 10:1 as the eluent to afford (*S*)-**3c'a** as colorless oil (49.3 mg, 95% yield). **R*f*** = 0.10 (*n*-hexane:ethyl acetate = 10:1). **^{1}H NMR** (500 MHz, $CDCl_3$) δ 7.52 (dd, *J* = 7.7, 1.3 Hz, 2H), 7.45–7.32 (m, 3H), 7.17 (dd, *J* = 4.9, 3.0 Hz, 1H), 6.98 (d, *J* = 2.7 Hz, 1H), 6.91 (d, *J* = 4.9 Hz, 1H), 1.86 (s, 2H), 0.65 (s, 1H), 0.39 (s, 3H), 0.33 (s, 3H) ppm. **^{13}C NMR** (126 MHz, $CDCl_3$) δ 144.2, 135.2, 134.2, 129.6, 127.9, 127.9, 124.9, 121.4, 30.1, 27.8, −4.7, −5.0 ppm. **^{1}H/^{29}Si HMQC NMR** (500/99 MHz, $CDCl_3$, 298 K, optimized for *J* = 7 Hz): δ 7.52/−1.2, 0.39/−0.7, 0.33/−0.7 ppm. **HRMS** (APCI) m/z: $[M+H]^+$ calcd for $C_{14}H_{18}NSSi^+$ 260.0924; found 260.0924. **IR** (ATR): ṽ 698, 734, 773, 812, 857, 922, 1017, 1110, 1247, 1426 cm^{-1}. Optical rotation: $[\alpha]_D^{20}$ = −4.44 (*c* 1.0, CH_2Cl_2, 77% *ee*). The enantiomeric excess of (*S*)-**3c'a** was determined by HPLC analysis on a chiral stationary phase (Daicel Chiralcel IA column, column temperature 20 °C, solvent heptane:*i*PrOH = 98:2, flow rate 1.0 mL/min): t_R = 16.5 min (major), t_R = 12.5 min (minor).

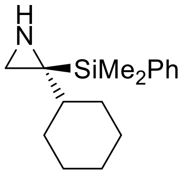

(*S*)-**3d'a**
$C_{16}H_{25}NSi$
M = 259.47 g/mol

(*S*)-2-cyclohexyl-2-(dimethyl(phenyl)silyl)aziridine [(*S*)-**3d'a**]: Prepared from 3-cyclohexyl-2*H*-azirine (**1d'**, 24.6 mg, 0.200 mmol, 1.00 equiv) according to **GP4** (*Method A*). The residue was purified by flash column chromatography on silica gel using *n*-hexane:ethyl acetate = 10:1 as the eluent to afford (*S*)-**3d'a** as colorless oil (25.9 mg, 50% yield). **R*f*** = 0.10 (*n*-hexane:ethyl acetate = 10:1). **M.P**. 37–39 °C. **^{1}H NMR** (500 MHz, $CDCl_3$, 298 K) δ 7.57–7.48 (m, 2H), 7.40–7.31 (m, 3H), 1.89–1.74 (m, 1H), 1.73–1.66 (m, 2H), 1.65–1.56 (m, 2H), 1.47 (s, 2H), 1.18–1.00 (m, 5H), 0.98– 0.85 (m, 1H), 0.40 (s, 3H), 0.38 (s, 3H) ppm. **^{13}C NMR** (126 MHz, $CDCl_3$, 298 K) δ 136.7, 134.1, 129.3, 127.8, 47.4, 31.6, 30.6, 29.8, 28.1, 26.8, 26.7, 26.5, −2.7, −2.9 ppm. **^{1}H/^{29}Si HMQC NMR** (500/99 MHz, $CDCl_3$, 298 K, optimized for *J* = 7 Hz): δ 7.57–7.48/−0.8, 0.40/−0.3, 0.38/−0.8 ppm. **HRMS** (APCI) m/z: $[M+H]^+$ calcd for $C_{16}H_{26}NSi^+$ 260.1829; found 260.1828. **IR** (ATR): ṽ 699, 735, 776, 821, 1112, 1251, 1426, 1447, 1545, 2255, 2852, 2925 cm^{-1}. Optical rotation: $[\alpha]_D^{20}$ = 10.2 (*c* 1.0, CH_2Cl_2, 80% *ee*). The enantiomeric excess of (*S*)-**3d'a** was determined by HPLC analysis on a chiral stationary phase (Daicel Chiralcel IA column, column temperature 20 °C, solvent heptane:*i*PrOH = 98:2, flow rate 0.6 mL/min): t_R = 14.8 min (major), t_R = 13.8 min (minor)

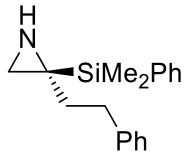

(S)-**3e'a**
$C_{18}H_{23}NSi$
M = 281.47 g/mol

(*S*)-2-(dimethyl(phenyl)silyl)-2-phenethylaziridine [(*S*)-**3e'a**]: Prepared from 3-phenethyl-2*H*-azirine (**1e'**, 29.0 mg, 0.200 mmol, 1.00 equiv) according to **GP4** (*Method A*). The residue was purified by flash column chromatography on silica gel using *n*-hexane:ethyl acetate = 10:1 as the eluent to afford (*S*)-**3e'a** as colorless oil (50.1 mg, 89% yield). $\mathbf{R_f}$ = 0.10 (*n*-hexane:ethyl acetate = 10:1). **^{1}H NMR** (500 MHz, $CDCl_3$, 298 K) δ 7.62–7.56 (m, 2H), 7.46–7.38 (m, 3H), 7.28–7.21 (m, 2H), 7.20–7.14 (m, 1H), 7.11–7.07 (m, 2H), 2.68–2.52 (m, 2H), 1.91–1.77 (m, 1H), 1.77–1.42 (m, 3H), 0.45 (s, 3H), 0.42 (s, 3H). **^{13}C NMR** (126 MHz, $CDCl_3$, 298 K) δ 142.2, 136.0, 134.0, 129.6, 128.2, 128.1, 128.0, 125.7, 39.0, 33.0, 28.6, 25.7, −4.1, −4.4 ppm. **^{1}H/^{29}Si HMQC NMR** (500/99 MHz, $CDCl_3$, 298 K, optimized for J = 7 Hz): δ 7.62–7.56/−0.3, 0.45/−0.3, 0.42/−0.3 ppm. **HRMS** (APCI) m/z: $[M+H]^+$ calcd for $C_{18}H_{24}NSi^+$ 282.1672; found 282.1671. **IR** (ATR): ṽ 660, 697, 733, 773, 819, 1111, 1250, 1426, 1453, 2922 cm^{-1}. Optical rotation: $[\alpha]_D^{20}$ = 2.30 (*c* 1.0, CH_2Cl_2, 87% *ee*). The enantiomeric excess of (*S*)-**3e'a** was determined by HPLC analysis on a chiral stationary phase (Daicel Chiralcel IA column, column temperature 20 °C, solvent heptane:*i*PrOH = 98:2, flow rate 0.6 mL/min): t_R = 18.7 min (major), t_R = 29.5 min (minor).

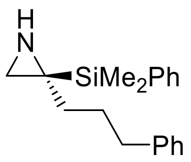

(*S*)-**3f'a**
$C_{19}H_{25}NSi$
M = 295.50 g/mol

(*S*)-2-(dimethyl(phenyl)silyl)-2-(3-phenylpropyl)aziridine [(*S*)-**3f'a**]: Prepared from 3-(3-phenylpropyl)-2*H*-azirine (**1f'**, 31.8 mg, 0.200 mmol, 1.00 equiv) according to **GP4** (*Method A*). The residue was purified by flash column chromatography on silica gel using *n*-hexane:ethyl acetate = 10:1 as the eluent to afford (*S*)-**3f'a** as colorless oil (53.2 mg, 90% yield). $\mathbf{R_f}$ = 0.10 (*n*-hexane:ethyl acetate = 10:1). **^{1}H NMR** (500 MHz, $CDCl_3$, 298 K) δ 7.52 (dd, *J* = 7.6, 1.5 Hz, 2H), 7.43–7.32 (m, 3H), 7.28–7.22 (m, 2H), 7.16 (t, *J* = 7.4 Hz, 1H), 7.11–7.05 (m, 2H), 2.53 (t, *J* = 6.8 Hz, 2H), 1.79–1.49 (m, 4H), 1.40 (s, 2H), 0.37 (s, 3H), 0.34 (s,3H). **^{13}C NMR** (126 MHz, $CDCl_3$, 298 K) δ 142.2, 136.1, 134.0, 129.4, 128.3, 128.2, 127.9, 125.6, 36.8, 36.1, 29.6, 28.6, 25.5, −4.0, −4.4. **^{1}H/^{29}Si HMQC NMR** (500/99 MHz, $CDCl_3$, 298 K, optimized for *J* = 7 Hz): δ 7.52/−0.3, 0.37/−0.3, 0.34/−0.3 ppm. **HRMS** (APCI) m/z: $[M+H]^+$ calcd for $C_{19}H_{26}NSi^+$ 296.1829; found 296.1828. **IR** (ATR): ṽ 660, 697, 733, 772, 813, 1110, 1249, 1426, 1453, 1715, 2926 cm^{-1}. Optical rotation: $[\alpha]_D^{20}$ = −0.870 (*c* 1.0, CH_2Cl_2, 87% *ee*). The enantiomeric excess of (*S*)-**3f'a** was determined by HPLC analysis on a chiral stationary phase (Daicel Chiralcel IA column, column temperature 20 °C, solvent heptane:*i*PrOH = 95:5, flow rate 0.7 mL/min): t_R = 9.83 min (major), t_R = 13.8 min (minor).

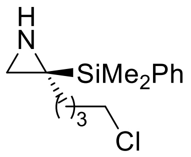

(*S*)-**3g'a**
$C_{14}H_{22}ClNSi$
M = 267.87 g/mol

(S)-2-(4-chlorobutyl)-2-(dimethyl(phenyl)silyl)aziridine [(*S*)-**3g'a**]: Prepared from 3-(4-chlorobutyl)-2*H*-azirine (**1g'**, 26.3 mg, 0.200 mmol, 1.00 equiv) according to **GP4** (*Method A*). The residue was purified by flash column chromatography on silica gel using *n*-hexane:ethyl acetate = 3:1 as the eluent to afford (*S*)-**3g'a** as colorless oil (31.2 mg, 65% yield). **R*f*** = 0.10 (*n*-hexane:ethyl acetate = 3:1). **^{1}H NMR** (500 MHz, $CDCl_3$) δ 7.51 (d, *J* = 7.3 Hz, 2H), 7.42–7.33 (m, 3H), 3.43 (t, *J* = 6.6 Hz, 2H), 1.75–1.64 (m, 2H), 1.62–1.23 (m, 6H), 0.40 (s, 3H), 0.36 (s, 3H) ppm. **^{13}C NMR** (126 MHz, $CDCl_3$) δ 135.8, 134.0, 129.6, 127.9, 44.7, 36.2, 32.7, 28.7, 24.1, −4.2, −4.5 ppm. **^{1}H/^{29}Si HMQC NMR** (500/99 MHz, $CDCl_3$, 298 K, optimized for *J* = 7 Hz): δ 7.51/−0.4, 0.40/−0.4, 0.36/−0.4 ppm. **HRMS** (APCI) m/z: $[M+H]^+$ calcd for $C_{14}H_{23}ClNSi^+$ 268.1283; found 268.1282. **IR** (ATR): ṽ 698, 727, 787, 827, 1025, 1046, 1116, 1251, 1426 cm^{-1}. Optical rotation: $[\alpha]_D^{20}$ = 2.45 (*c* 1.0, CH_2Cl_2, 85% *ee*). The enantiomeric excess of (*S*)-**3g'a** was determined by HPLC analysis on a chiral stationary phase (Daicel Chiralcel IA column, column temperature 20 °C, solvent heptane:*i*PrOH = 98:2, flow rate 0.8 mL/min): t_R = 14.1 min (major), t_R = 15.1 min (minor).

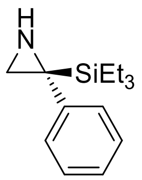

(*S*)-**3ab**
$C_{14}H_{23}NSi$
M = 233.43 g/mol

(*S*)-2-phenyl-2-(triethylsilyl)aziridine [(*S*)-**3ab**]: Prepared from 3-phenyl-2*H*-azirine (**1a**, 24.6 mg, 0.200 mmol, 1.00 equiv) according to **GP4** (*Method A*). The residue was purified by flash column chromatography on silica gel using *n*-hexane:ethyl acetate = 10:1 as the eluent to afford (*S*)-**3ab** as colorless oil (39.2 mg, 84% yield). $\mathbf{R_f}$ = 0.10 (*n*-hexane:ethyl acetate = 10:1). **^{1}H NMR** (500 MHz, $CDCl_3$, 298 K) δ 7.32–7.23 (m, 4H), 7.19–7.12 (m, 1H), 2.03 (s, 1H), 1.83 (s, 1H), 0.95 (t, *J* = 7.9 Hz, 9H), 0.58–0.51 (m, 6H). **^{13}C NMR** (126 MHz, $CDCl_3$, 298 K) δ 144.3, 128.4, 128.0, 125.9, 31.3, 29.5, 7.3, 1.7 ppm. **^{1}H/^{29}Si HMQC NMR** (500/99 MHz, $CDCl_3$, 298 K, optimized for *J* = 7 Hz): δ 0.95/7.3, 0.58–0.51/7.3 ppm. **HRMS** (APCI) m/z: $[M+H]^+$ calcd for $C_{14}H_{24}NSi^+$ 234.1672; found 234.1672. **IR** (ATR): ṽ 698, 752, 801, 870, 1004, 1133, 1214, 1238, 1459, 2874, 2909, 2951 cm^{-1}. Optical rotation: $[\alpha]_D^{20}$ = −15.1 (*c* 1.0, CH_2Cl_2, 64% *ee*). The enantiomeric excess of (*S*)-**3ab** was determined by HPLC analysis on a chiral stationary phase (Daicel Chiralcel IA column, column temperature 37 °C, solvent heptane:*i*PrOH = 99:1, flow rate 0.6 mL/min): t_R = 11.1 min (major), t_R = 14.2 min (minor).

6 Large-Scale Synthesis of (*S*)-3aa

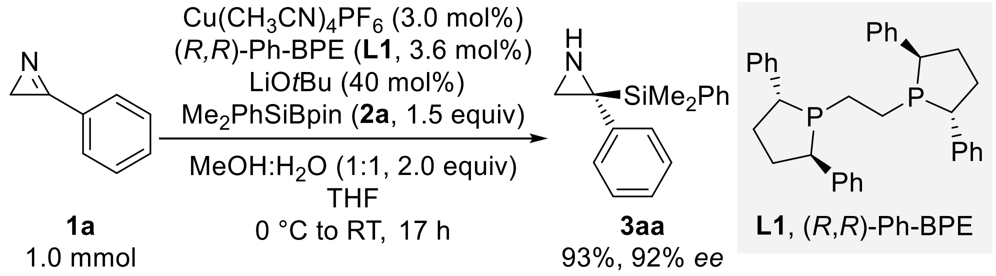

To a flame-dried Schlenk tube equipped with a septum and a magnetic stir bar were added $Cu(CH_3CN)_4PF_6$ (11.2 mg, 0.030 mmol, 3.00 mol%) and (*R*,*R*)-Ph-BPE (18.2 mg, 0.036 mmol, 3.60 mol%). The tube was evacuated under high vacuum and backfilled with nitrogen gas (3 times). THF (10 mL, 1.0 M) was added to the tube, and the resulting mixture was stirred under room temperature for 30 minutes. The mixture was then cooled to 0 °C, and LiO*t*Bu (182 μL, 4.00 mmol, 40.0 mol%) and $Me_2PhSiBpin$ (**2a**, 1.50 mmol, 1.50 equiv) were successively added dropwise. The mixture was stirred under 0 °C for 5 minutes. And then the mixture of methanol/water (1:1, 140 μL, 2.00 equiv) was added, followed by the dropwise addition of 2*H*-azirine (**1a**, 117 mg, 1.00 mmol, 1.00 equiv). The ice bath was subsequently removed, and the reaction was stirred for 17 h at room temperature. After the indicated reaction time, the reaction mixture was filtered through a short plug of silica, and the filter cake was washed with EtOAc (15 mL). The filtrate was concentrated under vacuum. Purification of the residue by flash column chromatography on silica gel with *n*-hexane and ethyl acetate as the eluent to afford the silylated product (*S*)-**3aa** (344 mg, 93%, 92% *ee*).

7 Transformations of (*S*)-3aa

7.1 Rearrangement[S10]

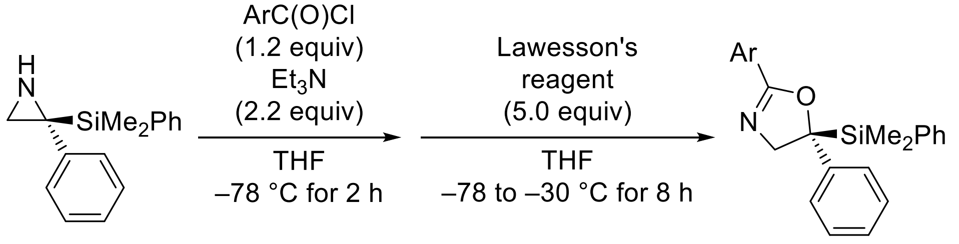

(*S*)-**3aa**: 95% *ee*

Ar = 3,5-dinitrophenyl
(*S*)-**7**: 40%, 94% *ee*

(*S*)-**3aa** (50.7 mg, 0.200 mmol, 1.00 equiv) and triethylamine (61.0 μL, 0.440 mmol, 2.20 equiv) was added to THF (1.2 mL) at −78 °C, and then 3,5-dinitrobenzoyl chloride (55.3 mg, 0.240 mmol, 1.20 equiv) was added to the reaction mixture. After stirred at −78 °C for 2 h, Lawesson's reagent (404 mg, 1.00 mmol, 5.00 equiv) was added to the reaction mixture and stirred at −78 °C for 1 h, and then the reaction temperature was warmed to −30 °C. After stirring for 7 h, sat. $NaHCO_3$ aq. was added, and aqueous layer was extracted with CH_2Cl_2 for three times. The combined organic layer was dried over Na_2SO_4 and concentrated under reduced pressure to give the crude product, which was purified by silica gel column chromatography (*n*-hexane:ethyl acetate = 10:1, R_f = 0.10) to give compound (*S*)-**7** as colorless oil (38.0 mg, 40% yield, 94% *ee*).

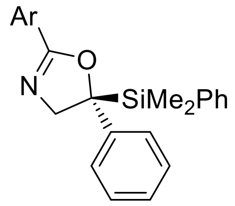

Ar = 3,5-dinitrophenyl
(*S*)-**7**
$C_{23}H_{21}N_3O_5Si$
M = 447.52 g/mol

(*S*)-5-(dimethyl(phenyl)silyl)-2-(3,5-dinitrophenyl)-5-phenyl-4,5-dihydrooxazole [(*S*)-**7**]: **^{1}H NMR** (500 MHz, $CDCl_3$, 298 K) δ 9.14 (t, *J* = 2.1 Hz, 1H), 9.07 (d, *J* = 2.1 Hz, 2H), 7.41–7.36 (m, 3H), 7.33–7.28 (m, 4H), 7.25–7.21 (m, 1H), 7.09–7.04 (m, 1H), 4.55 (d, *J* = 14.8 Hz, 1H), 4.32 (d, *J* = 14.8 Hz, 1H), 0.39 (d, *J* = 1.4 Hz, 3H) ppm. **^{13}C NMR** (126 MHz, $CDCl_3$, 298 K) δ 148.6, 143.4, 134.5, 133.2, 131.8, 130.1, 128.2,

127.9, 127.9, 126.7, 123.9, 120.7, 86.1, 65.2, −6.3, −6.5 ppm. **^{29}Si DEPT NMR** (99 MHz, $CDCl_3$, 298 K): δ −1.5 ppm. **HRMS** (APCI) m/z: $[M+H]^+$ calcd for $C_{23}H_{22}N_3O_5Si^+$ 448.1323; found 448.1322. **IR** (ATR): ṽ 654, 697, 730, 782, 807, 832, 916, 932, 1074, 1111, 1147, 1252, 1337, 1541, 1653, 2920 cm^{-1}. Optical rotation: $[\alpha]_D^{20}$ = −73.44 (*c* 1.0, CH_2Cl_2, 94% *ee*). The enantiomeric excess of (*S*)-**7** was determined by HPLC analysis on a chiral stationary phase (Daicel Chiralcel AD-H column, column temperature 20 °C, solvent heptane:*i*PrOH = 97:3, flow rate 0.8 mL/min): t_R = 21.1 min (major), t_R = 12.7 min (minor).

7.2 Hydrogenation[S11]

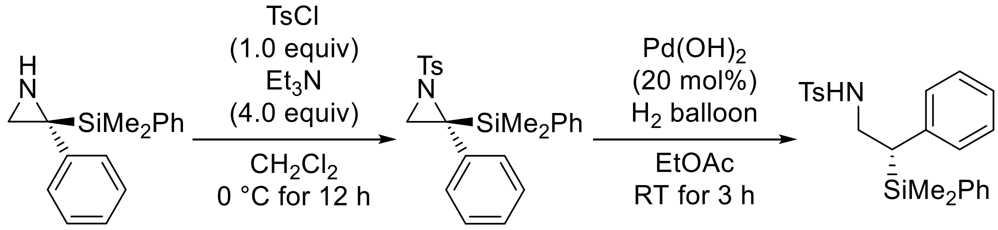

(*S*)-**3aa**: 95% *ee* (*S*)-**8**: 85%, 95% *ee* (*S*)-**9**: 95%, 90% *ee*

To a solution of (*S*)-**3aa** (50.7 mg, 0.200 mmol, 1.00 equiv) in dichloromethane was added Et_3N (0.111 mL, 0.800 mmol, 4.00 equiv) was followed by the sulfonyl chloride (38.1 mg, 0.200 mmol,1.00 equiv). The reaction mixture was left overnight at 4 °C. After removal of solvent under reduced pressure, the crude was purified by silica gel column chromatography (*n*-hexane:ethyl acetate = 15:1, R_f = 0.30) to afford desired product (*S*)-**8** as a white solid.

To a solution of protected aziridine (40.7 mmol, 0.100 mmol, 1.00 equiv) in 2 mL of EtOAc was added $Pd(OH)_2$ (2.81 mg, 0.0200 mmol, 20.0 mol%). The mixture was connected via cannula to a balloon filled with hydrogen at room temperature and stirred for 3 h. The reaction mixture was filtered on a Celite® pad and concentrated in vacuo. The crude was purified by silica gel column chromatography (*n*-hexane:ethyl acetate = 5:1, R_f = 0.20) to afford desired product (*S*)-**9** as a white solid.

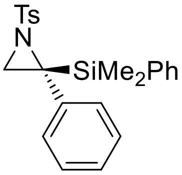

(*S*)-**8**
$C_{23}H_{25}NO_2SSi$
M = 407.60 g/mol

(*S*)-2-(dimethyl(phenyl)silyl)-2-phenyl-1-tosylaziridine [(*S*)-**8**]: **M.P.** 84–86 °C. **^{1}H NMR** (500 MHz, $CDCl_3$, 298 K) δ 7.63 (d, *J* = 8.3 Hz, 2H), 7.36–7.30 (m, 2H), 7.29–7.24 (m, 1H), 7.20 (t, *J* = 7.2 Hz, 2H), 7.15 (d, *J* = 8.0 Hz, 2H), 7.13–7.09 (m, 3H), 7.09–7.04 (m, 2H), 2.79 (s, 1H), 2.47 (s, 1H), 2.31 (s, 3H), 0.35 (s, 3H), 0.26 (s, 3H) ppm. **^{13}C NMR** (126 MHz, $CDCl_3$, 298 K) δ 143.6, 137.3, 137.1, 135.1, 134.4, 129.5, 129.3, 127.7, 127.7, 127.5, 127.2, 49.7, 37.0, 21.5, −4.5 ppm. **^{29}Si DEPT NMR** (99

MHz, $CDCl_3$, 298 K): δ −1.4 ppm. **HRMS** (APCI) m/z: $[M+H]^+$ calcd for $C_{23}H_{26}NO_2SSi^+$ 124.1448; found 408.1450. **IR** (ATR): ṽ 666, 693, 739, 783, 822, 932, 1057, 1085, 1106, 1154, 1204, 1254, 1302, 1319, 1426, 2961 cm^{-1}. Optical rotation: $[\alpha]_D^{20}$ = 82.13 (*c* 1.0, CH_2Cl_2, 95% *ee*). The enantiomeric excess of (*S*)-**8** was determined by HPLC analysis on a chiral stationary phase (Daicel Chiralcel IA column, column temperature 20 °C, solvent heptane:*i*PrOH = 95:5, flow rate 0.8 mL/min): t_R = 18.8 min (major), t_R = 13.4 min (minor).

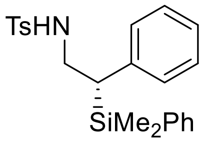

(*S*)-**9**
$C_{23}H_{27}NO_2SSi$
M = 409.62 g/mol

(*S*)-N-(2-(dimethyl(phenyl)silyl)-2-phenylethyl)-4-methylbenzenesulfonamide [(*S*)-**9**]: **M.P**. 71–73 °C. **^{1}H NMR** (500 MHz, $CDCl_3$, 298 K) δ 7.41 (d, *J* = 8.3 Hz, 2H), 7.26–7.20 (m, 1H), 7.19–7.12 (m, 4H), 7.12–7.08 (m, 2H), 7.03–6.93 (m, 3H), 6.61–6.49 (m, 2H), 4.04 (dd, *J* = 8.2, 2.2 Hz, 1H), 3.26 (ddd, *J* = 12.8, 8.4, 4.2 Hz, 1H), 3.12 (td, *J* = 12.8, 2.8 Hz, 1H), 2.33–2.27 (m, 3H), 2.19 (dd, *J* = 12.5, 4.2 Hz, 1H), 0.04 (s, 3H), 0.00 (s, 3H) ppm. **^{13}C NMR** (126 MHz, $CDCl_3$, 298 K) δ 143.2, 138.6, 136.8, 135.8, 133.9, 129.5, 129.5, 128.6, 127.8, 127.8, 127.1, 125.7, 43.4, 37.1, 21.5, −4.1, −5.4 ppm. **^{29}Si DEPT NMR** (99 MHz, $CDCl_3$, 298 K): δ −2.9 ppm. **HRMS** (APCI) m/z: $[M+H]^+$ calcd for $C_{23}H_{28}NO_2SSi^+$ 410.1604; found 410.1607. **IR** (ATR): ṽ 665, 693, 737, 811, 1049, 1078, 1161, 1248, 1302, 1314, 1424, 3241 cm^{-1}. Optical rotation: $[\alpha]_D^{20}$ = −6.42 (*c* 1.0, CH_2Cl_2, 90% *ee*). The enantiomeric excess of (*S*)-**9** was determined by HPLC analysis on a chiral stationary phase (Daicel Chiralcel OD-H column, column temperature 20 °C, solvent heptane:*i*PrOH = 97:3, flow rate 0.6 mL/min): t_R = 44.1 min (major), t_R = 41.1 min (minor).

8 Determination of the Absolute Configuration

(*S*)-2-(benzo[*d*][1,3]dioxol-5-yl)-2-(dimethyl(phenyl)silyl)aziridine [(*S*)-**3na**]:

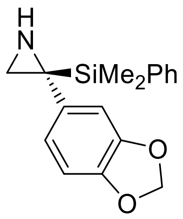

Chemical Formula: $C_{17}H_{19}NO_2Si$
Exact Mass: 297.12

Crystal (*S*)-**3na** was obtained through recrystallization in the solution of EtOAc at −20 °C. The absolute configuration of **3na** was confirmed unambiguously by X-ray diffraction analysis, and other compounds were assigned by analogy. CCDC 2208083 contains the supplementary crystallographic data for this compound.

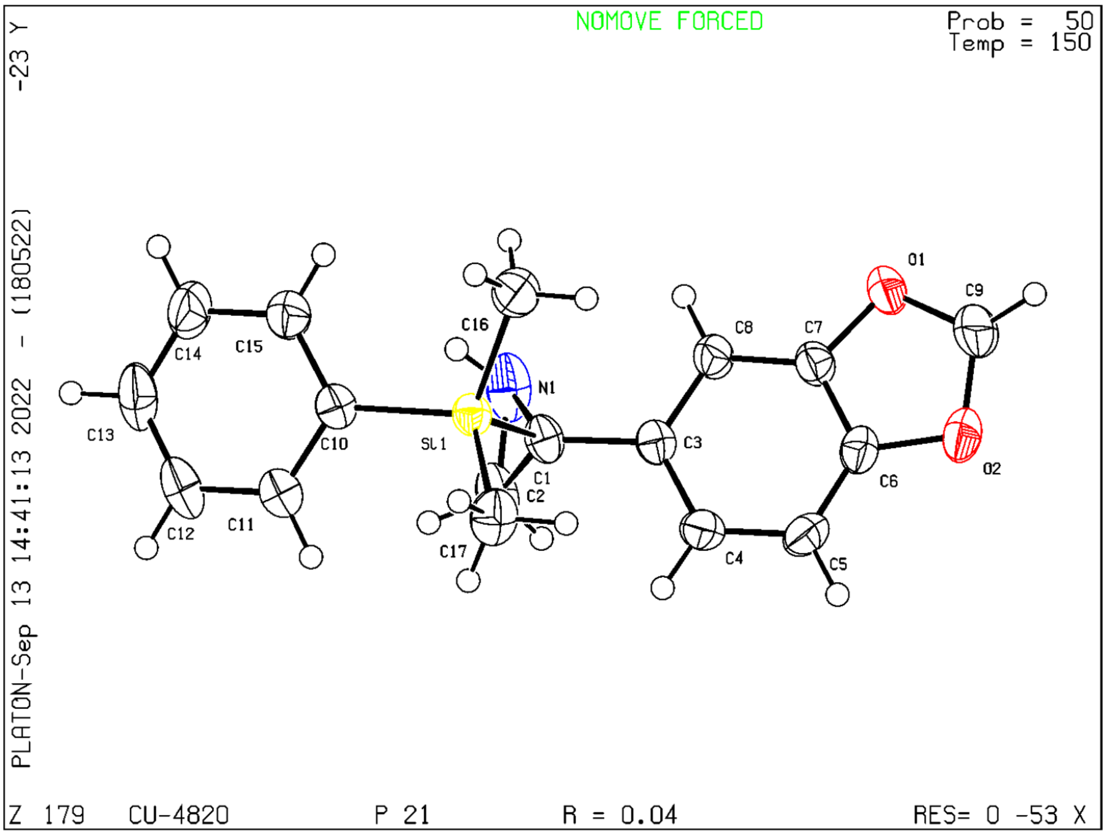

Figure S1. Molecular structure of (*S*)-**3na**.

Empirical formula	C17 H18 N O2 Si	
Formula weight	296.41	
Temperature	150.01(10) K	
Wavelength	1.54184 Å	
Crystal system	Monoclinic	
Space group	$P2_1$ (No. 4)	
Unit cell dimensions	a = 9.6984(4) Å	a= 90°.
	b = 7.8927(3) Å	b= 96.109(4)°.
	c = 10.2853(5) Å	g = 90°.
Volume	782.83(6) $Å^3$	
Z	2	
Density (calculated)	1.257 Mg/m^3	
Absorption coefficient	1.352 mm^{-1}	
F(000)	314	
Crystal size	0.250 x 0.189 x 0.186 mm^3	
Theta range for data collection	4.323 to 67.420°.	
Index ranges	-11<=h<=9, -8<=k<=9, -12<=l<=12	
Reflections collected	4852	
Independent reflections	2589 [R(int) = 0.0276]	
Completeness to theta = 67.420°	100.0 %	
Absorption correction	Semi-empirical from equivalents	
Max. and min. transmission	1.00000 and 0.14592	
Refinement method	Full-matrix least-squares on F^2	
Data / restraints / parameters	2589 / 1 / 196	
Goodness-of-fit on F^2	1.068	
Final R indices [I>2sigma(I)]	R1 = 0.0389, wR2 = 0.1067	
R indices (all data)	R1 = 0.0400, wR2 = 0.1078	
Absolute structure parameter	-0.02(4)	
Extinction coefficient	n/a	
Largest diff. peak and hole	0.425 and -0.214 e.$Å^{-3}$	

Table S8. Atomic coordinates (x 10^4) and equivalent isotropic displacement parameters ($Å^2$x 10^3) for (*S*)-**3na**. U(eq) is defined as one third of the trace of the orthogonalized U^{ij} tensor.

	x	y	z	U(eq)
Si(1)	6850(1)	9701(1)	3200(1)	30(1)
O(1)	2147(2)	4760(5)	2179(3)	51(1)
O(2)	775(2)	6585(4)	851(3)	48(1)
N(1)	7380(3)	6618(5)	1827(4)	51(1)
C(1)	6441(3)	8114(4)	1847(3)	34(1)
C(2)	7392(4)	7899(6)	813(4)	49(1)
C(3)	4938(3)	7711(4)	1512(3)	31(1)
C(4)	4106(4)	8788(5)	695(3)	39(1)
C(5)	2679(4)	8514(5)	416(4)	44(1)
C(6)	2143(3)	7150(5)	980(3)	35(1)
C(7)	2948(3)	6063(5)	1770(3)	34(1)
C(8)	4352(3)	6299(5)	2061(3)	35(1)
C(9)	755(4)	5267(7)	1775(5)	66(2)
C(10)	8797(3)	9845(5)	3472(3)	33(1)
C(11)	9533(4)	10764(5)	2616(4)	39(1)
C(12)	10979(4)	10777(5)	2747(4)	49(1)
C(13)	11698(4)	9892(7)	3736(5)	58(1)
C(14)	11003(4)	8997(6)	4605(5)	62(1)
C(15)	9550(4)	8982(6)	4478(4)	48(1)
C(16)	6145(4)	8854(7)	4681(4)	54(1)
C(17)	6045(4)	11791(5)	2742(4)	46(1)

Table S9. Bond lengths [Å] and angles [°] for (*S*)-**3na**.

Si(1)-C(16)	1.859(4)
Si(1)-C(17)	1.864(4)
Si(1)-C(10)	1.882(3)
Si(1)-C(1)	1.883(3)
O(1)-C(7)	1.381(5)
O(1)-C(9)	1.427(5)
O(2)-C(6)	1.393(4)
O(2)-C(9)	1.411(5)
N(1)-C(2)	1.454(6)
N(1)-C(1)	1.493(5)
C(1)-C(2)	1.490(5)
C(1)-C(3)	1.495(4)
C(3)-C(4)	1.391(5)
C(3)-C(8)	1.397(5)
C(4)-C(5)	1.400(5)
C(5)-C(6)	1.353(5)
C(6)-C(7)	1.367(5)
C(7)-C(8)	1.376(5)
C(10)-C(15)	1.381(5)
C(10)-C(11)	1.395(5)
C(11)-C(12)	1.395(5)
C(12)-C(13)	1.363(7)
C(13)-C(14)	1.372(7)
C(14)-C(15)	1.400(5)
C(16)-Si(1)-C(17)	110.2(2)
C(16)-Si(1)-C(10)	110.50(16)
C(17)-Si(1)-C(10)	111.77(18)
C(16)-Si(1)-C(1)	107.4(2)
C(17)-Si(1)-C(1)	110.62(17)
C(10)-Si(1)-C(1)	106.22(14)
C(7)-O(1)-C(9)	104.3(3)
C(6)-O(2)-C(9)	104.7(3)
C(2)-N(1)-C(1)	60.7(3)
C(2)-C(1)-N(1)	58.3(3)
C(2)-C(1)-C(3)	118.3(3)

N(1)-C(1)-C(3)	114.2(3)
C(2)-C(1)-Si(1)	120.3(2)
N(1)-C(1)-Si(1)	117.1(3)
C(3)-C(1)-Si(1)	115.8(2)
N(1)-C(2)-C(1)	60.9(2)
C(4)-C(3)-C(8)	119.8(3)
C(4)-C(3)-C(1)	119.8(3)
C(8)-C(3)-C(1)	120.3(3)
C(3)-C(4)-C(5)	121.8(3)
C(6)-C(5)-C(4)	116.9(3)
C(5)-C(6)-C(7)	122.2(3)
C(5)-C(6)-O(2)	128.3(3)
C(7)-C(6)-O(2)	109.5(3)
C(6)-C(7)-C(8)	122.3(3)
C(6)-C(7)-O(1)	110.2(3)
C(8)-C(7)-O(1)	127.6(3)
C(7)-C(8)-C(3)	117.1(3)
O(2)-C(9)-O(1)	108.9(3)
C(15)-C(10)-C(11)	117.6(3)
C(15)-C(10)-Si(1)	121.6(3)
C(11)-C(10)-Si(1)	120.7(3)
C(10)-C(11)-C(12)	121.3(4)
C(13)-C(12)-C(11)	119.8(4)
C(12)-C(13)-C(14)	120.2(3)
C(13)-C(14)-C(15)	120.2(4)
C(10)-C(15)-C(14)	120.9(4)

Symmetry transformations used to generate equivalent atoms:

Table S10. Anisotropic displacement parameters ($Å^2$x 10^3) for (*S*)-**3na**. The anisotropic displacement factor exponent takes the form: $-2\pi^2[h^2 a^{*2}U^{11} + ... + 2 h k a^* b^* U^{12}]$

	U^{11}	U^{22}	U^{33}	U^{23}	U^{13}	U^{12}
Si(1)	26(1)	33(1)	30(1)	-2(1)	7(1)	0(1)
O(1)	30(1)	53(2)	69(2)	20(2)	1(1)	-9(1)
O(2)	28(1)	56(2)	58(2)	2(2)	-6(1)	-2(1)
N(1)	32(2)	43(2)	80(2)	-22(2)	11(2)	4(1)
C(1)	28(2)	32(2)	41(2)	-6(1)	9(1)	-2(1)
C(2)	37(2)	60(3)	53(2)	-26(2)	18(2)	-12(2)
C(3)	29(2)	32(2)	33(2)	-5(1)	7(1)	0(1)
C(4)	45(2)	38(2)	34(2)	6(2)	0(1)	-9(2)
C(5)	42(2)	44(2)	43(2)	10(2)	-11(2)	1(2)
C(6)	29(2)	42(2)	34(2)	-6(1)	-1(1)	-3(1)
C(7)	29(2)	36(2)	39(2)	1(1)	6(1)	-3(1)
C(8)	27(2)	35(2)	43(2)	4(2)	4(1)	2(1)
C(9)	27(2)	87(4)	84(3)	34(3)	8(2)	-3(2)
C(10)	31(1)	31(2)	37(1)	-3(2)	6(1)	-1(2)
C(11)	36(2)	39(2)	44(2)	-2(2)	8(1)	-4(1)
C(12)	36(2)	44(2)	71(3)	-11(2)	20(2)	-11(2)
C(13)	27(2)	44(2)	100(3)	-7(3)	1(2)	-3(2)
C(14)	35(2)	54(2)	93(3)	18(2)	-13(2)	1(2)
C(15)	36(2)	48(2)	59(2)	12(2)	0(2)	-2(2)
C(16)	37(2)	88(3)	38(2)	11(2)	11(1)	-3(2)
C(17)	39(2)	39(2)	61(2)	-8(2)	3(2)	6(2)

(*S*)-4-(2-(dimethyl(phenyl)silyl)aziridin-2-yl)phenyl acetate [(*S*)-**3pa**]:

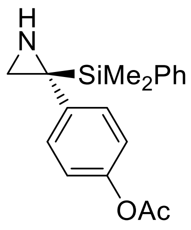

(*S*)-**3pa**
$C_{18}H_{21}NO_2Si$
M = 311.13 g/mol

Crystal (*S*)-**3pa** was obtained through recrystallization in the solution of EtOAc at −20 °C. The absolute configuration of **3pa** was confirmed unambiguously by X-ray diffraction analysis, and other compounds were assigned by analogy. CCDC 2208082 contains the supplementary crystallographic data for this compound.

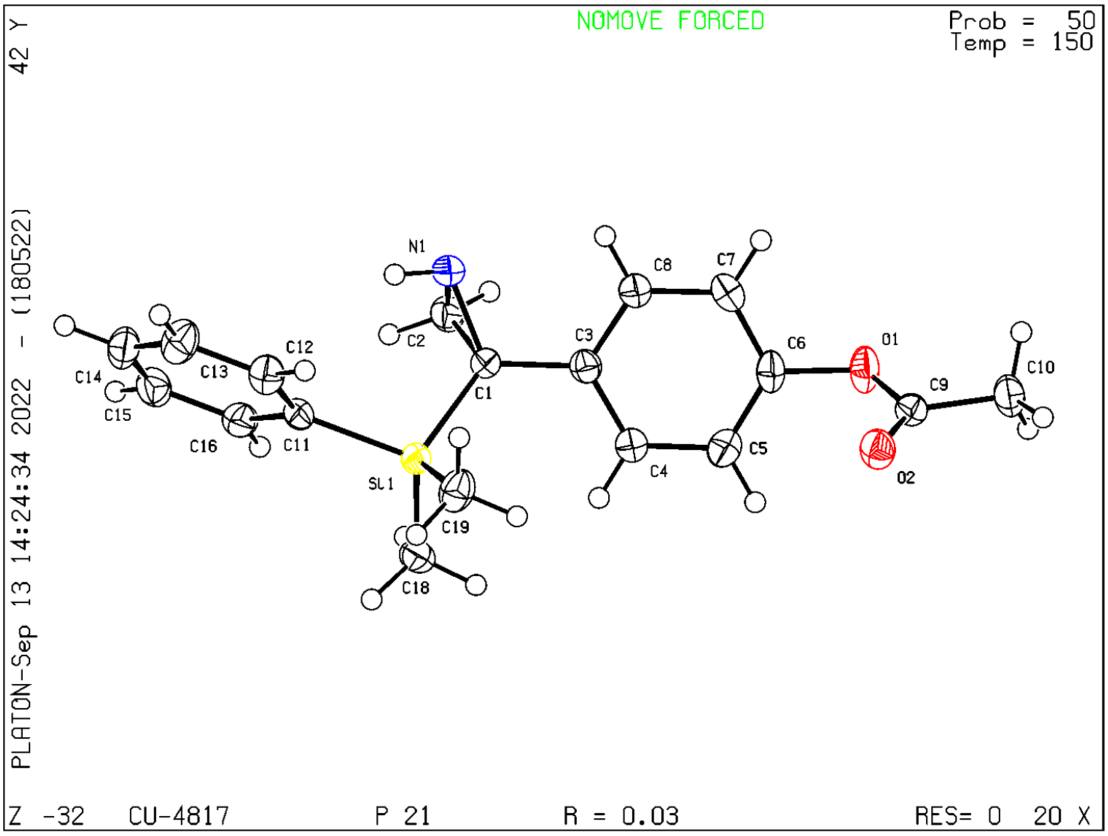

Figure S2. Molecular structure of (*S*)-**3pa**.

Empirical formula	C18 H21 N O2 Si
Formula weight	311.45

Temperature	150.01(10) K	
Wavelength	1.54184 Å	
Crystal system	Monoclinic	
Space group	$P2_1$ (No. 4)	
Unit cell dimensions	a = 6.5553(2) Å	a= 90°.
	b = 13.7101(3) Å	b=
100.977(2)°.		
	c = 9.4274(2) Å	g = 90°.
Volume	831.77(4) $Å^3$	
Z	2	
Density (calculated)	1.244 Mg/m^3	
Absorption coefficient	1.294 mm^{-1}	
F(000)	332	
Crystal size	0.210 x 0.187 x 0.167 mm^3	
Theta range for data collection	4.778 to 67.470°.	
Index ranges	-7<=h<=7, -15<=k<=16, -8<=l<=11	
Reflections collected	5139	
Independent reflections	2744 [R(int) = 0.0105]	
Completeness to theta = 67.470°	99.9 %	
Absorption correction	Semi-empirical from equivalents	
Max. and min. transmission	1.00000 and 0.32308	
Refinement method	Full-matrix least-squares on F^2	
Data / restraints / parameters	2744 / 1 / 206	
Goodness-of-fit on F^2	1.058	
Final R indices [I>2sigma(I)]	R1 = 0.0340, wR2 = 0.0906	
R indices (all data)	R1 = 0.0344, wR2 = 0.0916	
Absolute structure parameter	0.03(3)	
Extinction coefficient	n/a	
Largest diff. peak and hole	0.155 and -0.249 $e.Å^{-3}$	

Table S11. Atomic coordinates (x 10^4) and equivalent isotropic displacement parameters ($Å^2$x 10^3) for (*S*)-**3pa**. U(eq) is defined as one third of the trace of the orthogonalized U^{ij} tensor.

	x	y	z	U(eq)
Si(1)	8660(1)	4708(1)	8917(1)	23(1)
N(1)	10008(4)	6583(2)	7994(3)	34(1)
O(1)	13767(3)	4208(2)	3223(2)	35(1)
O(2)	15548(3)	3017(2)	4562(2)	33(1)
C(1)	9442(4)	5558(2)	7527(3)	23(1)
C(2)	8009(4)	6393(2)	7052(3)	33(1)
C(3)	10652(4)	5175(2)	6441(3)	24(1)
C(4)	10156(5)	4277(2)	5774(3)	32(1)
C(5)	11202(5)	3932(2)	4721(3)	35(1)
C(6)	12769(4)	4490(2)	4357(3)	28(1)
C(7)	13303(4)	5378(2)	4991(3)	31(1)
C(8)	12246(4)	5715(2)	6040(3)	28(1)
C(9)	15163(4)	3467(2)	3461(3)	26(1)
C(10)	16102(5)	3321(2)	2143(3)	35(1)
C(11)	7868(4)	5525(2)	10325(3)	25(1)
C(12)	9297(4)	5813(2)	11552(3)	31(1)
C(13)	8717(5)	6419(3)	12591(3)	38(1)
C(14)	6683(5)	6745(2)	12424(3)	37(1)
C(15)	5243(5)	6465(2)	11223(3)	36(1)
C(16)	5822(4)	5862(2)	10189(3)	30(1)
C(18)	6400(5)	3931(2)	8110(3)	32(1)
C(19)	10917(5)	3940(2)	9741(3)	35(1)

Table S12. Bond lengths [Å] and angles [°] for (*S*)-**3pa**.

Si(1)-C(19)	1.862(3)
Si(1)-C(18)	1.864(3)
Si(1)-C(11)	1.884(3)
Si(1)-C(1)	1.896(3)
N(1)-C(2)	1.459(4)
N(1)-C(1)	1.498(4)
O(1)-C(9)	1.357(3)
O(1)-C(6)	1.410(3)
O(2)-C(9)	1.192(3)
C(1)-C(2)	1.494(4)
C(1)-C(3)	1.504(4)
C(3)-C(8)	1.390(4)
C(3)-C(4)	1.392(4)
C(4)-C(5)	1.393(4)
C(5)-C(6)	1.376(4)
C(6)-C(7)	1.372(4)
C(7)-C(8)	1.390(4)
C(9)-C(10)	1.502(3)
C(11)-C(12)	1.400(4)
C(11)-C(16)	1.401(4)
C(12)-C(13)	1.391(4)
C(13)-C(14)	1.387(4)
C(14)-C(15)	1.383(5)
C(15)-C(16)	1.385(4)
C(19)-Si(1)-C(18)	110.71(15)
C(19)-Si(1)-C(11)	110.25(12)
C(18)-Si(1)-C(11)	108.83(12)
C(19)-Si(1)-C(1)	109.78(13)
C(18)-Si(1)-C(1)	111.60(11)
C(11)-Si(1)-C(1)	105.56(12)
C(2)-N(1)-C(1)	60.69(18)
C(9)-O(1)-C(6)	118.4(2)
C(2)-C(1)-N(1)	58.39(19)
C(2)-C(1)-C(3)	116.5(2)
N(1)-C(1)-C(3)	113.2(2)

C(2)-C(1)-Si(1)	116.15(17)
N(1)-C(1)-Si(1)	117.27(17)
C(3)-C(1)-Si(1)	120.2(2)
N(1)-C(2)-C(1)	60.92(18)
C(8)-C(3)-C(4)	118.1(2)
C(8)-C(3)-C(1)	121.3(3)
C(4)-C(3)-C(1)	120.5(2)
C(3)-C(4)-C(5)	121.2(3)
C(6)-C(5)-C(4)	118.7(3)
C(7)-C(6)-C(5)	121.7(2)
C(7)-C(6)-O(1)	117.2(3)
C(5)-C(6)-O(1)	120.8(3)
C(6)-C(7)-C(8)	119.0(3)
C(3)-C(8)-C(7)	121.2(3)
O(2)-C(9)-O(1)	123.8(2)
O(2)-C(9)-C(10)	126.8(3)
O(1)-C(9)-C(10)	109.4(2)
C(12)-C(11)-C(16)	117.5(2)
C(12)-C(11)-Si(1)	121.2(2)
C(16)-C(11)-Si(1)	121.3(2)
C(13)-C(12)-C(11)	121.3(3)
C(14)-C(13)-C(12)	119.9(3)
C(15)-C(14)-C(13)	119.7(3)
C(14)-C(15)-C(16)	120.4(3)
C(15)-C(16)-C(11)	121.2(3)

Symmetry transformations used to generate equivalent atoms:

Table S13. Anisotropic displacement parameters ($Å^2$x 10^3) for (*S*)-**3pa**. The anisotropic displacement factor exponent takes the form: $-2\pi^2[h^2 a^{*2}U^{11} + ... + 2 h k a^* b^* U^{12}]$

	U^{11}	U^{22}	U^{33}	U^{23}	U^{13}	U^{12}
Si(1)	26(1)	22(1)	22(1)	1(1)	7(1)	1(1)
N(1)	44(1)	26(1)	36(1)	-7(1)	20(1)	-10(1)
O(1)	44(1)	41(1)	24(1)	6(1)	14(1)	17(1)
O(2)	40(1)	33(1)	26(1)	5(1)	9(1)	7(1)
C(1)	24(1)	22(1)	24(1)	-2(1)	7(1)	-2(1)
C(2)	41(2)	26(1)	36(1)	5(1)	18(1)	5(1)
C(3)	26(1)	26(1)	22(1)	2(1)	4(1)	1(1)
C(4)	37(1)	30(1)	33(1)	-5(1)	15(1)	-6(1)
C(5)	42(2)	31(2)	33(1)	-6(1)	12(1)	0(1)
C(6)	30(1)	36(2)	20(1)	2(1)	7(1)	11(1)
C(7)	27(1)	37(2)	29(1)	4(1)	11(1)	-1(1)
C(8)	30(1)	29(2)	28(1)	-2(1)	9(1)	-2(1)
C(9)	25(1)	26(1)	26(1)	-1(1)	6(1)	-2(1)
C(10)	41(2)	36(2)	33(1)	6(1)	19(1)	10(1)
C(11)	30(1)	24(1)	23(1)	3(1)	10(1)	-1(1)
C(12)	33(1)	33(2)	26(1)	3(1)	4(1)	6(1)
C(13)	50(2)	40(2)	23(1)	-2(1)	3(1)	7(1)
C(14)	51(2)	33(2)	30(1)	1(1)	17(1)	10(1)
C(15)	34(1)	34(2)	43(2)	4(1)	18(1)	6(1)
C(16)	29(1)	28(1)	34(1)	0(1)	8(1)	-1(1)
C(18)	40(1)	31(1)	28(1)	-1(1)	11(1)	-10(1)
C(19)	40(2)	34(2)	32(1)	0(1)	6(1)	12(1)

9 HPLC Traces

(*S*)-2-(dimethyl(phenyl)silyl)-2-phenylaziridine [(*S*)-3aa]

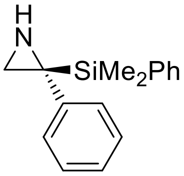

(*S*)-**3aa**

rac-**3aa**

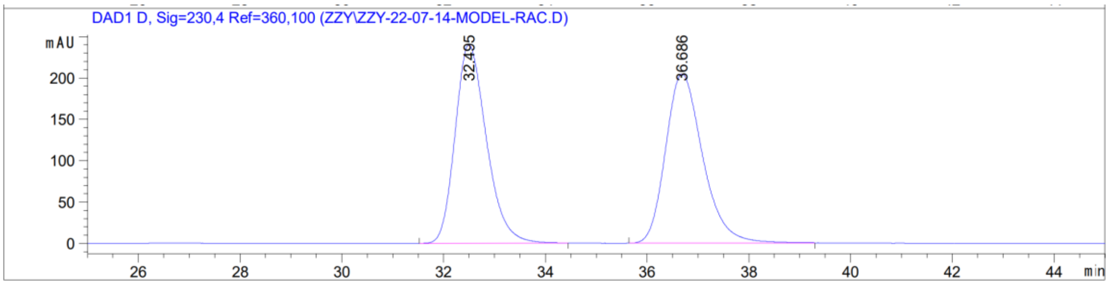

Peak #	RetTime [min]	Type	Width [min]	Area [mAU*s]	Height [mAU]	Area %
1	32.495	BB	0.6556	1.02822e4	240.12541	49.8092
2	36.686	BB	0.7749	1.03609e4	204.55884	50.1908

(*S*)-**3aa** (95% *ee*)

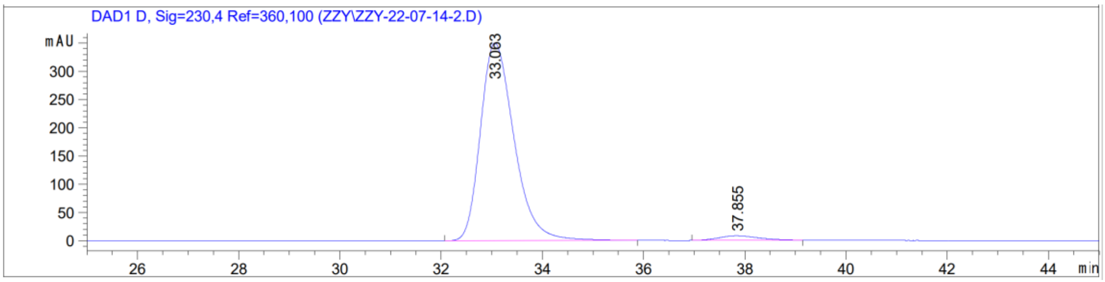

Peak #	RetTime [min]	Type	Width [min]	Area [mAU*s]	Height [mAU]	Area %
1	33.063	BB	0.7063	1.61603e4	350.24738	97.3626
2	37.855	BB	0.6439	437.75311	8.03077	2.6374

(*S*)-2-(dimethyl(phenyl)silyl)-2-(*m*-tolyl)aziridine [(*S*)-**3ba**]

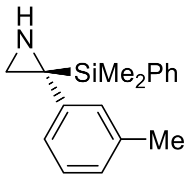

(*S*)-**3ba**

rac-**3ba**

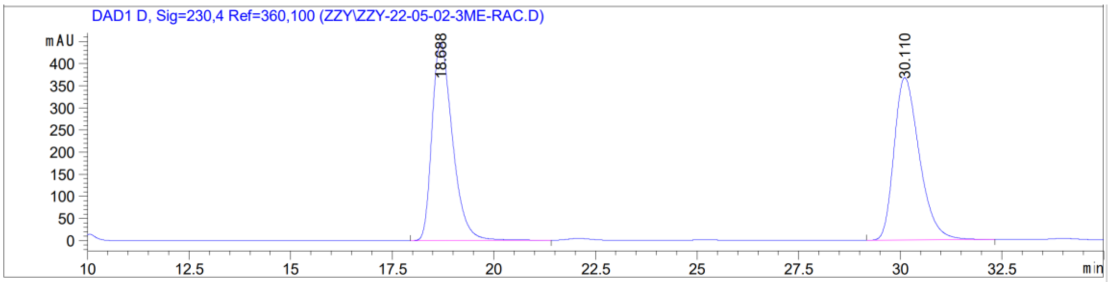

Peak #	RetTime [min]	Type	Width [min]	Area [mAU*s]	Height [mAU]	Area %
1	18.688	BB	0.5319	1.56386e4	447.25427	49.5797
2	30.110	BB	0.6595	1.59037e4	367.01367	50.4203

(*S*)-**3ba** (93% *ee*)

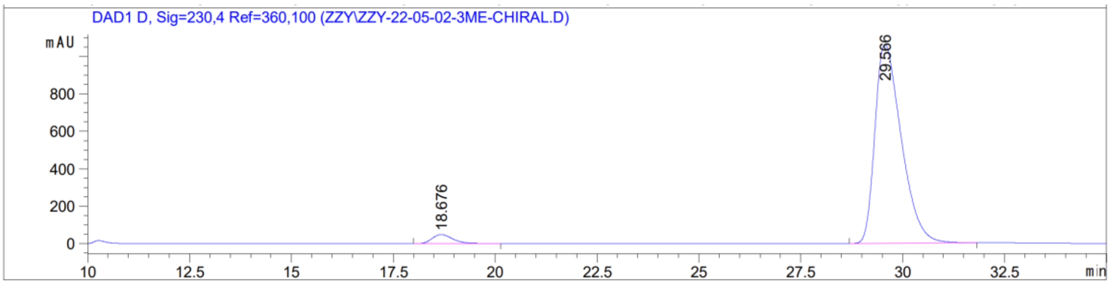

Peak #	RetTime [min]	Type	Width [min]	Area [mAU*s]	Height [mAU]	Area %
1	18.676	BB	0.5388	1681.88684	47.75959	3.3978
2	29.566	BB	0.6689	4.78179e4	1062.89856	96.6022

(*S*)-2-(dimethyl(phenyl)silyl)-2-(p-tolyl)aziridine [(*S*)-**3ca**]

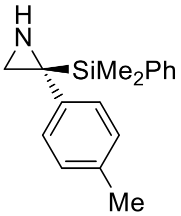

(*S*)-**3ca**

rac-**3ca**

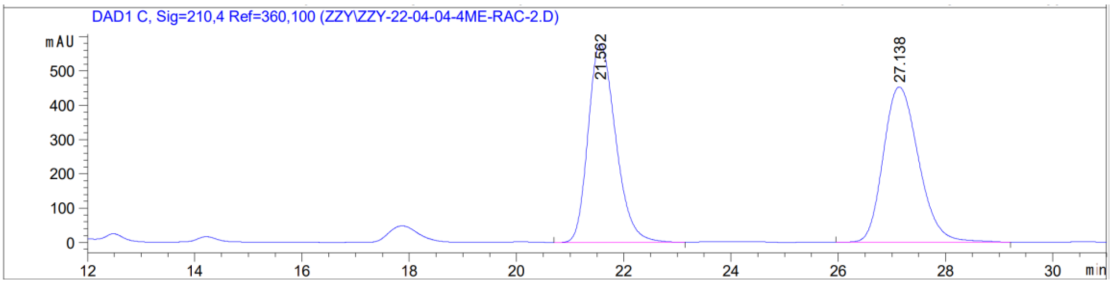

Peak #	RetTime [min]	Type	Width [min]	Area [mAU*s]	Height [mAU]	Area %
1	21.562	BB	0.5553	2.06229e4	579.37018	49.8679
2	27.138	BB	0.7162	2.07322e4	452.80460	50.1321

(*S*)-**3ca** (90% *ee*)

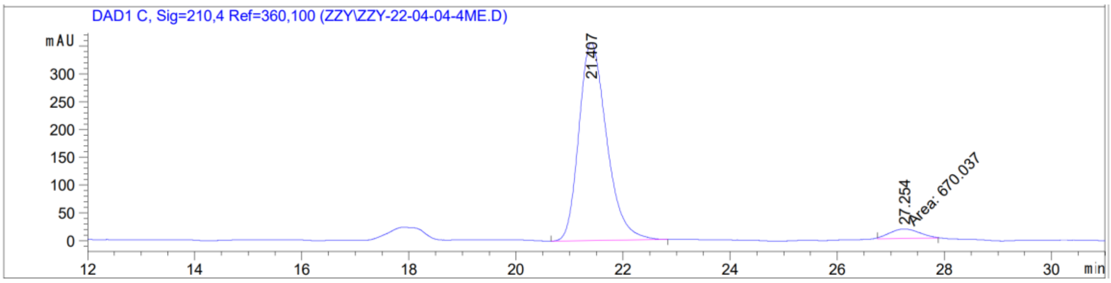

Peak #	RetTime [min]	Type	Width [min]	Area [mAU*s]	Height [mAU]	Area %
1	21.407	BB	0.5560	1.28290e4	356.40475	95.0364
2	27.254	MM	0.6463	670.03741	17.27763	4.9636

(*S*)-2-(dimethyl(phenyl)silyl)-2-(4-isopropylphenyl)aziridine [(*S*)-**3da**]

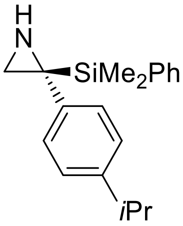

(*S*)-**3da**

rac-**3da**

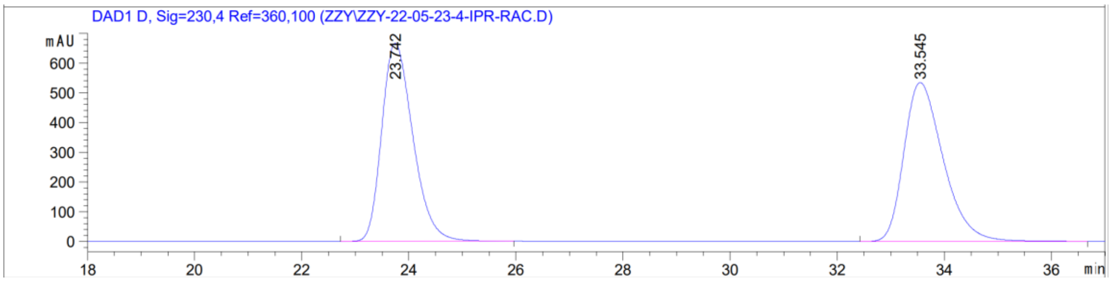

Peak #	RetTime [min]	Type	Width [min]	Area [mAU*s]	Height [mAU]	Area %
1	23.742	BB	0.6296	2.69531e4	666.79266	49.7453
2	33.545	BB	0.7842	2.72292e4	534.66241	50.2547

(*S*)-**3da** (90% *ee*)

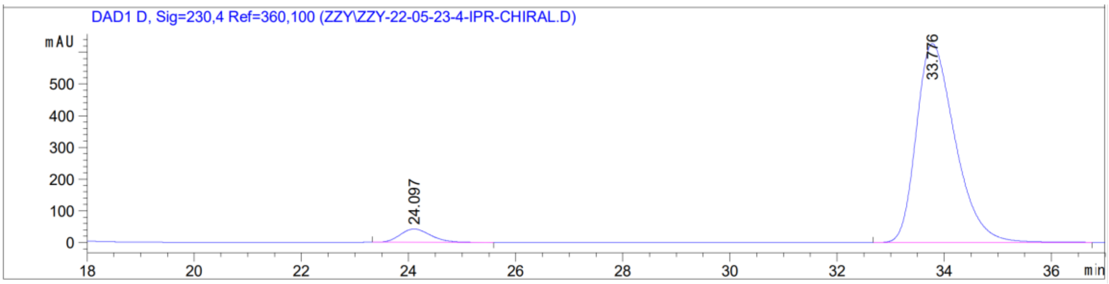

Peak #	RetTime [min]	Type	Width [min]	Area [mAU*s]	Height [mAU]	Area %
1	24.097	BB	0.6028	1678.68152	42.13691	5.0655
2	33.776	BB	0.7695	3.14608e4	626.86328	94.9345

(*S*)-2-(4-(*tert*-butyl)phenyl)-2-(dimethyl(phenyl)silyl)aziridine [(*S*)-**3ea**]

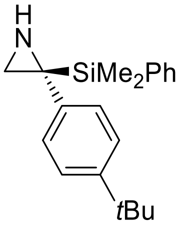

(*S*)-**3ea**

rac-**3ea**

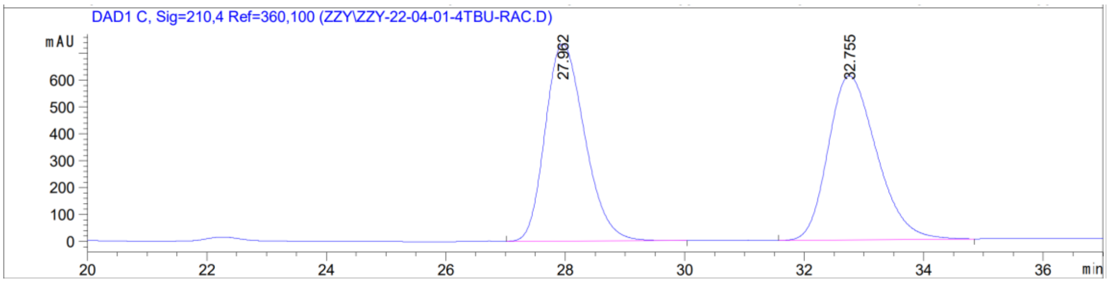

Peak #	RetTime [min]	Type	Width [min]	Area [mAU*s]	Height [mAU]	Area %
1	27.962	BB	0.7158	3.37274e4	734.36530	49.4929
2	32.755	BB	0.8488	3.44185e4	611.37701	50.5071

(*S*)-**3ea** (91% *ee*)

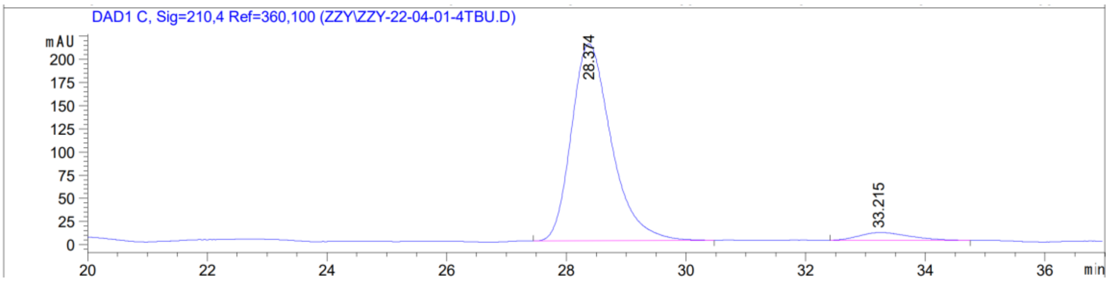

Peak #	RetTime [min]	Type	Width [min]	Area [mAU*s]	Height [mAU]	Area %
1	28.374	BB	0.7093	9862.77441	212.55690	95.2738
2	33.215	BB	0.6840	489.26056	8.44384	4.7262

(*S*)-2-(dimethyl(phenyl)silyl)-2-(3,4-dimethylphenyl)aziridine [(*S*)-**3fa**]

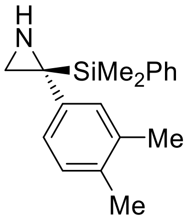

(*S*)-**3fa**

rac-**3fa**

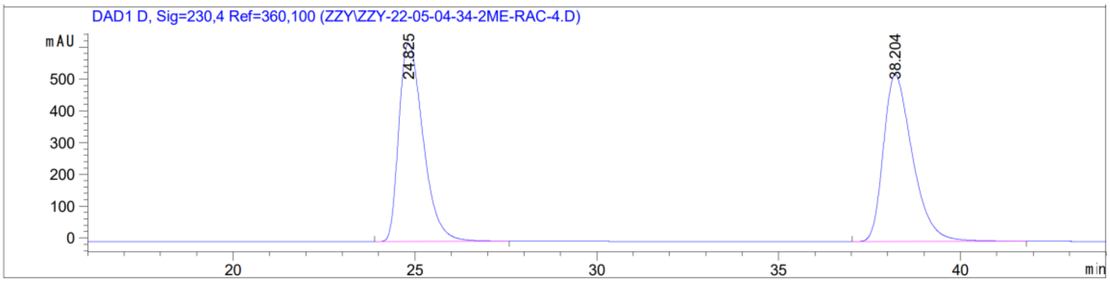

Peak #	RetTime [min]	Type	Width [min]	Area [mAU*s]	Height [mAU]	Area %
1	24.825	BB	0.7155	2.88576e4	624.00616	49.6998
2	38.204	BB	0.8184	2.92062e4	525.54095	50.3002

(*S*)-**3fa** (95% *ee*)

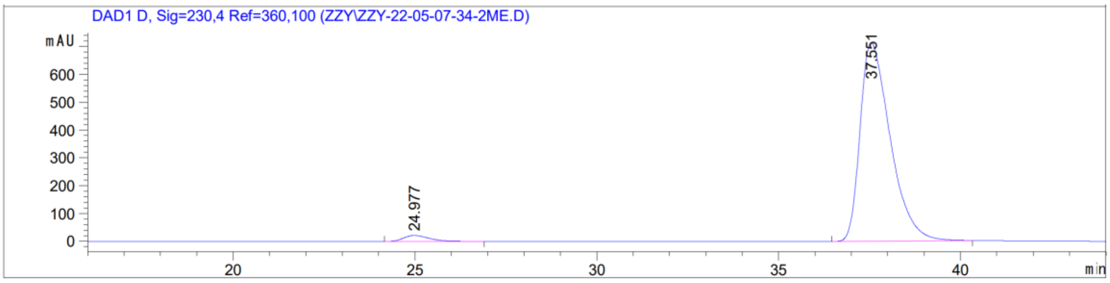

Peak #	RetTime [min]	Type	Width [min]	Area [mAU*s]	Height [mAU]	Area %
1	24.977	BB	0.6794	1062.44446	21.54081	2.5229
2	37.551	BB	0.8348	4.10491e4	713.94800	97.4771

(*S*)-2-(4-(2-(dimethyl(phenyl)silyl)aziridin-2-yl)benzyl)isoindoline-1,3-dione [(*S*)-3ga]

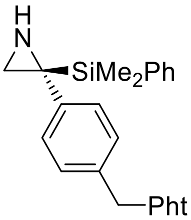

(*S*)-**3ga**

rac-**3ga**

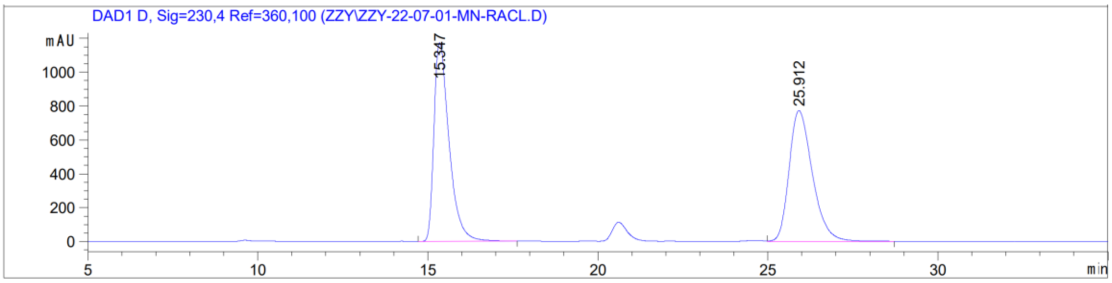

Peak #	RetTime [min]	Type	Width [min]	Area [mAU*s]	Height [mAU]	Area %
1	15.347	BB	0.4718	3.63753e4	1173.04883	49.7707
2	25.912	VB	0.7243	3.67105e4	772.72388	50.2293

(*S*)-**3ga** (92% *ee*)

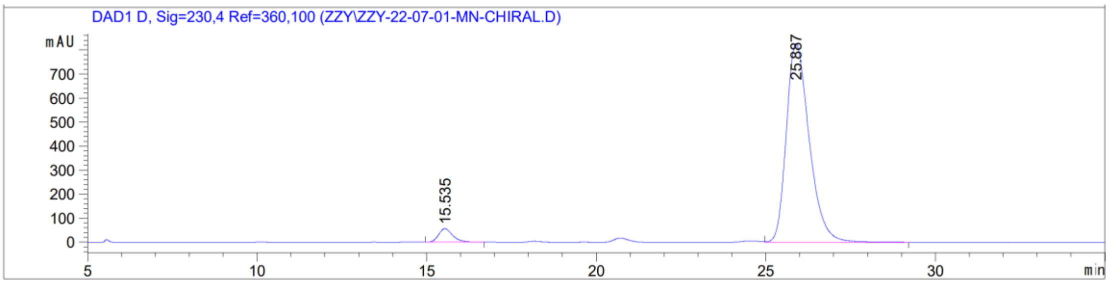

Peak #	RetTime [min]	Type	Width [min]	Area [mAU*s]	Height [mAU]	Area %
1	15.535	BB	0.4356	1626.31958	56.53765	4.0179
2	25.887	VB	0.7240	3.88505e4	827.14386	95.9821

(*S*)-2-([1,1'-biphenyl]-4-yl)-2-(dimethyl(phenyl)silyl)aziridine [(*S*)-**3ha**]

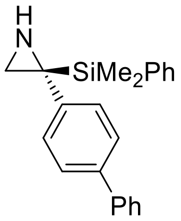

(*S*)-**3ha**

rac-**3ha**

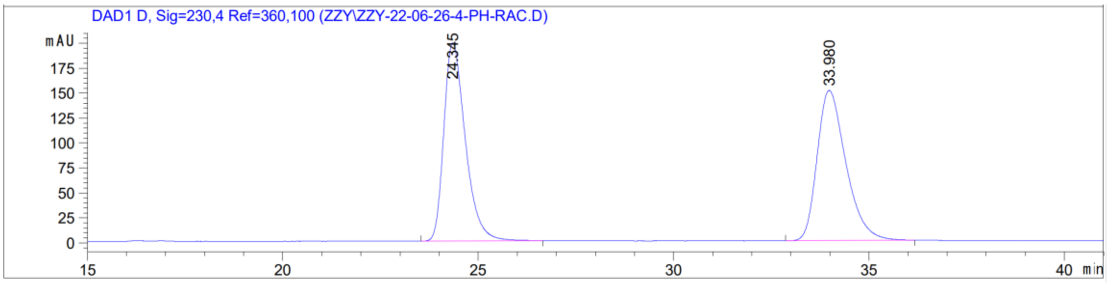

Peak #	RetTime [min]	Type	Width [min]	Area [mAU*s]	Height [mAU]	Area %
1	24.345	BB	0.5852	7580.45215	198.63576	49.6244
2	33.980	BB	0.7730	7695.20898	150.39223	50.3756

(*S*)-**3ha** (92% *ee*)

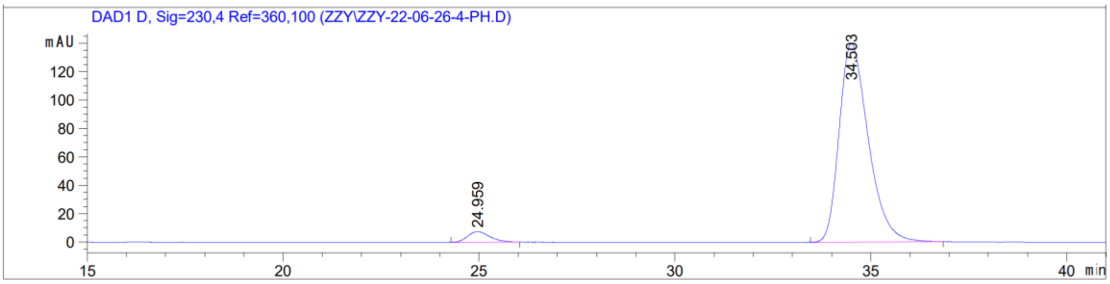

Peak #	RetTime [min]	Type	Width [min]	Area [mAU*s]	Height [mAU]	Area %
1	24.959	BB	0.5140	293.23047	7.46913	3.9020
2	34.503	BB	0.7805	7221.57324	139.83250	96.0980

(*S*)-2-(dimethyl(phenyl)silyl)-2-(naphthalen-2-yl)aziridine [(*S*)-**3ia**]

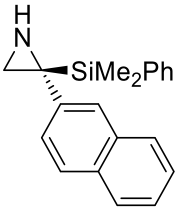

(*S*)-**3ia**

rac-**3ia**

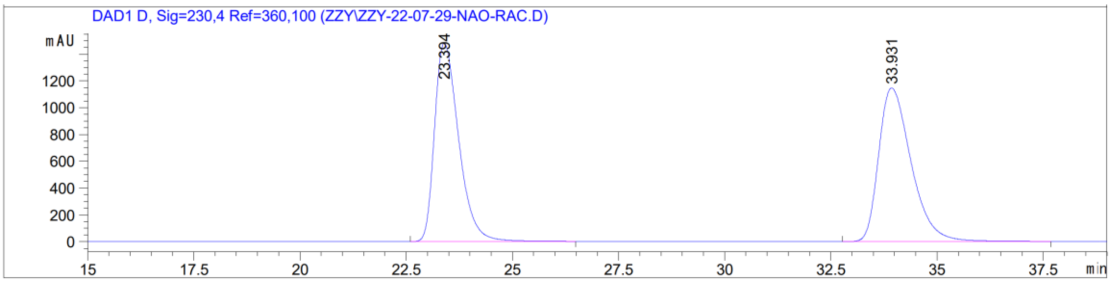

Peak #	RetTime [min]	Type	Width [min]	Area [mAU*s]	Height [mAU]	Area %
1	23.394	BB	0.6069	5.85606e4	1482.43726	49.0301
2	33.931	BB	0.7934	6.08774e4	1146.68726	50.9699

(*S*)-**3ia** (91% *ee*)

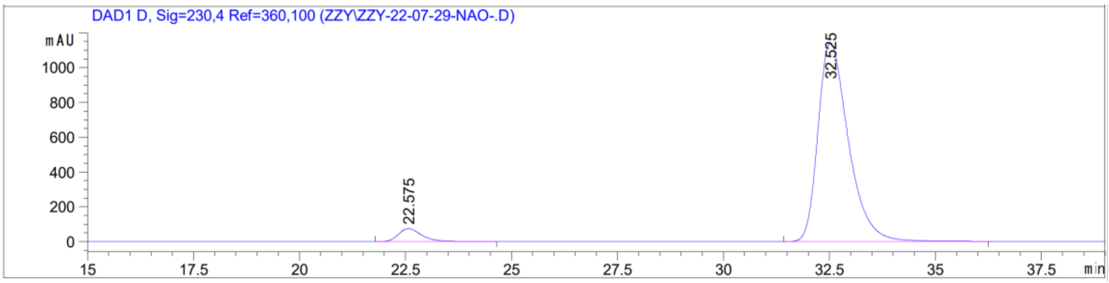

Peak #	RetTime [min]	Type	Width [min]	Area [mAU*s]	Height [mAU]	Area %
1	22.575	BB	0.5704	2829.51709	74.27835	4.7321
2	32.525	BB	0.7495	5.69646e4	1142.97815	95.2679

(*S*)-2-(dimethyl(phenyl)silyl)-2-(3-methoxyphenyl)aziridine [(*S*)-**3ja**]

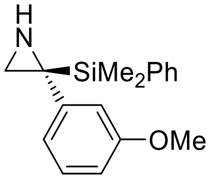

(*S*)-**3ja**

rac-**3ja**

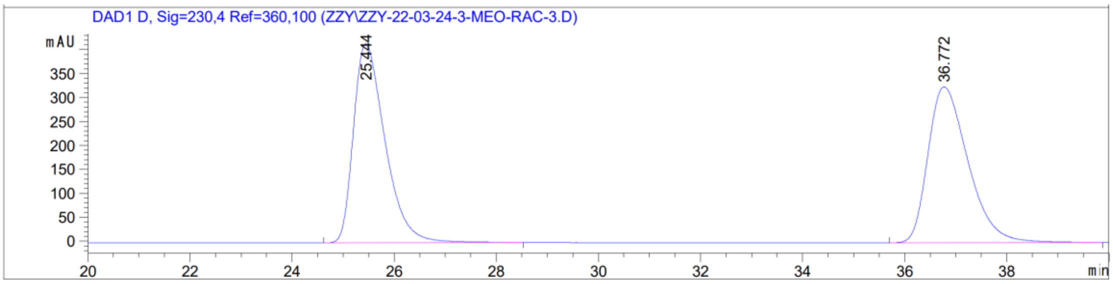

Peak #	RetTime [min]	Type	Width [min]	Area [mAU*s]	Height [mAU]	Area %
1	25.444	BB	0.6589	1.78468e4	415.67383	50.1246
2	36.772	BB	0.8386	1.77581e4	325.43237	49.8754

(*S*)-**3ja** (92% *ee*)

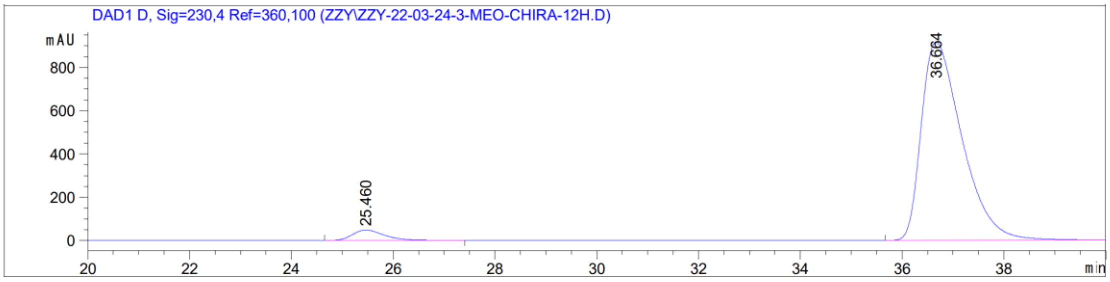

Peak #	RetTime [min]	Type	Width [min]	Area [mAU*s]	Height [mAU]	Area %
1	25.460	BB	0.6528	2076.92578	48.00374	3.9139
2	36.664	BBA	0.8421	5.09882e4	917.82214	96.0861

(*S*)-2-(dimethyl(phenyl)silyl)-2-(4-methoxyphenyl)aziridine [(*S*)-**3ka**]

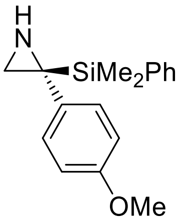

(*S*)-**3ka**

rac-**3ka**

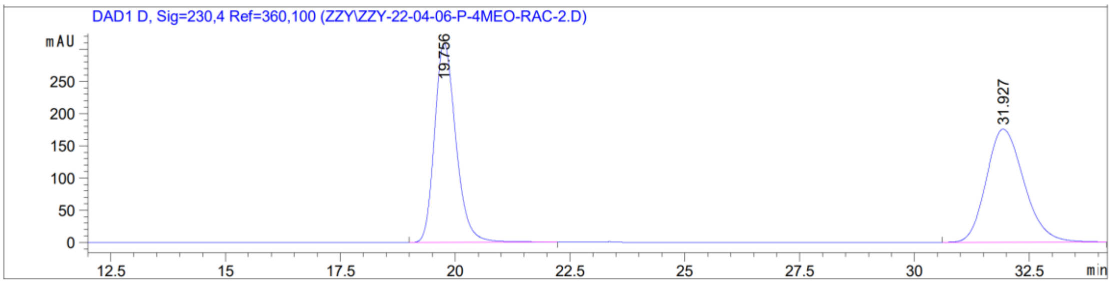

Peak #	RetTime [min]	Type	Width [min]	Area [mAU*s]	Height [mAU]	Area %
1	19.756	BB	0.4988	9990.97070	309.37469	49.9518
2	31.927	BBA	0.8926	1.00102e4	175.73314	50.0482

(*S*)-**3ka** (90% *ee*)

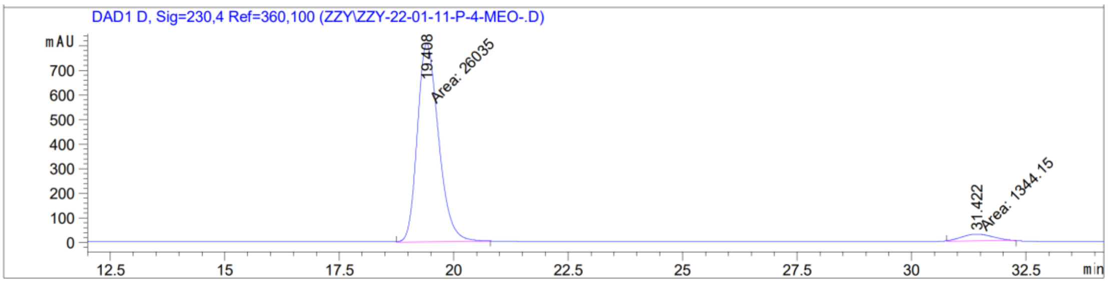

Peak #	RetTime [min]	Type	Width [min]	Area [mAU*s]	Height [mAU]	Area %
1	19.408	MM	0.5379	2.60350e4	806.75934	95.0906
2	31.422	MM	0.7935	1344.15186	28.23378	4.9094

(*S*)-2-(3-(benzyloxy)phenyl)-2-(dimethyl(phenyl)silyl)aziridine [(*S*)-**3la**]

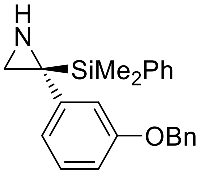

(*S*)-**3la**

rac-**3la**

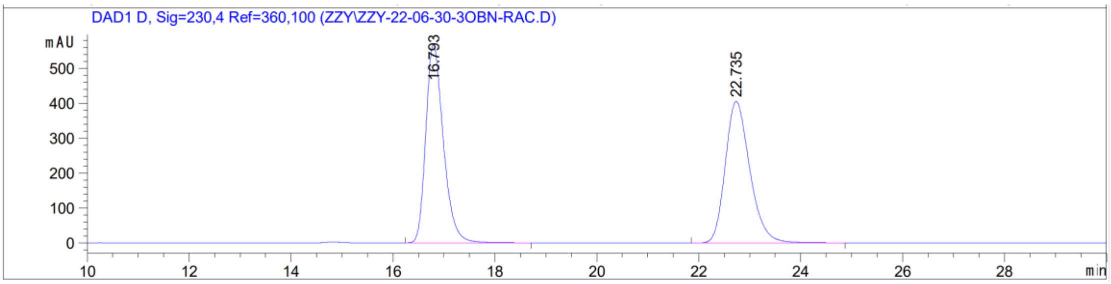

Peak #	RetTime [min]	Type	Width [min]	Area [mAU*s]	Height [mAU]	Area %
1	16.793	BB	0.3671	1.36052e4	568.65680	50.0304
2	22.735	BB	0.5133	1.35887e4	405.14624	49.9696

(*S*)-**3la** (95% *ee*)

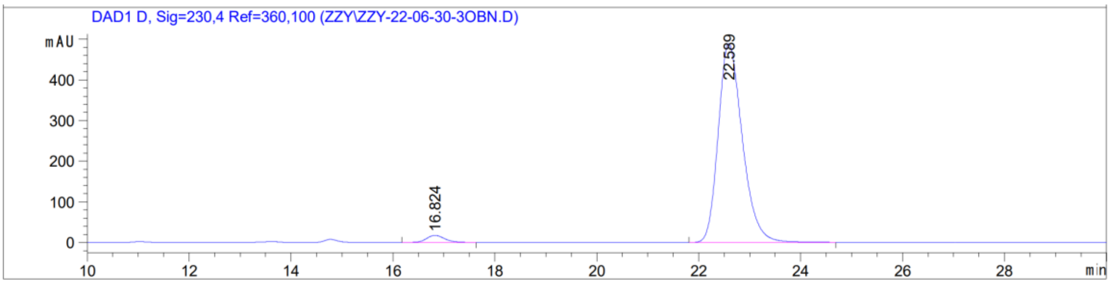

Peak #	RetTime [min]	Type	Width [min]	Area [mAU*s]	Height [mAU]	Area %
1	16.824	BB	0.3766	439.02170	17.50492	2.6558
2	22.589	BB	0.5061	1.60919e4	488.76630	97.3442

(*S*)-2-(4-(benzyloxy)phenyl)-2-(dimethyl(phenyl)silyl)aziridine [(*S*)-**3ma**]

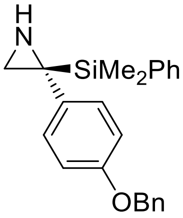

(*S*)-**3ma**

rac-**3ma**

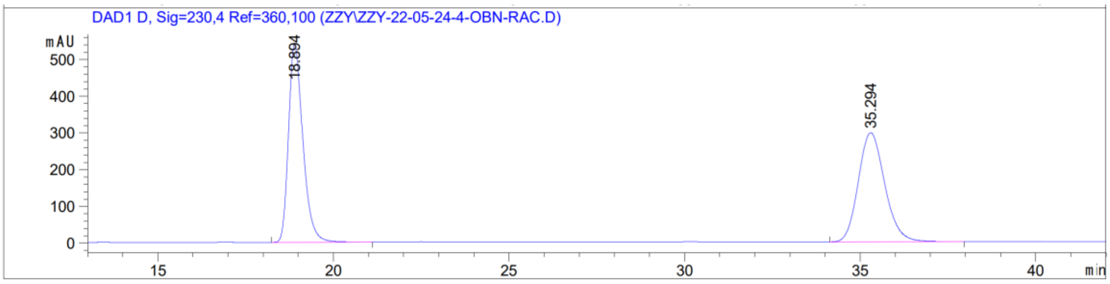

Peak #	RetTime [min]	Type	Width [min]	Area [mAU*s]	Height [mAU]	Area %
1	18.894	BB	0.4364	1.53873e4	540.08740	49.7511
2	35.294	BB	0.8057	1.55413e4	297.39380	50.2489

(*S*)-**3ma** (95% *ee*)

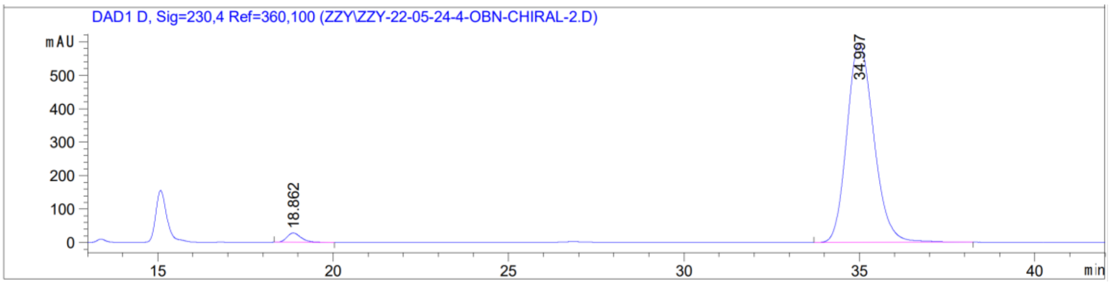

Peak #	RetTime [min]	Type	Width [min]	Area [mAU*s]	Height [mAU]	Area %
1	18.862	BB	0.4284	779.04578	28.01669	2.3974
2	34.997	BB	0.8270	3.17169e4	593.94373	97.6026

(*S*)-2-(benzo[d][1,3]dioxol-5-yl)-2-(dimethyl(phenyl)silyl)aziridine [(*S*)-**3na**]

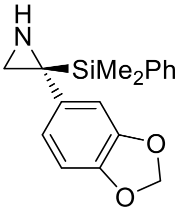

(*S*)-**3na**

rac-**3na**

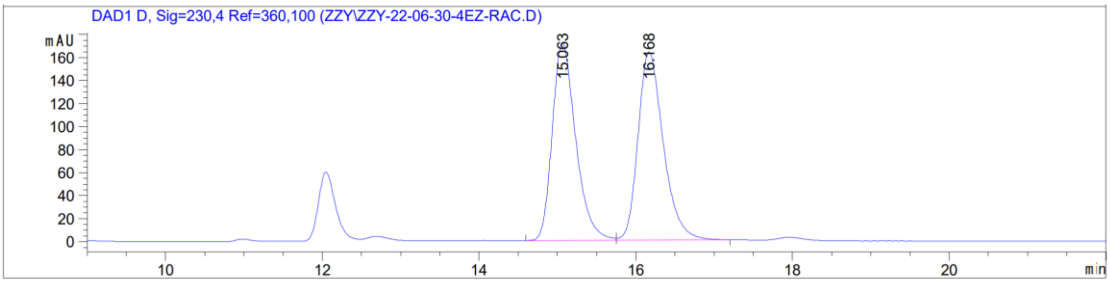

Peak #	RetTime [min]	Type	Width [min]	Area [mAU*s]	Height [mAU]	Area %
1	15.063	BV	0.3181	3565.10986	171.83615	49.3280
2	16.168	VB	0.3412	3662.25171	163.56862	50.6720

(*S*)-**3na** (96% *ee*)

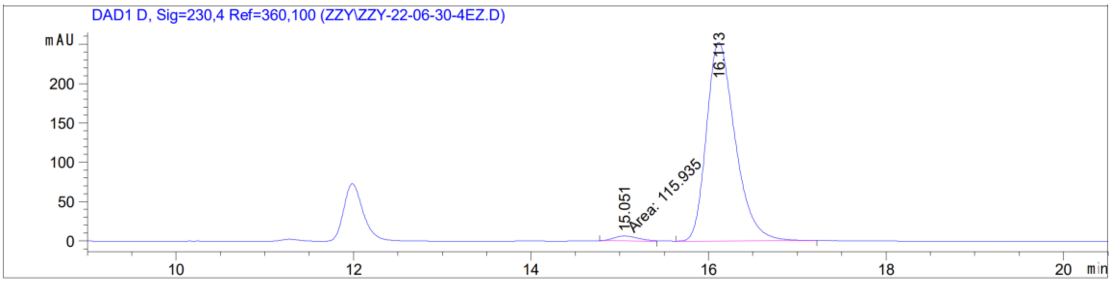

Peak #	RetTime [min]	Type	Width [min]	Area [mAU*s]	Height [mAU]	Area %
1	15.051	MM	0.3175	115.93504	6.08635	2.0020
2	16.113	BB	0.3419	5675.06543	252.80125	97.9980

(*S*)-2-(dimethyl(phenyl)silyl)-2-(4-(methylthio)phenyl)aziridine [(*S*)-**3oa**]

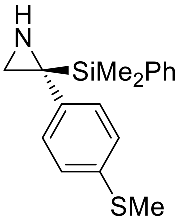

(*S*)-**3oa**

rac-**3oa**

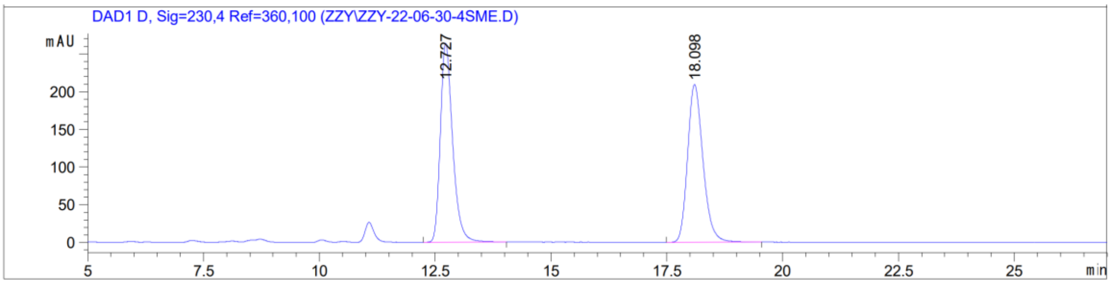

Peak #	RetTime [min]	Type	Width [min]	Area [mAU*s]	Height [mAU]	Area %
1	12.727	BB	0.2815	4871.59814	263.75394	50.2171
2	18.098	BB	0.3554	4829.47070	209.11719	49.7829

(*S*)-**3oa** (95% *ee*)

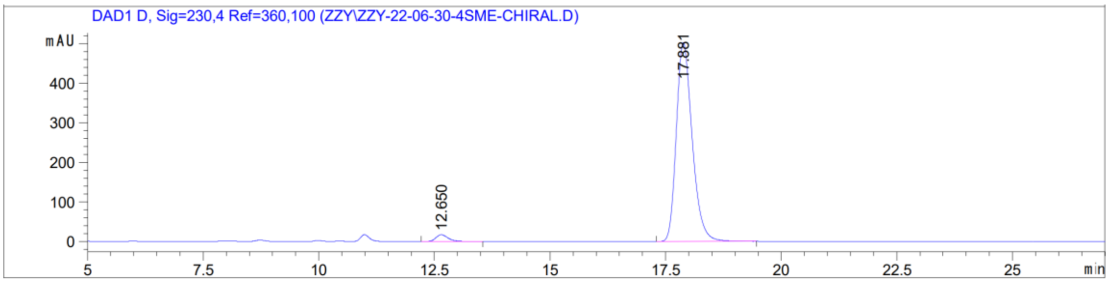

Peak #	RetTime [min]	Type	Width [min]	Area [mAU*s]	Height [mAU]	Area %
1	12.650	BB	0.2784	321.16779	17.31676	2.6152
2	17.881	BB	0.3657	1.19596e4	502.44318	97.3848

(*S*)-4-(2-(dimethyl(phenyl)silyl)aziridin-2-yl)phenyl acetate [(*S*)-**3pa**]

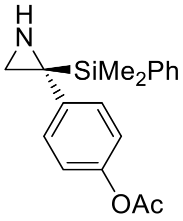

(*S*)-**3pa**

rac-**3pa**

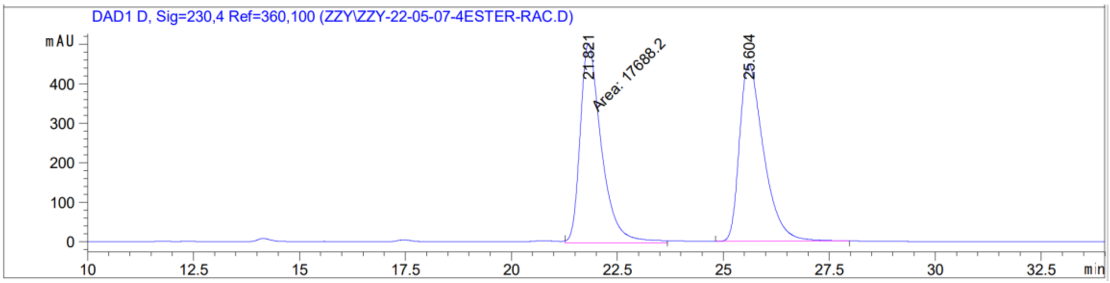

Peak #	RetTime [min]	Type	Width [min]	Area [mAU*s]	Height [mAU]	Area %
1	21.821	MM	0.5826	1.76882e4	506.00778	49.7618
2	25.604	BB	0.6012	1.78575e4	449.81216	50.2382

(*S*)-**3pa** (92% *ee*)

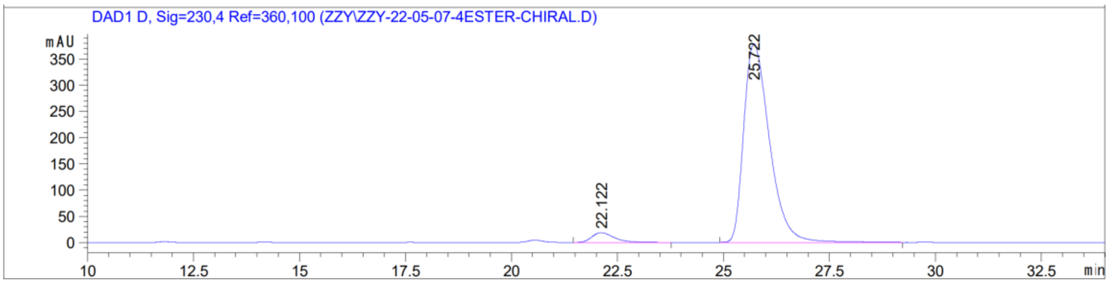

Peak #	RetTime [min]	Type	Width [min]	Area [mAU*s]	Height [mAU]	Area %
1	22.122	BB	0.5507	688.03143	18.55198	4.1285
2	25.722	BB	0.6563	1.59775e4	378.66782	95.8715

(*S*)-2-(dimethyl(phenyl)silyl)-2-(4-(trifluoromethoxy)phenyl)aziridine [(*S*)-**3qa**]

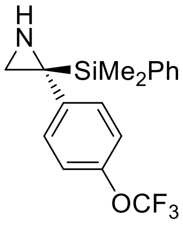

(*S*)-**3qa**

rac-**3qa**

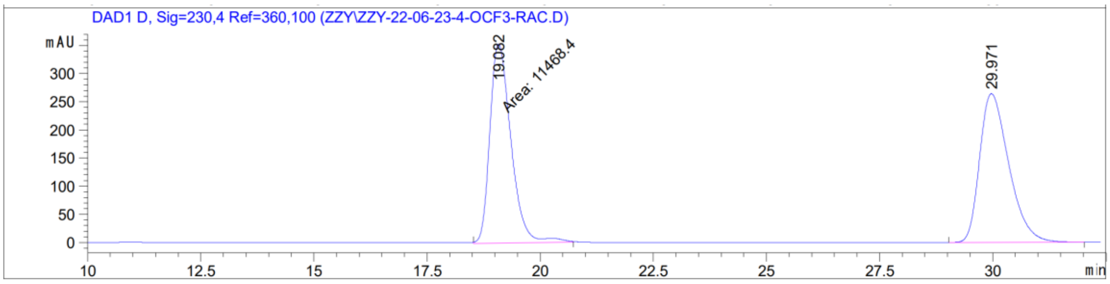

Peak #	RetTime [min]	Type	Width [min]	Area [mAU*s]	Height [mAU]	Area %
1	19.082	MM	0.5406	1.14684e4	353.57779	49.2098
2	29.971	BB	0.6825	1.18367e4	264.31131	50.7902

(*S*)-**3qa** (90% *ee*)

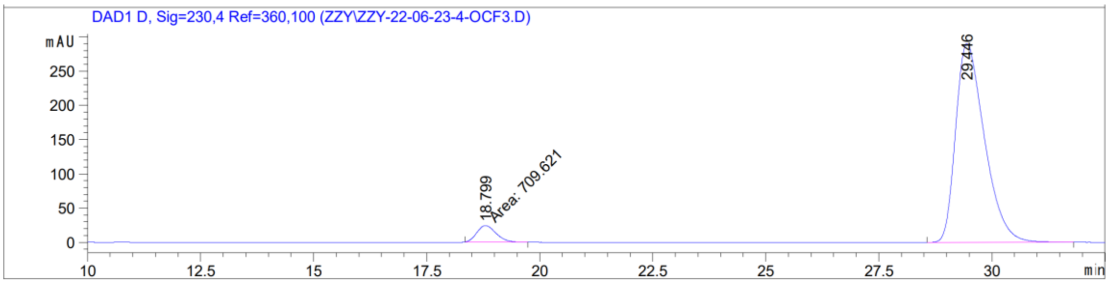

Peak #	RetTime [min]	Type	Width [min]	Area [mAU*s]	Height [mAU]	Area %
1	18.799	MM	0.4901	709.62073	24.12953	5.2250
2	29.446	BB	0.6695	1.28717e4	290.20645	94.7750

(*S*)-2-(dimethyl(phenyl)silyl)-2-(4-(trifluoromethyl)phenyl)aziridine [(*S*)-**3ra**]

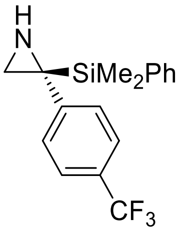

(*S*)-**3ra**

rac-**3ra**

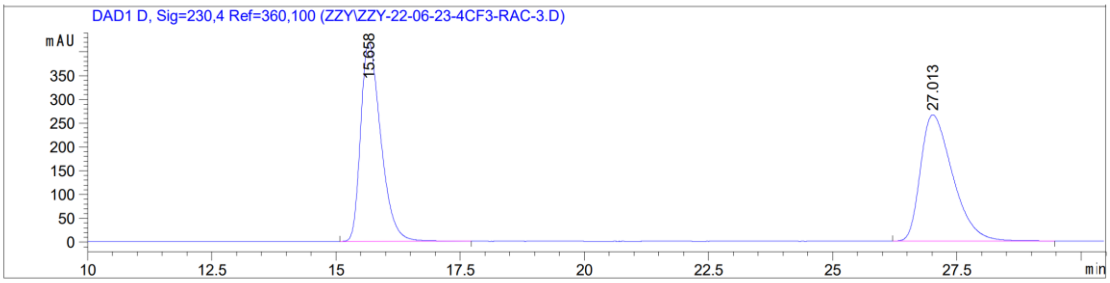

Peak #	RetTime [min]	Type	Width [min]	Area [mAU*s]	Height [mAU]	Area %
1	15.658	BB	0.4345	1.17531e4	417.40616	49.6443
2	27.013	BB	0.6966	1.19216e4	265.14645	50.3557

(*S*)-**3ra** (92% *ee*)

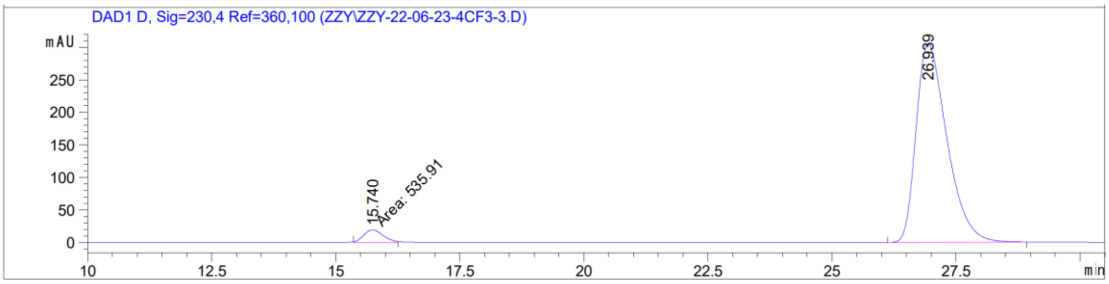

Peak #	RetTime [min]	Type	Width [min]	Area [mAU*s]	Height [mAU]	Area %
1	15.740	MM	0.4556	535.91040	19.60266	3.9126
2	26.939	BB	0.6632	1.31612e4	305.13428	96.0874

(*S*)-2-(dimethyl(phenyl)silyl)-2-(4-isocyanophenyl)aziridine [(*S*)-**3sa**]

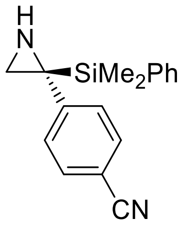

(*S*)-**3sa**

rac-**3sa**

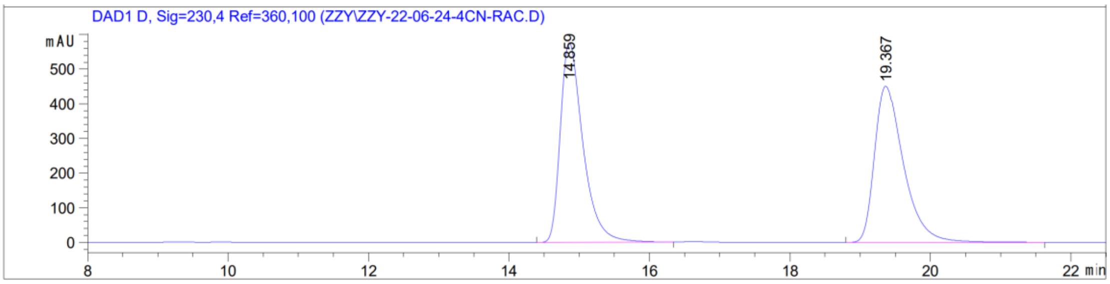

Peak #	RetTime [min]	Type	Width [min]	Area [mAU*s]	Height [mAU]	Area %
1	14.859	BB	0.3361	1.28056e4	574.34412	49.7381
2	19.367	BB	0.4354	1.29405e4	450.18283	50.2619

(*S*)-**3sa** (94% *ee*)

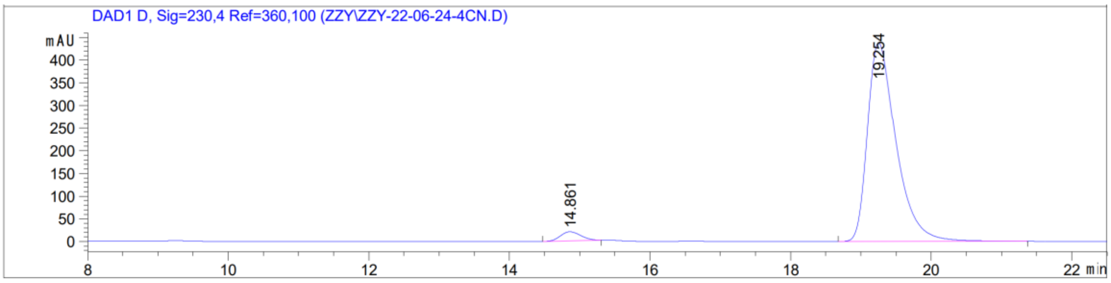

Peak #	RetTime [min]	Type	Width [min]	Area [mAU*s]	Height [mAU]	Area %
1	14.861	BB	0.3153	400.88428	20.04886	3.1377
2	19.254	BB	0.4274	1.23755e4	438.34818	96.8623

(*S*)-1-(4-(2-(dimethyl(phenyl)silyl)aziridin-2-yl)phenyl)ethan-1-one [(*S*)-**3ta**]

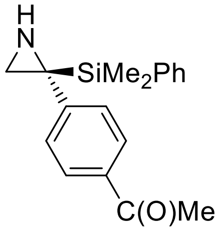

(*S*)-**3ta**

rac-**3ta**

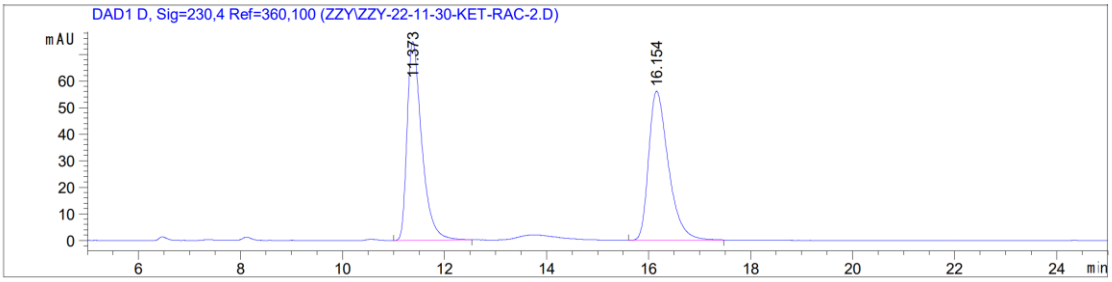

Peak #	RetTime [min]	Type	Width [min]	Area [mAU*s]	Height [mAU]	Area %
1	11.373	BB	0.3000	1488.21887	74.84998	49.7145
2	16.154	BB	0.4052	1505.31213	56.07181	50.2855

(*S*)-**3ta** (95% *ee*)

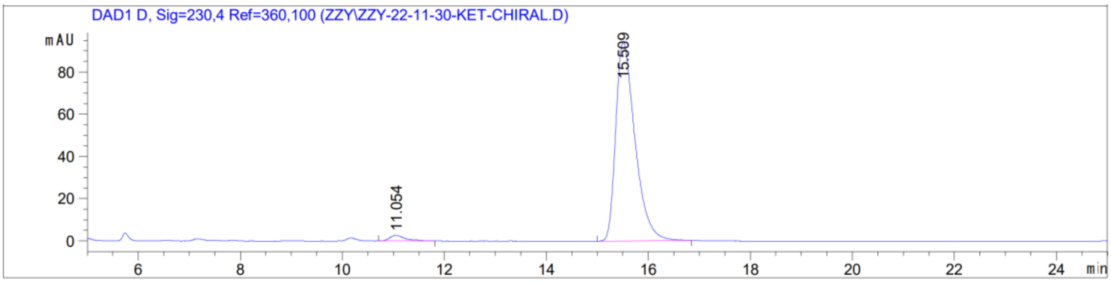

Peak #	RetTime [min]	Type	Width [min]	Area [mAU*s]	Height [mAU]	Area %
1	11.054	BB	0.3053	59.28860	2.68829	2.3434
2	15.509	BB	0.3980	2470.73315	94.19841	97.6566

(*S*)-2-(dimethyl(phenyl)silyl)-2-(3-fluorophenyl)aziridine [(*S*)-**3ua**]

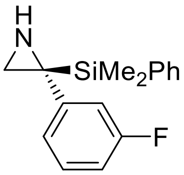

(*S*)-**3ua**

rac-**3ua**

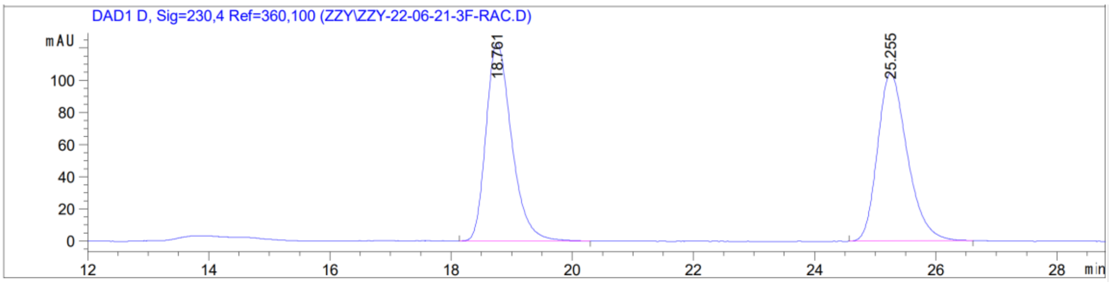

Peak #	RetTime [min]	Type	Width [min]	Area [mAU*s]	Height [mAU]	Area %
1	18.761	BB	0.4499	3613.70361	123.31605	50.3592
2	25.255	BB	0.5222	3562.15747	104.37632	49.6408

(*S*)-**3ua** (92% *ee*)

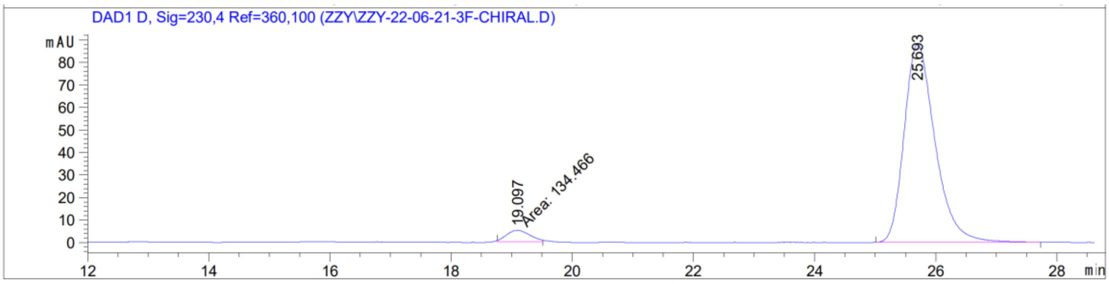

Peak #	RetTime [min]	Type	Width [min]	Area [mAU*s]	Height [mAU]	Area %
1	19.097	MM	0.4435	134.46597	5.05305	4.1365
2	25.693	BB	0.5308	3116.24951	88.07758	95.8635

(*S*)-2-(dimethyl(phenyl)silyl)-2-(4-fluorophenyl)aziridine [(*S*)-**3va**]

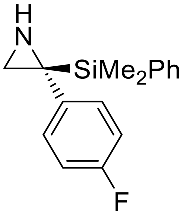

(*S*)-**3va**

rac-**3va**

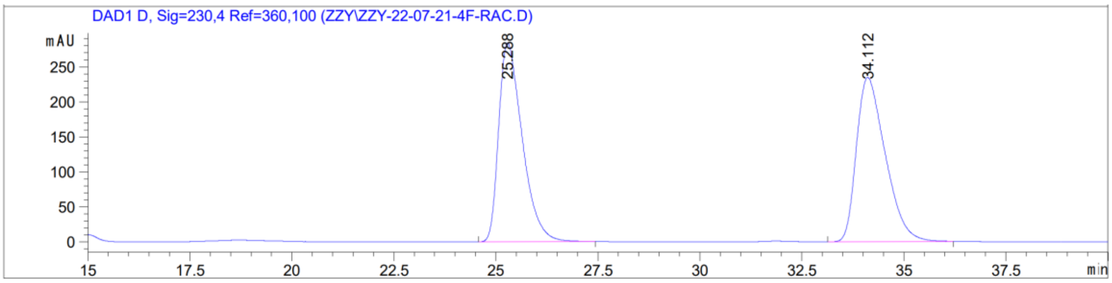

Peak #	RetTime [min]	Type	Width [min]	Area [mAU*s]	Height [mAU]	Area %
1	25.288	BB	0.5879	1.13991e4	284.21057	49.7090
2	34.112	BB	0.7394	1.15326e4	234.65775	50.2910

(*S*)-**3va** (92% *ee*)

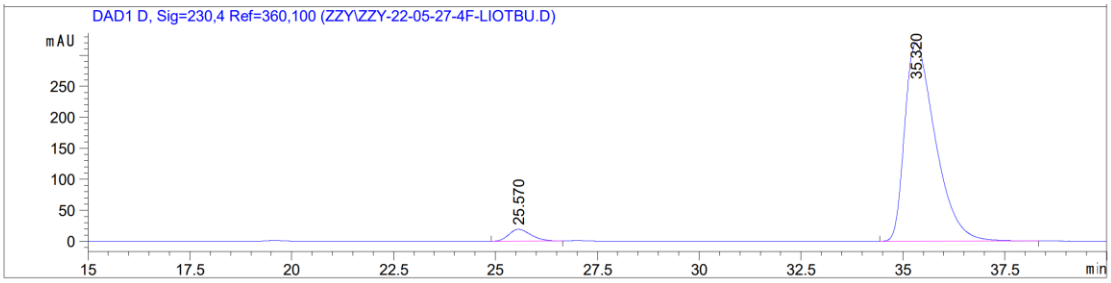

Peak #	RetTime [min]	Type	Width [min]	Area [mAU*s]	Height [mAU]	Area %
1	25.570	BB	0.5586	716.20428	19.05058	4.1184
2	35.320	BB	0.7942	1.66742e4	319.85989	95.8816

(*S*)-2-(3-chlorophenyl)-2-(dimethyl(phenyl)silyl)aziridine [(*S*)-**3wa**]

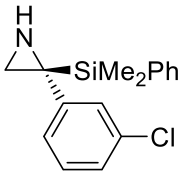

(*S*)-**3wa**

rac-**3wa**

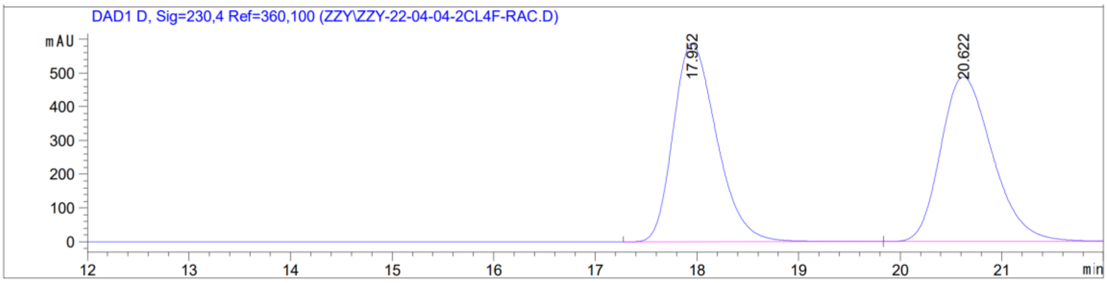

Peak #	RetTime [min]	Type	Width [min]	Area [mAU*s]	Height [mAU]	Area %
1	17.952	BB	0.4615	1.74158e4	588.05780	50.1817
2	20.622	BBA	0.5490	1.72897e4	488.35995	49.8183

(*S*)-**3wa** (92% *ee*)

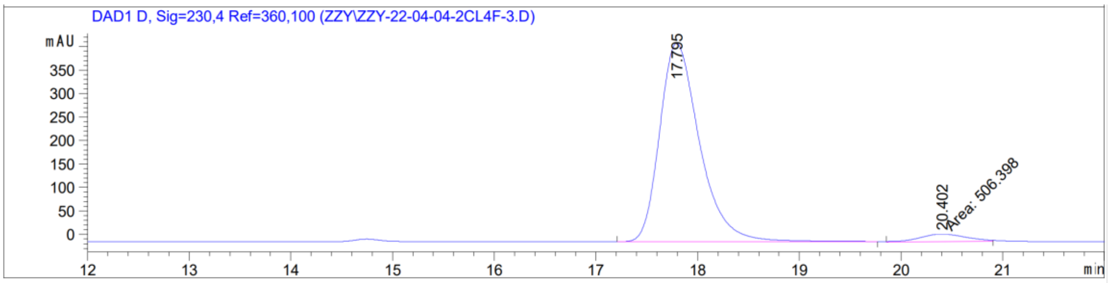

Peak #	RetTime [min]	Type	Width [min]	Area [mAU*s]	Height [mAU]	Area %
1	17.795	BB	0.4153	1.14970e4	422.75836	95.7812
2	20.402	MM	0.5249	506.39752	16.08022	4.2188

(*S*)-2-(4-chlorophenyl)-2-(dimethyl(phenyl)silyl)aziridine [(*S*)-**3xa**]

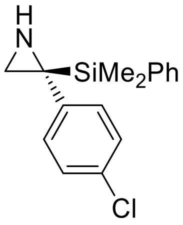

(*S*)-**3xa**

rac-**3xa**

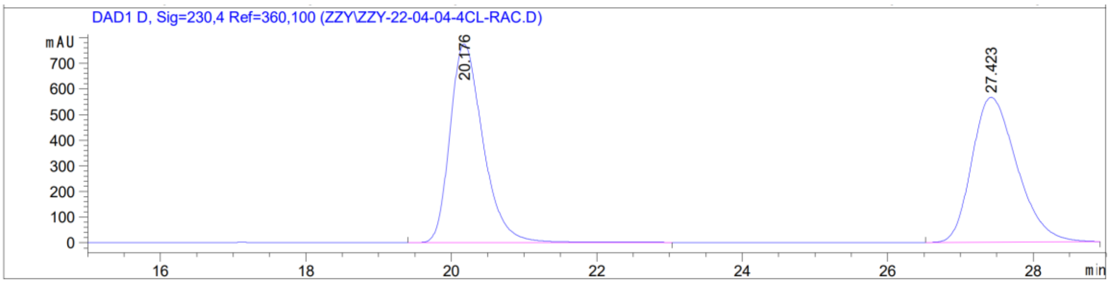

Peak #	RetTime [min]	Type	Width [min]	Area [mAU*s]	Height [mAU]	Area %
1	20.176	BB	0.4834	2.43778e4	774.18774	50.1266
2	27.423	BBA	0.6628	2.42547e4	565.01874	49.8734

(*S*)-**3xa** (93% *ee*)

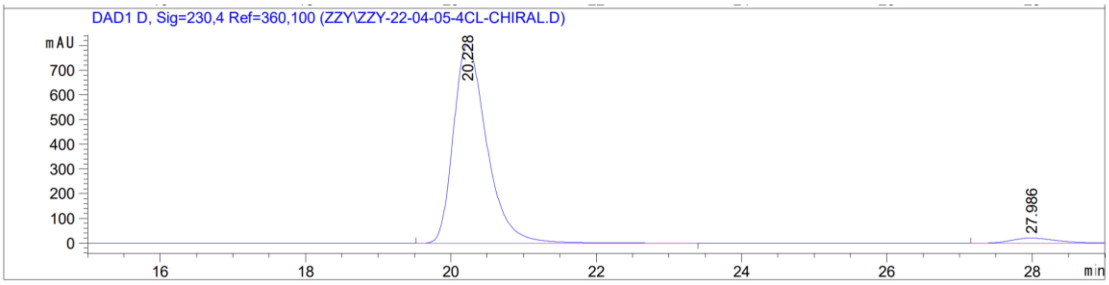

Peak #	RetTime [min]	Type	Width [min]	Area [mAU*s]	Height [mAU]	Area %
1	20.228	BB	0.4901	2.56537e4	804.37677	96.4636
2	27.986	BB	0.6390	940.48480	20.89823	3.5364

(*S*)-2-(3-bromophenyl)-2-(dimethyl(phenyl)silyl)aziridine [(*S*)-**3ya**]

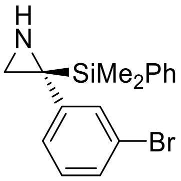

(*S*)-**3ya**

rac-**3ya**

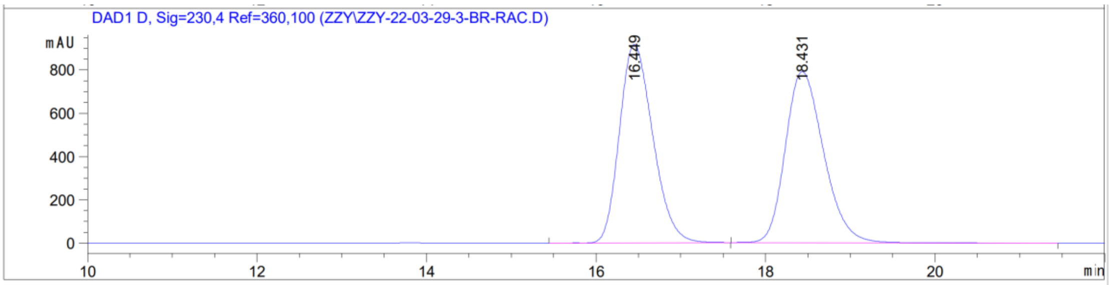

Peak #	RetTime [min]	Type	Width [min]	Area [mAU*s]	Height [mAU]	Area %
1	16.449	BB	0.4252	2.50550e4	915.78638	50.2801
2	18.431	BB	0.4840	2.47758e4	789.82471	49.7199

(*S*)-**3ya** (95% *ee*)

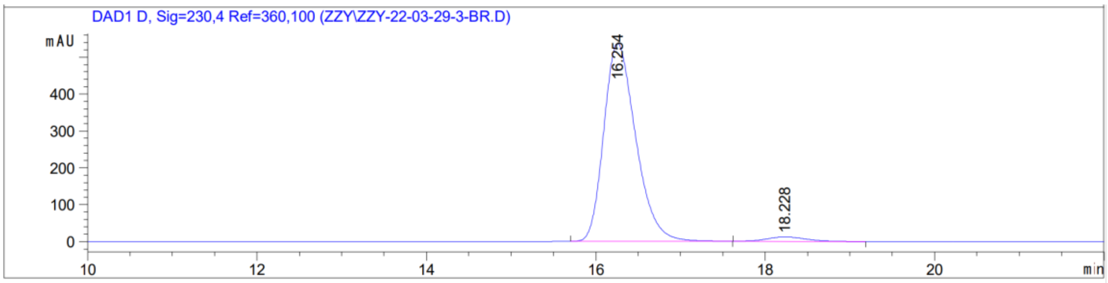

Peak #	RetTime [min]	Type	Width [min]	Area [mAU*s]	Height [mAU]	Area %
1	16.254	BB	0.4011	1.40335e4	536.68866	97.4022
2	18.228	BB	0.4833	374.28741	11.88810	2.5978

(*S*)-2-(4-bromophenyl)-2-(dimethyl(phenyl)silyl)aziridine [(*S*)-**3za**]

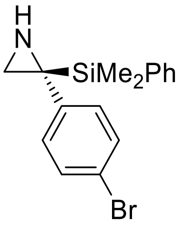

(*S*)-**3za**

rac-**3za**

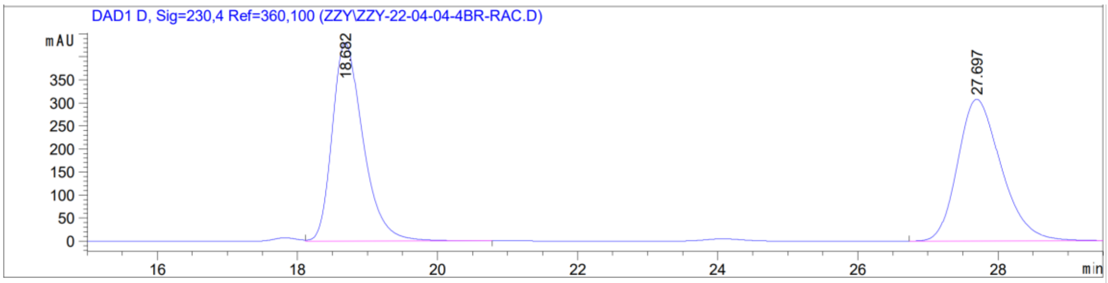

Peak #	RetTime [min]	Type	Width [min]	Area [mAU*s]	Height [mAU]	Area %
1	18.682	VB	0.4643	1.31499e4	430.56866	49.7489
2	27.697	BBA	0.6642	1.32826e4	307.35135	50.2511

(*S*)-**3za** (92% *ee*)

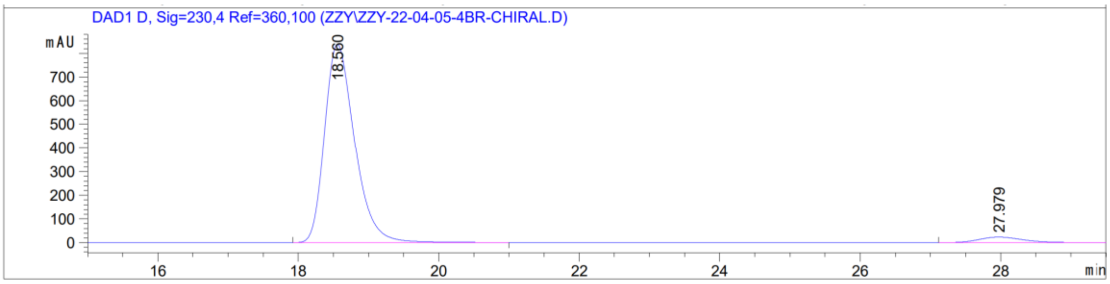

Peak #	RetTime [min]	Type	Width [min]	Area [mAU*s]	Height [mAU]	Area %
1	18.560	BB	0.4592	2.49621e4	838.71381	96.0566
2	27.979	BB	0.6351	1024.76831	23.21187	3.9434

(*S*)-2-(2,4-difluorophenyl)-2-(dimethyl(phenyl)silyl)aziridine [(*S*)-**3a’a**]

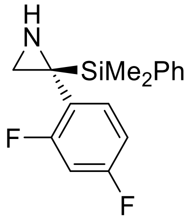

(*S*)-**3a'a**

rac-**3a’a**

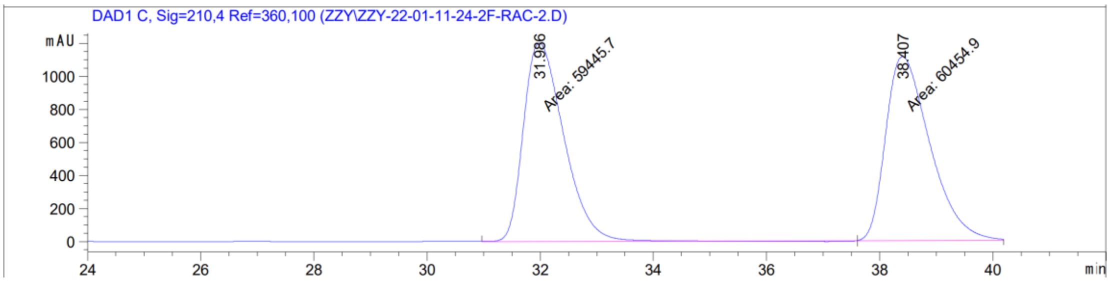

Peak #	RetTime [min]	Type	Width [min]	Area [mAU*s]	Height [mAU]	Area %
1	31.986	MF	0.8248	5.94457e4	1201.27087	49.5791
2	38.407	FM	0.9051	6.04549e4	1113.28748	50.4209

(*S*)-**3a’a** (86% *ee*)

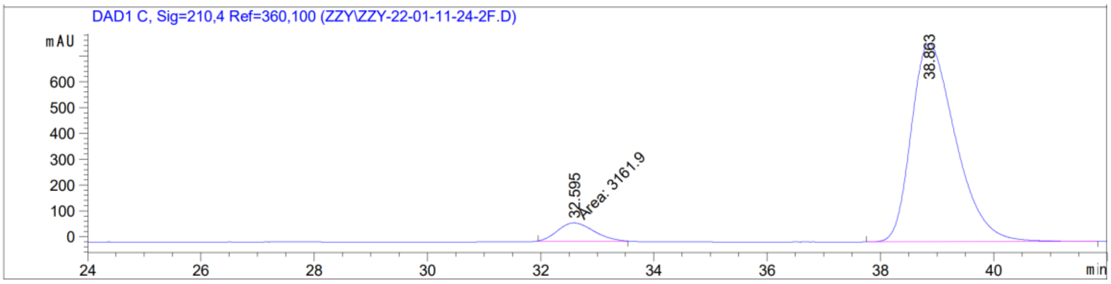

Peak #	RetTime [min]	Type	Width [min]	Area [mAU*s]	Height [mAU]	Area %
1	32.595	MM	0.7402	3161.89868	71.19887	7.0531
2	38.863	BB	0.8088	4.16682e4	768.20074	92.9469

(*S*)-2-(2-chloro-4-fluorophenyl)-2-(dimethyl(phenyl)silyl)aziridine [(*S*)-**3b'a**]

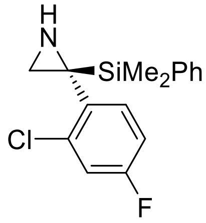

(*S*)-**3b'a**

rac-**3b'a**

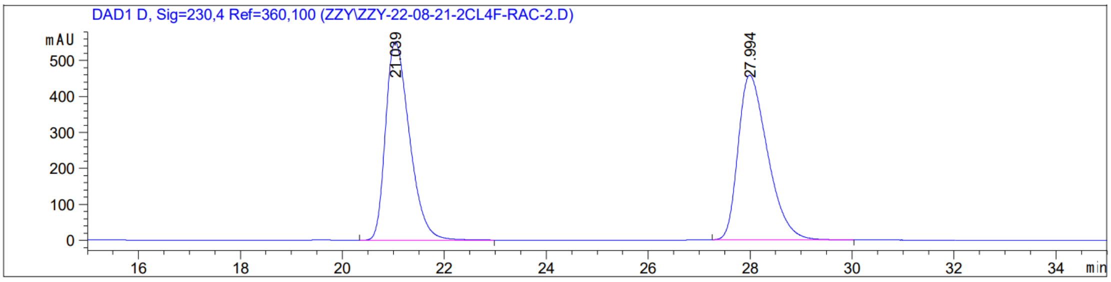

Peak #	RetTime [min]	Type	Width [min]	Area [mAU*s]	Height [mAU]	Area %
1	21.039	BB	0.5104	1.80072e4	552.33850	49.4704
2	27.994	BB	0.6090	1.83927e4	457.57324	50.5296

(*S*)-**3b'a** (50% *ee*)

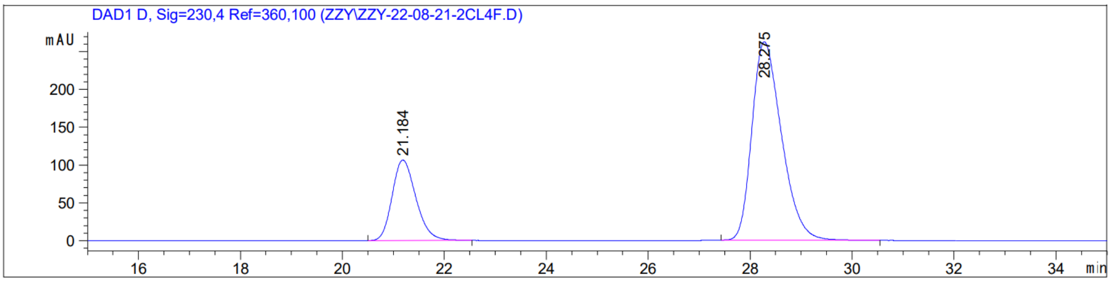

Peak #	RetTime [min]	Type	Width [min]	Area [mAU*s]	Height [mAU]	Area %
1	21.184	BB	0.4992	3464.26929	106.57021	24.8092
2	28.275	MM	0.6672	1.04994e4	262.28693	75.1908

(*S*)-2-(dimethyl(phenyl)silyl)-2-(thiophen-3-yl)aziridine [(*S*)-**3c'a**]

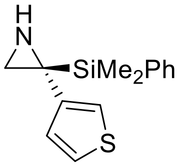

(*S*)-**3c'a**

rac-**3c'a**

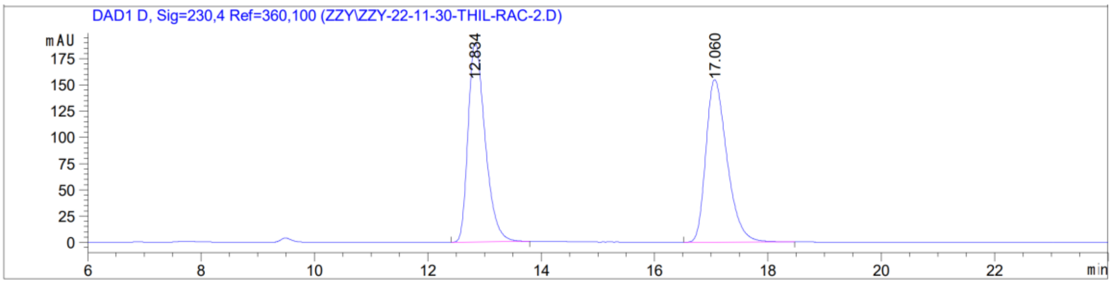

Peak #	RetTime [min]	Type	Width [min]	Area [mAU*s]	Height [mAU]	Area %
1	12.834	BB	0.3199	3970.03320	189.88528	49.7111
2	17.060	BB	0.3983	4016.17017	154.99419	50.2889

(*S*)-**3c'a** (77% *ee*)

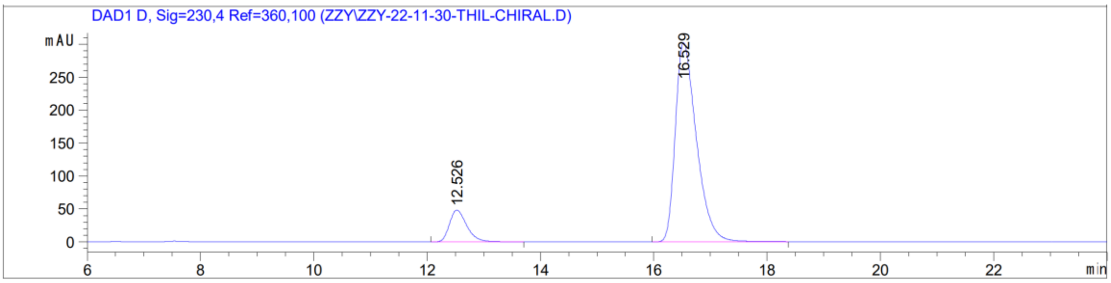

Peak #	RetTime [min]	Type	Width [min]	Area [mAU*s]	Height [mAU]	Area %
1	12.526	BB	0.3231	1023.20148	48.31425	11.5543
2	16.529	BB	0.3944	7832.38770	302.21951	88.4457

(*S*)-2-cyclohexyl-2-(dimethyl(phenyl)silyl)aziridine [(*S*)-**3d'a**]

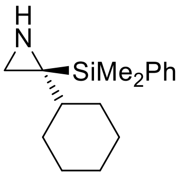

(*S*)-**3d'a**

rac-**3d'a**

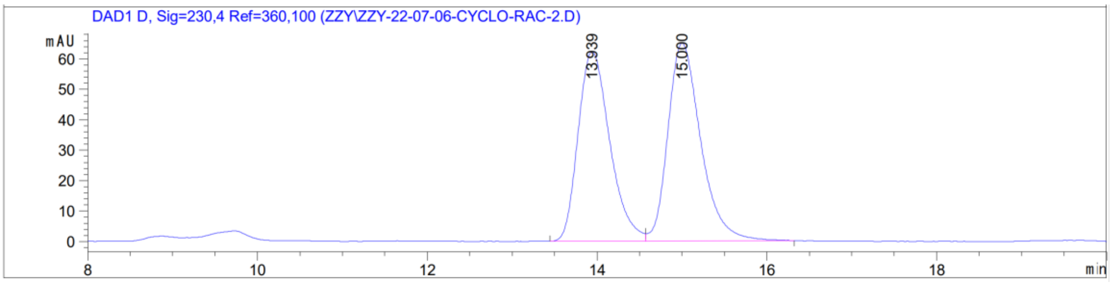

Peak #	RetTime [min]	Type	Width [min]	Area [mAU*s]	Height [mAU]	Area %
1	13.939	BV	0.4021	1613.39160	62.31247	48.0417
2	15.000	VB	0.4133	1744.92249	64.97966	51.9583

(*S*)-**3d'a** (80% *ee*)

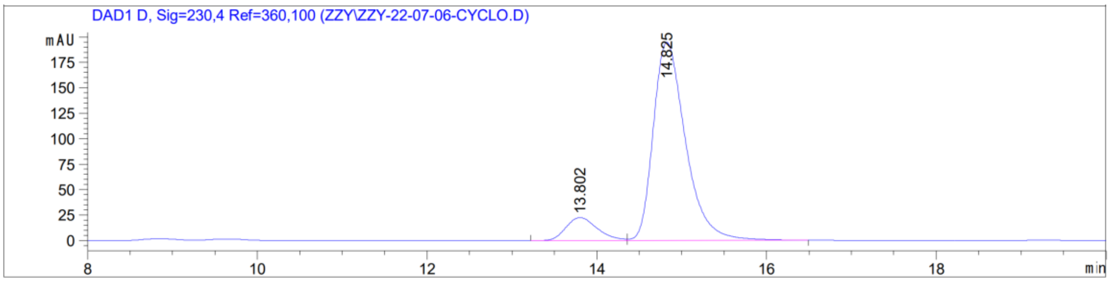

Peak #	RetTime [min]	Type	Width [min]	Area [mAU*s]	Height [mAU]	Area %
1	13.803	BV	0.4035	3850.70117	149.99466	10.1567
2	14.825	VB	0.4164	3.40622e4	1264.02454	89.8433

(*S*)-2-(dimethyl(phenyl)silyl)-2-phenethylaziridine [(*S*)-**3e'a**]

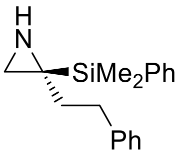

(*S*)-**3e'a**

rac-**3e'a**

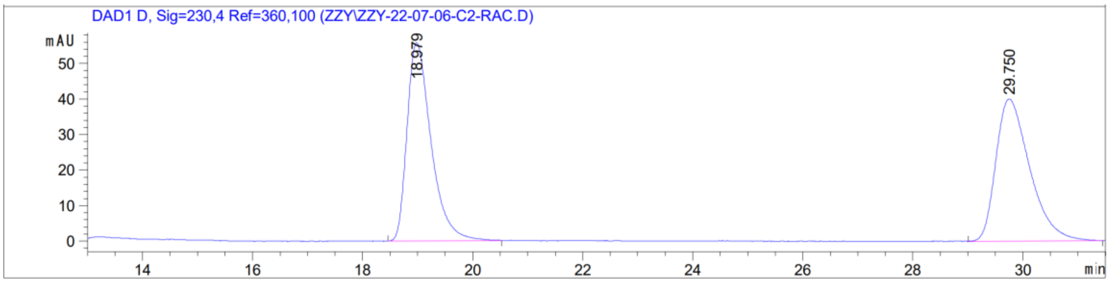

Peak #	RetTime [min]	Type	Width [min]	Area [mAU*s]	Height [mAU]	Area %
1	18.979	BB	0.4579	1692.12073	55.77555	49.7531
2	29.750	BB	0.6305	1708.91345	39.99509	50.2469

(*S*)-**3e'a** (87% *ee*)

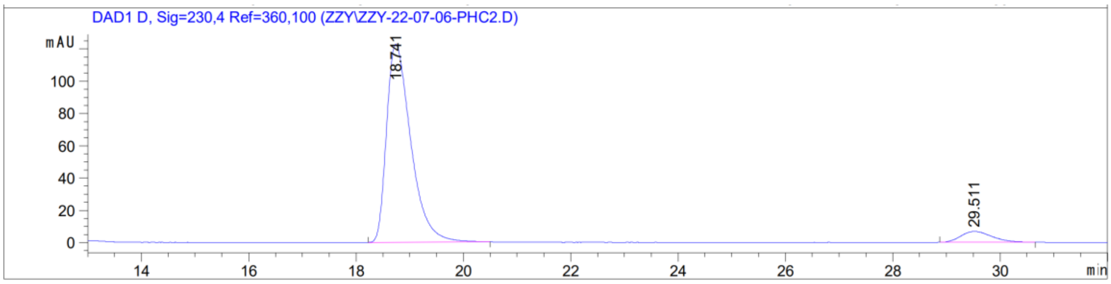

Peak #	RetTime [min]	Type	Width [min]	Area [mAU*s]	Height [mAU]	Area %
1	18.741	BB	0.4836	3850.70752	122.86555	93.3224
2	29.511	BB	0.4898	275.53406	6.75682	6.6776

(*S*)-2-(dimethyl(phenyl)silyl)-2-(3-phenylpropyl)aziridine [(*S*)-**3f'a**]

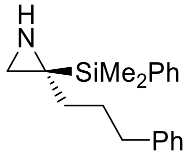

(*S*)-**3f'a**

rac-**3f'a**

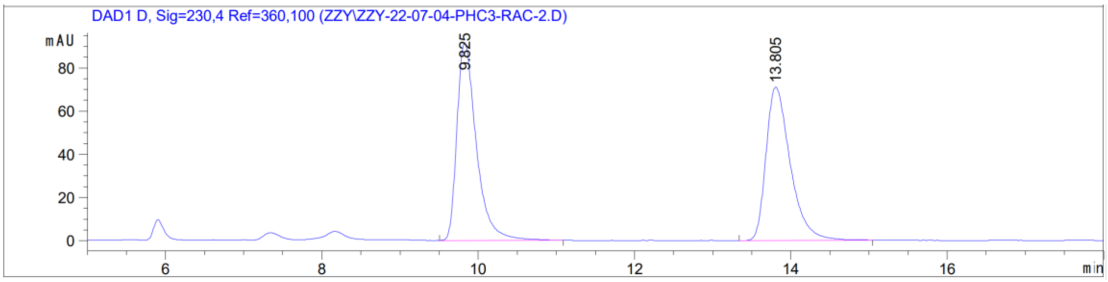

Peak #	RetTime [min]	Type	Width [min]	Area [mAU*s]	Height [mAU]	Area %
1	9.825	BB	0.2630	1577.88953	91.60751	50.7361
2	13.805	BB	0.3255	1532.10706	71.07732	49.2639

(*S*)-**3f'a** (87% *ee*)

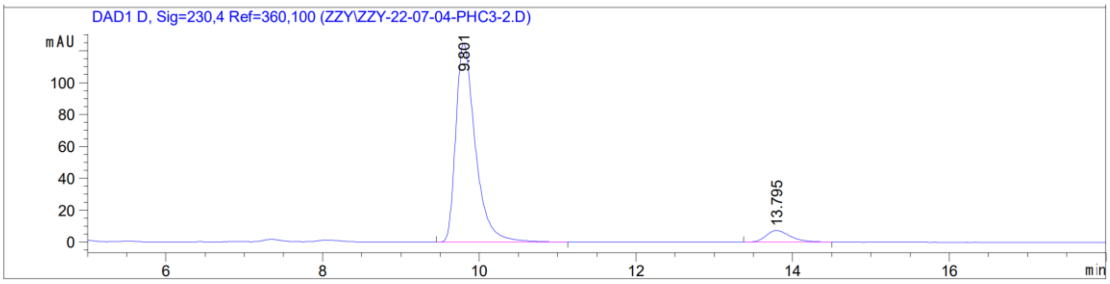

Peak #	RetTime [min]	Type	Width [min]	Area [mAU*s]	Height [mAU]	Area %
1	9.801	BB	0.2644	2159.09546	124.42641	93.3097
2	13.795	BB	0.3164	154.80609	7.27102	6.6903

(*S*)-2-(4-chlorobutyl)-2-(dimethyl(phenyl)silyl)aziridine [(*S*)-**3g'a**]

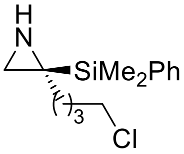

(*S*)-**3g'a**

rac-**3g'a**

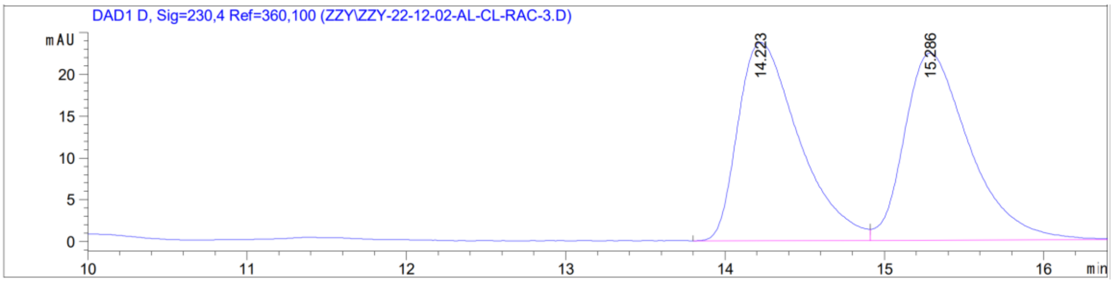

Peak #	RetTime [min]	Type	Width [min]	Area [mAU*s]	Height [mAU]	Area %
1	14.223	BV	0.4050	632.42426	23.72561	49.8220
2	15.286	VB	0.4164	636.94232	22.35804	50.1780

(*S*)-**3g'a** (85% *ee*)

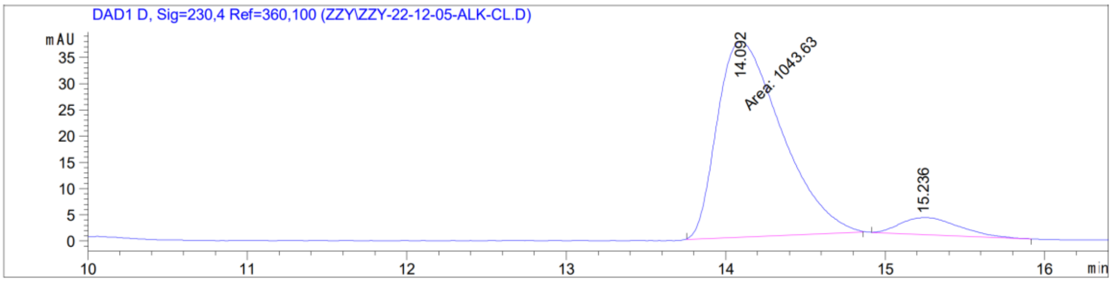

Peak #	RetTime [min]	Type	Width [min]	Area [mAU*s]	Height [mAU]	Area %
1	14.092	MM	0.4674	1043.62659	37.21225	92.7014
2	15.236	BB	0.3370	82.16715	3.18507	7.2986

(*S*)-2-phenyl-2-(triethylsilyl)aziridine [(*S*)-**3ab**]

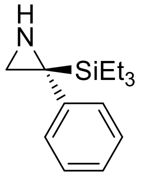

(*S*)-**3ab**

rac-**3ab**

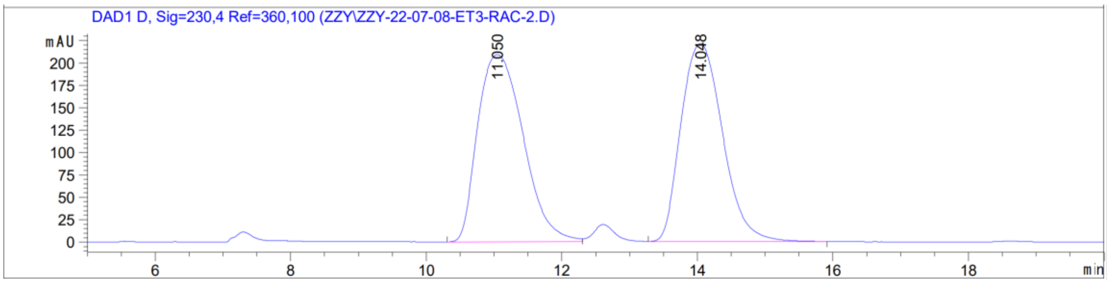

Peak #	RetTime [min]	Type	Width [min]	Area [mAU*s]	Height [mAU]	Area %
1	11.050	BV	0.7634	9767.78125	208.93712	50.8550
2	14.048	BB	0.6864	9439.34375	220.97937	49.1450

(*S*)-**3ab** (64% *ee*)

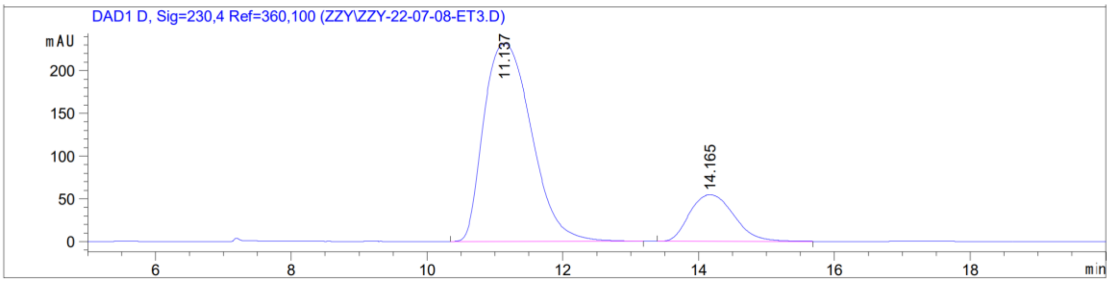

Peak #	RetTime [min]	Type	Width [min]	Area [mAU*s]	Height [mAU]	Area %
1	11.137	BB	0.7920	1.12998e4	232.09416	82.1378
2	14.165	BB	0.7218	2457.31519	54.52300	17.8622

(*S*)-5-(dimethyl(phenyl)silyl)-2-(3,5-dinitrophenyl)-5-phenyl-4,5-dihydrooxazole [(*S*)-**7**]

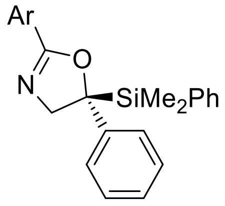

Ar = 3,5-dinitrophenyl
(*S*)-**7**

rac-**7**

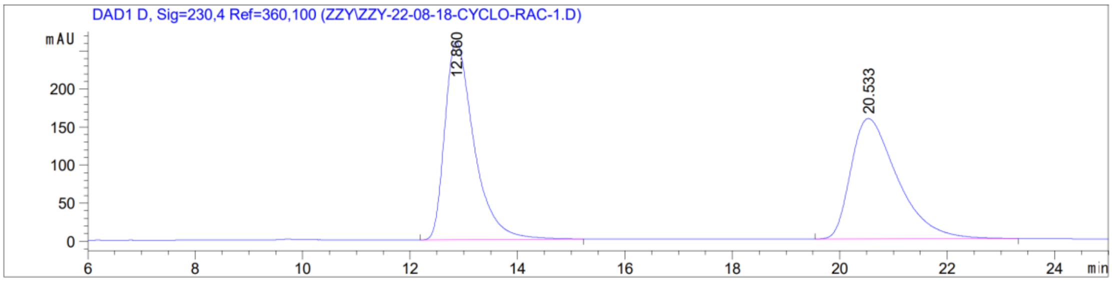

Peak #	RetTime [min]	Type	Width [min]	Area [mAU*s]	Height [mAU]	Area %
1	12.860	BB	0.5658	9835.24609	260.90536	50.7365
2	20.533	BB	0.8757	9549.69336	158.32585	49.2635

(*S*)-**7** (94% *ee*)

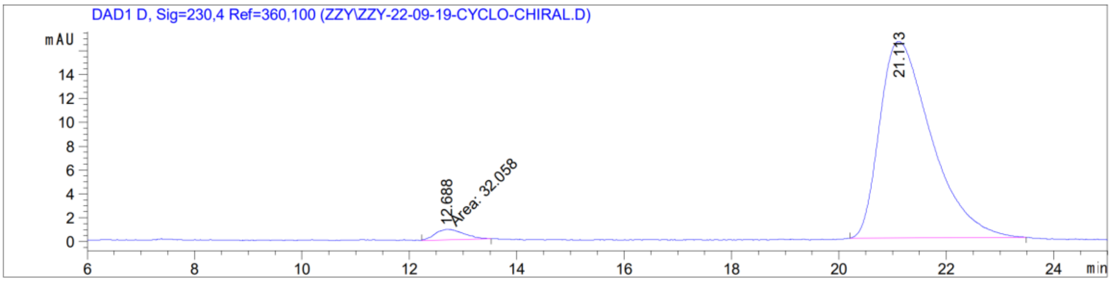

Peak #	RetTime [min]	Type	Width [min]	Area [mAU*s]	Height [mAU]	Area %
1	12.688	MM	0.6103	32.05803	8.75519e-1	2.8199
2	21.113	BB	0.9413	1104.79968	16.49762	97.1801

(*S*)-2-(dimethyl(phenyl)silyl)-2-phenyl-1-tosylaziridine [(*S*)-**8**]

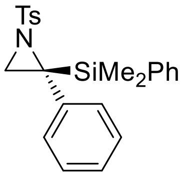

(*S*)-**8**

rac-**8**

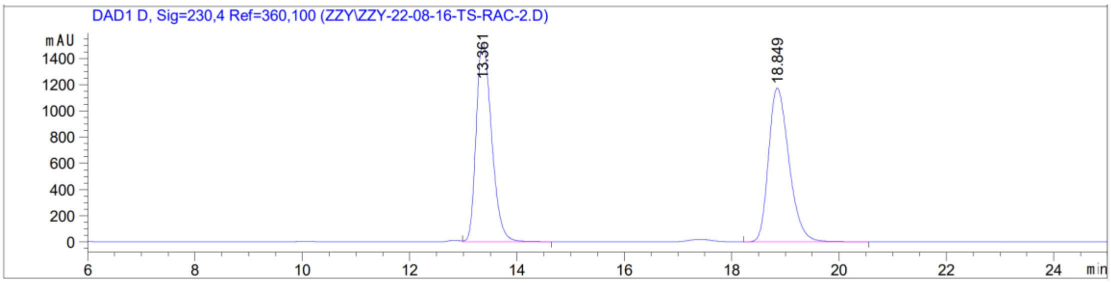

Peak #	RetTime [min]	Type	Width [min]	Area [mAU*s]	Height [mAU]	Area %
1	13.361	VB	0.3120	3.04917e4	1520.04993	49.5518
2	18.849	BB	0.4127	3.10432e4	1173.23779	50.4482

(*S*)-**8** (95% *ee*)

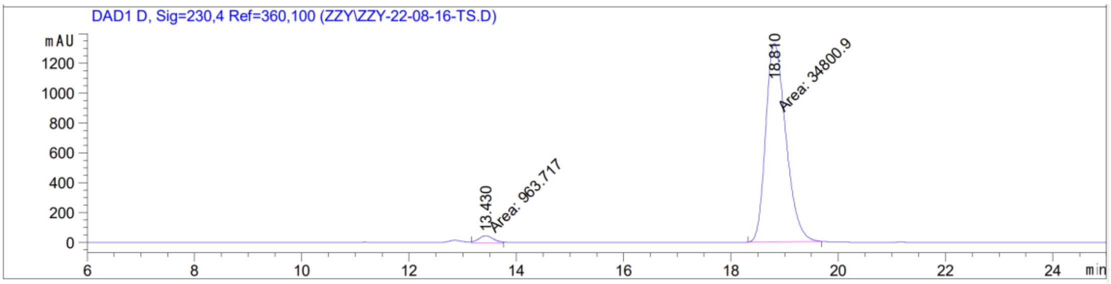

Peak #	RetTime [min]	Type	Width [min]	Area [mAU*s]	Height [mAU]	Area %
1	13.430	MM	0.3298	963.71686	48.69675	2.6946
2	18.810	MM	0.4353	3.48009e4	1332.44690	97.3054

(*S*)-N-(2-(dimethyl(phenyl)silyl)-2-phenylethyl)-4-methylbenzenesulfonamide

[(*S*)-**9**]

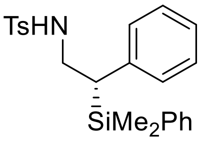

(*S*)-**9**

rac-**9**

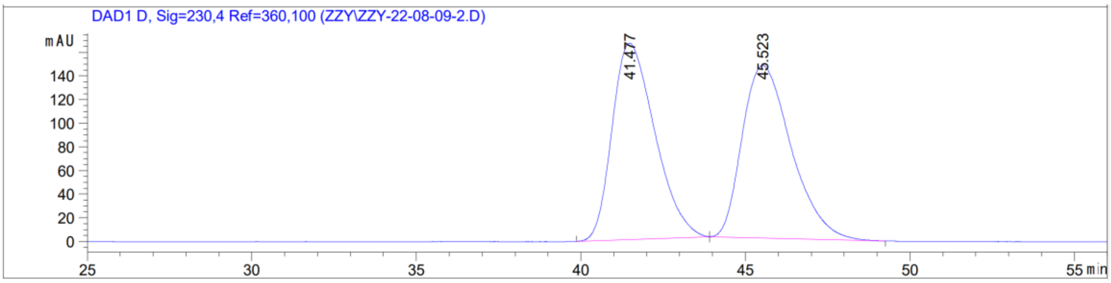

Peak #	RetTime [min]	Type	Width [min]	Area [mAU*s]	Height [mAU]	Area %
1	41.477	BB	1.2388	1.49689e4	166.19754	49.7031
2	45.523	BB	1.3751	1.51478e4	145.92471	50.2969

(*S*)-**9** (90% *ee*)

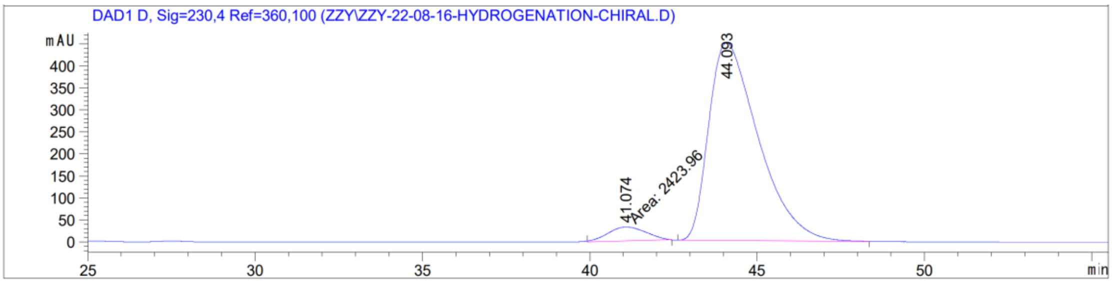

Peak #	RetTime [min]	Type	Width [min]	Area [mAU*s]	Height [mAU]	Area %
1	41.074	MM	1.2981	2423.95972	31.12283	4.9764
2	44.093	BB	1.4056	4.62849e4	450.13358	95.0236

10 NMR Spectra

Figure S3. **^{1}H NMR** (500 MHz, $CDCl_3$, 298 K) of **3-(3,4-dimethylphenyl)-2*H*-azirine** (**1f**).

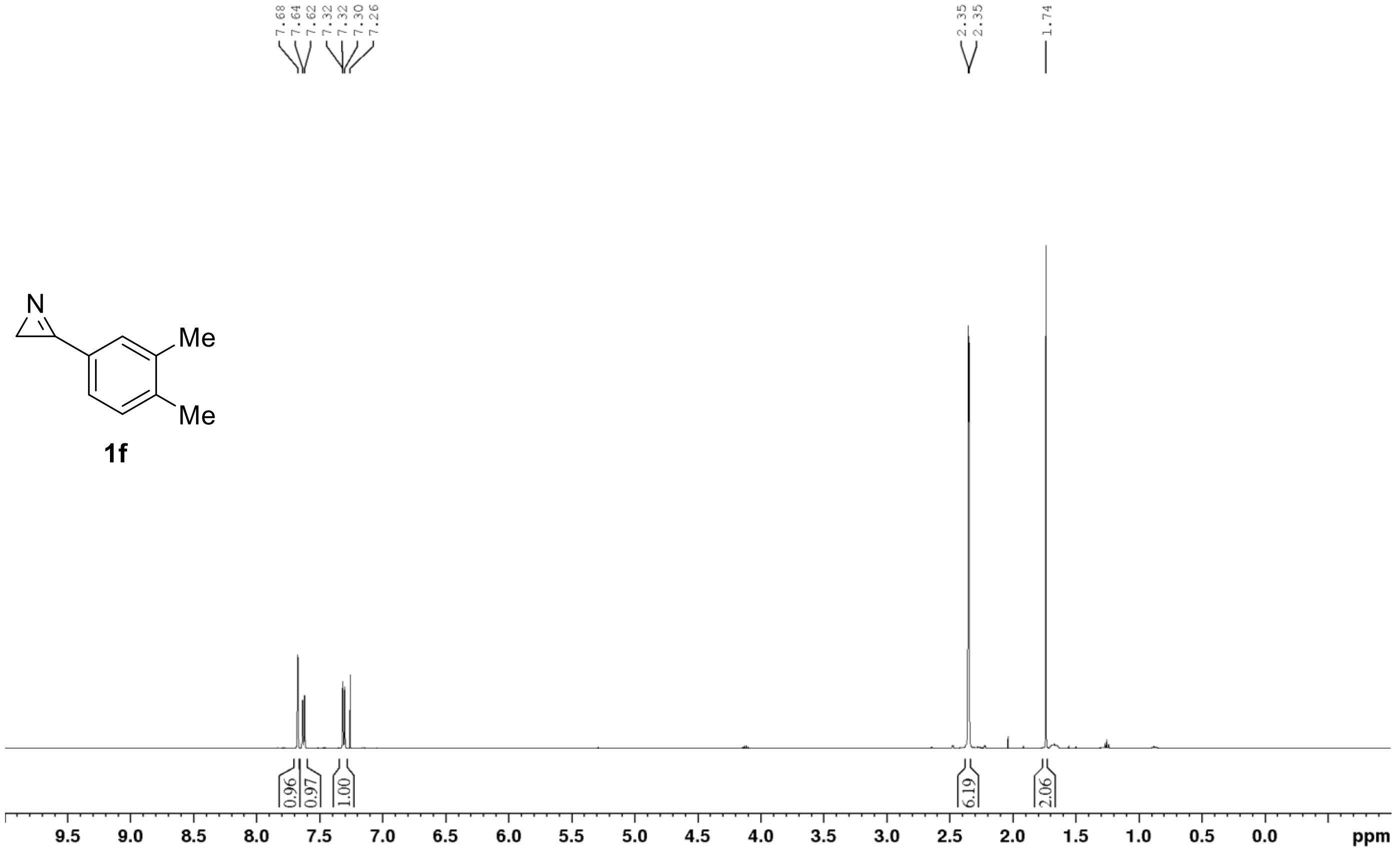

Figure S4. **^{13}C NMR** (126 MHz, $CDCl_3$, 298 K) of **3-(3,4-dimethylphenyl)-2*H*-azirine** (**1f**).

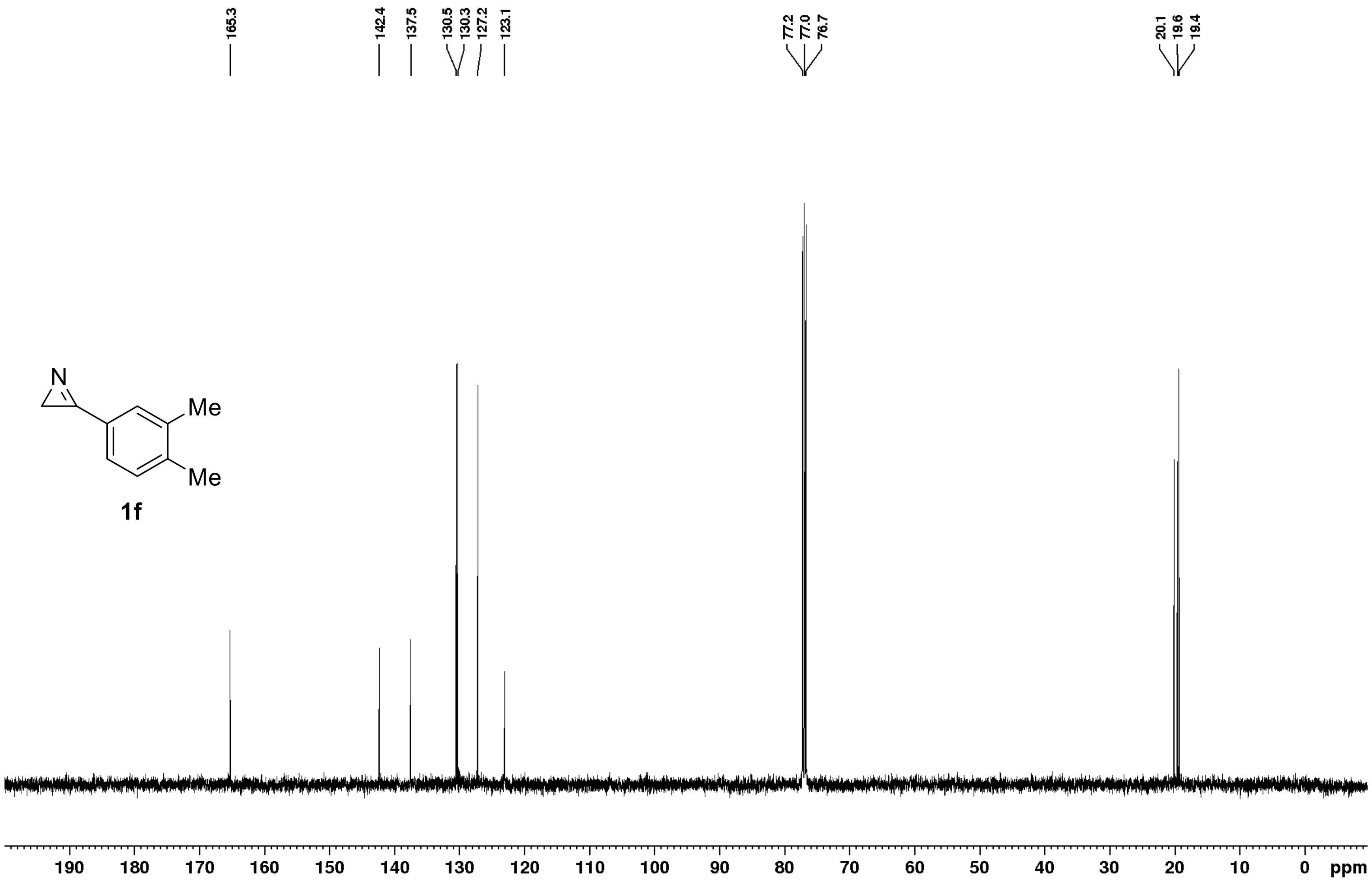

Figure S5. [1]**H NMR** (500 MHz, $CDCl_3$, 298 K) of **2-(4-(2*H*-azirin-3-yl)benzyl)isoindoline-1,3-dione** (**1g**).

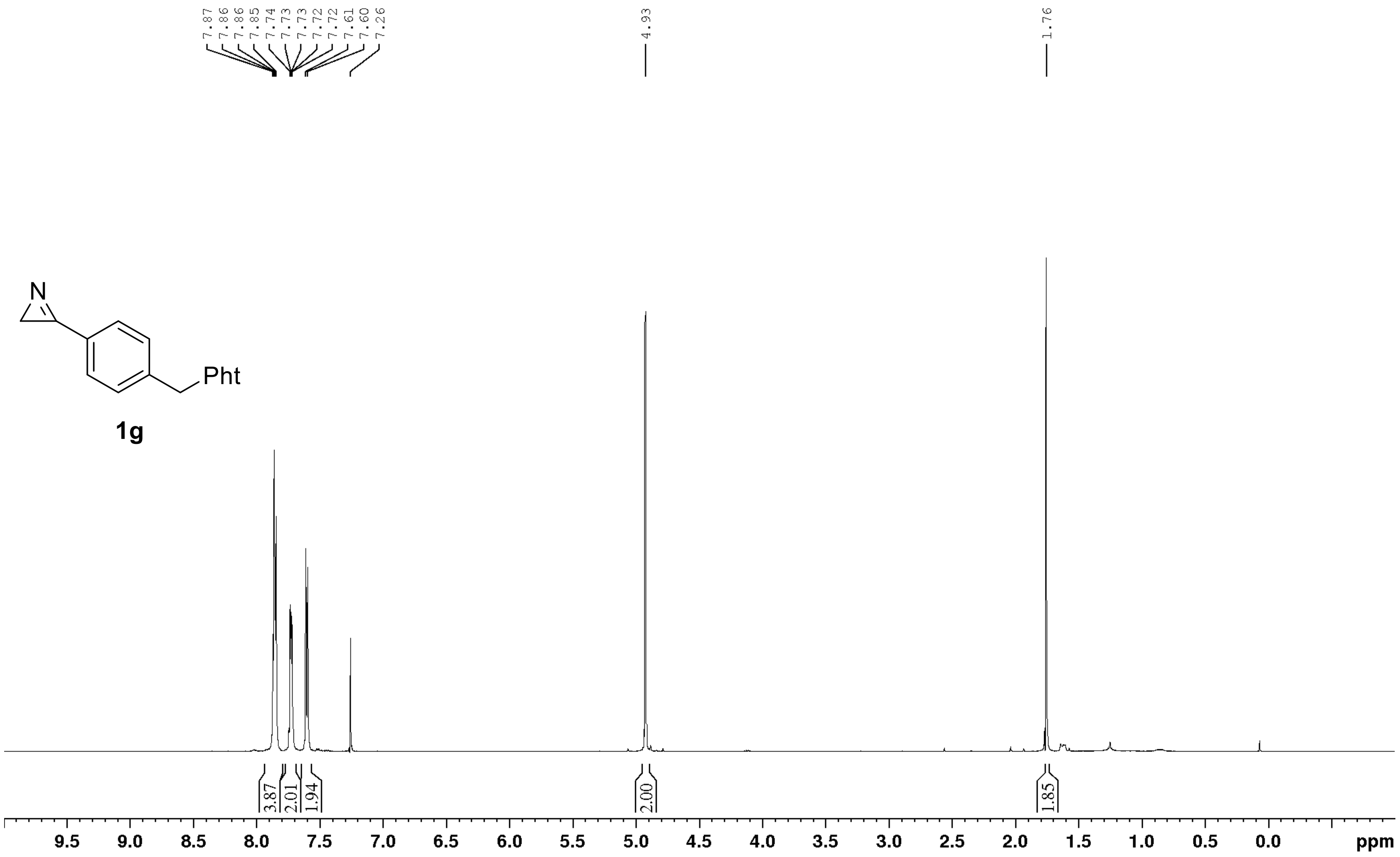

Figure S6. **^{13}C NMR** (126 MHz, $CDCl_3$, 298 K) of **2-(4-(2*H*-azirin-3-yl)benzyl)isoindoline-1,3-dione** (**1g**).

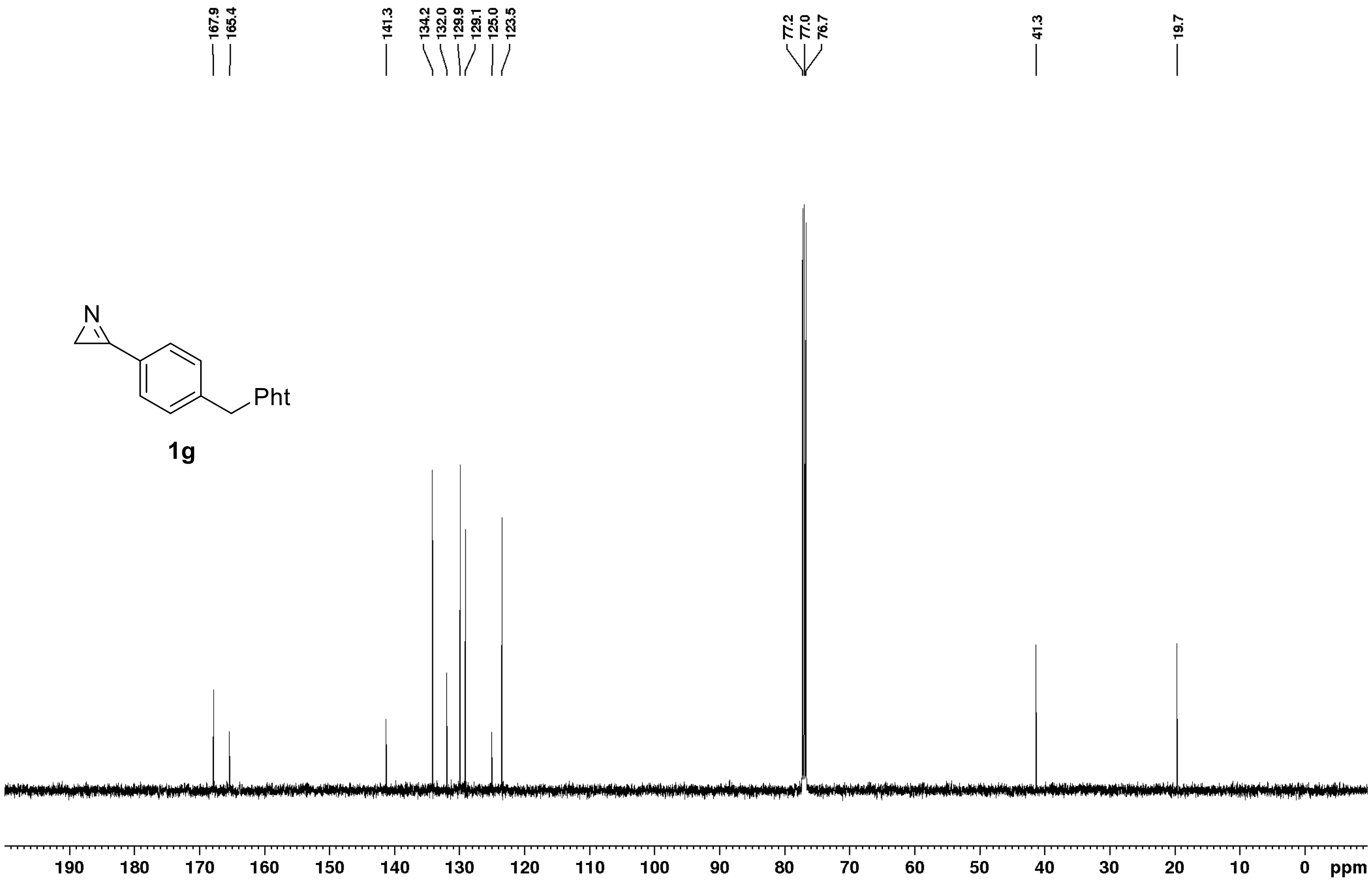

Figure S7. **^{1}H NMR** (500 MHz, $CDCl_3$, 298 K) of **3-(3-(benzyloxy)phenyl)-2*H*-azirine** (**1l**).

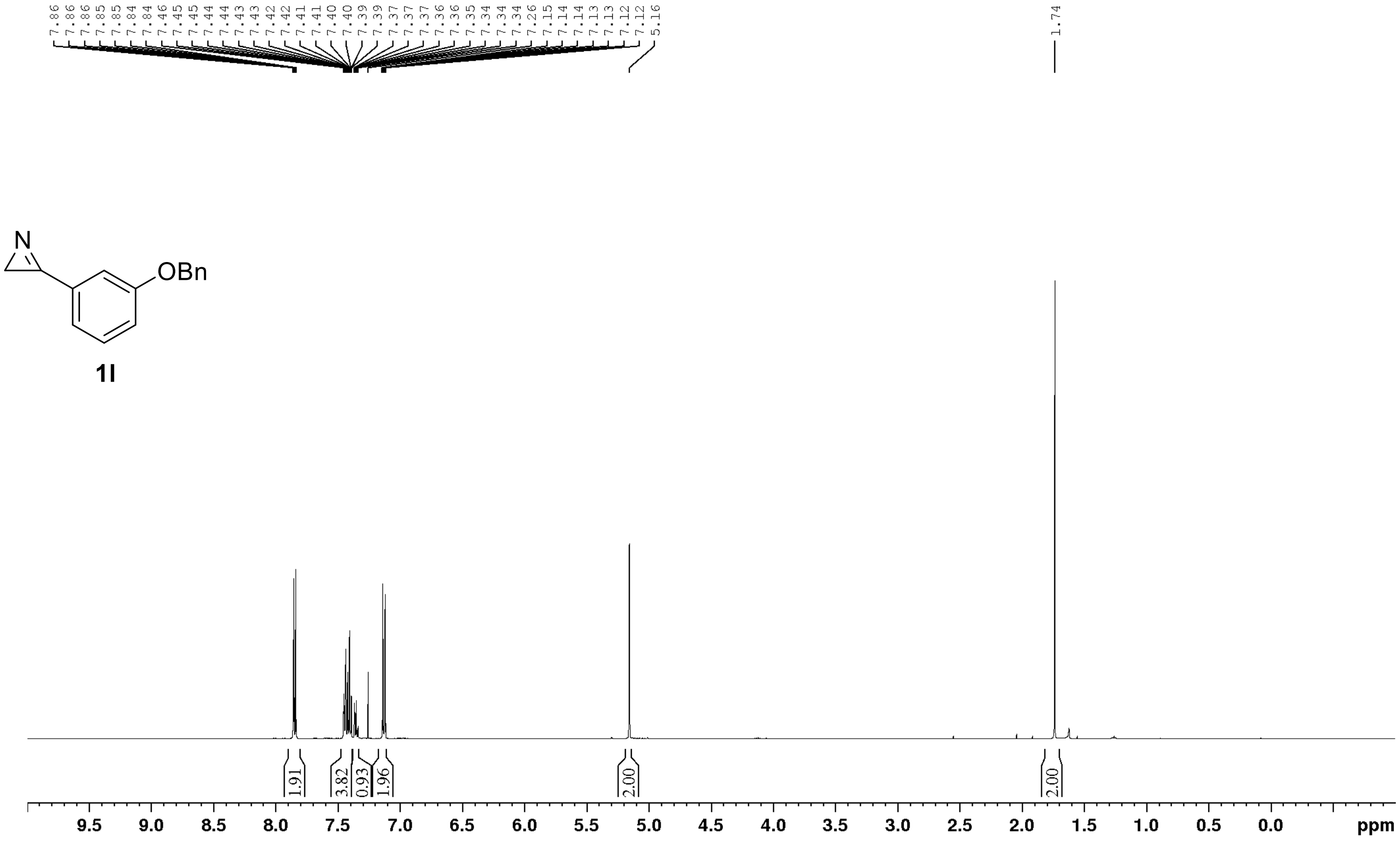

1l

Figure S8. ^{13}**C NMR** (126 MHz, $CDCl_3$, 298 K) of **3-(3-(benzyloxy)phenyl)-2*H*-azirine** (**1l**).

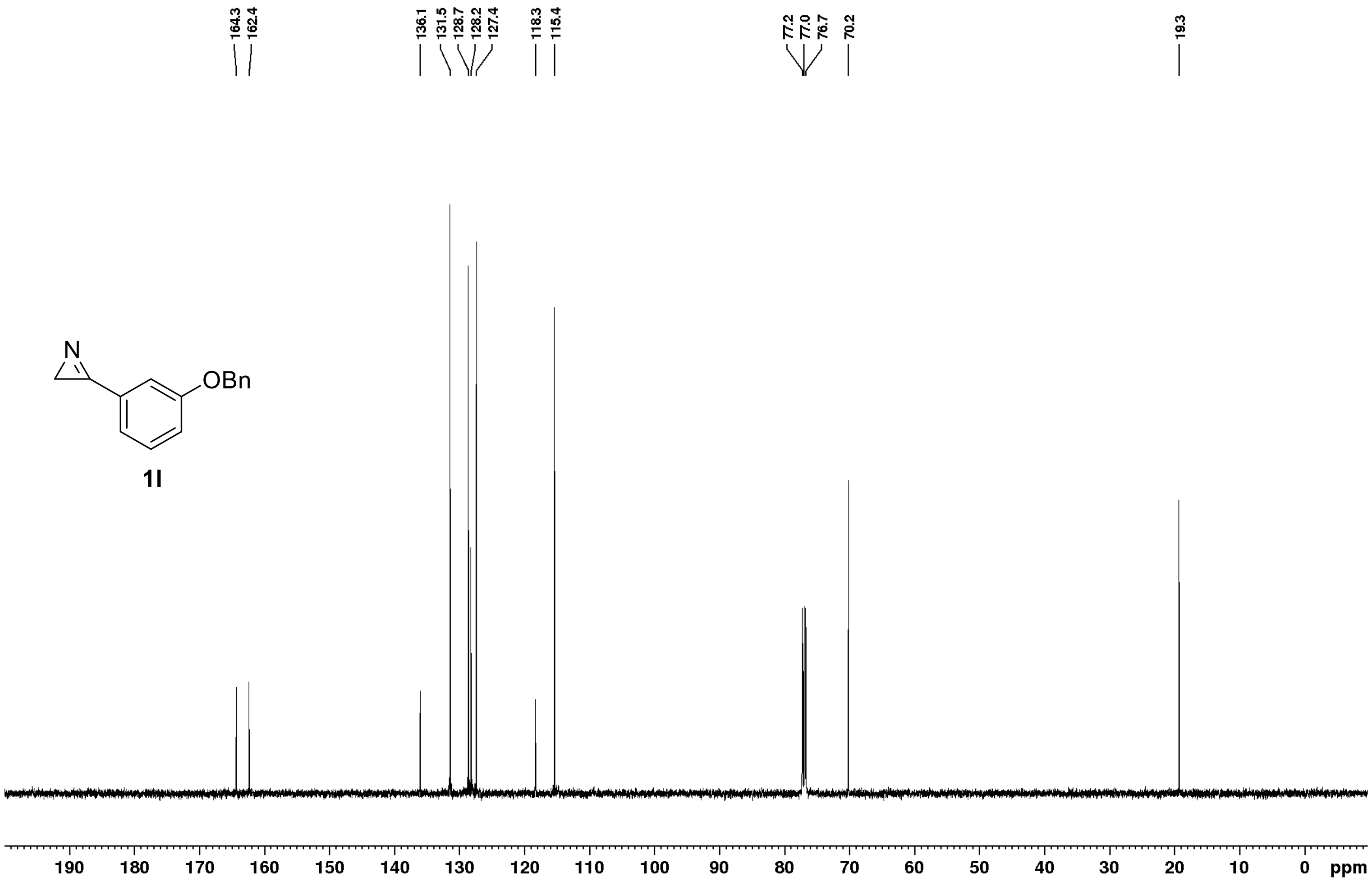

Figure S9. **^{1}H NMR** (500 MHz, $CDCl_3$, 298 K) of **3-(4-(benzyloxy)phenyl)-2*H*-azirine** (**1m**).

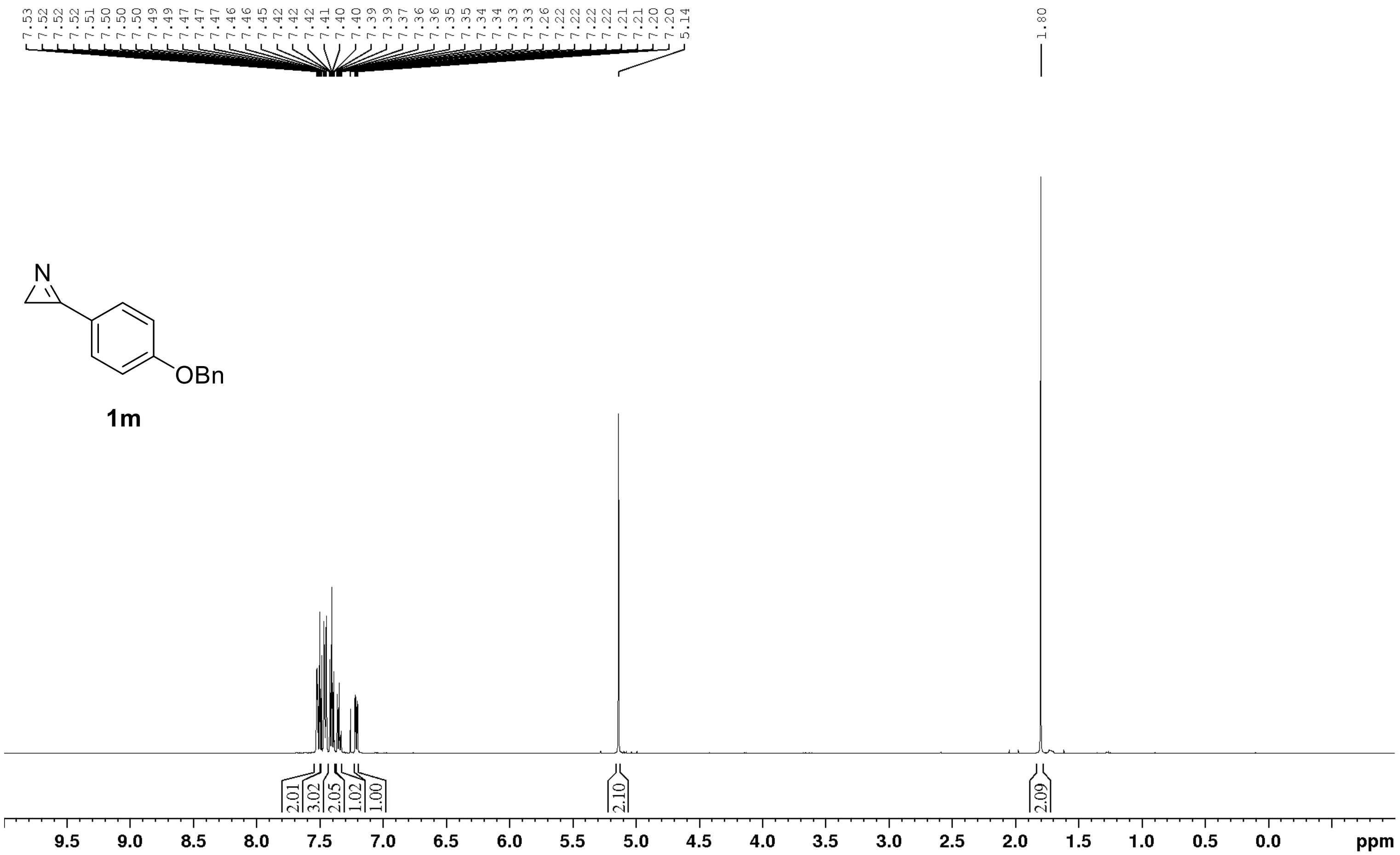

Figure S10. **^{13}C NMR** (126 MHz, $CDCl_3$, 298 K) of **3-(4-(benzyloxy)phenyl)-2*H*-azirine** (**1m**).

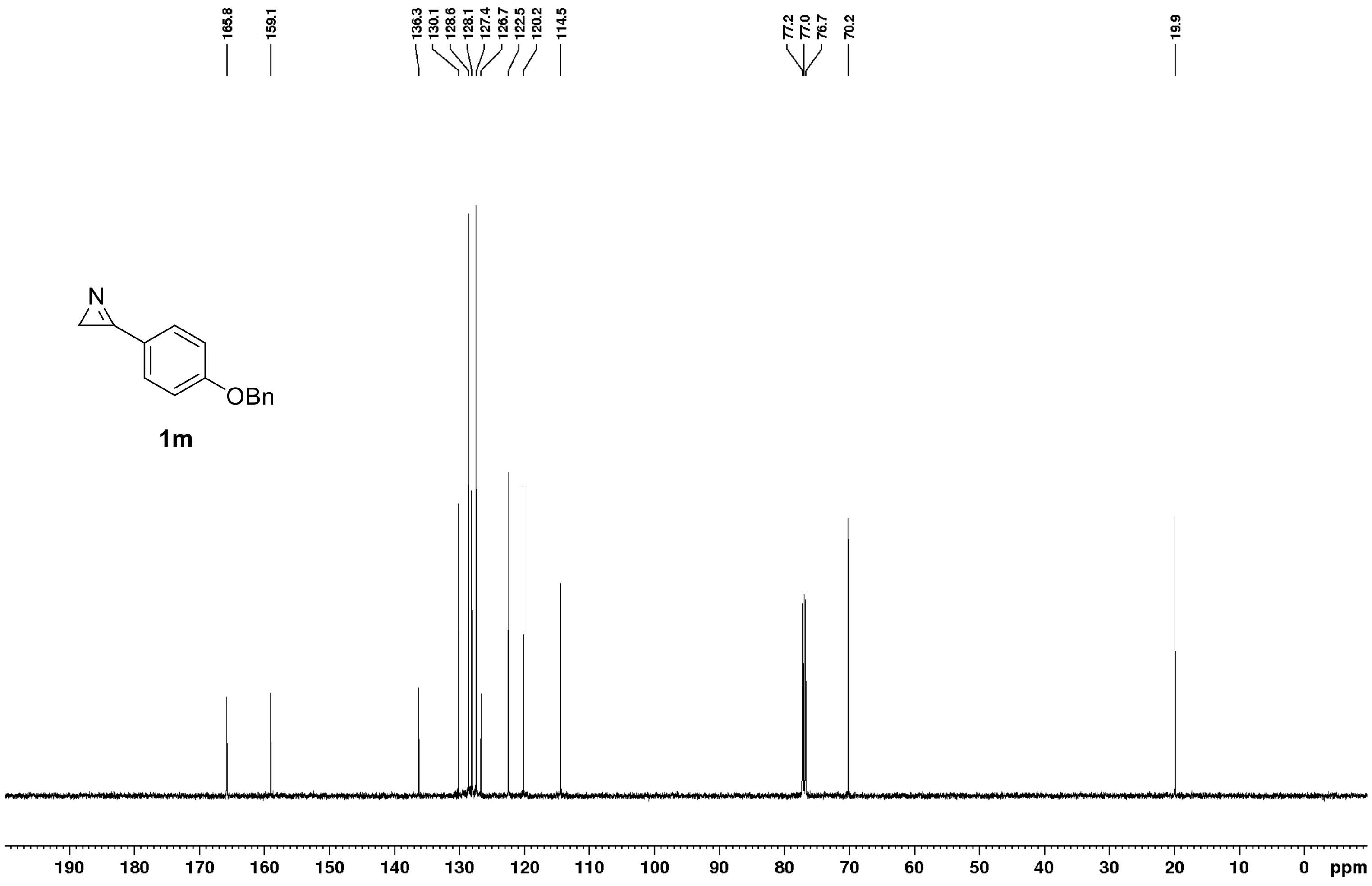

Figure S11. **^{1}H NMR** (500 MHz, $CDCl_3$, 298 K) of **3-(benzo[d][1,3]dioxol-5-yl)-2*H*-azirine** (**1n**).

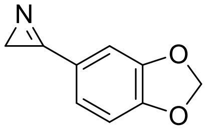

1n

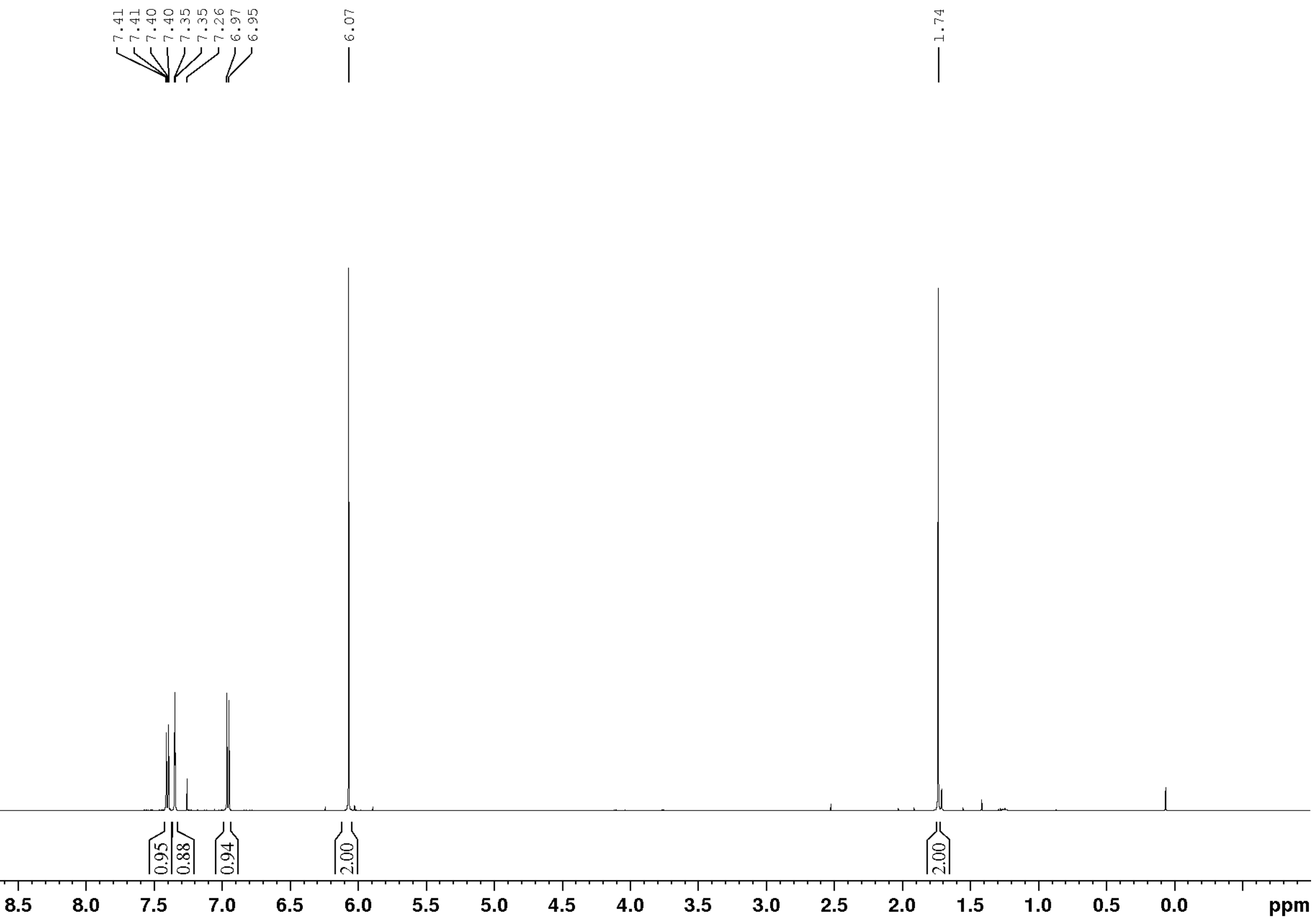

Figure S12. ^{13}**C NMR** (126 MHz, $CDCl_3$, 298 K) of **3-(benzo[d][1,3]dioxol-5-yl)-2*H*-azirine** (**1n**).

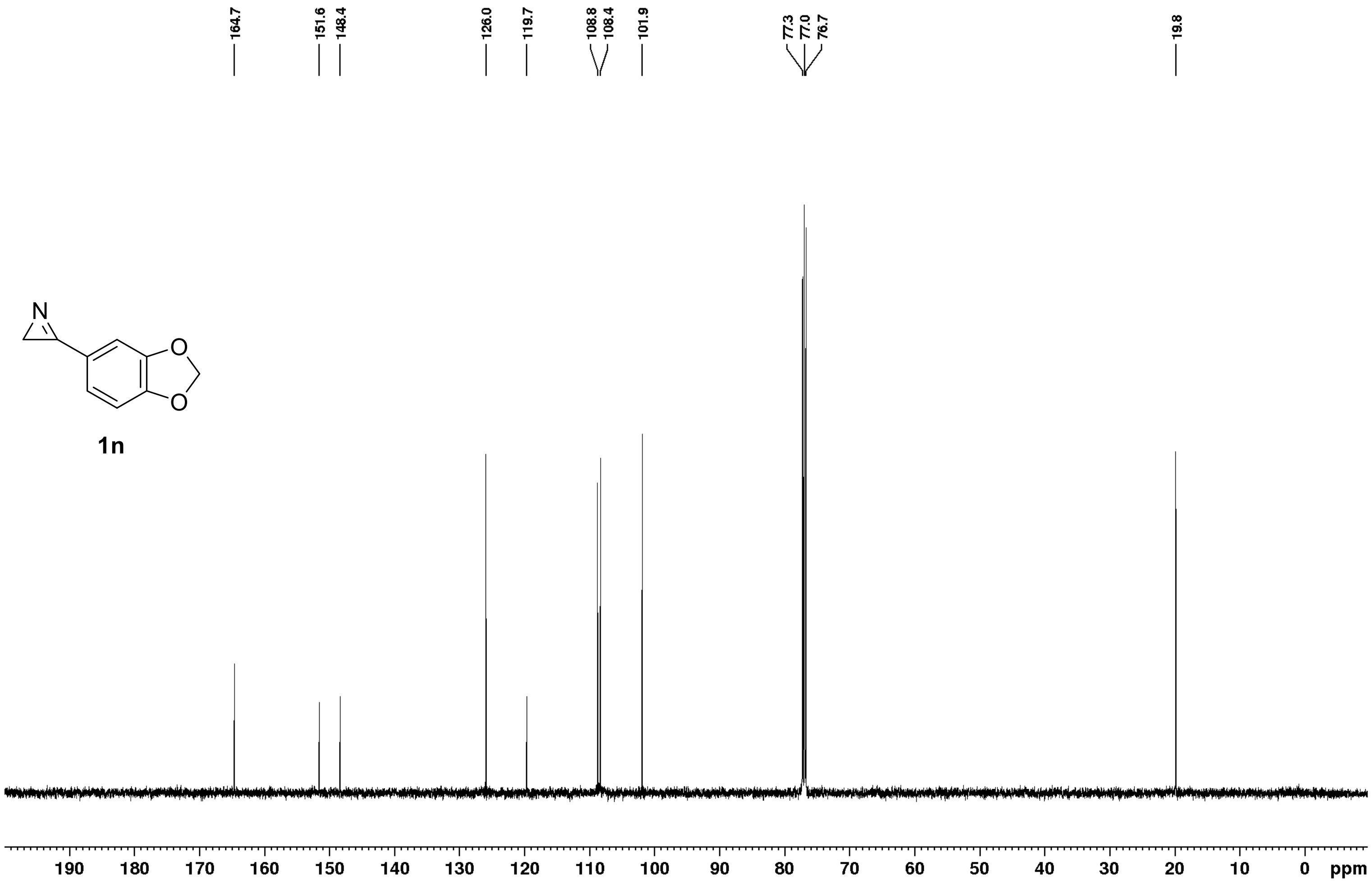

Figure S13. **^{1}H NMR** (500 MHz, $CDCl_3$, 298 K) of **1-(4-(2*H*-azirin-3-yl)phenyl)ethan-1-one** (**1t**).

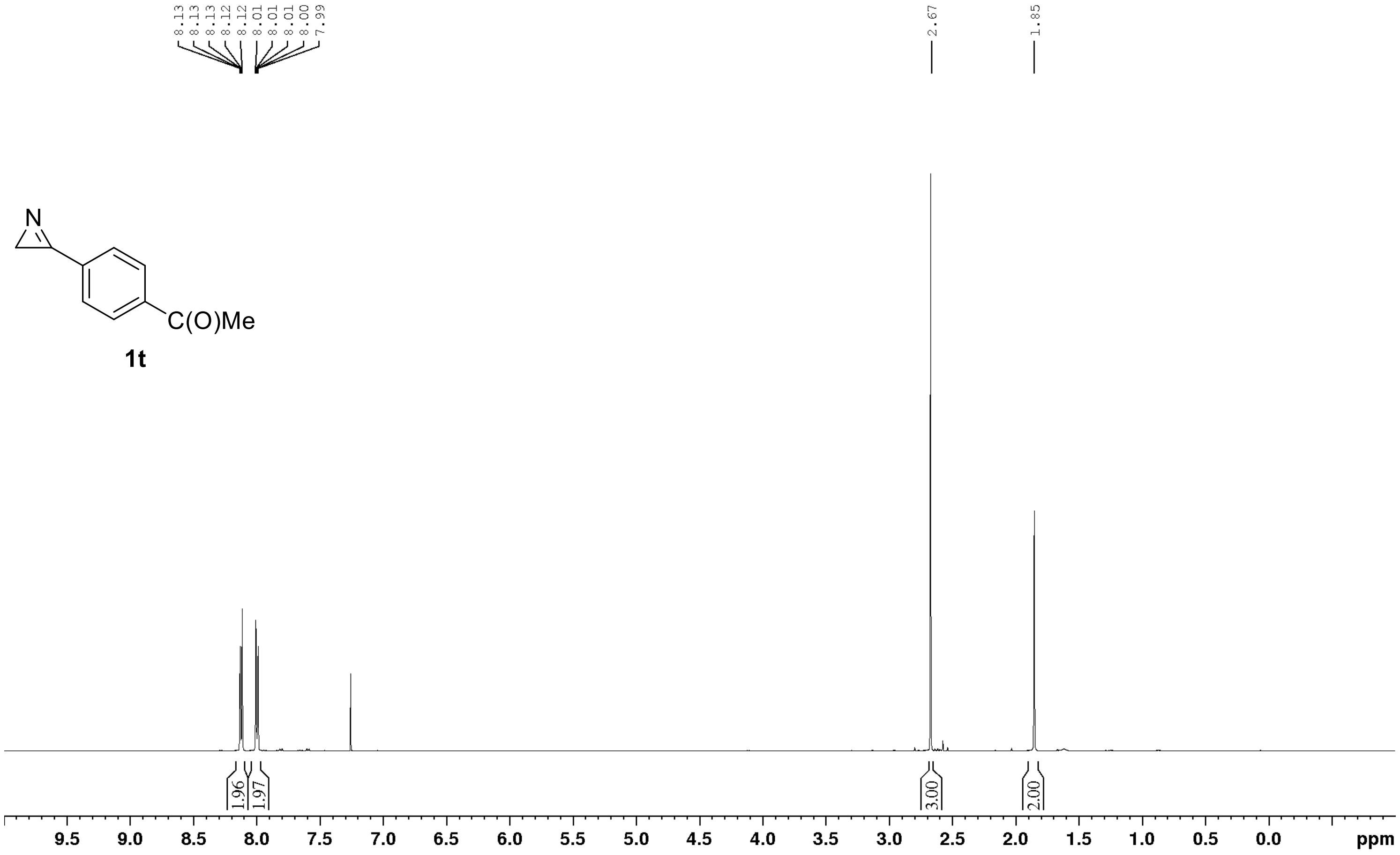

Figure S14. **^{13}C NMR** (126 MHz, $CDCl_3$, 298 K) of **1-(4-(2*H*-azirin-3-yl)phenyl)ethan-1-one** (**1t**).

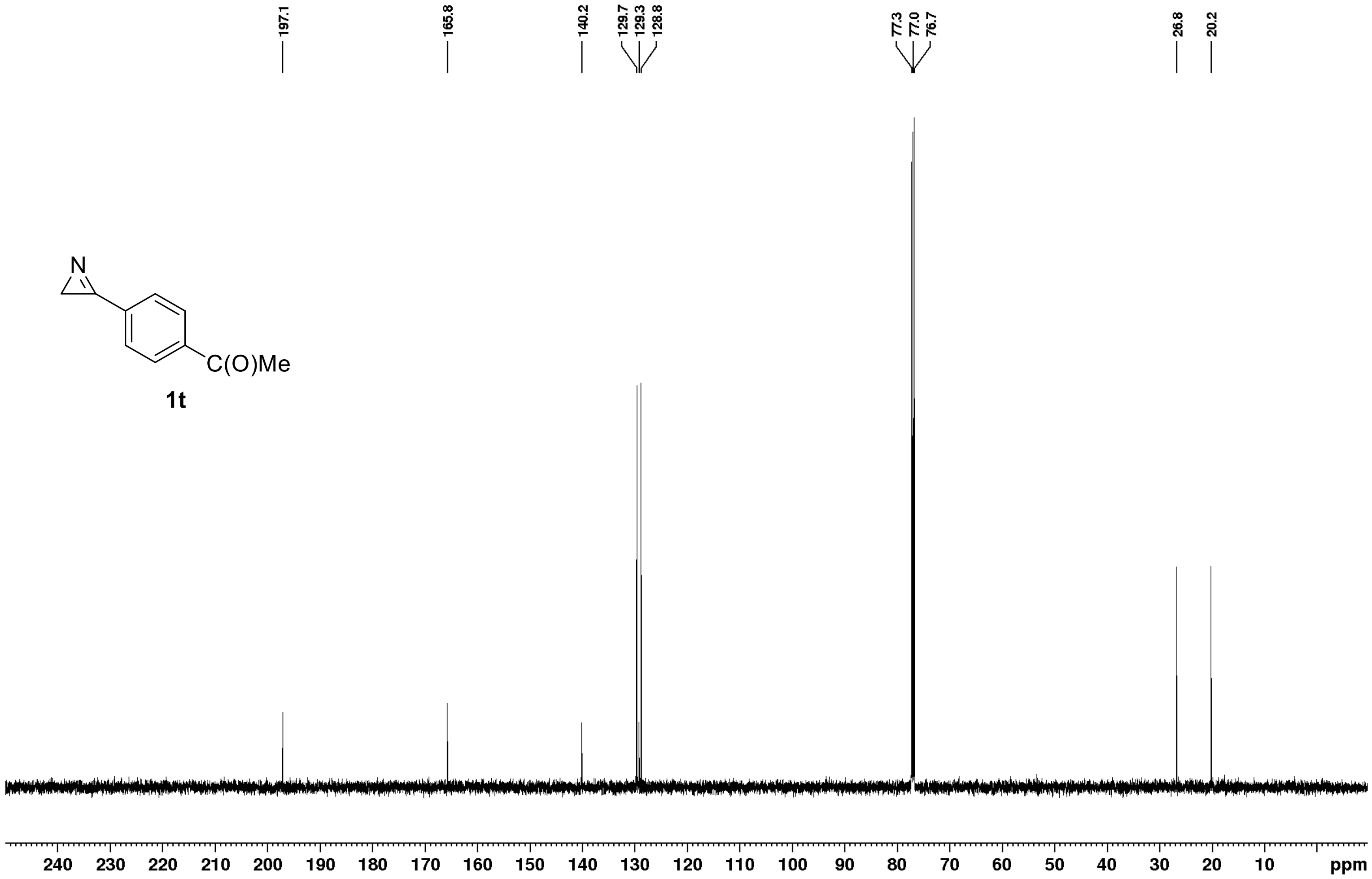

Figure S15. **^{1}H NMR** (500 MHz, $CDCl_3$, 298 K) of **3-(2,4-difluorophenyl)-2*H*-azirine** (**1a'**).

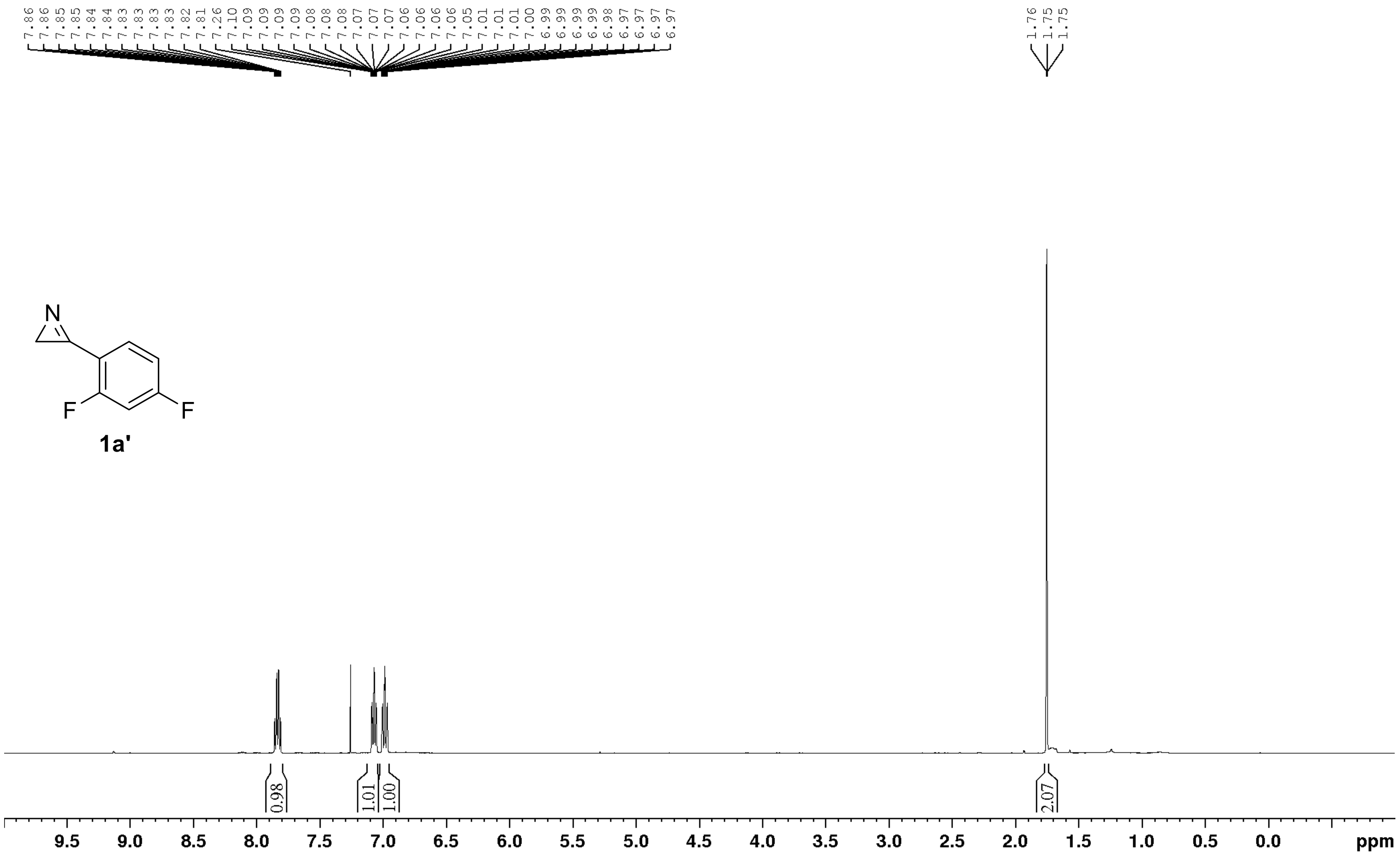

Figure S16. **^{13}C NMR** (126 MHz, $CDCl_3$, 298 K) of **3-(2,4-difluorophenyl)-2*H*-azirine** (**1a'**).

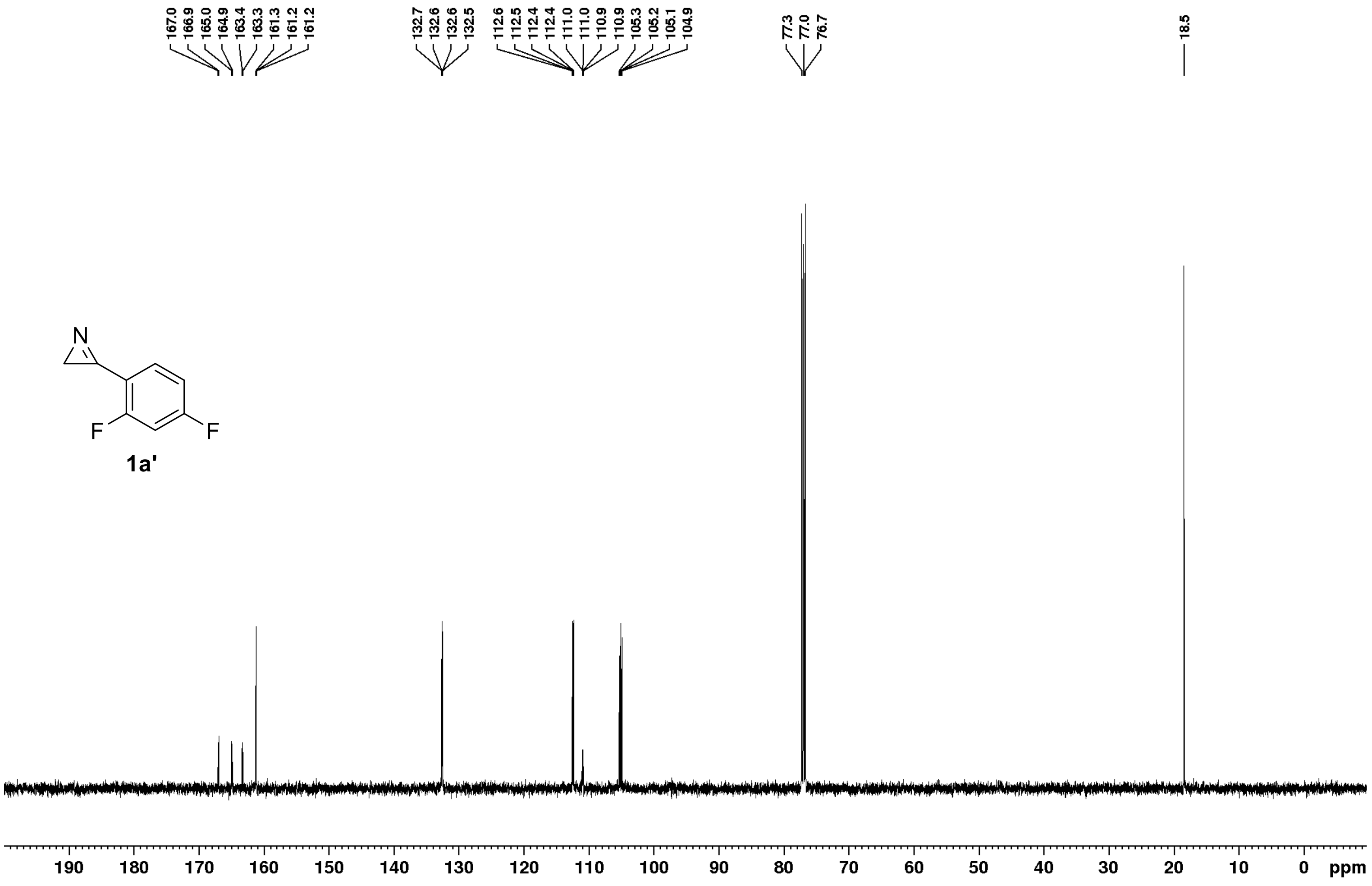

Figure S17. **^{19}F NMR** spectrum (471 MHz, $CDCl_3$, 298 K) of **3-(2,4-difluorophenyl)-2*H*-azirine** (**1a'**).

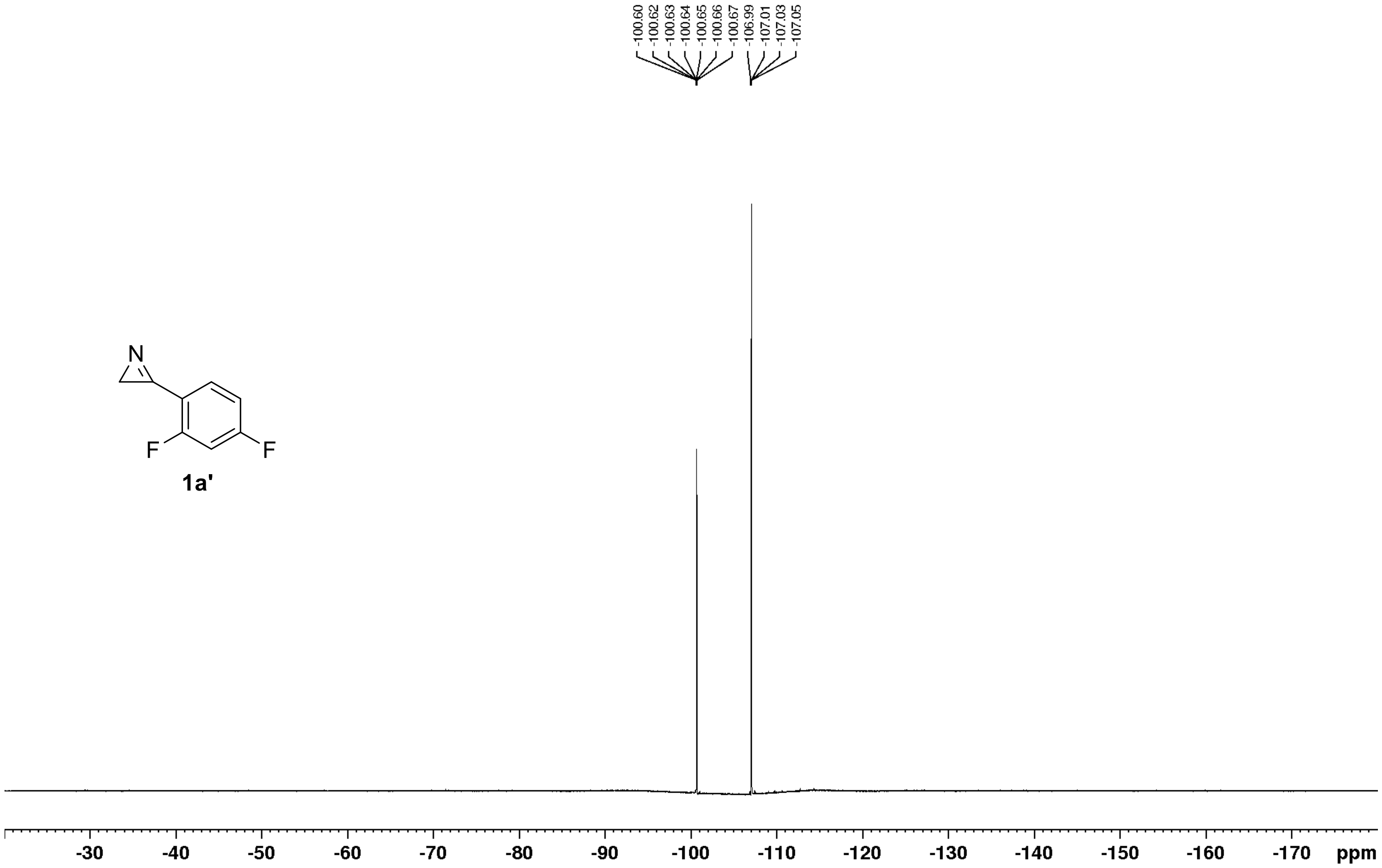

Figure S18. **^{1}H NMR** (500 MHz, $CDCl_3$, 298 K) of **3-(2-chloro-4-fluorophenyl)-2*H*-azirine** (**1b'**).

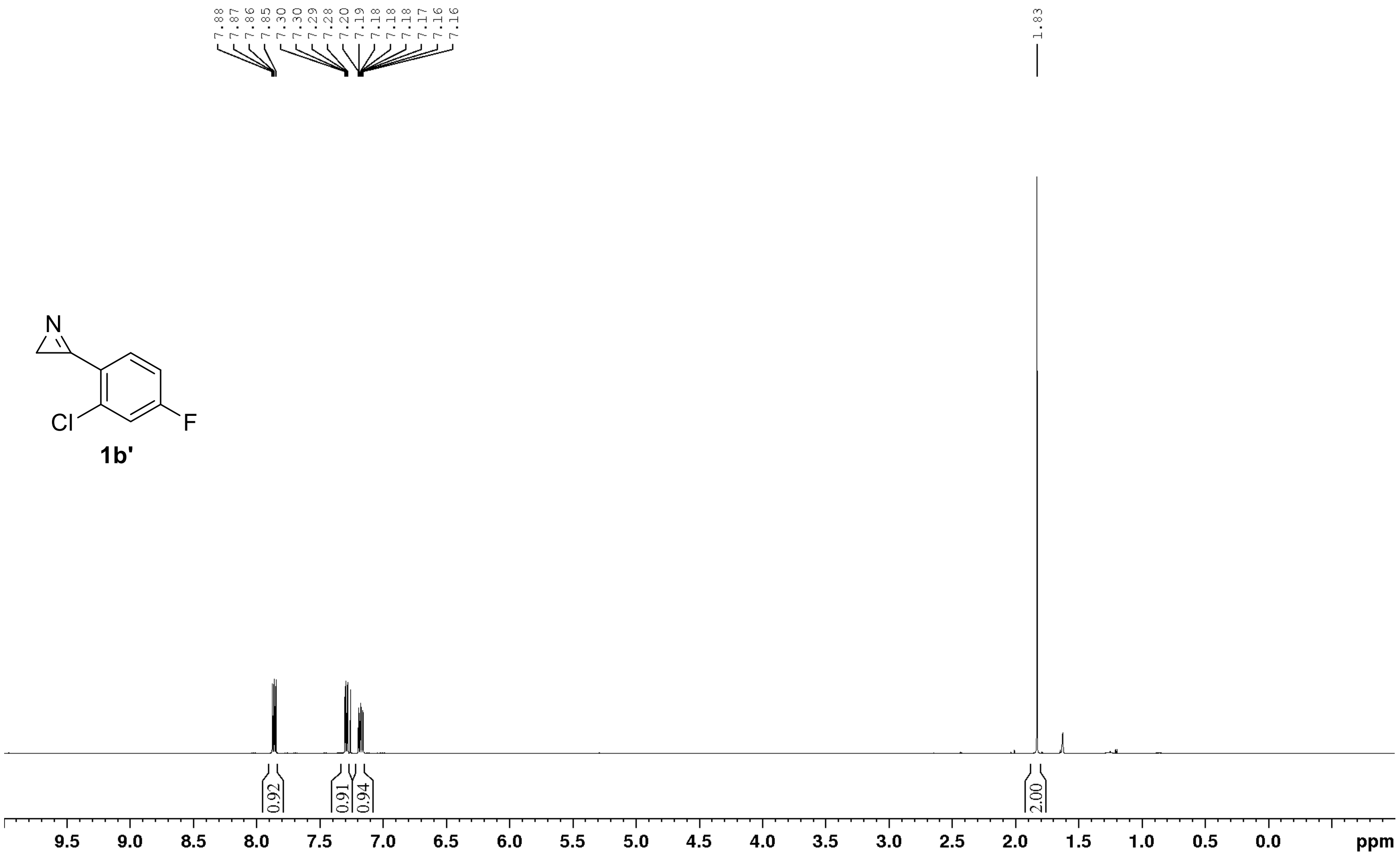

Figure S19. ^{13}C **NMR** (126 MHz, $CDCl_3$, 298 K) of **3-(2-chloro-4-fluorophenyl)-2*H*-azirine** (**1b'**).

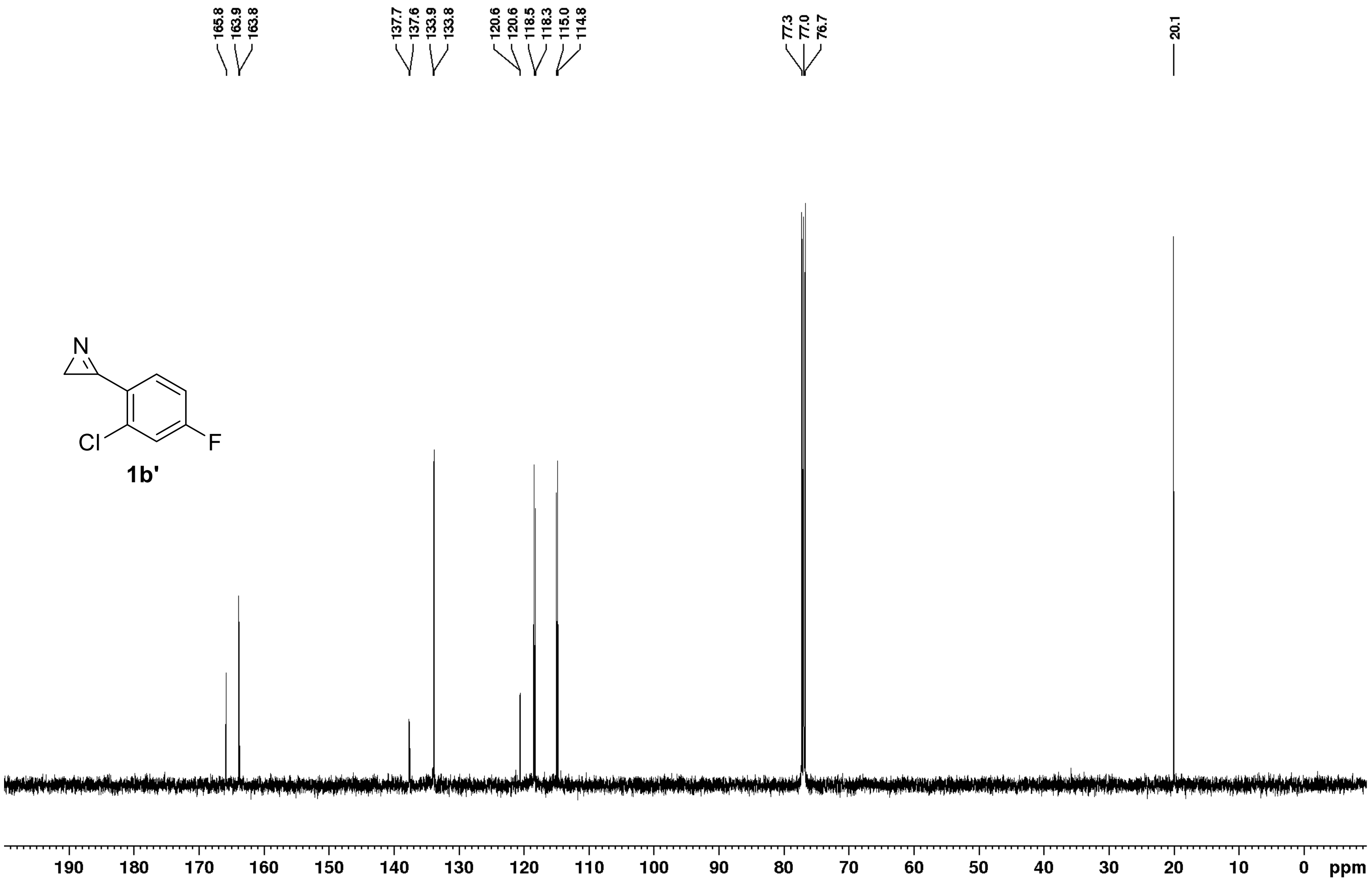

Figure S20. **^{19}F NMR** spectrum (471 MHz, $CDCl_3$, 298 K) of **3-(2-chloro-4-fluorophenyl)-2*H*-azirine** (**1b'**).

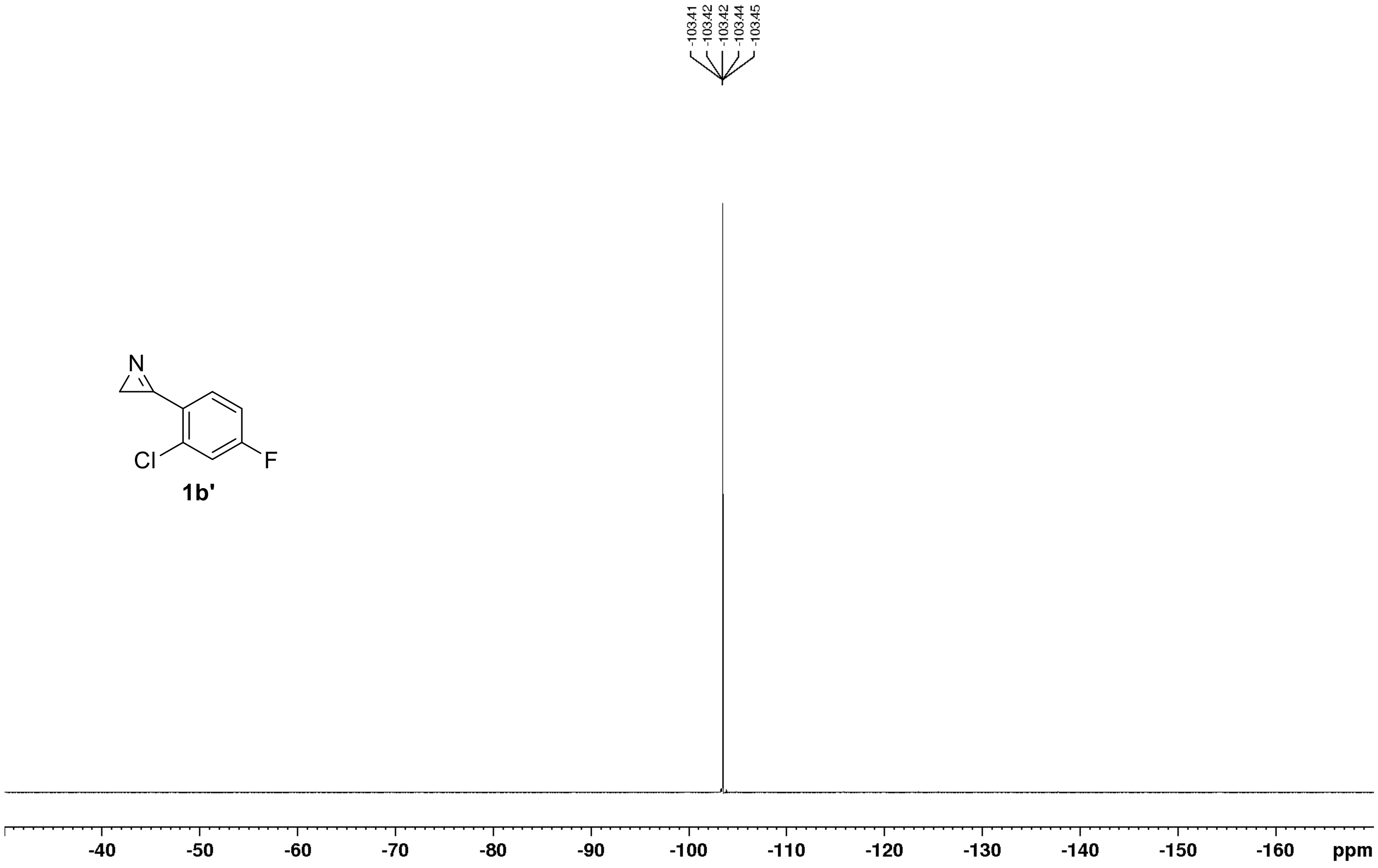

Figure S21. **¹H NMR** (500 MHz, CDCl$_3$, 298 K) of **3-cyclohexyl-*2H*-azirine** (**1d'**).

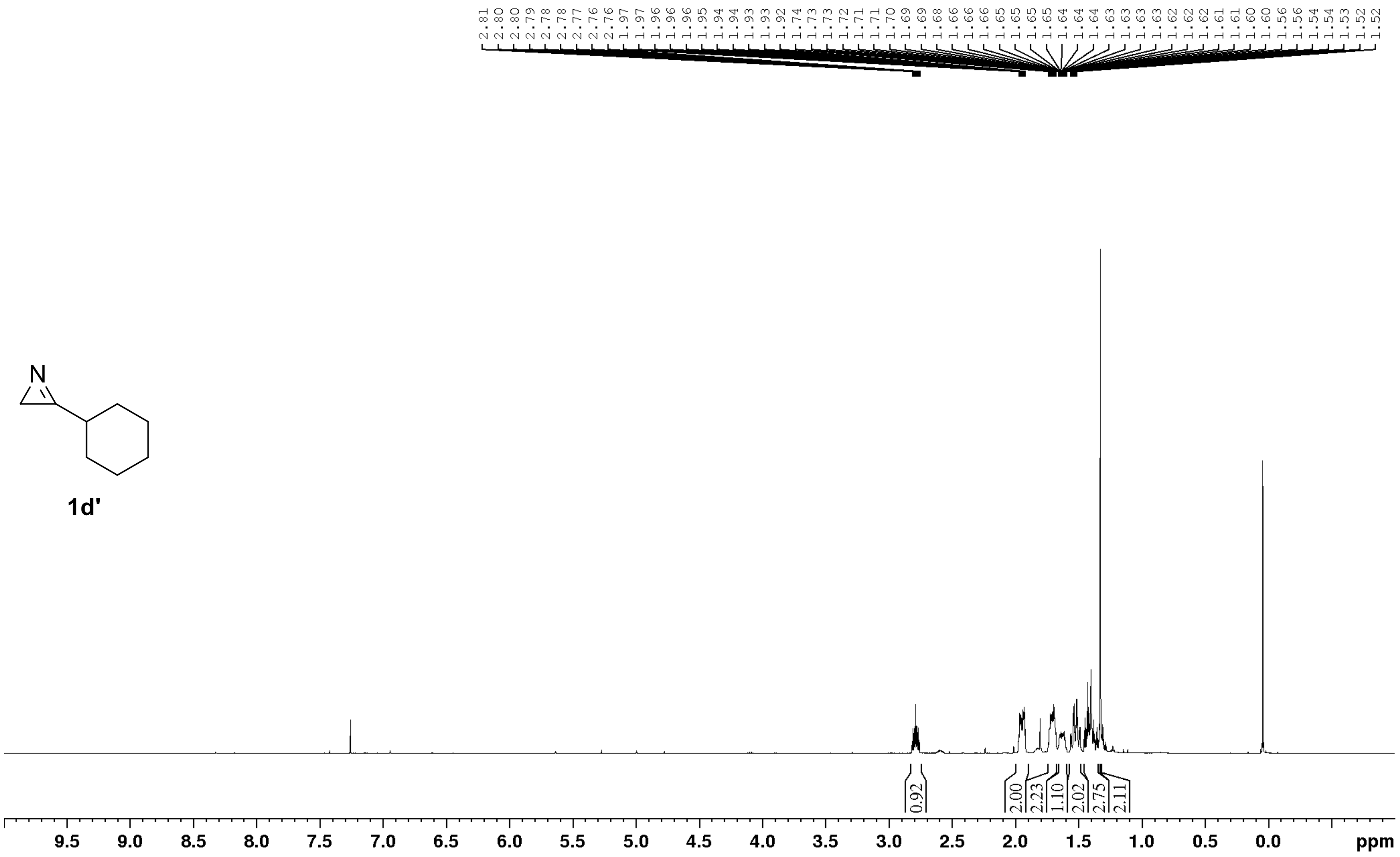

Figure S22. **^{13}C NMR** (126 MHz, $CDCl_3$, 298 K) of **3-cyclohexyl-*2H*-azirine** (**1d'**).

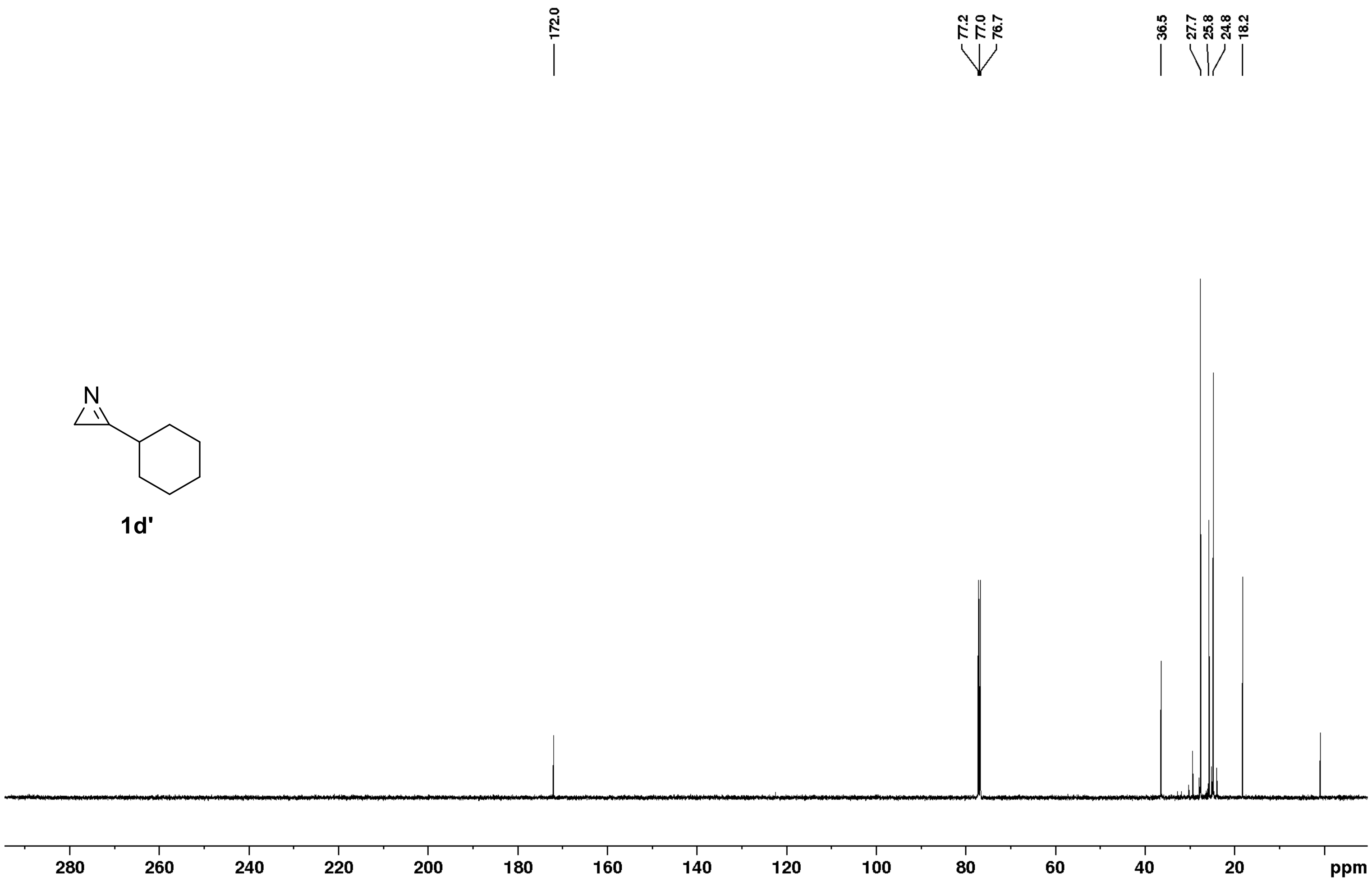

Figure S23. **¹H NMR** (500 MHz, $CDCl_3$, 298 K) of **3-(3-phenylpropyl)-2*H*-azirine** (**1f'**).

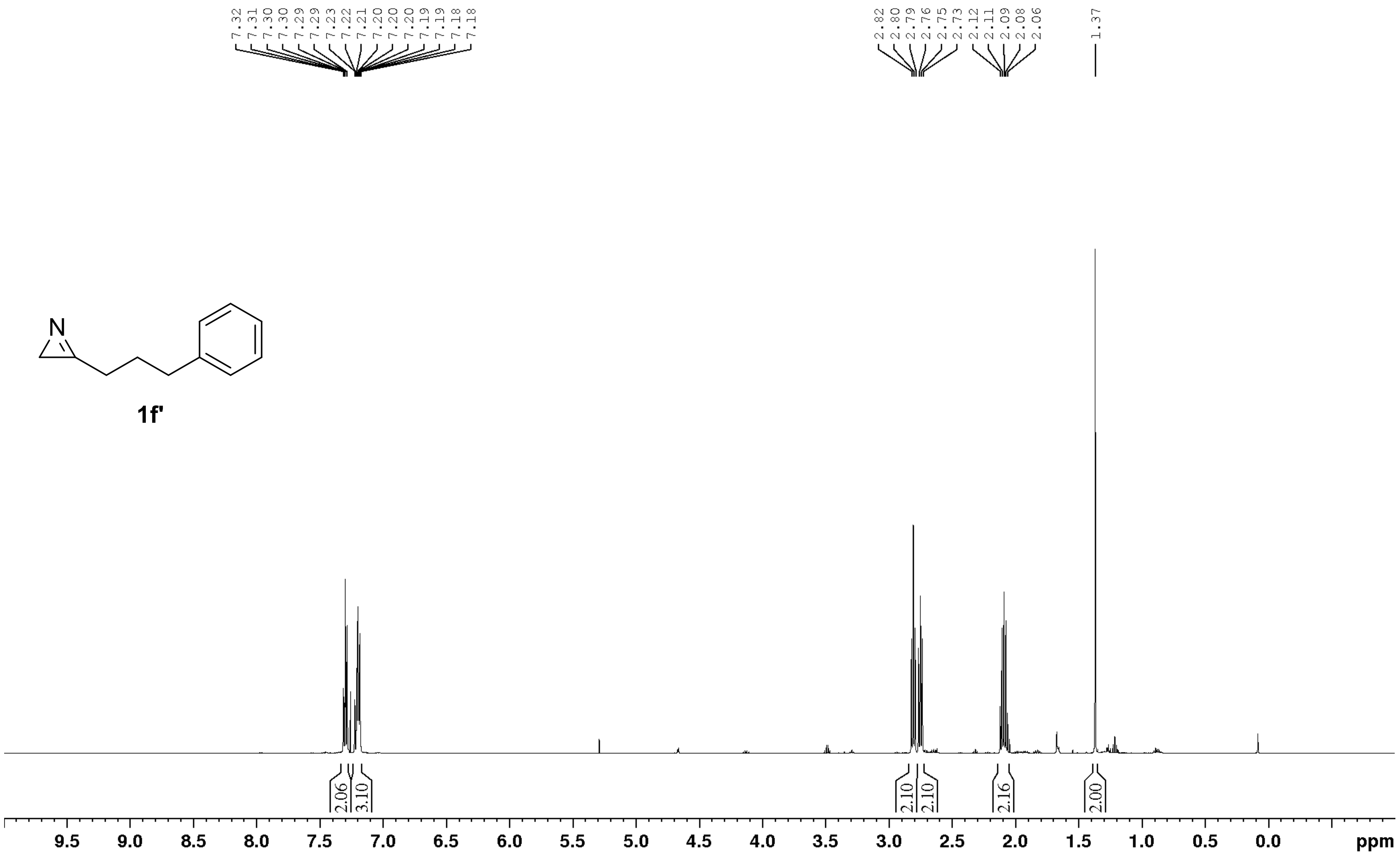

Figure S24. ^{13}**C NMR** (126 MHz, $CDCl_3$, 298 K) of **3-(3-phenylpropyl)-2*H*-azirine** (**1f’**).

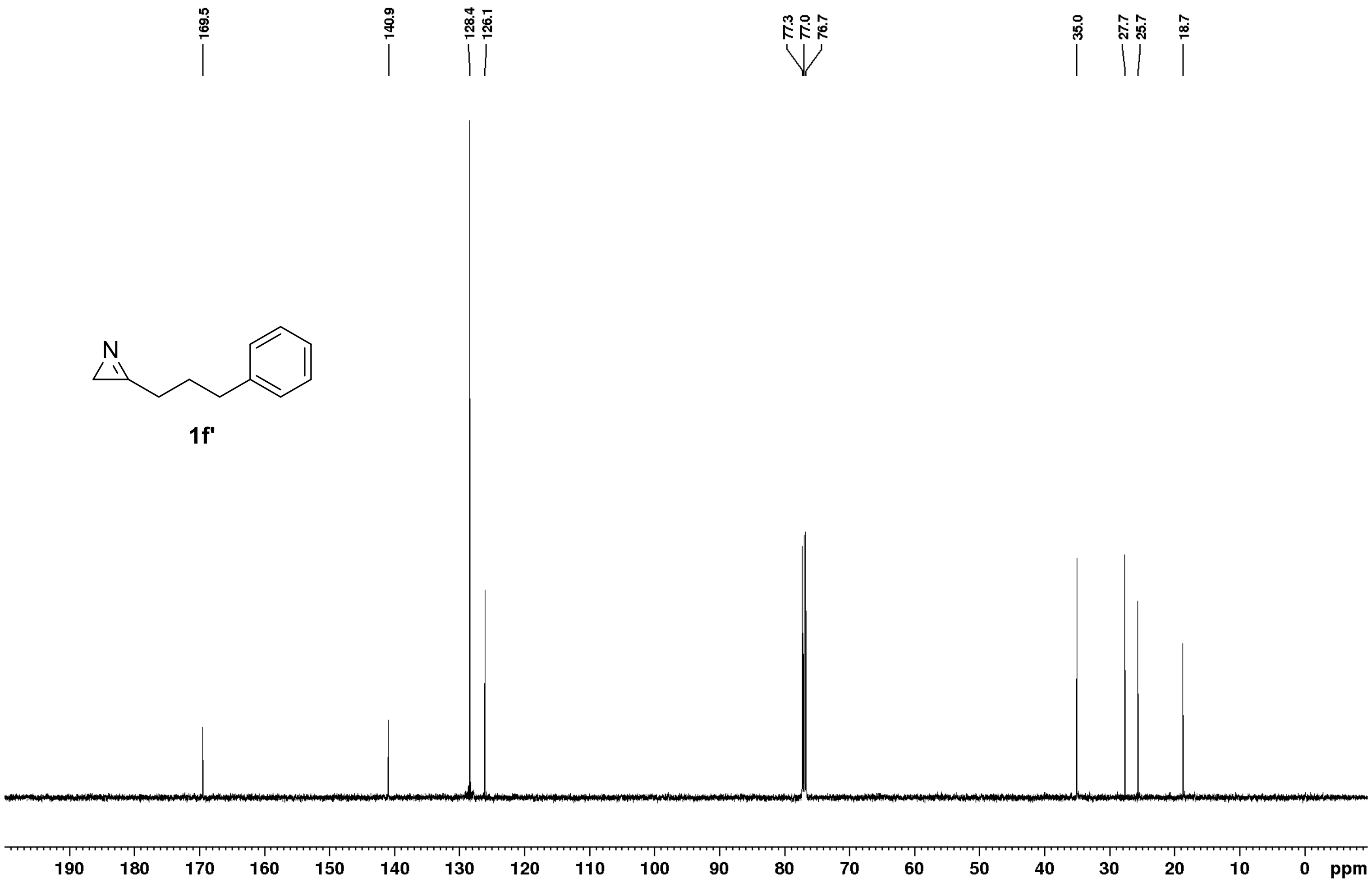

Figure S25. **^{1}H NMR** (500 MHz, $CDCl_3$, 298 K) of **3-(4-chlorobutyl)-2*H*-azirine** (**1g'**).

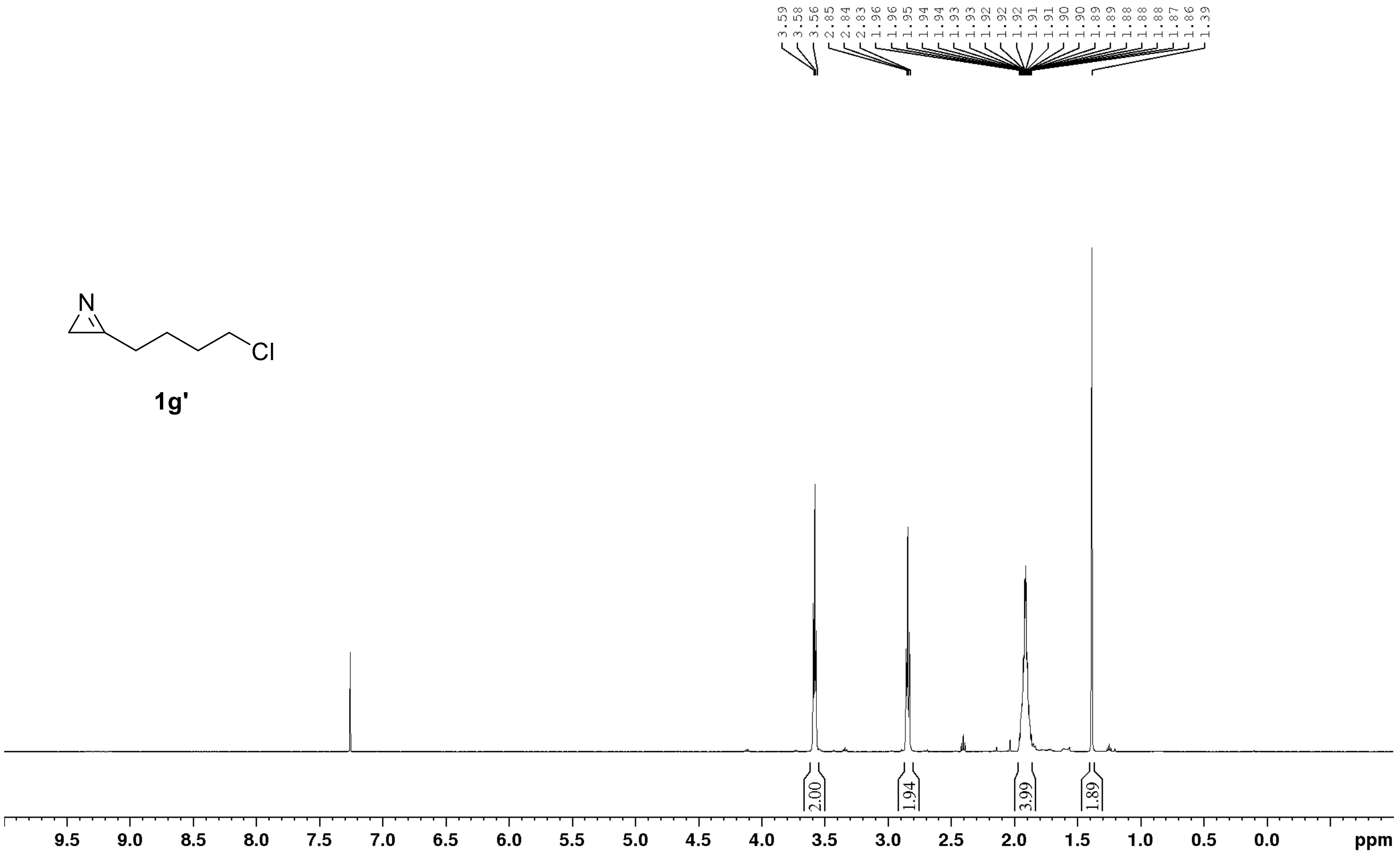

Figure S26. **^{13}C NMR** (126 MHz, $CDCl_3$, 298 K) of **3-(4-chlorobutyl)-2*H*-azirine** (**1g'**).

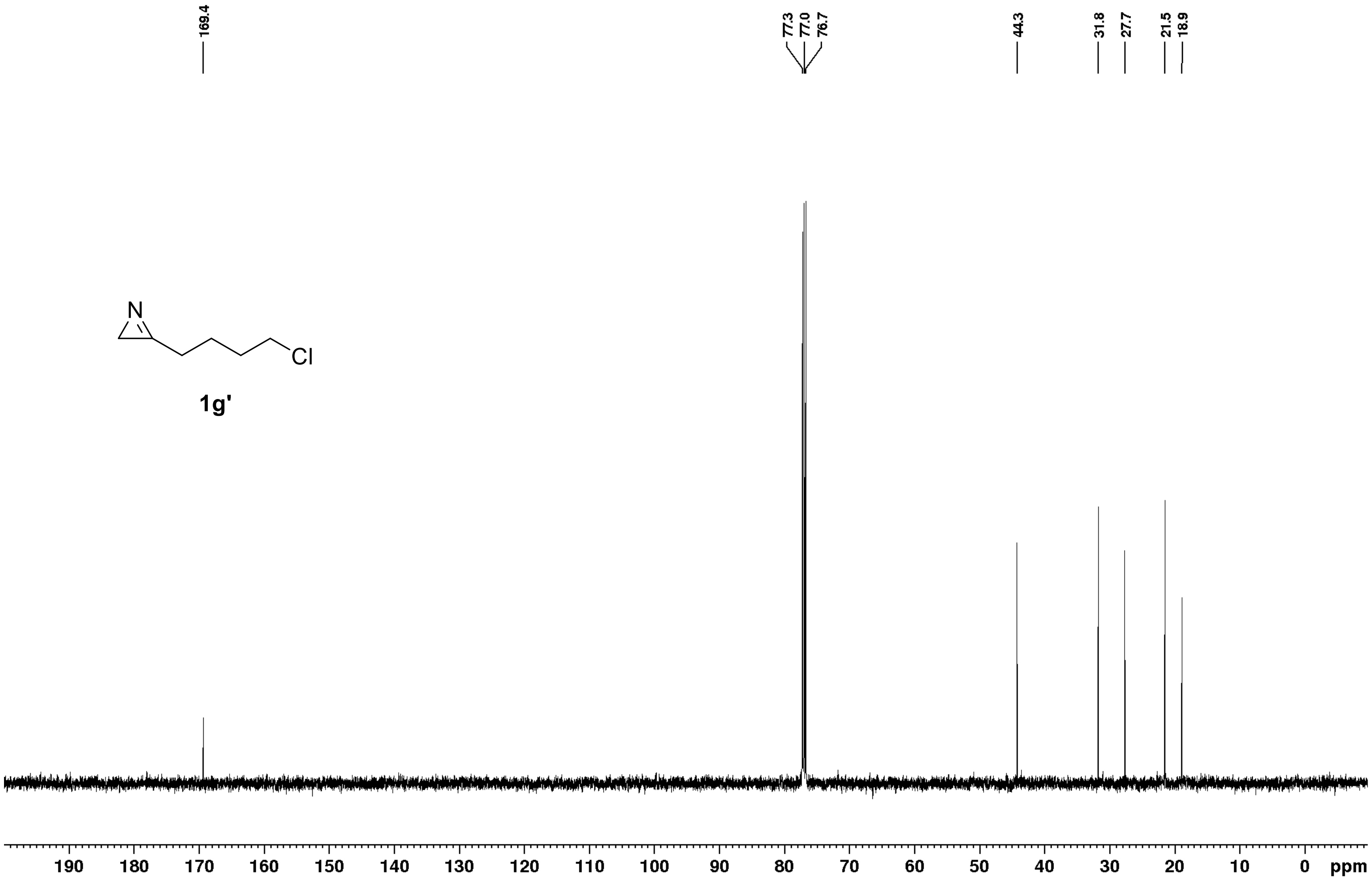

Figure S27. **^{1}H NMR** (500 MHz, $CDCl_3$, 298 K) of **(*S*)-2-(dimethyl(phenyl)silyl)-2-phenylaziridine** [(*S*)-**3aa**].

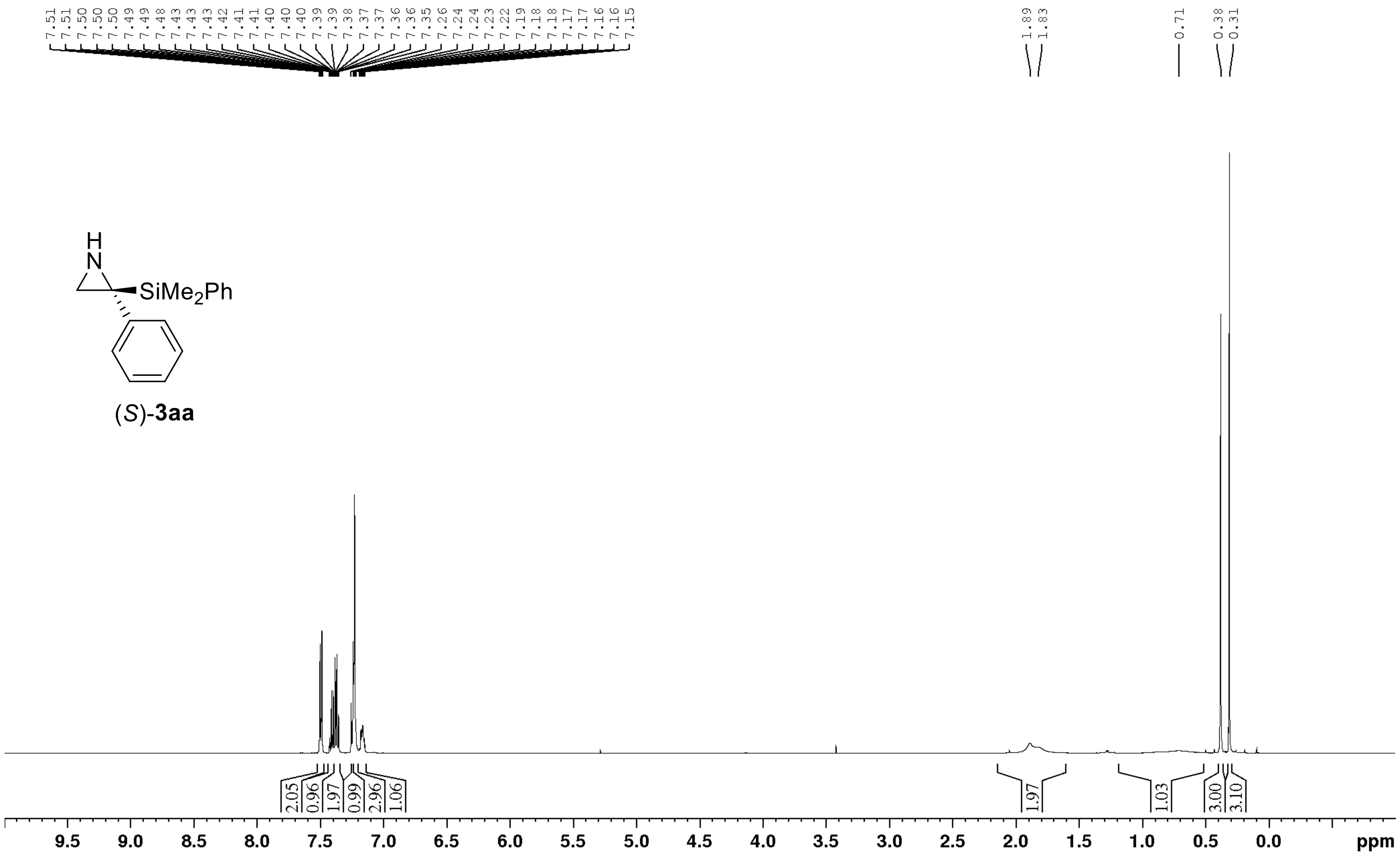

Figure S28. ^{13}**C NMR** (126 MHz, $CDCl_3$, 298 K) of **(*S*)-2-(dimethyl(phenyl)silyl)-2-phenylaziridine** [(*S*)-**3aa**].

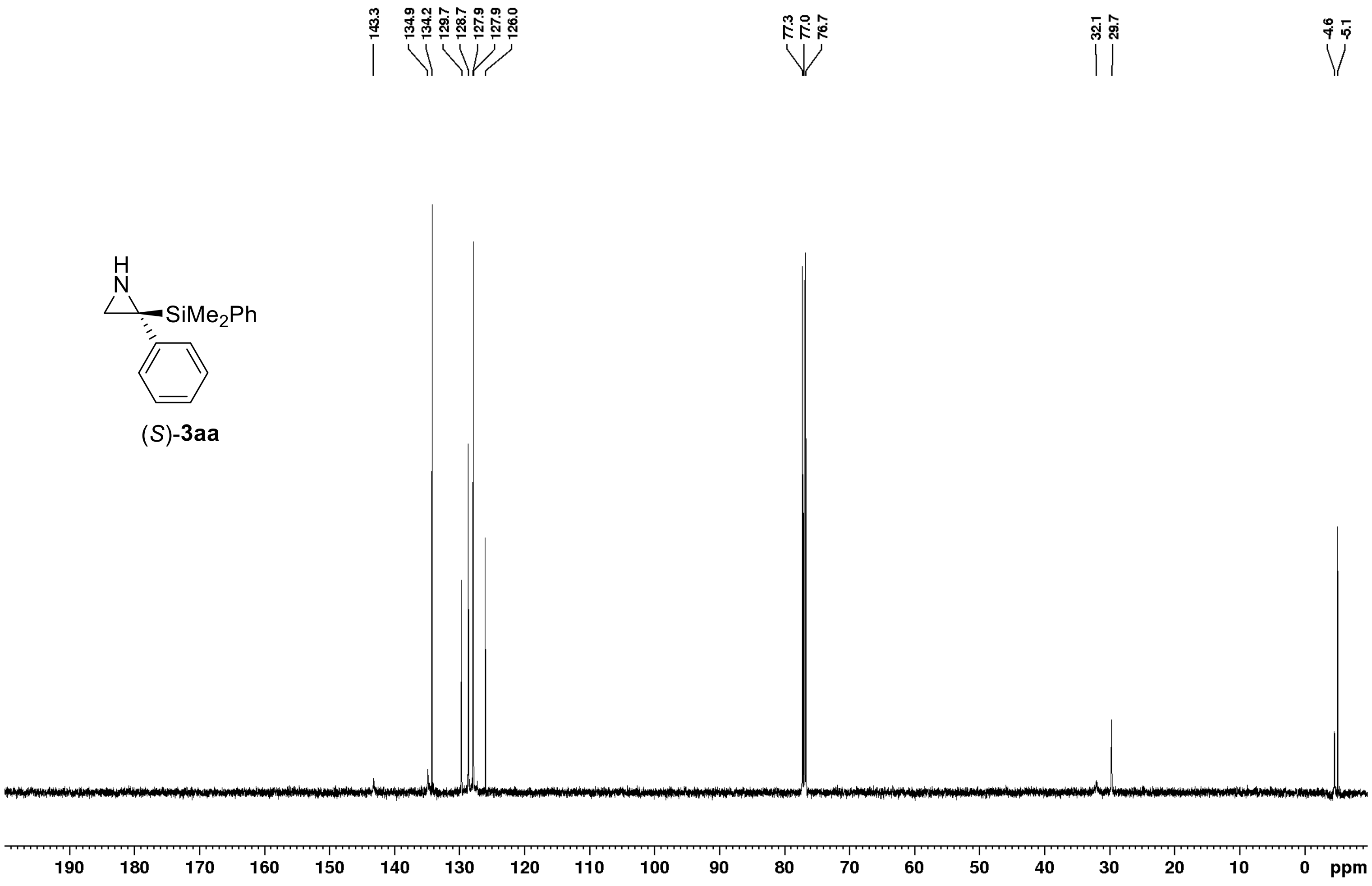

Figure S29. **^{1}H/^{29}Si HMQC NMR** spectrum (500/99 MHz, $CDCl_3$, 298 K, optimized for *J* = 7 Hz) of **(*S*)-2-(dimethyl(phenyl)silyl)-2-phenylaziridine** [(*S*)-**3aa**].

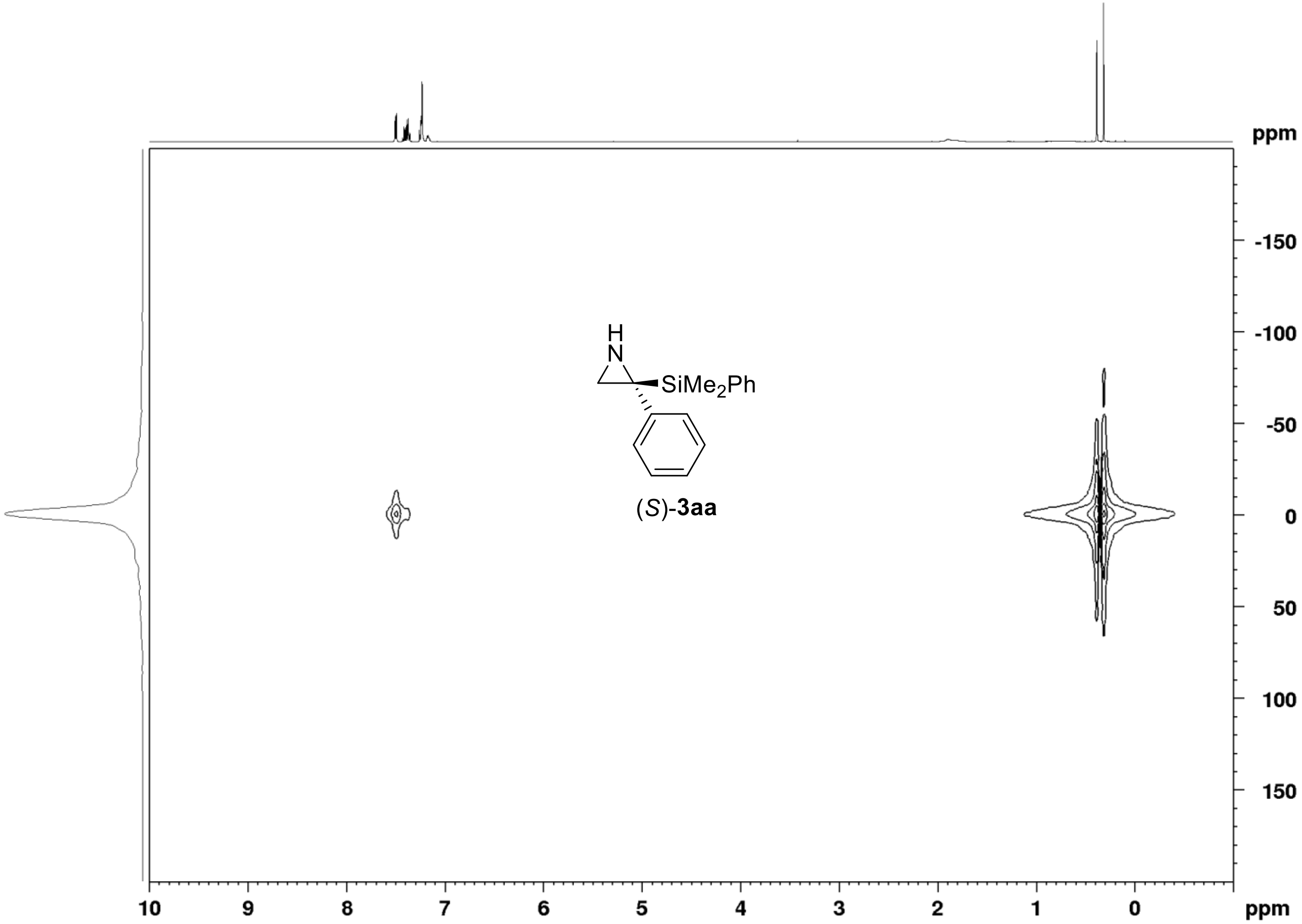

Figure S30. **^{1}H NMR** (500 MHz, $CDCl_3$, 298 K) of **(*S*)-2-(dimethyl(phenyl)silyl)-2-(*m*-tolyl)aziridine** [(*S*)-**3ba**].

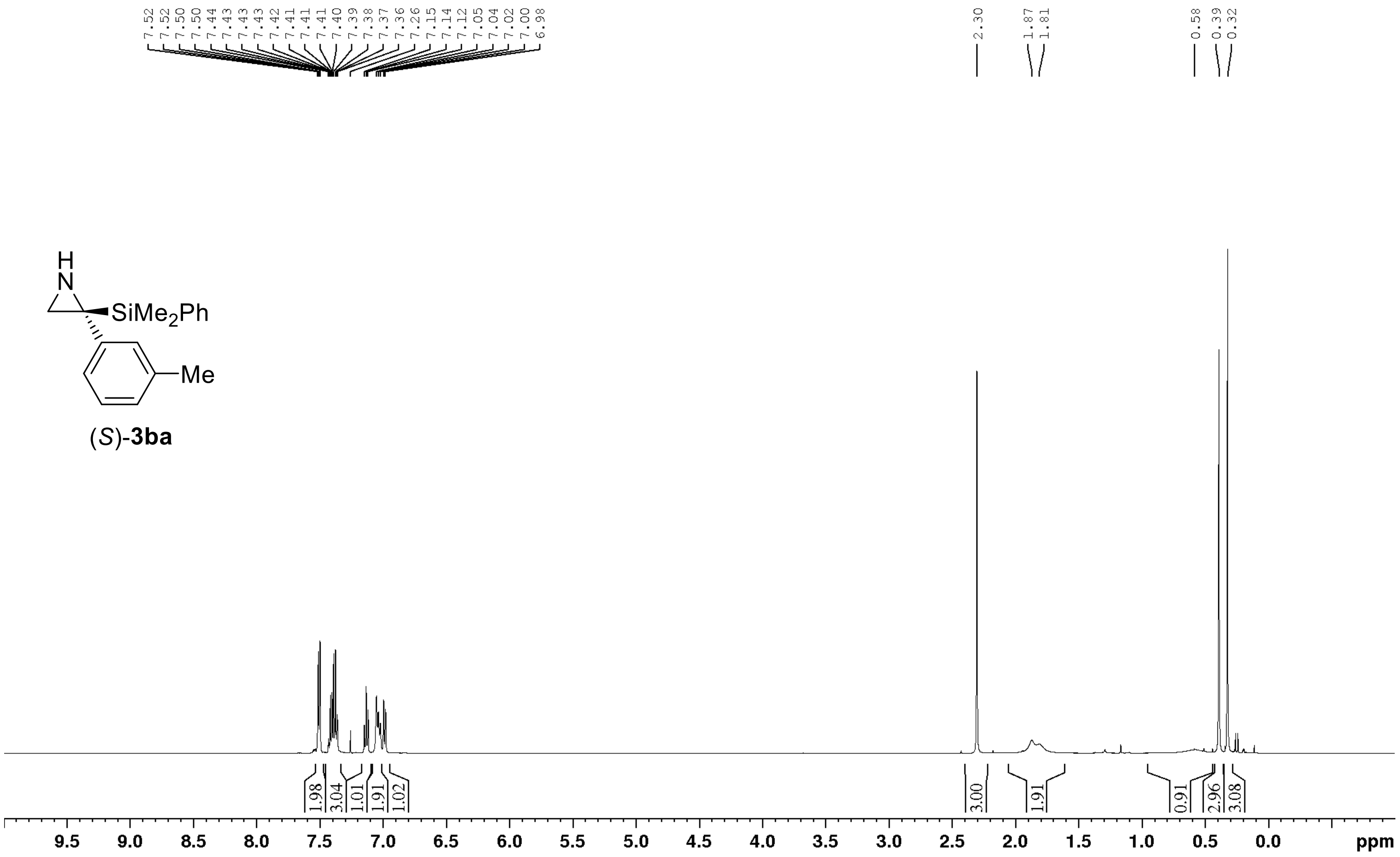

Figure S31. **^{13}C NMR** (126 MHz, $CDCl_3$, 298 K) of **(*S*)-2-(dimethyl(phenyl)silyl)-2-(*m*-tolyl)aziridine** [(*S*)-**3ba**].

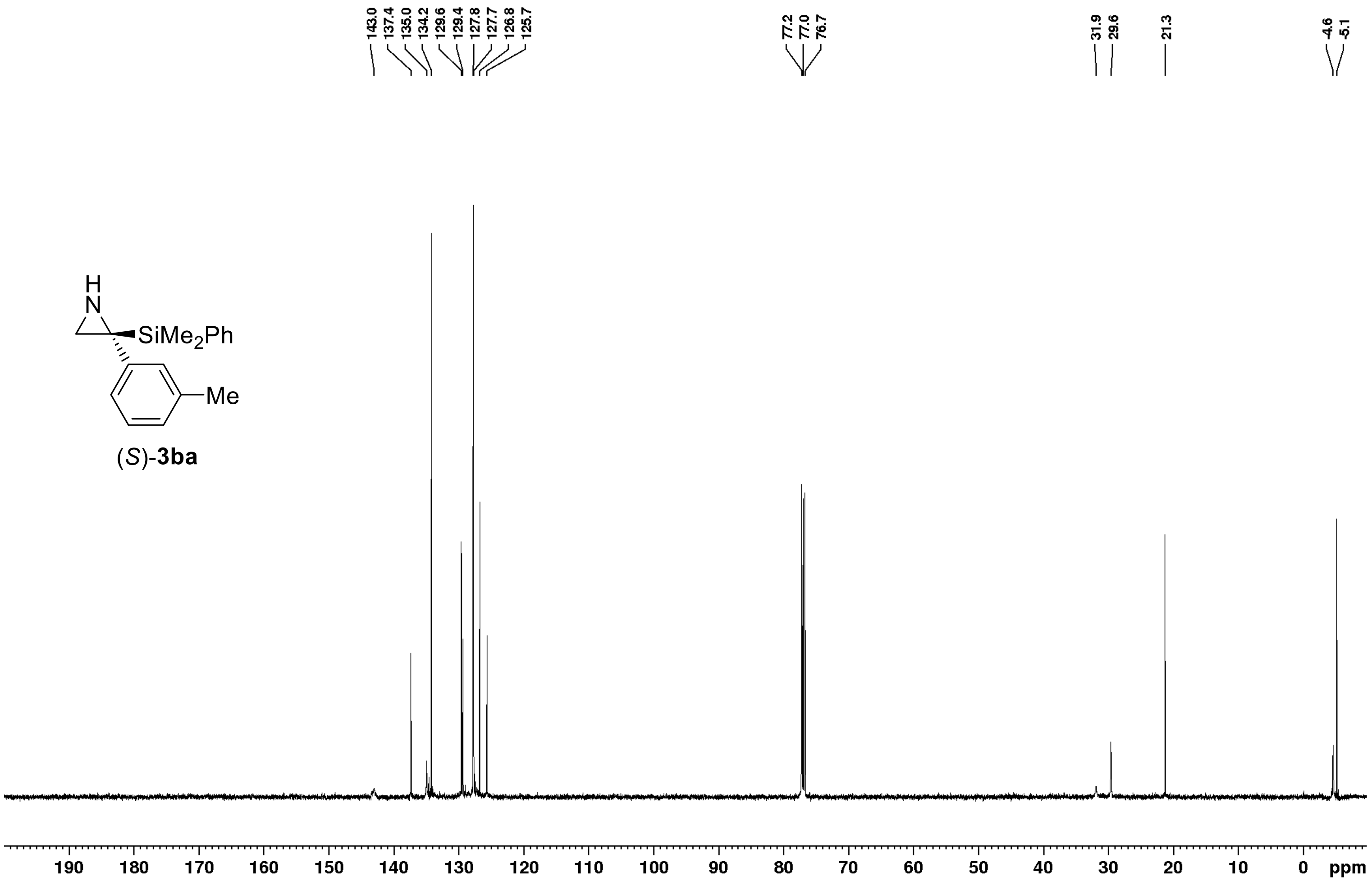

Figure S32. **$^{1}H/^{29}Si$ HMQC NMR** spectrum (500/99 MHz, $CDCl_3$, 298 K, optimized for J = 7 Hz) of **(*S*)-2-(dimethyl(phenyl)silyl)-2-(*m*-tolyl)aziridine** [(*S*)-**3ba**].

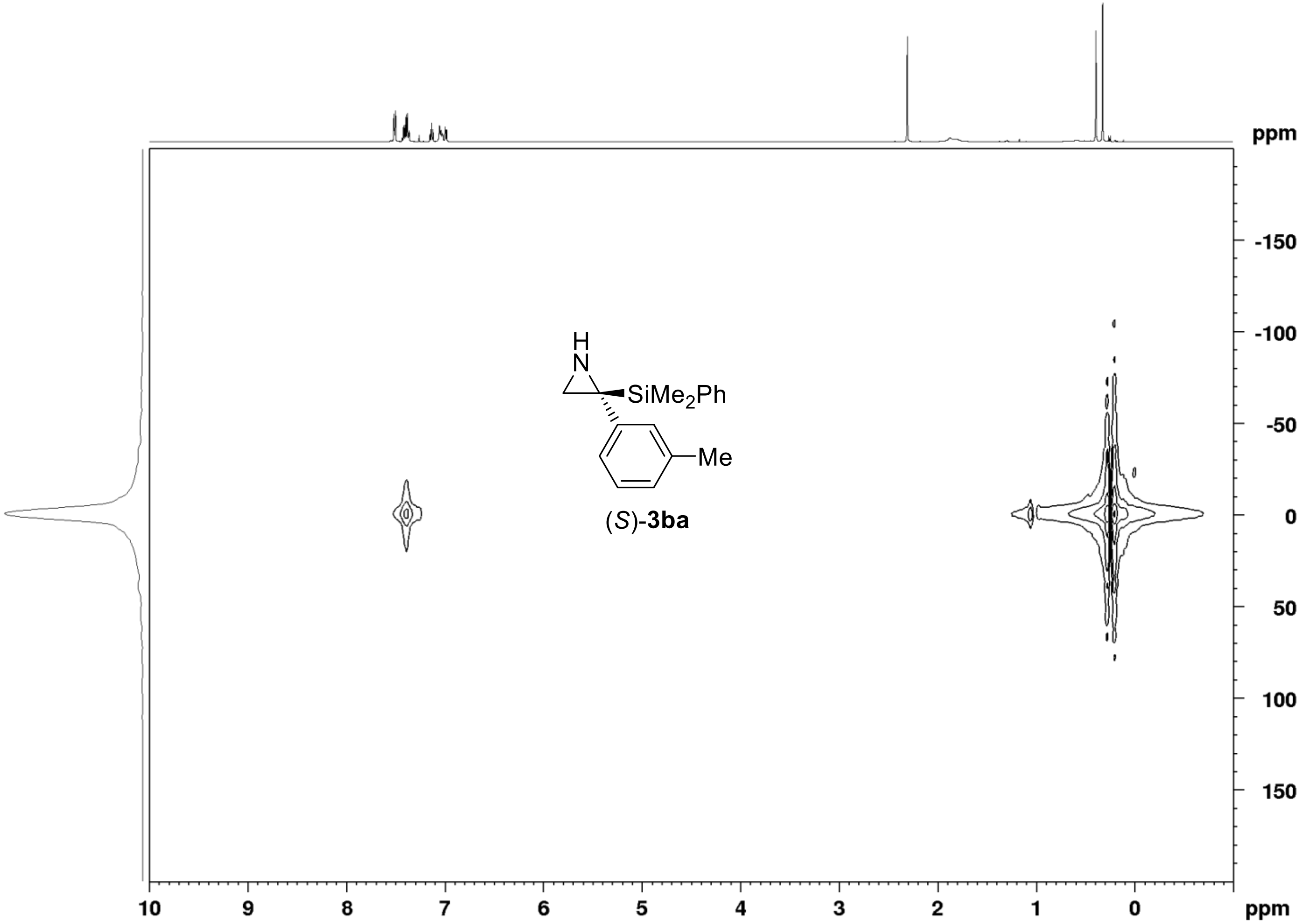

Figure S33. **^{1}H NMR** (500 MHz, $CDCl_3$, 298 K) of **(*S*)-2-(dimethyl(phenyl)silyl)-2-(p-tolyl)aziridine** [(*S*)-**3ca**].

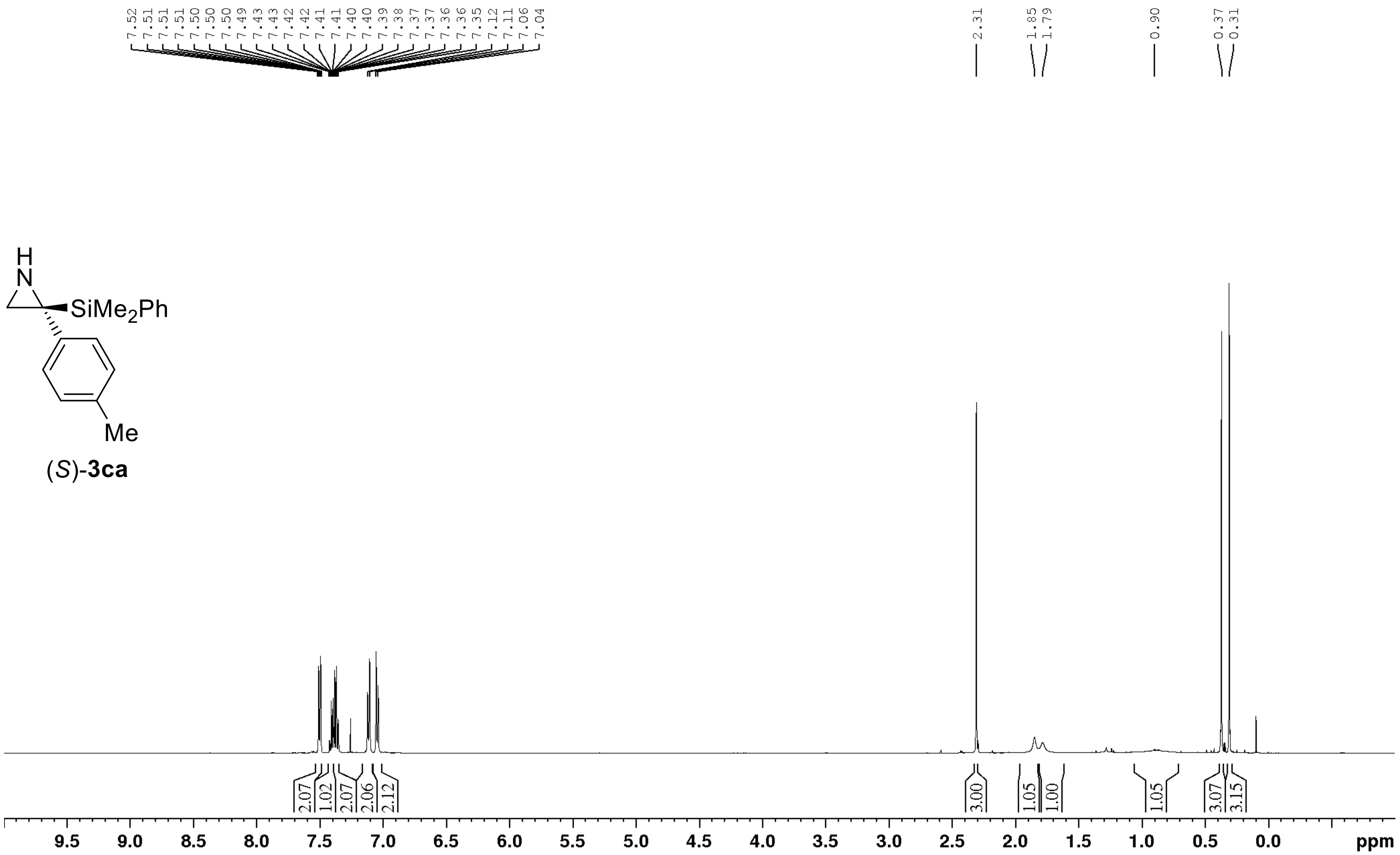

Figure S34. **^{13}C NMR** (126 MHz, $CDCl_3$, 298 K) of **(*S*)-2-(dimethyl(phenyl)silyl)-2-(p-tolyl)aziridine** [(*S*)-**3ca**].

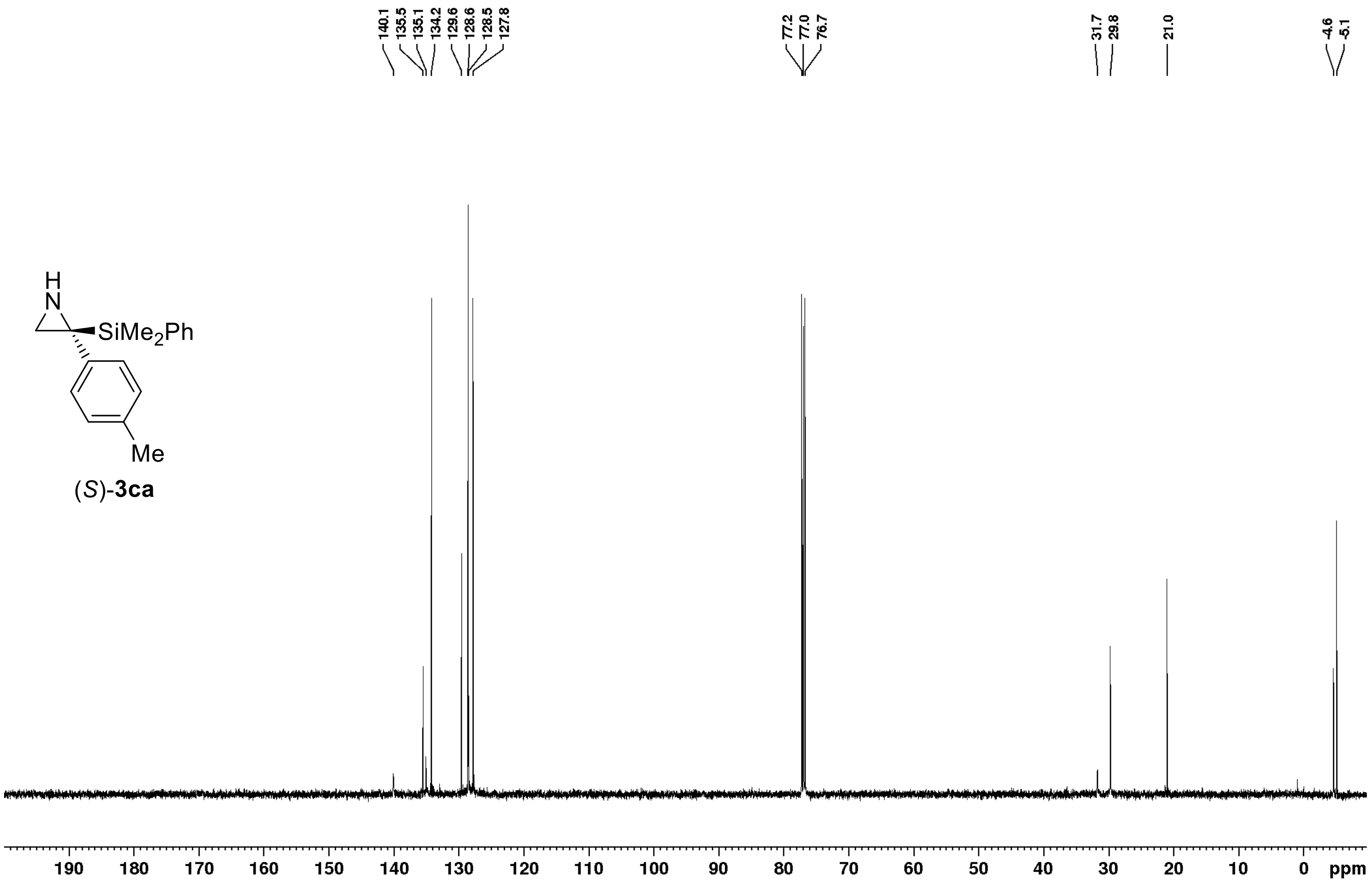

Figure S35. **$^1H/^{29}Si$ HMQC NMR** spectrum (500/99 MHz, $CDCl_3$, 298 K, optimized for *J* = 7 Hz) of **(*S*)-2-(dimethyl(phenyl)silyl)-2-(p-tolyl)aziridine** [(*S*)-**3ca**].

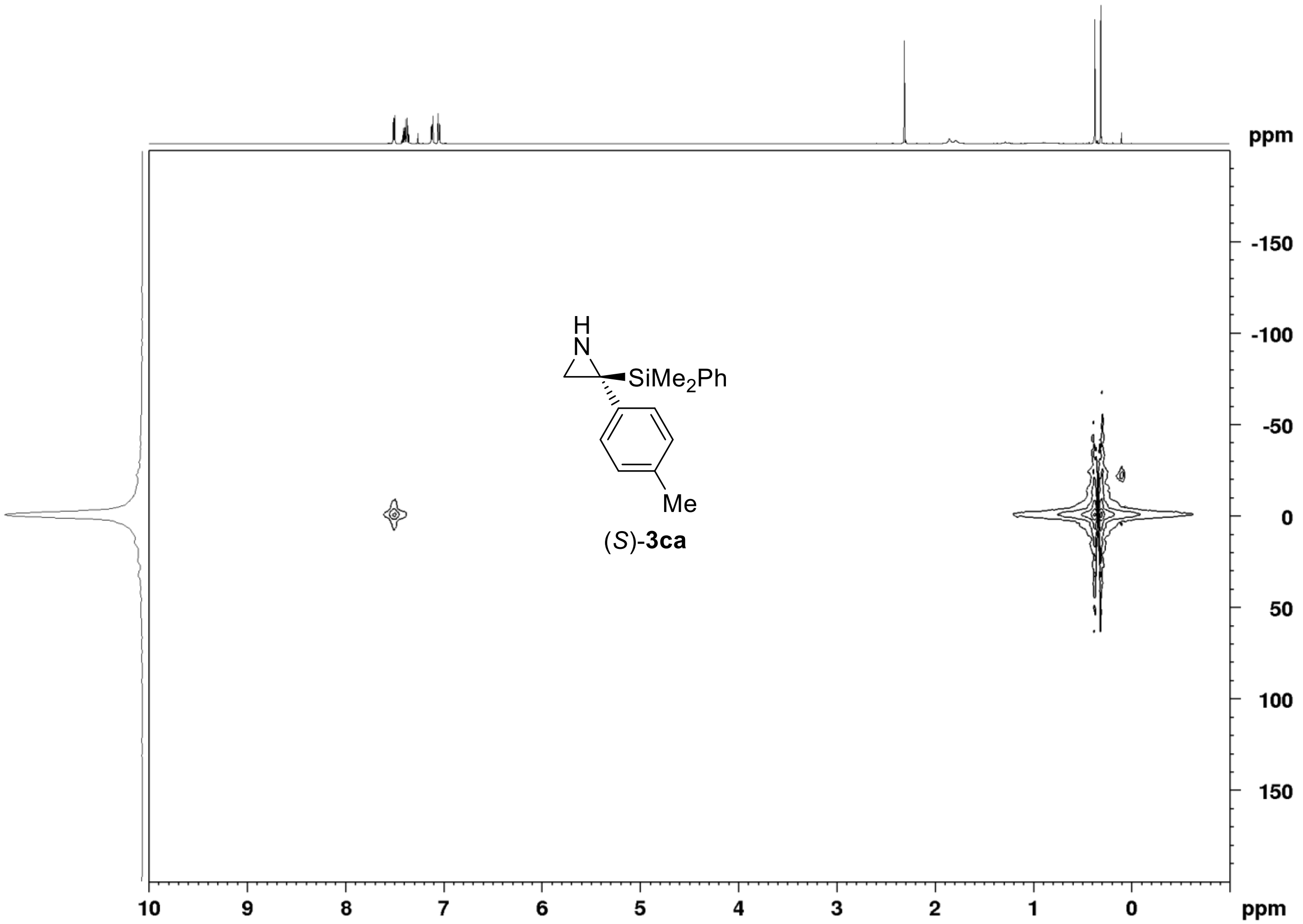

Figure S36. **^{1}H NMR** (500 MHz, CDCl3, 298 K) of **(*S*)-2-(dimethyl(phenyl)silyl)-2-(4-isopropylphenyl)aziridine** [(*S*)-**3da**].

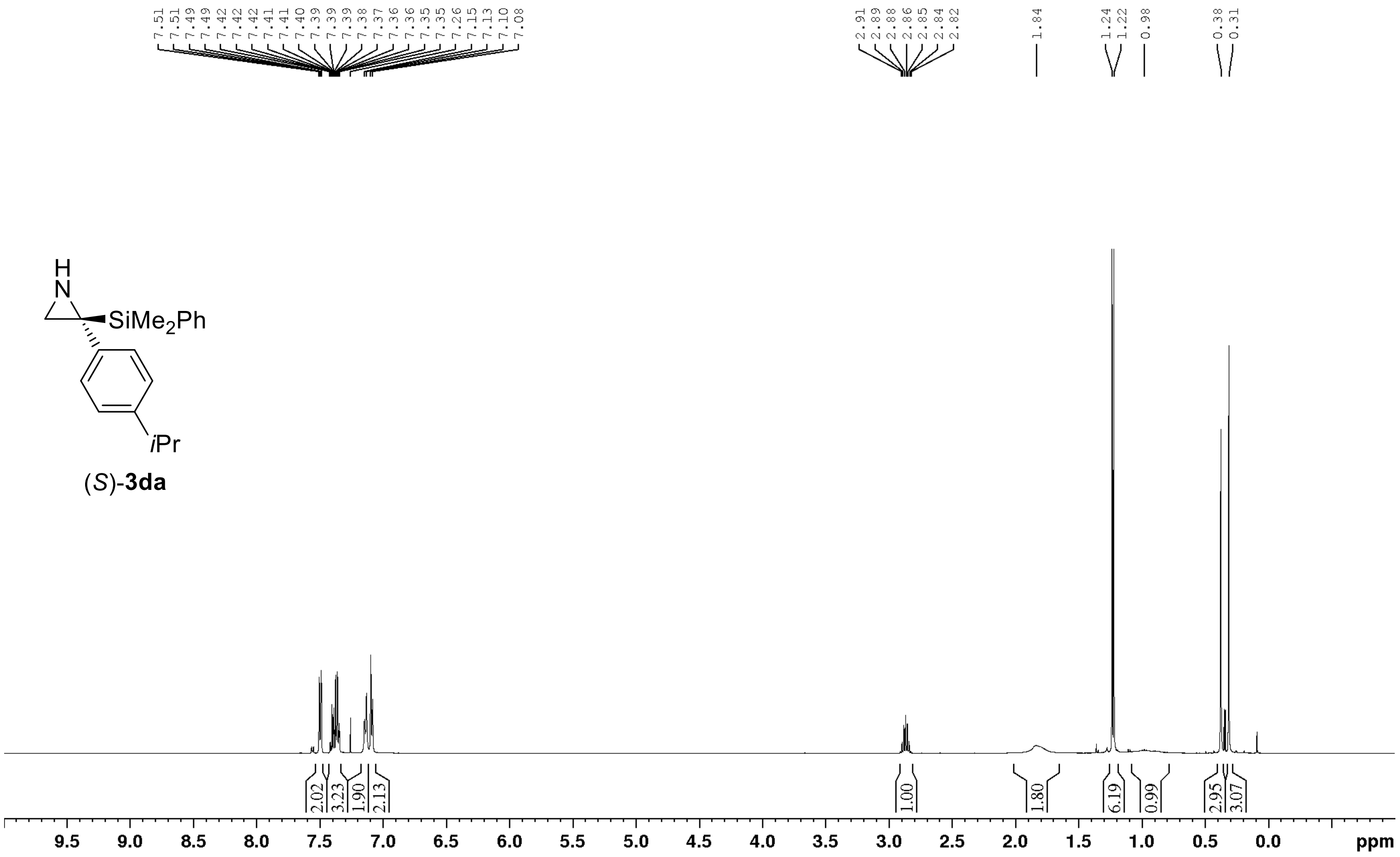

Figure S37. **^{13}C NMR** (126 MHz, $CDCl_3$, 298 K) of **(*S*)-2-(dimethyl(phenyl)silyl)-2-(4-isopropylphenyl)aziridine** [(*S*)-**3da**].

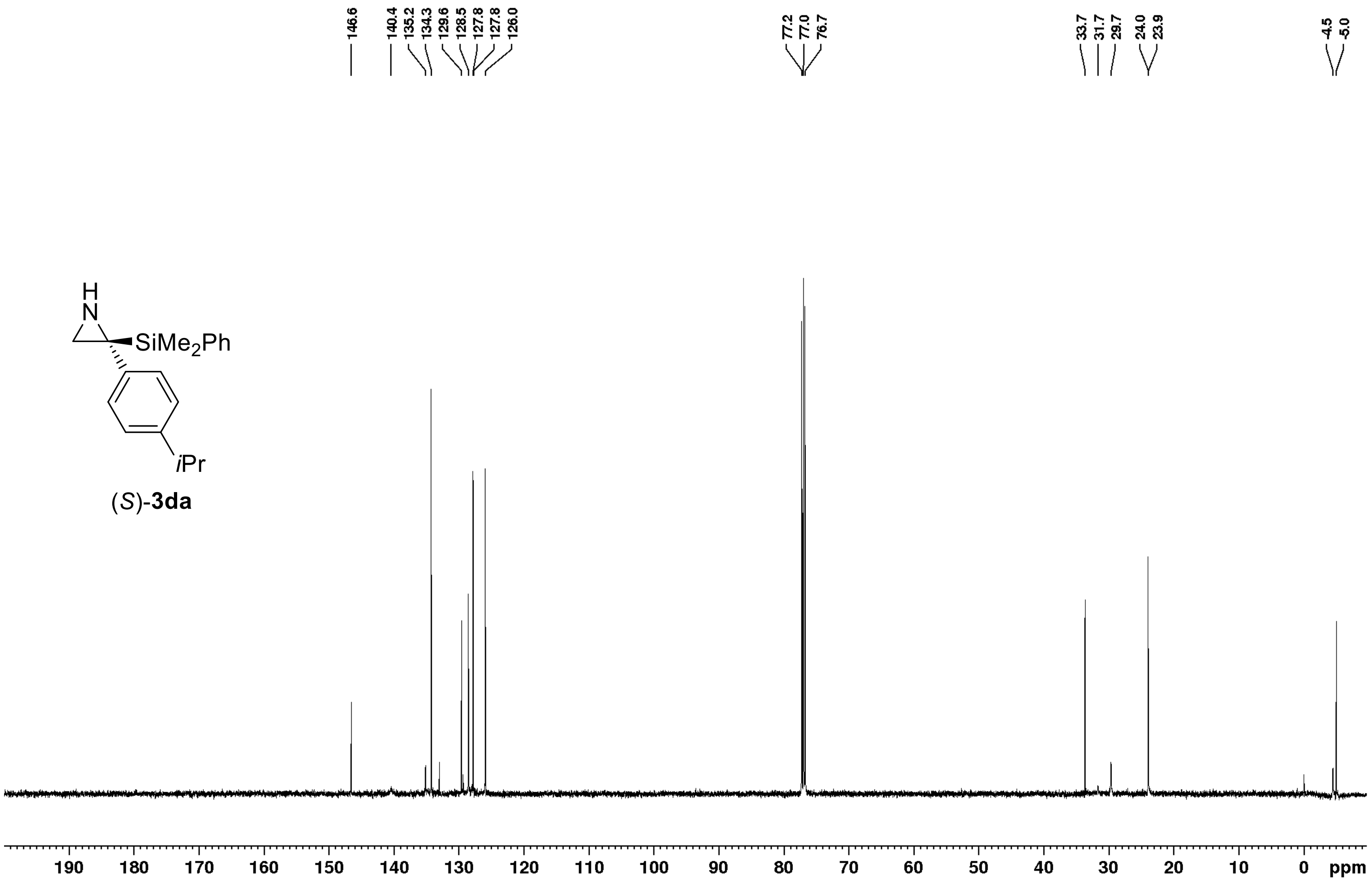

Figure S38. **$^{1}H/^{29}Si$ HMQC NMR** spectrum (500/99 MHz, $CDCl_3$, 298 K, optimized for *J* = 7 Hz) of **(*S*)-2-(dimethyl(phenyl)silyl)-2-(4-isopropylphenyl)aziridine** [(*S*)-**3da**].

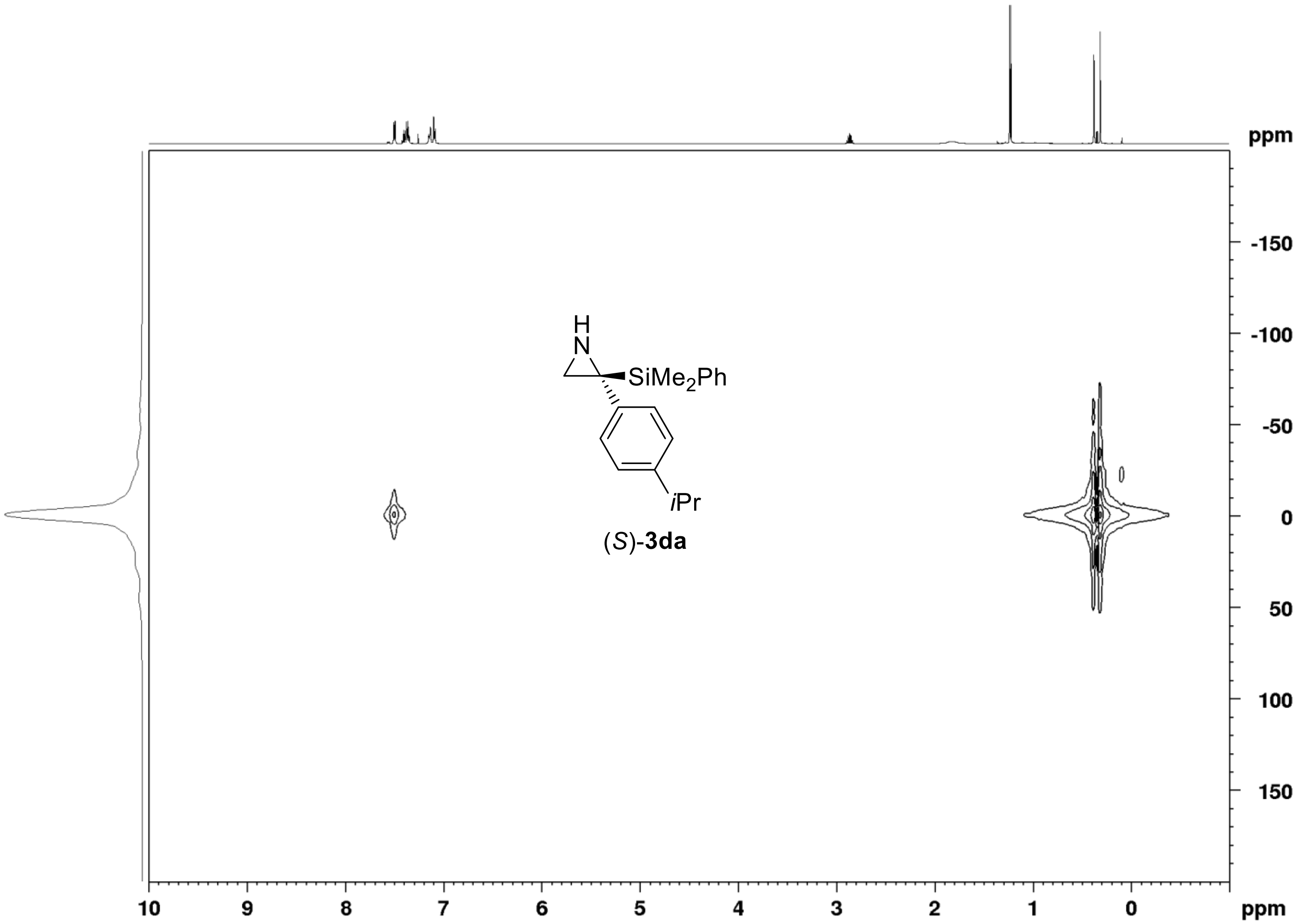

Figure S39. **^{1}H NMR** (500 MHz, $CDCl_3$, 298 K) of **(*S*)-2-(4-(*tert*-butyl)phenyl)-2-(dimethyl(phenyl)silyl)aziridine** [(*S*)-**3ea**].

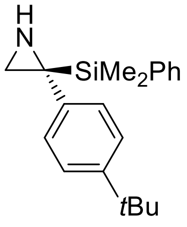

(*S*)-**3ea**

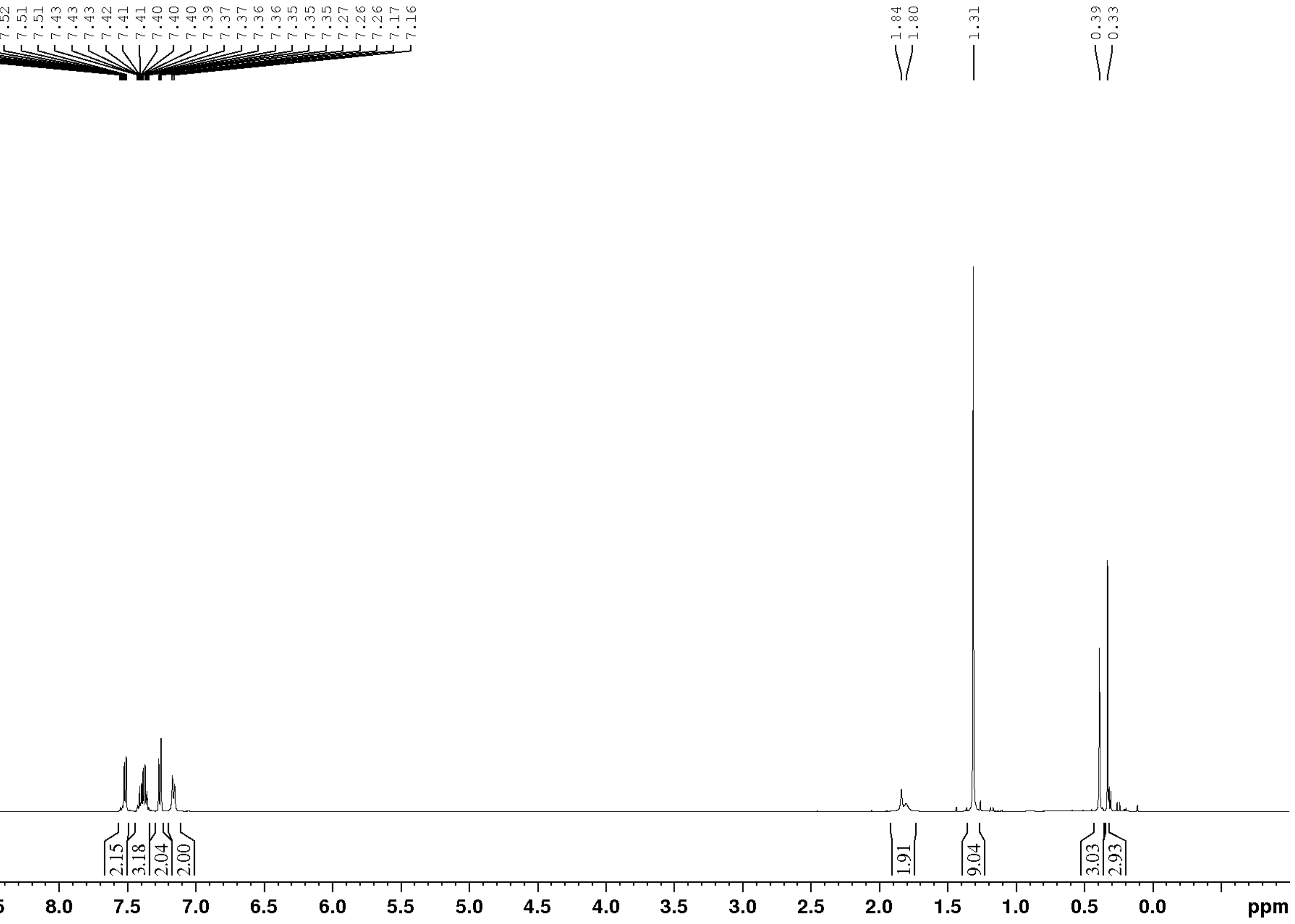

Figure S40. **^{13}C NMR** (126 MHz, $CDCl_3$, 298 K) of **(*S*)-2-(4-(*tert*-butyl)phenyl)-2-(dimethyl(phenyl)silyl)aziridine** [(*S*)-**3ea**].

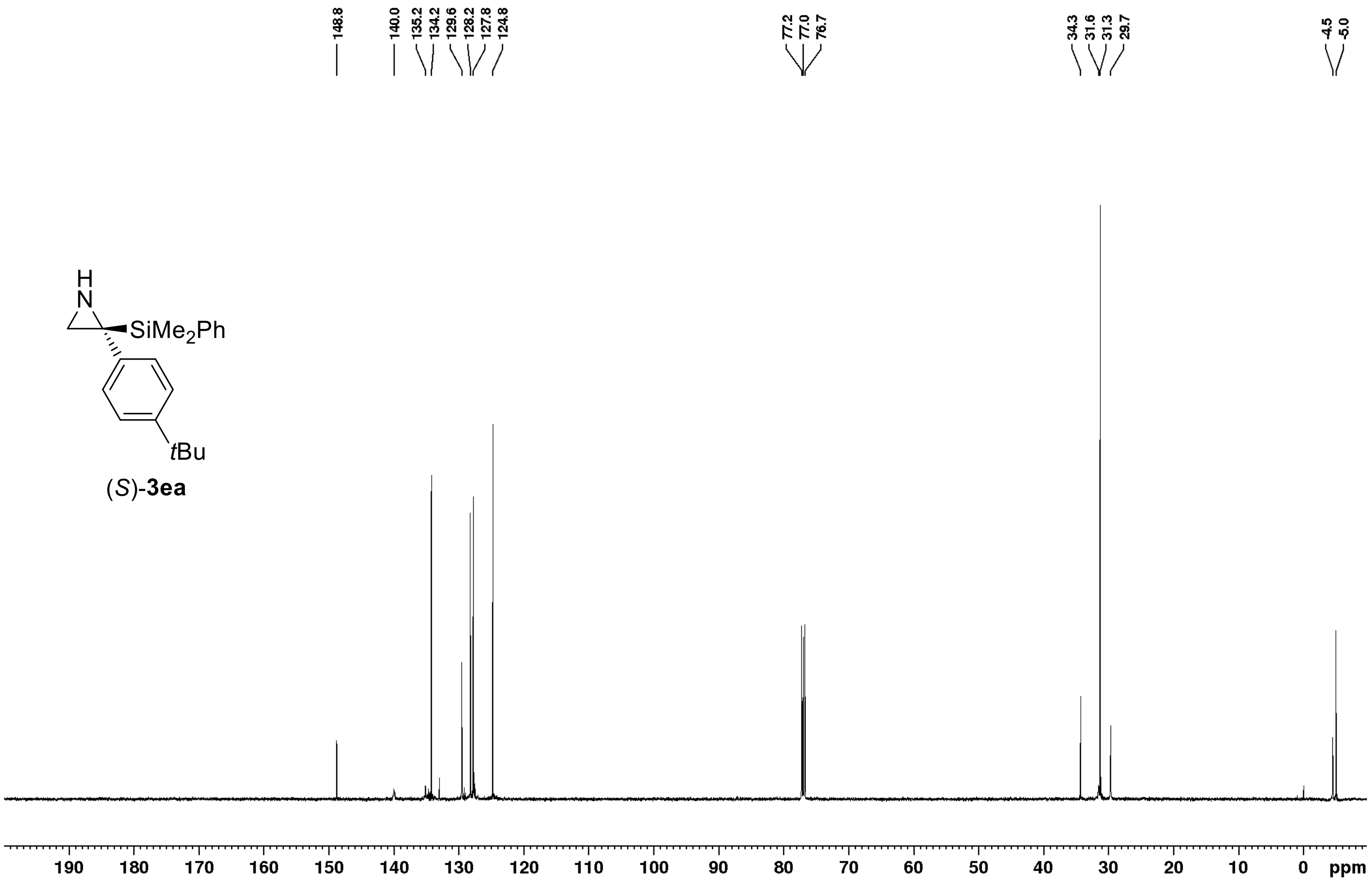

Figure S41. **$^{1}H/^{29}Si$ HMQC NMR** spectrum (500/99 MHz, $CDCl_3$, 298 K, optimized for *J* = 7 Hz) of **(*S*)-2-(4-(*tert*-butyl)phenyl)-2-(dimethyl(phenyl)silyl)aziridine** [(*S*)-**3ea**].

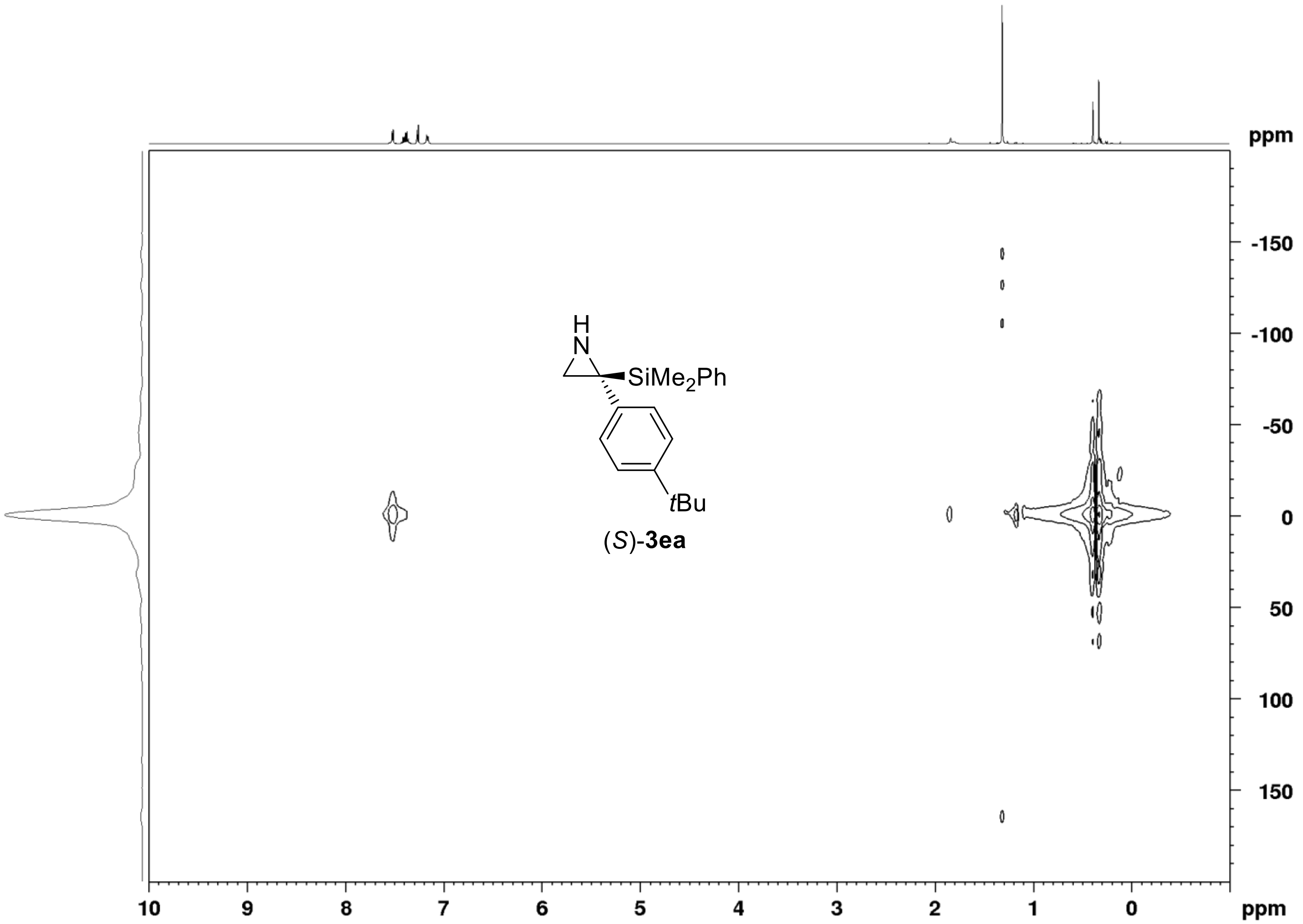

Figure S42. **^{1}H NMR** (500 MHz, $CDCl_3$, 298 K) of **(*S*)-2-(dimethyl(phenyl)silyl)-2-(3,4-dimethylphenyl)aziridine** [(*S*)-**3fa**].

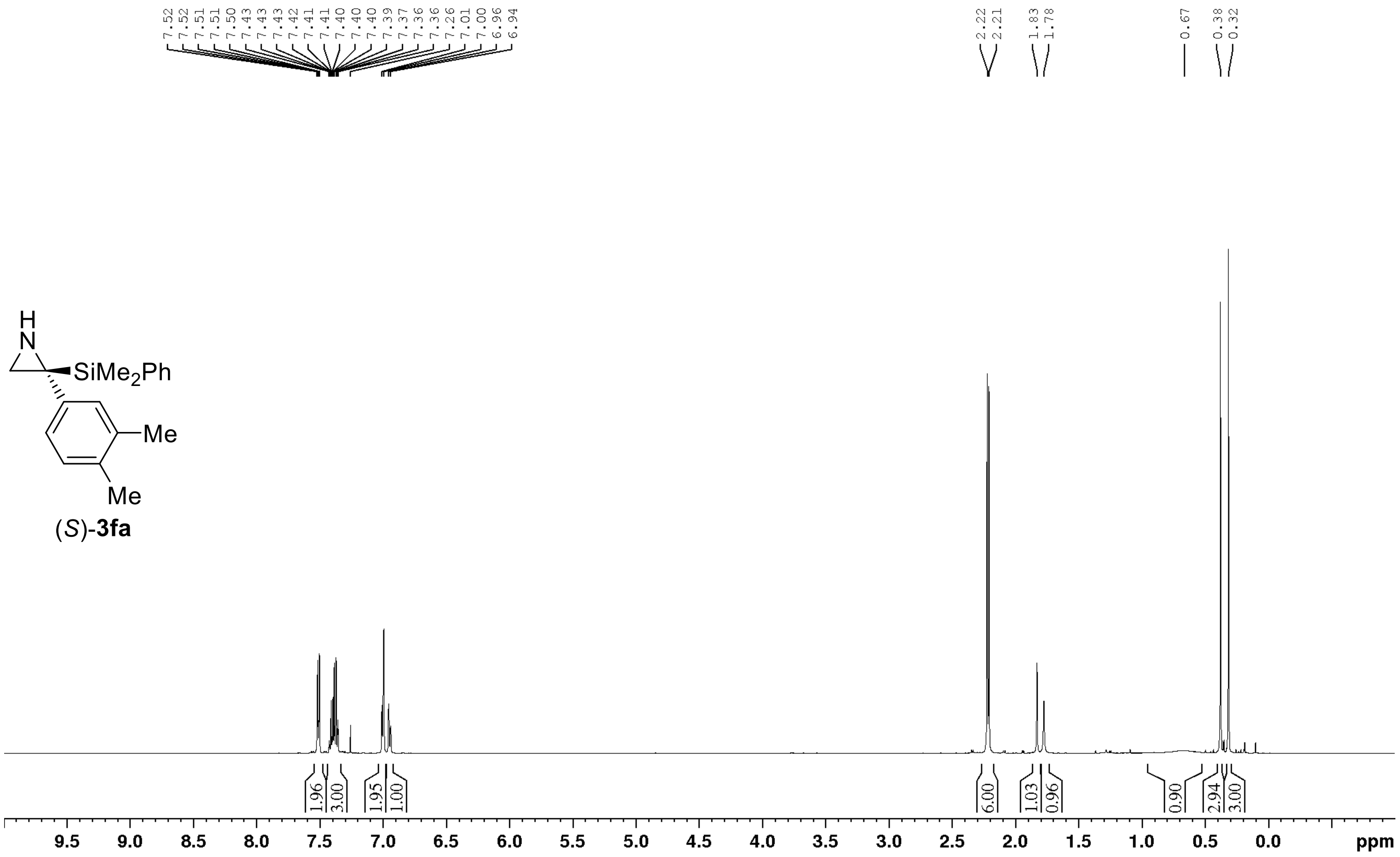

Figure S43. **^{13}C NMR** (126 MHz, $CDCl_3$, 298 K) of **(*S*)-2-(dimethyl(phenyl)silyl)-2-(3,4-dimethylphenyl)aziridine** [(*S*)-**3fa**].

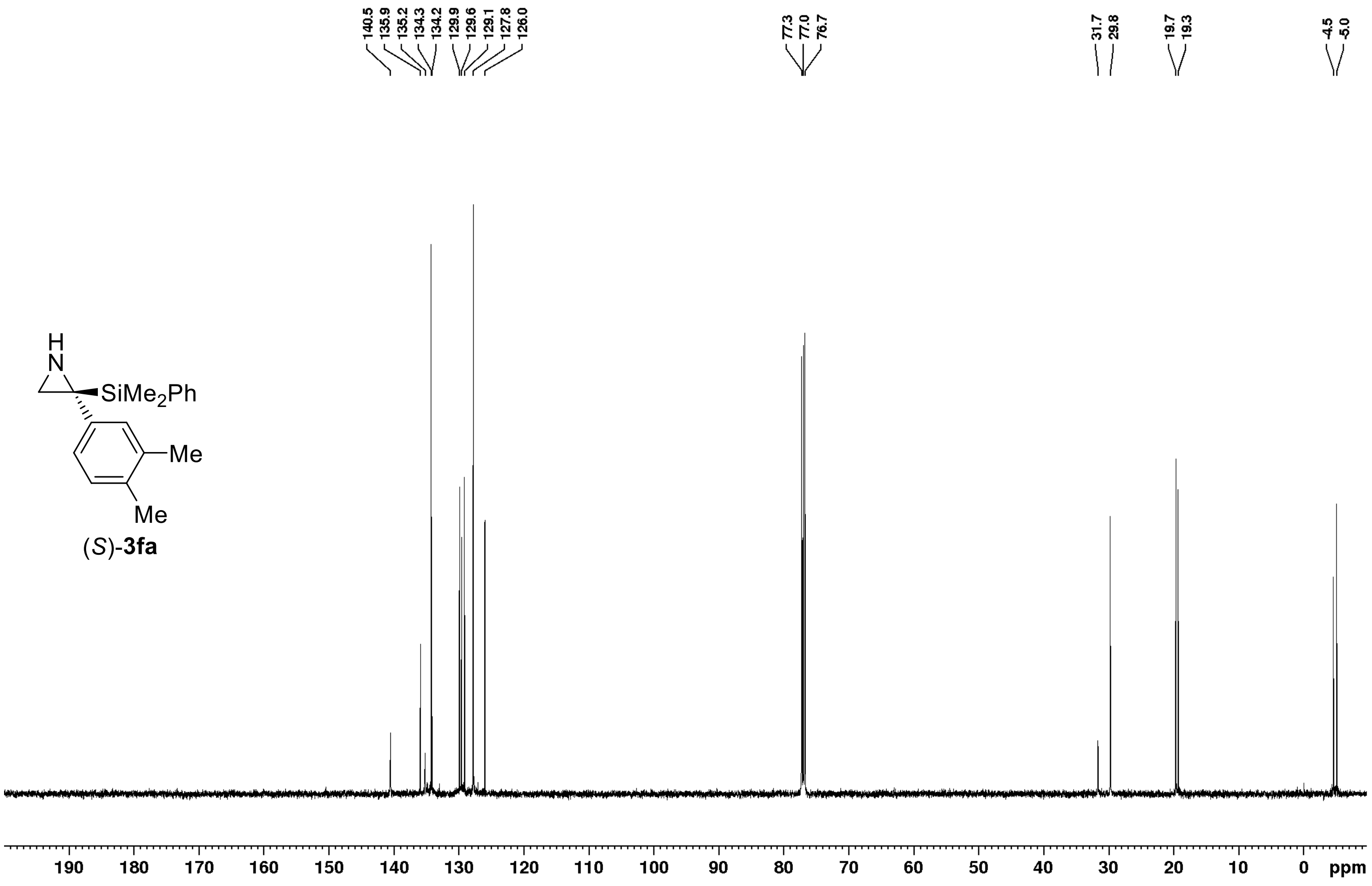

Figure S44. **$^1H/^{29}Si$ HMQC NMR** spectrum (500/99 MHz, $CDCl_3$, 298 K, optimized for *J* = 7 Hz) of **(*S*)-2-(dimethyl(phenyl)silyl)-2-(3,4-dimethylphenyl)aziridine** [(*S*)-**3fa**].

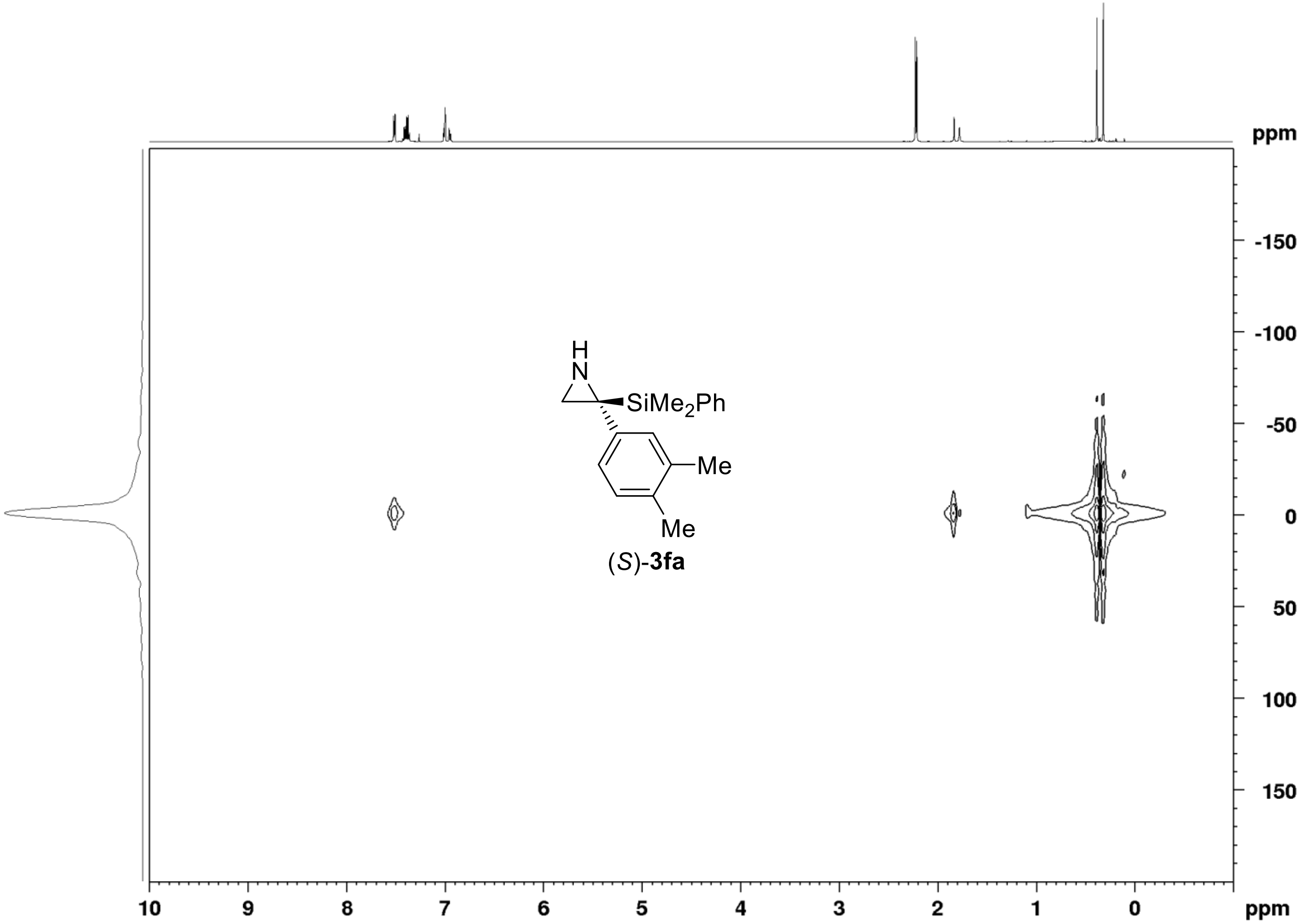

Figure S45. **^{1}H NMR** (500 MHz, $CDCl_3$, 298 K) of **(*S*)-2-(4-(2-(dimethyl(phenyl)silyl)aziridin-2-yl)benzyl)isoindoline-1,3-dione** [(*S*)-**3ga**].

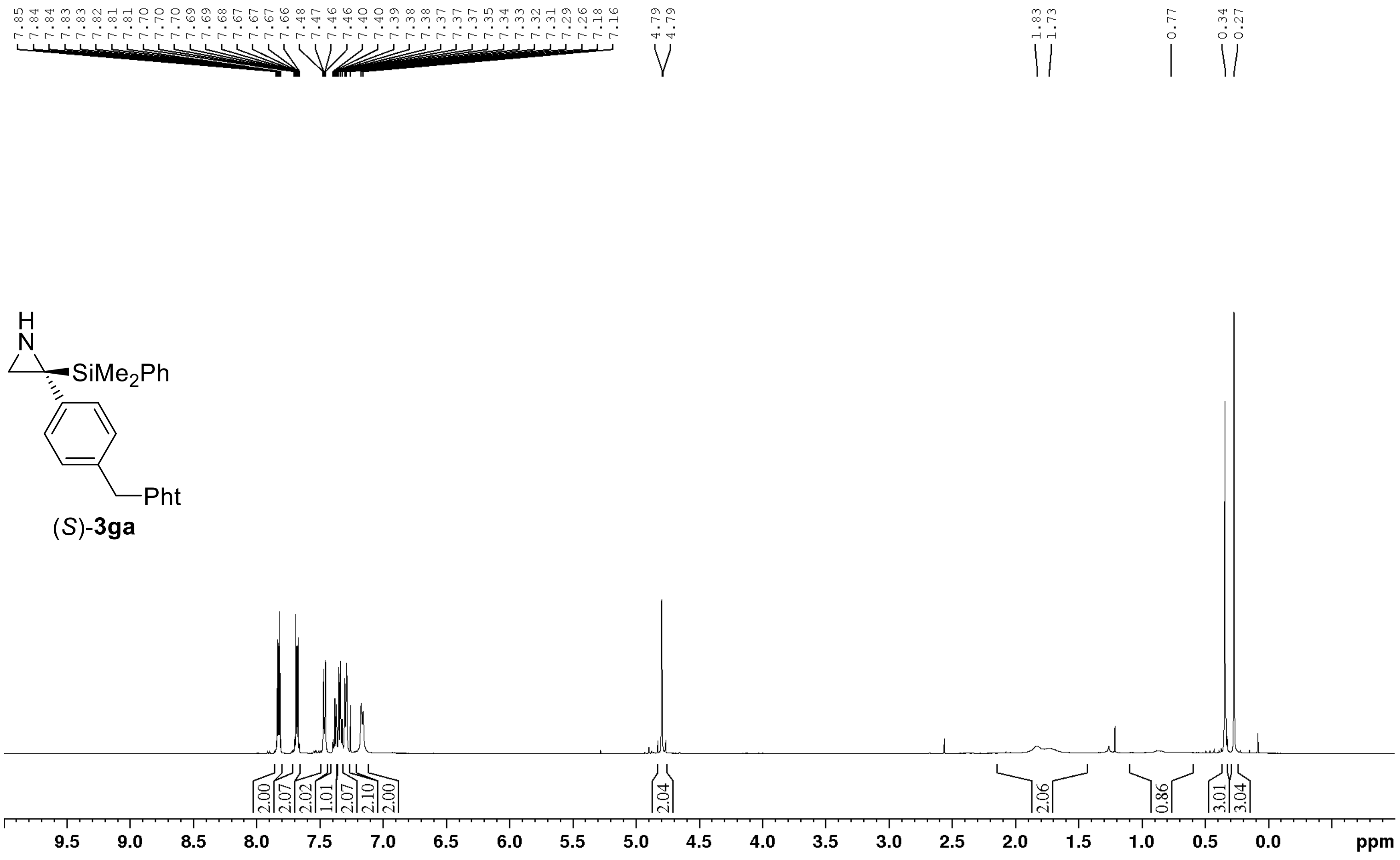

Figure S46. **^{13}C NMR** (126 MHz, $CDCl_3$, 298 K) of **(*S*)-2-(4-(2-(dimethyl(phenyl)silyl)aziridin-2-yl)benzyl)isoindoline-1,3-dione** [(*S*)-**3ga**].

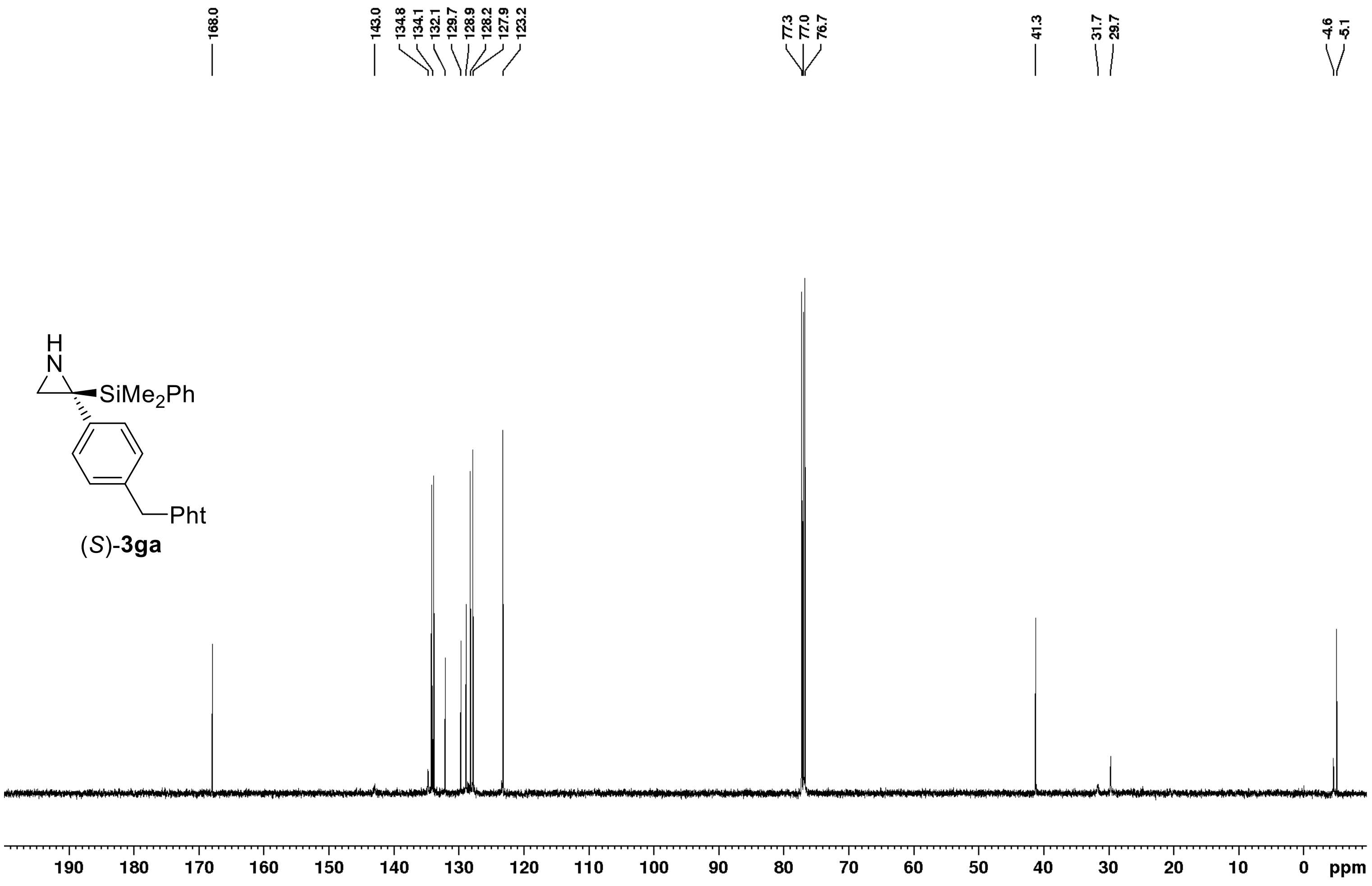

Figure S47. **$^{1}H/^{29}Si$ HMQC NMR** spectrum (500/99 MHz, $CDCl_3$, 298 K, optimized for J = 7 Hz) of **(*S*)-2-(4-(2-(dimethyl(phenyl)silyl)aziridin-2-yl)benzyl)isoindoline-1,3-dione** [(*S*)-**3ga**].

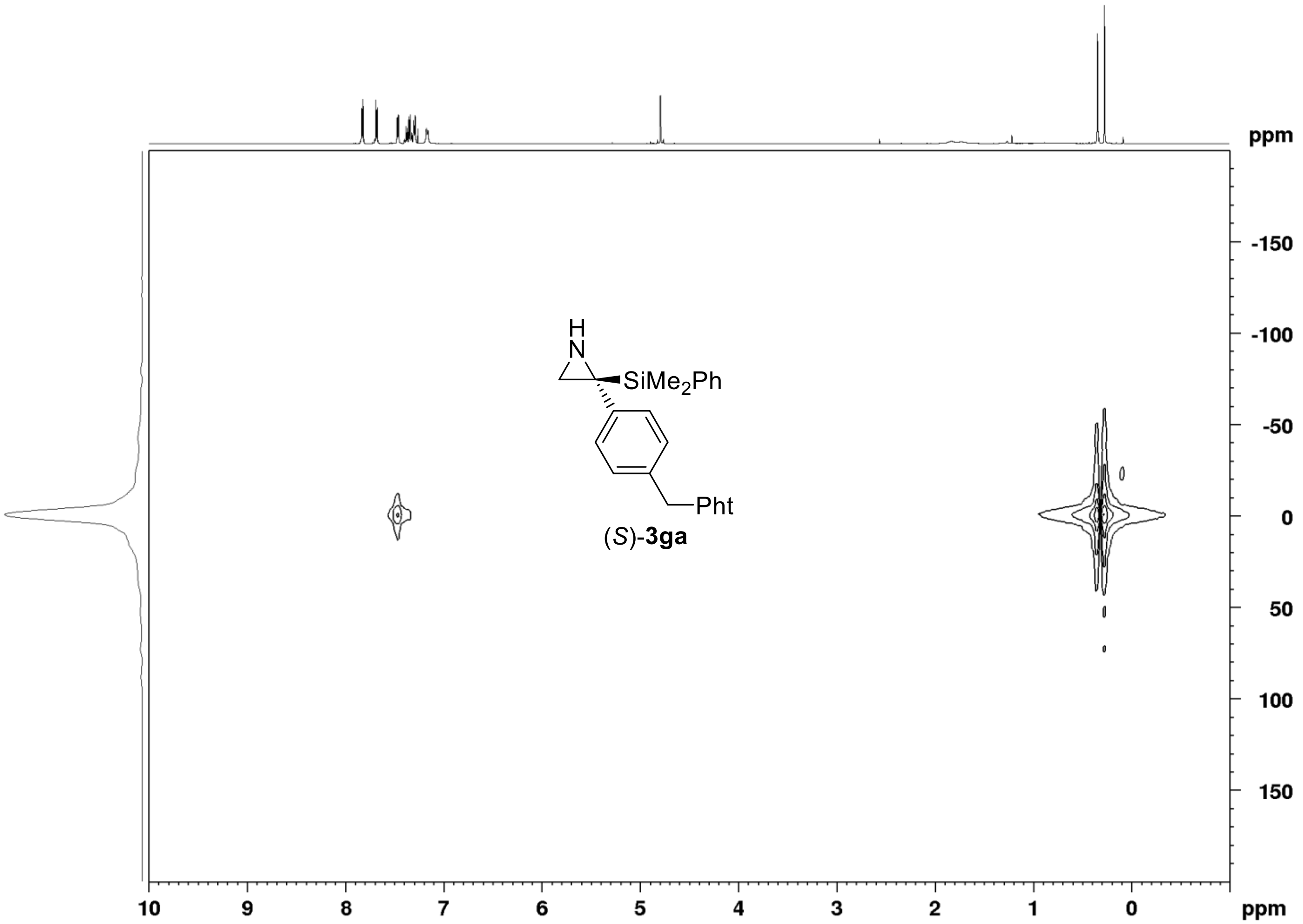

Figure S48. **¹H NMR** (500 MHz, $CDCl_3$, 298 K) of **(*S*)-2-([1,1'-biphenyl]-4-yl)-2-(dimethyl(phenyl)silyl)aziridine** [(*S*)-**3ha**].

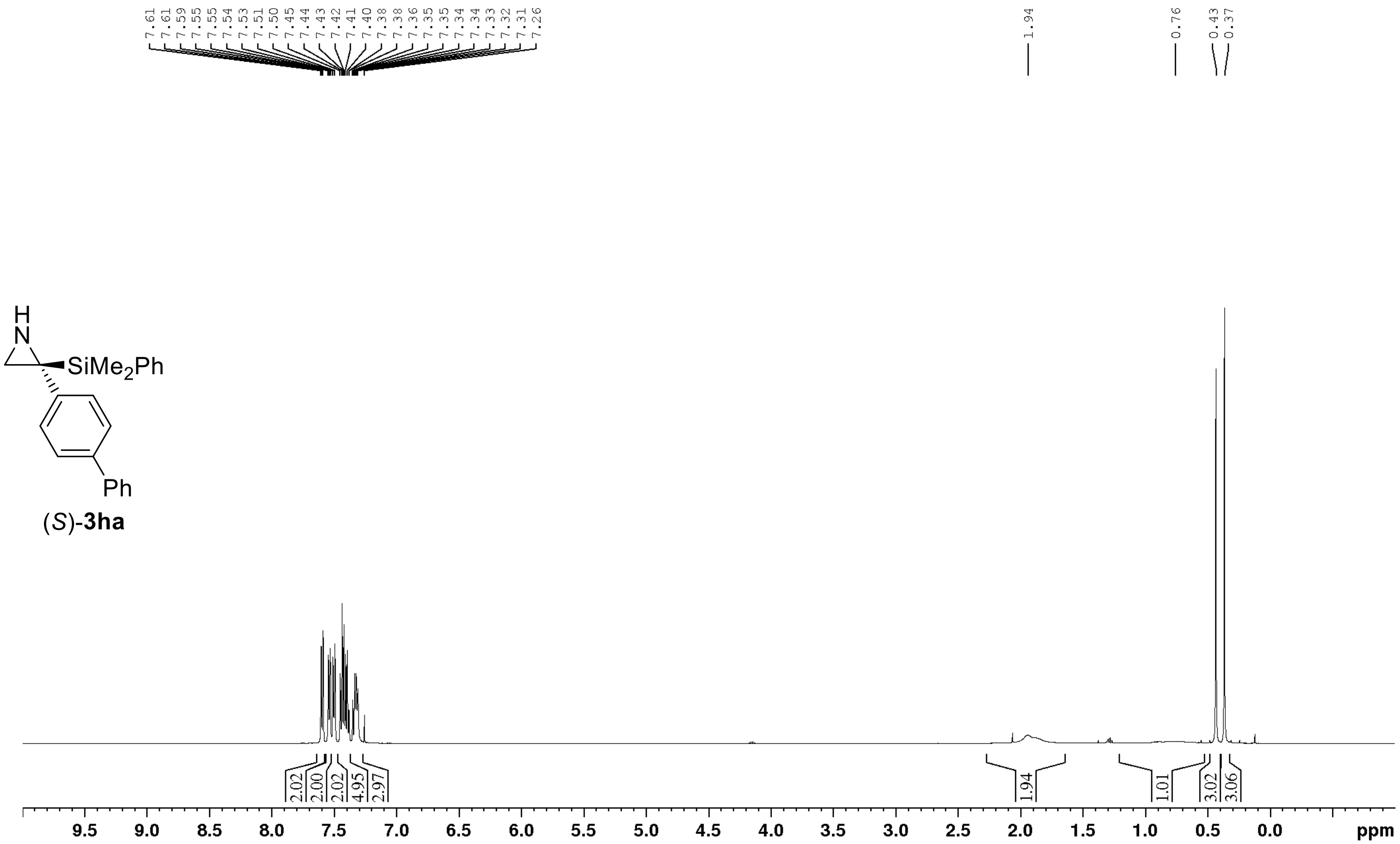

Figure S49. **^{13}C NMR** (126 MHz, $CDCl_3$, 298 K) of **(*S*)-2-([1,1'-biphenyl]-4-yl)-2-(dimethyl(phenyl)silyl)aziridine** [(*S*)-**3ha**].

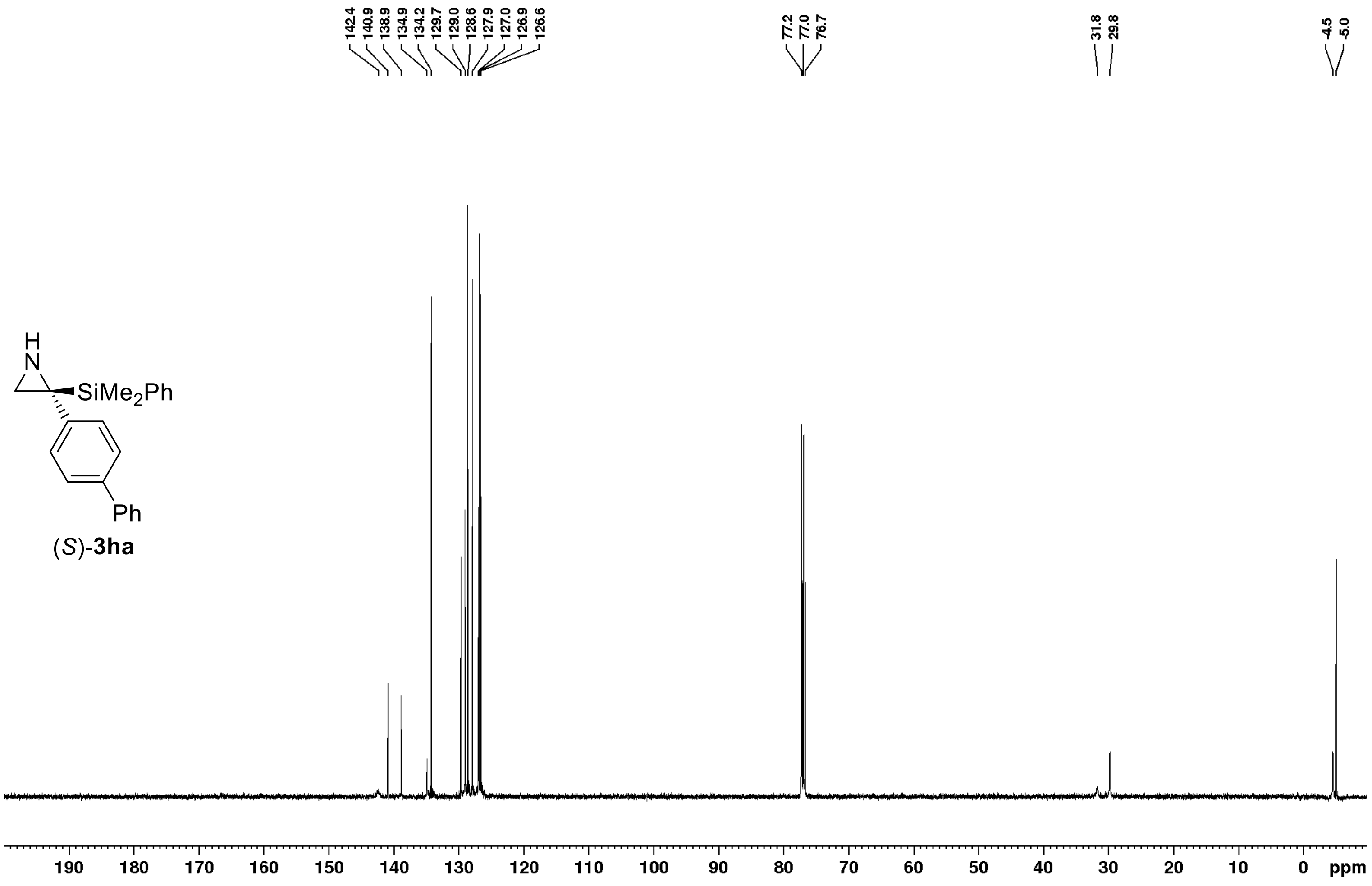

(*S*)-**3ha**

Figure S50. **^{1}H/^{29}Si HMQC NMR** spectrum (500/99 MHz, $CDCl_3$, 298 K, optimized for *J* = 7 Hz) of **(*S*)-2-([1,1'-biphenyl]-4-yl)-2-(dimethyl(phenyl)silyl)aziridine** [(*S*)-**3ha**].

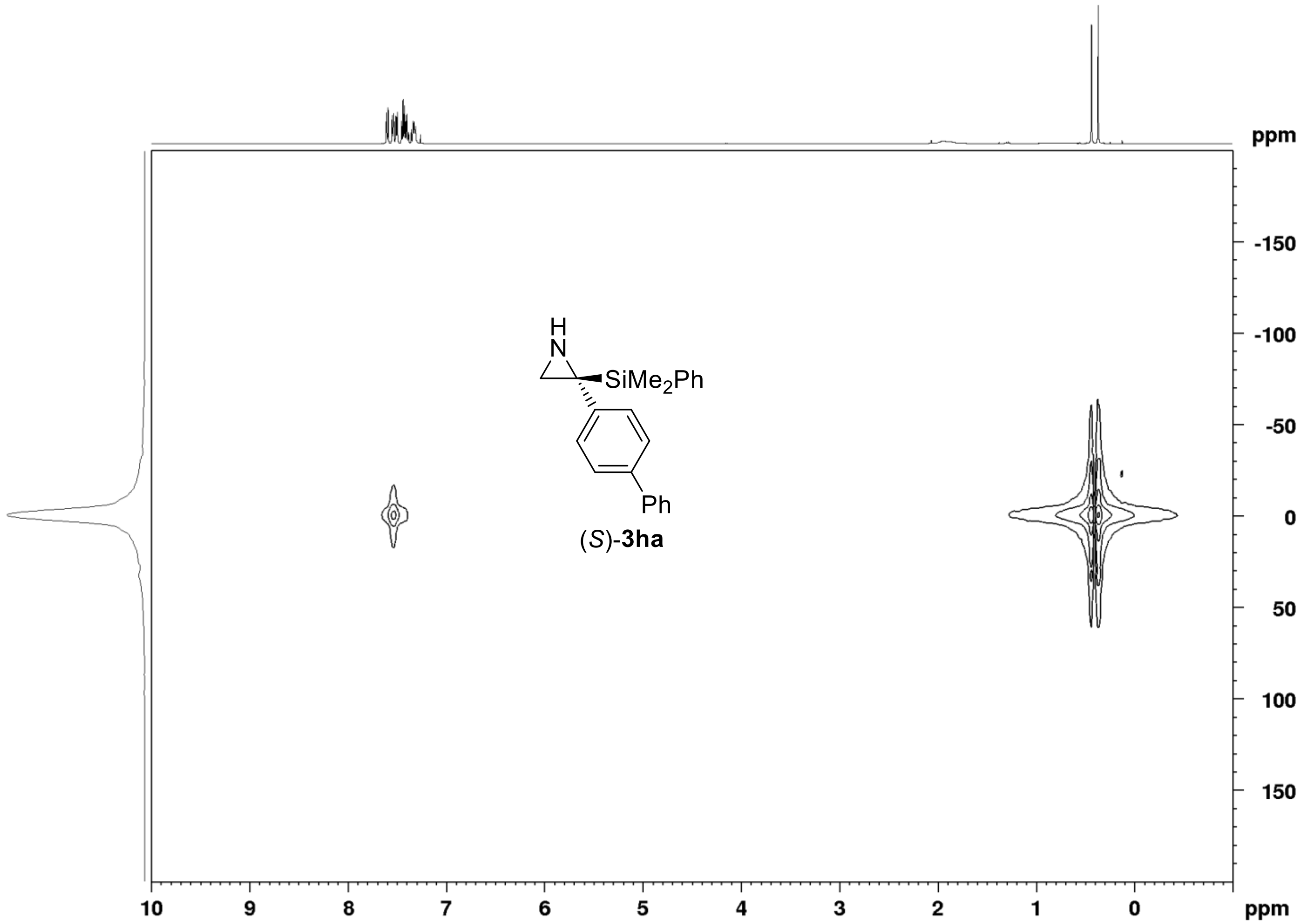

Figure S51. **^{1}H NMR** (500 MHz, $CDCl_3$, 298 K) of **(*S*)-2-(dimethyl(phenyl)silyl)-2-(naphthalen-2-yl)aziridine** [(*S*)-**3ia**].

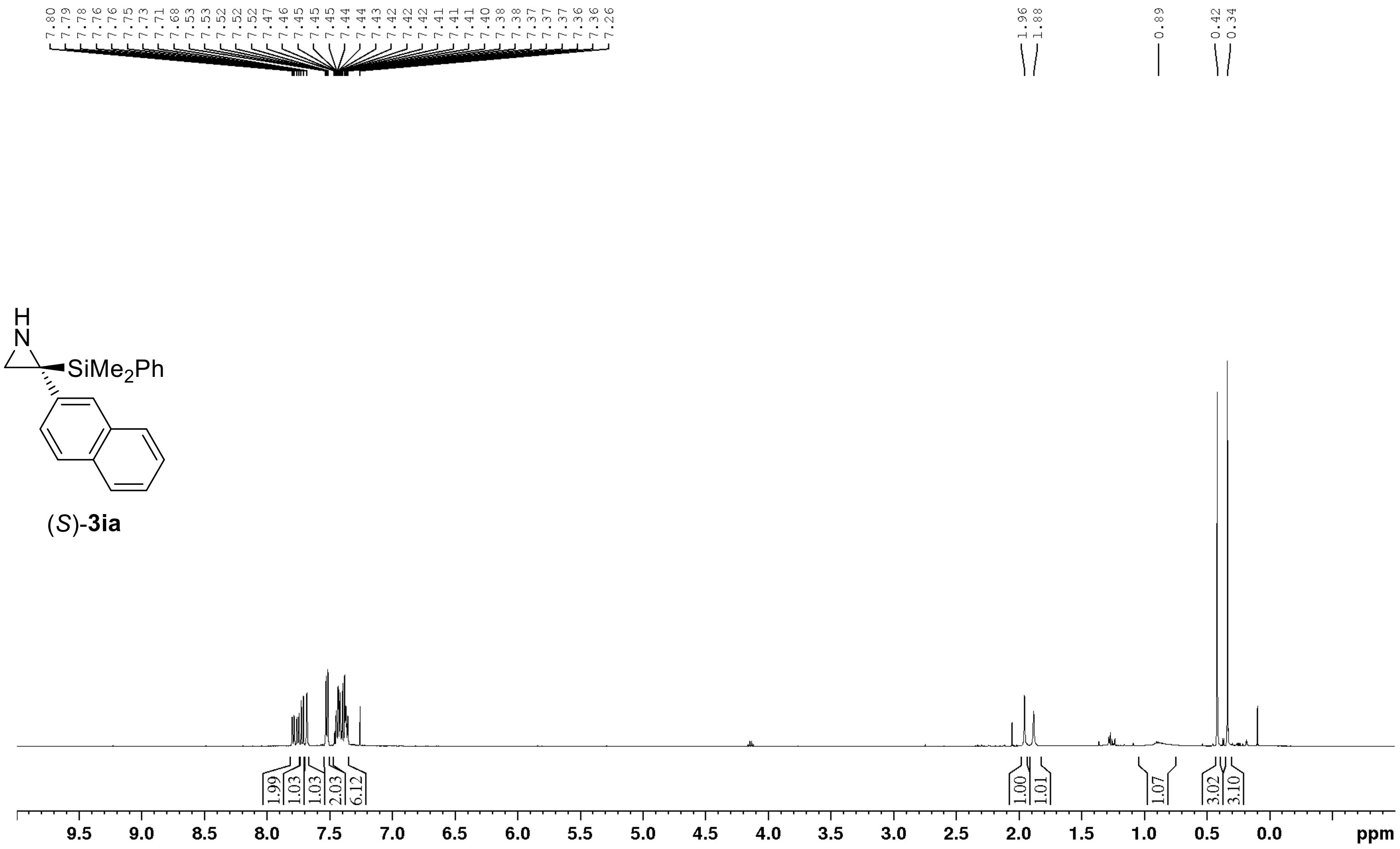

Figure S52. **^{13}C NMR** (126 MHz, $CDCl_3$, 298 K) of **(*S*)-2-(dimethyl(phenyl)silyl)-2-(naphthalen-2-yl)aziridine** [(*S*)-**3ia**].

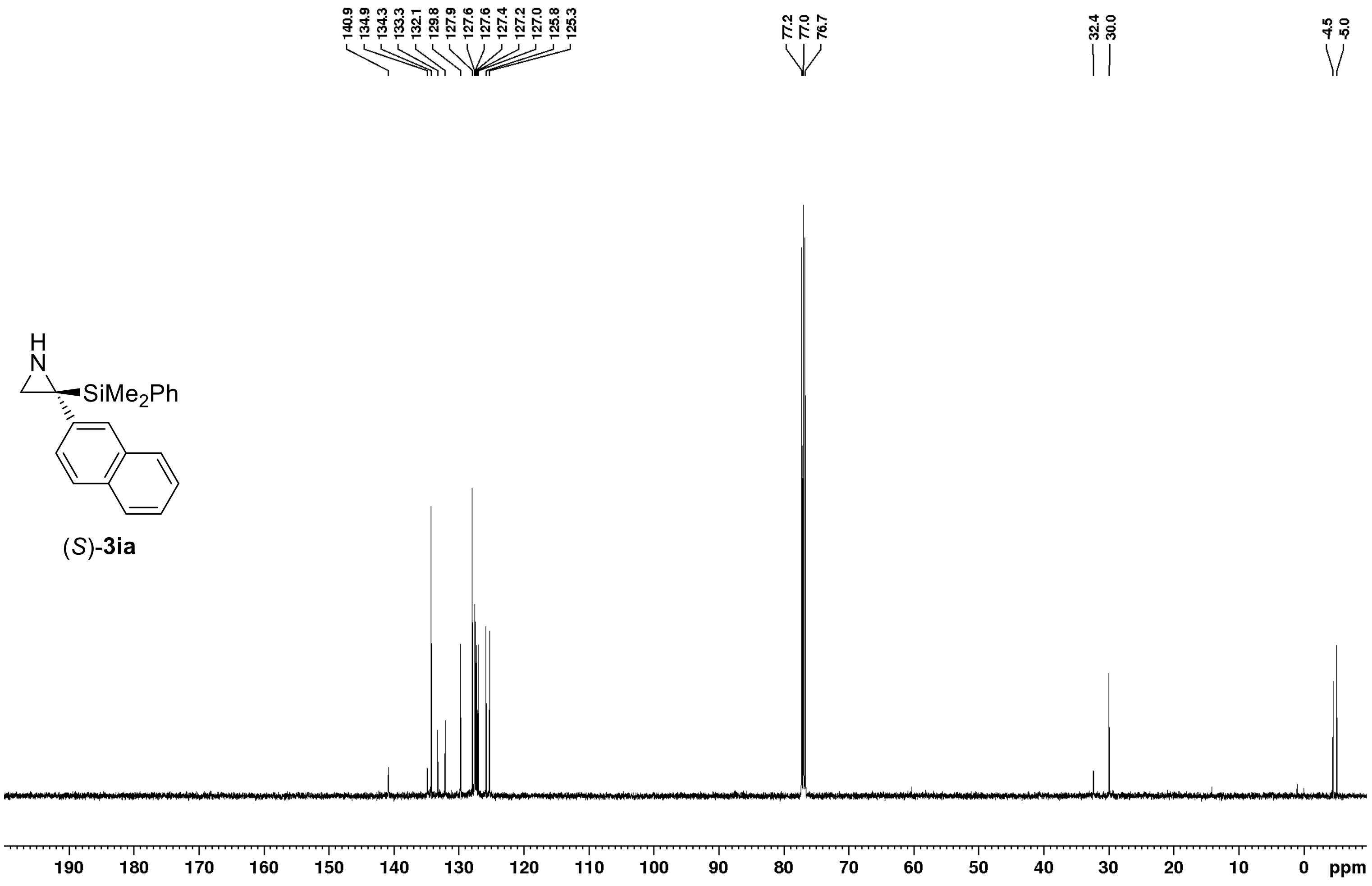

Figure S53. **$^{1}H/^{29}Si$ HMQC NMR** spectrum (500/99 MHz, $CDCl_3$, 298 K, optimized for *J* = 7 Hz) of **(*S*)-2-(dimethyl(phenyl)silyl)-2-(naphthalen-2-yl)aziridine** [(*S*)-**3ia**].

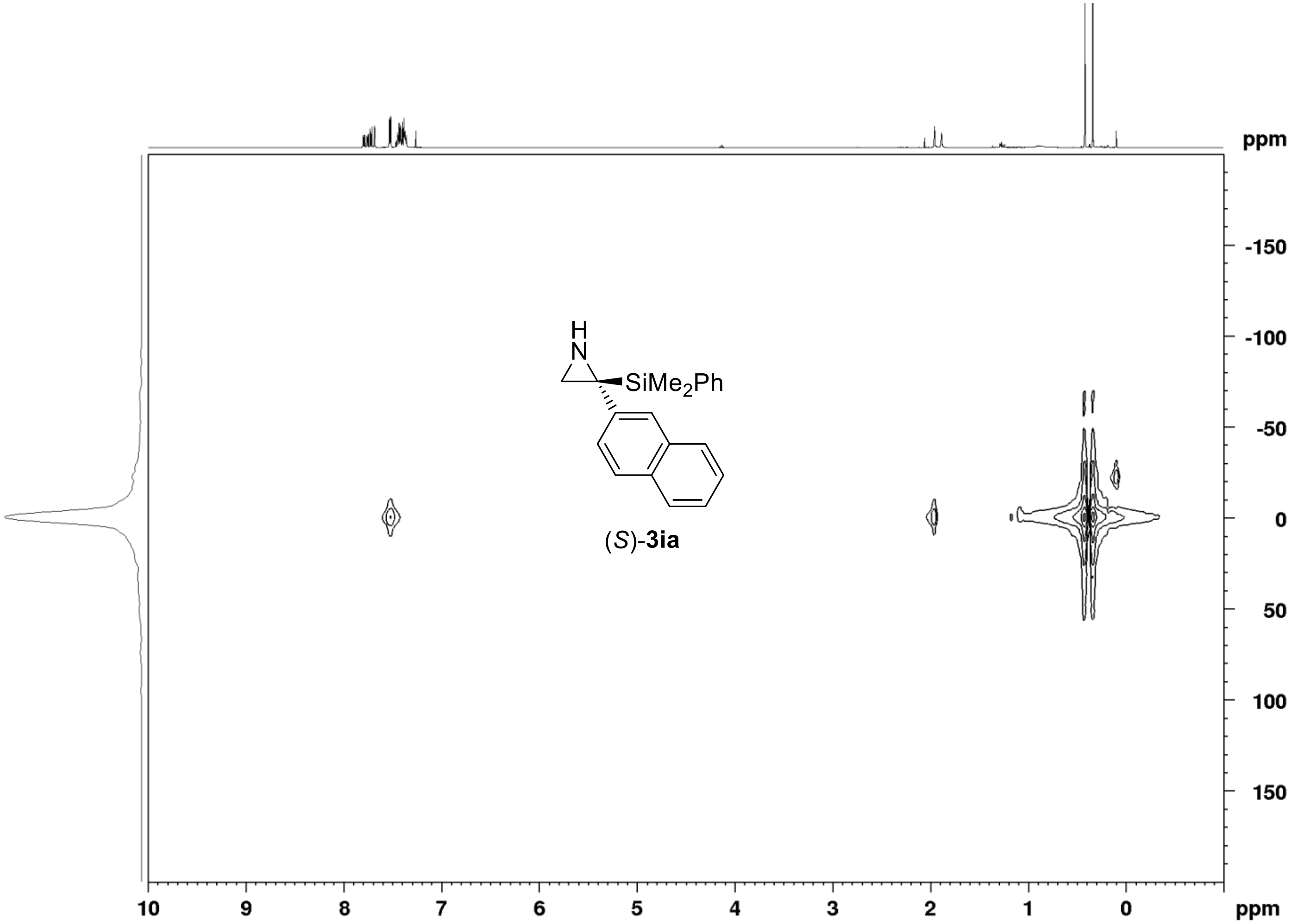

Figure S54. **^{1}H NMR** (500 MHz, $CDCl_3$, 298 K) of **(*S*)-2-(dimethyl(phenyl)silyl)-2-(3-methoxyphenyl)aziridine** [(*S*)-**3ja**].

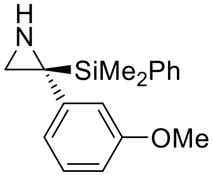

(*S*)-**3ja**

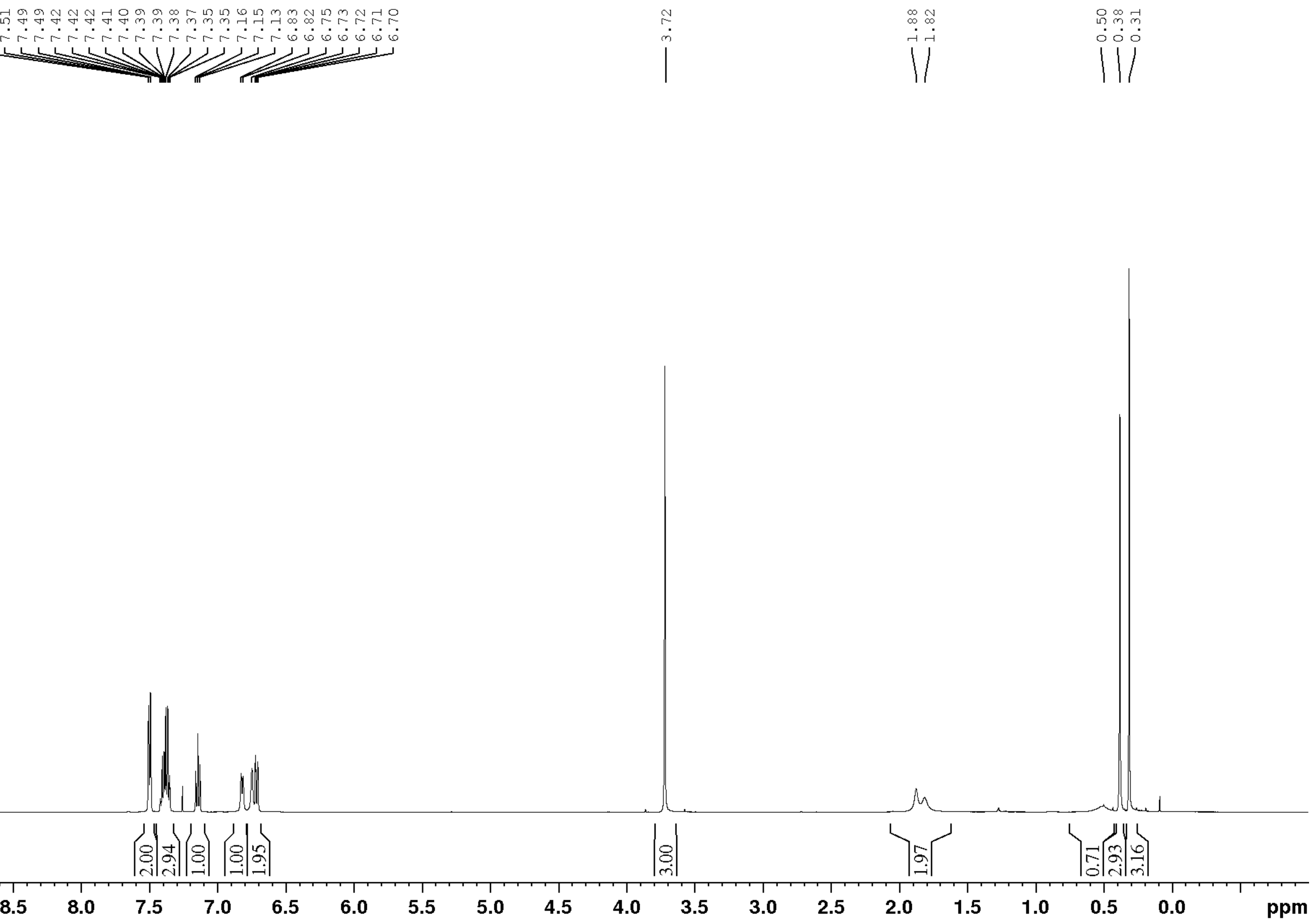

Figure S55. **^{13}C NMR** (126 MHz, $CDCl_3$, 298 K) of **(*S*)-2-(dimethyl(phenyl)silyl)-2-(3-methoxyphenyl)aziridine** [(*S*)-**3ja**].

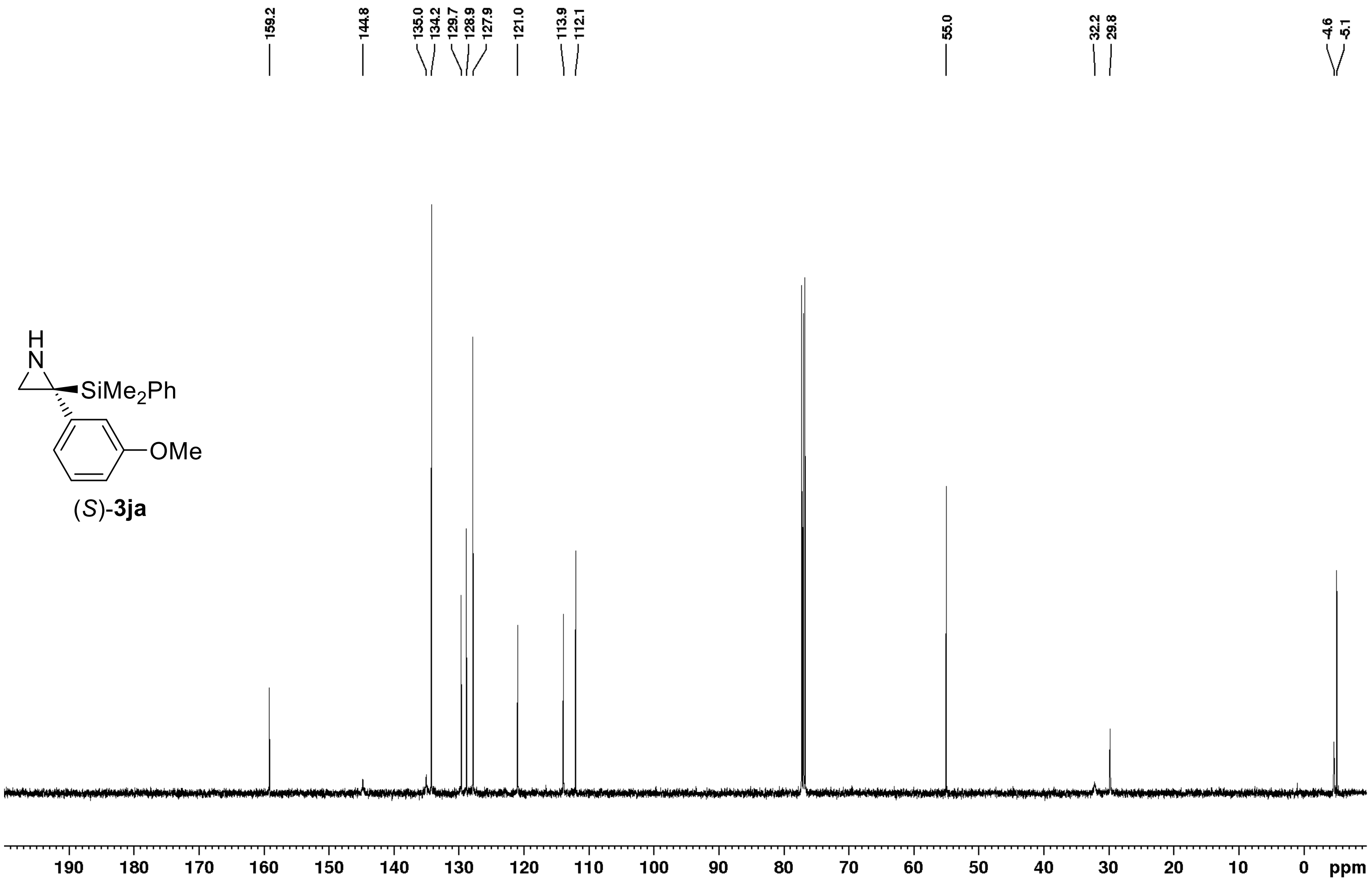

Figure S56. **^{29}Si DEPT NMR** (99 MHz, CDCl$_3$, 298 K) of **(*S*)-2-(dimethyl(phenyl)silyl)-2-(3-methoxyphenyl)aziridine** [(*S*)-**3ja**].

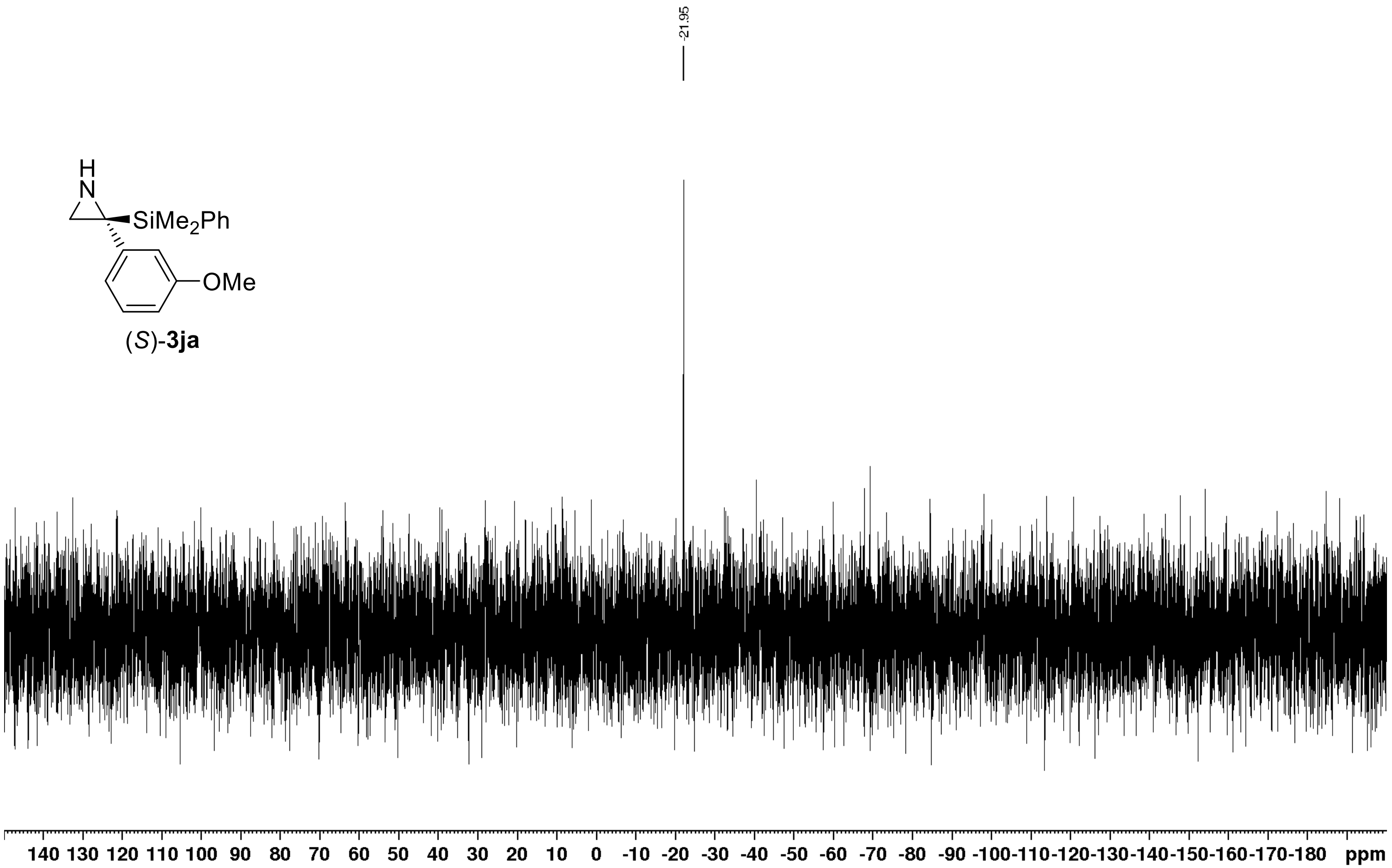

Figure S57. **^{1}H NMR** (500 MHz, $CDCl_3$, 298 K) of **(*S*)-2-(dimethyl(phenyl)silyl)-2-(4-methoxyphenyl)aziridine** [(*S*)-**3ka**].

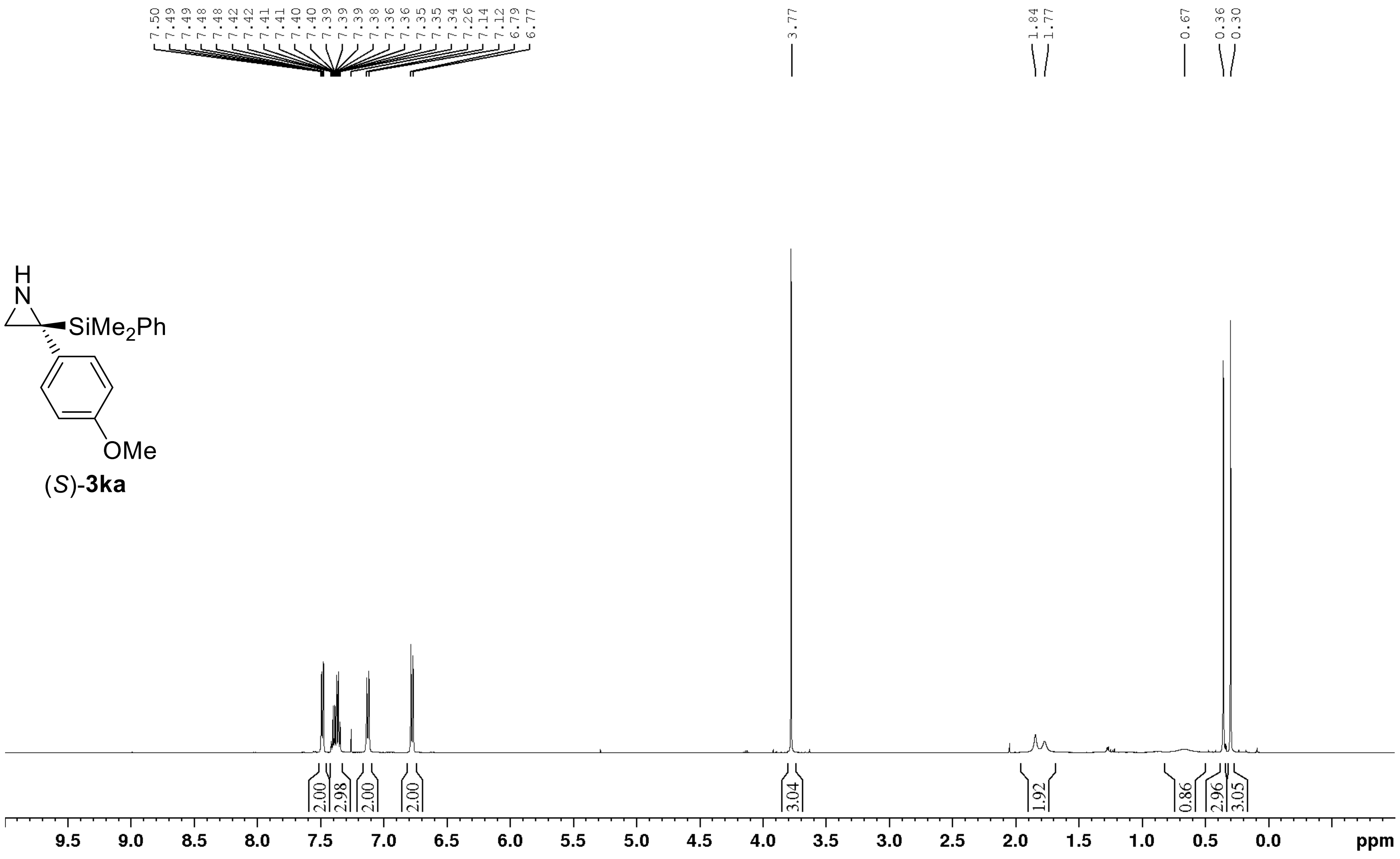

Figure S58. **^{13}C NMR** (126 MHz, $CDCl_3$, 298 K) of **(*S*)-2-(dimethyl(phenyl)silyl)-2-(4-methoxyphenyl)aziridine** [(*S*)-**3ka**].

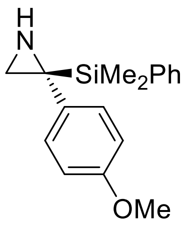

(*S*)-**3ka**

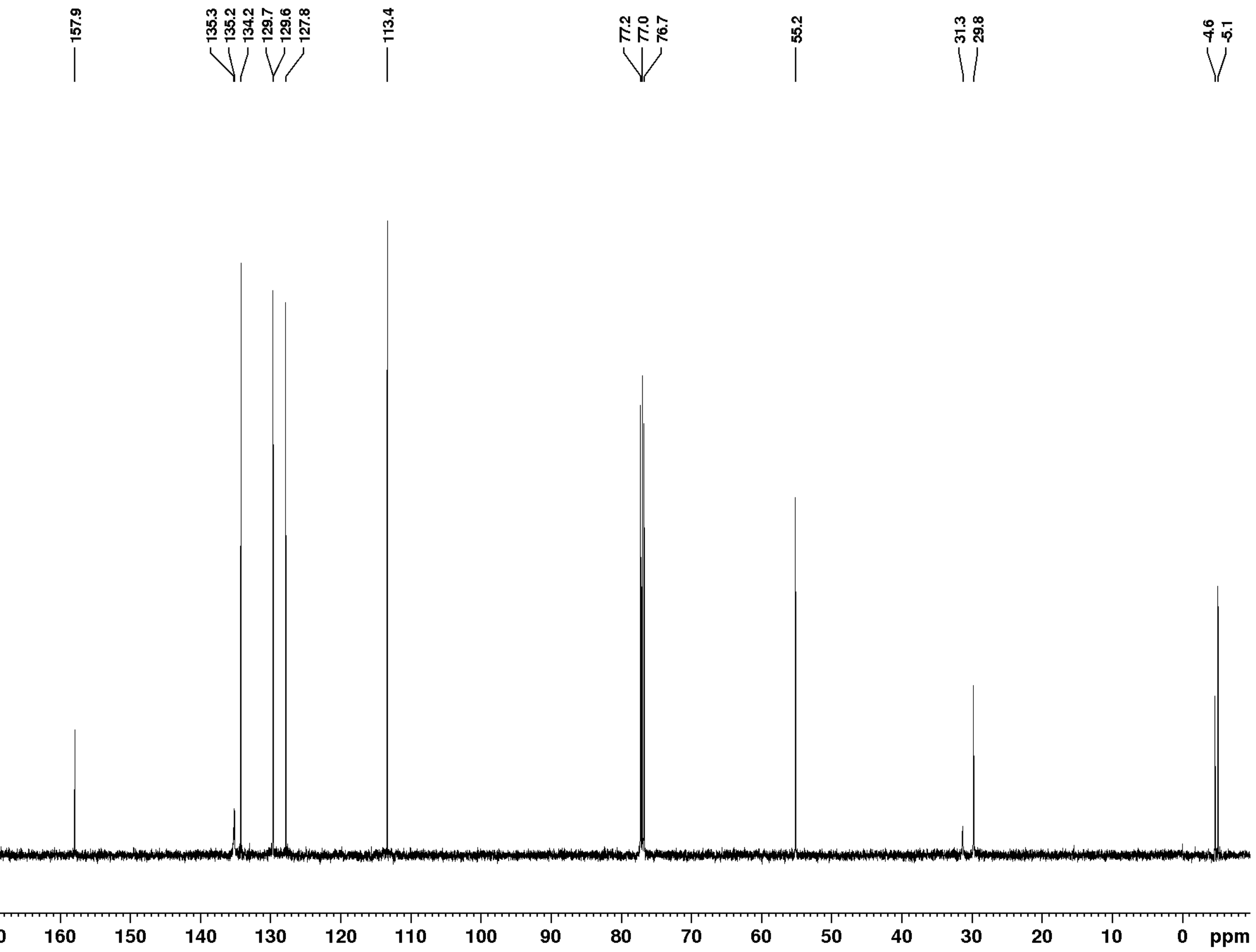

Figure S59. **$^{1}H/^{29}Si$ HMQC NMR** spectrum (500/99 MHz, $CDCl_3$, 298 K, optimized for J = 7 Hz) of **(*S*)-2-(dimethyl(phenyl)silyl)-2-(4-methoxyphenyl)aziridine** [(*S*)-**3ka**].

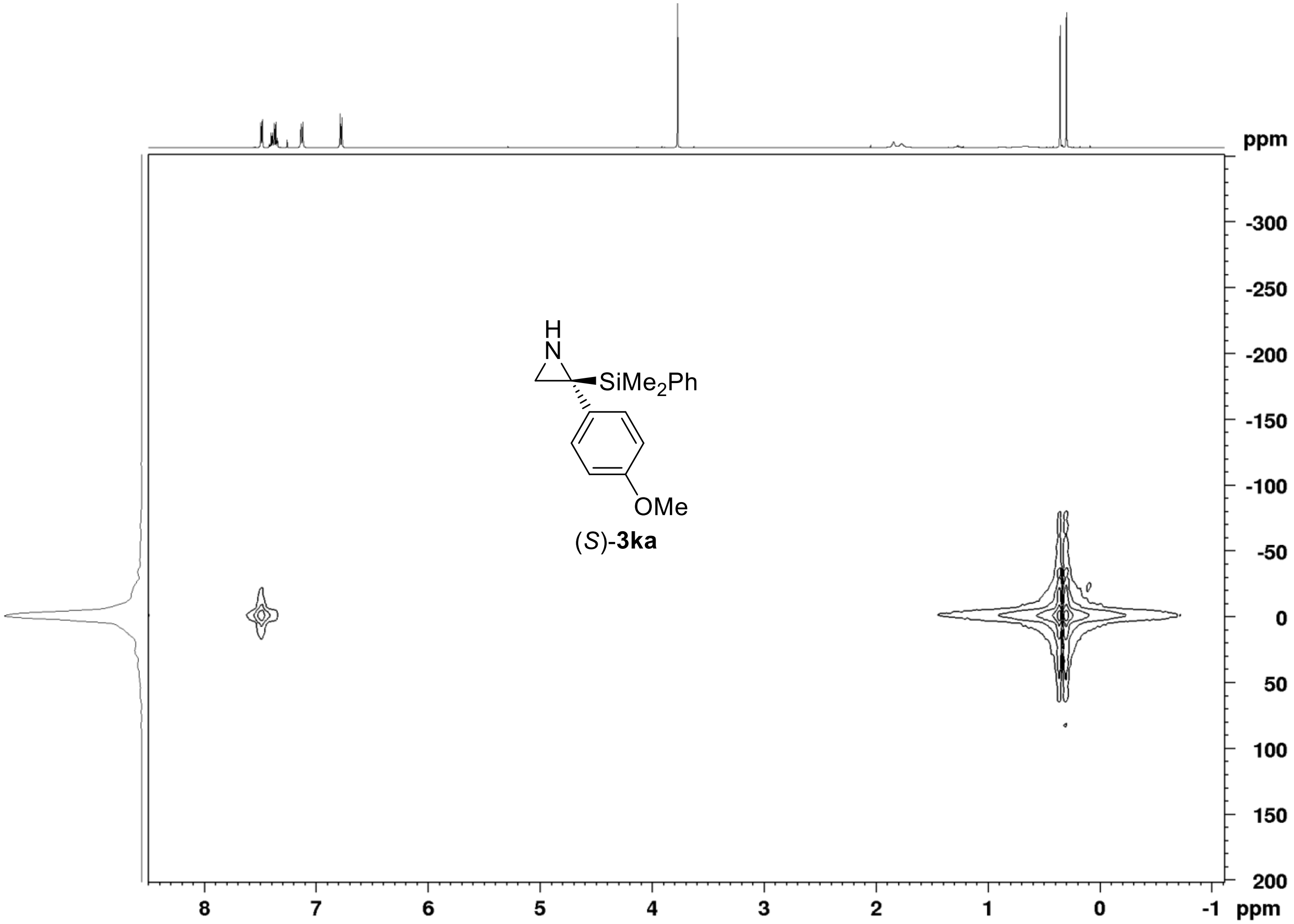

Figure S60. **^{1}H NMR** (500 MHz, $CDCl_3$, 298 K) of **(*S*)-2-(3-(benzyloxy)phenyl)-2-(dimethyl(phenyl)silyl)aziridine** [(*S*)-**3la**].

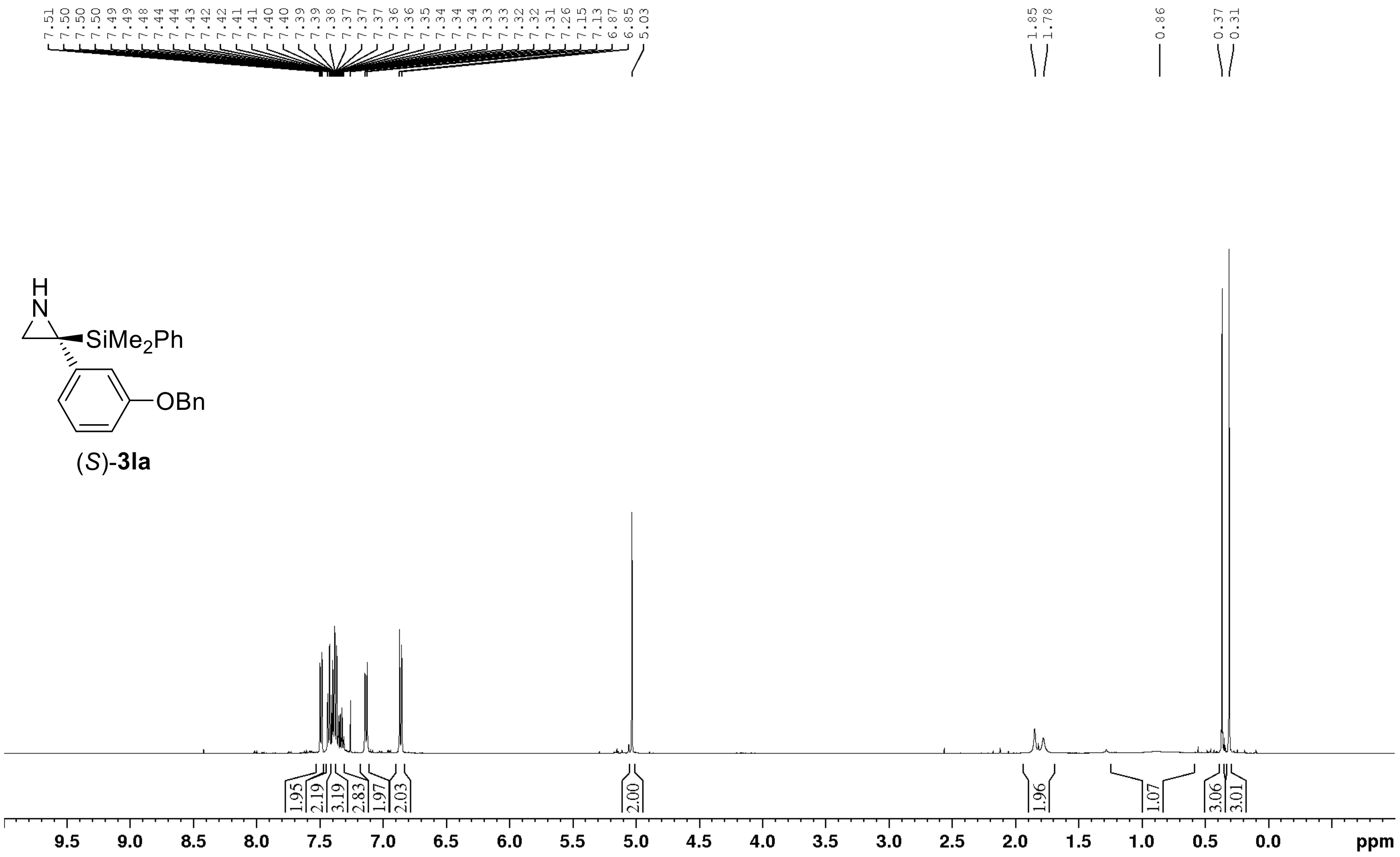

Figure S61. **^{13}C NMR** (126 MHz, $CDCl_3$, 298 K) of **(*S*)-2-(3-(benzyloxy)phenyl)-2-(dimethyl(phenyl)silyl)aziridine** [(*S*)-**3la**].

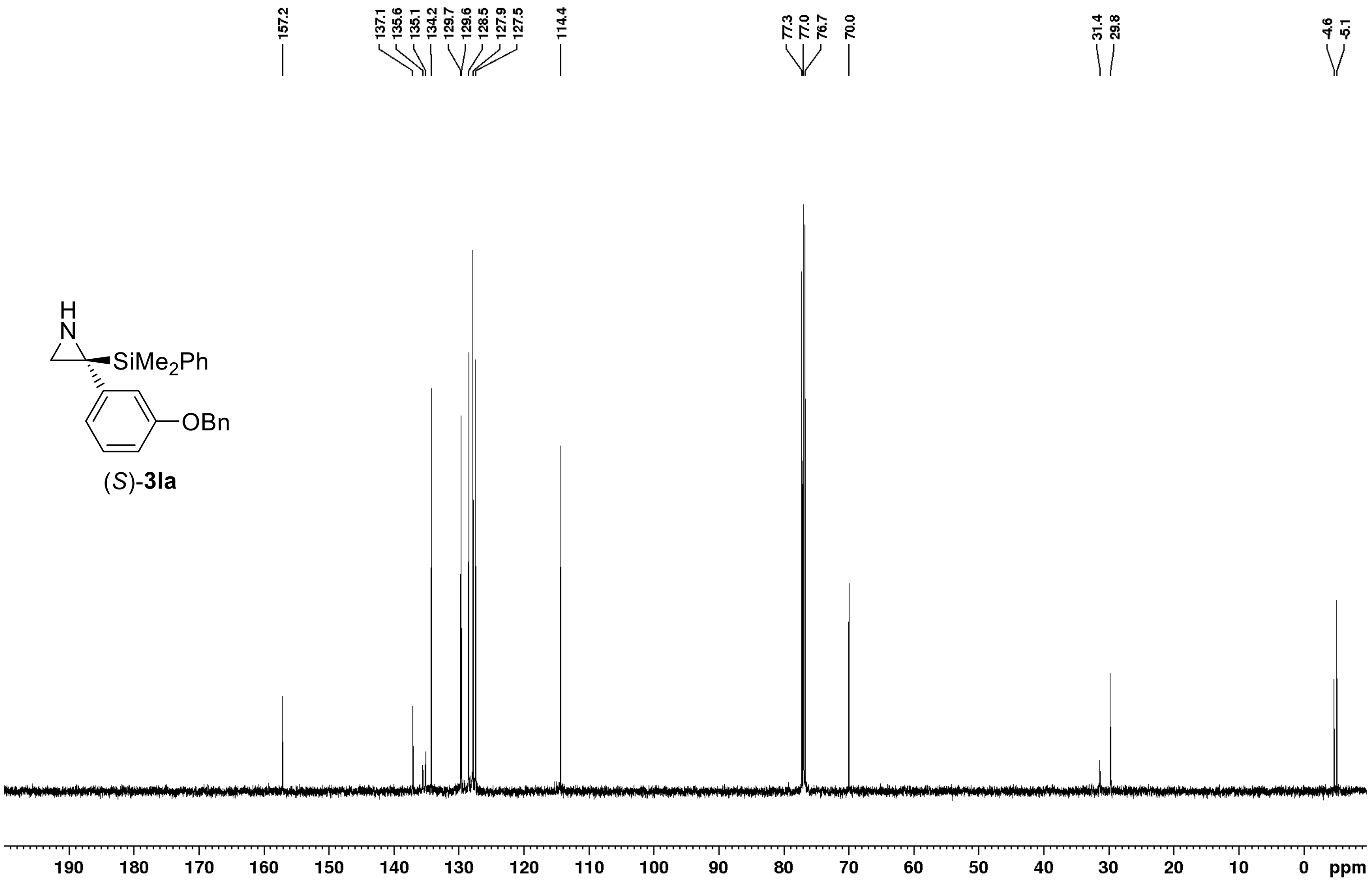

Figure S62. **$^{1}H/^{29}Si$ HMQC NMR** spectrum (500/99 MHz, $CDCl_3$, 298 K, optimized for *J* = 7 Hz) of **(*S*)-2-(3-(benzyloxy)phenyl)-2-(dimethyl(phenyl)silyl)aziridine** [(*S*)-**3la**].

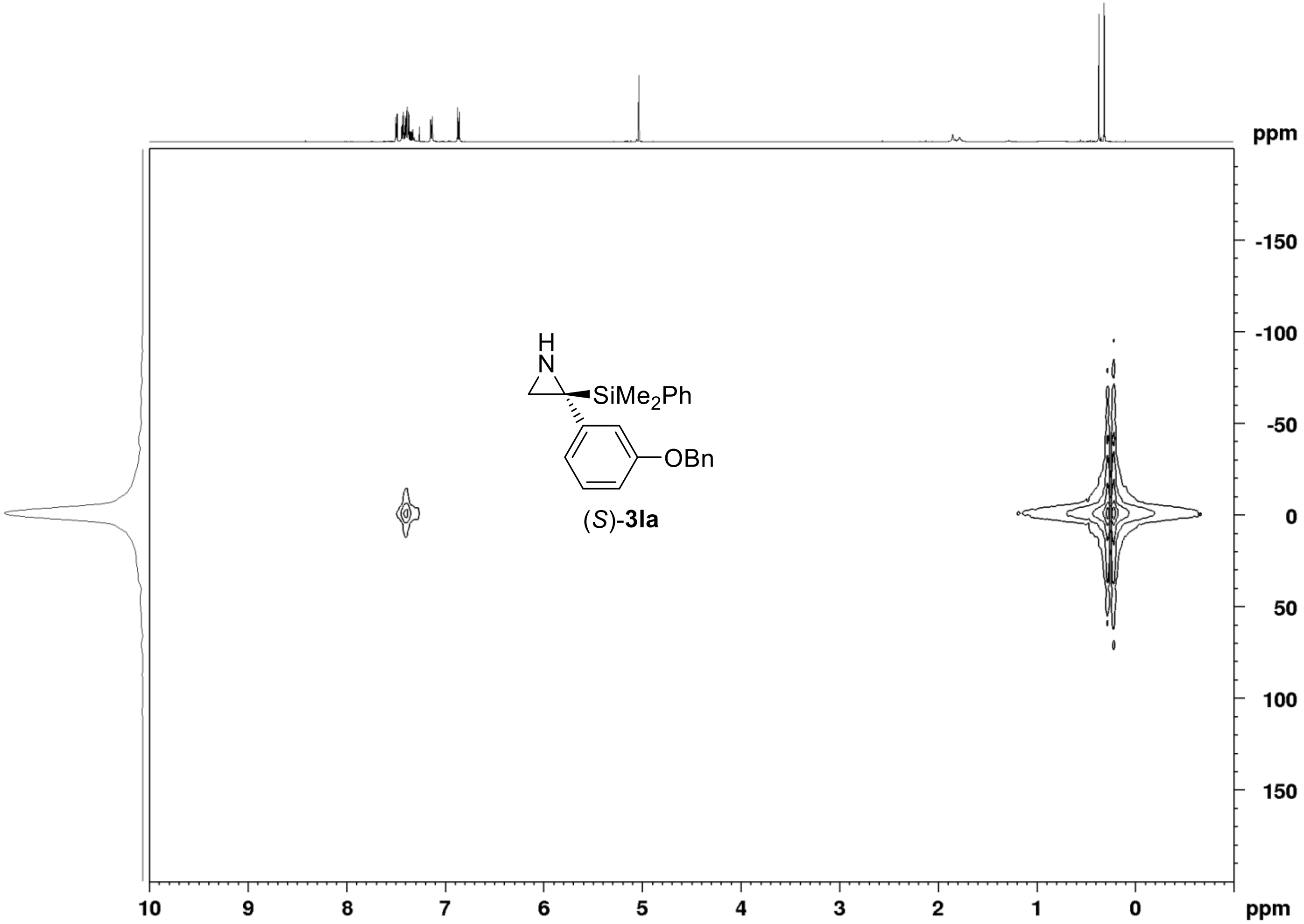

Figure S63. **^{1}H NMR** (500 MHz, $CDCl_3$, 298 K) of **(*S*)-2-(4-(benzyloxy)phenyl)-2-(dimethyl(phenyl)silyl)aziridine** [(*S*)-**3ma**].

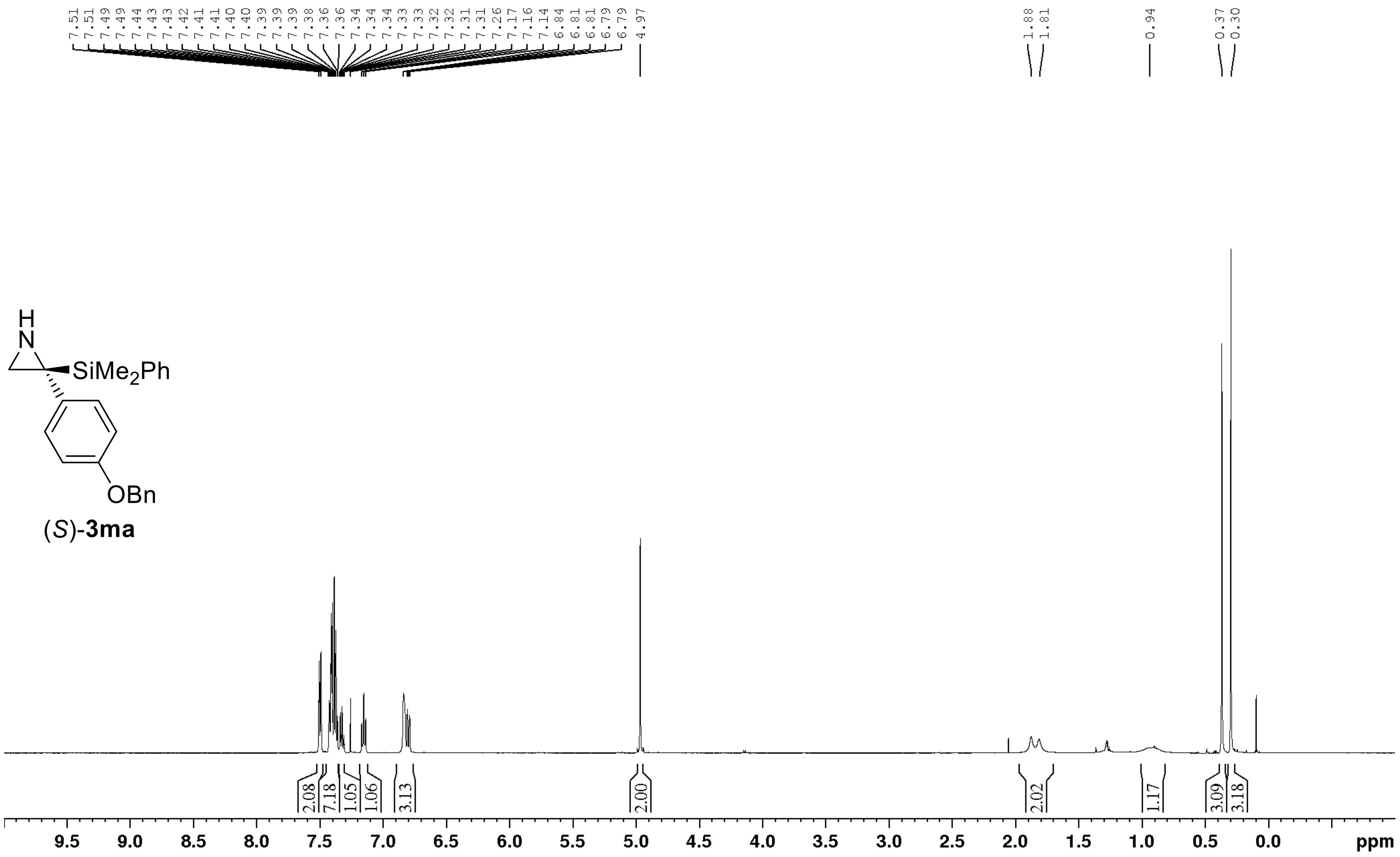

Figure S64. **^{13}C NMR** (126 MHz, $CDCl_3$, 298 K) of **(*S*)-2-(4-(benzyloxy)phenyl)-2-(dimethyl(phenyl)silyl)aziridine** [(*S*)-**3ma**].

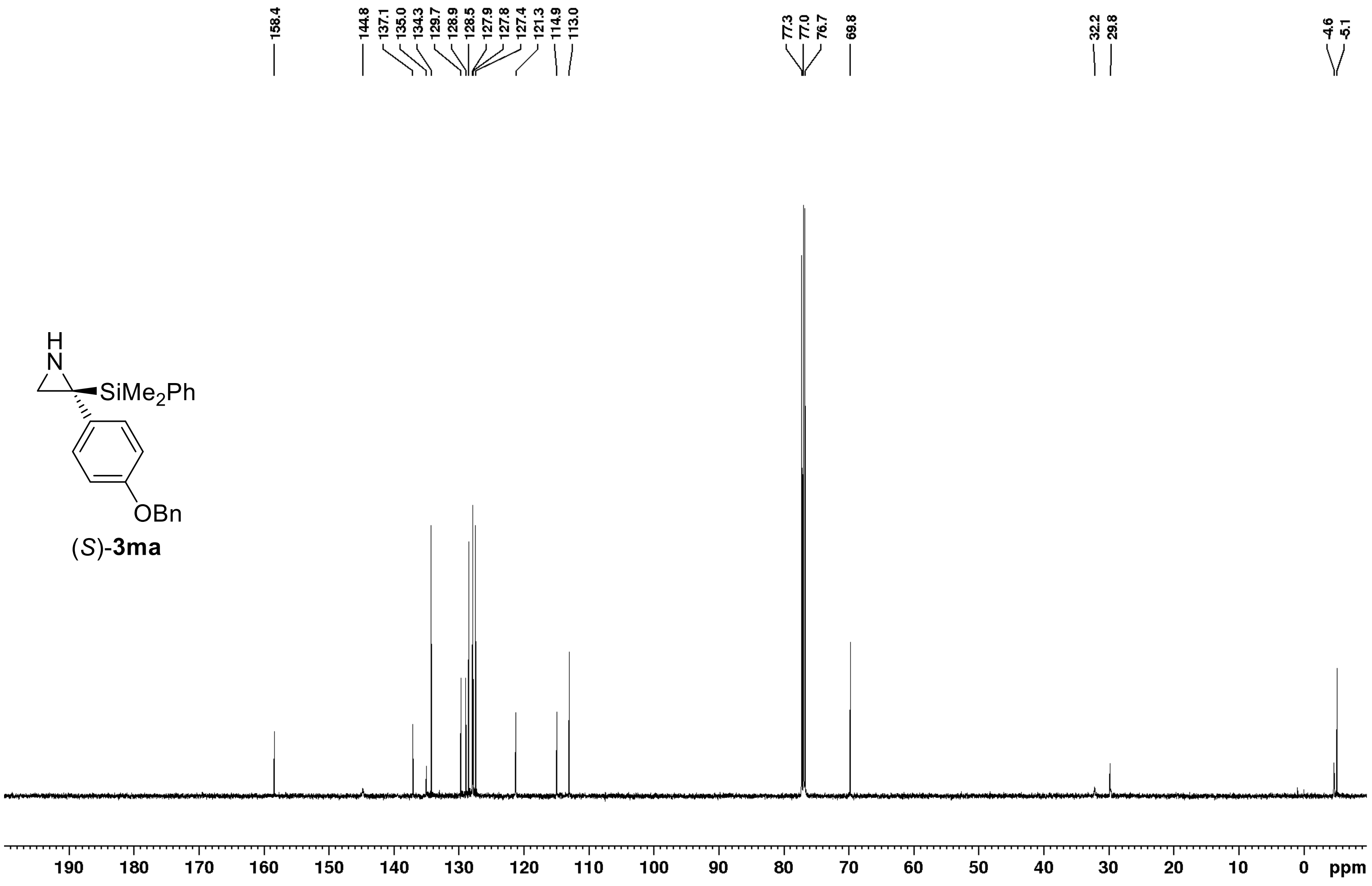

Figure S65. **^{1}H/^{29}Si HMQC NMR** spectrum (500/99 MHz, $CDCl_3$, 298 K, optimized for J = 7 Hz) of **(*S*)-2-(4-(benzyloxy)phenyl)-2-(dimethyl(phenyl)silyl)aziridine** [(*S*)-**3ma**].

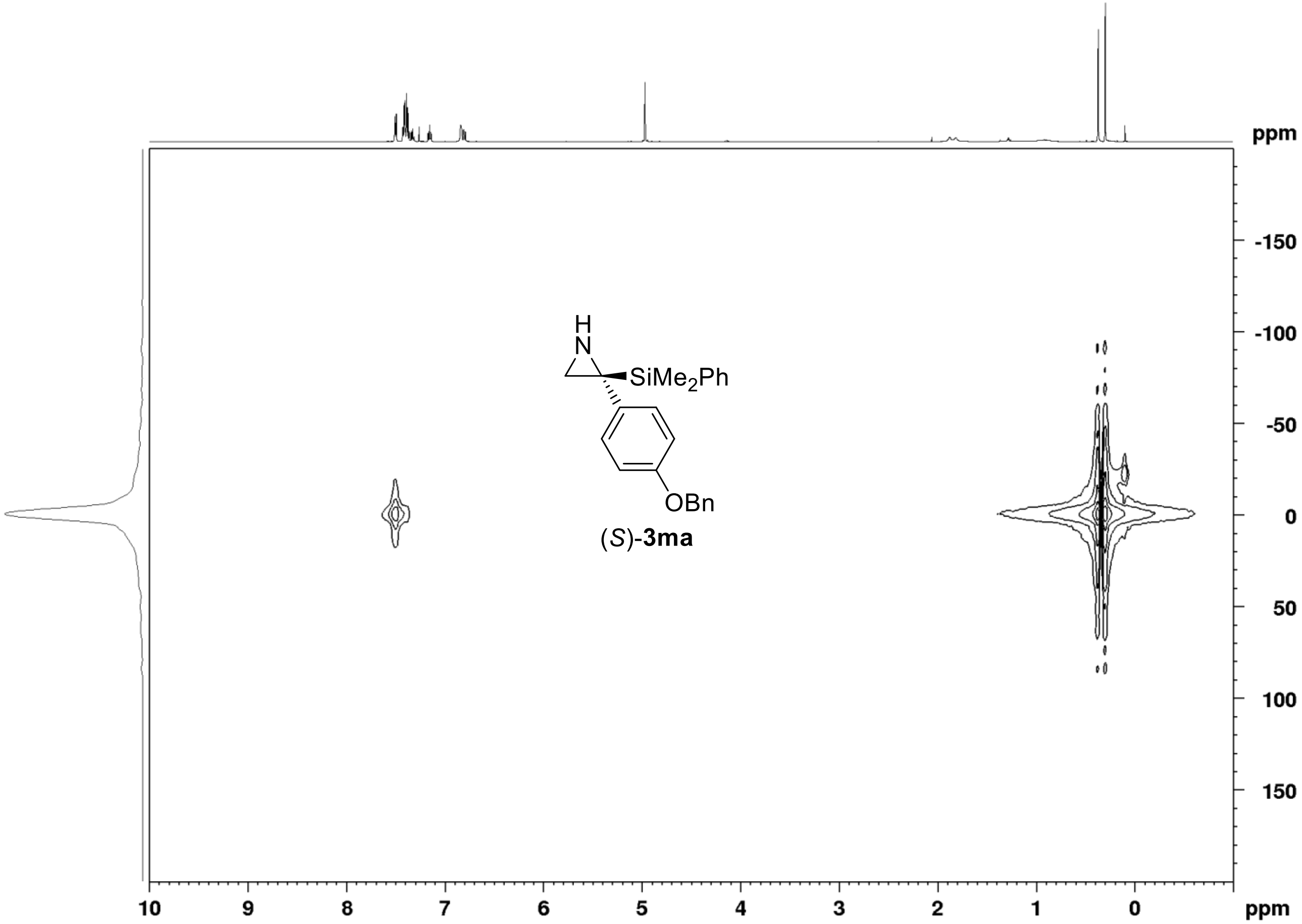

Figure S66. **^{1}H NMR** (500 MHz, $CDCl_3$, 298 K) of **(*S*)-2-(benzo[d][1,3]dioxol-5-yl)-2-(dimethyl(phenyl)silyl)aziridine** [(*S*)-**3na**].

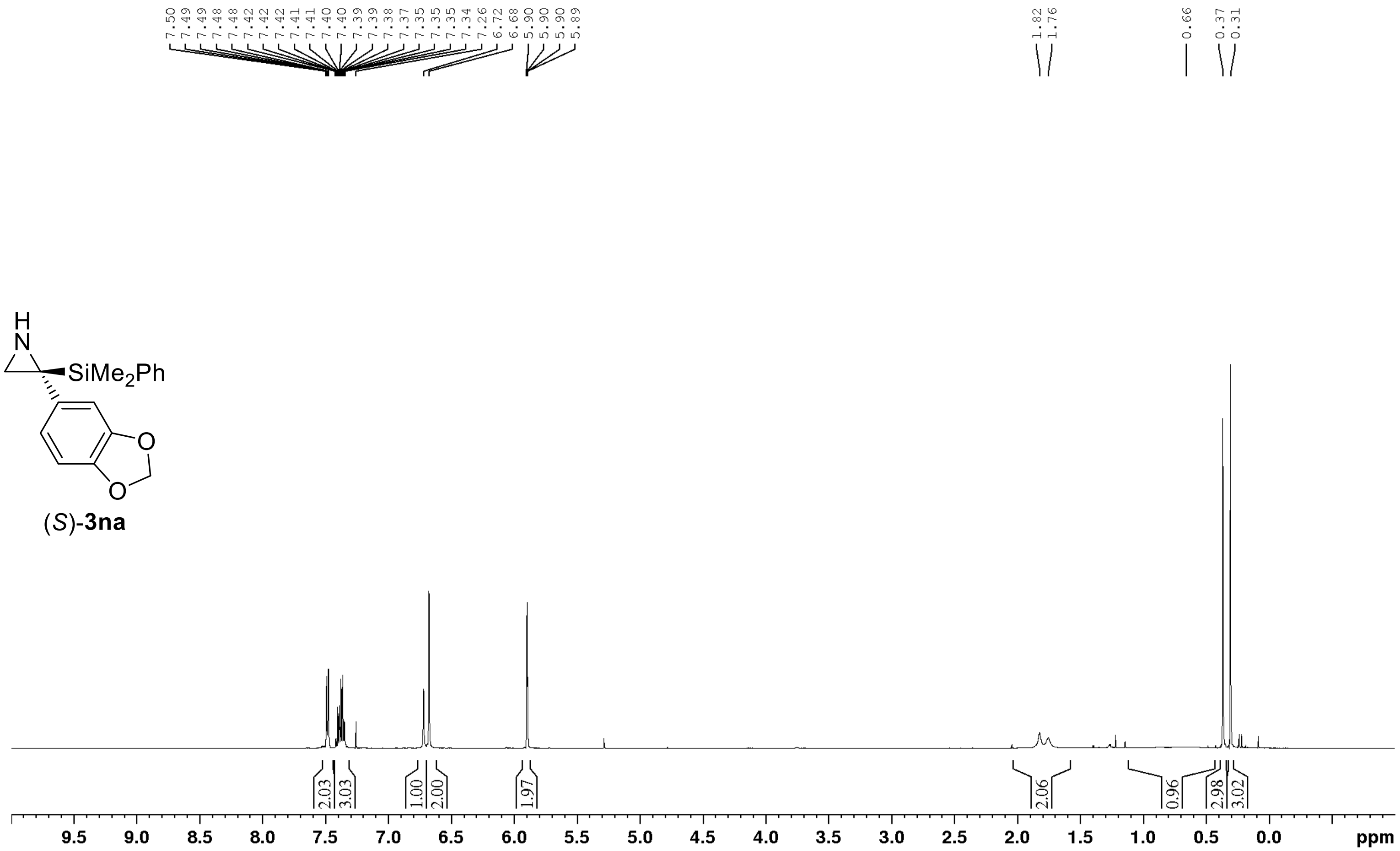

Figure S67. **^{13}C NMR** (126 MHz, $CDCl_3$, 298 K) of **(*S*)-2-(benzo[d][1,3]dioxol-5-yl)-2-(dimethyl(phenyl)silyl)aziridine** [(*S*)-**3na**].

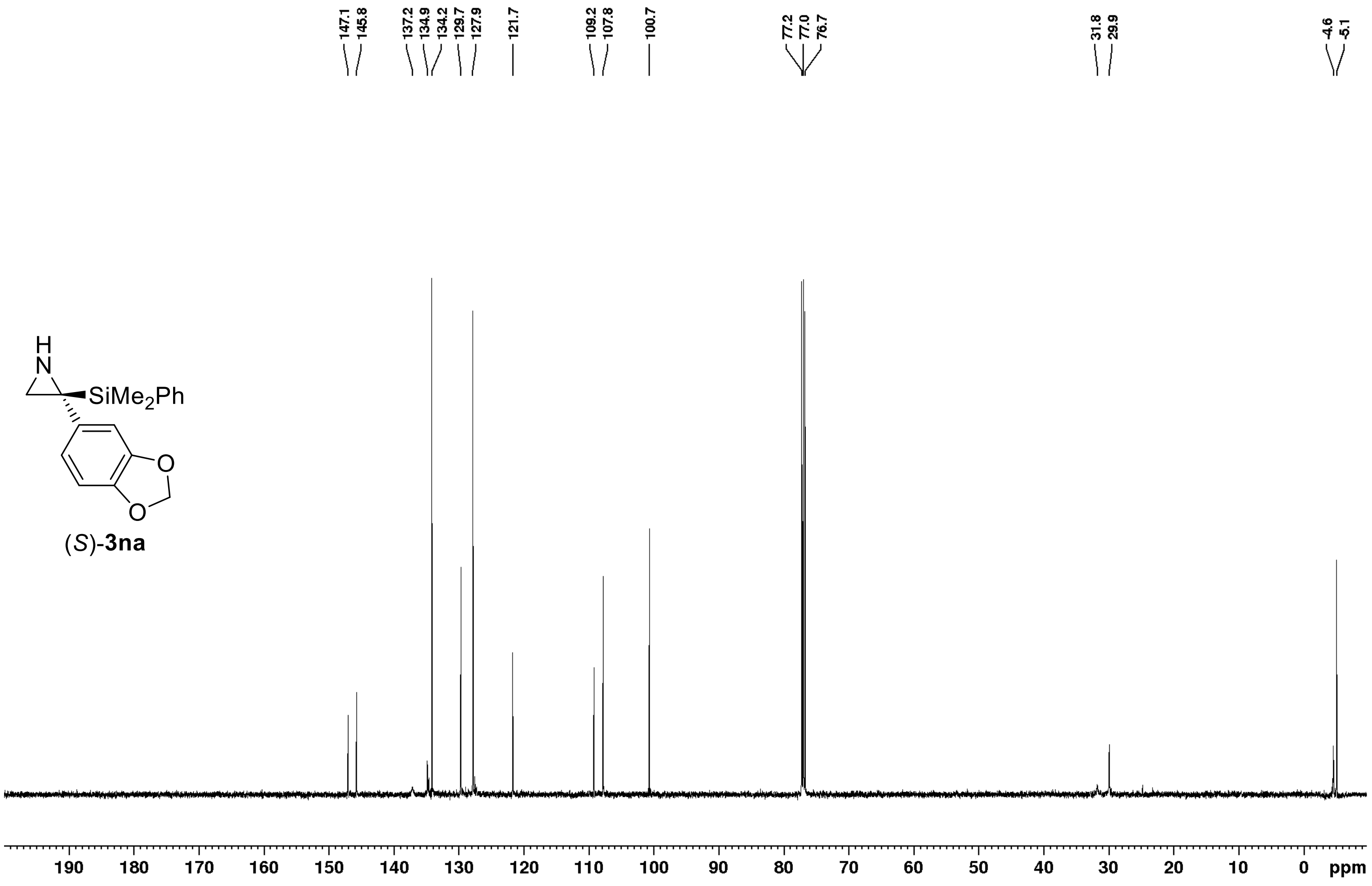

Figure S68. **^{1}H/^{29}Si HMQC NMR** spectrum (500/99 MHz, $CDCl_3$, 298 K, optimized for *J* = 7 Hz) of **(*S*)-2-(benzo[d][1,3]dioxol-5-yl)-2-(dimethyl(phenyl)silyl)aziridine** [(*S*)-**3na**].

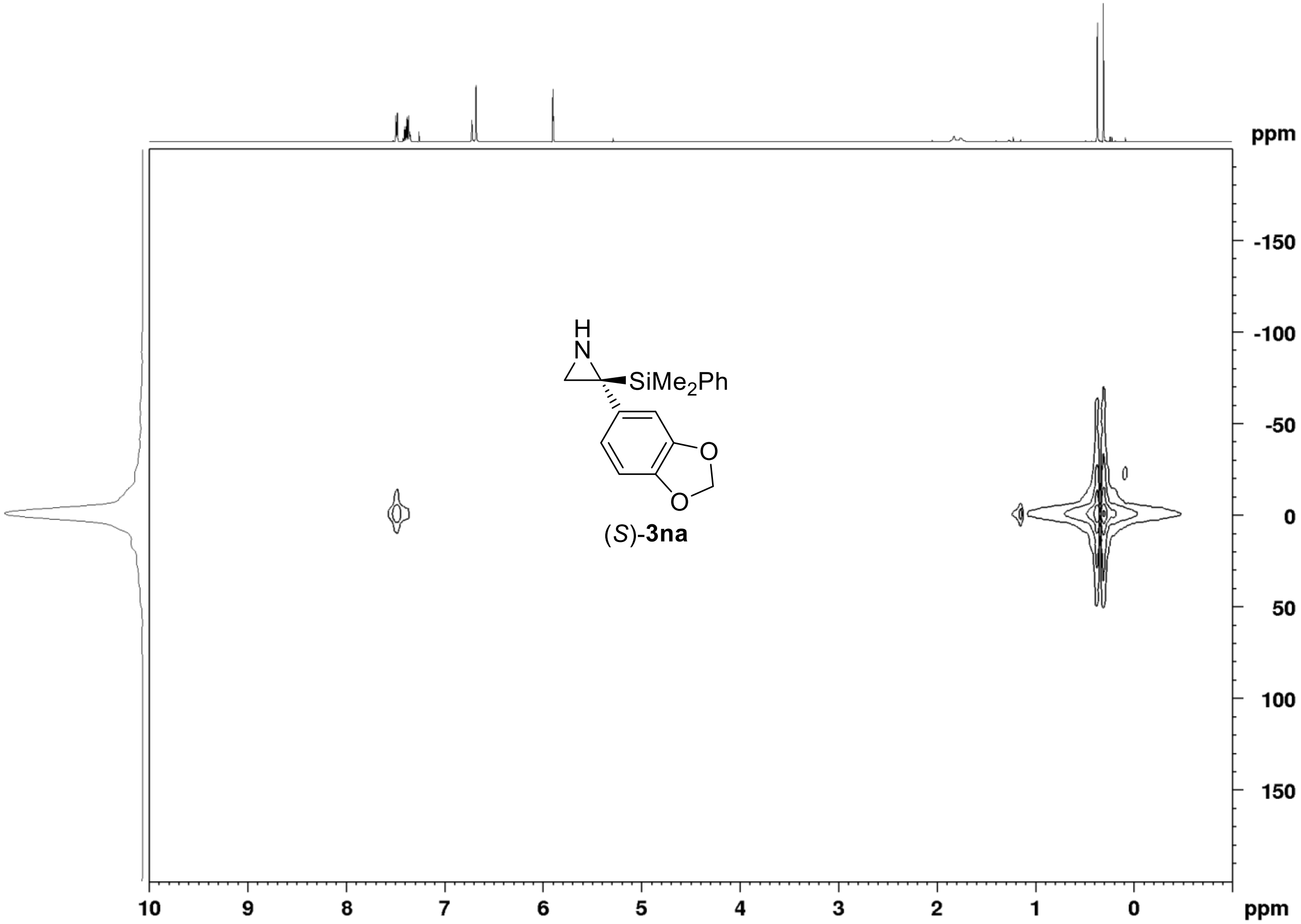

Figure S69. **^{1}H NMR** (500 MHz, $CDCl_3$, 298 K) of **(*S*)-2-(dimethyl(phenyl)silyl)-2-(4-(methylthio)phenyl)aziridine** [(*S*)-**3oa**].

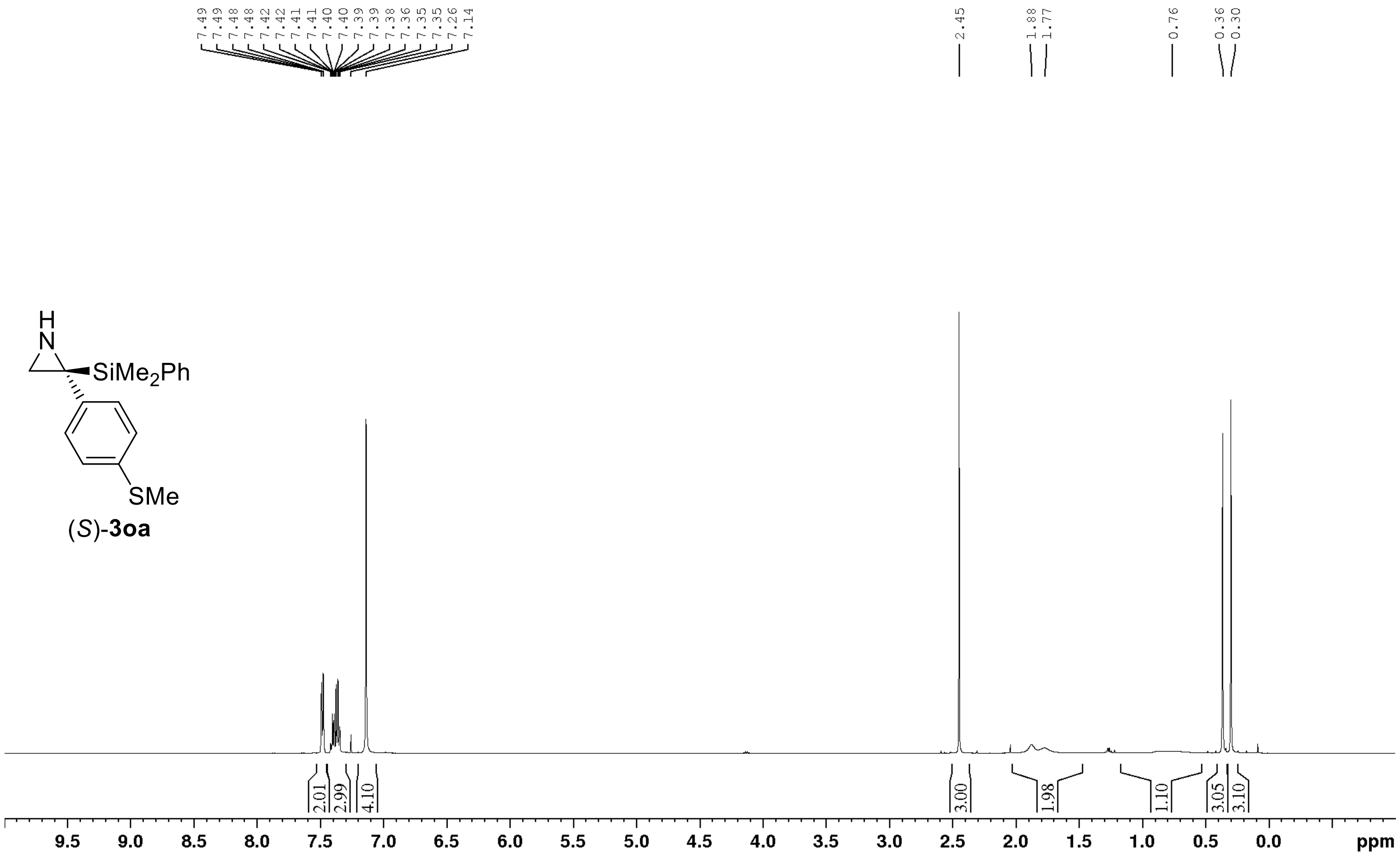

Figure S70. **^{13}C NMR** (126 MHz, $CDCl_3$, 298 K) of **(*S*)-2-(dimethyl(phenyl)silyl)-2-(4-(methylthio)phenyl)aziridine** [(*S*)-**3oa**].

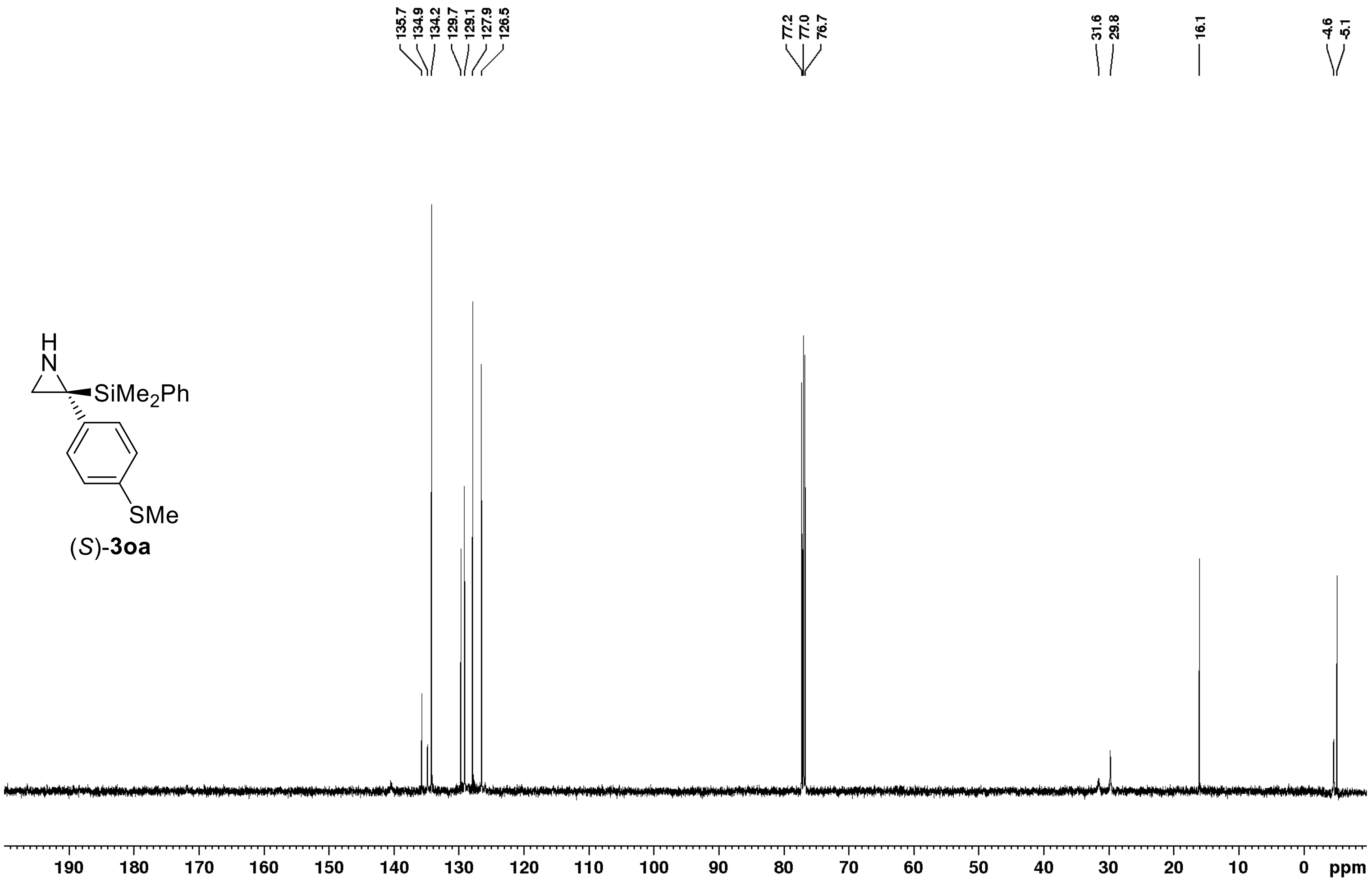

Figure S71. **$^{1}H/^{29}Si$ HMQC NMR** spectrum (500/99 MHz, $CDCl_3$, 298 K, optimized for *J* = 7 Hz) of **(*S*)-2-(dimethyl(phenyl)silyl)-2-(4-(methylthio)phenyl)aziridine** [(*S*)-**3oa**].

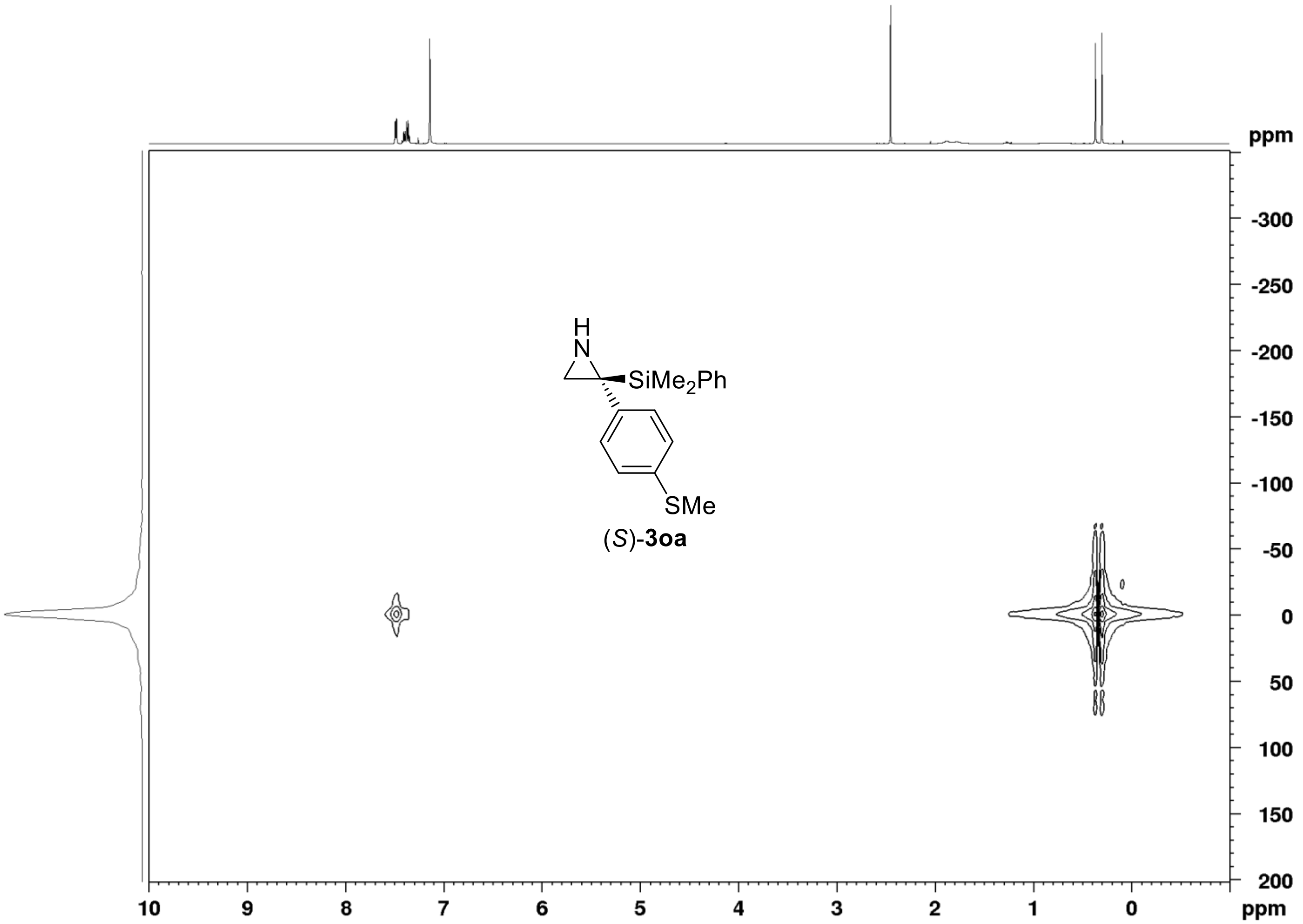

Figure S72. **^{1}H NMR** (500 MHz, $CDCl_3$, 298 K) of **(*S*)-4-(2-(dimethyl(phenyl)silyl)aziridin-2-yl)phenyl acetate** [(*S*)-**3pa**].

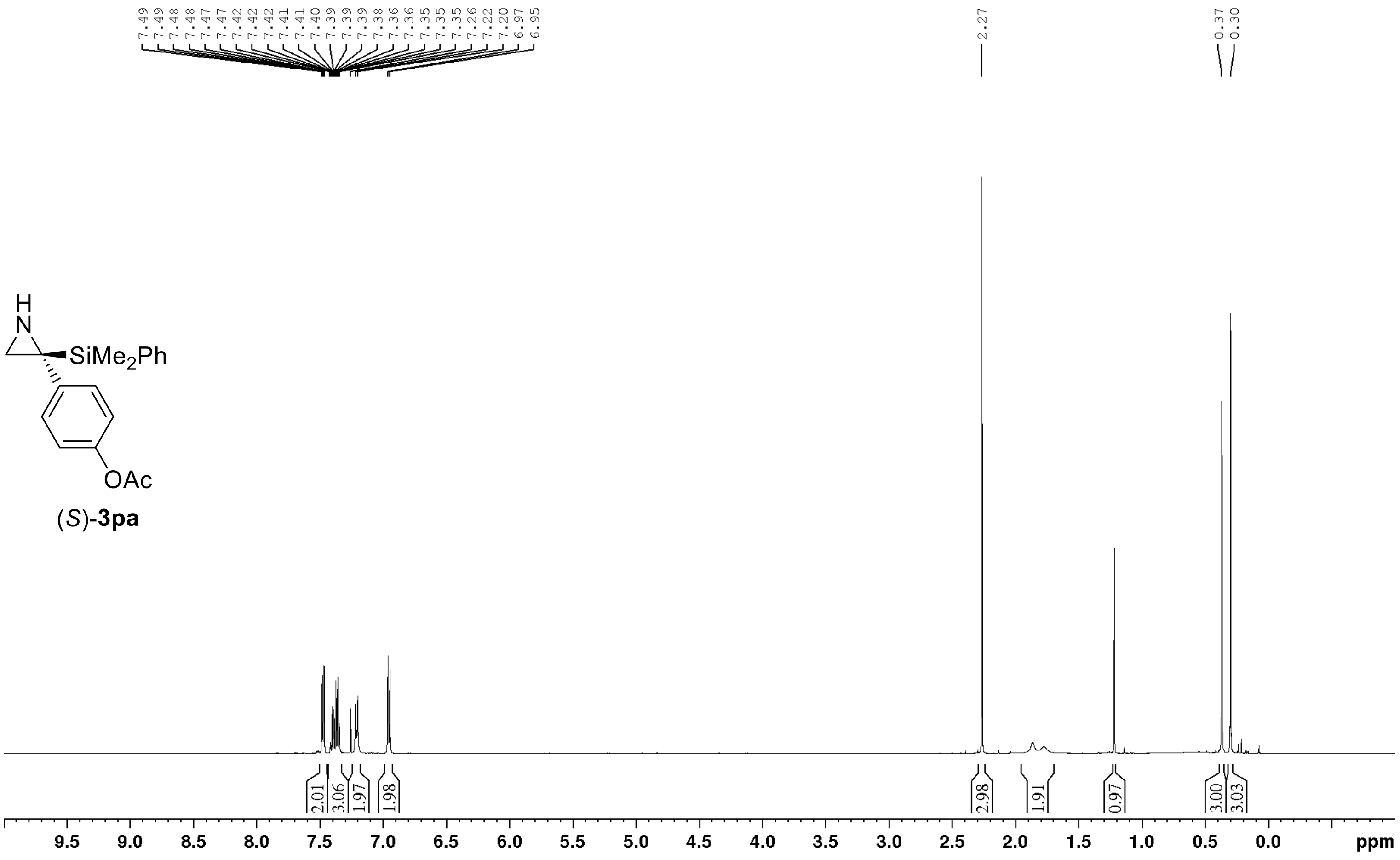

Figure S73. **^{13}C NMR** (126 MHz, $CDCl_3$, 298 K) of **(*S*)-4-(2-(dimethyl(phenyl)silyl)aziridin-2-yl)phenyl acetate** [(*S*)-**3pa**].

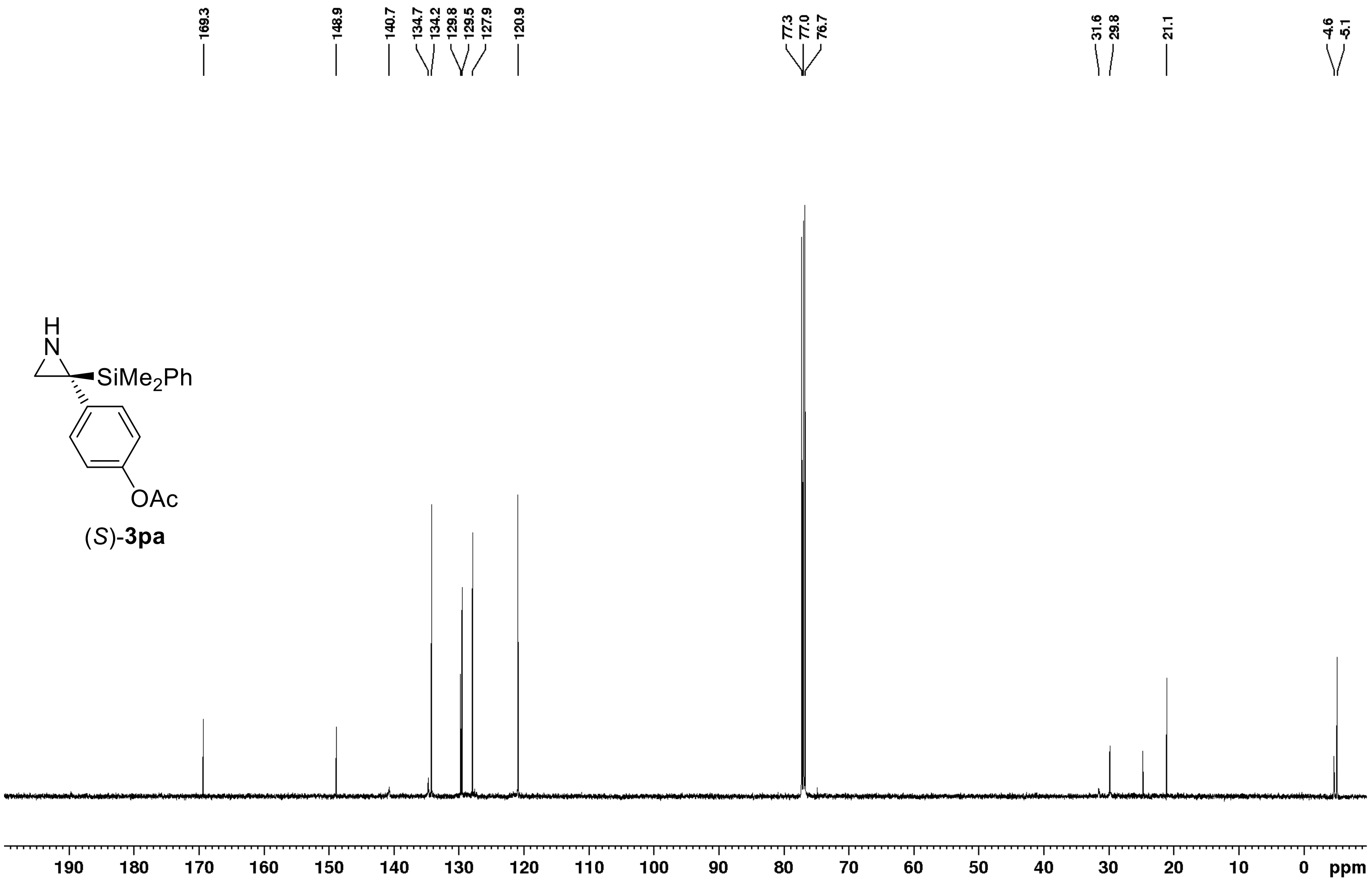

Figure S74. **$^{1}H/^{29}Si$ HMQC NMR** spectrum (500/99 MHz, $CDCl_3$, 298 K, optimized for J = 7 Hz) of **(*S*)-4-(2-(dimethyl(phenyl)silyl)aziridin-2-yl)phenyl acetate** [(*S*)-**3pa**].

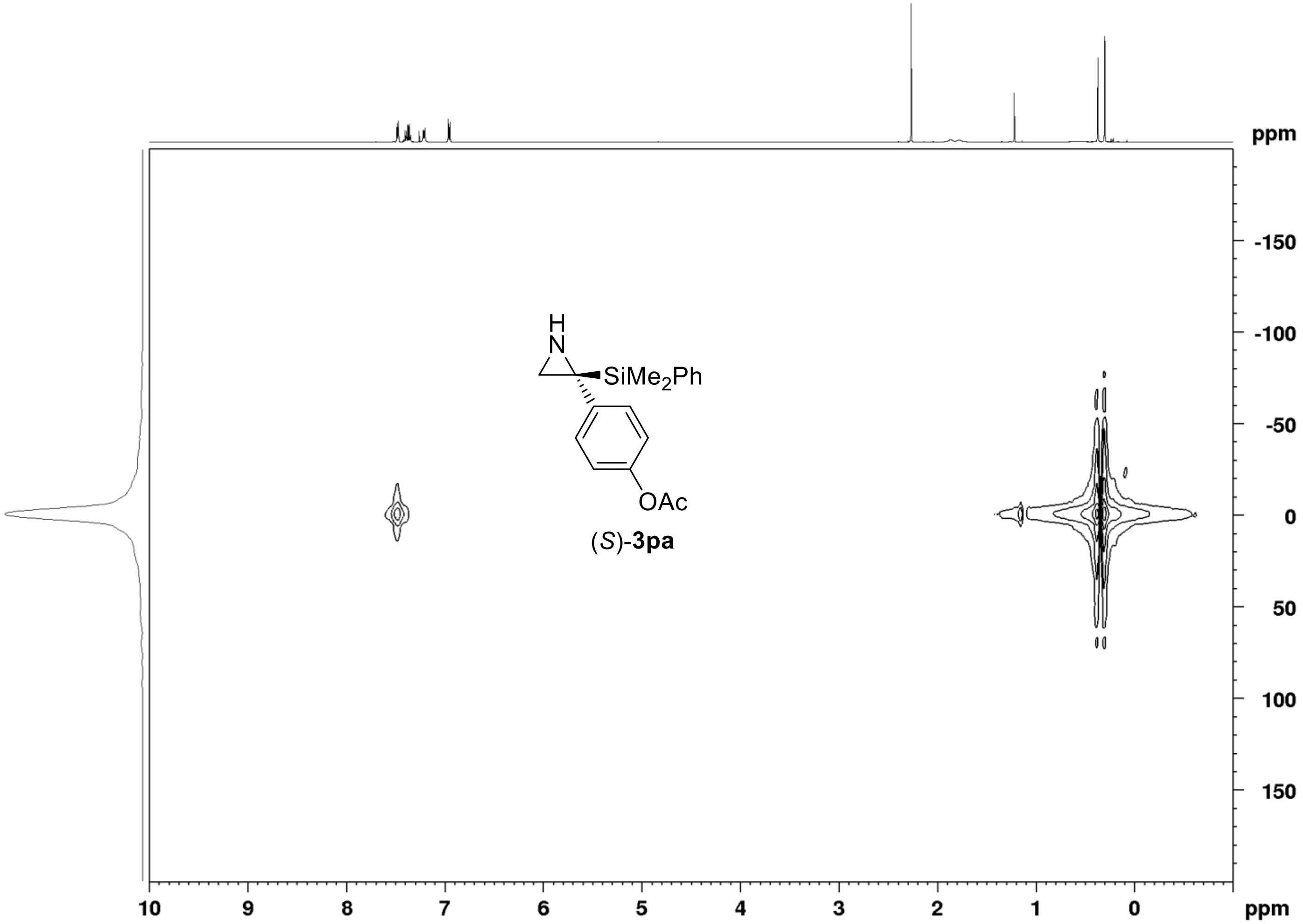

Figure S75. **^{1}H NMR** (500 MHz, $CDCl_3$, 298 K) of **(*S*)-2-(dimethyl(phenyl)silyl)-2-(4-(trifluoromethoxy)phenyl)aziridine** [(*S*)-**3qa**].

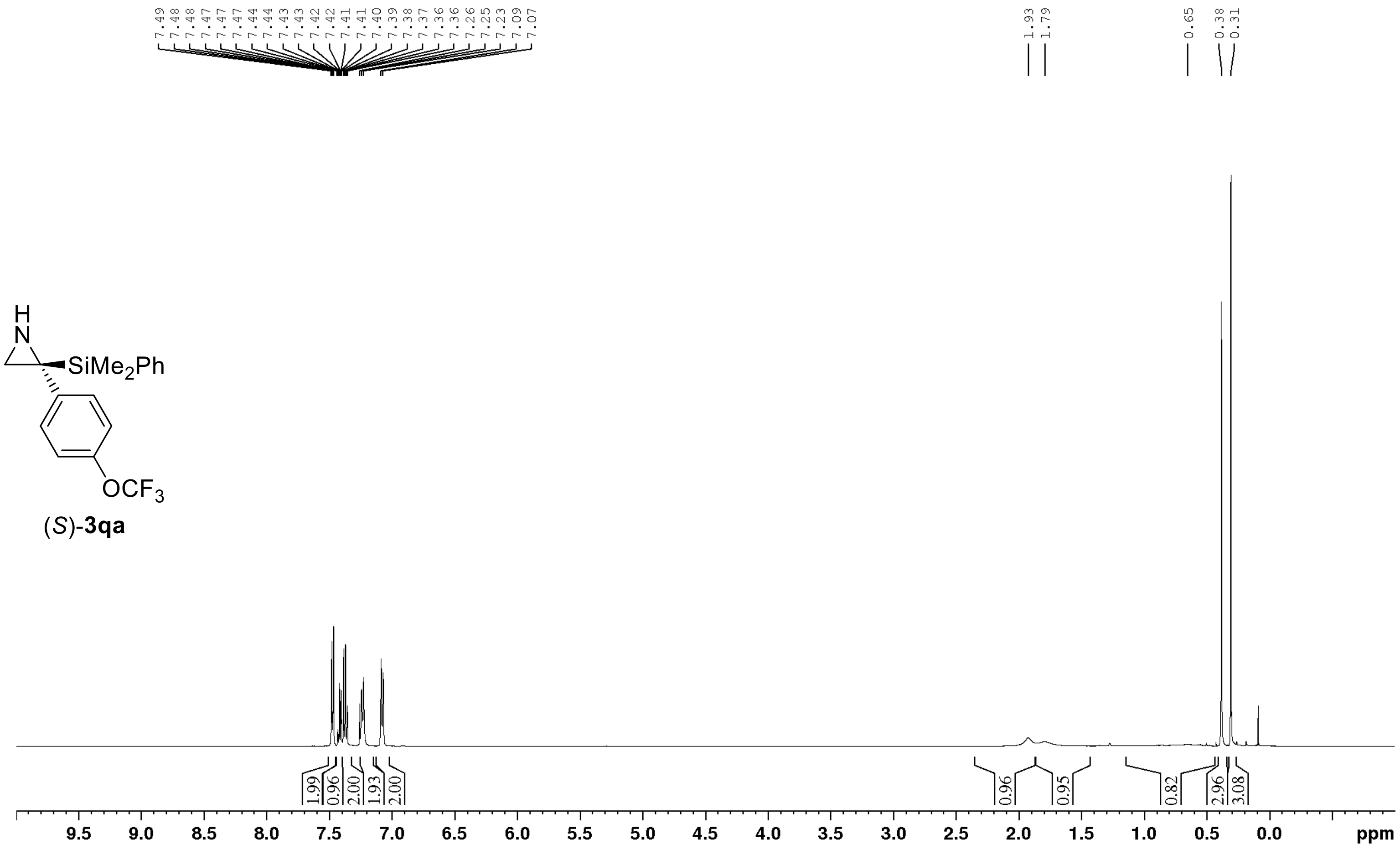

Figure S76. **^{13}C NMR** (126 MHz, $CDCl_3$, 298 K) of **(*S*)-2-(dimethyl(phenyl)silyl)-2-(4-(trifluoromethoxy)phenyl)aziridine** [(*S*)-**3qa**].

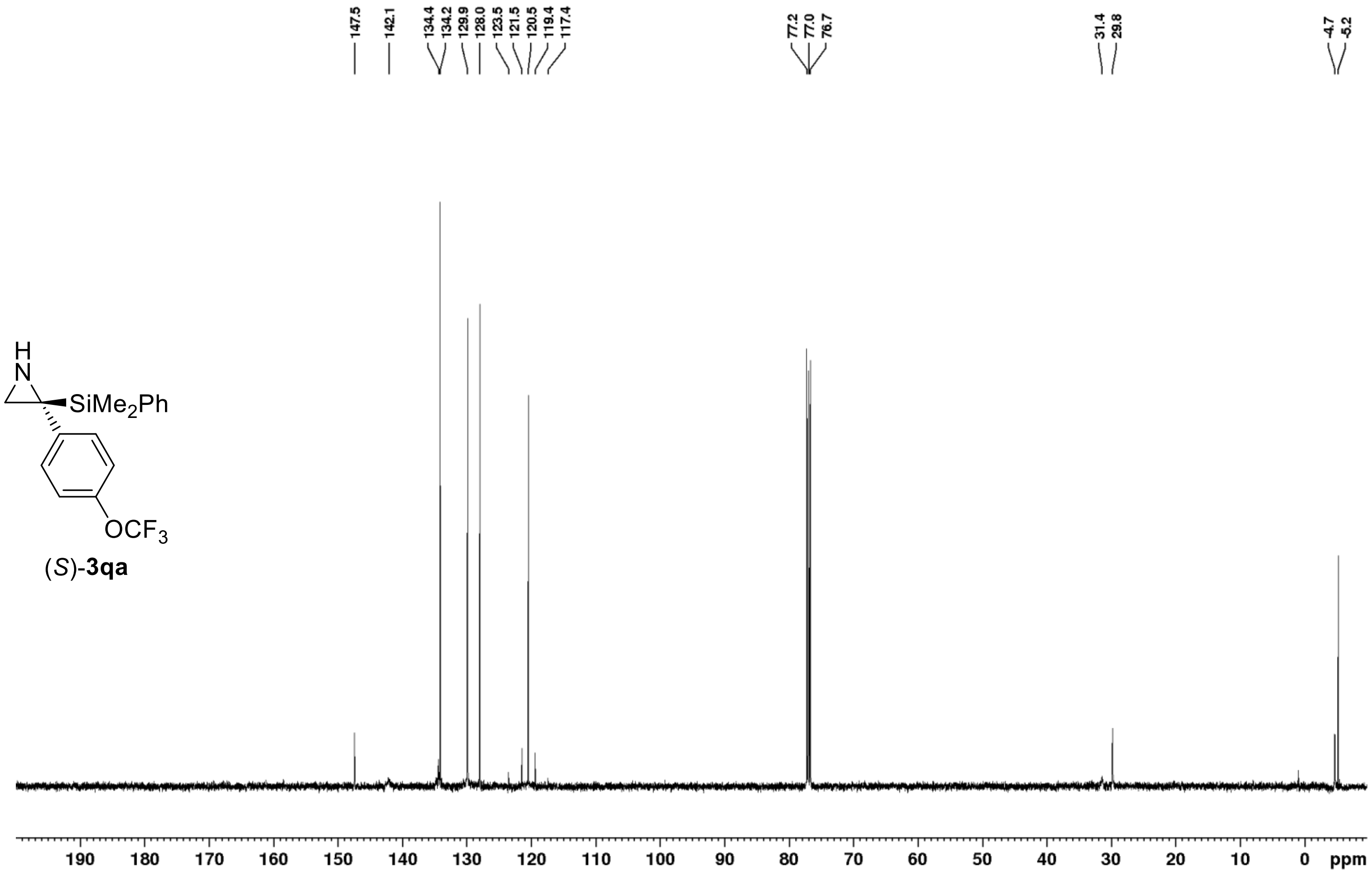

Figure S77. **^{19}F NMR** spectrum (471 MHz, $CDCl_3$, 298 K) of **(*S*)-2-(dimethyl(phenyl)silyl)-2-(4-(trifluoromethoxy)phenyl)aziridine** [(*S*)-**3qa**].

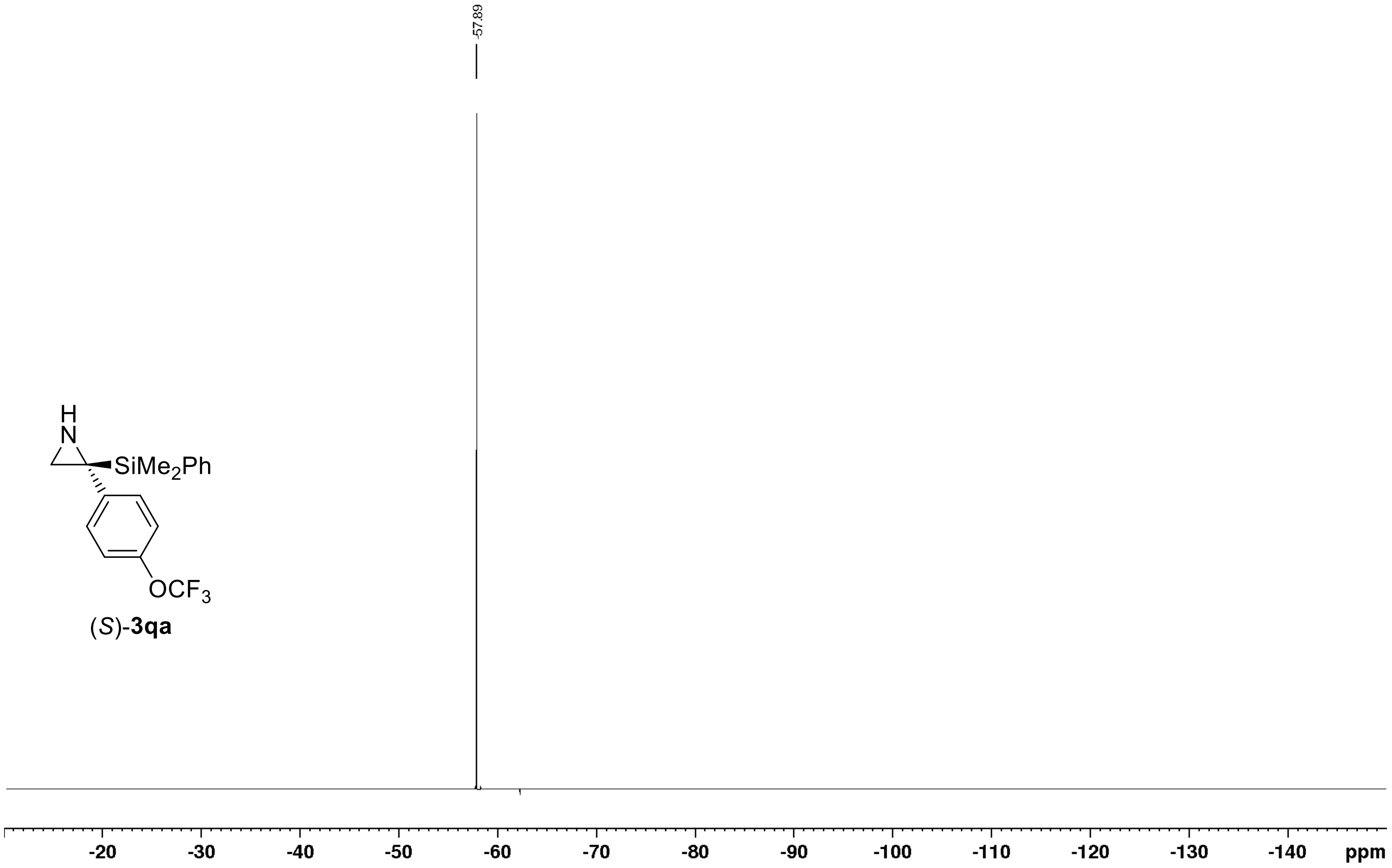

(*S*)-**3qa**

Figure S78. ^{1}**H/**^{29}**Si HMQC NMR** spectrum (500/99 MHz, $CDCl_3$, 298 K, optimized for J = 7 Hz) of **(*S*)-2-(dimethyl(phenyl)silyl)-2-(4-(trifluoromethoxy)phenyl)aziridine** [(*S*)-**3qa**].

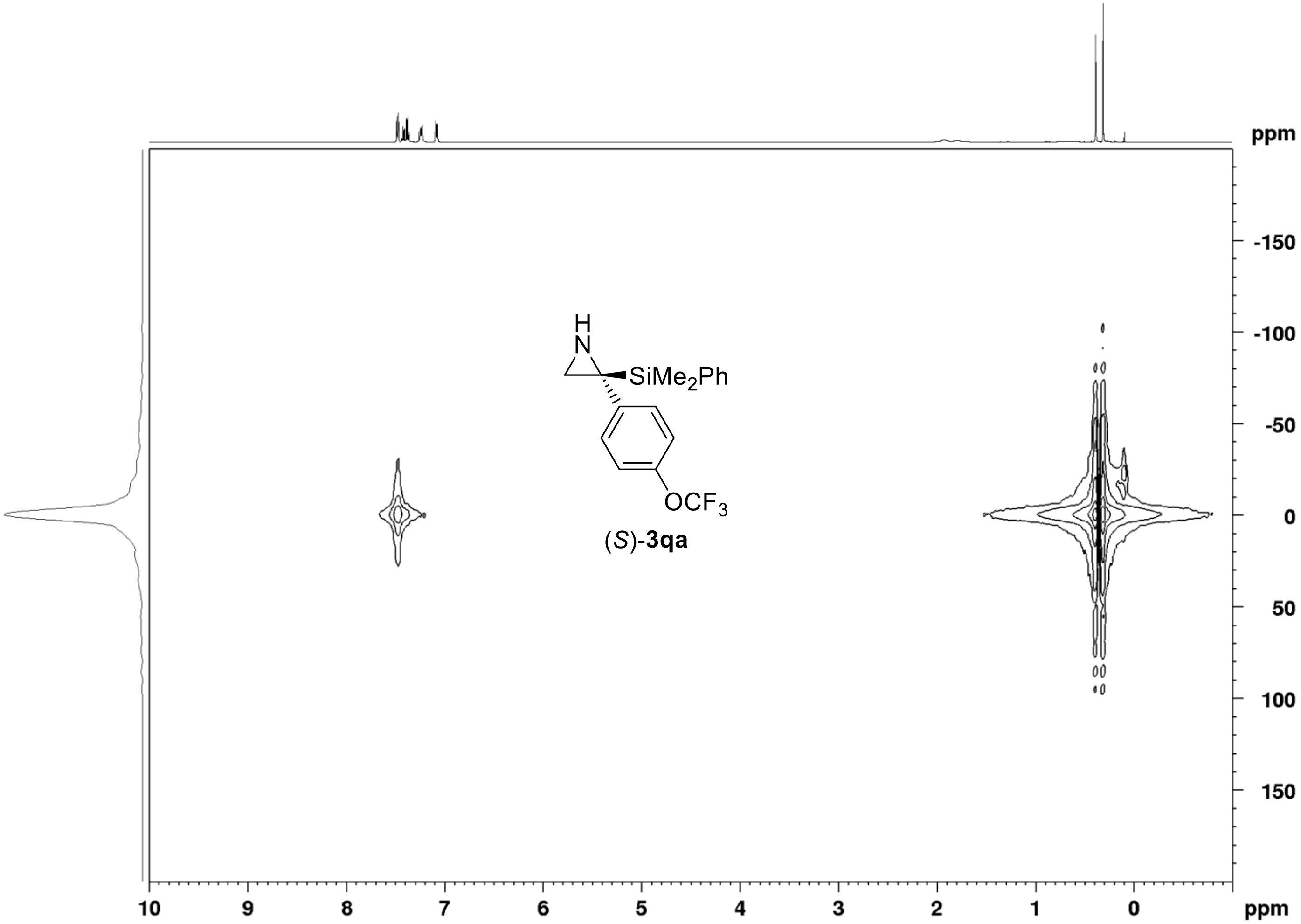

Figure S79. **^{1}H NMR** (500 MHz, $CDCl_3$, 298 K) of **(*S*)-2-(dimethyl(phenyl)silyl)-2-(4-(trifluoromethyl)phenyl)aziridine** [(*S*)-**3ra**].

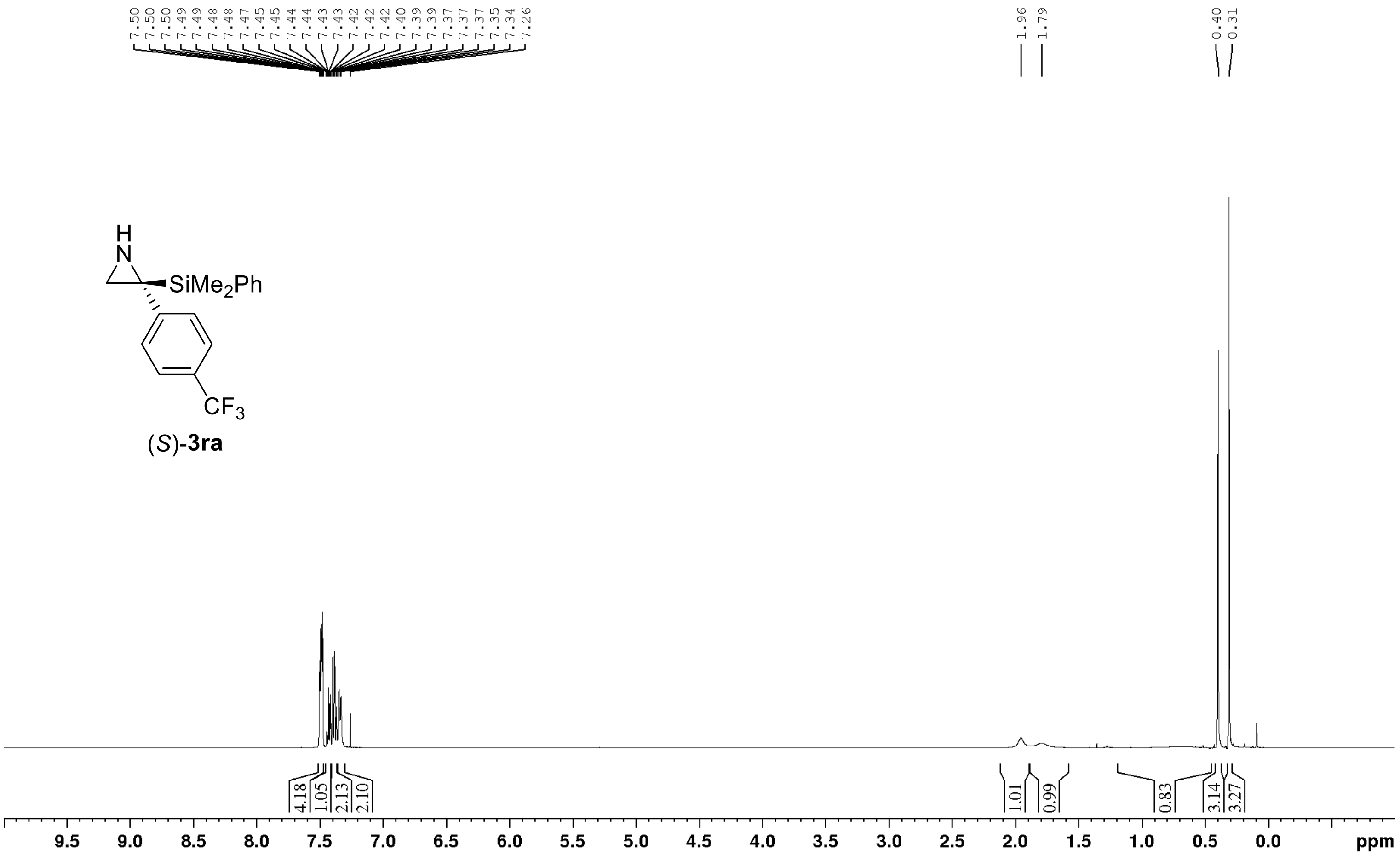

Figure S80. **^{13}C NMR** (126 MHz, $CDCl_3$, 298 K) of **(*S*)-2-(dimethyl(phenyl)silyl)-2-(4-(trifluoromethyl)phenyl)aziridine** [(*S*)-**3ra**].

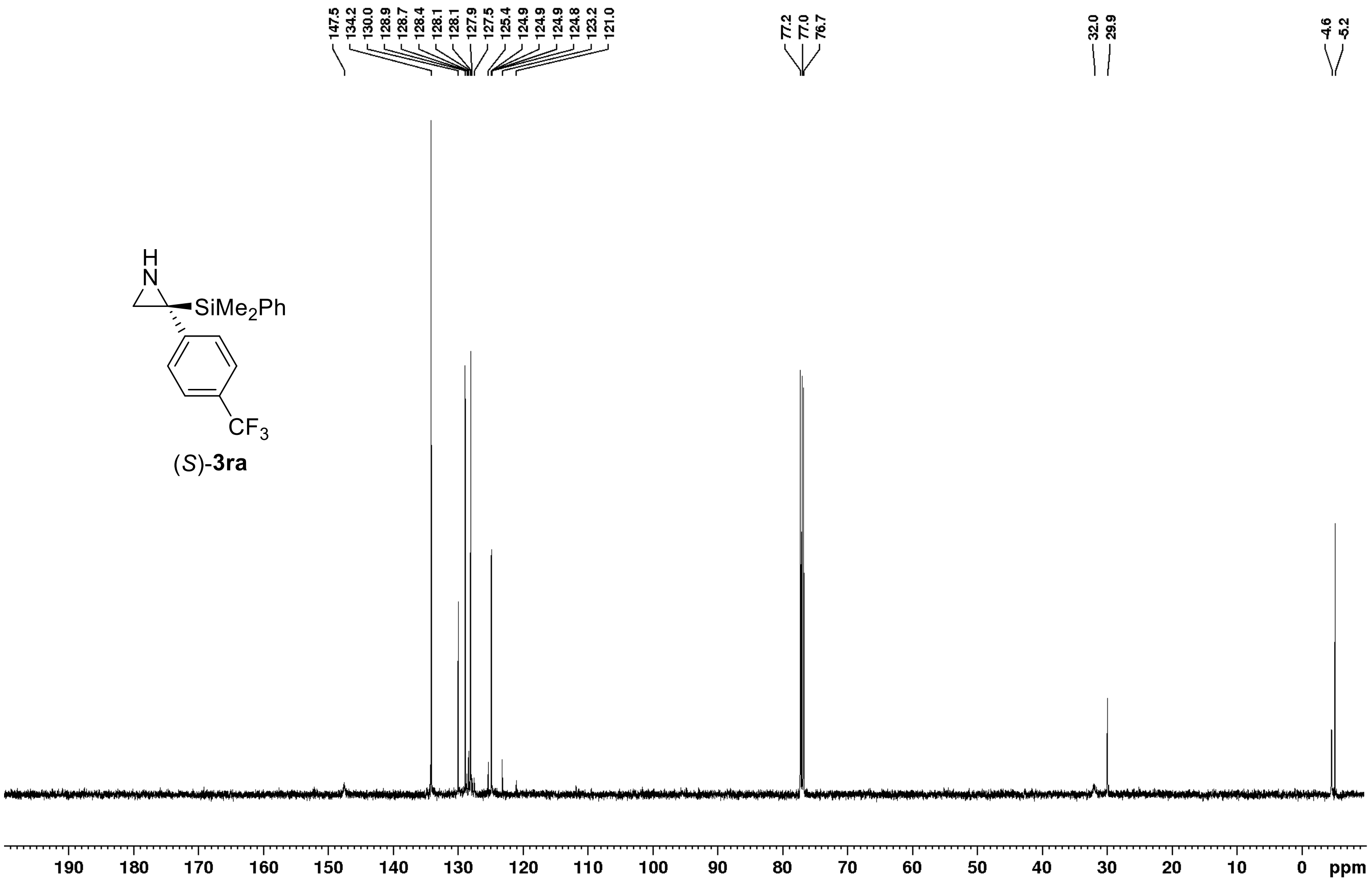

Figure S81. **^{19}F NMR** spectrum (471 MHz, $CDCl_3$, 298 K) of **(*S*)-2-(dimethyl(phenyl)silyl)-2-(4-(trifluoromethyl)phenyl)aziridine** [(*S*)-**3ra**].

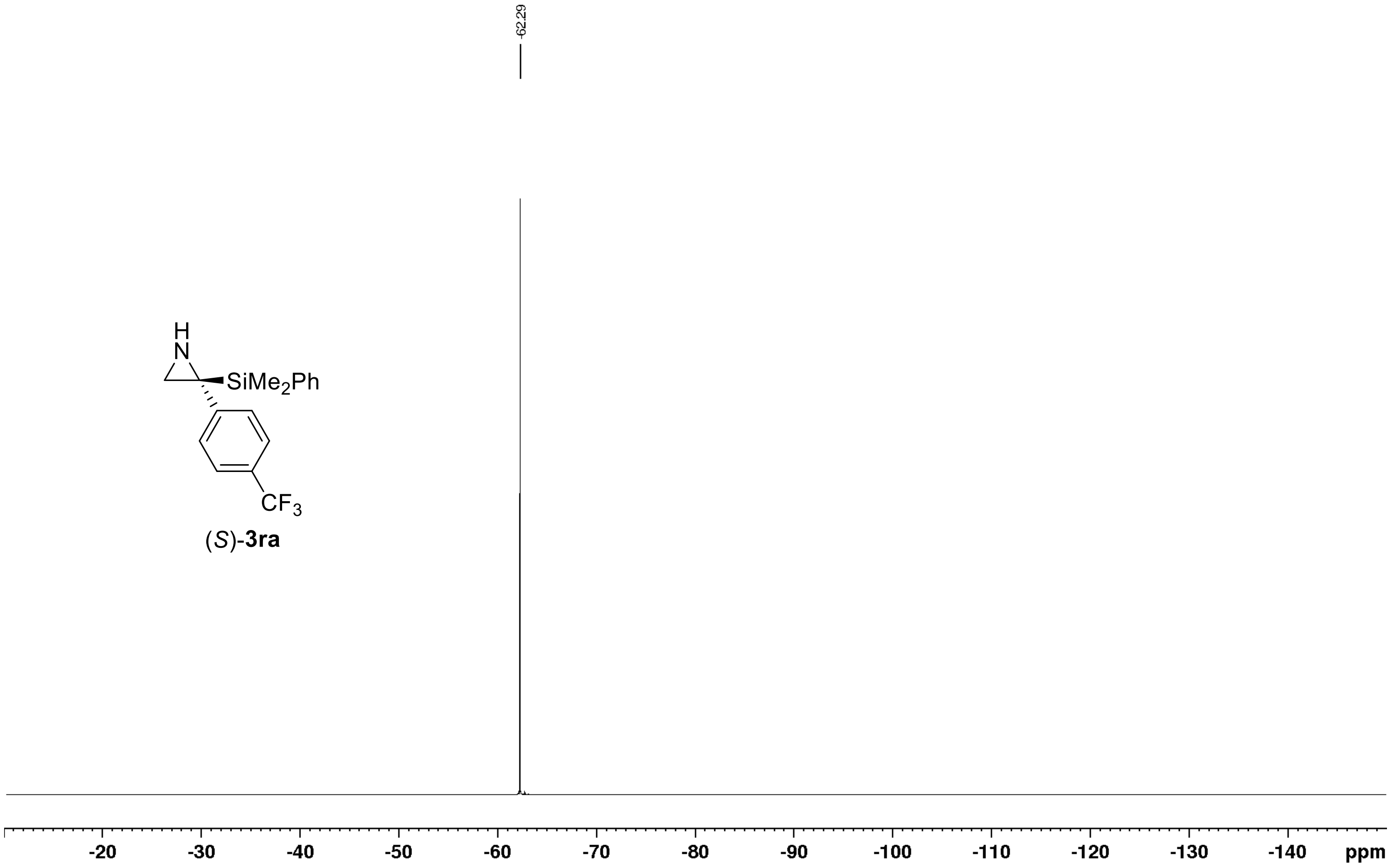

Figure S82. **^{1}H/^{29}Si HMQC NMR** spectrum (500/99 MHz, $CDCl_3$, 298 K, optimized for *J* = 7 Hz) of **(*S*)-2-(dimethyl(phenyl)silyl)-2-(4-(trifluoromethyl)phenyl)aziridine** [(*S*)-**3ra**].

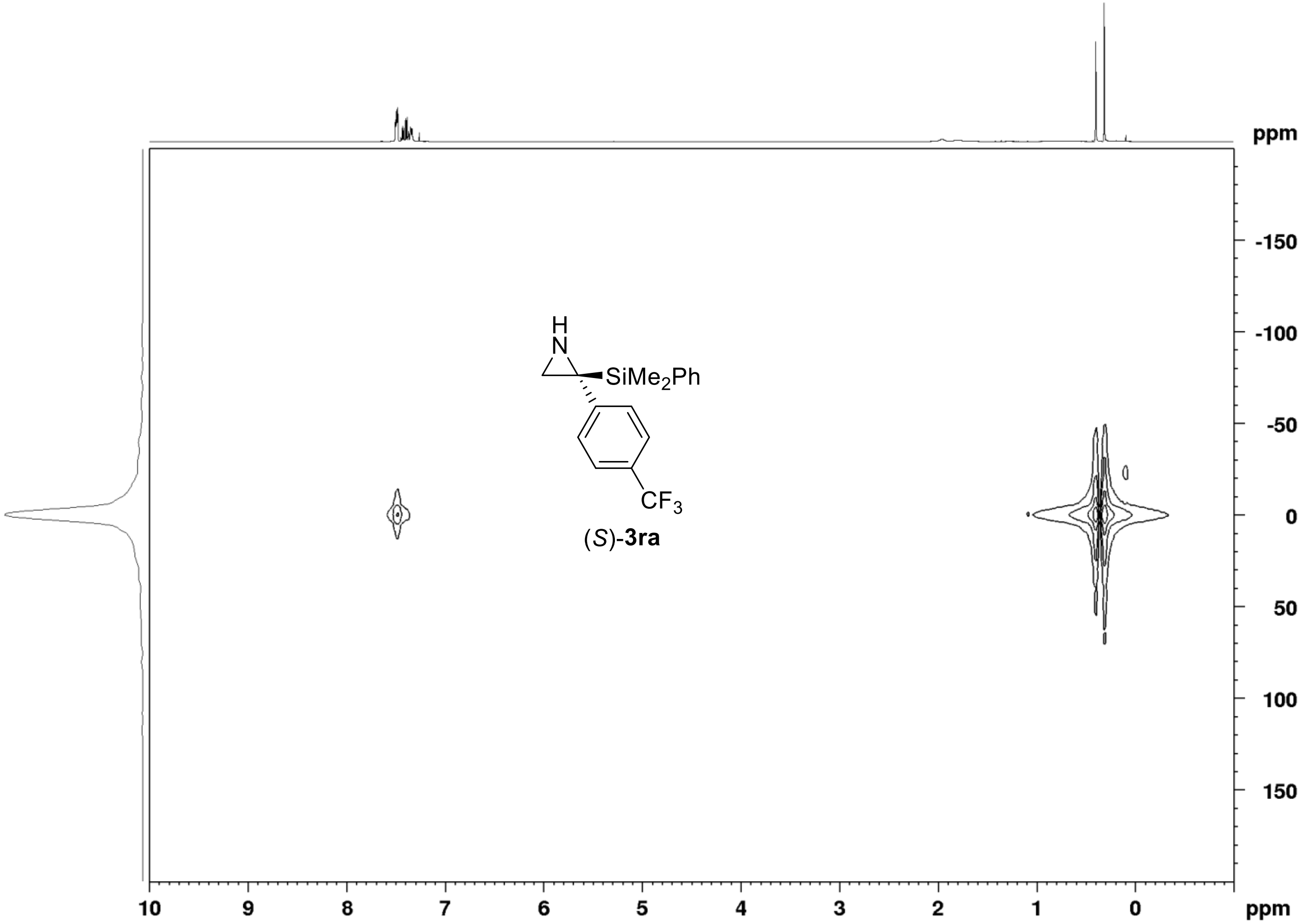

Figure S83. **^{1}H NMR** (500 MHz, $CDCl_3$, 298 K) of **(*S*)-2-(dimethyl(phenyl)silyl)-2-(4-isocyanophenyl)aziridine** [(*S*)-**3sa**].

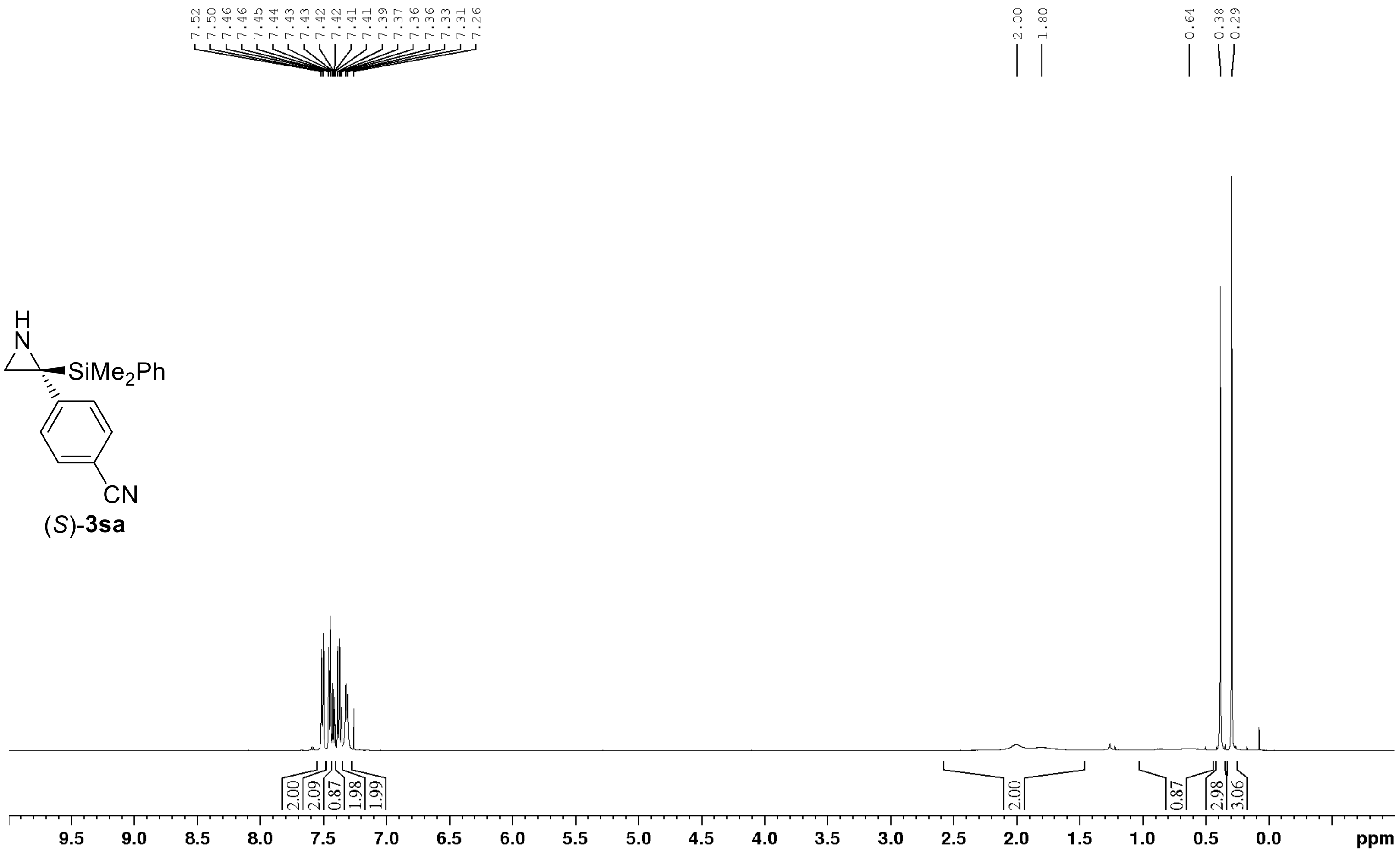

Figure S84. **^{13}C NMR** (126 MHz, $CDCl_3$, 298 K) of **(*S*)-2-(dimethyl(phenyl)silyl)-2-(4-isocyanophenyl)aziridine** [(*S*)-**3sa**].

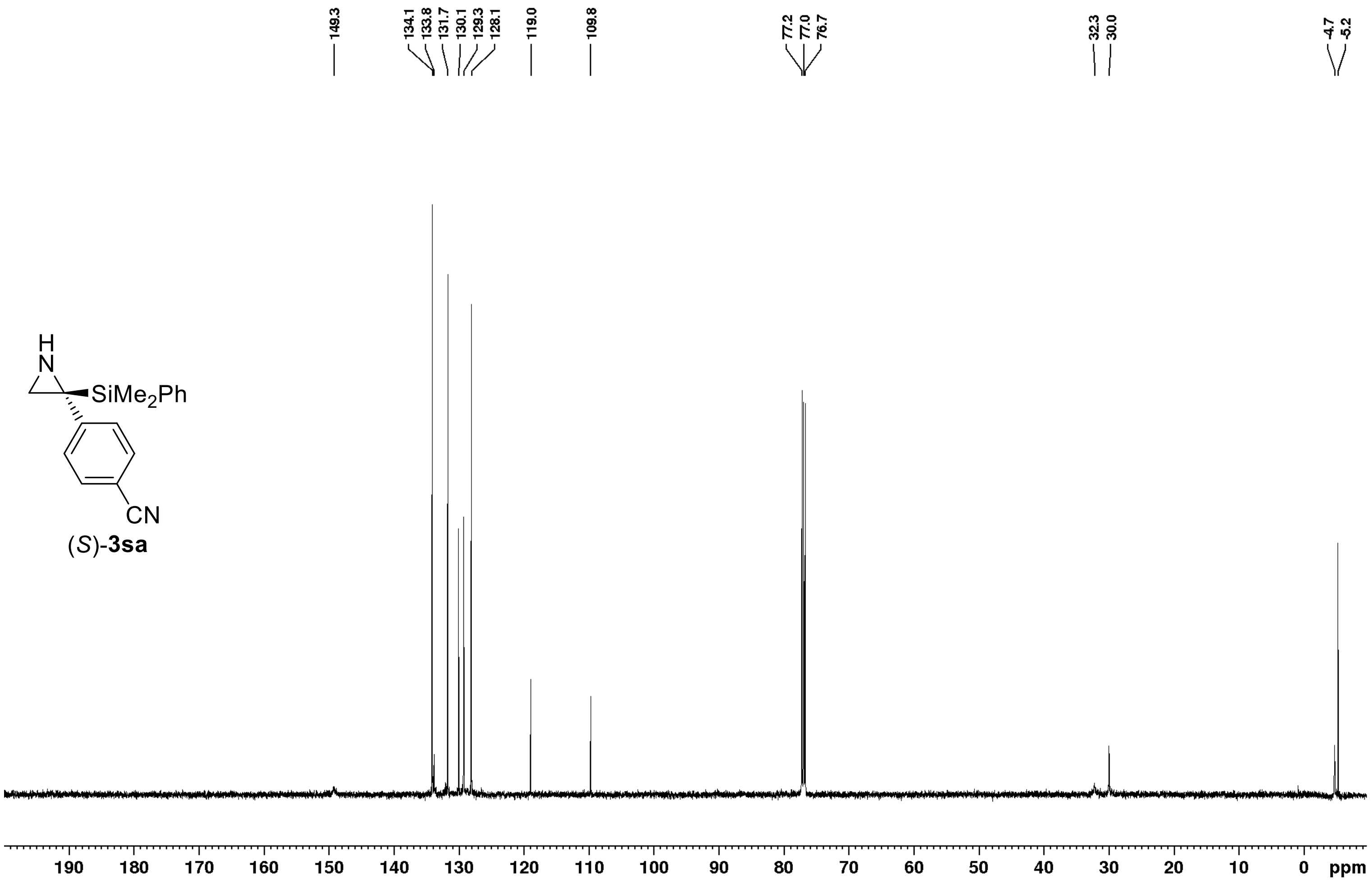

Figure S85. **^{1}H/^{29}Si HMQC NMR** spectrum (500/99 MHz, $CDCl_3$, 298 K, optimized for *J* = 7 Hz) of **(*S*)-2-(dimethyl(phenyl)silyl)-2-(4-isocyanophenyl)aziridine** [(*S*)-**3sa**].

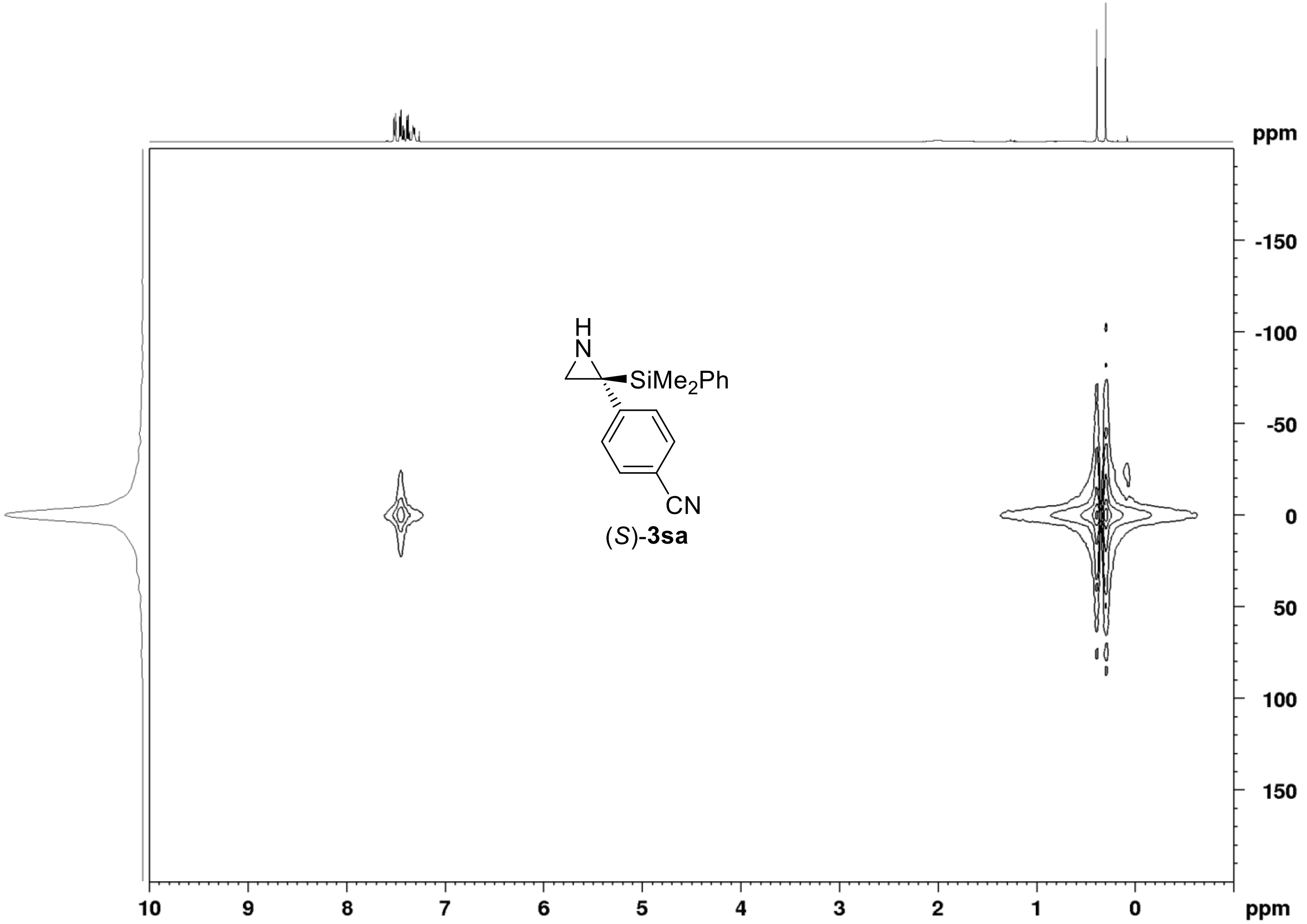

Figure S86. **^{1}H NMR** (500 MHz, $CDCl_3$, 298 K) of **(*S*)-1-(4-(2-(dimethyl(phenyl)silyl)aziridin-2-yl)phenyl)ethan-1-one** [(*S*)-**3ta**].

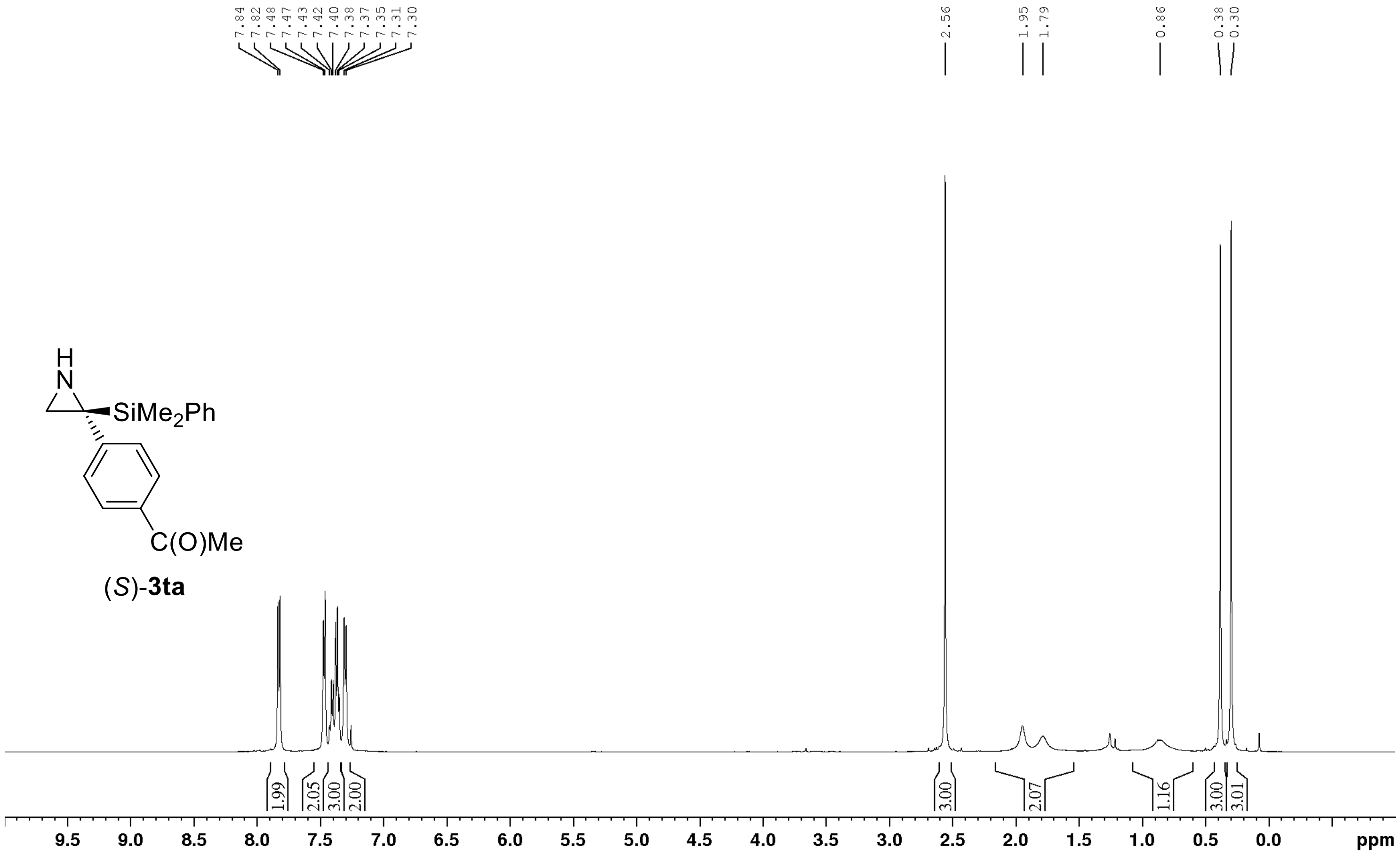

Figure S87. ^{13}**C NMR** (126 MHz, $CDCl_3$, 298 K) of **(*S*)-1-(4-(2-(dimethyl(phenyl)silyl)aziridin-2-yl)phenyl)ethan-1-one** [(*S*)-**3ta**].

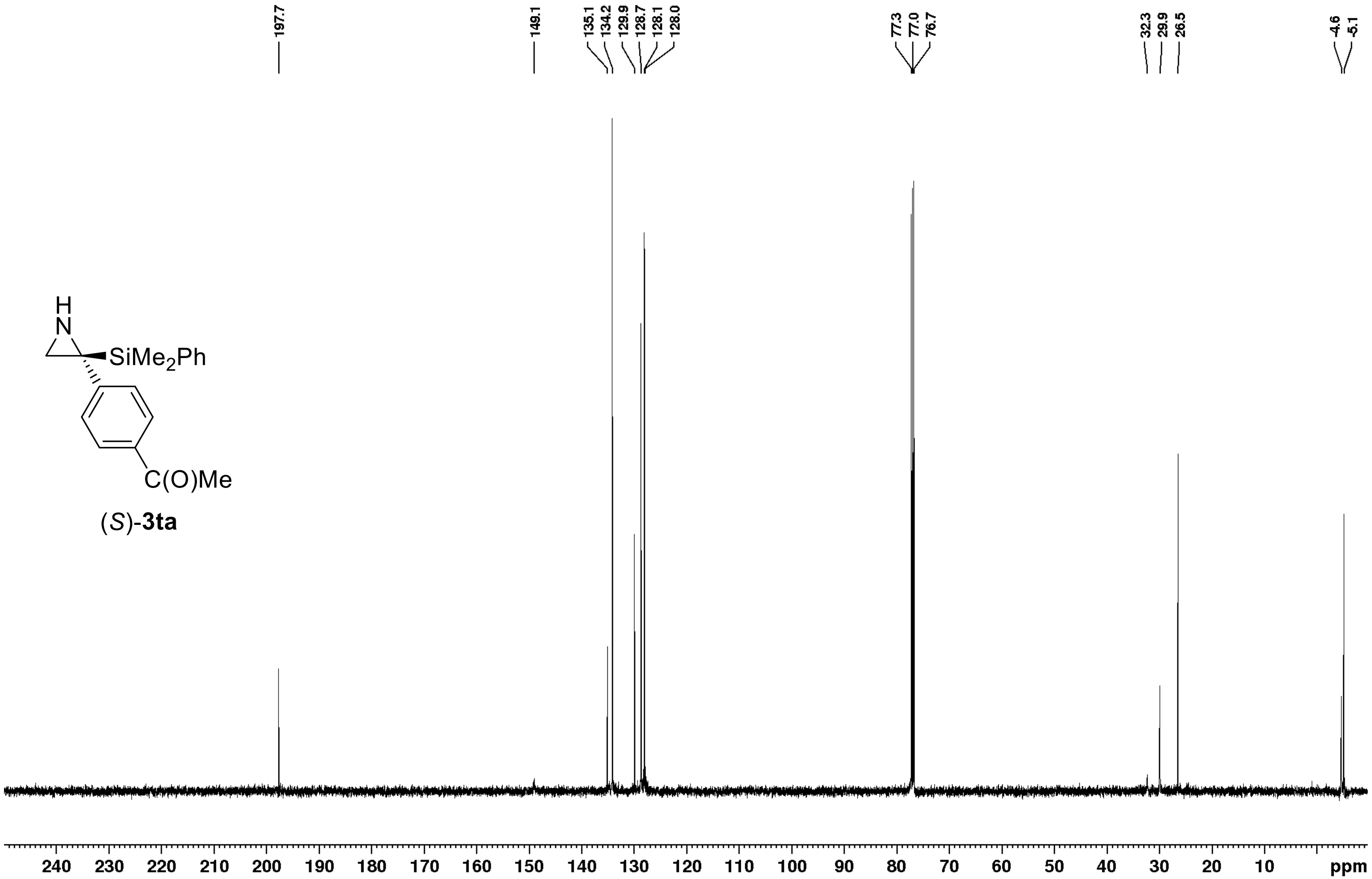

Figure S88. **$^{1}H/^{29}Si$ HMQC NMR** spectrum (500/99 MHz, $CDCl_3$, 298 K, optimized for J = 7 Hz) of **(*S*)-1-(4-(2-(dimethyl(phenyl)silyl)aziridin-2-yl)phenyl)ethan-1-one** [(*S*)-**3ta**].

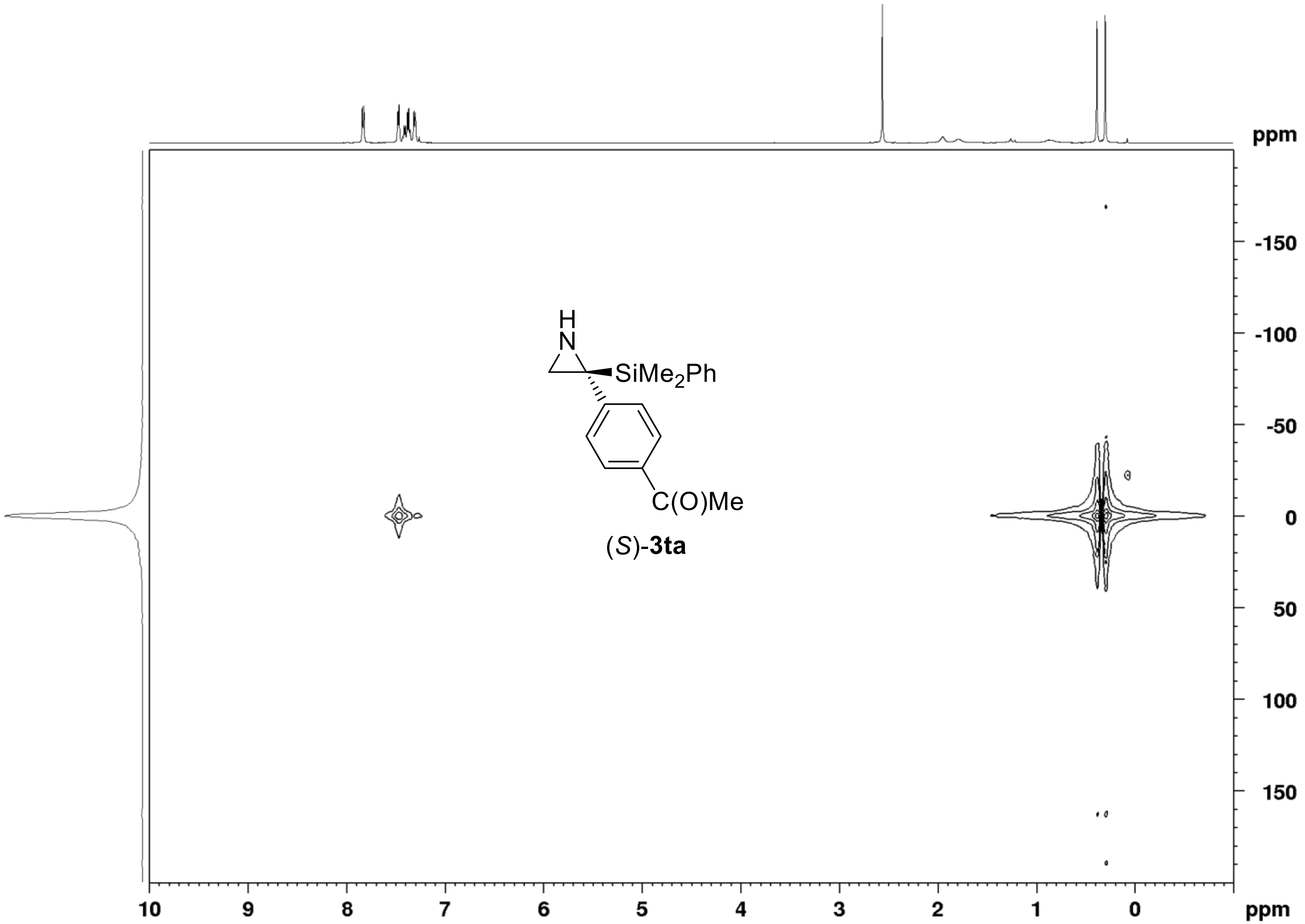

Figure S89. **^{1}H NMR** (500 MHz, $CDCl_3$, 298 K) of **(*S*)-2-(dimethyl(phenyl)silyl)-2-(3-fluorophenyl)aziridine** [(*S*)-**3ua**].

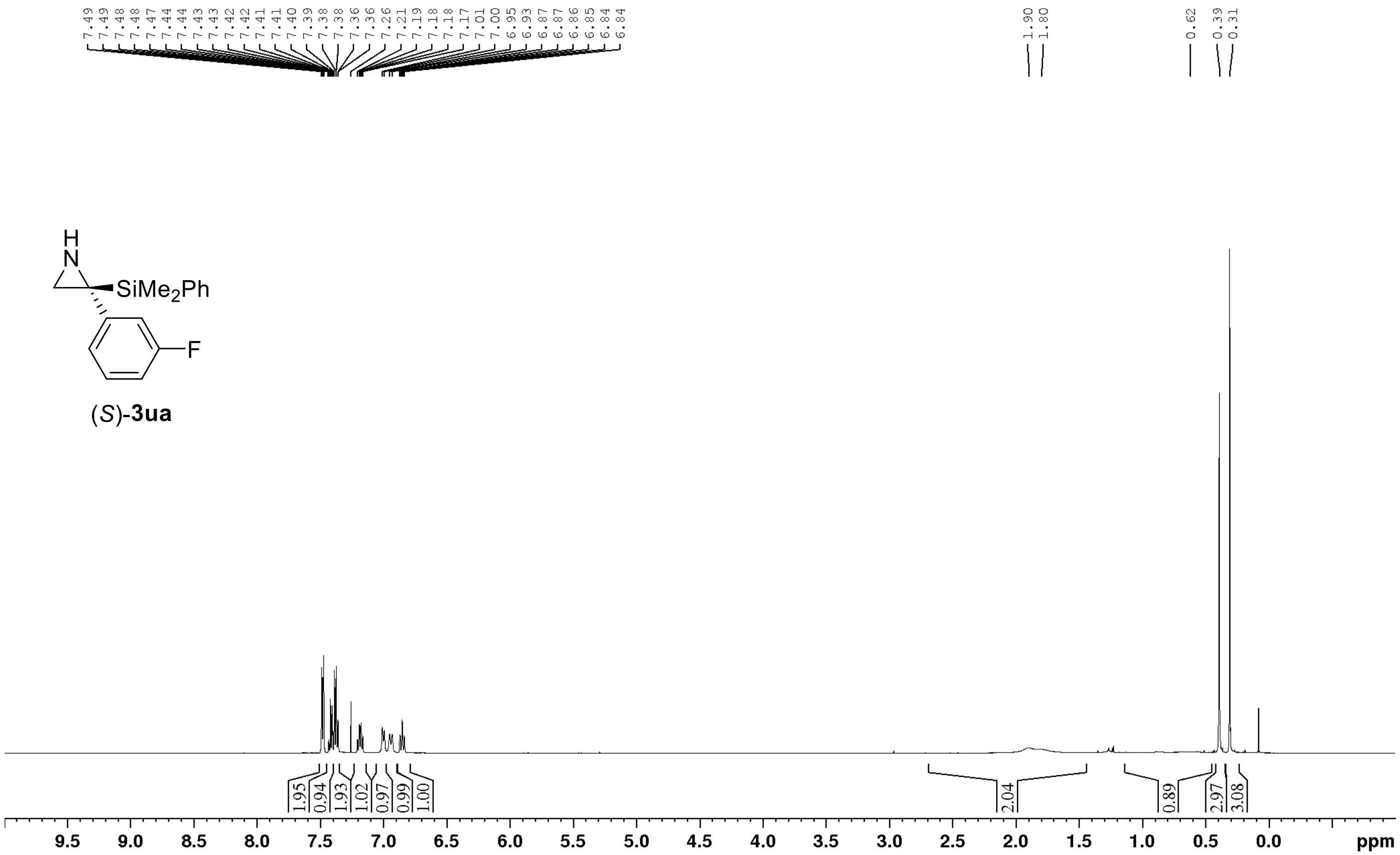

Figure S90. **^{13}C NMR** (126 MHz, $CDCl_3$, 298 K) of **(*S*)-2-(dimethyl(phenyl)silyl)-2-(3-fluorophenyl)aziridine** [(*S*)-**3ua**].

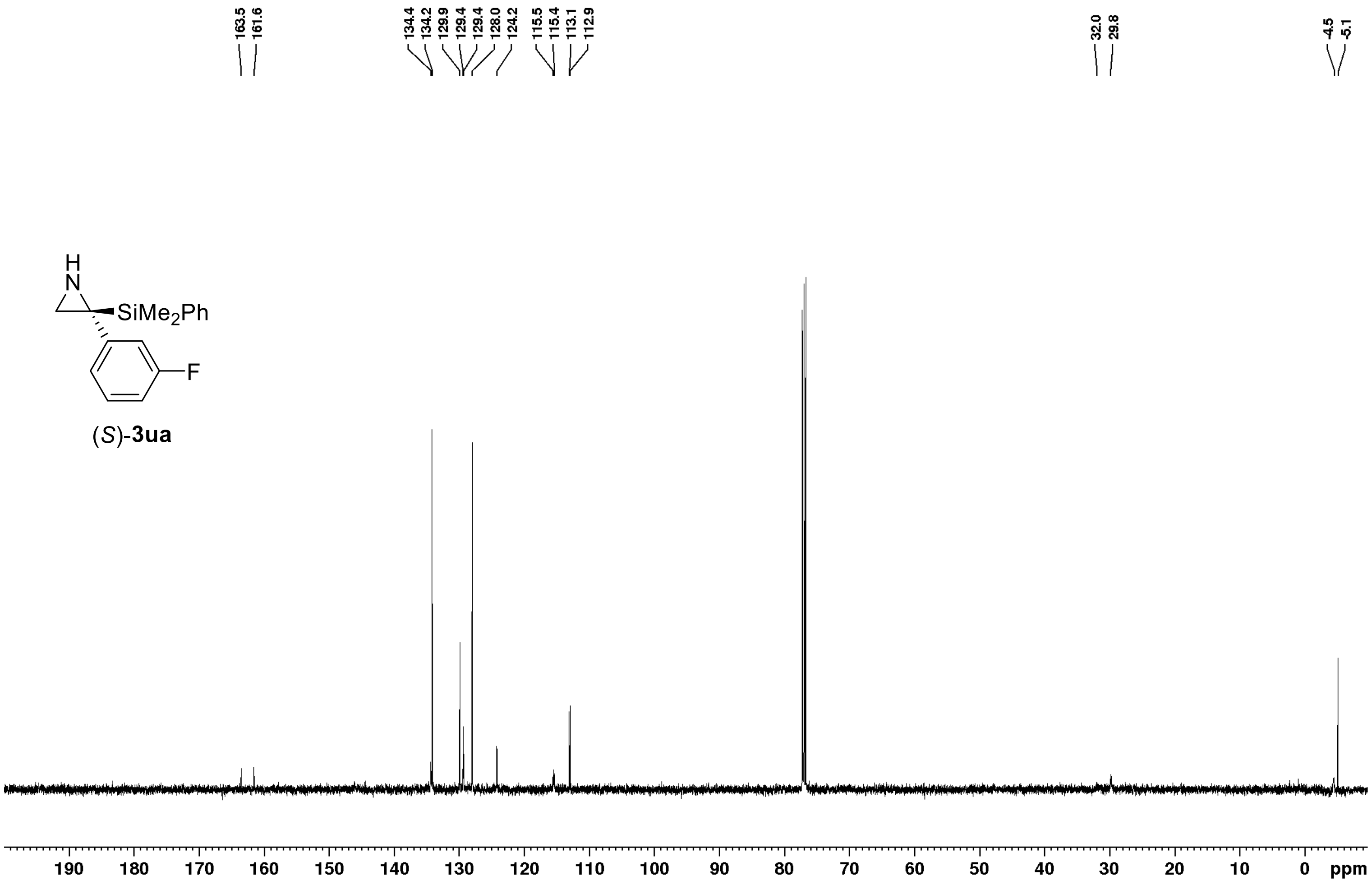

Figure S91. **^{19}F NMR** spectrum (471 MHz, $CDCl_3$, 298 K) of **(*S*)-2-(dimethyl(phenyl)silyl)-2-(3-fluorophenyl)aziridine** [(*S*)-**3ua**].

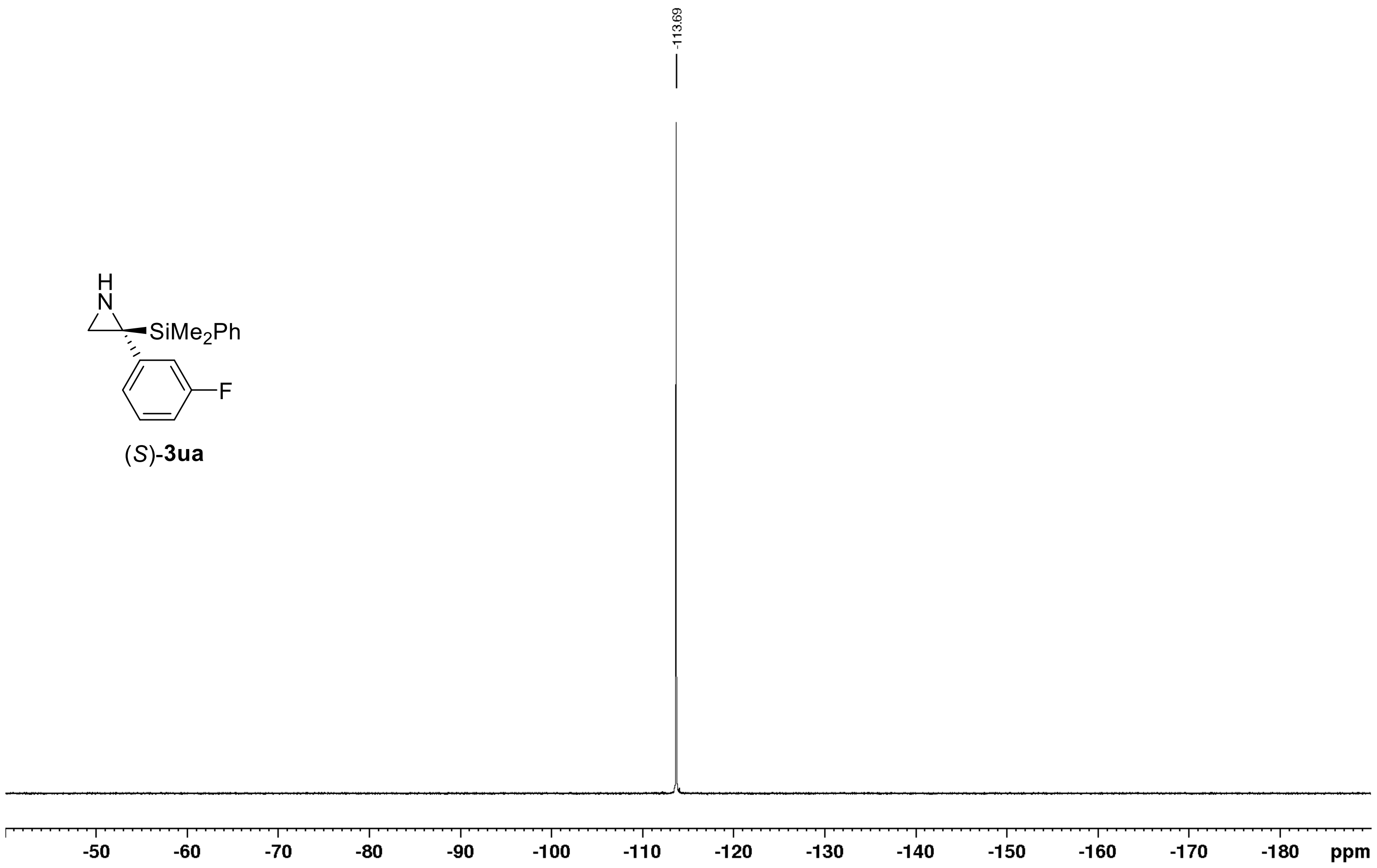

Figure S92. **^{1}H/^{29}Si HMQC NMR** spectrum (500/99 MHz, $CDCl_3$, 298 K, optimized for J = 7 Hz) of **(*S*)-2-(dimethyl(phenyl)silyl)-2-(3-fluorophenyl)aziridine** [(*S*)-**3ua**].

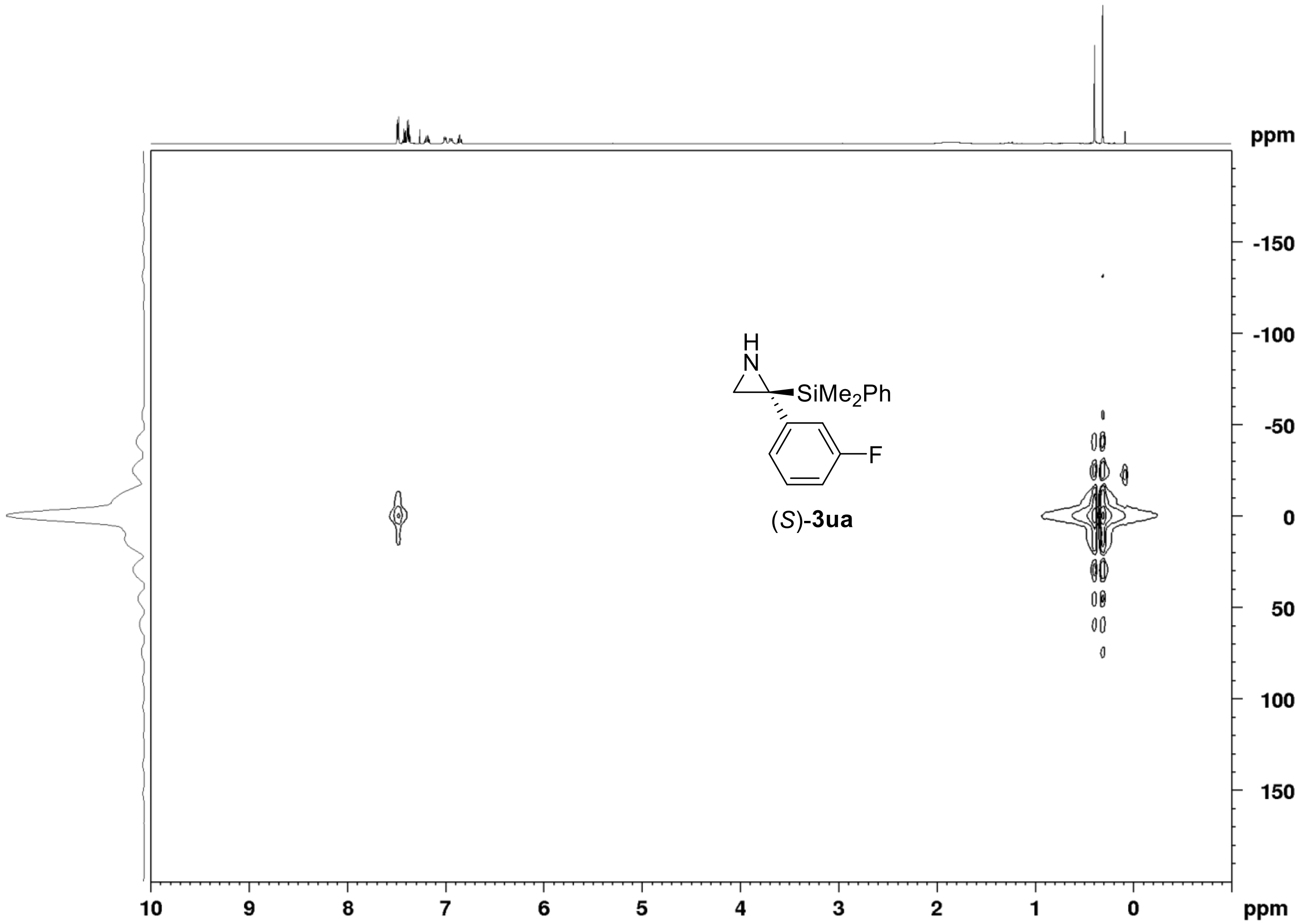

Figure S93. **^{1}H NMR** (500 MHz, $CDCl_3$, 298 K) of **(*S*)-2-(dimethyl(phenyl)silyl)-2-(4-fluorophenyl)aziridine** [(*S*)-**3va**].

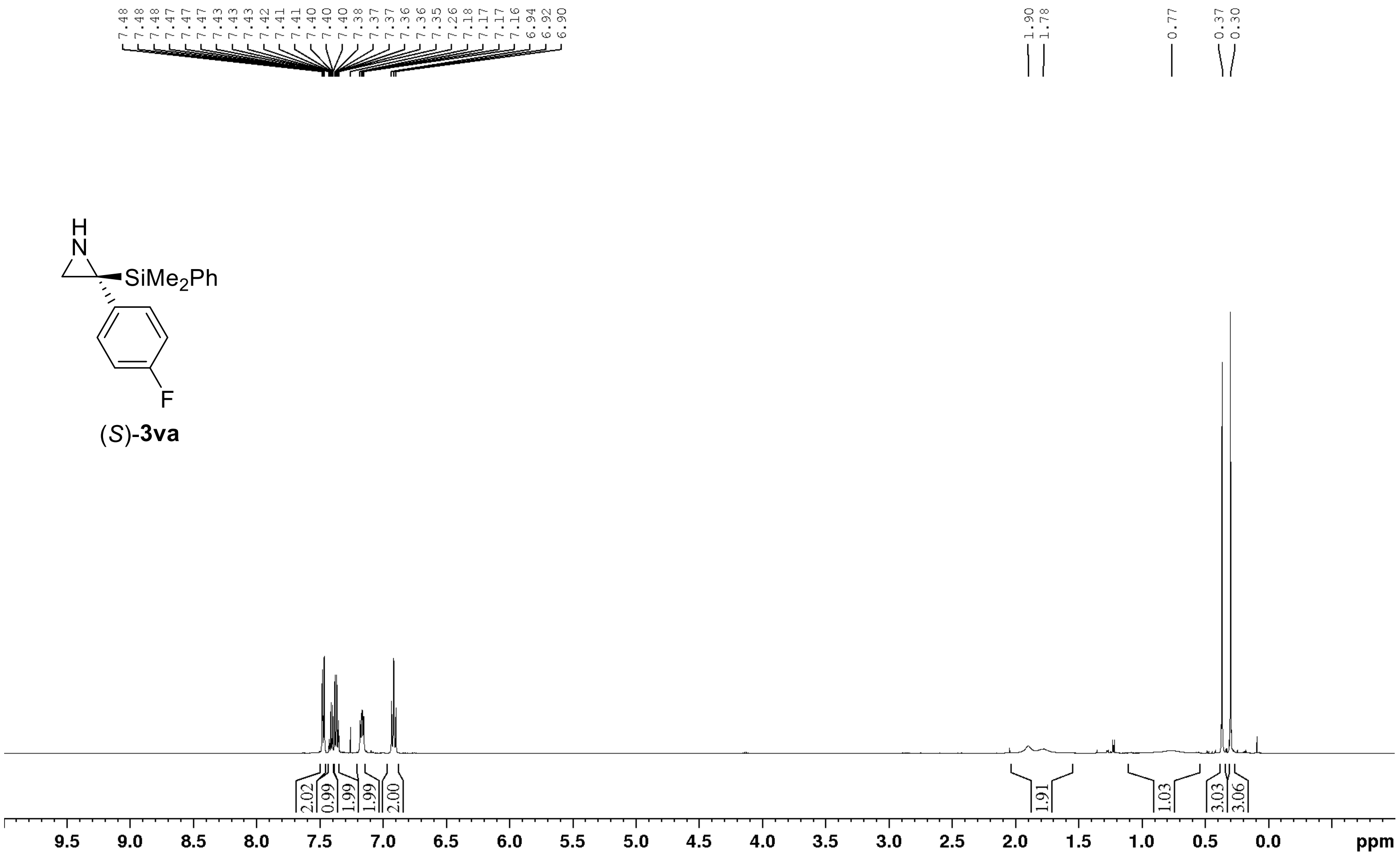

Figure S94. **^{13}C NMR** (126 MHz, $CDCl_3$, 298 K) of **(*S*)-2-(dimethyl(phenyl)silyl)-2-(4-fluorophenyl)aziridine** [(*S*)-**3va**].

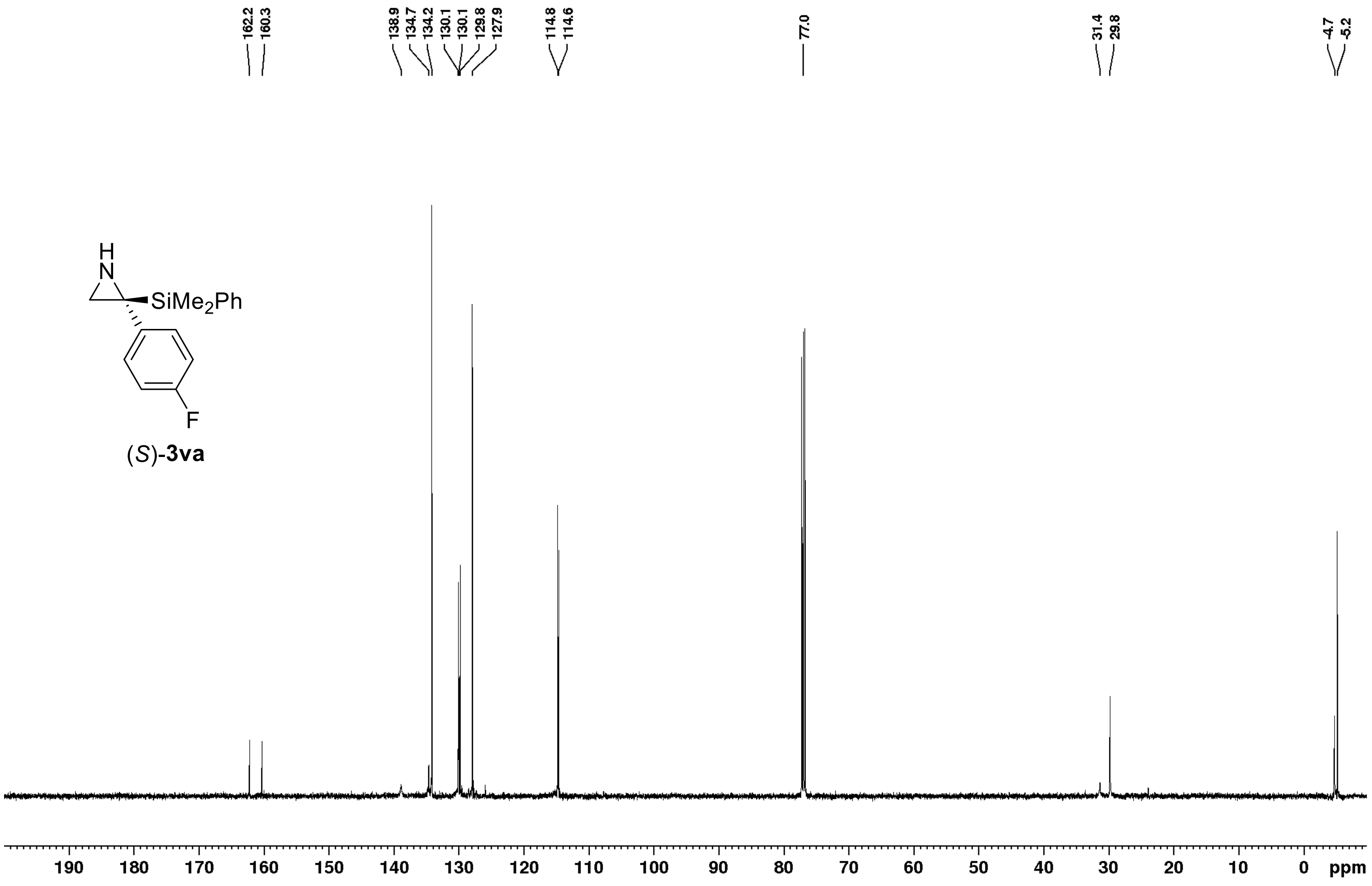

(*S*)-**3va**

Figure S95. **^{19}F NMR** spectrum (471 MHz, $CDCl_3$, 298 K) of **(*S*)-2-(dimethyl(phenyl)silyl)-2-(4-fluorophenyl)aziridine** [(*S*)-**3va**].

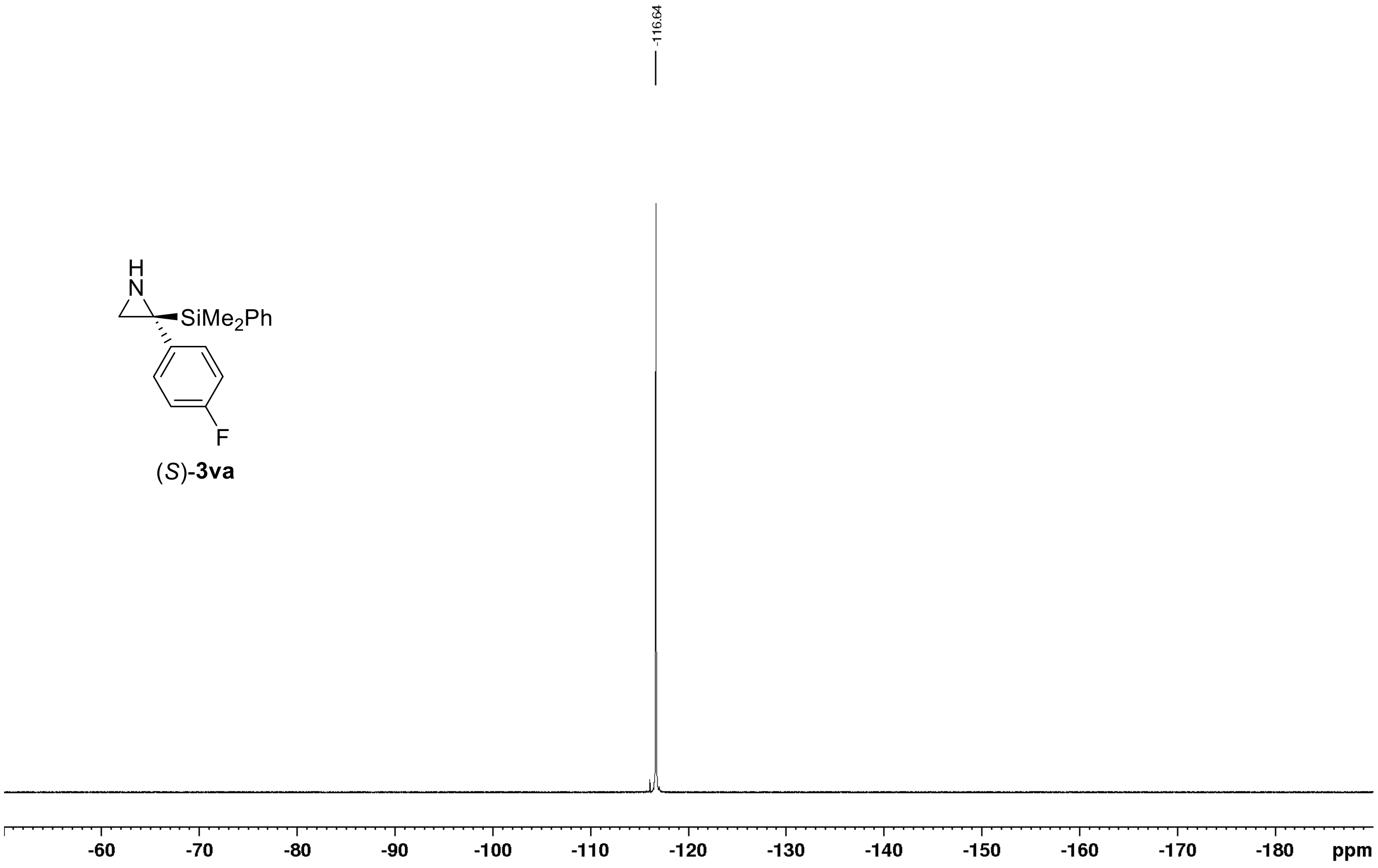

Figure S96. **$^1H/^{29}Si$ HMQC NMR** spectrum (500/99 MHz, $CDCl_3$, 298 K, optimized for *J* = 7 Hz) of **(*S*)-2-(dimethyl(phenyl)silyl)-2-(4-fluorophenyl)aziridine** [(*S*)-**3va**].

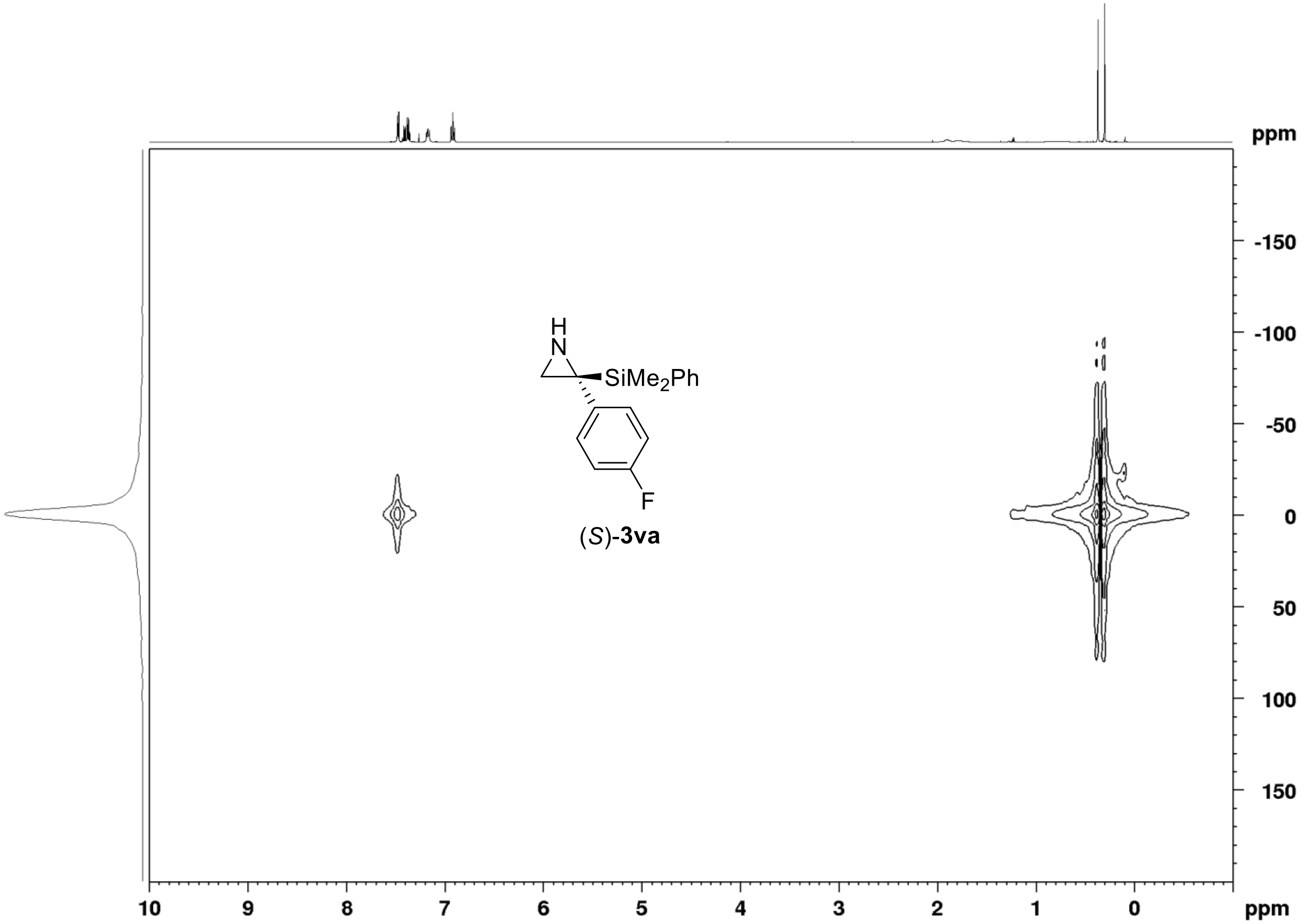

Figure S97. **^{1}H NMR** (500 MHz, $CDCl_3$, 298 K) of **(*S*)-2-(3-chlorophenyl)-2-(dimethyl(phenyl)silyl)aziridine** [(*S*)-**3wa**].

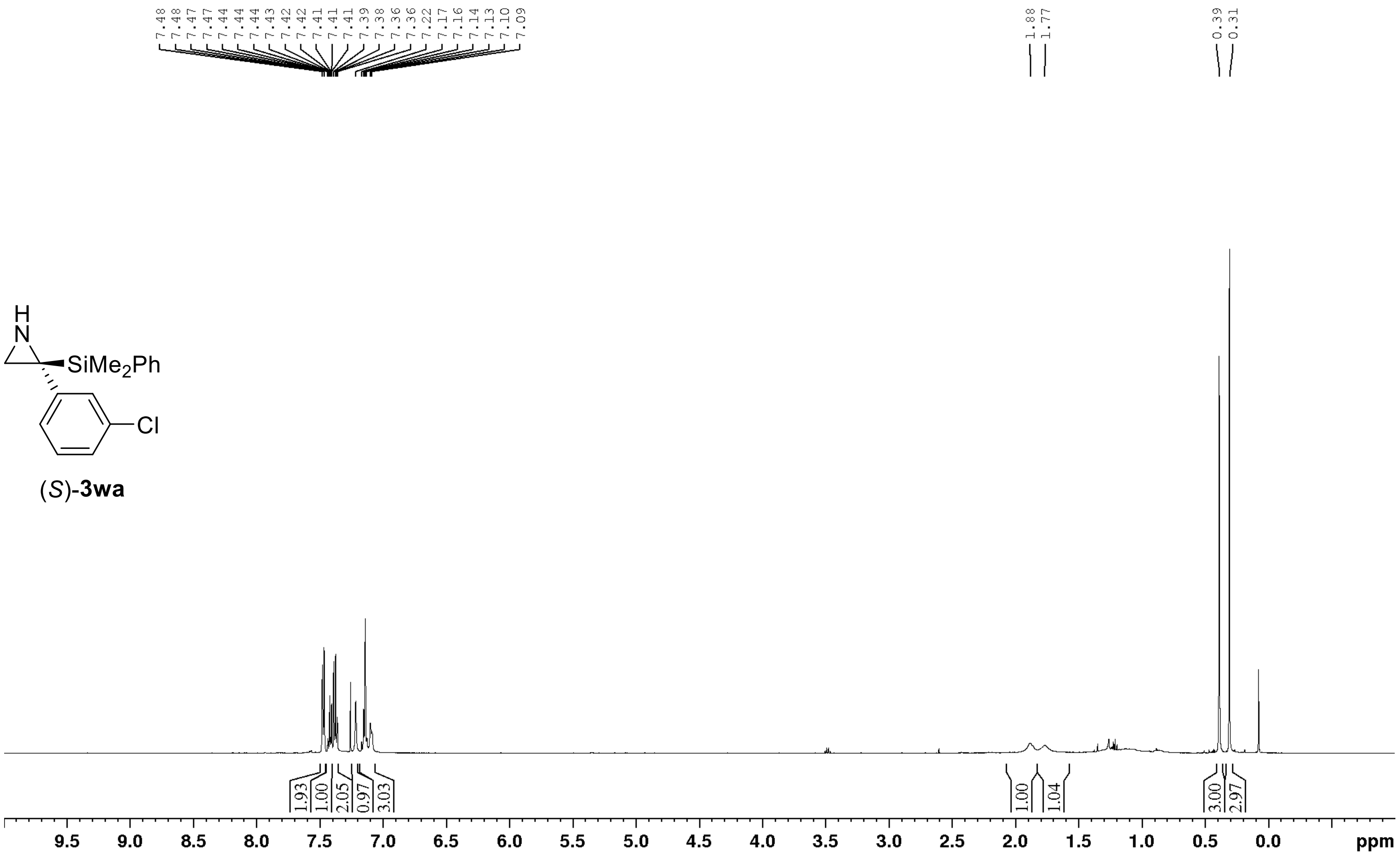

Figure S98. ^{13}C **NMR** (126 MHz, $CDCl_3$, 298 K) of **(*S*)-2-(3-chlorophenyl)-2-(dimethyl(phenyl)silyl)aziridine** [(*S*)-**3wa**].

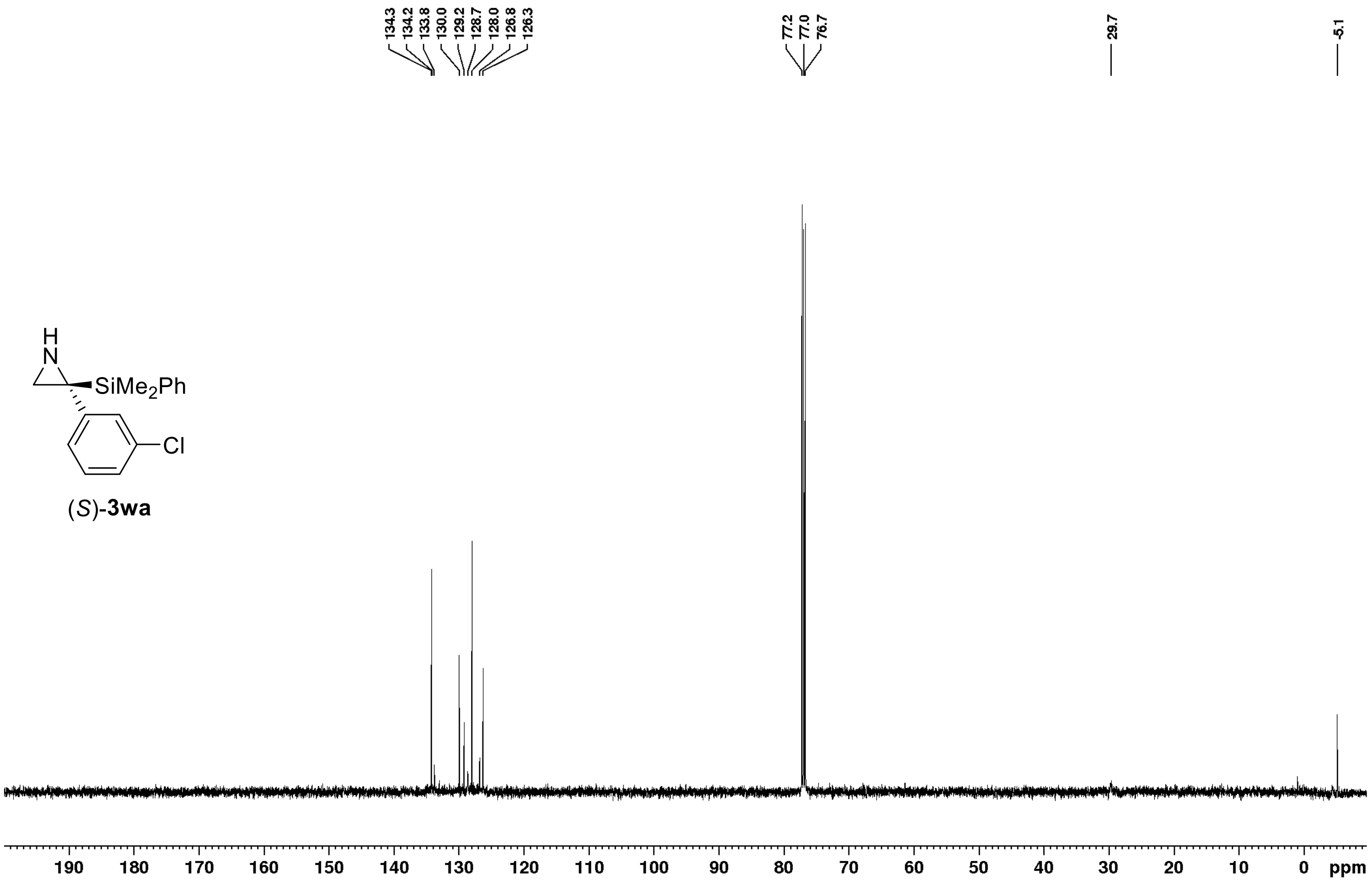

Figure S99. **$^1H/^{29}Si$ HMQC NMR** spectrum (500/99 MHz, $CDCl_3$, 298 K, optimized for J = 7 Hz) of **(*S*)-2-(3-chlorophenyl)-2-(dimethyl(phenyl)silyl)aziridine** [(*S*)-**3wa**].

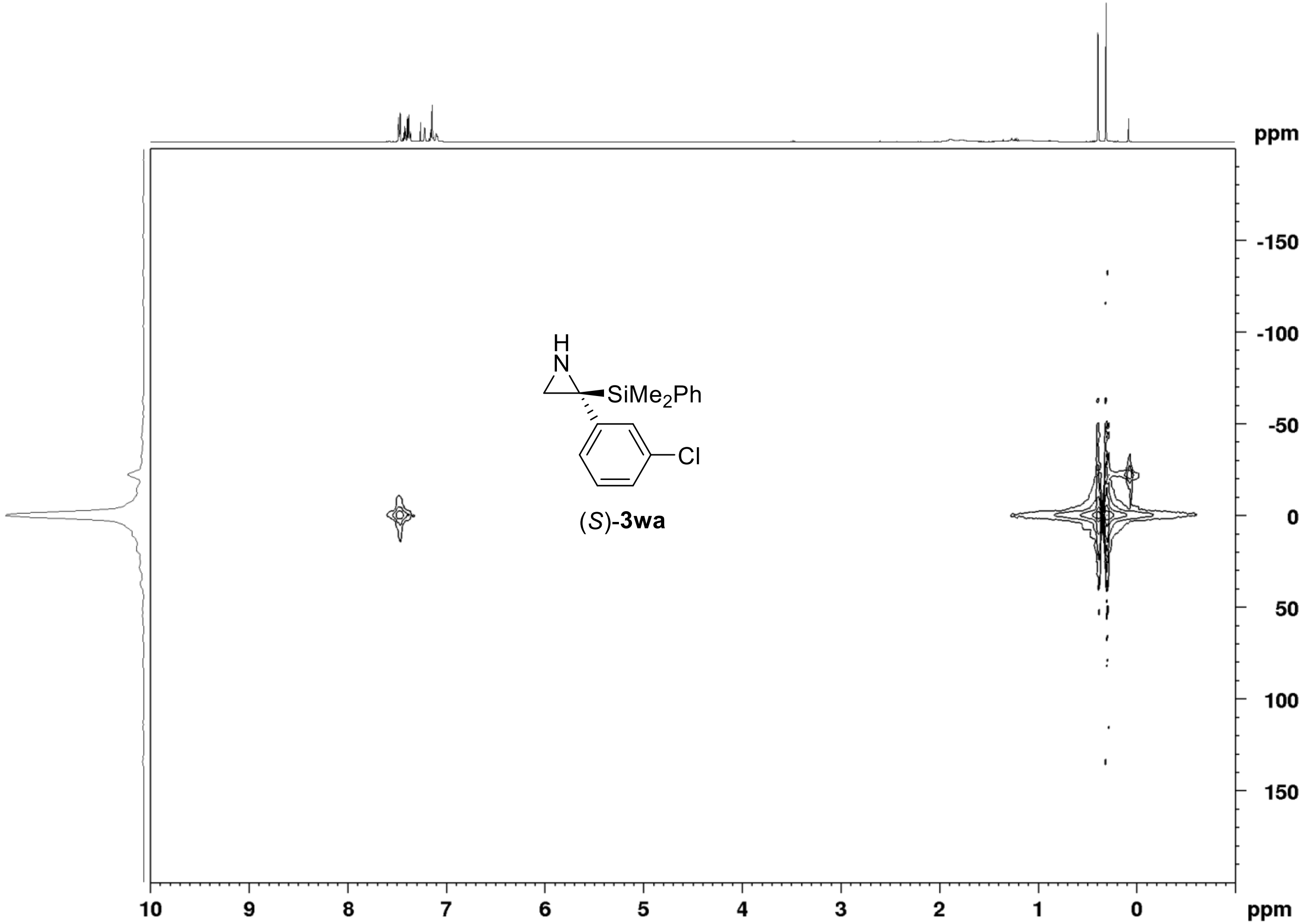

Figure S100. **^{1}H NMR** (500 MHz, $CDCl_3$, 298 K) of **(*S*)-2-(4-chlorophenyl)-2-(dimethyl(phenyl)silyl)aziridine** [(*S*)-**3xa**].

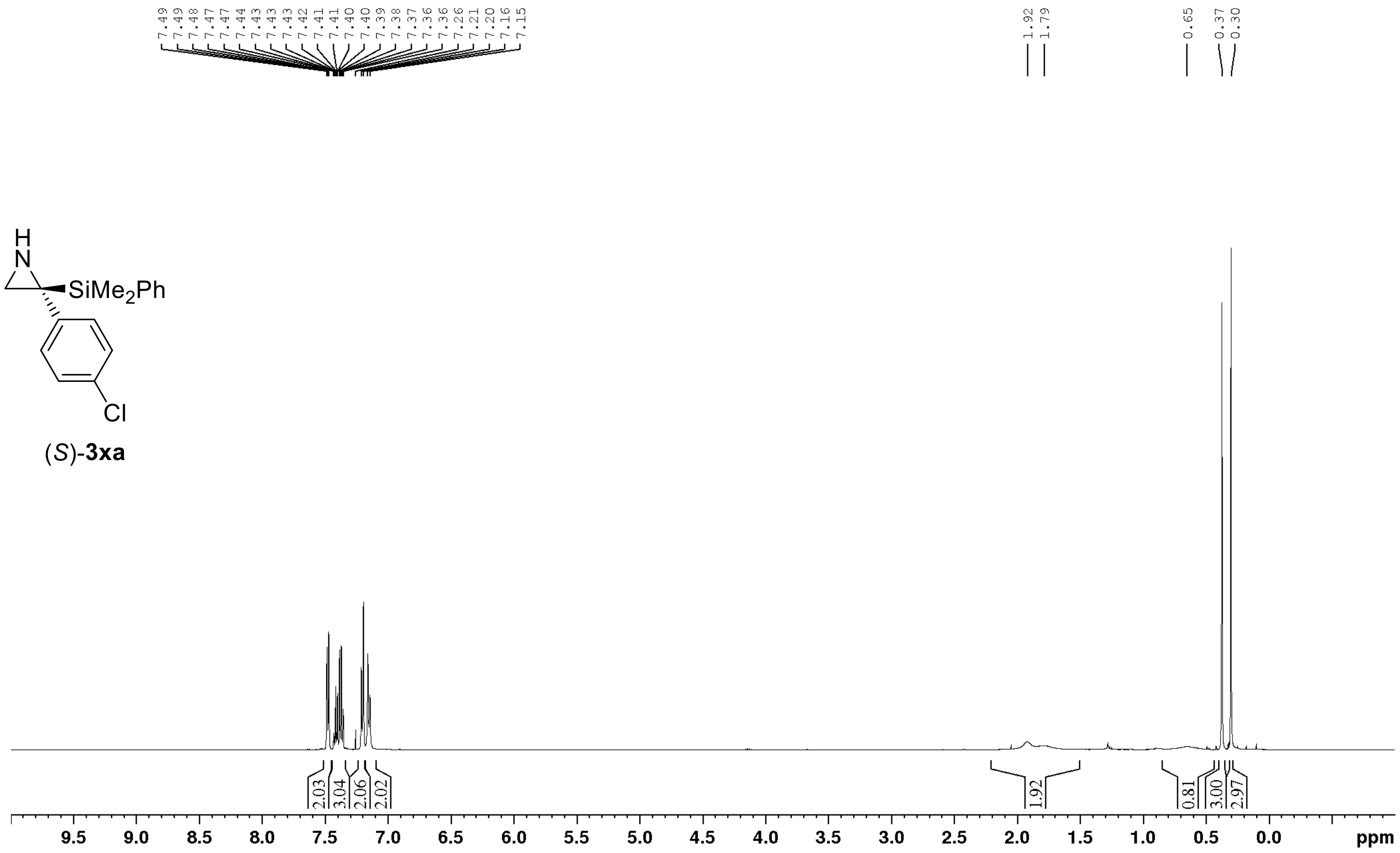

Figure S101. **^{13}C NMR** (126 MHz, $CDCl_3$, 298 K) of **(*S*)-2-(4-chlorophenyl)-2-(dimethyl(phenyl)silyl)aziridine** [(*S*)-**3xa**].

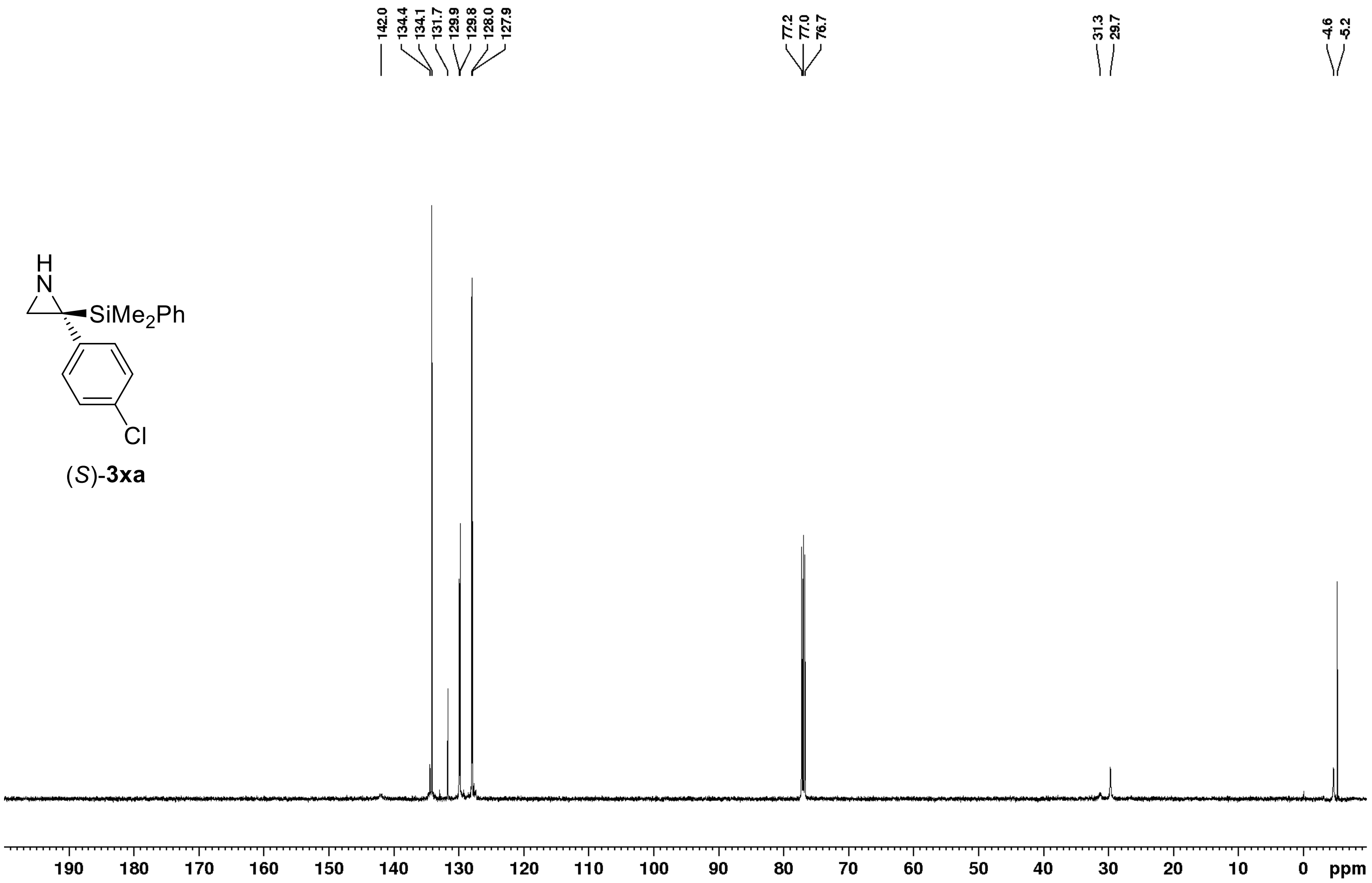

Figure S102. **^{1}H/^{29}Si HMQC NMR** spectrum (500/99 MHz, $CDCl_3$, 298 K, optimized for *J* = 7 Hz) of **(*S*)-2-(4-chlorophenyl)-2-(dimethyl(phenyl)silyl)aziridine** [(*S*)-**3xa**].

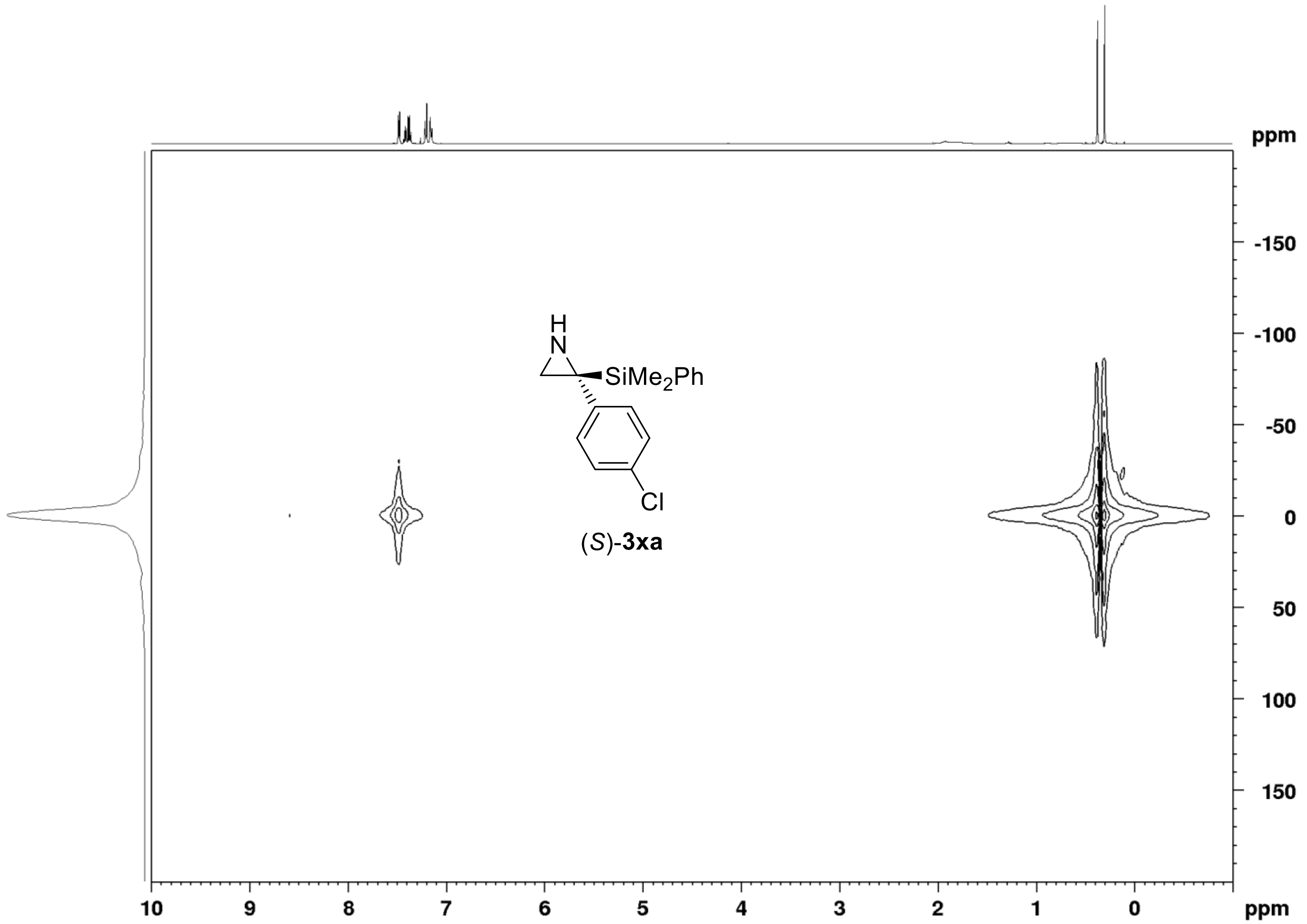

Figure S103. **^{1}H NMR** (500 MHz, $CDCl_3$, 298 K) of **(*S*)-2-(3-bromophenyl)-2-(dimethyl(phenyl)silyl)aziridine** [(*S*)-**3ya**].

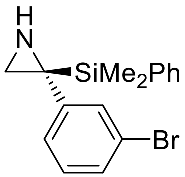

(*S*)-**3ya**

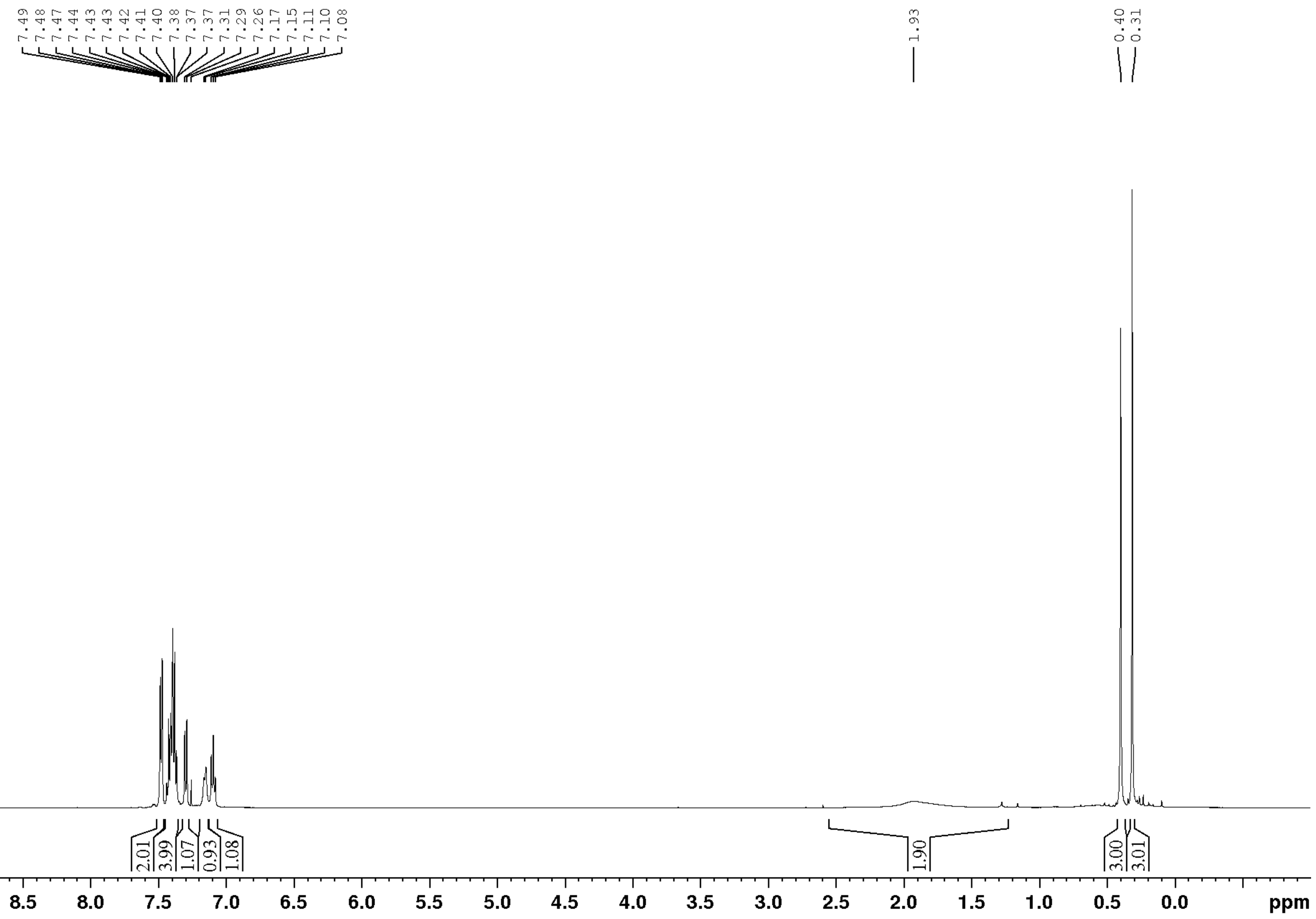

Figure S104. **^{13}C NMR** (126 MHz, $CDCl_3$, 298 K) of **(*S*)-2-(3-bromophenyl)-2-(dimethyl(phenyl)silyl)aziridine** [(*S*)-**3ya**].

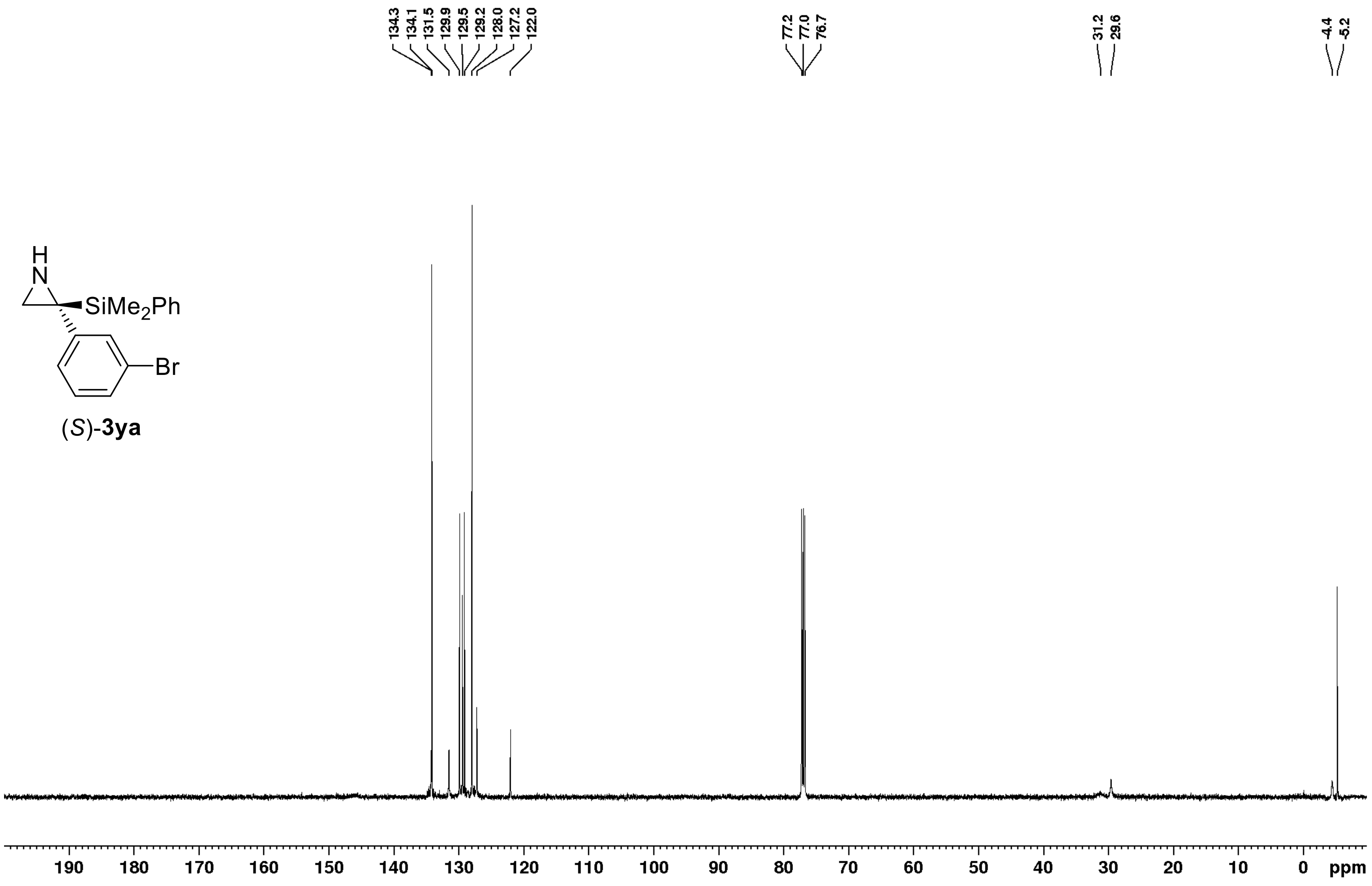

Figure S105. **$^{1}H/^{29}Si$ HMQC NMR** spectrum (500/99 MHz, $CDCl_3$, 298 K, optimized for J = 7 Hz) of **(*S*)-2-(3-bromophenyl)-2-(dimethyl(phenyl)silyl)aziridine** [(*S*)-**3ya**].

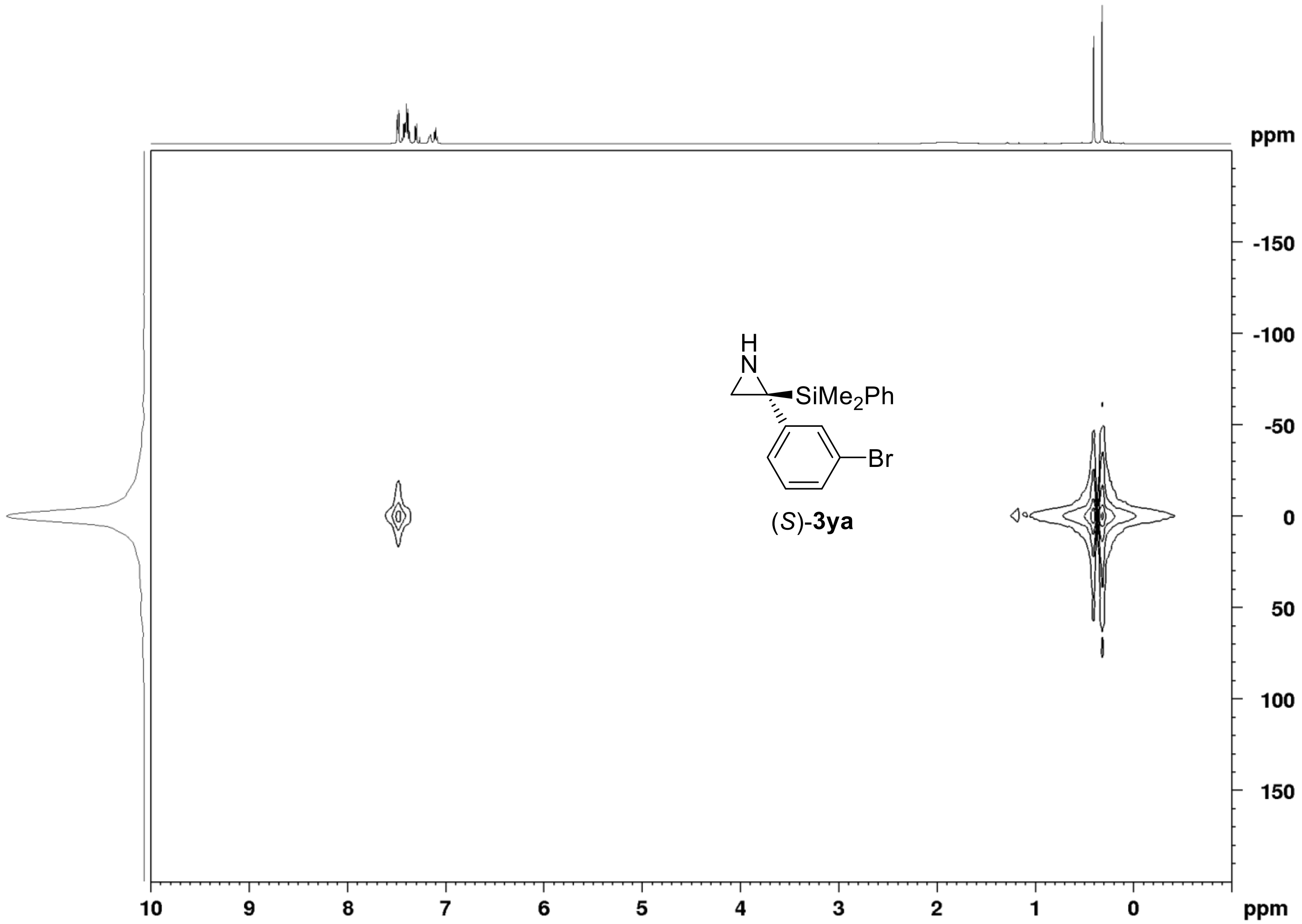

Figure S106. **^{1}H NMR** (500 MHz, $CDCl_3$, 298 K) of **(*S*)-2-(4-bromophenyl)-2-(dimethyl(phenyl)silyl)aziridine** [(*S*)-**3za**].

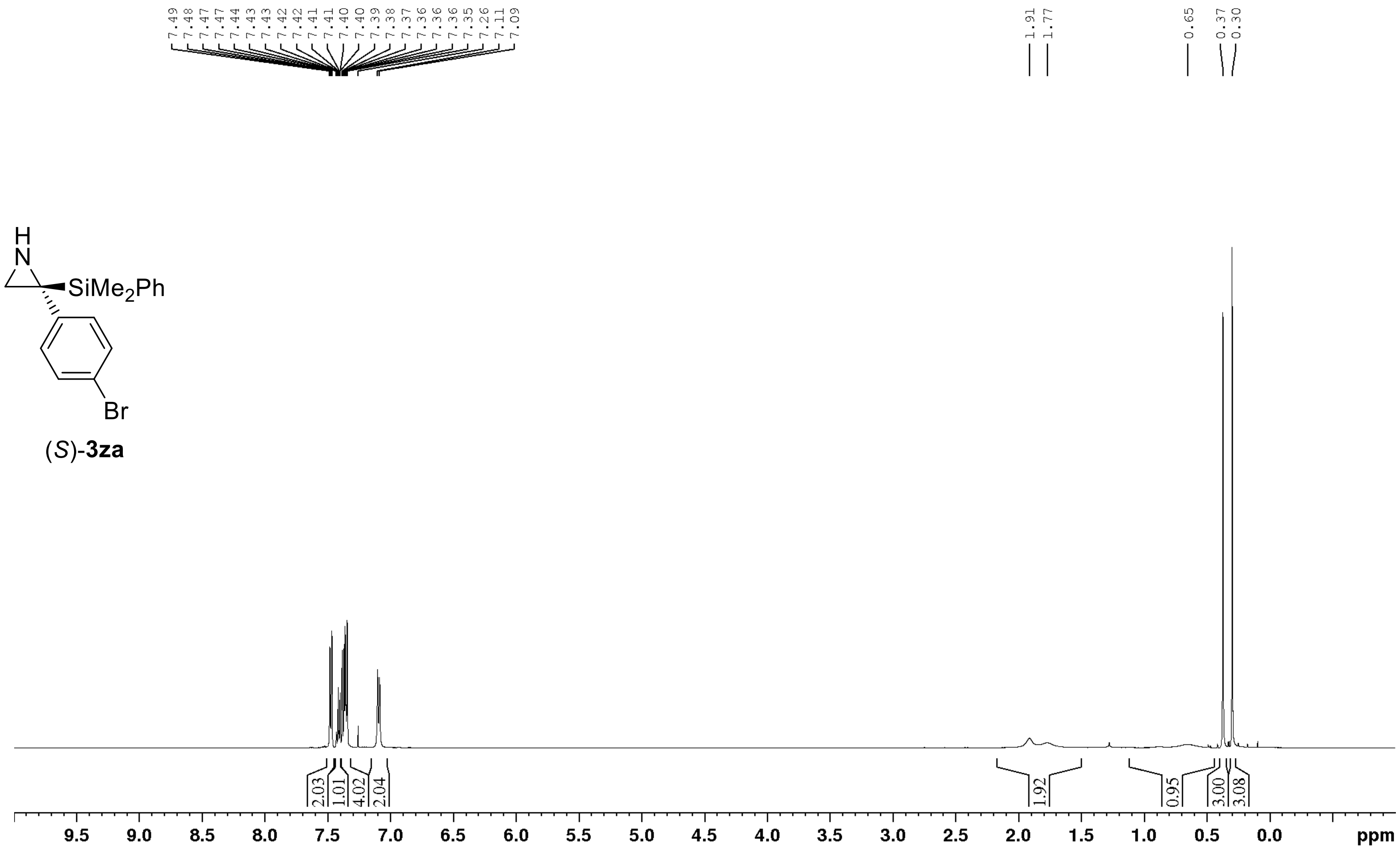

(*S*)-**3za**

Figure S107. **^{13}C NMR** (126 MHz, $CDCl_3$, 298 K) of **(*S*)-2-(4-bromophenyl)-2-(dimethyl(phenyl)silyl)aziridine** [(*S*)-**3za**].

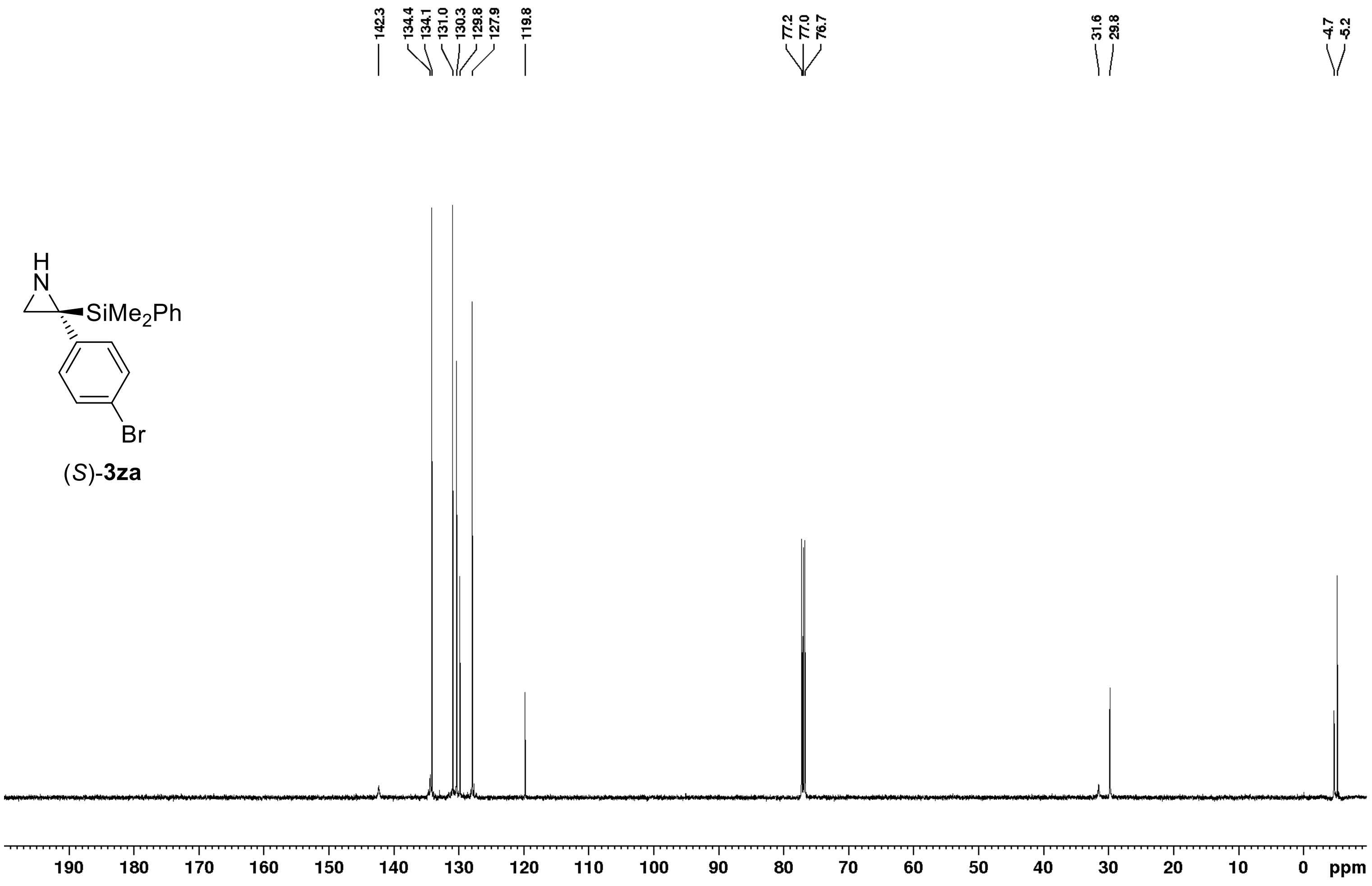

Figure S108. **$^{1}H/^{29}Si$ HMQC NMR** spectrum (500/99 MHz, $CDCl_3$, 298 K, optimized for *J* = 7 Hz) of **(*S*)-2-(4-bromophenyl)-2-(dimethyl(phenyl)silyl)aziridine** [(*S*)-**3za**].

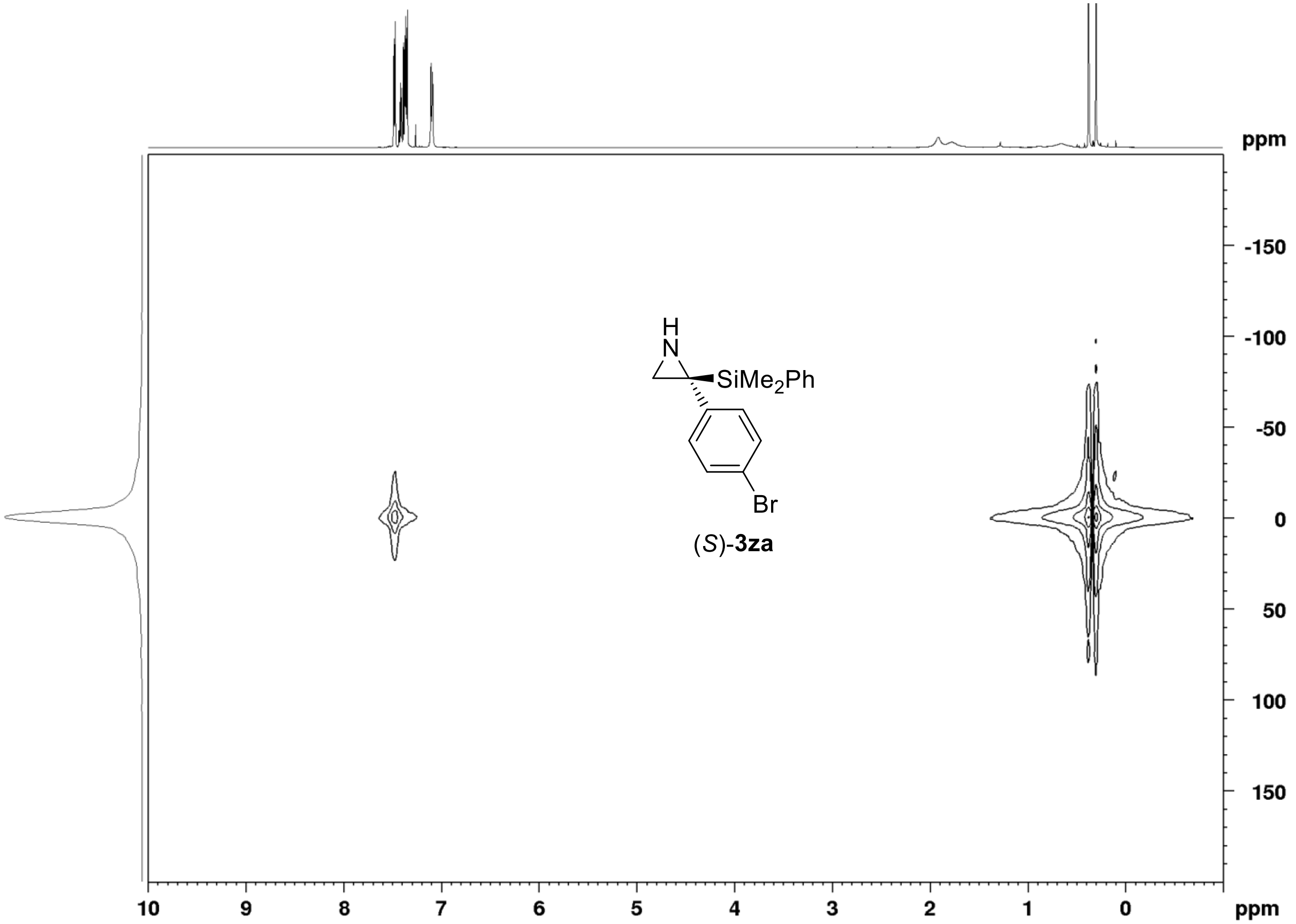

Figure S109. ^{1}**H NMR** (500 MHz, $CDCl_3$, 298 K) of **(*S*)-2-(2,4-difluorophenyl)-2-(dimethyl(phenyl)silyl)aziridine** [(*S*)-**3a'a**].

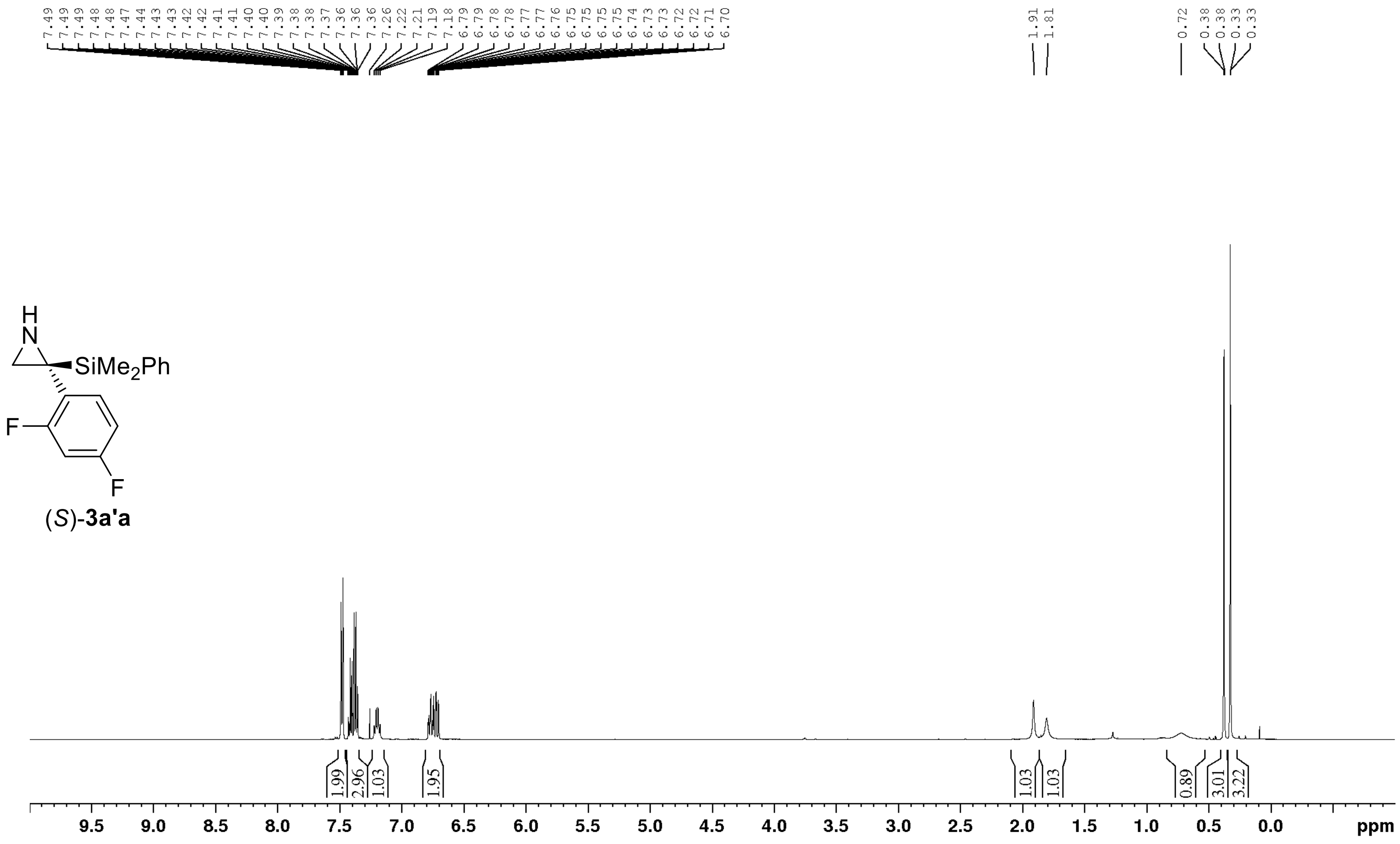

Figure S110. **^{13}C NMR** (126 MHz, $CDCl_3$, 298 K) of **(*S*)-2-(2,4-difluorophenyl)-2-(dimethyl(phenyl)silyl)aziridine** [(*S*)-**3a'a**].

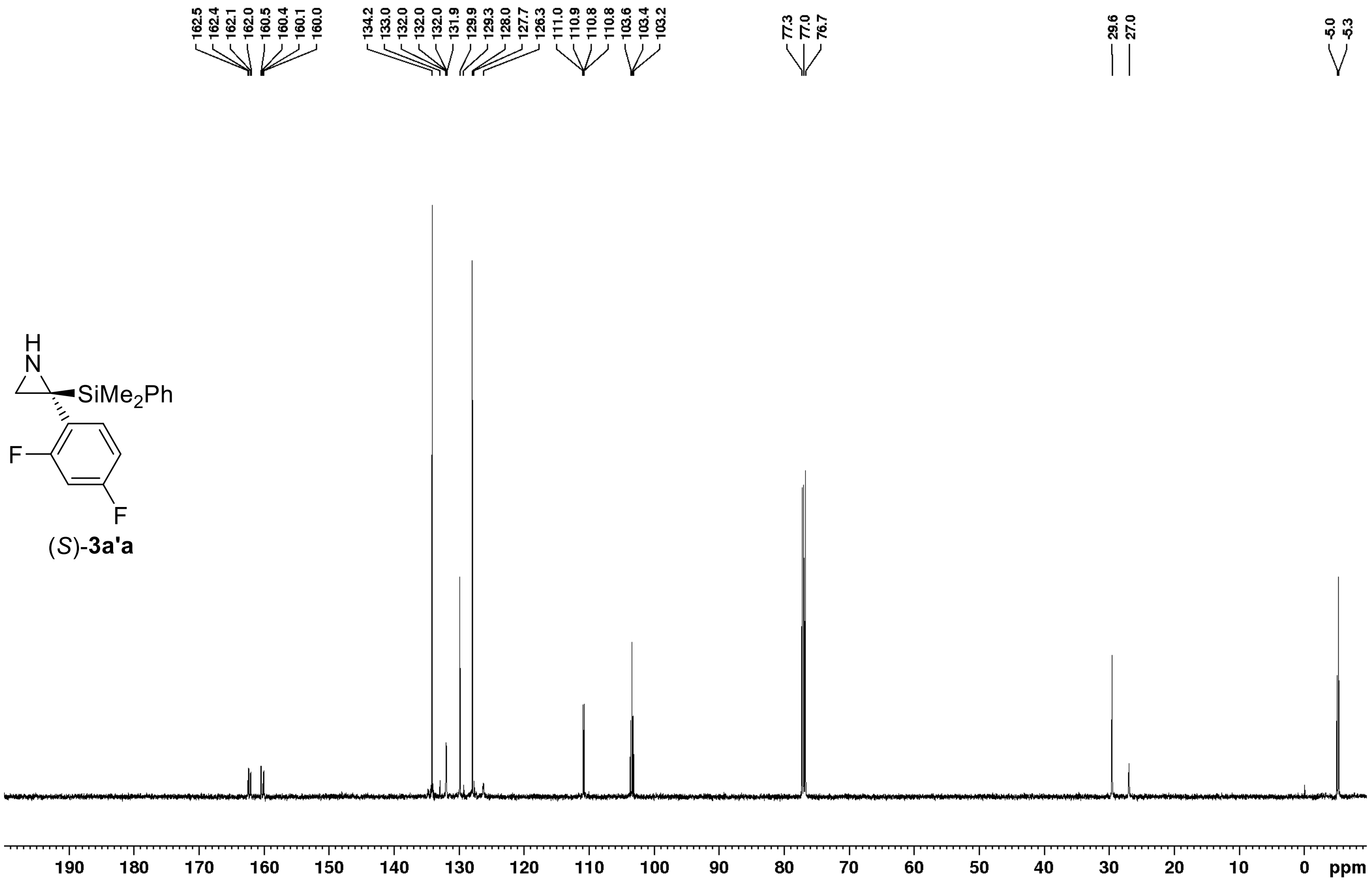

Figure S111. **^{19}F NMR** spectrum (471 MHz, $CDCl_3$, 298 K) of **(*S*)-2-(2,4-difluorophenyl)-2-(dimethyl(phenyl)silyl)aziridine** [(*S*)-**3a'a**].

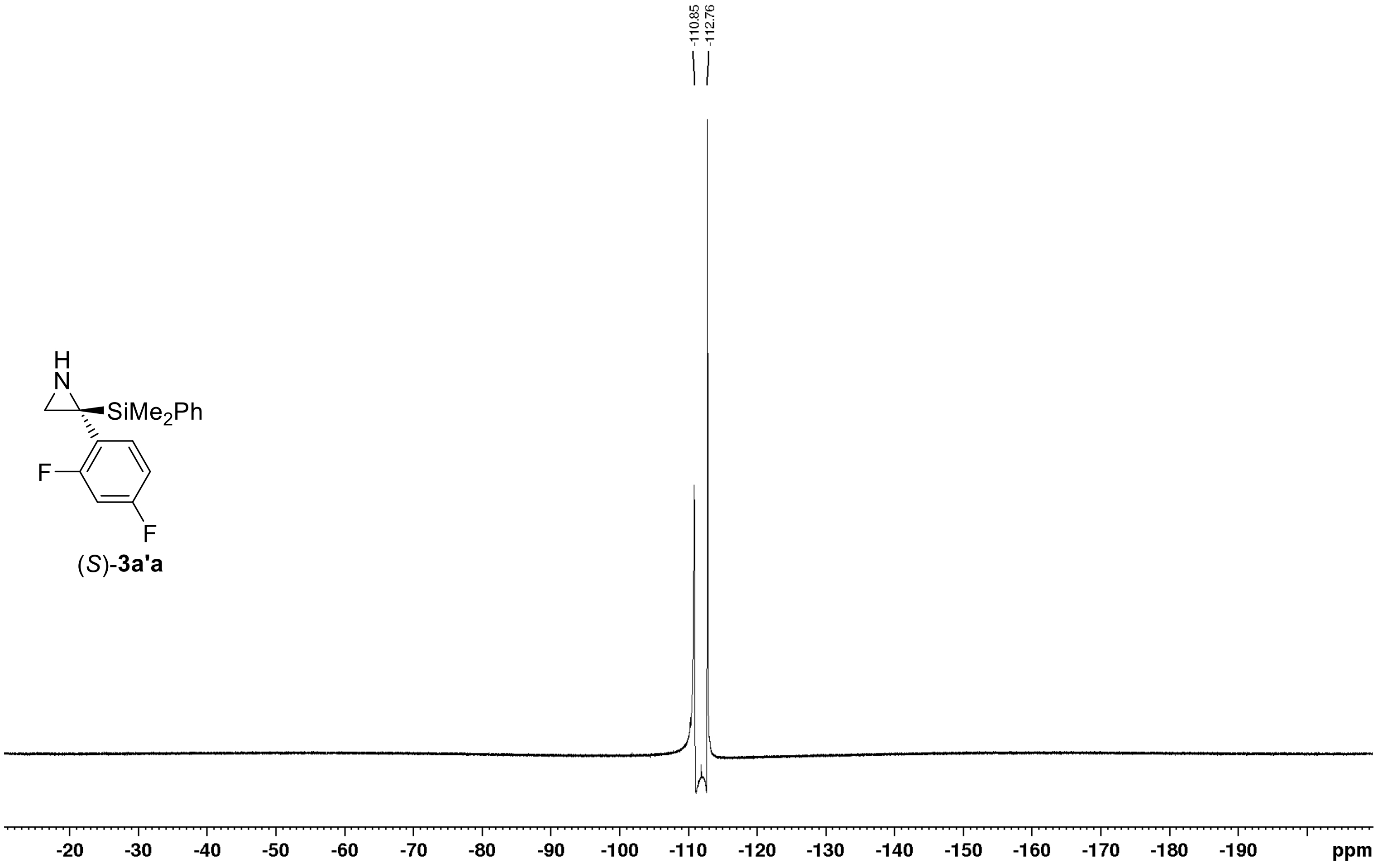

Figure S112. **$^1H/^{29}Si$ HMQC NMR** spectrum (500/99 MHz, $CDCl_3$, 298 K, optimized for *J* = 7 Hz) of **(*S*)-2-(2,4-difluorophenyl)-2-(dimethyl(phenyl)silyl)aziridine** [(*S*)-**3a’a**].

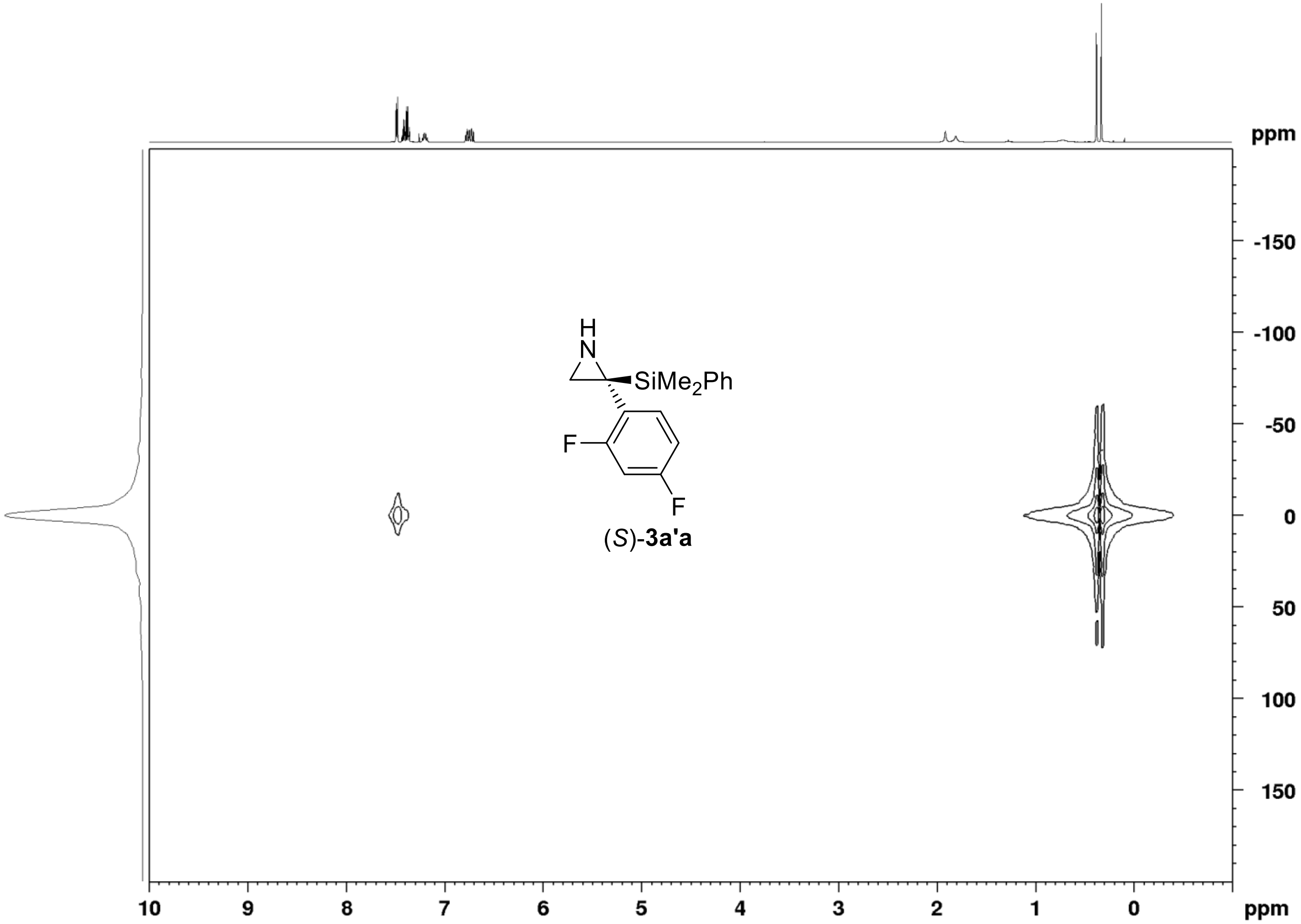

Figure S113. **^{1}H NMR** (500 MHz, $CDCl_3$, 298 K) of **(*S*)-2-(2-chloro-4-fluorophenyl)-2-(dimethyl(phenyl)silyl)aziridine** [(*S*)-**3b'a**].

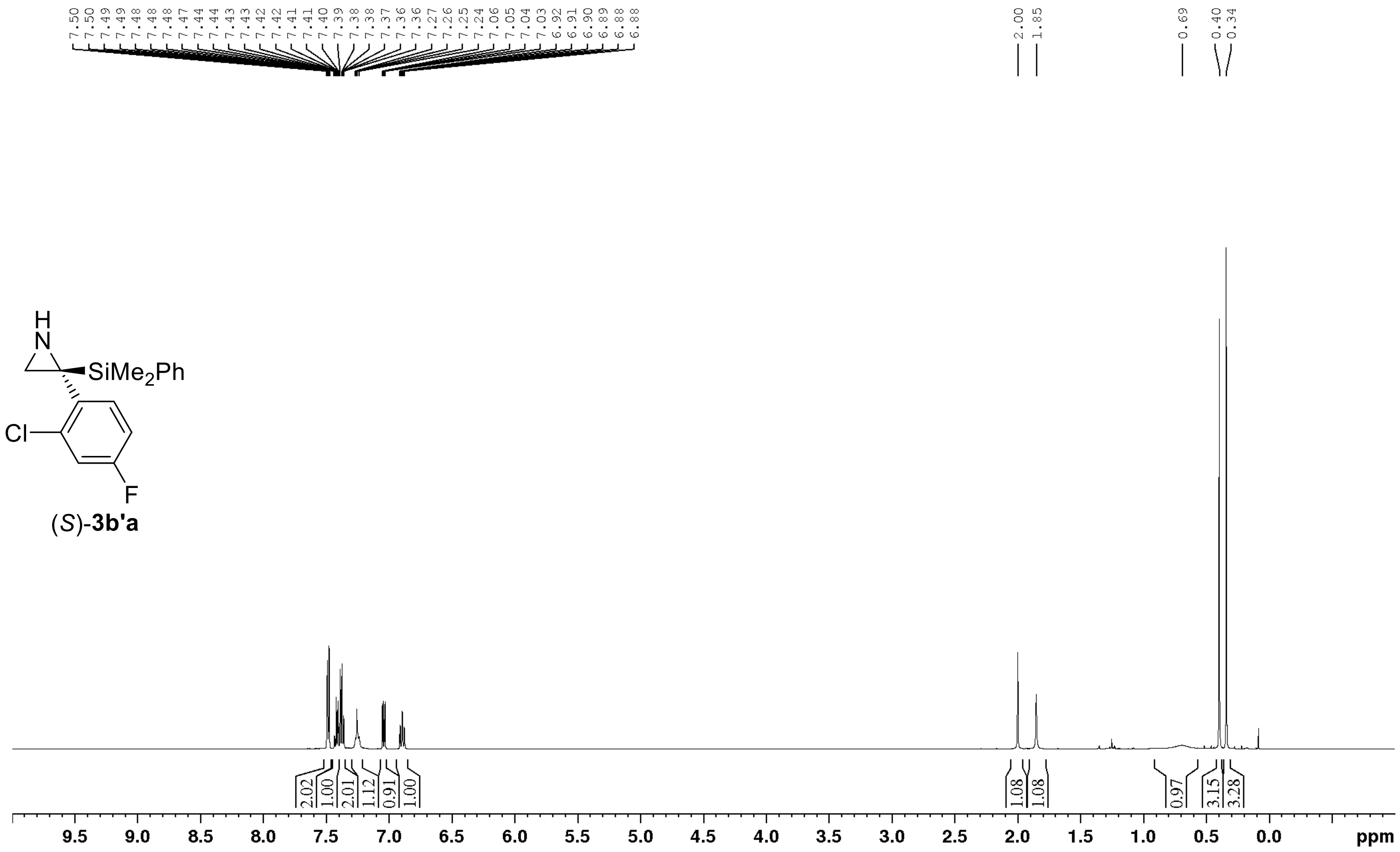

Figure S114. **^{13}C NMR** (126 MHz, $CDCl_3$, 298 K) of **(*S*)-2-(2-chloro-4-fluorophenyl)-2-(dimethyl(phenyl)silyl)aziridine** [(*S*)-**3b'a**].

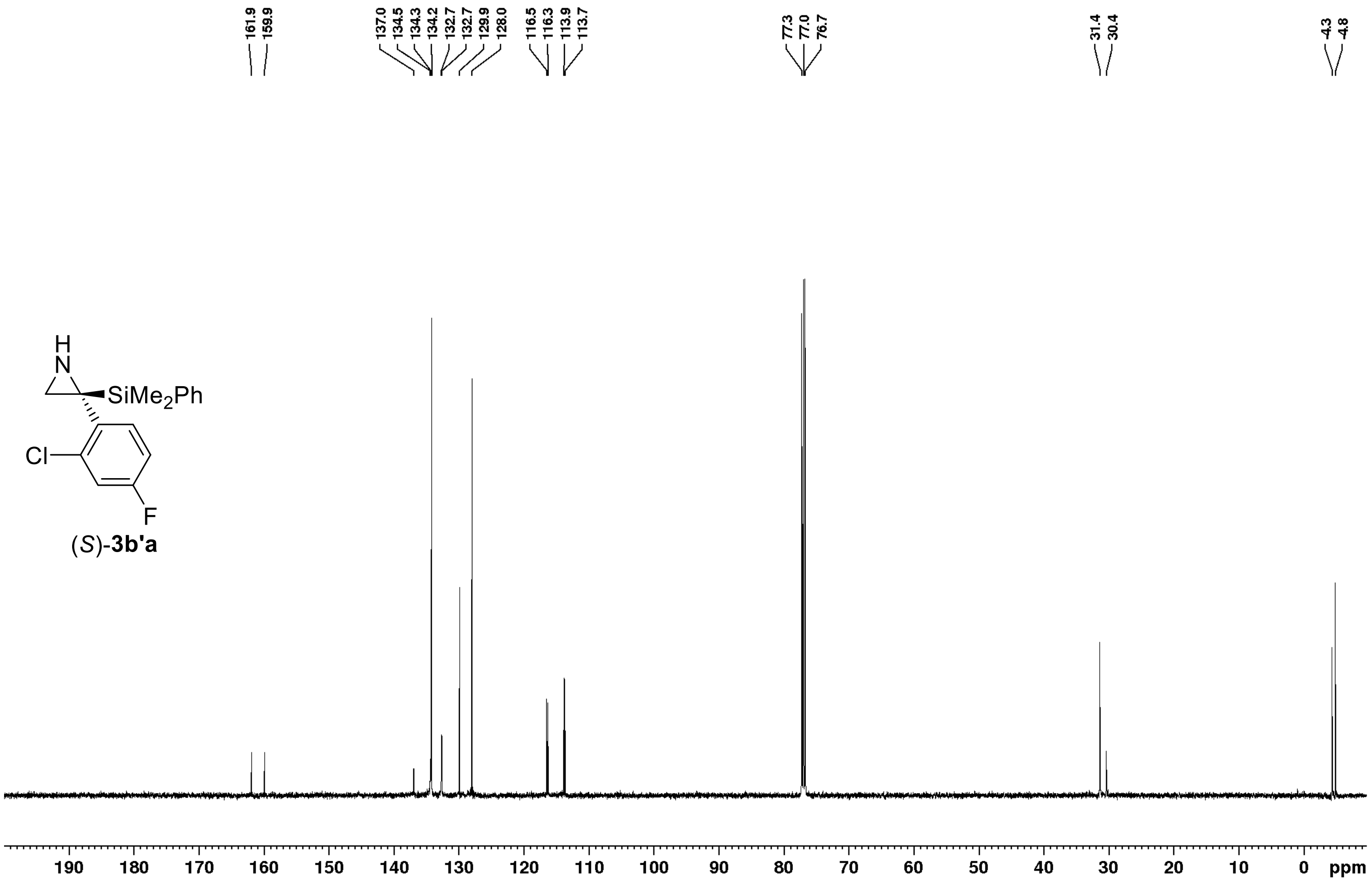

(*S*)-**3b'a**

Figure S115. **^{19}F NMR** spectrum (471 MHz, $CDCl_3$, 298 K) of **(*S*)-2-(2-chloro-4-fluorophenyl)-2-(dimethyl(phenyl)silyl)aziridine** [(*S*)-**3b'a**].

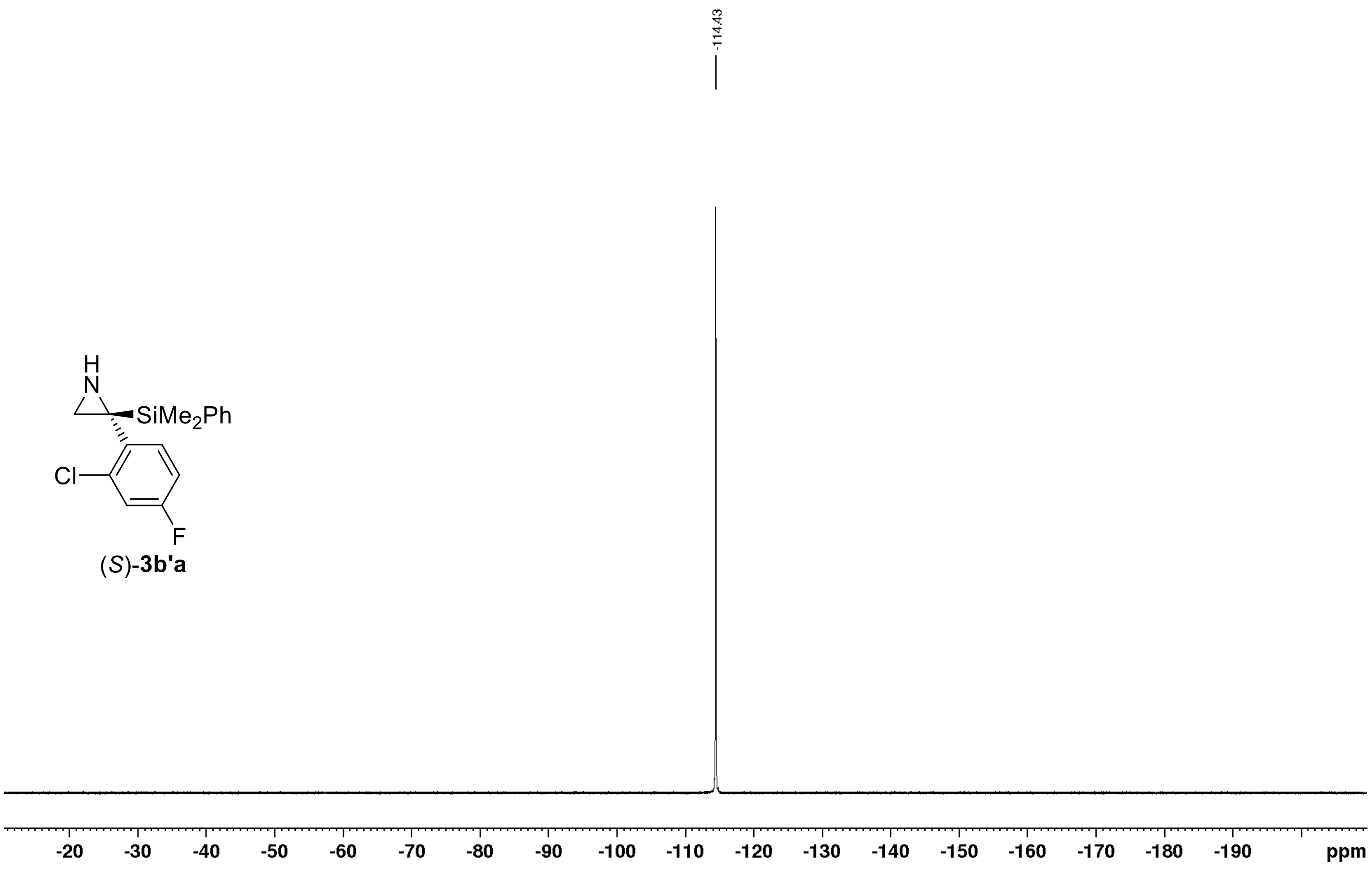

Figure S116. **^{1}H/^{29}Si HMQC NMR** spectrum (500/99 MHz, $CDCl_3$, 298 K, optimized for *J* = 7 Hz) of **(*S*)-2-(2-chloro-4-fluorophenyl)-2-(dimethyl(phenyl)silyl)aziridine** [(*S*)-**3b'a**].

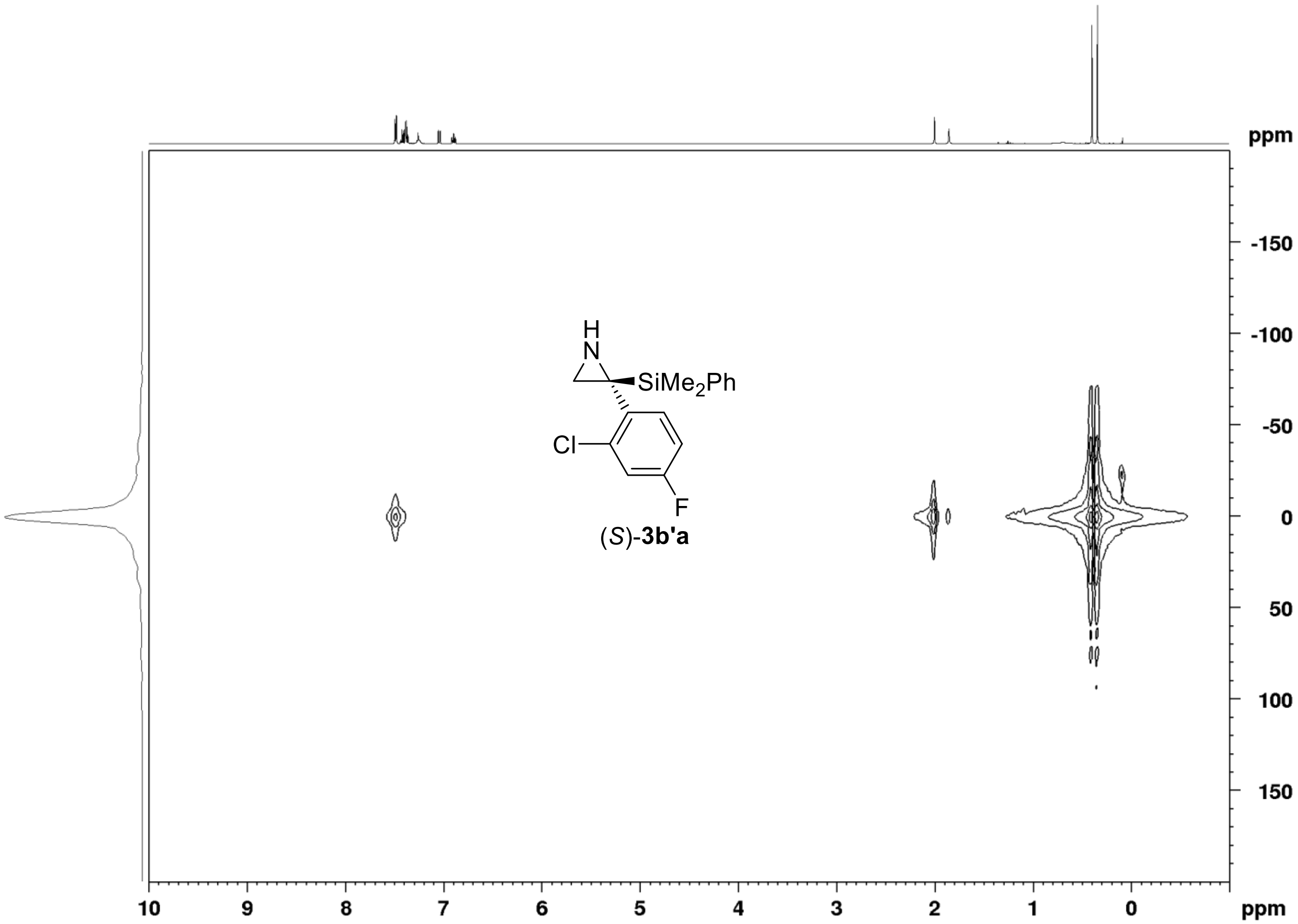

Figure S117. **^{1}H NMR** (500 MHz, $CDCl_3$, 298 K) of **(*S*)-2-(dimethyl(phenyl)silyl)-2-(thiophen-3-yl)aziridine** [(*S*)-**3c'a**].

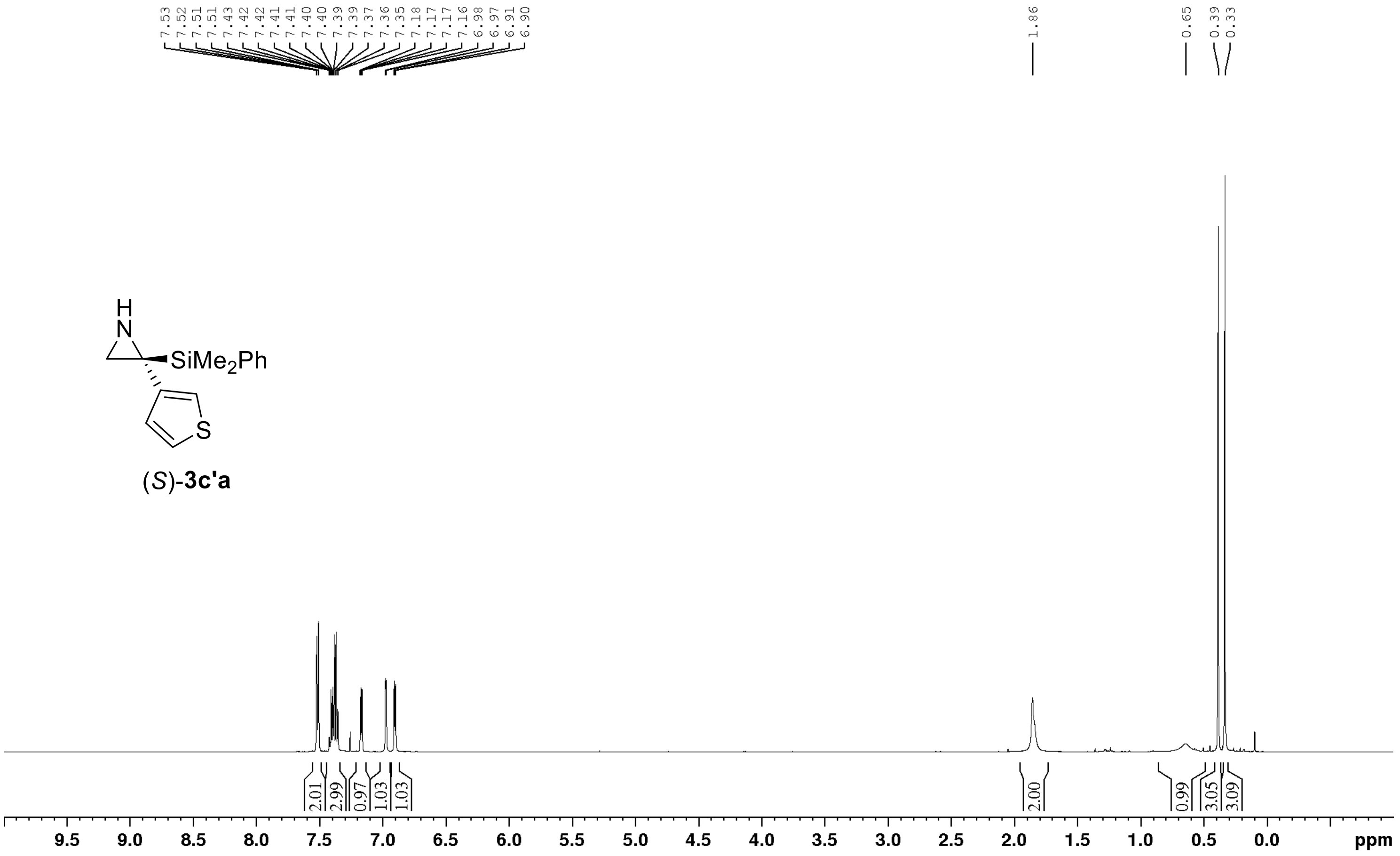

Figure S118. **^{13}C NMR** (126 MHz, $CDCl_3$, 298 K) of **(*S*)-2-(dimethyl(phenyl)silyl)-2-(thiophen-3-yl)aziridine** [(*S*)-**3c'a**].

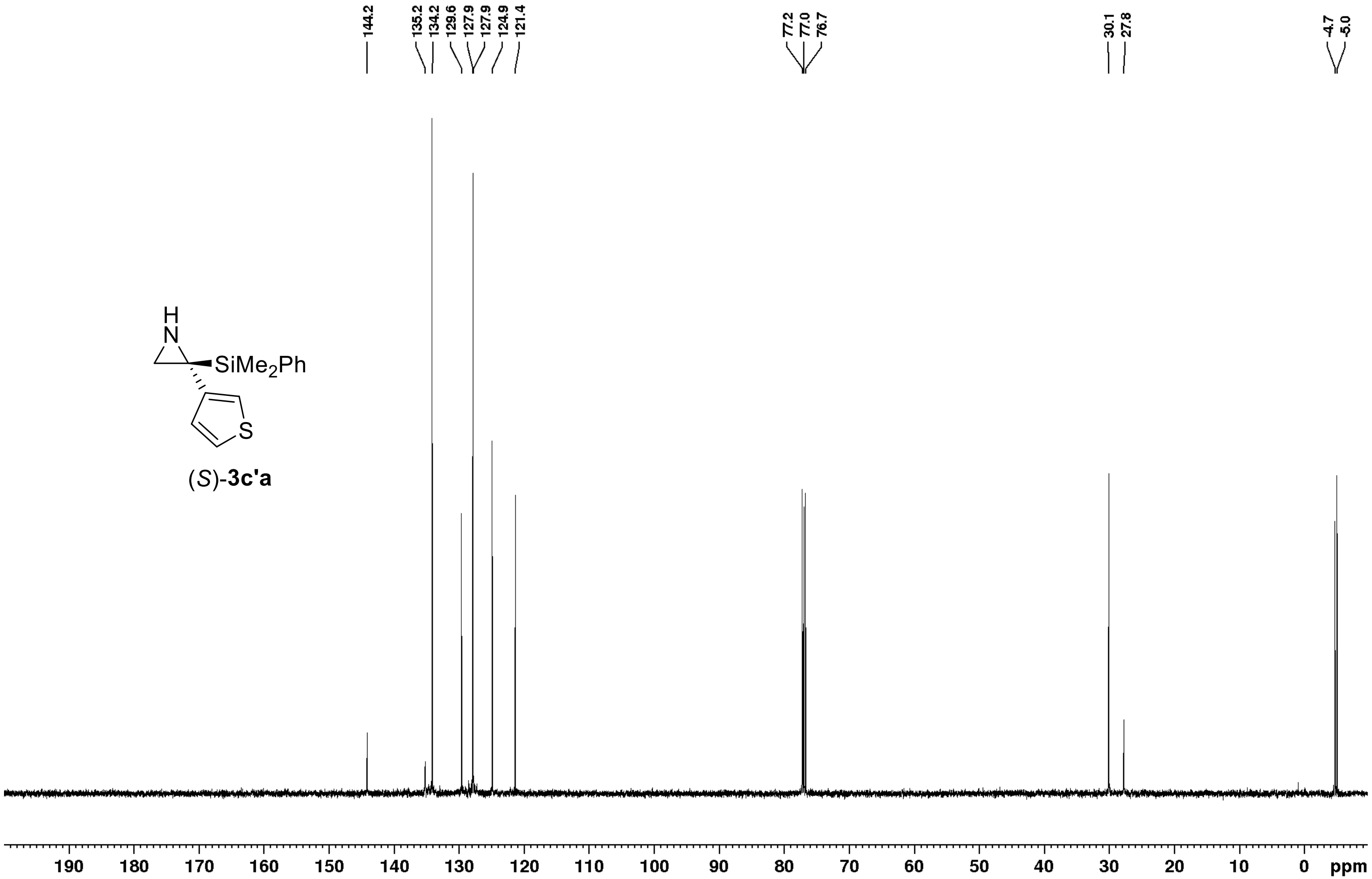

Figure S119. **^{1}H/^{29}Si HMQC NMR** spectrum (500/99 MHz, $CDCl_3$, 298 K, optimized for J = 7 Hz) of **(*S*)-2-(dimethyl(phenyl)silyl)-2-(thiophen-3-yl)aziridine** [(*S*)-**3c'a**].

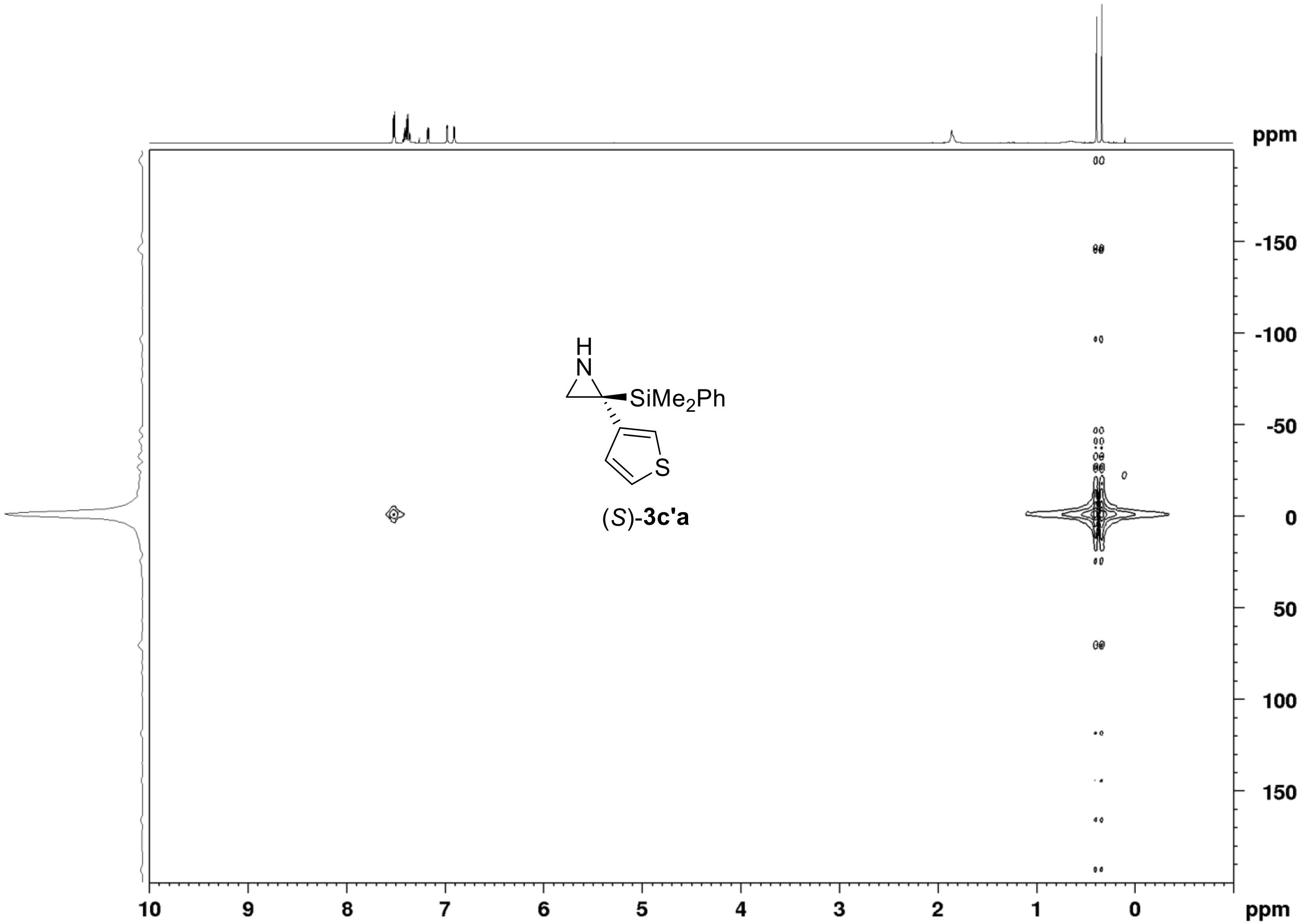

Figure S120. **^{1}H NMR** (500 MHz, $CDCl_3$, 298 K) of **(*S*)-2-cyclohexyl-2-(dimethyl(phenyl)silyl)aziridine** [(*S*)-**3d'a**].

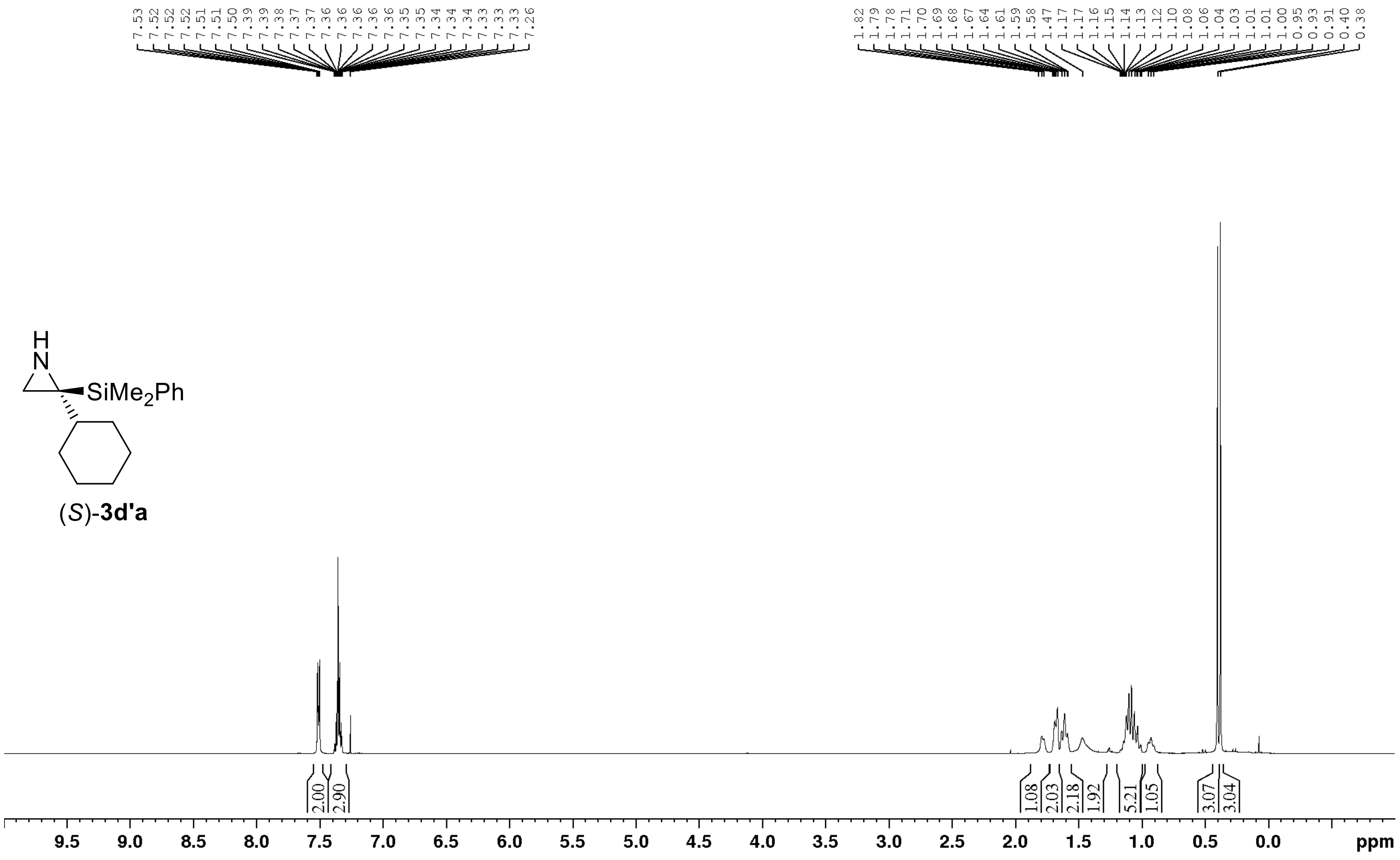

Figure S121. ^{13}**C NMR** (126 MHz, $CDCl_3$, 298 K) of **(*S*)-2-cyclohexyl-2-(dimethyl(phenyl)silyl)aziridine** [(*S*)-**3d'a**].

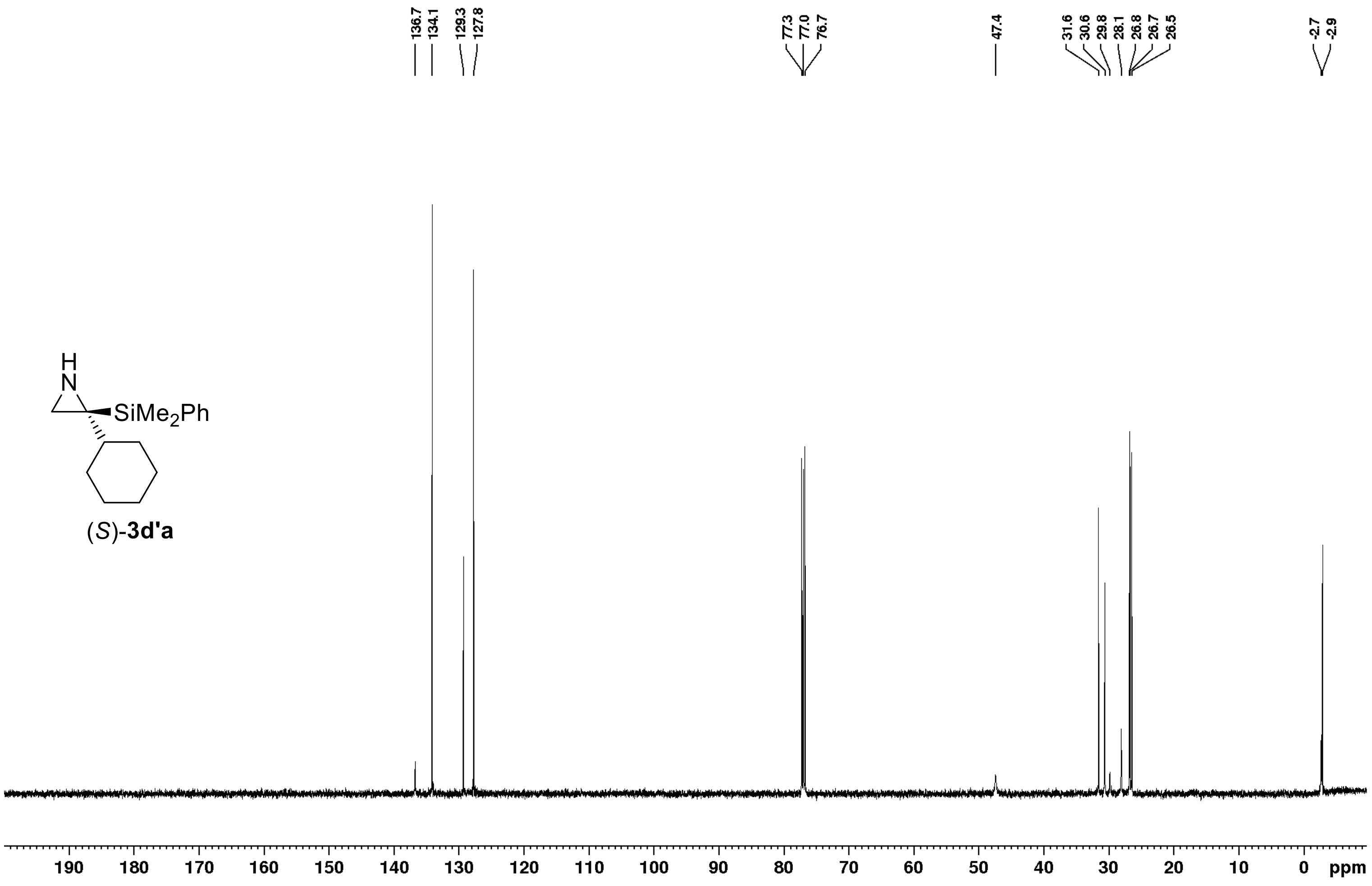

Figure S122. **^{1}H/^{29}Si HMQC NMR** spectrum (500/99 MHz, $CDCl_3$, 298 K, optimized for *J* = 7 Hz) of **(*S*)-2-cyclohexyl-2-(dimethyl(phenyl)silyl)aziridine** [(*S*)-**3d'a**].

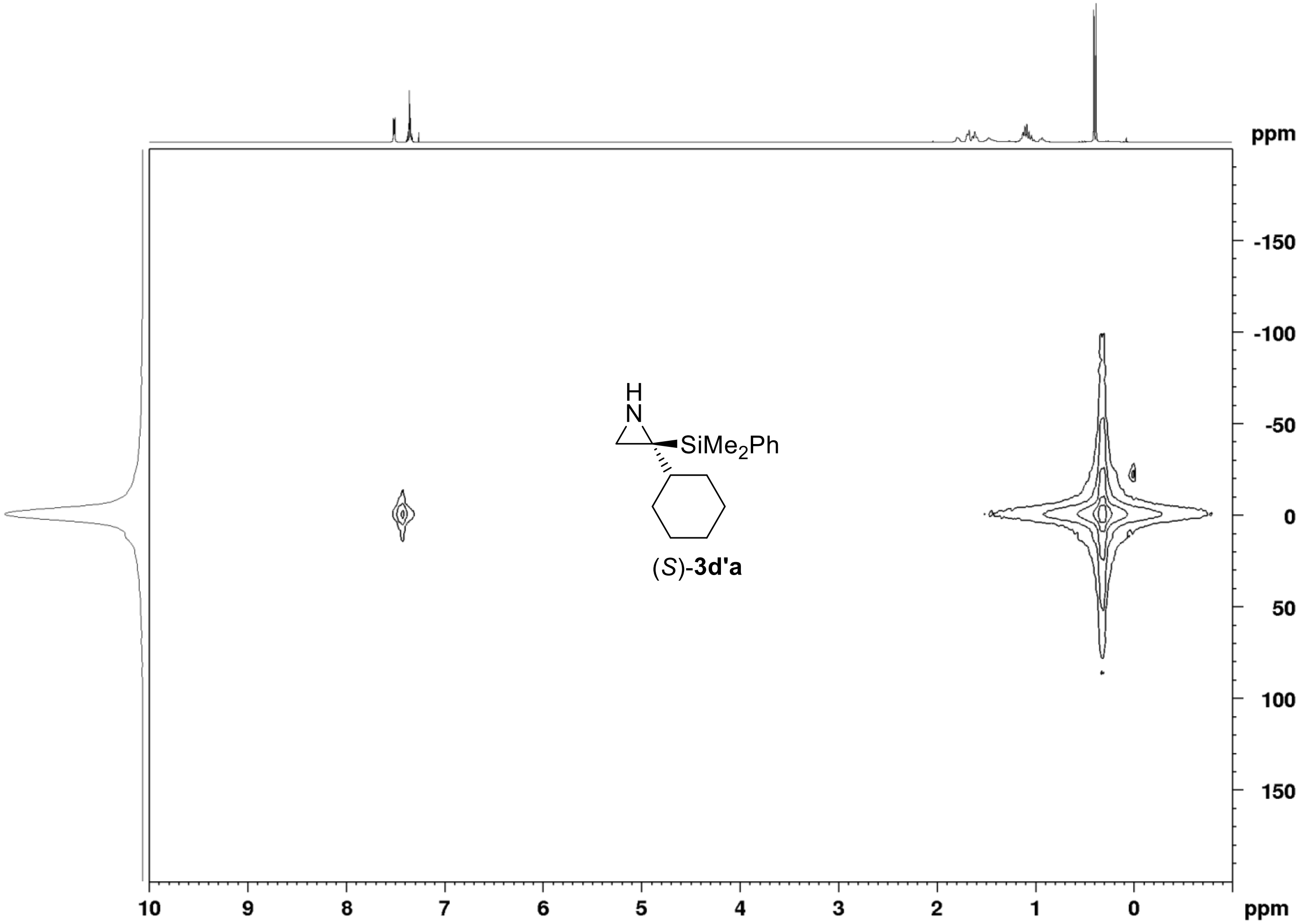

Figure S123. **^{1}H NMR** (500 MHz, $CDCl_3$, 298 K) of **(*S*)-2-(dimethyl(phenyl)silyl)-2-phenethylaziridine** [(*S*)-**3e'a**].

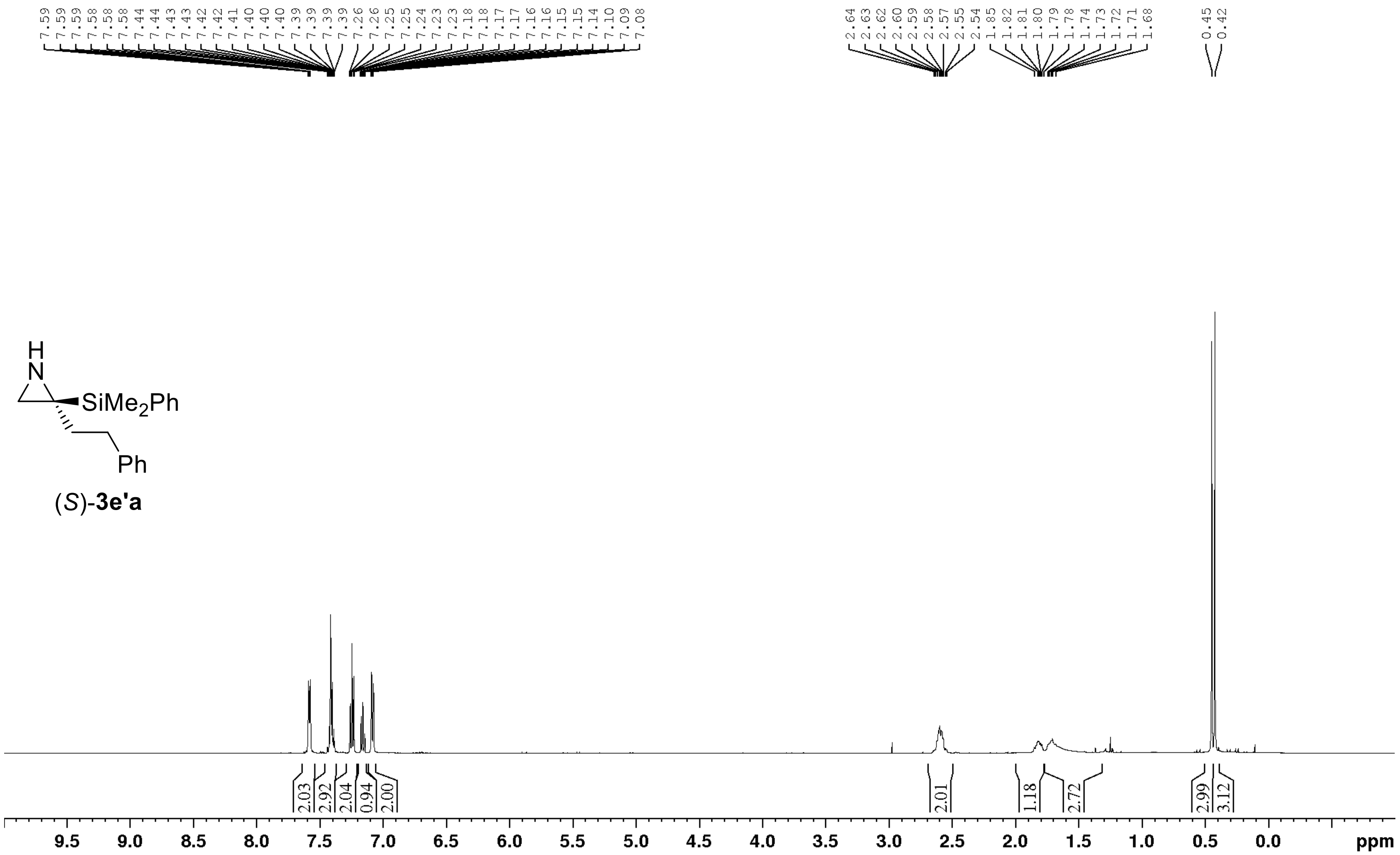

Figure S124. **^{13}C NMR** (126 MHz, CDCl$_3$, 298 K) of **(*S*)-2-(dimethyl(phenyl)silyl)-2-phenethylaziridine** [(*S*)-**3e'a**].

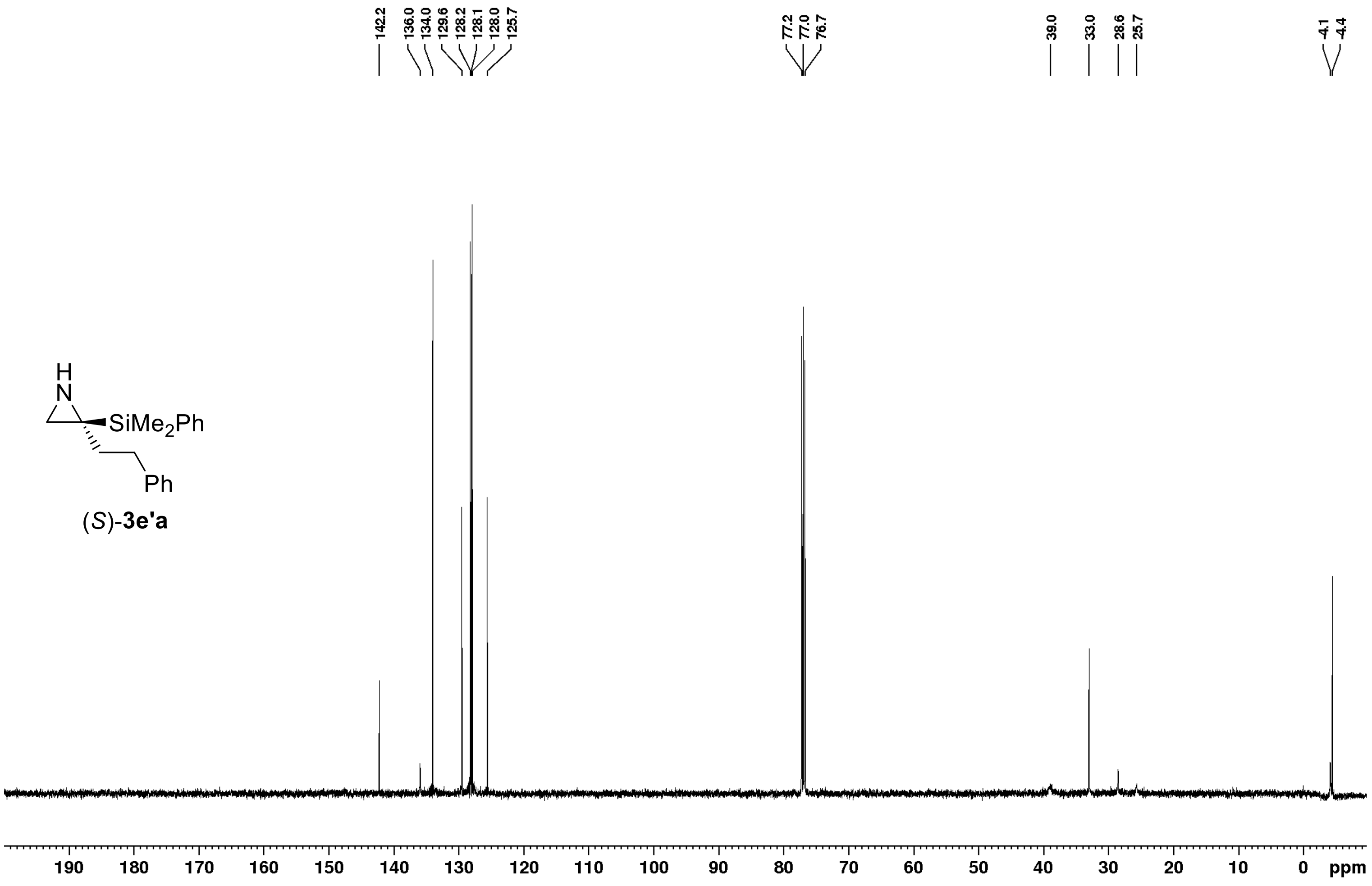

Figure S125. **^{1}H/^{29}Si HMQC NMR** spectrum (500/99 MHz, $CDCl_3$, 298 K, optimized for *J* = 7 Hz) of **(*S*)-2-(dimethyl(phenyl)silyl)-2-phenethylaziridine** [(*S*)-**3e'a**].

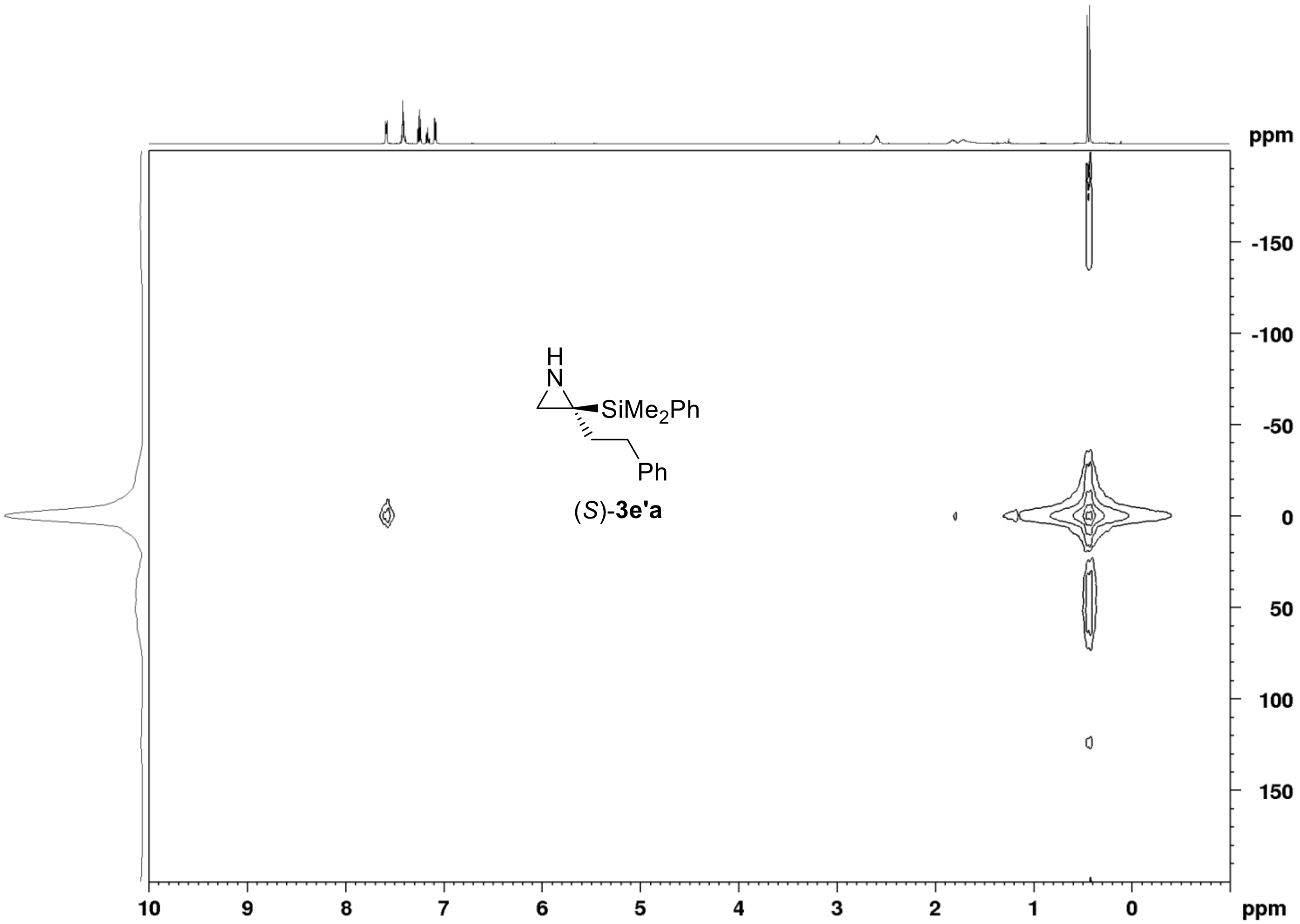

Figure S126. **^{1}H NMR** (500 MHz, $CDCl_3$, 298 K) of **(*S*)-2-(dimethyl(phenyl)silyl)-2-(3-phenylpropyl)aziridine** [(*S*)-**3f'a**].

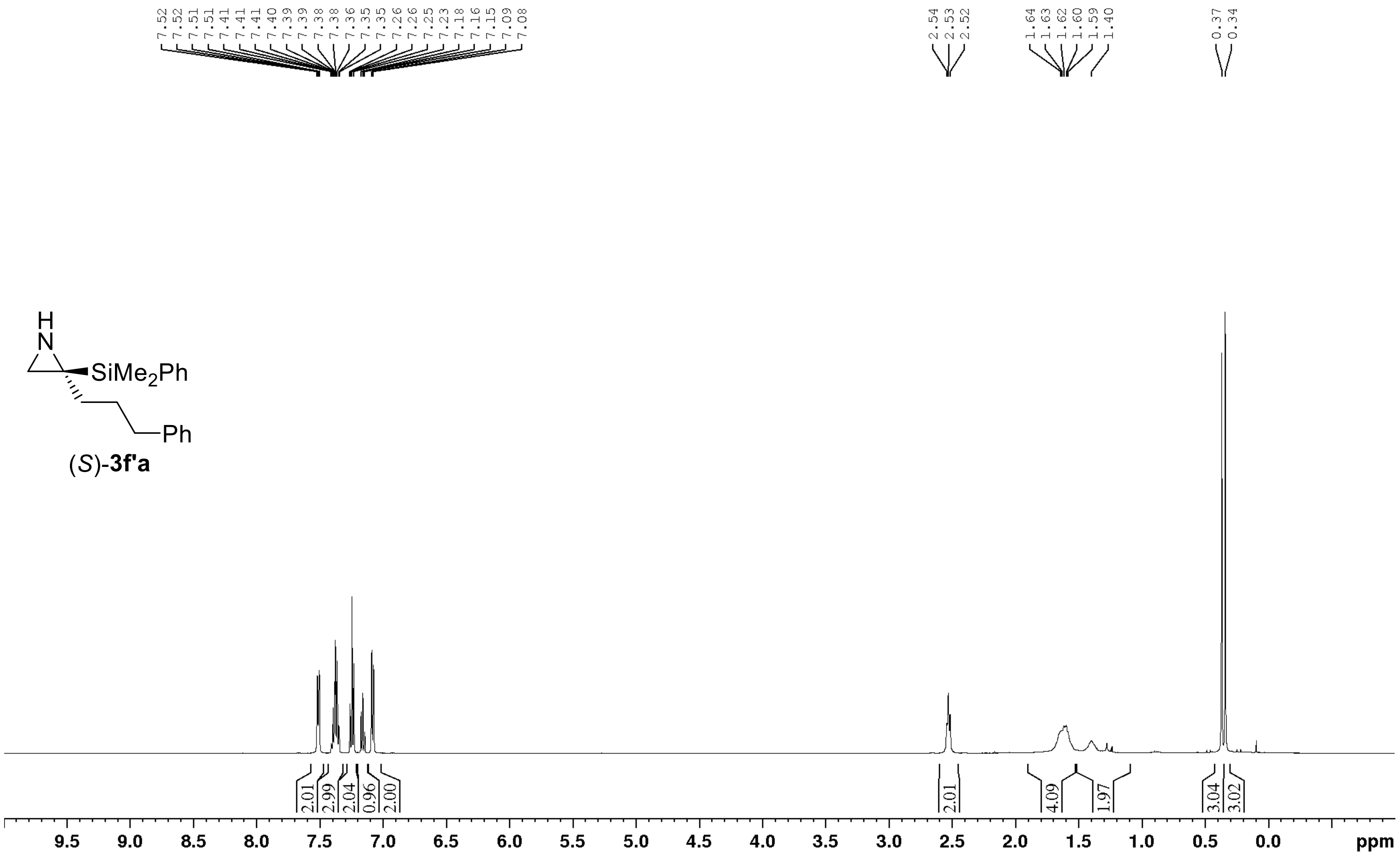

Figure S127. **^{13}C NMR** (126 MHz, $CDCl_3$, 298 K) of **(*S*)-2-(dimethyl(phenyl)silyl)-2-(3-phenylpropyl)aziridine** [(*S*)-**3f'a**].

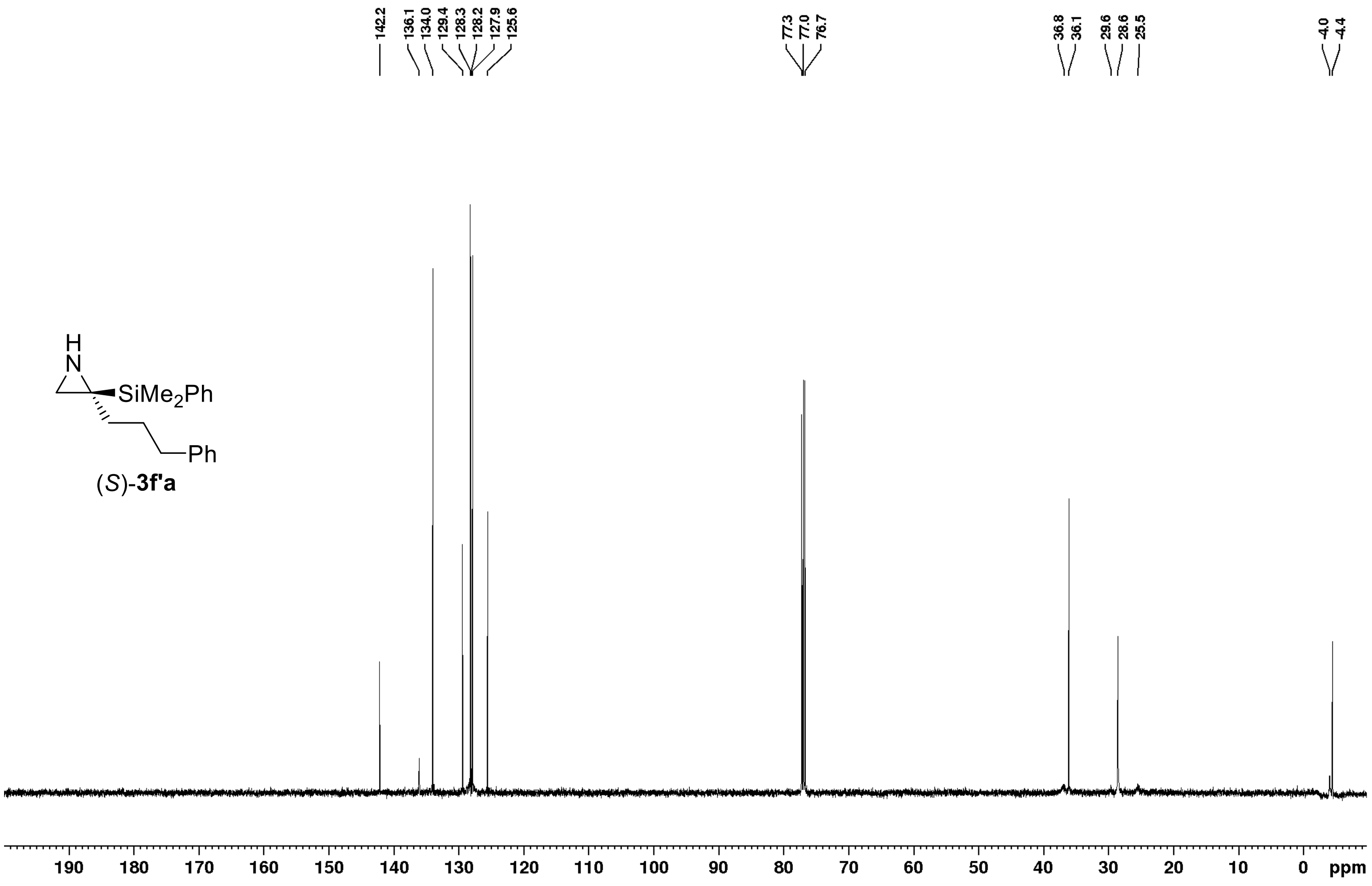

Figure S128. **^{1}H/^{29}Si HMQC NMR** spectrum (500/99 MHz, $CDCl_3$, 298 K, optimized for J = 7 Hz) of **(*S*)-2-(dimethyl(phenyl)silyl)-2-(3-phenylpropyl)aziridine** [(*S*)-**3f'a**].

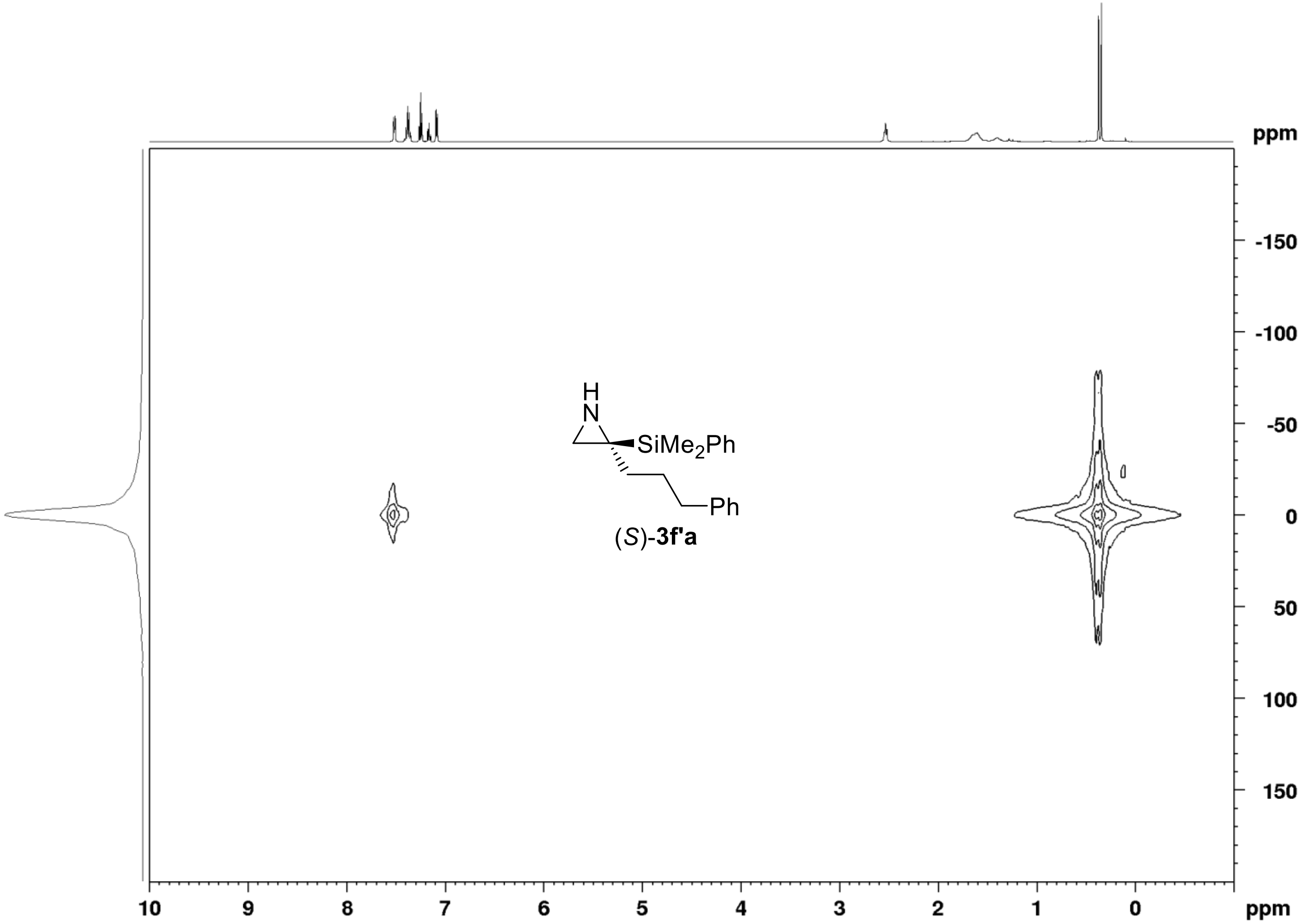

Figure S129. **^{1}H NMR** (500 MHz, $CDCl_3$, 298 K) of **(S)-2-(4-chlorobutyl)-2-(dimethyl(phenyl)silyl)aziridine** [(*S*)-**3g'a**].

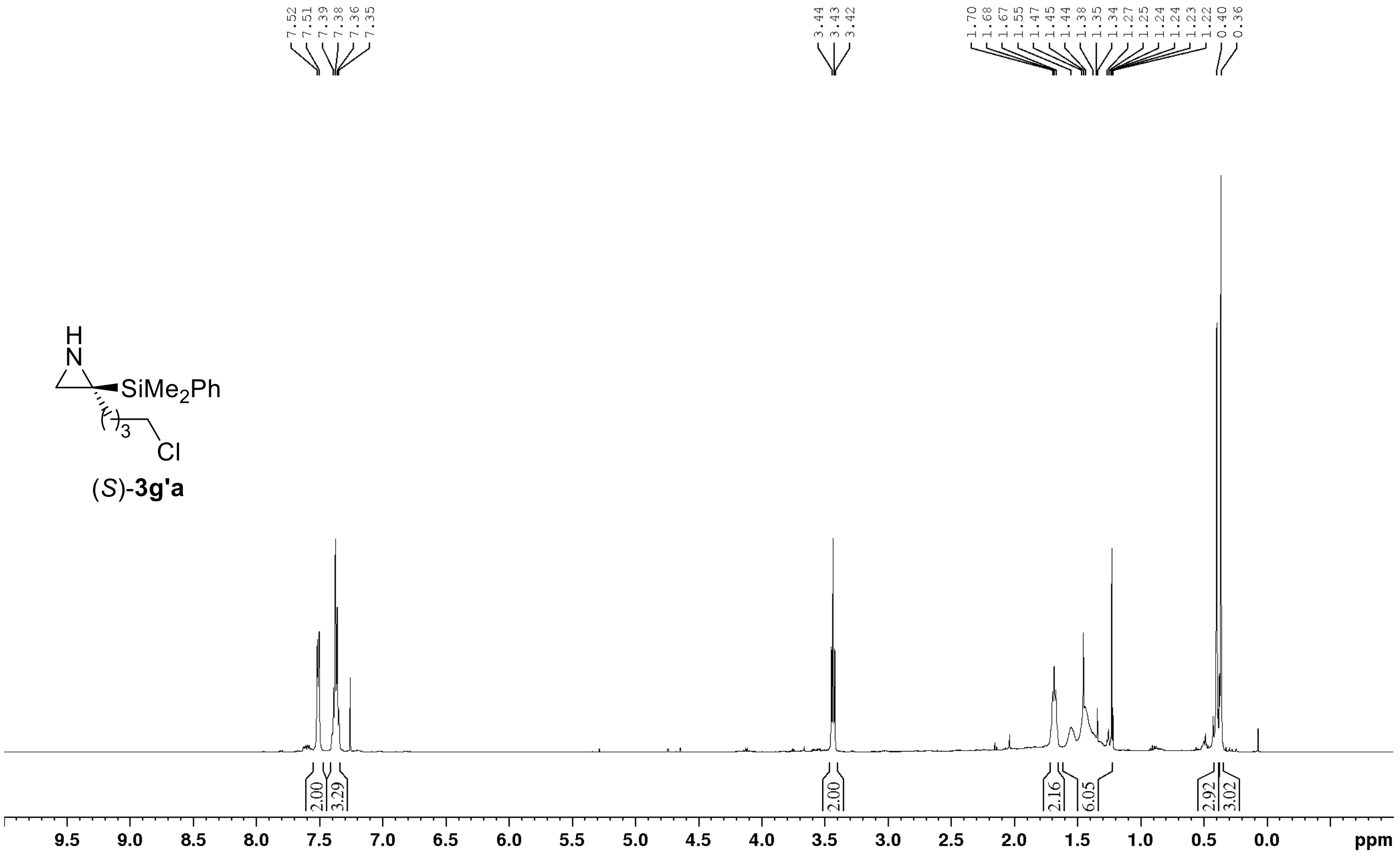

Figure S130. **^{13}C NMR** (126 MHz, $CDCl_3$, 298 K) of **(S)-2-(4-chlorobutyl)-2-(dimethyl(phenyl)silyl)aziridine** [(*S*)-**3g'a**].

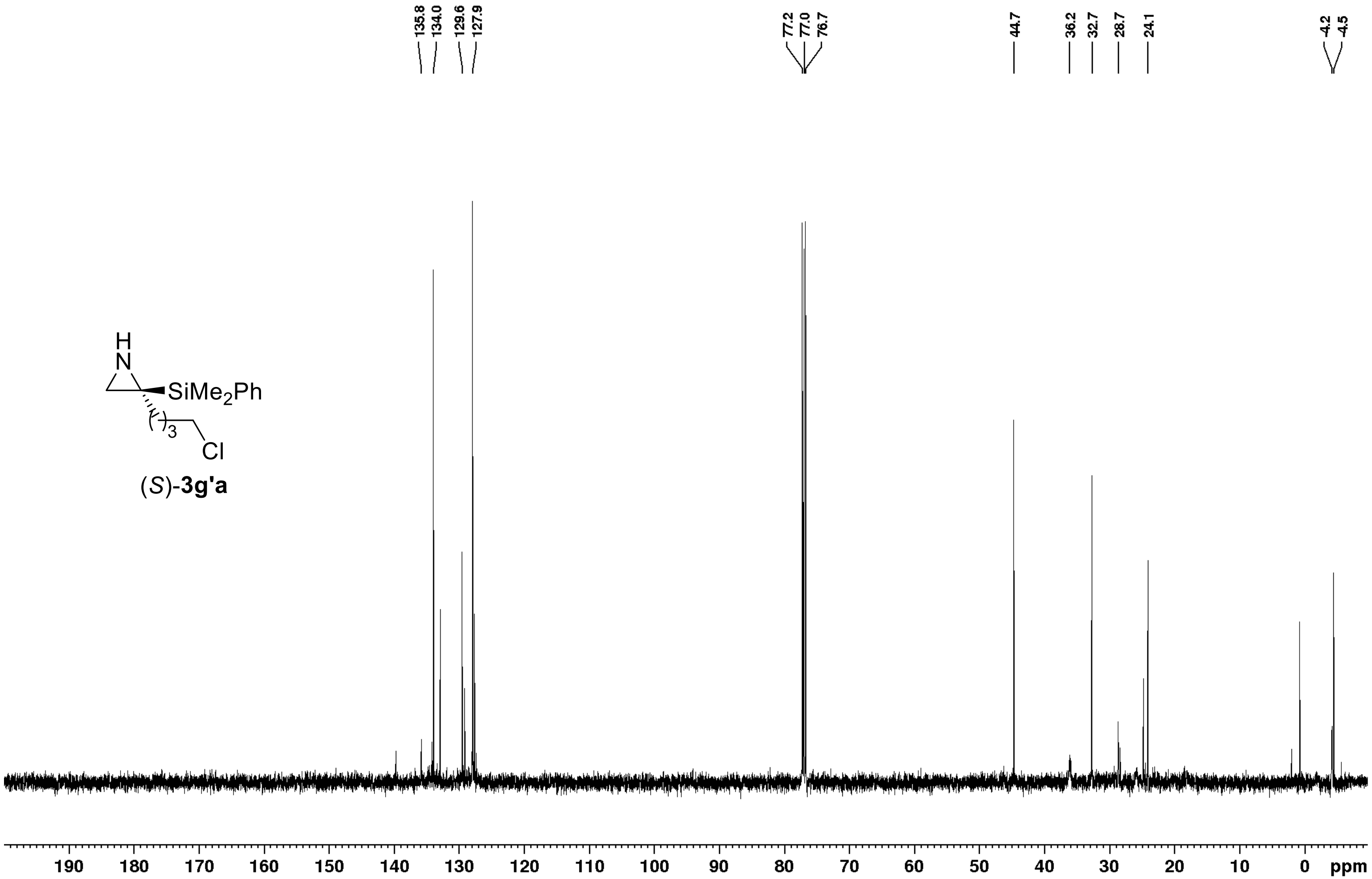

(*S*)-**3g'a**

Figure S131. **$^{1}H/^{29}Si$ HMQC NMR** spectrum (500/99 MHz, $CDCl_3$, 298 K, optimized for *J* = 7 Hz) of **(S)-2-(4-chlorobutyl)-2-(dimethyl(phenyl)silyl)aziridine** [(*S*)-**3g'a**].

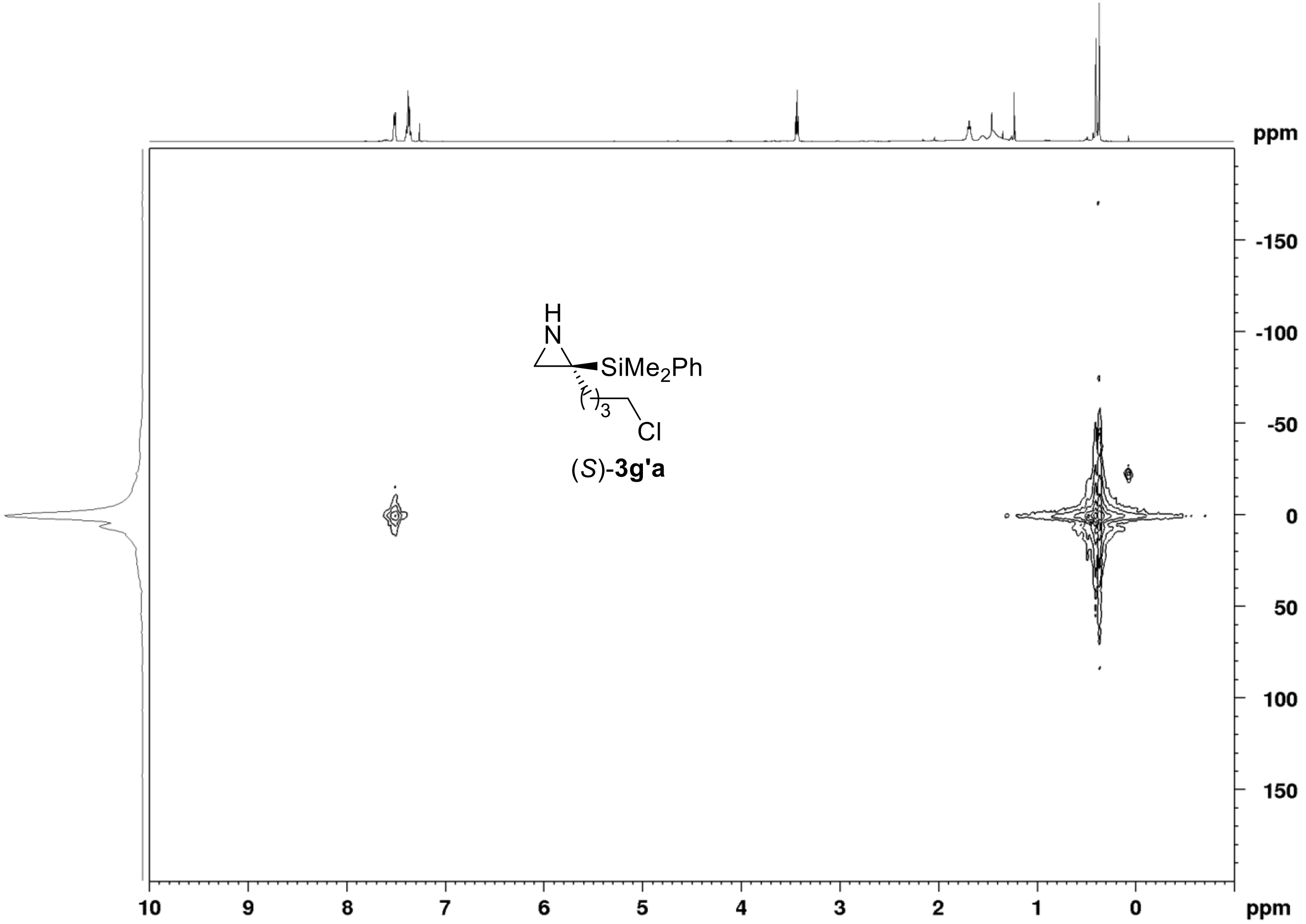

Figure S132. **^{1}H NMR** (500 MHz, $CDCl_3$, 298 K) of **(*S*)-2-phenyl-2-(triethylsilyl)aziridine** [(*S*)-**3ab**].

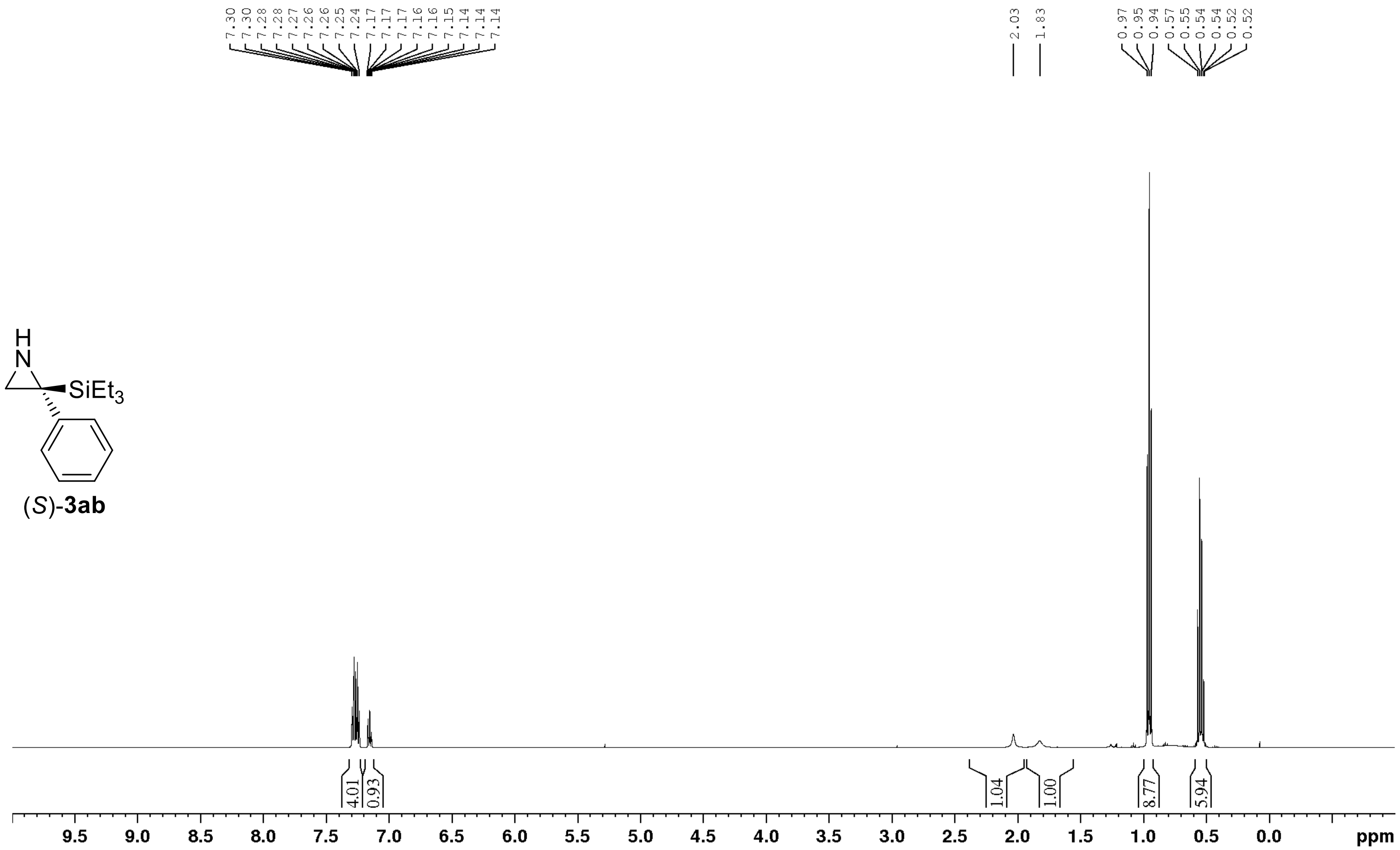

Figure S133. **^{13}C NMR** (126 MHz, $CDCl_3$, 298 K) of **(*S*)-2-phenyl-2-(triethylsilyl)aziridine** [(*S*)-**3ab**].

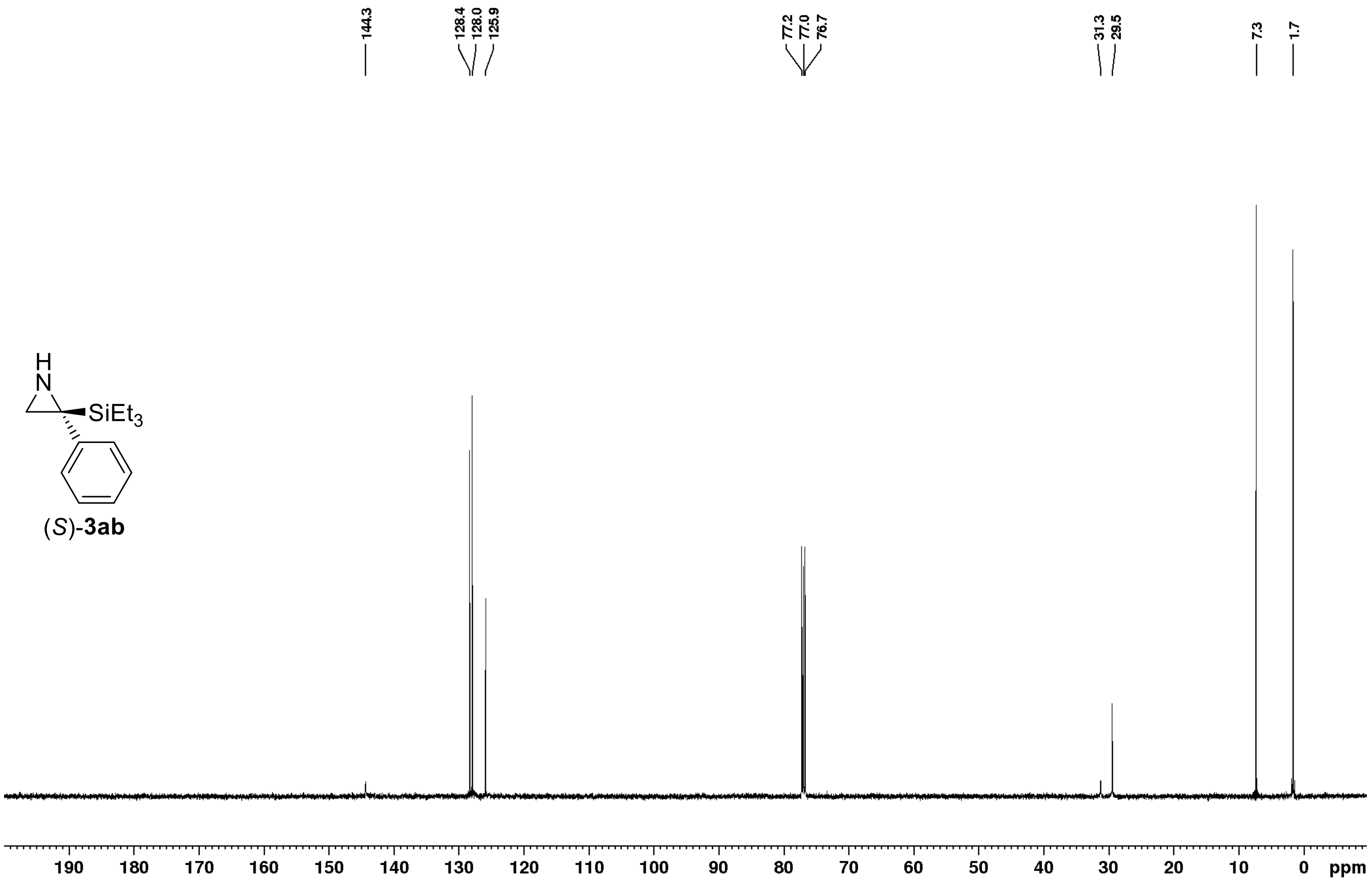

Figure S134. **^{1}H/^{29}Si HMQC NMR** spectrum (500/99 MHz, $CDCl_3$, 298 K, optimized for *J* = 7 Hz) of **(*S*)-2-phenyl-2-(triethylsilyl)aziridine** [(*S*)-**3ab**].

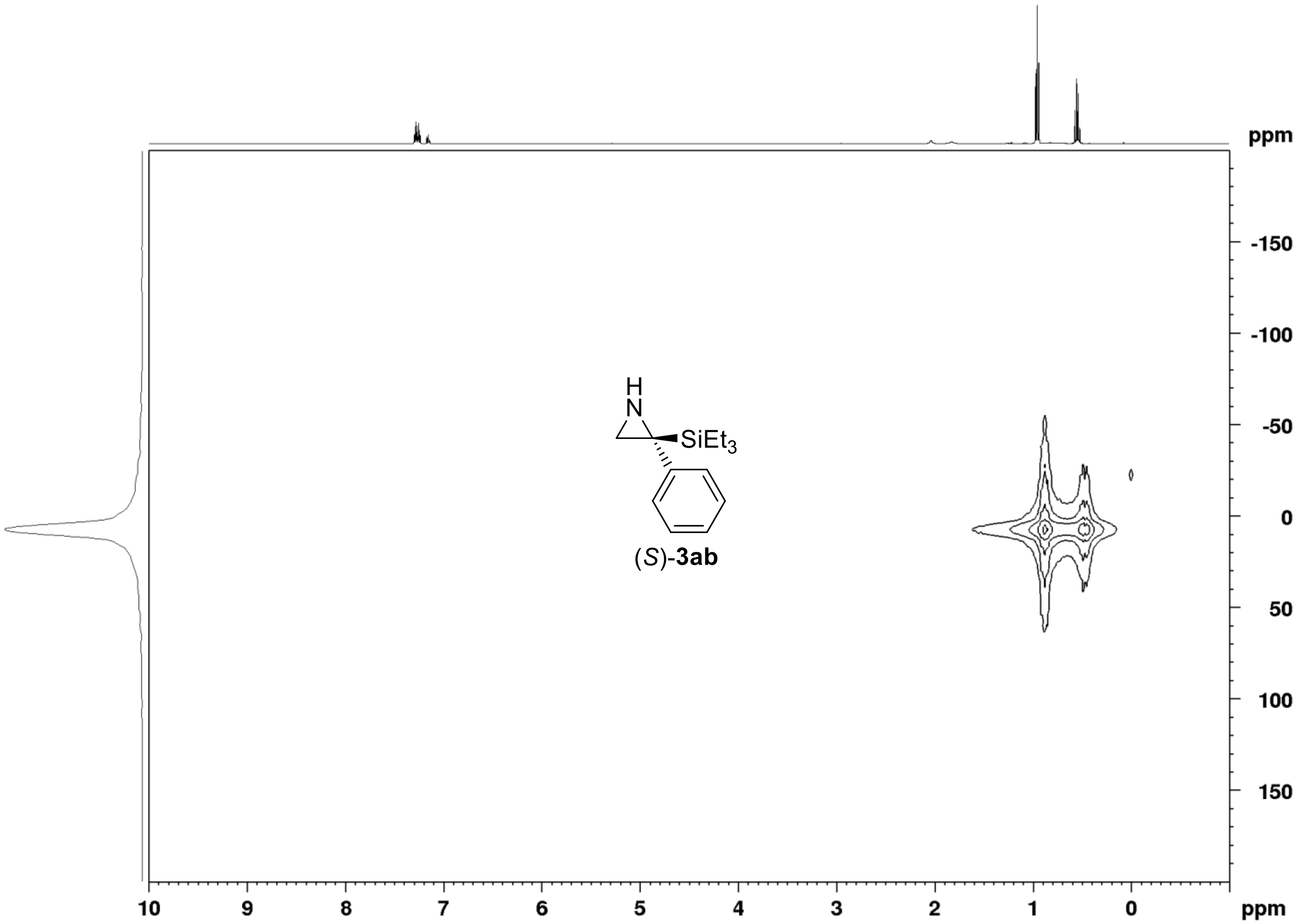

Figure S135. **^{1}H NMR** (500 MHz, $CDCl_3$, 298 K) of **(*S*)-5-(dimethyl(phenyl)silyl)-2-(3,5-dinitrophenyl)-5-phenyl-4,5-dihydrooxazole** [(*S*)-**7**].

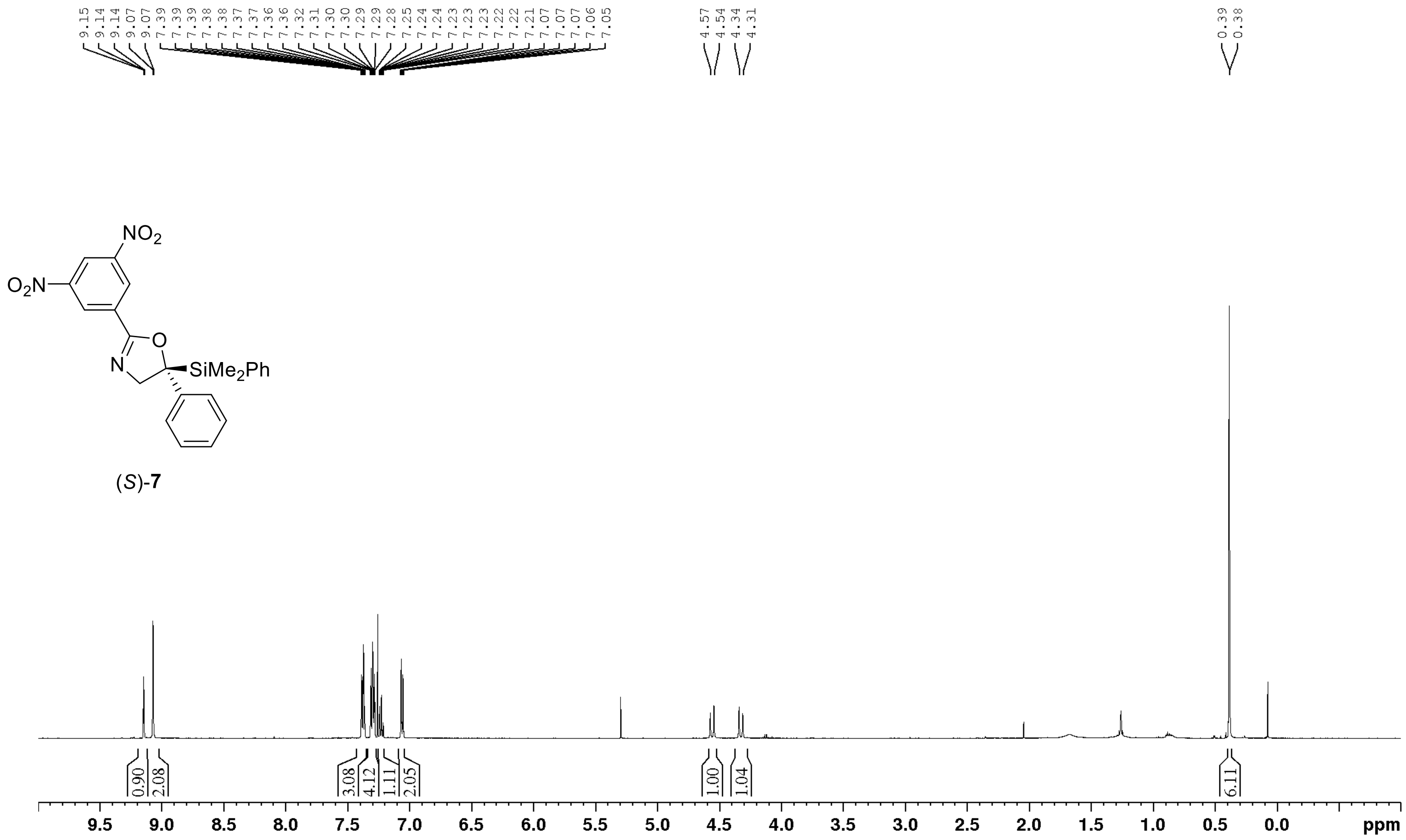

Figure S136. **^{13}C NMR** (126 MHz, $CDCl_3$, 298 K) of **(*S*)-5-(dimethyl(phenyl)silyl)-2-(3,5-dinitrophenyl)-5-phenyl-4,5-dihydrooxazole** [(*S*)-**7**].

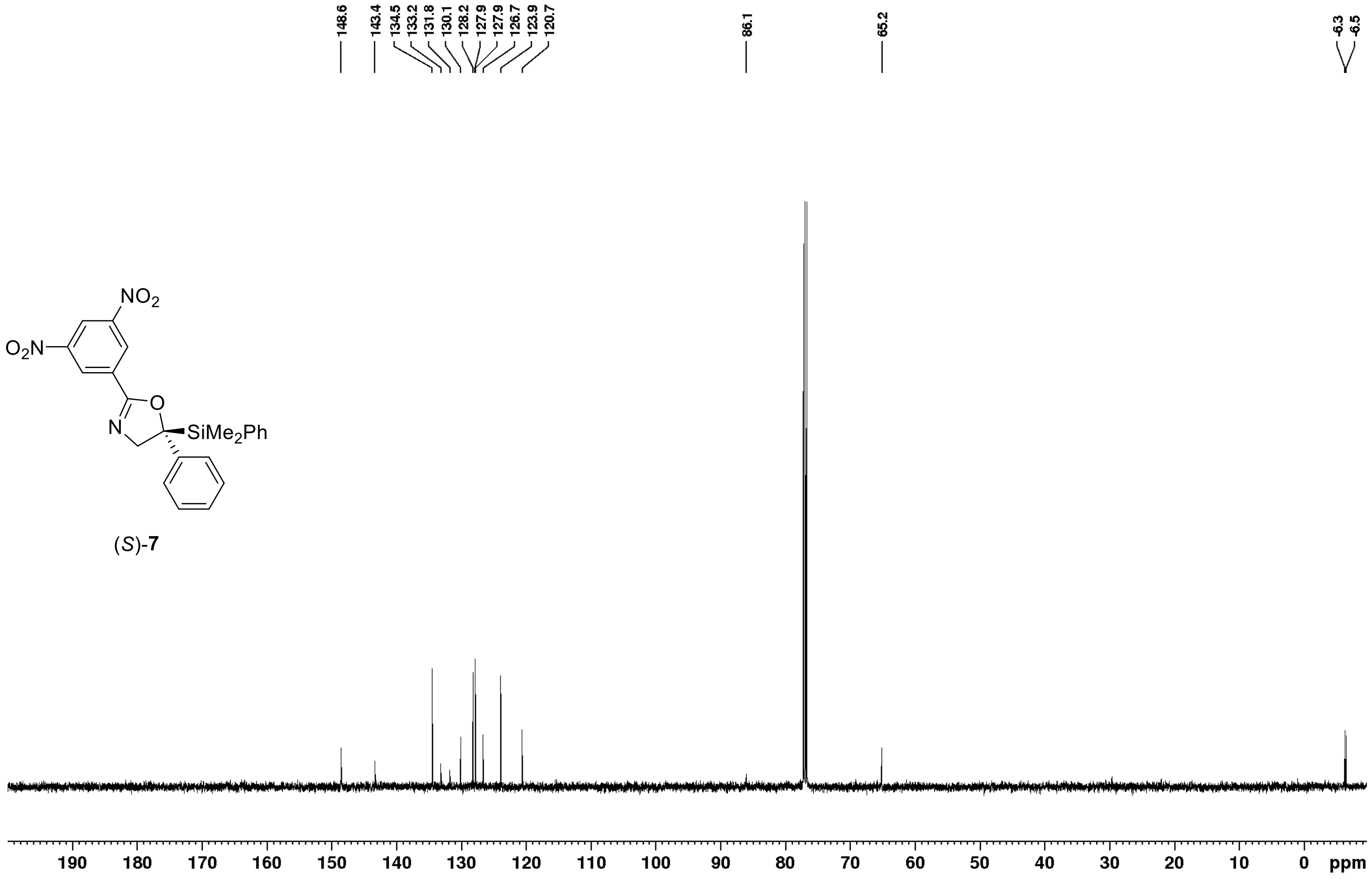

Figure S137. **^{29}Si DEPT NMR** (99 MHz, $CDCl_3$, 298 K) of **(*S*)-5-(dimethyl(phenyl)silyl)-2-(3,5-dinitrophenyl)-5-phenyl-4,5-dihydrooxazole** [(*S*)-**7**].

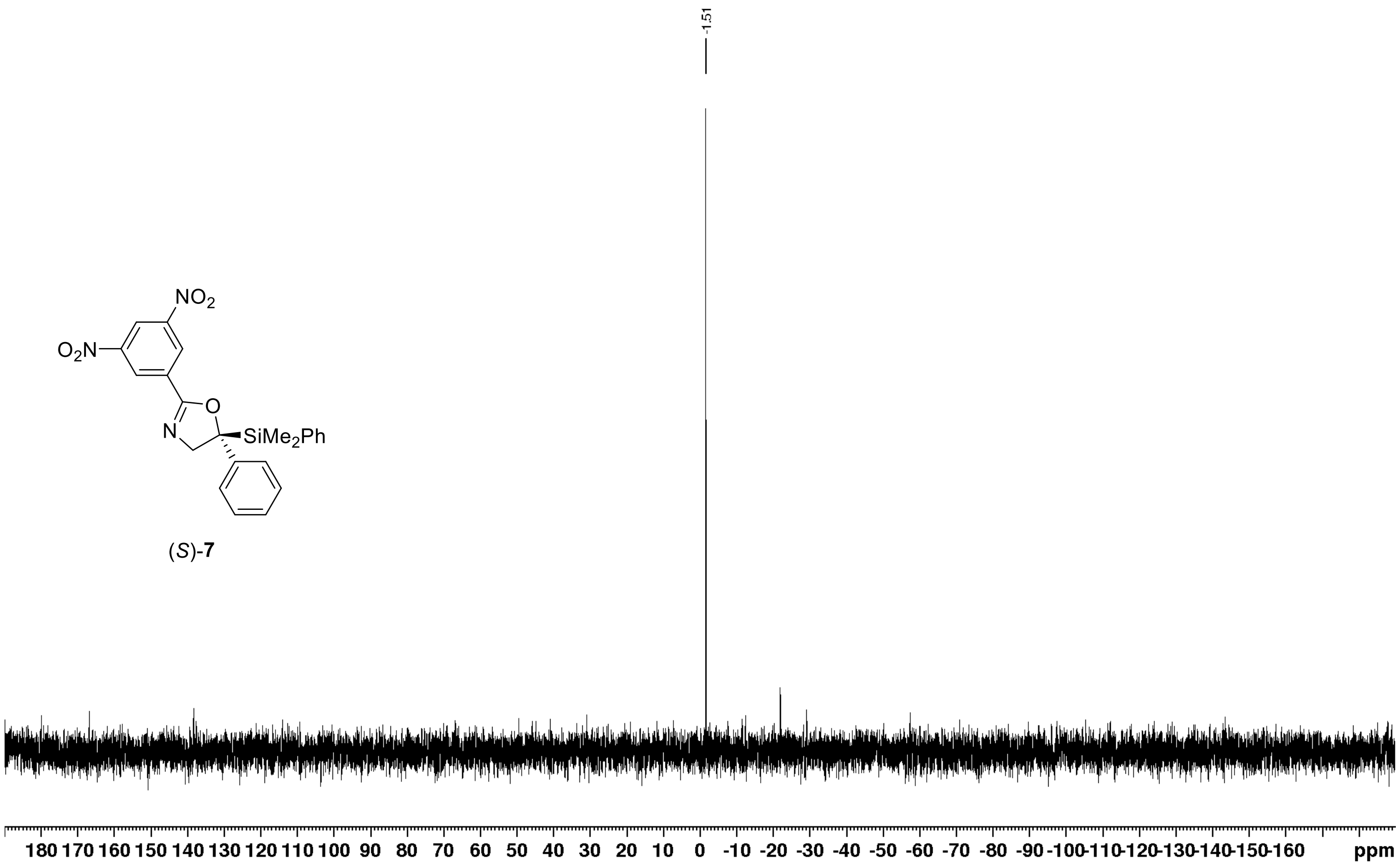

Figure S138. **^{1}H NMR** (500 MHz, $CDCl_3$, 298 K) of **(*S*)-2-(dimethyl(phenyl)silyl)-2-phenyl-1-tosylaziridine** [(*S*)-**8**].

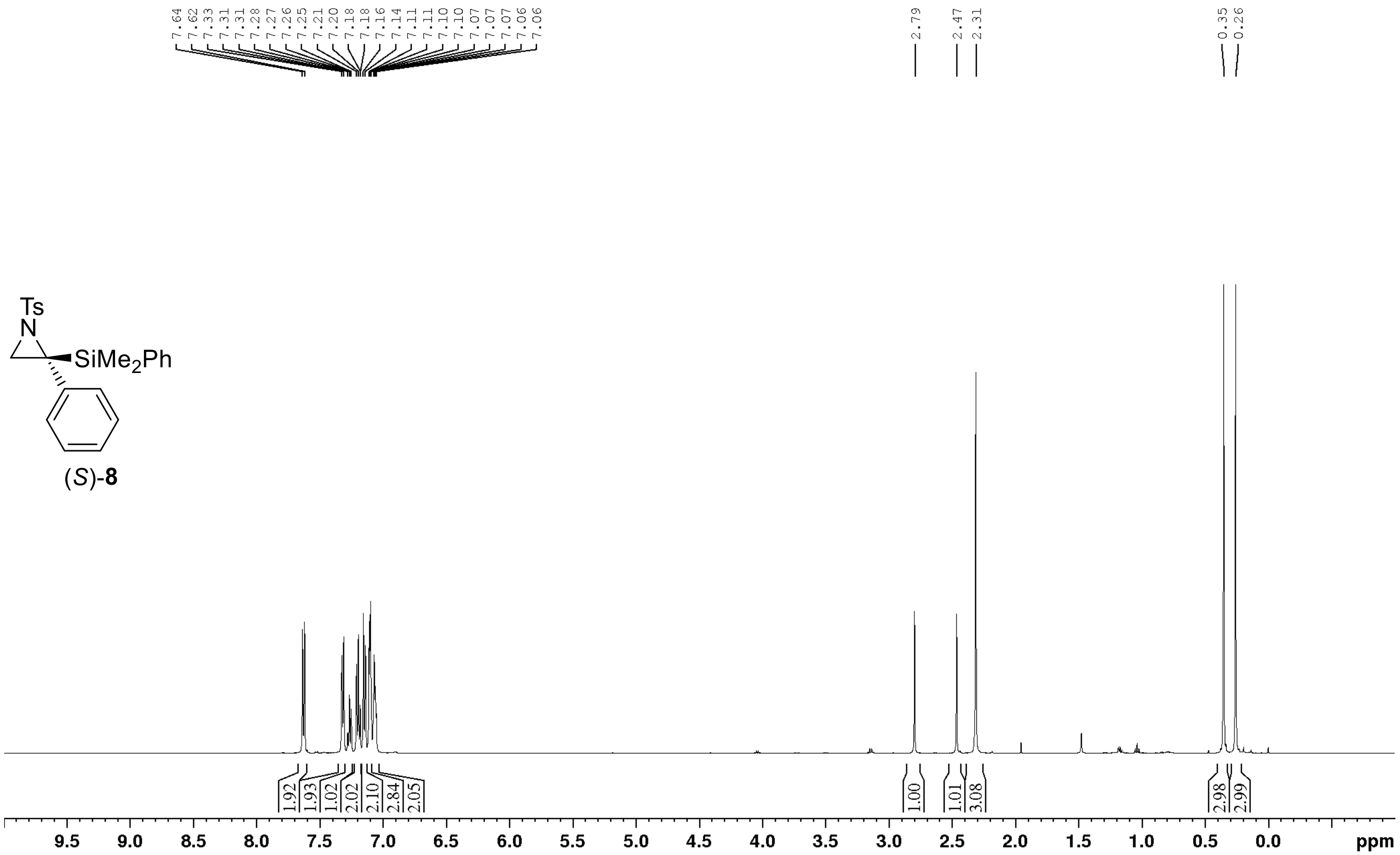

Figure S139. **^{13}C NMR** (126 MHz, $CDCl_3$, 298 K) of **(*S*)-2-(dimethyl(phenyl)silyl)-2-phenyl-1-tosylaziridine** [(*S*)-**8**].

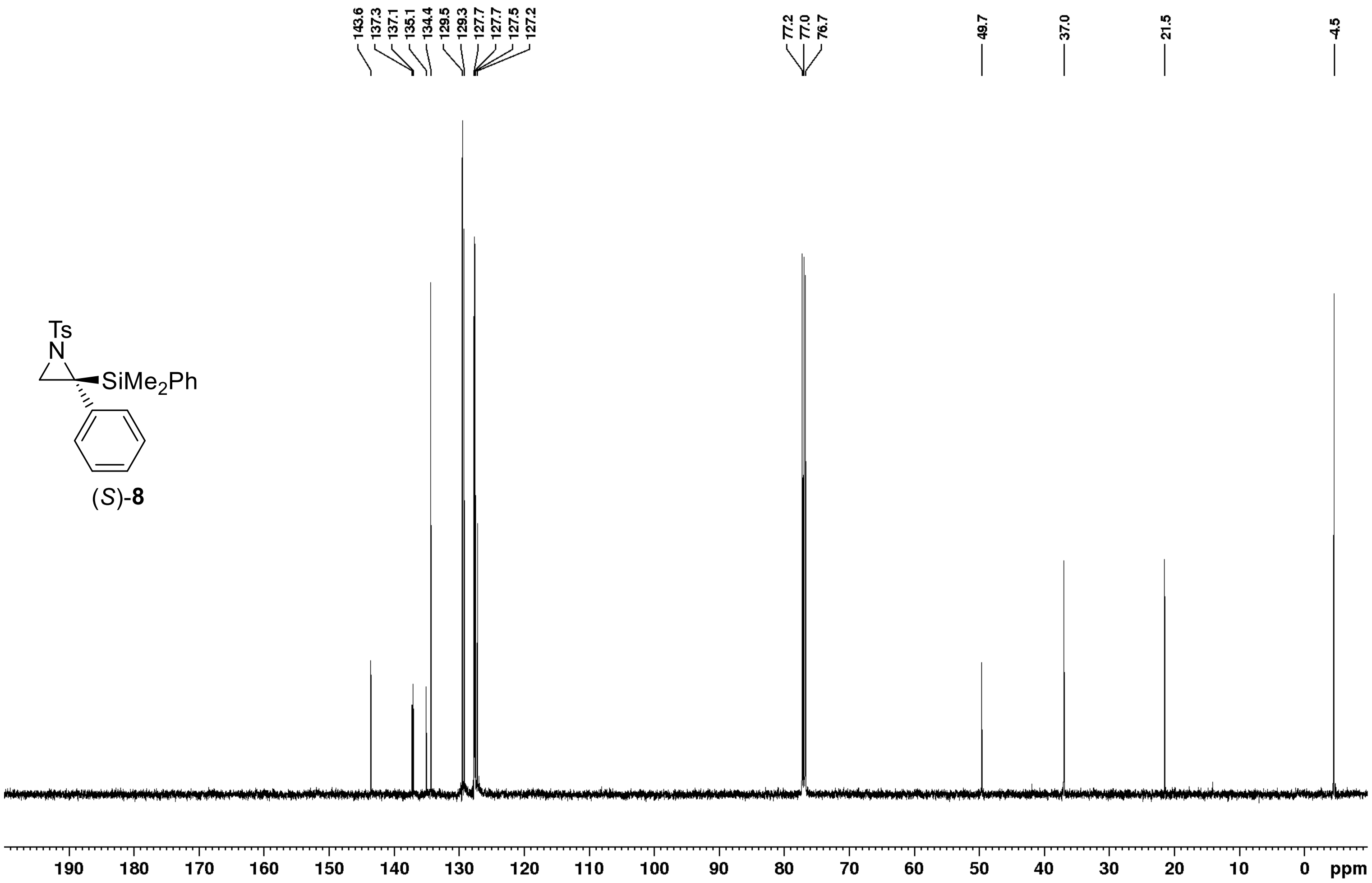

(*S*)-**8**

Figure S140. ^{29}**Si DEPT NMR** (99 MHz, $CDCl_3$, 298 K) of **(*S*)-2-(dimethyl(phenyl)silyl)-2-phenyl-1-tosylaziridine** [(*S*)-**8**].

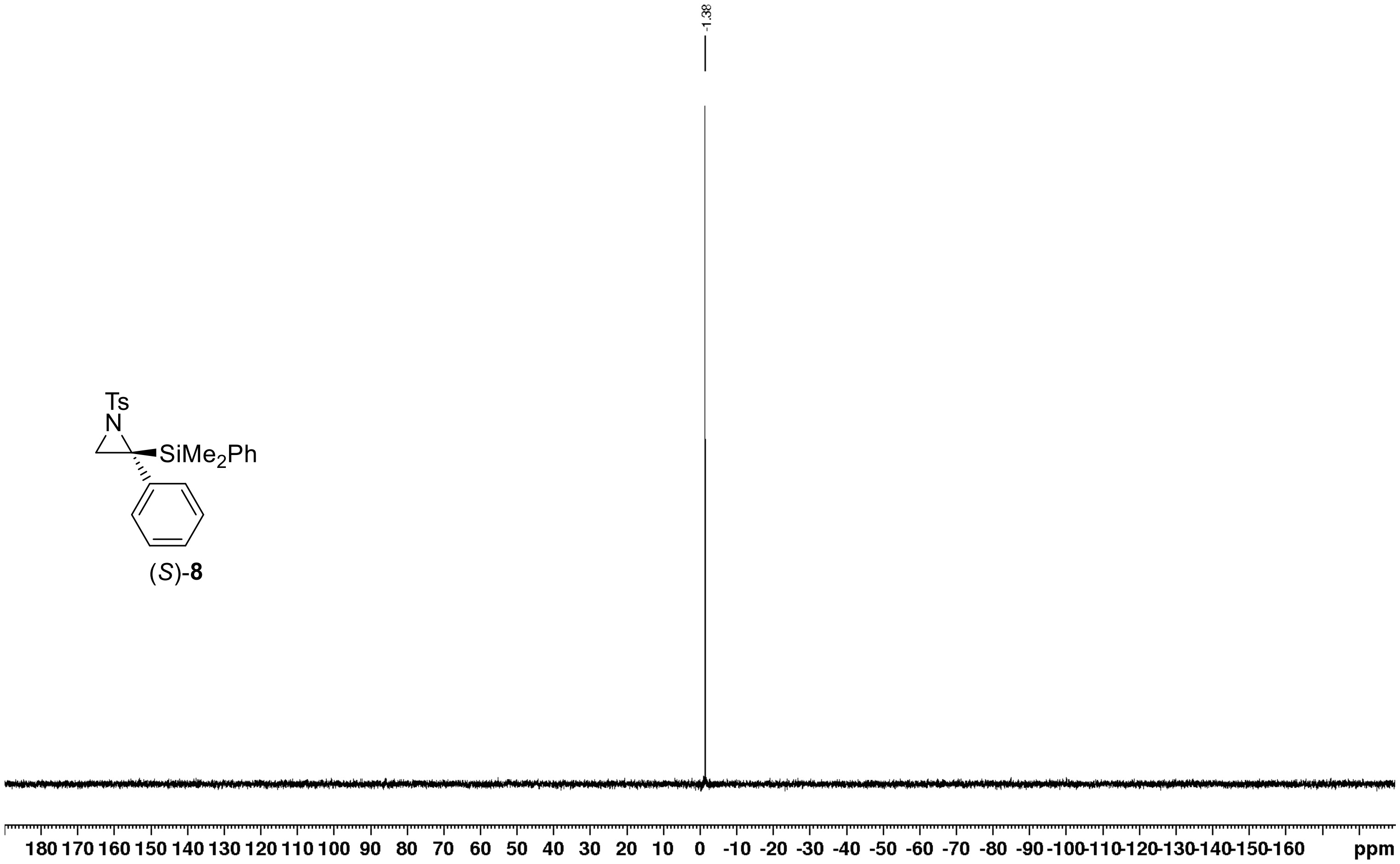

Figure S141. **^{1}H NMR** (500 MHz, $CDCl_3$, 298 K) of **(*S*)-N-(2-(dimethyl(phenyl)silyl)-2-phenylethyl)-4-methylbenzenesulfonamide** [(*S*)-**9**].

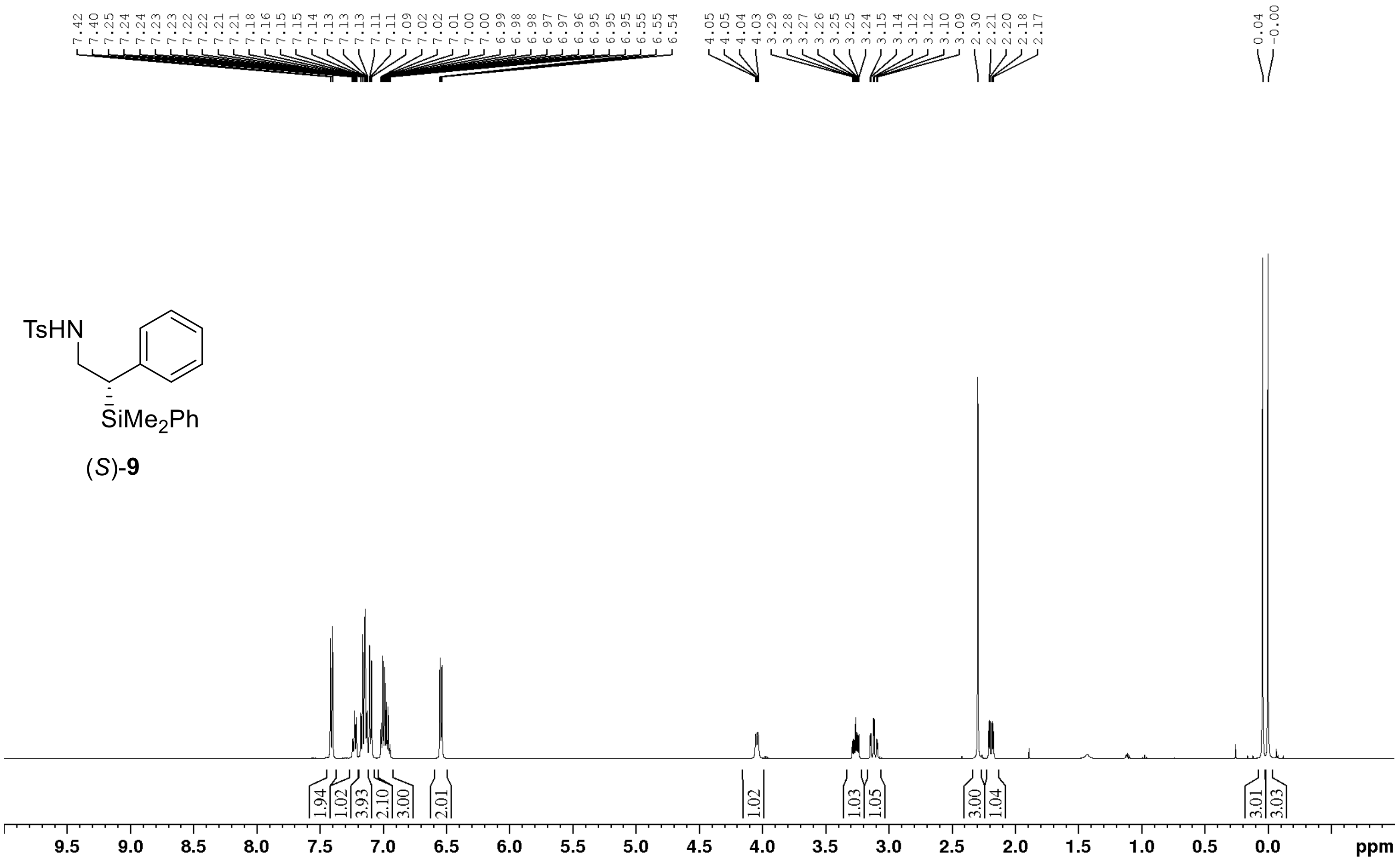

Figure S142. **^{13}C NMR** (126 MHz, $CDCl_3$, 298 K) of **(*S*)-N-(2-(dimethyl(phenyl)silyl)-2-phenylethyl)-4-methylbenzenesulfonamide** [(*S*)-**9**].

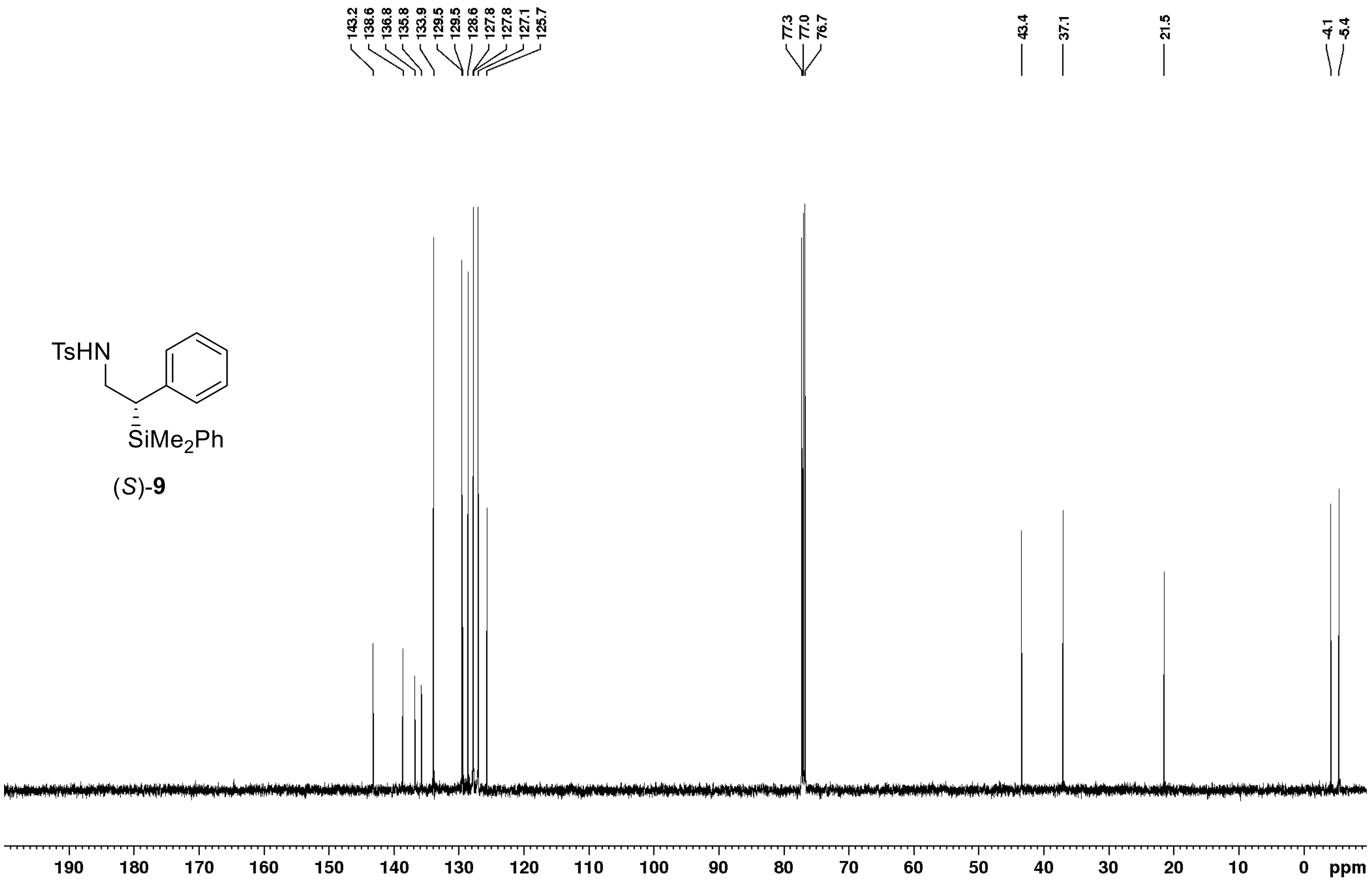

Figure S143. **^{29}Si DEPT NMR** (99 MHz, $CDCl_3$, 298 K) of **(*S*)-N-(2-(dimethyl(phenyl)silyl)-2-phenylethyl)-4-methylbenzenesulfonamide** [(*S*)-**9**].

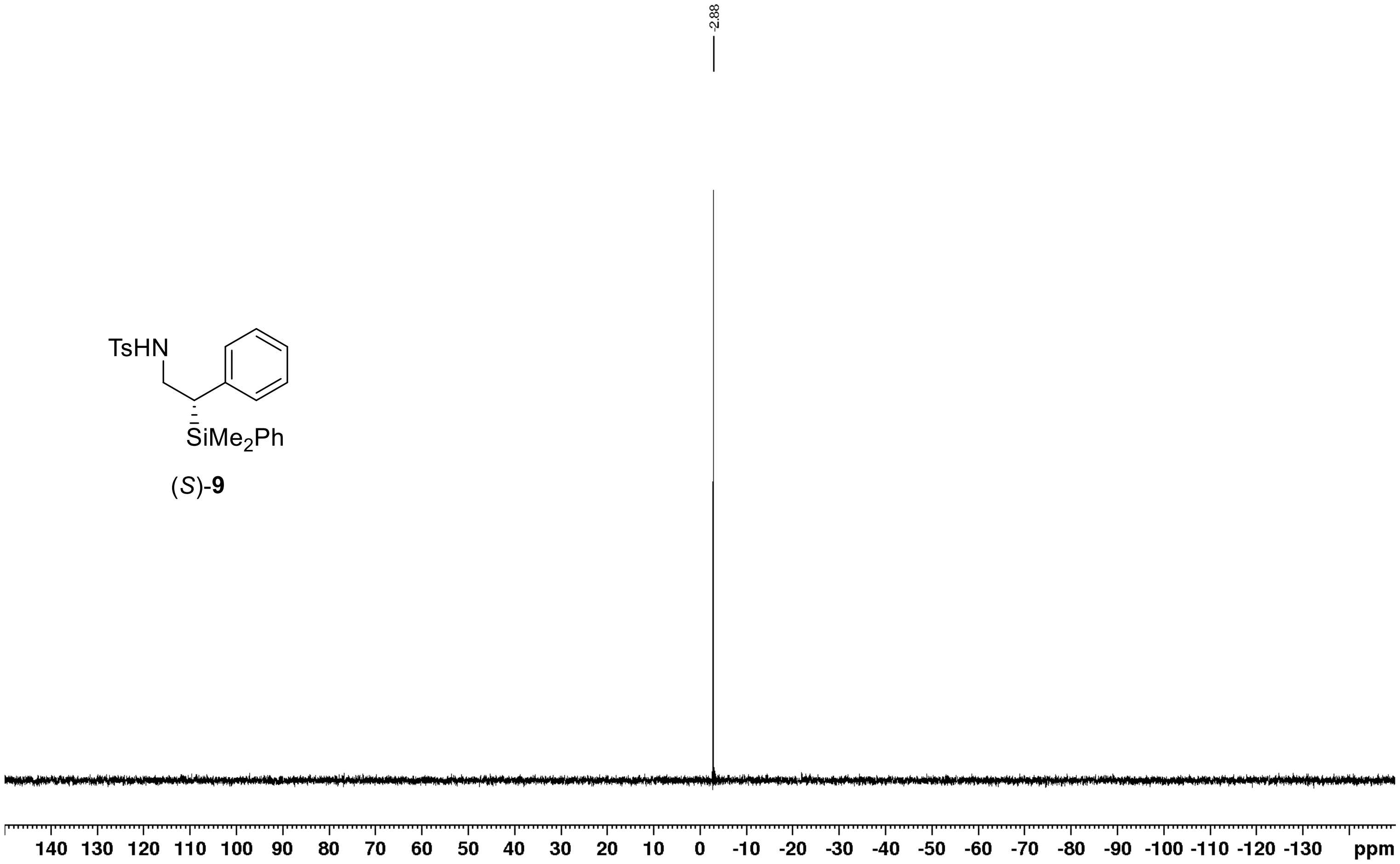

11 References

[S1] R. K. Harris, E. D. Becker, S. M. Cabral de Menezes, R. Goodfellow, P. Granger, *Pure Appl. Chem.* **2001**, *73*, 1795–1818.

[S2] Agilent CrysAlis PRO, **2012**, Agilent Technologies, Yarnton, UK.

[S3] G. M. Sheldrick, *Acta Crystallogr., Sect. A.* **1990**, *46*, 467–473.

[S4] G. M. Sheldrick, *Acta Crystallogr., Sect. A.* **2008**, *64*, 112–122.

[S5] Cambridge Crystallographic Data Centre: http://www.ccdc.cam.ac.uk/Solutions/CSDSystem/Pages/Mercury.aspx.

[S6] a) M. Suginome, T. Matsuda, Y. Ito, *Organometallics* **2000**, *19*, 4647–4649; b) T. A. Boebel, J. F. Hartwig, *Organometallics* **2008**, *27*, 6013–6019.

[S7] a) Y.-F. Wang, M. Hu, H. Hayashi, B. Xing, S. Chiba, *Org. Lett.* **2016**, *18*, 992–995; b) B. Nie, W. Wu, W. Zeng, Q. Ren, J. Zhang, Y. Zhang, H. Jiang, *Adv. Synth.Catal.* **2020**, *362*, 1362–1369.

[S8] H.-J. Zhang, Y.-C. Xie, L. Yin, *Nat. Commun.* **2019**, *10*, 1699.

[S9] a) B. M. Trost, C. Zhu, *Org. Lett.* **2020**, *22*, 9683–9687; b) L. Chen, H. Li, P. Li, L. wang, *Org. Lett.* **2016**, *18*, 3646–3649; c) S. Nakamura, D. Hayama, *Angew. Chem. Int. Ed.* **2017**, 56, 8785–8789; *Angew. Chem.* **2017**, *129*, 8911–8915.

[S10] S. Nakamura, D. Hayama, M. Miura, T. Hatanaka, Y. Funahashi, *Org. Lett.* **2018**, *20*, 856–859.

[S11] M. C. de Ceglie, B. Musio, F. Affortunato, A. Moliterni, A. Altomare, S. Florio, R. Luisi, *Chem. Eur. J.* **2011**, *17*, 286–296.
