# Supplementary material for: Copper‐Catalyzed Highly Enantioselective Addition of a Silicon Nucleophile to 3‐Substituted 2H‐Azirines Using an Si−B Reagent
Source: Angew Chem Int Ed Engl. 2023 Jan 9;62(7):e202215032. doi: 10.1002/anie.202215032 (PMC10108078; doi:10.1002/anie.202215032)

checkCIF/PLATON report

You have not supplied any structure factors. As a result the full set of tests cannot be run.

THIS REPORT IS FOR GUIDANCE ONLY. IF USED AS PART OF A REVIEW PROCEDURE FOR PUBLICATION, IT SHOULD NOT REPLACE THE EXPERTISE OF AN EXPERIENCED CRYSTALLOGRAPHIC REFEREE.

No syntax errors found.   CIF dictionary   Interpreting this report

Datablock: CU-4820

```
Bond precision:   C-C = 0.0052 A          Wavelength=1.54184

Cell:              a=9.6984(4)     b=7.8927(3)         c=10.2853(5)
                   alpha=90        beta=96.109(4)      gamma=90
Temperature:       150 K

                 Calculated                       Reported
Volume           782.83(6)                        782.83(6)
Space group      P 21                             P 21
Hall group       P 2yb                            P 2yb
Moiety formula   C17 H18 N O2 Si                  ?
Sum formula      C17 H18 N O2 Si                  C17 H18 N O2 Si
Mr               296.41                           296.41
Dx,g cm-3        1.258                            1.257
Z                2                                2
Mu (mm-1)        1.352                            1.352
F000             314.0                            314.0
F000'            315.34
h,k,lmax         11,9,12                          11,9,12
Nref             2815[ 1519]                      2589
Tmin,Tmax        0.773,0.778                      0.146,1.000
Tmin'            0.679

Correction method= # Reported T Limits: Tmin=0.146 Tmax=1.000
AbsCorr = MULTI-SCAN

Data completeness= 1.70/0.92       Theta(max)= 67.420

                                                   wR2(reflections)=
R(reflections)= 0.0389( 2519)                      0.1078( 2589)

S = 1.068                  Npar= 196
```

The following ALERTS were generated. Each ALERT has the format
test-name_ALERT_alert-type_alert-level.
Click on the hyperlinks for more details of the test.

Alert level C

```
PLAT241_ALERT_2_C High   'MainMol' Ueq as Compared to Neighbors of        C9 Check
PLAT340_ALERT_3_C Low Bond Precision on  C-C Bonds ...............    0.00521 Ang.
PLAT420_ALERT_2_C D-H Bond Without Acceptor  N1       --H1       .     Please Check
```

Alert level G

```
PLAT398_ALERT_2_G Deviating  C-O-C   Angle From 120 for O1       .      104.4 Degree
PLAT398_ALERT_2_G Deviating  C-O-C   Angle From 120 for O2       .      104.7 Degree
PLAT791_ALERT_4_G Model has Chirality at C1          (Sohnke SpGr)          S Verify
PLAT883_ALERT_1_G No Info/Value for _atom_sites_solution_primary .     Please Do !
PLAT941_ALERT_3_G Average HKL Measurement Multiplicity ..........        3.2 Low
PLAT967_ALERT_5_G Note: Two-Theta Cutoff Value in Embedded .res ..      135.0 Degree
```

0 **ALERT level A** = Most likely a serious problem - resolve or explain
0 **ALERT level B** = A potentially serious problem, consider carefully
3 **ALERT level C** = Check. Ensure it is not caused by an omission or oversight
6 **ALERT level G** = General information/check it is not something unexpected

1 ALERT type 1 CIF construction/syntax error, inconsistent or missing data
4 ALERT type 2 Indicator that the structure model may be wrong or deficient
2 ALERT type 3 Indicator that the structure quality may be low
1 ALERT type 4 Improvement, methodology, query or suggestion
1 ALERT type 5 Informative message, check

It is advisable to attempt to resolve as many as possible of the alerts in all categories. Often the minor alerts point to easily fixed oversights, errors and omissions in your CIF or refinement strategy, so attention to these fine details can be worthwhile. In order to resolve some of the more serious problems it may be necessary to carry out additional measurements or structure refinements. However, the purpose of your study may justify the reported deviations and the more serious of these should normally be commented upon in the discussion or experimental section of a paper or in the "special_details" fields of the CIF. checkCIF was carefully designed to identify outliers and unusual parameters, but every test has its limitations and alerts that are not important in a particular case may appear. Conversely, the absence of alerts does not guarantee there are no aspects of the results needing attention. It is up to the individual to critically assess their own results and, if necessary, seek expert advice.

Publication of your CIF in IUCr journals

A basic structural check has been run on your CIF. These basic checks will be run on all CIFs submitted for publication in IUCr journals (*Acta Crystallographica*, *Journal of Applied Crystallography*, *Journal of Synchrotron Radiation*); however, if you intend to submit to *Acta Crystallographica Section C* or *E* or *IUCrData*, you should make sure that full publication checks are run on the final version of your CIF prior to submission.

Publication of your CIF in other journals

Please refer to the *Notes for Authors* of the relevant journal for any special instructions relating to CIF submission.

PLATON version of 18/05/2022; check.def file version of 17/05/2022

Datablock CU-4820 - ellipsoid plot

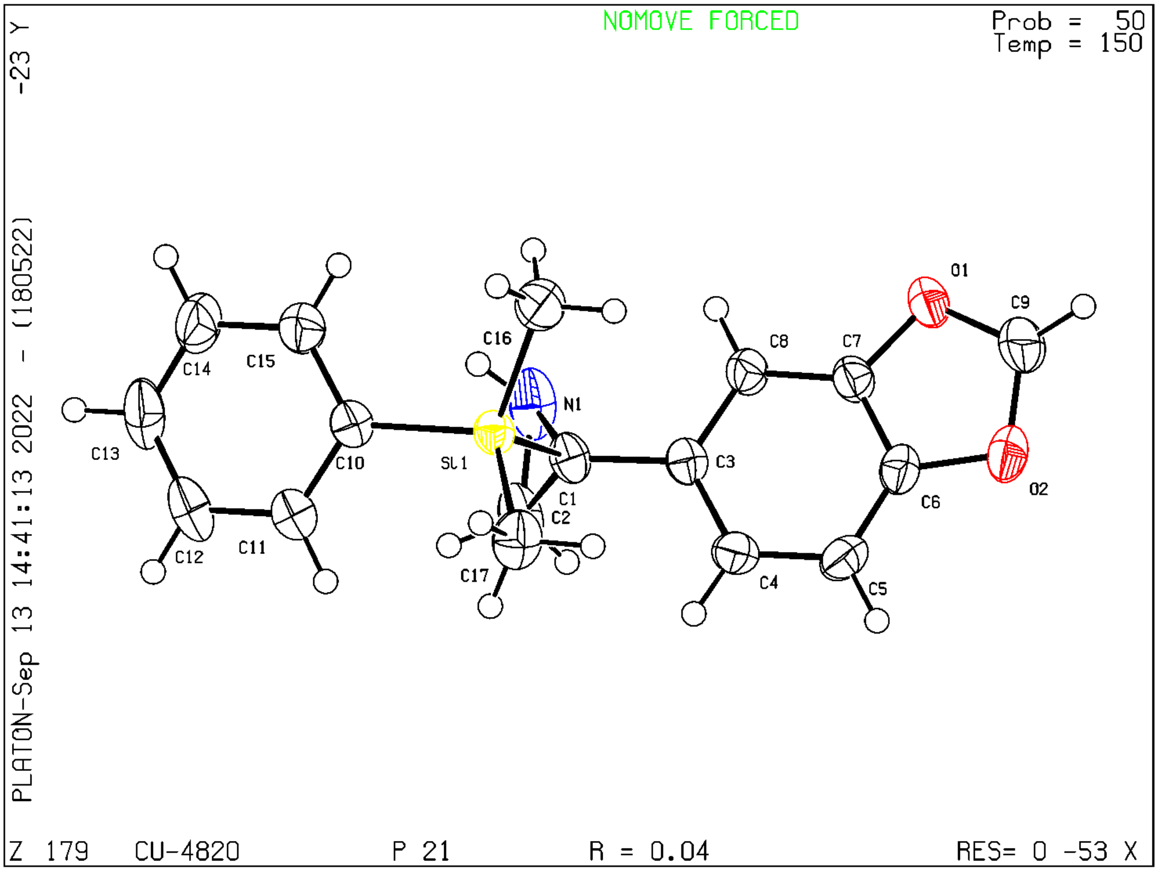

Supplement: Supplementary file 2 — Supporting Information [file ANIE-62-0-s003.pdf]
